# Evidence-Based Translation for the Genomic Responses of Murine Models for the Study of Human Immunity

**DOI:** 10.1371/journal.pone.0118017

**Published:** 2015-02-13

**Authors:** Junhee Seok

**Affiliations:** School of Electrical Engineering, Korea University, Seoul, South Korea

## Abstract

Murine models are an essential tool to study human immune responses and related diseases. However, the use of traditional murine models has been challenged by recent systemic surveys that show discordance between human and model immune responses in their gene expression. This is a significant problem in translational biomedical research for human immunity. Here, we describe evidence-based translation (EBT) to improve the analysis of genomic responses of murine models in the translation to human immune responses. Based on evidences from prior experiments, EBT introduces pseudo variances, penalizes gene expression changes in a model experiment, and finally detects false positive translations of model genomic responses that poorly correlate with human responses. Demonstrated over multiple data sets, EBT significantly improves the agreement of overall responses (up to 56%), experiment-specific responses (up to 143%), and enriched biological contexts (up to 100%) between human and model systems. In addition, we provide the category of genes specifically benefiting from EBT and the factors affecting the performance of EBT. The overall result indicates the usefulness of the proposed computational translation in biomedical research for human immunity using murine models.

## Introduction

In modern biomedical research, murine models have extensively used for better understanding of human disease processes as well as for improving, developing and testing new drugs and therapies without risks on actual human beings [1–3]. Despite the common use of murine model systems, the translations of model experiment results into human studies have shown low success rates [4,5]. For example, to date, more than 150 clinical trials for agents developed from animal models have failed to suppress inflammatory responses in severely injured patients [6,7]. This poor translation can be partially explained by the discordance of molecular-level responses between murine model and target human systems. While a model system share phenotypic similarity with humans in a certain immunological condition, its molecular and genomic responses can be very different. It has been known that murine models have substantially different immune responses from humans in the molecular level [8]. In addition, a recent systematic survey has reported that the genomic responses of murine models poorly mimic human inflammatory diseases, in which the correlation of the genome-wide expression changes of orthologous genes is low [9].

Even with the potential drawbacks, murine models still have their own advantages in biomedical research as a way to reduce risks on human beings and costs for experiments. Moreover, accumulated experimental results using murine models should be reused for the future research. The low correlation between murine and human response mainly comes from the conventional naïve translation, which considers all model responses are human-like. It does not selectively distinguish human-like murine responses from human-unlike ones. Thus, the key challenge is the accurate and efficient translation of model responses in the contexts of target human responses.

While developing better models, which reflect both phenotypic and molecular responses [10,11], might be able to improve the use of murine model systems, the experiment results using such a model inevitably need to be translated. Pure computational models simulating human disease conditions have been suggested [12]. However, the construction of such a computational model still requires translated knowledge from model experiments. Although common interspecies responses to diseases have been investigated [13], they are not directly applicable to the translation of model responses in a specific condition. Despite the importance and unmet needs, analysis methods to improve the translation of model responses have been yet unexplored much.

In this work, we propose evidence-based translation (EBT) for the genomic responses of murine model systems to improve model-target translation. EBT regulates the significance of genomic responses of a model system by introducing pseudo variances estimated from a prior model-target experiment pair. Genes regulated by large pseudo variances are consequently considered as false positives in the translation of an independent experiment using a similar model system. As far as we know, this is the first study to computationally improve the translation of model system responses.

EBT was demonstrated over five public data sets that were found from the intensive search in the whole GEO database. We focused on murine models for human immune responses, of which genomic responses have been reported to poorly mimic human responses [9]. Through EBT, the overall and experiment-specific responses as well as enriched biological contexts of model systems showed improved concordance with those of target human systems. The overall results imply that EBT will be able to significantly improve the current translational biomedical research using model systems.

## Materials and Methods

### Evidence-based translation (EBT)

For gene expression analysis of a model experiment (test experiment), EBT penalizes genes of which responses are different between the model and target systems in a prior experiment (training experiment) by introducing pseudo variances. The pseudo variances are estimated from the training experiment, and added to the variances of expression changes in the test experiment. As a result, EBT limits the significance of gene expression changes in the test experiment by the additional pseudo variances. In this work, we focus on EBT for two-class problems, which are common in biomedical experiments.

Pseudo variances are calculated by two steps. First, for the genes with the same direction of expression changes in the model and target systems, pseudo variances are directly estimated by matching the modified z-scores of the two systems. Then, in the second step, the pseudo variances of the rest of the genes are inferred from a linear model fitted from the pseudo variances of the first step.

For the first step, let *f*_*m*_ be the fold change of average gene expression between two classes in a prior model system experiment, and *f*_*t*_ be that of a corresponding target system. Also let *s*_*m*_ and *s*_*t*_ be the within-class standard deviations of gene expression levels of model and target systems in the prior experiment, respectively. In a two-class problem, a within-class standard deviation *s* is calculated by

$$s=\sqrt{\frac{(n_{1}-1)s_{1}^{2}+(n_{2}-1)s_{2}^{2}}{n_{1}+n_{2}-2}},$$

where *s*_1_ and *s*_2_ are the standard deviations of gene expression of two classes, and *n*_1_ and *n*_2_ are the numbers of samples in two classes. For genes with the same direction of changes between model and target systems, the non-negative pseudo variance *α* is obtained by minimizing the difference between the modified z-scores of the model and target systems,

$$\alpha^{*}=\underset{\alpha :\alpha \geq 0}{\text{arg}\;\text{min}}{\left(\frac{{\left(\left|f_{m}\right|-\delta \right)}_{+}+\delta }{s_{m}+\alpha }-\frac{{\left(\left|f_{t}\right|-\delta \right)}_{+}+\delta }{s_{t}}\right)}^{2},$$

where (*x*)_+_ = max(*x*,0) and *δ* is a pre-defined offset for the lower bound of expression fold changes. $\left({\left(f-\delta \right)}_{+}+\delta \right)/s$ is a modified z-score because the absolute fold change less than *δ* is truncated to *δ*. For the demonstration in this work, we used *δ* = 0.25.

The estimation of the first step is applicable only for the genes with the same direction of changes between the model and target responses. For the rest of the genes with opposite changes, we infer pseudo variances through a linear model. Logged pseudo variances are linearly modeled with logged difference of the modified z-scores between the model and target responses, which is

$$\text{log}\left|\frac{{\left(\left|f_{m}\right|-\delta \right)}_{+}+\delta }{s_{m}}-\frac{{\left(\left|f_{t}\right|-\delta \right)}_{+}+\delta }{s_{t}}\right|,$$

as well as the within-class standard deviations of model and target gene expression changes (*s*_*m*_ and *s*_*t*_). This linear model is fitted by genes with non-zero pseudo variances in the first step. For genes with opposite expression changes between the model and target systems, the pseudo variances are estimated by the linear model using the logged difference of the modified z-scores,

$$\text{log}\left(\left|\frac{{\left(\left|f_{m}\right|-\delta \right)}_{+}+\delta }{s_{m}}\right|-\left|\frac{{\left(\left|f_{t}\right|-\delta \right)}_{+}+\delta }{s_{t}}\right|\right),$$

which corresponds to the logged difference when sign(*f*_*m*_) and sign(*f*_*t*_) are different, as well as the within-class standard deviations. Negative estimations are clipped to zero.

For the gene expression analysis of a new experiment using a similar model system (test experiment), EBT penalizes the statistical significance of gene expression changes by the pseudo variances. For a two-class problem, the t-statistics of the differential expression is obtained by

$$t=\frac{f}{\left(s+\alpha \right)\sqrt{1/n_{1}+1/n_{2}}},$$

where *α* is the pseudo variance of the gene, *f* is the expression fold change between two classes, *s* is the within-class standard deviation of the gene expression, and *n*_*1*_ and *n*_*2*_ are the numbers of samples in two classes. The p-values can be calculated by assuming that the penalized t-statistics also follow Student’s t-distributions.

### Gene expression data sets

EBT was demonstrated over publically available gene expression data sets in the Gene Expression Omnibus (GEO) [14]. In this work, we focus on mouse model experiments to simulate human immune responses, where model responses have been reported to be significantly different from those of target human responses [9]. For the evaluation of EBT, pseudo variances need to be estimated from one pairs of model and target experiments (training data) and applied to another experiment pair (test data). We searched data sets with more than two pairs of model and target gene expression profiles under similar two-class comparison tests. To minimize potential biases by different experiment settings, we chose experiments performed by the same research group. Through the extensive search for such data sets in the whole GEO database, we found the following data sets.

(1) GSE19492 for macrophage responses to lipopolysaccharide stimulations [15]. This data set includes the genomic responses of macrophage to lipopolysaccharide at multiple time points after the simulation as well as corresponding control genomic profiles. Mouse and human macrophage experiments at one time point were used for training data, and experiments at another time point were used for test data.
(2) GSE16387 for the macrophage responses to IL-4 induction [16]. This data set contains the genomic responses to IL-4 induction of four mouse macrophage models with and without the agonist Rosiglitazone (RSG) as well as those of human macrophage responses with and without RSG. Using a pair of mouse and human experiments without RSG as a prior experiment, EBT was applied to the mouse experiment with RSG and the translated results were compared with the corresponding human macrophage experiment with RSG.
(3) GSE33341 for bacterial infection [17]. This data set has the genomic responses to *Escherichia coli* and *Staphylococcus aureus* of the whole blood of two strains of mice at multiple time points post-infection as well as those of human whole blood from infected patients. It also has corresponding mouse and human baseline gene expression profiles as control. EBT was trained in the *Escherichia coli* infection experiments and applied to the translation of the *Staphylococcus aureus* infection experiments, and vice versa.
(4) GSE48200 for influenza virus infection [18]. This data set has the genomic responses of mouse kidney and human lung cell lines to five H5N1 virus mutant strains. The pseudo variances of EBT were estimated in the experiments with one virus strain and applied to the analysis of the model responses to another virus strain.
(5) Glue Grant data set (GSE37069, GSE36809, GSE3824, and GSE7404) for human and mouse responses to severe injury [9]. The data set has the genomic profiles of mouse models for burn, trauma and endotoxemia as well as corresponding controls. It also has the genomic responses of burn and trauma patients as well as those of *in vivo* endotoxemia human models. Considering the endotoxemia mouse and human models as a prior experiment, EBT was applied to the analyses of the genomic responses of mouse burn and trauma models.

We used the processed gene expression indices obtained from the GEO database. Some platforms have multiple probe sets for a single gene to represent its isoforms. Among them, the probe set with the largest variance over the whole data set was chosen to represent the single gene. The human and mouse orthologous genes were mapped by gene symbols, largely following the previous study [9]. More than 10,000 orthologous genes were used for the all data sets.

## Results and Discussion

### Evidence-based translation (EBT)

Evidence-based translation (EBT) improves the conventional translation of genomic responses of a model system based on the evidences of concordance between model and human responses in prior experiments with similar settings. In the conventional translation, the significant genes in a model experiment are directly translated to the corresponding target human system, and the orthologous genes of model and human systems are considered to have similar expression patterns. However, this naïve translation potentially includes many false positive genes of which expression changes in the model system are poorly correlated with those of the target human system [9]. In contrast, EBT prevents such false positive translation by introducing pseudo variances that are calculated according to the discordance between model and human responses in prior experiments. Pseudo variances are applied to the statistical analysis of a new experiment using the same or similar model systems. Genes with high pseudo variances are penalized in the significance analysis of gene expression changes, and they are less likely to be detected as significant genes. As a result, EBT improves the agreement of gene expression changes between model and target responses.

The idea of adding pseudo variances is common. It has been used in the analysis of gene expression and microarray data to stabilize the calculation of differential expression [19,20]. In Bayes estimations, the effect of a prior distribution can be implemented as a pseudo variance in a posterior distribution [21]. In this work, pseudo variances are considered to represent the amount of additional noises in translation. The ratio of a fold change to a standard deviation, which is often used as a statistic for differential expression, can be interpreted as a signal-to-noise ratio, where the standard deviation represents the amount of an experiment noise [22]. This noise includes sample variations, technical artifacts, and experimental errors. In the translational analysis, we can consider an additional noise due to imperfect translation. The experiment and translation noises can be also considered as source and channel noises, respectively, when we observe signals from a source through a channel. If a gene response is translated well (or well-correlated between model and target), it has a small translational noise and consequently a small pseudo variance. Since the translational noise is expected to be independent to the experiment noise, it should be additive as a pseudo variance in the calculation of a signal-to-noise ratio or a statistic for differential expression.

Pseudo variances are obtained from a pair of prior model and corresponding target experiments. EBT proposed in this work focuses on a two-class comparison problem, which is commonly used in biomedical experiment designs. For each of model and target experiments, z-scores of gene expression changes, a fold change between two conditions divided by a within-class standard deviation, can be calculated. EBT uses a modified z-score using a truncated and shifted fold change to prevent potential noises from small fold changes. The pseudo variance of a gene is calculated to minimize the difference between the modified z-scores of the model and target experiments by being added to the variance of the model response. For genes with the opposite directions of expression changes between the model and target systems, pseudo variances are estimated by a linear model. This linear model is fitted by the pseudo variances of genes with the same direction of expression changes.

As an example, pseudo variances were calculated for the genomic responses of mouse macrophages to lipopolysaccharide stimulations at 6 hours post-stimulation in the comparison with those of human macrophages at the same time [15]. In each of mouse and human experiments, gene expression fold changes were calculated between macrophages with and without stimulations. The estimated pseudo variances and the difference of the modified z-scores had a good linear relation (S1 Fig.), which supports the use of a linear model to estimate the pseudo variance for genes with the opposite direction of expression changes. Among 10,003 genes in the experiment, 2,888 (29%) genes had no additional variance, 2,202 (22%) genes had moderate variances between 0 and 0.5^2^, and 4,913 (49%) genes had large pseudo variances more than 0.5^2^.

**Fig 1:**
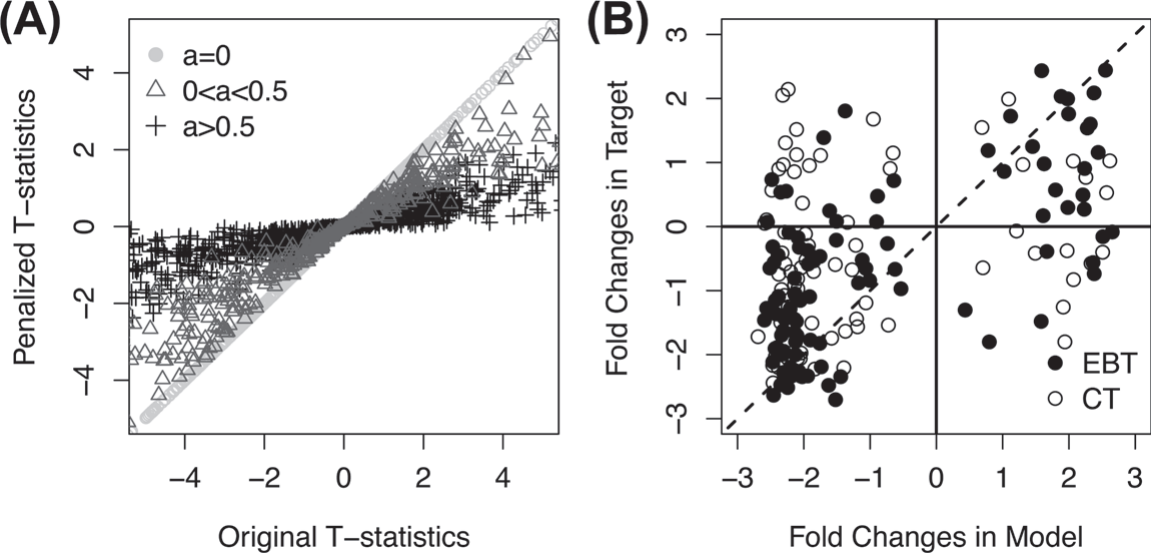
Calculation of pseudo variances. **(A)** The distribution of pseudo variances according to the original and penalized t-statistics in the test experiment. Circles, triangles and crosses represent genes with no additional pseudo variances, variances between 0 to 0.5^2^, and variances larger than 0.5^2^, respectively. **(B)** Fold changes in the test target and model experiment of the top 100 significant genes selected in the EBT (black) and in the conventional translation (white). Commonly significant genes between the EBT and conventional translation are shown in black.

The calculated pseudo variances were applied to the analysis of the genomic responses of the model system at 24 hours post-stimulation. T-statistics penalized in the EBT were calculated between two classes with the additional pseudo variances, and compared with the original t-statistics without pseudo variances (Fig. 1A). In the EBT, the analysis found 1,363 genes with p-values < 0.05, among which 1,036 (76%) genes showed the same direction of changes with those of the target human responses in a similar setting at 24 hours (S2A Fig.). In contrast, among the significant genes found in the conventional translation, only 66% had concordant expression changes. Especially, false positive genes, which were significant in the conventional translation (p-values < 0.05) but not in the EBT (p-values > 0.5), had only a 50% agreement rate (S2B Fig.). Among the top 100 genes in the order of the EBT significance, 81 genes showed the concordant directions of changes while only 69 genes were in the concordance among the same number of top genes in the order of the significance of the conventional translation (Fig. 1B).

Among 1,363 significant genes found in the EBT (p-values < 0.05), 266 genes represented experiment-specific responses, which had the opposite direction of expression changes at 24 hours post-stimulation compared to the responses at 6 hours. These genes have the responses specific to 24 hours that cannot be observed at 6 hours. Among these genes, 80 (30%) genes showed the same experiment-specific responses in the target human system. Similarly, 40 of the top 100 experiment-specific genes found by EBT showed concordant responses with the target system. For the experiment-specific genes found in the conventional translation, only 21% of significant genes (p-values <0.05) and 20 of the top 100 genes showed concordant responses. If a gene has similar responses between human and mouse training data (i.e. true positive), it is also expected to show the similar responses in test data even though its expression is changed to the opposite direction. EBT is based on removing false positives in translation, which is disagreement in human and mouse responses. Therefore, it is expected that EBT can improve the agreements of experiment-specific responses.

### Evaluation of EBT

EBT proposed in this work was further evaluated using a public data set (GSE19492) for the genomic responses of human and mouse macrophages to lipopolysaccharide [15]. The data set contains three pairs of mouse model and target human responses at 2, 6 and 24 hours post-stimulation. By estimating pseudo variances from one pair of experiments and applying them to another experiment pair, we evaluated EBT for all of the six pairwise cases.

**Fig 2:**
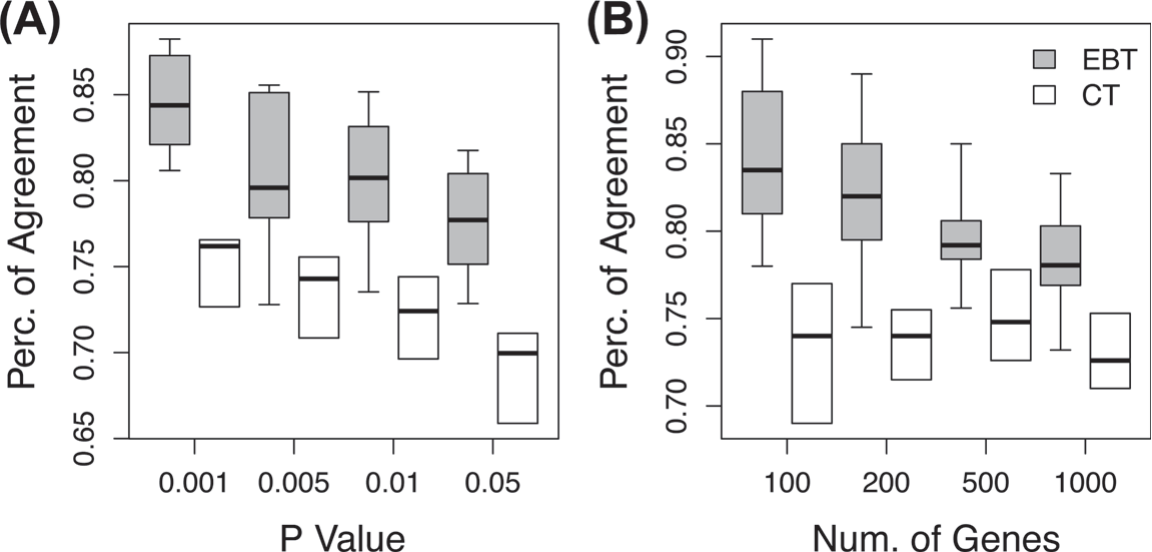
The agreement of the overall responses in the EBT and conventional translation. The agreement percentages **(A)** of significant genes selected by p-values and **(B)** of top genes in the order of significances in the EBT (gray) and conventional translation (CT; white).

First, EBT was evaluated for the translation of overall responses. In the analysis of a model experiment, significant genes were selected by EBT with pseudo variances trained from a prior experiment. The directions of expression changes of these mouse genes were compared with those of the orthologous genes of the corresponding target human system, and the percentage of direction agreements was measured. The agreement percentages of genes selected by EBT were compared with those of genes selected in conventional translation, which considers all orthologous human genes will have similar expression patterns with those of significant model genes. Fig. 2A shows the agreement percentages of genes selected by various p-value criteria for the pairwise six cases of the whole data set. In addition, Fig. 2B shows the agreement percentages of the top significant genes chosen in the order of significance in the EBT and conventional translation. The concordance rates of EBT are significantly higher than those of conventional tests. For the genes with p-value less than 0.05, the agreement percentages of EBT are on average 0.78 (±0.03 standard deviation) while those of conventional translation are 0.69 (±0.02). The p-value for the agreement difference between the two translation methods by a paired t-test is less than 0.001. For a wide range of selection criteria, EBT shows higher concordance between the model and target responses than conventional translation, which implies the improved translation of model system experiments to target system responses.

**Fig 3:**
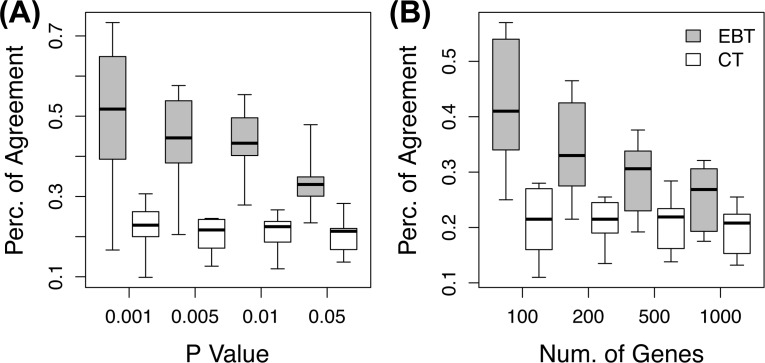
The agreement of the specific responses in the EBT and conventional translation. The agreement percentages **(A)** of significant genes selected by p-values and **(B)** of top genes in the order of significances in the EBT (gray) and conventional translation (CT; white).

Second, EBT was evaluated for the translation of experiment-specific responses. In a new experiment, genomic responses that are different from those of a prior experiment are often of interest. Here, activated gene expression changes in a new experiment while suppressed in a prior experiment, and vice versa, are considered to be specific to the new experiment. We investigated the agreement percentages of the specific responses of model systems with those of target systems in the same human and mouse macrophage data set. If a gene with an experiment-specific response in a model system shows the same experiment-specific response in a target system, it is counted as an agreement. The percentages of agreements were measured for genes selected by p-value criteria as well as for the fixed numbers of top genes in the order of test significance by EBT and conventional translation (Fig. 3). EBT shows higher agreement percentages than conventional translation over a wide range of selection criteria. When genes are selected by p-values less than 0.05, the average agreement percentage of EBT is 0.34 (±0.08) while that of conventional translation is 0.21 (±0.08), which is significantly different (p-value < 0.001). The agreement percentages of the specific responses are lower than those of overall responses because genomic responses are considered to be agreed only when the directions of both overall expression changes and experiment-specific changes are the same between model and target experiments.

**Fig 4:**
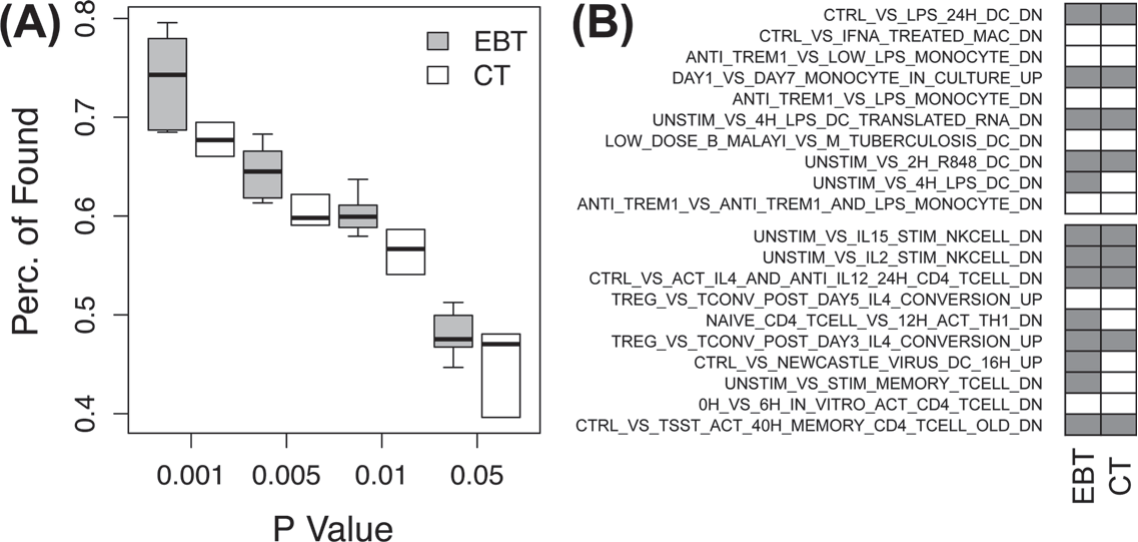
The agreement of biological contexts in the EBT and conventional translation. **A)** The agreement percentages of biological contexts significantly enriched in genes selected in the EBT (gray) and conventional translation (CT; white). **(B)** Top 10 biological contexts of immunological signatures enriched in activated (upper panel) and suppressed (lower panel) genes in the target human system. Contexts with gray color are commonly found by model experiments through the EBT or through the conventional translation (CT).

Finally, we tested translation of biological contexts enriched in the selected genes by EBT (Fig. 4). Biological contexts significantly enriched in each of activated and suppressed genes were obtained by Fisher’s exact tests [23]. The immunologic signature gene sets of Molecular Signature Database (MSigDB) [24] were used for biological contexts because the major responses to lipopolysaccharide of macrophages in the data set were expected to be related to immunologic signatures. Fig. 4A shows that the percentage of the biological contexts commonly found in the model and target experiments (p-values < 0.05) among the all contexts significantly enriched in the model experiments. The biological contexts selected by EBT have more concordance than those in conventional translation. For example, in the EBT with pseudo variances estimated from the model and target experiments at 6 hours post-stimulation, five of ten activated contexts and eight of ten suppressed contexts in the model responses at 24 hours were commonly found in the corresponding target human experiment (Fig. 4B). In contrast, only four and five contexts were common in the conventional translation.

### Evaluation of EBT over multiple data sets

EBT was further evaluated over multiple public data sets. In the Gene Expression Omnibus (GEO) database, we intensively searched for data sets with pairs of model and target system experiments. We specifically focused on data sets that study human immune responses using mouse models because mouse models have been reported to be poor to mimic human responses in immune-related diseases [9]. Finally, five mouse model and target human experiment data sets were used for the further evaluations: GSE19492 for the macrophage responses to lipopolysaccharide stimulations [15], GSE16387 for the macrophage responses to IL-4 induction [16], GSE33341 for bacterial infection [17], GSE48200 for influenza virus infection [18], and the Glue Grant data set for severe injury [9]. Each data set has multiple pairs of murine model experiments and target experiments using human samples. Pseudo variances are estimated from one pair of model and target experiments, and applied to another experiment pair. EBT was evaluated for the multiple combinations of model-target experiment pairs in a data set, in total for 50 combinations. The evaluations were performed for overall, experiment-specific and context-level responses as described in the previous section.

EBT shows improved concordance between the genomic responses of mouse models and the corresponding human systems (Table 1). The agreement percentages of overall responses show up to a 56% improvement (6 ~ 56%, 23% on average) by EBT when significant genes are selected by p-values < 0.05. The concordance rates of EBT are significantly improved compared with those of the conventional translation for all of the five data sets (paired t-test p-values < 0.05). For experiment-specific responses, the agreement percentages of EBT have up to a 143% improvement (33 ~ 143%, 72% on average), which are significantly higher than those of conventional translation in the all data sets. The percentages of biological contexts commonly enriched in model and target responses are also improved by up to 100% (0 ~ 100%, 33% on average). These improvements are significant in four of the five data sets. The proposed method also shows similar improved agreements for the top 500 genes selected in the order of test significances (Table 2). Even in the worst cases, EBT shows comparable concordance with naïve conventional translation. The averages of pseudo variances in each data set are summarized in S1 Table.

**Table 1:** Agreement of genes and biological contexts selected by a significance criterion.

	**Overall**			**Specific**			**Contexts**		
	**EBT**	**CT**	**PV**	**EBT**	**CT**	**PV**	**EBT**	**CT**	**PV**
GSE19492	0.78±0.03	0.69±0.02	<0.001	0.34±0.08	0.21±0.05	<0.001	0.48±0.02	0.45±0.04	0.057
GSE16387	0.86±0.04	0.55±0.04	<0.001	0.18±0.04	0.10±0.03	0.016	0.40±0.03	0.20±0.03	<0.001
GSE33341	0.75±0.07	0.62±0.05	<0.001	0.08±0.03	0.06±0.02	0.027	0.51±0.03	0.45±0.04	<0.001
GSE48200	0.73±0.13	0.55±0.05	<0.001	0.14±0.08	0.10±0.05	0.018	0.29±0.06	0.20±0.04	<0.001
GlueGrant	0.67±0.06	0.63±0.07	0.023	0.34±0.06	0.14±0.04	<0.001	0.34±0.08	0.34±0.07	0.708

The agreement of significant genes and biological contexts (p-value < 0.05) in the model response analyses with those of target response analyses. Shown are average percentages of agreements ± standard deviations and p-values from paired t-tests between evidence-based translation (EBT) and conventional translation (CT).

**Table 2:** Agreement of top genes and biological contexts in the significance order.

	**Overall**			**Specific**			**Contexts**		
	**EBT**	**CT**	**PV**	**EBT**	**CT**	**PV**	**EBT**	**CT**	**PV**
GSE19492	0.80±0.03	0.75±0.02	0.005	0.29±0.07	0.21±0.05	<0.001	0.68±0.06	0.65±0.08	0.031
GSE16387	0.87±0.03	0.55±0.03	<0.001	0.15±0.02	0.10±0.02	0.013	0.44±0.04	0.25±0.11	0.025
GSE33341	0.85±0.06	0.74±0.04	<0.001	0.09±0.03	0.05±0.02	<0.001	0.72±0.07	0.67±0.06	0.003
GSE48200	0.76±0.13	0.56±0.04	<0.001	0.14±0.06	0.11±0.06	<0.001	0.34±0.07	0.23±0.05	<0.001
GlueGrant	0.68±0.07	0.65±0.08	0.098	0.31±0.06	0.15±0.04	<0.001	0.44±0.16	0.44±0.17	0.789

The agreement of top 500 significant genes and biological contexts in the model response analyses with those of target response analyses. Shown are average percentages of agreements ± standard deviations and p-values from paired t-tests between evidence-based translation (EBT) and conventional translation (CT).

### Characteristics of EBT

We further investigated the characteristics of EBT. EBT is trained from a pair of model and target experiments, and applied for another model-target experiment pair. In this section, we used the same five data sets in the previous section, which have 50 different combinations of model-target experiment pairs in total.

First, genes benefiting from EBT were investigated. Among the significant genes in the conventional translation (regular p-value < 0.05), we selected genes that were also significant by EBT (p-value with a pseudo variance < 0.05) for each combination of model-target experiment pairs. These genes were considered to benefit from EBT. Over the 50 test combinations of the five data sets, 1,382 genes were found to benefit in multiple tests and data sets (≥10 tests and ≥3 data sets). Among these genes, 8 genes particularly benefited in ≥20 tests as well as in all of the five data sets (S2 Table). These 8 genes include important immune-related genes such as interferon induced transmembrane protein 2/3 (IFITM2/3) and interleukin 1 receptor antagonist (IL1RN) as well as cell signaling genes such as chemokine (C-X-C motif) ligand 10 (CXCL10). Using the canonical pathway and gene ontology gene sets in MSigDB [24], we further investigated the biological functions enriched by the 1,382 benefitting genes. Enrichment of a gene set was tested by Fisher’s exact test [23]. The analyses showed that the benefitting genes were mainly involved in immune functions such as cytokine signaling and interferon pathways as well as in metabolic functions such as biopolymer and protein metabolic processes (S3 Table). The overall result implies that EBT can be more beneficial to murine model experiments related to immune and metabolic responses.

Second, we searched factors affecting the performance of EBT, focusing on the similarity of genomic response patterns between models and targets as well as the similarity between training and test sets. To measure the similarity, we used rank correlation of gene expression changes between two experiments. For the 50 test combinations of the five data sets, we measured the improvement rates of agreement percentages. Then, we examined the contribution of the genomic response similarities to the improvement rates in a linear regression model (S4 Table). The significance of each factor was estimated by ANOVA tests. For overall responses, the similarity of mouse responses between training and test sets most significantly contributed to the improvement (p-value = 7.19x10^-14^). For specific responses, the most significantly contributing factor was the similarity between mouse and human responses in test sets (p-value = 6.64x10^-4^). In the data sets, the overall rank correlation coefficients were 0.0 to 0.3. In this range of low correlations, the mouse-human similarity positively contributed to EBT. The overall result implies the potential efficiency of EBT for experiments in similar settings with prior ones as well as for good models of human conditions.

## Conclusions

In this work, we introduce EBT, a computational method to efficiently translate the genomic immune responses of a murine model into the corresponding target human system. By introducing pseudo variances estimated from prior model-target experiments, EBT efficiently prevents false positive detections in the translation of the results of new experiments. Evaluated over multiple data sets and compared with conventional direct translations, model experiment results in the EBT showed significantly improved concordance with target system responses for overall and experiment-specific responses as well as for associated biological contexts. EBT achieved up to a two-fold improvement in the percentage of agreement, and it showed at least comparable concordance with conventional translation even in the worst case.

While this work is inspired by dissimilarity between human and mouse responses reported by Seok *et al* [9], there have been various debates on the human-mouse similarity. Among them, Takao *et al* [25] claims high similarity based on well-correlated responses of genes that are commonly significant in both human and mouse data. Since their analysis excludes many significant genes (close to 90%) in a human condition, it only supports partial similarity between human and mouse responses, not the overall similarity. Nevertheless, the analysis approach of Takao *et al* [25] can be considered as an alternative strategy for the translation, i.e. for the analysis of a test set, considering only significant genes in both human and model training set. We have applied this approach to the data of Fig. 1 (GSE19492, macrophage response to lipopolysaccharide, 6hr response for training data and 24hr response for test data). 874 genes are commonly significant (p-value < 0.05) in both human and mouse training data. 554 among them are also significant in the mouse test data, among which 401 (72%) genes have the same direction of changes with those of the human test data. As expected, the commonly significant genes have much smaller pseudo variances (median = 0.029) than the rest of the genes (median = 0.543). However, this excluding approach misses many other genes that can be translated well. For the same setting, EBT finds 1,363 significant genes in the mouse test data, among which 1,036 (76%) genes have the same direction of changes. Applied to other cases, on average the exclusion approach finds only 35% of significant genes of EBT while the agreement rates are similar or even worse. This result shows that EBT is more efficient than the exclusion approach of Takao *et al* [25].

To fill the gap between model and human responses, there have been many efforts, mostly focusing on developing better models, such as humanized mice [11] and *in vitro* reconstruction of disease-related tissues [10], which try to mimic both phenotypic and molecular responses of human. While these models might be promising to provide new insights for target system responses, they are not the same with the target systems and the experiment results still need to be translated. Moreover, developing and validating such a new model requires a lot of biomedical resources and a long time. In contrast, the computational translation proposed in this work provides a fast and convenient way to interpret model experiment results. Given previous model-target experiment pairs, the responses of a model system can be translated through EBT. Applied to newly developed models resembling the underlying molecular responses of humans, the proposed method will be able to predict target human responses with an improved accuracy and efficiency.

A pure computational model can be considered as an alternative way to the current model experiments, which is one of the essential long-term goals of computational biology [12,26]. Computational models preform *in silico* simulations to predict the phenotypic changes of target systems. The construction of such a computational model requires previous knowledge for molecular interactions, protein activations, and signaling pathways, which cannot be solely obtained from experiments using human samples. Our previous knowledge needs to be translated from model experiments to the target system of computational models. This translation can also be benefited by EBT in this work.

EBT was evaluated over various types of biological experiments. In GSE33341 and Glue Grant data sets, EBT was tested between *in vivo* mouse models and actual human patients from clinical sites. It was also tested for *in vitro* cell experiments (GSE19492 and GSE16487) as well as between the heterogeneous cell lines (GSE48200). Especially, EBT was tested for the effect of antagonists in GSE16487 data set, which can be considered as a simulation of drug effects. Theses demonstrations imply that EBT is useful for various experiment settings.

In the case that a research group does not have mouse and human experiment pairs that can be used to train EBT, it will be a good strategy to utilize public data sets in a similar setting. The characteristics of EBT provide a general guideline to select training data sets. High similarity of mouse experiments between training and test sets is an important criterion. For example, in the Glue Grant data sets mouse-mouse correlation was 0.06 on average while >0.45 in GSE19492, GSE16387 and GSE48200, which explains a relatively low improvement of the translation of the overall responses in the Glue Grant data set. However, this general guideline does not provide the optimal choice. To maximize the utilization of the public data sets for EBT, we need to further investigate important problems such as correction of platform heterogeneity and incorporation of multiple training data sets.

While in this work EBT was demonstrated on murine models for human immune responses, we believe, it is equally applicable for the translation of other model systems for cancer, metabolic disorder and mental diseases. Additionally, in the computational drug repositioning using the compendium of drug response genomic profiles [27], EBT can be applied to the translation of the drug responses of cell lines to target host responses. It will be also possible for EBT to improve the discovery of suitable model systems for a given biological problem [28]. In summary, EBT proposed in this work is highly expected to be beneficial for a wide-range of biomedical research using model systems.

## Supporting Information

S1 Fig A scatter plot of the estimated pseudo variances and the difference of modified z-scores in the training experiments. (PDF)

S2 Fig Expression patterns of significant genes in the EBT and conventional translation. (PDF)

S1 Table Calculated pseudo variances in the tested data sets. (PDF)

S2 Table Genes benefited by EBT. (PDF)

S3 Table Top 5 biological functions enriched in the benefited genes by EBT. (PDF)

S4 Table Factors contributing to EBT. (PDF)
