# Supplementary material for: Evidence-Based Translation for the Genomic Responses of Murine Models for the Study of Human Immunity
Source: PLoS One. 2015 Feb 13;10(2):e0118017. doi: 10.1371/journal.pone.0118017 (PMC4332676; doi:10.1371/journal.pone.0118017)
Supplement: S1 Fig — (PDF) [file pone.0118017.s001.pdf]

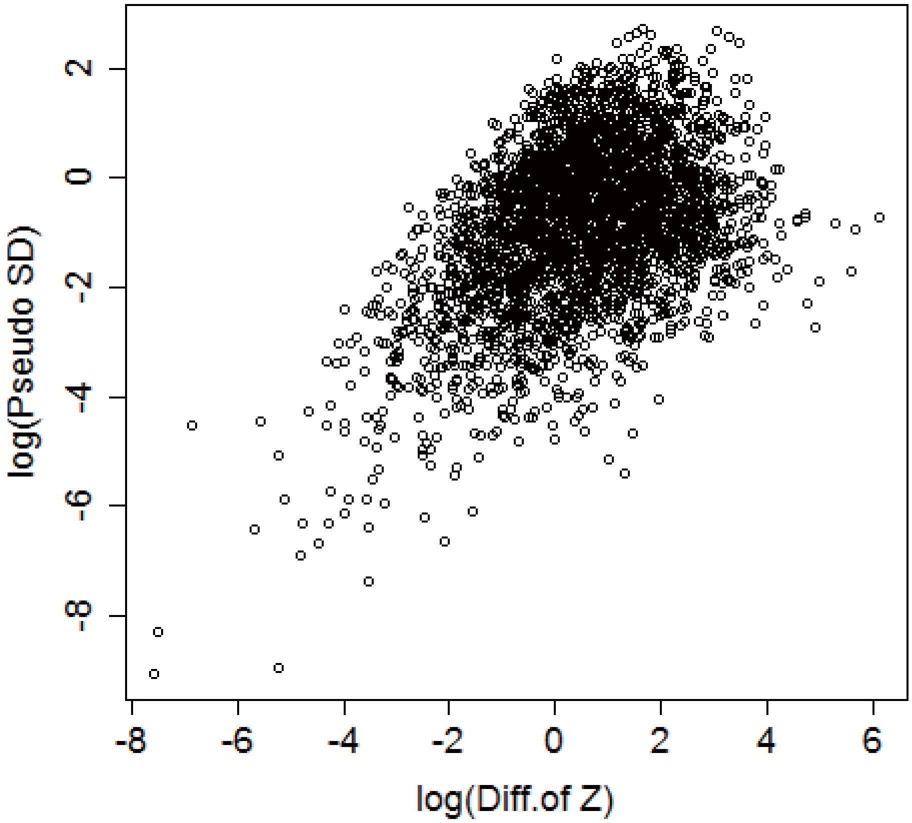

Figure S1. A scatter plot of the estimated pseudo variances and the difference of modified z-scores in the training experiments.
