# Supplementary material for: Evidence-Based Translation for the Genomic Responses of Murine Models for the Study of Human Immunity
Source: PLoS One. 2015 Feb 13;10(2):e0118017. doi: 10.1371/journal.pone.0118017 (PMC4332676; doi:10.1371/journal.pone.0118017)
Supplement: S2 Fig — (PDF) [file pone.0118017.s002.pdf]

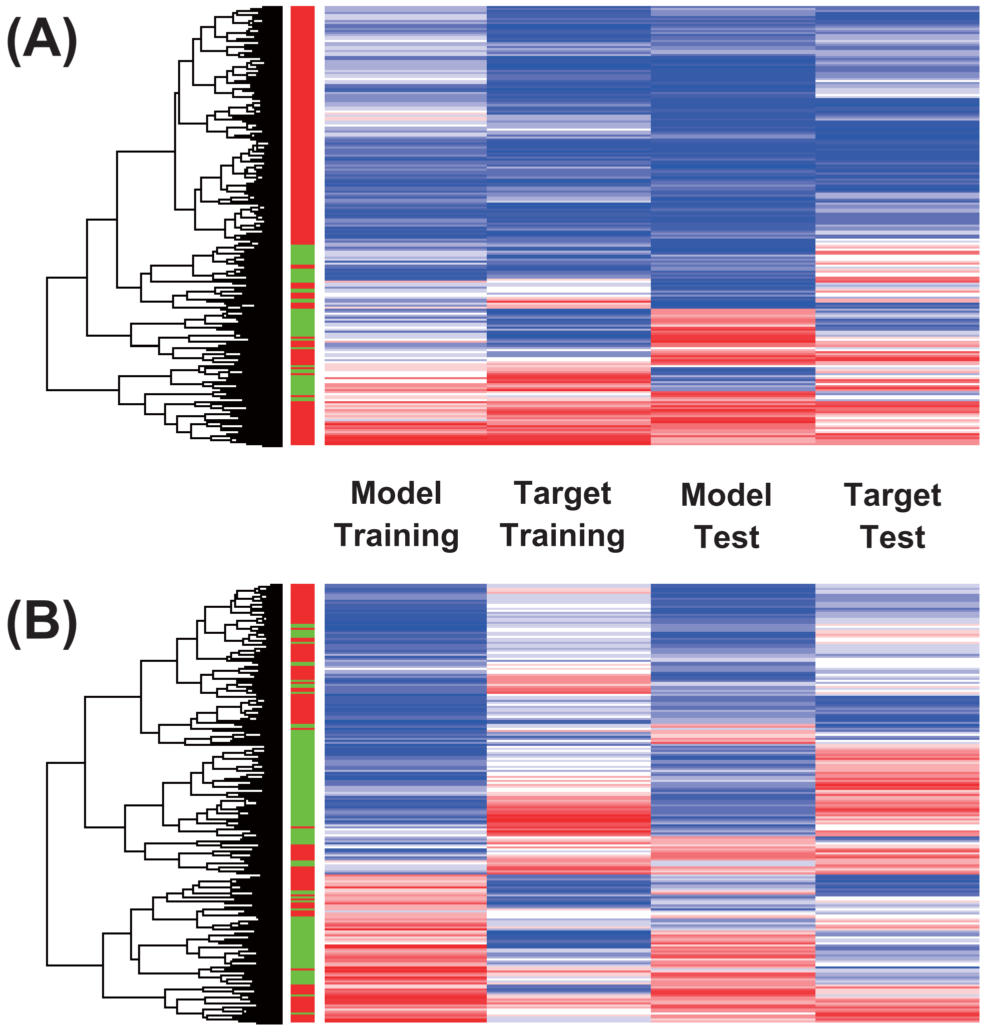

Figure S2. Expression patterns of significant genes in the EBT and conventional translation. Expression patterns of **(A)** significant genes in the EBT (p-values<0.05) and **(B)** genes significant in the conventional translation (p-values<0.05) but not in the EBT (p-values>0.5) of the model and target systems in the prior training experiments as well as in the test experiments. In the side bars, genes with concordant expression changes between model and target responses in the test experiments are marked in red, and genes with discordant changes are marked in green.
