# Supplementary material for: Evidence-Based Translation for the Genomic Responses of Murine Models for the Study of Human Immunity
Source: PLoS One. 2015 Feb 13;10(2):e0118017. doi: 10.1371/journal.pone.0118017 (PMC4332676; doi:10.1371/journal.pone.0118017)
Supplement: S1 Table — (PDF) [file pone.0118017.s003.pdf]

Table S1. Calculated pseudo variances in the tested data sets.

The averages of pseudo variances of training sets in each data set of Table 1 are shown. If a gene is not measured in a data set, its pseudo variance is noted as NA.

Gene	GSE19492	GSE33341	GSE16387	GSE48200	GlueGrant
A1bg	1.81	NA	NA	NA	NA
A1cf	NA	1.55	0	2.41	1.11
A2bp1	0.39	NA	NA	NA	1.71
A2ld1	NA	NA	NA	NA	0
A2m	0	0.39	0	2.16	NA
A4galt	NA	2.83	0.94	1.08	0
A4gnt	NA	0.15	0	1.71	3.05
Aaas	0.26	0	2.74	0.22	0
Aacs	0.48	1.68	1.86	0.03	0
Aadac	4.16	4.21	1.9	4.04	3.82
Aadacl2	NA	NA	NA	3.9	NA
Aadat	0.22	NA	3.99	5.71	1.39
Aaed1	NA	NA	0.96	1.47	NA
Aagab	NA	1.46	1.05	2.65	2.75
Aak1	NA	0.19	0.98	1.81	0.49
Aamdc	NA	1.66	1.23	NA	NA
Aamp	0.02	1.65	0.46	0.8	0
Aanat	1.38	1.96	0	1.68	0
Aar2	NA	1.3	1.18	6.3	NA
Aard	NA	NA	NA	0.95	NA
Aars	0.56	2.15	0.37	4.82	1.93
Aars2	NA	NA	2.33	0	0
Aarsd1	NA	1.38	0.3	NA	2.5
Aasdh	NA	NA	1.33	0.64	0
Aasdhppt	0.28	0.77	1.2	NA	0
Aass	0.34	1.46	2.88	1.04	0.57
Aatf	0.48	1.9	0.38	1.68	0
Aatk	0.57	2.47	3.99	1.1	0
Abat	0.45	0.24	1.58	0	0.17
Abca1	0	0	1.96	0	0
Abca12	0.89	0.11	2.22	1.76	0
Abca13	1.5	NA	0.09	0.28	NA
Abca2	0.58	2.9	2.29	0.02	0
Abca3	0.4	1.29	1.42	0.82	1.88
Abca4	0	2.13	1.67	20.86	3.84
Abca5	0.11	0.13	1.14	0.27	0.87

Abca6	0.65	2.34	0.27	0.61	3.1
Abca7	0.75	1.59	0.5	0.04	0
Abca8	2.47	NA	NA	NA	NA
Abca9	0.52	NA	0.06	0.26	2.32
Abcb10	0.37	NA	3.61	0.68	0
Abcb11	1.63	1.64	0	1.45	1.54
Abcb4	0.03	0	2.89	2.74	1.84
Abcb5	NA	NA	NA	0.49	NA
Abcb6	0.26	2.07	2.71	0	1.2
Abcb7	0.61	0	0.3	2.01	0
Abcb8	NA	1.81	0.43	1	0
Abcb9	0.94	1	0.61	0.31	0
Abcc1	0.46	1.59	1.21	0	0.39
Abcc10	0	0	0.33	0	0
Abcc12	0	NA	0.37	1.44	NA
Abcc2	1.16	3.33	1.74	0	2.91
Abcc3	0.71	1.49	3.32	1.27	2.19
Abcc4	0.18	1.49	1.91	0	0
Abcc5	0	0	1.25	0.22	0
Abcc6	0.96	1.12	1.24	1.03	1.5
Abcc8	0.38	1.18	0.87	1.24	8.25
Abcc9	0.39	0.64	0.66	4.71	1.75
Abcd1	1.07	2.15	2.74	0	3.08
Abcd2	2.03	1.47	2.44	0.14	0.39
Abcd3	0	0.35	0.29	0.55	0
Abcd4	0	0	1.73	0.07	0
Abce1	0.49	0.96	0.63	0.63	0
Abcf1	0.82	0.5	0.37	0	0.82
Abcf2	0	0	0.91	1.79	1.66
Abcf3	0.47	0	0.26	1.94	0
Abcg1	0.59	1.68	2.28	0.89	2.84
Abcg2	1.79	1.77	1.58	0.1	2.13
Abcg4	0.67	0.53	0.78	2.04	2.94
Abcg5	0	2.46	2.67	1.7	1.28
Abcg8	1.02	NA	6.31	0.65	1.48
Abhd1	0.88	NA	2.35	0.36	0
Abhd10	NA	0.92	0.82	1.02	0
Abhd11	NA	2.9	0.69	2.22	0
Abhd12	NA	NA	0.68	0.93	0
Abhd12b	NA	NA	NA	3.15	NA
Abhd13	NA	NA	1.73	0.12	1.92

Abhd14a	NA	0	1.92	0.47	1.94
Abhd14b	NA	NA	2.15	1.85	0
Abhd15	NA	NA	1.19	1.49	0
Abhd16a	NA	NA	1.4	2.08	NA
Abhd16b	NA	NA	0.89	2.07	NA
Abhd17a	NA	0.24	2.29	0.01	NA
Abhd17b	NA	0.34	0.6	1.46	NA
Abhd17c	NA	NA	1.67	0.89	NA
Abhd2	0.37	0.57	1.93	0	0
Abhd3	1.01	1.07	0.19	1.79	1.13
Abhd4	0	1.96	2.57	0.14	1.25
Abhd5	0.3	0.58	0.61	2.52	1.48
Abhd6	1.1	0	1.85	2.12	0.63
Abhd8	0	3.33	2.04	1.01	0
Abhd9	0.06	NA	NA	NA	NA
Abi1	0	0.9	1.42	1.24	0
Abi2	0	0.21	0.63	1.66	0
Abi3	0.44	NA	1.32	3.24	0.12
Abi3bp	NA	0.67	0.45	1.89	0
Abl1	0.44	0	1.54	6.58	1.59
Abl2	0	1.51	0.61	1.27	1.28
Ablim1	0	0	1.43	0.68	2.11
Ablim2	0	NA	1.16	1.29	0
Ablim3	NA	0	0.85	4.01	0
Abo	2.56	1.72	0.65	8.48	0
Abp1	1.25	2.62	0	0.99	1.82
Abr	0	2.45	4.08	0	0
Abra	NA	NA	1.25	1.06	NA
Abracl	NA	NA	1.84	0.64	NA
Abt1	2.66	1.93	0.06	1.24	0
Abtb1	0.16	NA	2.95	0.14	1.09
Abtb2	NA	3.73	3.24	1.76	0.26
Acaa1	0.64	NA	NA	NA	NA
Acaa2	0.13	1.64	0.68	1.75	0.74
Acaca	NA	0.36	0.26	0	0
Acacb	1.53	0	0.96	0	0
Acad10	NA	1.32	1.2	0.53	0
Acad11	NA	NA	1.33	NA	NA
Acad8	0.34	1.42	0.89	0.68	0
Acad9	0.15	NA	0.54	0.89	0
Acadl	0.78	1.25	0.95	2.97	0

Acadm	0	1.09	1.65	1.59	0
Acads	0.04	1.09	0.56	0	1.2
Acadsb	1.88	2.82	1.18	2.01	1.8
Acadvl	2.12	0.57	0.21	NA	2.52
Acan	NA	0.43	0.58	2.28	0
Acap1	NA	2.74	0.49	NA	0
Acap2	NA	0	1.07	0	0.83
Acap3	NA	NA	0.63	2.6	0
Acat1	1.97	2.62	1.08	1.26	0
Acat2	0.09	0.32	1.72	NA	0.64
Acbd3	2.96	0.73	1.1	0.01	3.16
Acbd4	1.28	0.61	2.39	1.11	1.02
Acbd5	0.86	NA	0.95	0.39	0
Acbd6	0	NA	1.12	2.13	1.55
Acbd7	NA	NA	NA	3.2	NA
Accn1	0.7	NA	NA	NA	0
Accn2	2.73	NA	NA	NA	4.26
Accn3	0.34	NA	NA	NA	2.7
Accn4	2.68	NA	NA	NA	0
Accn5	2.13	NA	NA	NA	NA
Accs	NA	NA	2.29	1.62	0.45
Accsl	NA	NA	NA	0.42	NA
Acd	NA	0	3.5	17.62	0
Ace	0	2.67	0.91	2.91	1.6
Ace2	1.25	0.31	0.85	13.72	1.8
Acer1	NA	NA	0	0	NA
Acer2	NA	NA	NA	0	NA
Acer3	NA	NA	1.87	0.63	2.66
Ache	0.34	2.41	0.33	2.04	1.55
Acin1	0.24	0.4	3.51	2.72	0.16
Acly	0.65	0.91	0.19	0.84	0
Acmsd	2.93	NA	NA	3.61	NA
Acn9	0	0.88	1.75	1.44	1.04
Aco1	0.03	0	1.01	0	2.21
Aco2	0	1.73	0.49	0	0
Acot1	NA	0	0.1	2.97	NA
Acot11	NA	0.76	1.2	NA	2.23
Acot12	NA	NA	0.54	0	0.56
Acot13	NA	4.49	2.05	1.3	0
Acot2	NA	NA	NA	3.38	NA
Acot4	NA	NA	1.84	0.69	1.3

Acot6	NA	NA	3.08	0	0
Acot7	NA	1.61	1.2	1.46	0
Acot8	NA	0	1.1	0	0.93
Acot9	NA	0	3	1.42	0.31
Acox1	0.37	1.42	0.86	0	1.18
Acox2	6.49	2.9	1.85	0.77	1.59
Acox3	2.45	0.4	1.05	0.53	0.6
Acoxl	1.66	2.54	1.97	0.94	0
Acp1	0	1.29	0.09	0.98	0
Acp2	0.12	0.89	1.88	NA	3.38
Acp5	0	0.03	2.45	0	4.88
Acp6	0.42	1.15	0.89	1.42	0
Acpl2	NA	NA	1.6	1.84	1.85
Acpp	0	0.22	0.44	1.46	0.64
Acpt	NA	NA	NA	1.08	NA
Acr	2.24	2.27	0.23	0.77	NA
Acrbp	0	NA	1	0	2.37
Acrv1	0.35	1.24	0.79	3.05	0.04
Acsbg1	NA	1.51	2.15	3.31	5.69
Acsbg2	NA	1.88	5.49	0.79	0
Acsf2	NA	0	0	0.15	0
Acsf3	NA	NA	0.16	0	0.57
Acsl1	0.07	3	1.39	1.29	2.59
Acsl3	0.19	1.43	0.79	NA	0
Acsl4	0.2	0.24	2.62	0.22	0
Acsl5	1.38	2.49	1.12	0.32	0
Acsl6	1.72	0	0.03	0.85	0.1
Acsm1	NA	1.97	1.81	2.86	0.88
Acsm3	NA	0.32	0.81	NA	0.42
Acsm5	NA	1.1	0.3	0.5	0
Acss1	NA	NA	0.16	0.2	1.32
Acss2	NA	NA	1.33	1.84	0
Acss3	NA	NA	NA	1.05	NA
Acta1	NA	1.35	2.79	0.15	3.39
Acta2	0	0	1.85	1.92	0.01
Actb	0.25	1.83	1.32	1.29	0.65
Actbl2	NA	NA	NA	3.8	NA
Actc1	0	1.37	2.72	0.04	NA
Actg1	0.1	2.25	2.56	1.4	0.68
Actg2	4.02	1.69	2.18	2.92	0.93
Actl10	NA	NA	0.83	0.92	NA

Actl6a	0	1.78	0.94	0.54	0
Actl6b	NA	1.43	0.65	0.49	0
Actl7a	2.58	2.52	1.68	3	0
Actl7b	0	3.85	0.5	0.74	0
Actl9	NA	NA	0	1.94	0
Actn1	0.57	0.48	2.18	1.13	0.16
Actn2	7.24	0.56	0.9	1.57	0
Actn3	1.19	0.94	0.73	2.3	0.99
Actn4	0.77	0	1.05	0	1.15
Actr10	0.3	2.11	1.81	1.15	0.98
Actr1a	0.81	0.53	0.69	0	0
Actr1b	0.15	0	1.7	3.88	0
Actr2	0	0.83	1.16	1.52	0.3
Actr3	0.24	0.31	2.84	1.63	0
Actr3b	NA	0	1.87	5.99	0
Actr5	0	0	0.64	2.09	1.49
Actr6	0.97	1.89	1.71	0.58	0
Actr8	0	0	0	0	0
Actrt1	1.72	NA	NA	0	NA
Actrt2	NA	NA	1.06	2.82	1.17
Actrt3	NA	NA	0.67	0	NA
Acvr1	0	1.67	1.4	1.11	0
Acvr1b	0.34	0.24	1.07	0	0
Acvr1c	4.19	NA	0.68	3.32	NA
Acvr2a	NA	3.66	0	2.31	0
Acvr2b	0	1.37	0.3	0.16	0
Acvrl1	0.94	1.26	1.46	2.54	0.03
Acy1	0.08	NA	1.78	0.62	0.84
Acy3	0.05	NA	3.14	5.43	0.72
Acyp1	4.95	0	0	9.31	2.11
Acyp2	5.44	0.73	2.6	3.34	0
Ada	0.52	1.18	2.1	0.23	0
Adad1	NA	NA	0.4	0.36	3.84
Adad2	NA	NA	3.29	1.55	1.74
Adal	NA	NA	1.1	0.67	0.95
Adam10	0.27	1.45	1.56	0.07	0
Adam11	0.11	1.58	0.67	1.44	1.44
Adam12	0.93	0.13	1.05	2.56	0
Adam15	1.27	1.17	0.9	1.79	1.5
Adam17	0	0	2.51	0.83	0.04
Adam18	1.47	0	0	0.74	0

Adam19	0	2.05	0.55	0	NA
Adam1a	NA	NA	0.61	NA	NA
Adam2	1.02	0.98	3.07	0.53	4.45
Adam20	NA	NA	NA	0	NA
Adam21	1.84	NA	1.27	1.36	NA
Adam22	0.47	0.56	1.21	0.91	0
Adam23	1.39	1.36	0.4	1.02	3.16
Adam28	0.01	0.87	0.76	1.01	0.33
Adam29	NA	NA	NA	2.32	NA
Adam30	0.77	NA	NA	0.07	NA
Adam32	1.09	NA	1.31	0	NA
Adam33	1.48	NA	0	0.47	0.08
Adam5	0	2.17	0.85	0.39	NA
Adam6	0	NA	NA	NA	NA
Adam7	2.61	2.3	1.54	0.47	0
Adam8	1.68	2.07	0.47	0.79	0
Adam9	3.24	0.45	2.61	0	1.7
Adamdec1	0.54	0.75	0.11	0.74	2.01
Adamts1	0.09	1.21	1.53	1.88	1.35
Adamts10	0.57	NA	1.23	1.07	0.94
Adamts12	1.01	2.25	1.46	1.83	1.98
Adamts13	0	NA	NA	0.75	NA
Adamts14	NA	NA	NA	0	NA
Adamts15	NA	NA	2.06	0.2	NA
Adamts16	0.47	NA	0.47	0.52	0
Adamts17	NA	NA	NA	0.46	NA
Adamts18	0.61	NA	0	3.06	0
Adamts19	0.9	NA	1.58	0.37	NA
Adamts2	0	0.34	1.01	0	1.87
Adamts20	0.91	0.46	0.87	0	1.95
Adamts3	NA	0.01	2.3	0	0
Adamts4	0	NA	1.21	1.13	NA
Adamts5	0.85	1.86	0.65	3.44	1.18
Adamts6	2.42	3.65	0.9	0.52	1.36
Adamts7	NA	3.86	0.71	0.45	4.54
Adamts8	1.16	0.33	1.38	0.12	NA
Adamts9	0.42	1.41	0.39	0	2.87
Adamtsl1	0.76	NA	0.74	0.63	0.92
Adamtsl2	NA	0	0.41	1.26	1.82
Adamtsl3	NA	0.08	0.53	0	2.54
Adamtsl4	NA	0.03	2.74	0.99	1.36

Adamtsl5	NA	NA	1.7	1.35	0
Adap1	NA	0.89	1.35	0.95	1.62
Adap2	NA	0	2	1.02	1.24
Adar	2.4	1.41	1.35	0	0
Adarb1	0.85	0	1.2	0	0.68
Adarb2	1.04	1.33	0.16	1.54	0
Adat1	1.35	0	1.36	1.66	1.68
Adat2	NA	NA	1.18	1.35	0.13
Adat3	NA	NA	0.55	1.73	0.54
Adc	NA	NA	0.53	0.58	0
Adck1	0	NA	0.34	1.02	3.48
Adck2	0	0	0.58	0.97	1.57
Adck3	NA	0	0.54	0.69	NA
Adck4	1.22	1.24	0.82	0	0.63
Adck5	0.74	NA	1.99	0	1.26
Adcy1	0.75	0.72	0.26	1.06	2.27
Adcy10	NA	1.04	0.74	0	0
Adcy2	1.5	1.08	1.86	1.54	6.56
Adcy3	0.37	1.56	1.3	0	0.12
Adcy4	1.77	NA	1.32	1.59	3.35
Adcy5	0.47	NA	0.63	1.9	2.3
Adcy6	0.44	4.52	1.18	1.62	0
Adcy7	0	0.23	0.79	0	0
Adcy8	4.03	2.03	0	3.33	0.26
Adcy9	0.25	0	0.53	1.33	0.51
Adcyap1	1.46	0.85	0.82	0.44	1.23
Adcyap1r1	0.66	1.61	0.74	0.29	1.72
Add1	1.25	0	0.93	0.61	0.49
Add2	0	0.6	0.04	0.73	0
Add3	0.64	0.22	1.66	0.84	0
Adfp	0.2	NA	NA	NA	NA
Adgb	NA	1.4	0.68	2.43	NA
Adh4	0.78	NA	0.05	0	3.47
Adh5	0	0.35	1.39	0.82	0
Adh7	NA	1.41	1.07	2.5	2.43
Adhfe1	0.02	NA	0.62	0	1.6
Adi1	NA	0.18	2.81	0.38	0
Adig	NA	NA	0.19	1.98	NA
Adipoq	NA	1.22	0	2.53	0
Adipor1	0.14	0.34	5.05	0.24	1.37
Adipor2	0.06	0.38	1.09	4.1	3.95

Adk	0	0	1.01	1.18	0
Adm	0	0	2.86	1.13	0
Adm2	1.25	1.93	2.45	1.08	0
Admr	1.23	NA	NA	NA	NA
Adnp	0	NA	NA	NA	0
Adnp2	NA	0.6	0.6	1.23	1.33
Ado	NA	0.31	1.06	0.44	0
Adora1	1.3	0.73	1.7	1.25	0
Adora2a	0	NA	NA	NA	NA
Adora2b	1.03	0.09	2.06	2.44	NA
Adora3	0.58	1.86	0.2	0.81	1.71
Adpgk	1.32	2.32	0.31	0.86	5.26
Adprh	0.91	0.22	1.98	1.3	3.11
Adprhl1	0	NA	1.96	0.94	0
Adprhl2	0	NA	3.95	1.75	1.22
Adprm	NA	1.06	1.38	3.09	NA
Adra1a	0.79	0.64	0.82	1.29	1.44
Adra1b	0.52	4.56	1.6	1.39	0.1
Adra1d	0	NA	NA	0.68	NA
Adra2a	0.41	0	1.03	0.04	1.84
Adra2b	1.36	2.07	1.14	0.49	0
Adra2c	0.24	1.91	1	0	0
Adrb1	0.95	0.93	0.99	0.62	1.55
Adrb2	0.11	0.76	2.04	1.29	0
Adrb3	2.06	0.3	1.02	0.85	0
Adrbk1	0.6	0.45	2.36	1.68	0.02
Adrbk2	0	0.81	1.97	0	0.04
Adrm1	0.53	2.11	0.39	2.87	0
Adsl	0.01	1.55	0.44	2.33	0
Adss	NA	0	2.25	0.8	3
Adssl1	0.23	NA	1.03	4.15	0
Adtrp	NA	0	0.69	2.72	NA
Aebp1	1.48	1.23	1.27	0.59	0.08
Aebp2	0.29	NA	1.45	0.75	1.96
Aen	NA	1.18	2.85	1.2	0.58
Aes	3.48	0	2.57	0	4.17
Afap1	NA	0.09	1.3	1.04	1.24
Afap1l1	NA	NA	2.32	1.51	2
Afap1l2	NA	NA	0	0.33	0
Aff1	NA	0.33	0.06	1.14	0
Aff2	NA	0.45	0.81	1.5	1.15

Aff3	NA	2.05	0.81	2.44	0
Aff4	NA	0.89	0.69	0	0.18
Afg3l1	0.83	NA	NA	NA	1.66
Afg3l2	0.67	0	2.69	0.83	0
Afm	1.21	1.11	0	2.26	1.71
Afmid	0.35	NA	0.95	0.82	0.02
Afp	1.34	1.78	0.92	1.36	3.47
Aftph	NA	1.5	1.1	0	0
Aga	2.98	1.33	2.44	0.67	0.25
Agap1	NA	2.2	1.28	1.06	0.26
Agap2	NA	0.57	1.59	2.38	2.85
Agap3	NA	NA	0.47	1.04	1.22
Agbl1	NA	NA	NA	1.35	NA
Agbl2	NA	NA	NA	0.07	NA
Agbl3	NA	1.38	0	0	0.08
Agbl4	NA	NA	0.86	0	NA
Agbl5	NA	0.01	2.21	0.76	1.69
Ager	0.58	0.55	0.73	0.01	0
Agfg1	NA	0	1.35	18.95	0
Agfg2	NA	0.78	2.53	2.11	0.53
Aggf1	NA	0.14	0.95	0.43	2.36
Agk	NA	0	0.81	1.08	1.76
Agl	0.38	0.9	1.31	3.06	0
Agmat	0.87	0	0	0.75	1.56
Agmo	NA	NA	NA	0.81	NA
Ago1	NA	0	1.77	0	NA
Ago2	NA	2.23	0.76	3.3	NA
Ago3	NA	1.2	0.09	0	NA
Ago4	NA	1.05	2.81	0	NA
Agpat1	0.44	0.72	0.07	NA	2.46
Agpat2	0	0	2.44	1.25	0
Agpat3	0.38	2.24	0.88	2.21	1.61
Agpat4	1.5	0.85	1.3	0	2.24
Agpat5	NA	1.91	1.08	0.04	6.98
Agpat6	NA	NA	0.26	1.45	0
Agpat9	NA	NA	2.69	0.34	5.08
Agphd1	NA	NA	NA	NA	2.16
Agps	0.84	1.6	0.88	0.56	0
Agr2	1.38	1.29	0.93	0.88	4.44
Agr3	NA	NA	1.83	0.77	0
Agrn	NA	0.62	0.42	0.87	1.7

Agrp	0.32	1.63	3.14	2.96	0
Agt	0.54	1.19	0.54	0	1.58
Agtpbp1	0	1.14	0.58	0.87	0.15
Agtr1	0.79	NA	NA	NA	NA
Agtr2	2.69	1.14	1.97	1.98	4.36
Agtrap	0.1	NA	1.62	0.27	0.87
Agtrl1	0.68	NA	NA	NA	NA
Agxt	0.81	1.49	0.98	4.1	0
Agxt2	NA	NA	1.2	1.42	1.61
Agxt2l1	4.22	NA	NA	NA	0.26
Agxt2l2	NA	NA	NA	NA	0
Ahctf1	NA	0	0.41	1.49	1.47
Ahcy	0	1.21	0.67	0	0
Ahcyl1	1.28	0.46	4.62	0	2.05
Ahcyl2	NA	0	0.86	0.01	0
Ahdc1	NA	0.79	0.26	0	0
Ahi1	0	0.84	0.64	3.82	0
Ahnak	1.72	0	1.37	18.68	0
Ahnak2	NA	1.66	1.21	0.06	0
Ahr	2.03	0.75	0.6	0	0
Ahrr	1.35	NA	0.72	1.75	1.35
Ahsa1	0.39	0.37	0.78	0.32	0.01
Ahsa2	NA	NA	2.24	1.73	8.28
Ahsg	1.14	2.16	0.06	1.33	2.55
Ahsp	NA	1.69	0.32	0.59	NA
Aicda	0	2.43	0.05	1.88	0
Aida	NA	1.83	1.15	0.8	2.44
Aif1	0.31	0.38	2.36	0.01	0
Aif1l	NA	NA	0.89	0.7	1.17
Aifm1	NA	1.18	1.27	0.22	3.35
Aifm2	NA	NA	1.44	1.89	0.51
Aifm3	NA	NA	0	1.02	1.56
Aig1	0.89	NA	1.32	2.83	0.62
Aim1	1.25	2.43	2.07	0.2	0
Aim1l	1.7	1.28	1.19	3.39	0
Aim2	NA	0	0	2.4	1.21
Aimp1	NA	0.98	4.54	5.18	2.51
Aimp2	NA	1.09	0.96	NA	3.79
Aip	0.27	0	0.33	0.76	0
Aipl1	0.5	1.76	2.71	1.11	0
Aire	0.68	1.46	0.32	1.16	1.27

Ajap1	NA	1.01	0	1.33	5.67
Ajuba	NA	NA	1.24	2.7	NA
Ak1	0	1.09	0.33	0	1.66
Ak2	0.67	1.1	0.6	0.66	NA
Ak3	NA	NA	1.31	1.68	2.26
Ak3l1	NA	NA	NA	NA	3.07
Ak4	NA	1.26	1.37	1.35	NA
Ak5	0	2.7	0.1	1.68	0
Ak7	1.42	NA	0	2.71	14.02
Ak8	NA	NA	1.23	0.47	NA
Ak9	NA	NA	NA	0.44	NA
Akap1	0.18	0.63	0.01	1.88	1.83
Akap10	0.9	1.17	1.06	0.18	0.04
Akap11	0	0	0.69	0	1.52
Akap12	0.08	1.31	1.37	0.16	1.06
Akap13	NA	0.15	0.95	0	0
Akap14	NA	NA	1.44	5.65	1.03
Akap2	1.07	NA	NA	NA	NA
Akap3	0.03	1.04	0.73	2.46	0.07
Akap4	0.96	0.83	0.02	NA	0.71
Akap5	NA	0.94	0.94	1.59	0
Akap6	NA	0.89	0.97	0	1.45
Akap7	0.61	0	0.21	0.68	0
Akap8	0.14	0	2.94	1.13	0
Akap8l	0.31	1.97	2.58	3.15	0
Akap9	0.65	0.18	0.44	0	0.18
Akip1	NA	2.95	2.52	1.56	NA
Akirin1	NA	2.49	1.99	3.03	0
Akirin2	NA	1.43	1.79	0.19	2.07
Akna	NA	NA	2.05	0.78	0.49
Aknad1	NA	NA	0	0.95	NA
Akr1a1	NA	0.42	0.94	0.02	NA
Akr1b10	NA	2.29	4.69	1.61	1.27
Akr1d1	2.77	1.61	1.46	0	2.44
Akt1	0.01	1.99	2.1	0.69	1.65
Akt1s1	0.26	NA	0.81	2.5	0
Akt2	0.07	1.07	0.59	1.56	1.53
Akt3	0.65	0	2.22	0.3	0
Aktip	NA	0.51	1.63	NA	0
Alad	1.17	0.28	0.72	4.81	NA
Alas1	0.63	0.24	0	0.51	0

Alas2	3.17	1.53	1.07	1.85	1.26
Alb	NA	1.75	1.32	2.79	10.47
Alcam	0.4	0.69	1.44	2.09	0
Aldh16a1	NA	NA	0	0.4	0
Aldh18a1	NA	1.84	2.21	0.88	1.95
Aldh1a1	1.81	0.1	1.19	1.35	0
Aldh1a2	0.35	0.99	0.84	1.84	0
Aldh1a3	1.66	1.06	0.06	3.06	0
Aldh1b1	1	2.01	2.82	0	0
Aldh1l1	NA	1.21	1.15	0	6.24
Aldh1l2	NA	NA	1.41	1.01	0
Aldh2	0.87	0.06	0.72	0	0.53
Aldh3a1	1.31	0.58	0.75	0.76	0
Aldh3a2	0	0	0.05	0.78	3.53
Aldh3b1	0	2.36	1.57	NA	3.81
Aldh3b2	NA	0.79	2.59	0.44	5.25
Aldh4a1	0.39	1.58	1.08	0.96	1.5
Aldh5a1	2.52	0	4.4	1.06	0
Aldh6a1	0.44	0	1.26	NA	0
Aldh7a1	0	0.96	1.06	2.17	1.41
Aldh8a1	0.41	1.96	0.07	0	0.75
Aldh9a1	0	0	1.83	1.12	0
Aldoa	0.76	1.46	0.07	0.33	1.48
Aldob	1.49	1.57	0.44	1.04	3.64
Aldoc	0.25	0	2.57	1.97	1
Alg1	0	NA	0.19	NA	0.84
Alg10b	NA	NA	1.33	0	0.14
Alg11	NA	NA	NA	0.12	NA
Alg12	0	0	0.2	0	0
Alg13	NA	1.57	1.11	0.61	1.57
Alg14	NA	NA	0.43	0.77	NA
Alg2	0.6	NA	0.97	0.9	1.22
Alg3	0.2	1.92	3.55	2.61	0.29
Alg5	0.04	0.75	0.79	0.04	1.4
Alg6	0	2.12	2.36	1.14	0
Alg8	0	1.79	1.5	0.93	0
Alg9	NA	NA	1.07	0	0
Alk	0	1.1	1.77	1.85	3.11
Alkbh1	NA	0.4	1.86	NA	0
Alkbh2	NA	NA	0.35	1.2	0
Alkbh3	NA	NA	0.13	NA	2.13

Alkbh4	NA	1.02	0.14	2.85	1.65
Alkbh5	NA	NA	2.85	0.76	1.91
Alkbh6	NA	NA	1.91	1.23	2.67
Alkbh7	NA	NA	2.5	1.47	0
Alkbh8	NA	NA	0.28	1.29	0
Allc	0	1.39	1.28	5.99	0
Alms1	0	0	1.44	0	0
Alox12	0	0.46	1.28	1.34	0.32
Alox12b	6.42	1.21	1.3	1.13	NA
Alox15	0.89	0	3.84	0.65	0
Alox15b	0	NA	NA	NA	NA
Alox5	NA	0.28	0	0.82	0
Alox5ap	0.21	0.06	0	0.04	0
Aloxe3	0.85	0	0.72	1.73	2.35
Alpi	NA	0	0.25	0.77	1.69
Alpk1	NA	0	1.02	1.2	0
Alpk2	NA	NA	0.1	1.94	0
Alpk3	NA	0	1.84	1.75	3.09
Alpl	NA	3.26	2.03	1.32	0
Alppl2	NA	1.26	1.2	3.7	1.38
Als2	0	NA	0.15	NA	1.29
Als2cl	NA	1.36	1.96	1.69	0
Als2cr11	NA	NA	0.47	1.02	NA
Als2cr12	NA	NA	2.93	0	0
Als2cr13	0.02	NA	NA	NA	NA
Als2cr2	0	NA	NA	NA	NA
Als2cr4	NA	NA	NA	NA	0
Alx1	NA	1.07	0	0.12	1.36
Alx3	1.53	0.96	0.73	2.11	0
Alx4	0.59	4.23	5.47	2.42	0
Alyref	NA	NA	3.01	0.45	NA
Amac1	0	NA	NA	NA	NA
Amacr	0.61	0.54	2.59	NA	0.5
Ambn	1.06	2.48	3.1	0.95	4.22
Ambp	1.85	1.67	3.06	2.75	6.35
Ambra1	NA	0	0.71	1.71	0
Amd1	0.06	0	1.56	1.43	0
Amdhd1	NA	NA	1.36	1.69	1.31
Amdhd2	NA	2.97	1.57	0	0
Amelx	1.93	2.89	0.66	0.78	1.73
Amer1	NA	NA	0.92	0	NA

Amer2	NA	NA	1.35	0.73	NA
Amer3	NA	NA	0	0.18	NA
Amfr	1.3	1.5	3.16	1.55	NA
Amh	0.41	1.04	2.19	NA	4.87
Amhr2	0.69	0.89	0.54	0.02	0
Amica1	NA	NA	NA	2.65	NA
Amigo1	NA	NA	0.86	1.14	2.09
Amigo2	NA	0	0.49	NA	4.76
Amigo3	1.51	NA	NA	1.61	NA
Ammecr1	0	0.79	1.78	2.22	1.62
Ammecr1l	NA	NA	1.51	0.29	2.2
Amn	1.85	2.9	0.27	1.38	2.62
Amn1	NA	NA	0.96	NA	2.37
Amot	0.37	1.23	1.33	0.18	2.18
Amotl1	0.67	NA	1.26	4.15	0.89
Amotl2	0	0.05	1.26	1.44	0
Ampd1	NA	1.7	0.53	0.06	0
Ampd2	0.28	1.6	0.22	2.57	0
Ampd3	0.4	2.42	6.75	0.42	0
Amph	0.76	0.99	0.19	0.83	9.9
Amtn	NA	NA	NA	0.98	NA
Amz1	NA	NA	1.22	0.92	NA
Amz2	NA	1.7	1.7	0	0
Anapc1	0	0	0.77	0	0
Anapc10	0.81	0.21	1.55	2.05	0.89
Anapc11	0	NA	1.29	2.03	0.23
Anapc13	0.28	1.05	0.47	NA	0
Anapc15	NA	0.96	1.94	NA	NA
Anapc16	NA	NA	1.53	1.04	NA
Anapc2	0.98	0	3.81	0.04	0
Anapc4	0.3	NA	1.06	0.48	0
Anapc5	0.07	0	1.65	0.17	0
Anapc7	NA	NA	0.92	0.38	2.83
Ang	NA	2.57	1.92	NA	0.56
Angel1	NA	0	3.98	4.16	0.84
Angel2	NA	0.86	1.03	0.59	1.85
Angpt1	NA	1.09	1.95	2.29	1.16
Angpt2	NA	0.32	1.51	4	1.13
Angpt4	NA	0.65	1.55	3.23	1.83
Angptl1	1.55	NA	0.38	1.59	0.81
Angptl2	0	0.62	2.1	2.7	0

Angptl3	0.73	3.76	1.84	1.25	2.38
Angptl4	0.54	1.34	0.32	1.3	0
Angptl6	0.54	NA	1.74	NA	3.99
Angptl7	NA	2.12	0.69	1.15	4.31
Ank1	0.61	0.73	0	0.8	1.06
Ank2	1.67	0.51	0.42	9.76	0.92
Ank3	0.81	0.82	0.47	1.68	0.21
Ankar	NA	NA	NA	0.94	NA
Ankef1	NA	0.99	0.39	0.91	NA
Ankfn1	NA	NA	NA	4.09	NA
Ankfy1	0.42	1.6	1.45	0	0
Ankhd1	0.78	0.59	0.57	NA	1.1
Ankib1	NA	NA	0.96	0.64	0
Ankk1	NA	NA	NA	0	NA
Ankle1	NA	NA	0.51	1.37	NA
Ankle2	NA	0.56	1.44	0	0.6
Ankmy1	NA	NA	NA	0.47	NA
Ankmy2	0	0.71	0.8	0.16	0
Ankra2	0.1	0.21	0.61	1.06	4.22
Ankrd1	0	0.8	0	0.19	2.31
Ankrd10	0.12	0.96	0.63	1.48	0
Ankrd11	NA	1.11	0.94	0	0.84
Ankrd12	NA	0.8	0.56	1.84	0
Ankrd13a	NA	NA	8.34	0.57	0
Ankrd13b	NA	NA	2.96	2.24	5.55
Ankrd13c	NA	NA	0.41	0.64	3.01
Ankrd13d	NA	NA	2.32	3.76	0
Ankrd16	0.5	NA	1.25	1.62	0.2
Ankrd17	0.23	1.11	0.12	1.24	0.78
Ankrd2	6.88	0	0	0.29	3.94
Ankrd22	0	NA	1.22	0	0
Ankrd23	0.52	NA	1.2	NA	1.04
Ankrd24	NA	NA	0.65	3.2	0
Ankrd25	0	NA	NA	NA	NA
Ankrd26	NA	1.16	2.05	1.22	0.02
Ankrd27	0	0	0.8	4.02	2.4
Ankrd28	1.19	2.25	3.03	1.86	1.46
Ankrd29	0	NA	0.21	0.97	0
Ankrd31	NA	NA	1.39	NA	NA
Ankrd32	0.67	NA	0.3	0.59	1.94
Ankrd33	NA	NA	0.2	1.3	0

Ankrd33b	NA	NA	0.48	1.03	NA
Ankrd34a	NA	NA	0.87	2.9	0
Ankrd34b	NA	NA	NA	0.54	NA
Ankrd34c	NA	NA	NA	2.41	NA
Ankrd35	NA	NA	0.05	19.1	1.02
Ankrd36	NA	1.21	0.95	0.53	1.47
Ankrd37	NA	NA	2.33	2.15	1.61
Ankrd39	NA	NA	1.17	0.21	0
Ankrd40	NA	1.01	1.16	1.28	0.52
Ankrd42	NA	NA	0.29	0	2.5
Ankrd43	NA	NA	NA	NA	2.18
Ankrd44	NA	NA	1.68	0	1.29
Ankrd45	NA	NA	4.51	1.33	0
Ankrd46	NA	2.34	1.61	0.38	4
Ankrd49	NA	0.83	1.73	0.97	0
Ankrd5	1.04	NA	NA	NA	0.64
Ankrd50	NA	NA	1.1	3.78	0
Ankrd52	NA	NA	0.96	2.37	0.88
Ankrd53	NA	NA	NA	0.77	NA
Ankrd54	NA	NA	2.27	3.3	1.94
Ankrd55	NA	4.05	1.44	1.14	0
Ankrd57	NA	NA	NA	NA	1.87
Ankrd6	0.63	0.75	1.4	NA	1.51
Ankrd7	NA	3.79	0.17	2.94	1.72
Ankrd9	12.96	NA	0.96	0.24	0.09
Anks1b	NA	0	0.49	0.73	0
Anks3	NA	NA	0.77	0.02	2.05
Anks4b	NA	NA	0.01	1.51	0
Anks6	NA	NA	NA	0.43	NA
Ankzf1	NA	0	0.88	0	2.97
Anln	0.07	NA	1.54	0	0.75
Ano1	NA	0	0.04	2.57	0.6
Ano10	NA	0	0.26	0.95	0
Ano2	NA	NA	NA	1.28	NA
Ano3	NA	0.68	0.9	NA	1.33
Ano4	NA	NA	1.32	NA	0
Ano5	NA	NA	NA	1.23	NA
Ano6	NA	NA	1.95	1.48	2.06
Ano7	NA	NA	NA	1.19	NA
Ano8	NA	NA	2.34	0	NA
Ano9	NA	NA	1.87	1.67	2.98

Anp32a	0.73	0.18	1.92	0.97	2.15
Anp32b	0	3.01	1.88	1.07	0
Anp32e	1.14	0.61	1	0.66	0
Anpep	2.85	0	1.75	0	2.02
Antxr1	0.48	0.62	1.13	1.1	1.55
Antxr2	1.18	NA	2.06	2.52	0.11
Anubl1	3.28	NA	NA	NA	0.05
Anxa1	0.42	0.1	0.31	2.27	2.15
Anxa10	0	1.22	0	1.25	1.9
Anxa11	0	2.52	0.98	1.31	NA
Anxa13	0	1.2	2.19	0.88	8.84
Anxa2	0	1.65	1.5	4.7	3.2
Anxa3	0.94	0	0	1.36	0
Anxa4	1.14	0.43	3.98	1.47	0
Anxa5	0	0.54	0.77	0	0.5
Anxa6	0.74	0	0.5	0	0.81
Anxa7	0	1.65	4.28	2.56	0.6
Anxa8	9.74	NA	NA	NA	NA
Anxa9	NA	0.58	4.08	NA	1.52
Aoah	0.24	0	2.02	1.12	0
Aoc2	1.31	NA	NA	0.23	NA
Aoc3	0.26	0	8.64	1.2	1.83
Aof1	0	NA	NA	NA	NA
Aof2	0	NA	NA	NA	NA
Aox1	0.48	1.46	2.11	3.34	2.75
Ap1ar	NA	2.28	0.51	0.77	NA
Ap1b1	0.14	0.6	0.91	0	0
Ap1g1	0.69	0	1.46	0.24	0
Ap1g2	1.54	0.66	1.94	0	0
Ap1gbp1	1.4	NA	NA	NA	NA
Ap1m1	0.13	NA	2.38	0.01	0
Ap1m2	0.5	1.08	0.65	2.05	0
Ap1s1	0	0	0.72	0.2	0.69
Ap1s2	0	0.63	0.12	0.39	0.44
Ap1s3	0	NA	1.6	0.46	2.67
Ap2a1	0.59	NA	0	0.94	0.11
Ap2a2	0.14	1.16	0.64	0	0
Ap2b1	0.7	0.86	0.29	0.9	0.13
Ap2m1	0	0	0.69	0.06	0
Ap2s1	NA	1.43	1.85	0.04	0
Ap3b1	0.02	0	1.56	0.21	0

Ap3b2	1.28	2.04	0.97	0.84	0.09
Ap3d1	0.26	0.91	1.18	0	1.67
Ap3m1	0.28	NA	1.02	0.4	0
Ap3m2	3.12	0	0.68	0.67	1.21
Ap3s1	1.55	0.9	1.73	1.97	1.98
Ap3s2	0.37	1.56	0.35	1.51	1.76
Ap4b1	2.13	NA	1.7	0.44	0.7
Ap4e1	0.53	0.9	1.22	0.73	0
Ap4m1	0	1.42	7.13	0.45	3.98
Ap4s1	0.74	1.27	2.42	0.7	0.77
Ap5b1	NA	NA	0.78	1.09	NA
Ap5m1	NA	1.66	0.91	0.55	NA
Ap5s1	NA	2.27	1.1	0.77	NA
Ap5z1	NA	0.93	0.63	0.62	NA
Apaf1	0.07	0	0.76	0	0.61
Apba1	1.15	1.06	2.16	2.06	1.38
Apba2	0	0	0	1.49	0
Apba2bp	0.15	NA	NA	NA	NA
Apba3	0.27	1.47	1.49	1.6	0
Apbb1	0.72	0	0.82	0.82	0
Apbb1ip	0	0.8	2.22	0	1.74
Apbb2	0.72	0.59	2.08	1.45	0
Apbb3	3.92	3.02	0.13	7.59	2.69
Apc	0.61	1.31	0.1	0	0.15
Apc2	4.78	1.29	0.64	1.09	1.39
Apcdd1	NA	NA	3.11	4.86	0.84
Apcs	0.62	0	2.47	0	23.93
Apeg1	1.28	NA	NA	NA	NA
Apeh	0	0	0.02	0	1.68
Apex1	0.32	1.98	0.62	NA	6.18
Apex2	1.02	1.43	0	0.82	0
Aph1a	NA	0.07	2.11	0.05	0
Aph1b	NA	0	0.04	4.16	3.58
Api5	0	0.75	0.75	0.69	0
Apip	NA	0.97	2.23	2.48	0
Apitd1	NA	0.14	3.03	NA	2.54
Aplf	NA	NA	0.79	2.33	0.95
Apln	1.08	NA	0	0.73	4.59
Aplnr	NA	1.51	1.92	0.14	0
Aplp1	2.59	0.03	1.84	0.91	0
Aplp2	2.65	0.88	1.23	0.33	1.27

Apmap	NA	0	0.27	0.58	NA
Apoa1	0.45	1.61	0	0.64	4.65
Apoa1bp	0	NA	1.87	0	0
Apoa2	0	2.54	0.61	1.31	9.13
Apoa4	1.18	1.37	1.2	0.76	1.03
Apoa5	0	NA	1.55	0.61	2.59
Apob	1.76	1.3	0.79	0.98	3.04
Apob48r	0	NA	NA	NA	0
Apobec1	0.86	1.5	2.06	1.14	3.46
Apobec2	0	1.33	1.83	0.49	0
Apobec4	NA	NA	4.57	1.6	NA
Apobr	NA	2.61	1.65	1.32	NA
Apoc1	0.69	2.39	1.76	0	3.24
Apoc2	0.65	0.52	2.63	4.12	2.66
Apoc3	2.61	2.95	1.57	1.53	7.87
Apoc4	1.25	1.37	2.24	0.59	3.05
Apod	0.39	0.69	0.85	2.66	1.65
Apoe	8.52	2.19	0.74	NA	3.03
Apof	1.77	3.22	1.78	1	1.82
Apoh	4.32	0.66	0.79	1.18	0
Apol2	6.37	NA	NA	NA	NA
Apol6	NA	0	1.55	0	0
Apold1	NA	2.32	1.8	1.01	1.03
Apom	2.65	0	0.48	0.3	3.91
Apoo	NA	0	2.76	NA	0
Apool	NA	0.46	0.37	1.6	2.18
Apopt1	NA	NA	0.96	2.92	NA
App	0	1.37	0.93	0.14	1.36
Appbp1	0.83	NA	NA	NA	NA
Appbp2	1.97	0.32	0.7	0.78	0
Appl1	NA	1.28	2.19	0	0
Appl2	NA	0.65	8.6	0	0
Aprin	0.64	NA	NA	NA	NA
Aprt	0.1	2.67	1.56	1.84	0
Aptx	0.49	1.6	0.57	0.86	2.5
Aqp1	0	1.32	0.47	2.6	NA
Aqp11	0	NA	1.79	1.7	0
Aqp2	3.68	0.69	2.53	1.38	0
Aqp3	0.44	1.36	0.38	1.67	0
Aqp4	0.23	0	1.44	0.48	1.69
Aqp5	1.31	1.82	2.61	1.78	NA

Aqp6	1.58	0.89	0.53	1.54	0
Aqp7	1.12	1.15	1.81	0.95	1.8
Aqp8	0.21	3.12	1	0	2.87
Aqp9	0	4.46	3.35	0.87	4.24
Aqr	0.24	0.09	0.37	0.22	0
Ar	2.2	0.76	0.56	0.66	0.97
Araf	0.19	1.18	0.18	0.66	0.94
Arap1	NA	2.19	0.63	1.86	0.14
Arap2	NA	0.84	1.61	0	1.3
Arap3	NA	2.07	0.92	0	2.59
Arc	1.54	2.31	4.31	0.64	9.61
Arcn1	0	1.6	0.57	0.95	0.76
Areg	0	0.24	2.38	1.63	0
Arel1	NA	1.7	1.09	1.22	NA
Arf1	NA	1.24	0.07	1.89	0.33
Arf3	0	0.64	0.99	2.78	1.06
Arf4	0.84	0.13	1.7	0.11	0
Arf5	0	2.59	0.58	0.08	NA
Arf6	1.72	1.29	2.79	0.91	0
Arfgap1	0.57	1	0.23	0	0
Arfgap2	NA	0	1.01	NA	0
Arfgap3	0.78	0.9	0.52	1.1	0
Arfgef1	0.32	0.42	1.06	1.38	0.17
Arfgef2	1.2	0.28	1.55	0	1
Arfip1	0.64	0.08	2.26	0.13	6.51
Arfip2	2.2	0	0.22	0.74	0
Arfrp1	0.93	0.68	0.39	0.81	0.77
Arg1	0.59	3.31	1.46	1.82	1.54
Arg2	0.88	3.45	1.56	NA	0
Arglu1	NA	1.22	0.93	0.68	0
Arhgap1	0	0	2.22	0	0
Arhgap10	0	0	0.2	0.2	2.07
Arhgap11a	NA	0.06	2.84	0.68	2.64
Arhgap12	8.06	1.64	2.65	0.97	1.27
Arhgap15	0.76	1.72	1.1	0.88	1.74
Arhgap17	0.53	0	2.71	1.02	0
Arhgap18	0.25	NA	4.14	5.06	0
Arhgap19	0.97	1.18	1.75	NA	2.05
Arhgap20	0.92	NA	0.4	0	1.37
Arhgap21	1.1	NA	1.51	0	0.04
Arhgap22	0.46	2.93	2.03	0.36	0.17

Arhgap23	NA	NA	0.51	NA	0
Arhgap24	1.19	0.03	1.37	1.4	0
Arhgap25	0.02	2.04	0.03	0.58	7.57
Arhgap26	NA	0.27	1.65	2.41	1.23
Arhgap27	NA	NA	1.16	0.92	1.09
Arhgap28	NA	0.8	0.95	0	0
Arhgap29	NA	1.26	0.34	0.29	1.87
Arhgap30	NA	NA	2.54	0.68	0.78
Arhgap31	NA	NA	1	8.83	NA
Arhgap32	NA	0	1.25	0	NA
Arhgap33	NA	0.98	1.56	0.1	NA
Arhgap36	NA	NA	0	2.5	NA
Arhgap39	NA	NA	1.1	1.05	NA
Arhgap4	0.73	1.14	1.12	1.45	0.59
Arhgap40	NA	NA	NA	2.45	NA
Arhgap42	NA	NA	0.56	0.78	NA
Arhgap44	NA	0.55	1.2	0.54	NA
Arhgap5	0.42	1.03	1.78	0.49	0
Arhgap6	0.85	1.04	1.39	3.23	0.9
Arhgap8	0.3	NA	0.94	NA	2.51
Arhgap9	0	NA	1.71	1.41	1.51
Arhgdia	0	2.02	2.51	21.43	4.16
Arhgdib	0.19	1.06	0.78	0	1.19
Arhgdig	1.2	0.2	1.87	3.13	0
Arhgef1	0	0	0.37	2.93	0.25
Arhgef10	0.81	0.86	0.68	2.64	0.69
Arhgef10l	NA	2.97	1.81	0.36	0.02
Arhgef11	0.95	0	1.88	0	2.1
Arhgef12	0	1.26	1.63	0	0.72
Arhgef15	NA	1	0.43	1.08	9.81
Arhgef16	1.32	1.99	2.93	1.13	0
Arhgef17	1.97	0.74	0.75	0.98	0
Arhgef18	0	0	0.93	NA	1.51
Arhgef19	0	NA	2.98	0.82	0.24
Arhgef2	0	1.08	1.65	0	2.33
Arhgef25	NA	NA	0.65	1.87	NA
Arhgef26	NA	1.12	2.22	1.41	NA
Arhgef28	NA	1.06	0.49	0	NA
Arhgef3	0.56	0	0	0	4.06
Arhgef37	NA	NA	2.05	0	NA
Arhgef38	NA	0	3.1	2.11	NA

Arhgef39	NA	NA	2.42	NA	NA
Arhgef4	NA	0.64	3.1	1.28	1.59
Arhgef40	NA	0.84	0.39	1.5	NA
Arhgef5	0	1.63	0.69	NA	0
Arhgef6	0	1	0.79	0	1.94
Arhgef7	0.76	0	1.28	1.06	0.64
Arhgef9	NA	2.98	0.53	0.49	1.9
Arid1a	0	0	0.91	1.03	0.98
Arid1b	0.75	NA	0.82	1.22	0
Arid2	0.45	NA	0.98	1.9	1.45
Arid3a	0.57	1.03	3.26	1.92	0
Arid3b	0.58	0.32	0.36	11.13	0.95
Arid3c	NA	NA	NA	0.82	NA
Arid4a	0.63	0.47	1.37	0.56	2.88
Arid4b	1.15	0.43	0.59	0.09	0
Arid5a	0.54	0.47	1.35	1.05	0
Arid5b	0	1.31	1.42	0.09	0
Arih1	0.33	0.65	1.37	0	0.09
Arih2	0.74	0.6	0.26	0.41	2.09
Arl1	0.7	0.92	1.06	3.09	0.01
Arl10	NA	NA	1.06	1.01	0
Arl11	1.94	NA	NA	0.25	NA
Arl13a	NA	NA	NA	0	NA
Arl13b	NA	NA	0.69	1.46	0.17
Arl14	NA	3.06	1.32	0.98	1.06
Arl14ep	NA	NA	1.25	0.65	NA
Arl15	NA	0.63	1.87	0.08	0.48
Arl16	NA	NA	1.33	NA	0
Arl2	0	0	0.42	0	2.97
Arl2bp	0.05	0.53	2.17	1.19	4.38
Arl3	0.44	0.58	0.77	0.39	0
Arl4a	NA	2.36	0.8	3.25	2.65
Arl4c	NA	0	1.07	2.79	NA
Arl4d	NA	1.14	0.54	0.24	0
Arl5a	NA	1.23	1.09	0.9	2.37
Arl5b	NA	NA	0	0.07	0
Arl5c	NA	NA	NA	2.47	NA
Arl6	0.82	NA	1.92	2.99	0.94
Arl6ip1	0.09	1.5	3.19	0	2.48
Arl6ip2	0.23	NA	NA	NA	NA
Arl6ip4	0.1	0.98	2.19	1.56	NA

Arl6ip5	0	1.46	5.94	0	1.85
Arl6ip6	1.08	NA	1.13	0	2.57
Arl8a	NA	NA	0.22	1.38	2.76
Arl8b	NA	1.11	2.31	0	0.71
Arl9	0.53	NA	NA	0.11	NA
Armc1	0	0	0.54	0.07	0
Armc10	NA	NA	0.24	0.72	0
Armc12	NA	NA	1.21	0.86	NA
Armc2	NA	NA	2.06	0.66	0
Armc3	NA	NA	0.17	1.15	0.68
Armc4	0.85	0	1.51	0.57	0
Armc5	0.73	NA	0.59	1.41	0.28
Armc6	0	1.21	1.67	NA	0.78
Armc7	0.67	1.93	1.65	0.83	0
Armc8	0.45	0.27	1.65	0	0.56
Armc9	NA	0.97	0.26	0.85	0
Armcx1	1.36	1.53	1.29	1.76	2.54
Armcx2	0	0	1.91	0.55	0.09
Armcx3	0.01	0.02	2.11	0.75	0.44
Armcx4	NA	NA	NA	NA	0.36
Armcx5	NA	3.22	0.75	0	2.09
Armcx6	NA	1.51	1.25	0.7	NA
Armet	4.53	NA	NA	NA	NA
Arnt	0.89	1.08	1.2	0	0.7
Arnt2	1.31	1.4	1.39	1.29	0.59
Arntl	1.12	0.78	0.54	0	0.84
Arntl2	2.1	1.94	0.44	0.51	0
Arpc1a	0	4.95	3.58	0	0.05
Arpc1b	NA	0.29	1.17	0.61	0
Arpc2	0	0	0.05	0	0
Arpc3	0.58	1.31	3.15	0.37	0.01
Arpc4	0.28	1.35	6.06	NA	0
Arpc5	2.66	1	0.46	0.38	0.55
Arpc5l	0	1.49	1.91	0.73	2.72
Arpm1	1.81	NA	NA	NA	0.73
Arpp19	NA	0.68	1.75	0.62	0
Arpp21	NA	0	0.83	NA	NA
Arr3	0.44	0.54	0.45	0.74	0
Arrb1	0.11	0	2.31	0	1.48
Arrb2	0.24	0.02	5.33	1.39	0
Arrdc1	0	NA	1.43	1.47	0.14

Arrdc2	0.16	NA	2.29	0.8	8.65
Arrdc3	0.89	NA	1.83	1.34	0
Arrdc4	0.59	NA	0.89	0	2.54
Ars2	0.59	NA	NA	NA	NA
Arsa	1.37	2.19	2.98	0.84	2.31
Arsb	0.23	1.69	0.81	1.22	0
Arsg	NA	NA	0.82	0.43	NA
Arsi	NA	NA	0	0	3.04
Arsj	NA	0.45	1.07	0.92	1.23
Arsk	NA	NA	1.6	1.14	0
Art1	0	1	0	NA	0
Art3	0.32	0.81	0.62	0.28	0.84
Art4	2.25	2.68	1.53	5.13	1.51
Art5	0	NA	0.1	2.08	NA
Artn	0.34	1.18	0.2	1.38	6.56
Arv1	NA	NA	0.11	0	0
Arvcf	0.3	1.14	0.36	1.06	2.39
Arx	1.76	NA	0.7	0.87	0
As3mt	0.78	NA	2.47	2.78	0.58
Asah1	1.03	0.43	1.6	1.35	0.31
Asah2	0.21	NA	NA	NA	NA
Asah3	1.92	NA	NA	NA	NA
Asahl	1.58	NA	NA	NA	NA
Asap1	NA	1.11	1.31	2.96	0.64
Asap2	NA	1.12	2.17	2.83	0.31
Asap3	NA	1.68	3.56	0.16	1.28
Asb1	2.28	0.06	0.56	0	0
Asb10	2.6	NA	1.65	0.7	NA
Asb11	0.37	NA	4.99	3.14	NA
Asb12	0	NA	5.49	0.22	11.44
Asb13	0	0	2.22	0	0.25
Asb14	1.91	NA	0.84	2.2	0
Asb15	0.46	NA	1.2	1.54	NA
Asb16	2.07	NA	0.33	NA	NA
Asb17	1.18	NA	2.14	0	0
Asb18	1.71	NA	NA	5.75	NA
Asb2	1.1	NA	0.29	2.68	8.36
Asb3	0.94	NA	0	NA	NA
Asb4	0.36	0.6	1.78	1.63	1.11
Asb5	0.89	NA	1.03	0	1.96
Asb6	0.86	1.11	1.5	1.31	0

Asb7	0	0.11	0.78	3.57	0.29
Asb8	0.5	2.01	1.26	0.58	0.55
Asb9	0	0	0.72	0.97	0
Ascc1	0	3.16	2.73	0	2.76
Ascc2	NA	0	0	1.15	3.27
Ascc3	NA	1.42	0.44	0	3.72
Ascc3l1	0.62	NA	NA	NA	NA
Ascl1	0	0.85	0.81	1.19	2.16
Ascl2	0	1.3	0.31	3.35	0
Ascl3	0.84	1.67	0.01	0.43	0
Ascl4	NA	NA	NA	0.7	NA
Asf1a	0.23	0.07	0.85	0.83	0
Asf1b	0.06	0	1.82	0.89	0
Asgr1	0	3.26	2	0.88	0
Asgr2	0.49	2.4	1.07	1.52	0.24
Ash1l	0.35	0	0.83	NA	0.52
Ash2l	0	1.29	1.39	1.04	0
Asic1	NA	1.57	0.57	0.04	NA
Asic2	NA	1.3	5.48	0.81	NA
Asic3	NA	0.18	2.33	0.64	NA
Asic4	NA	0	0.53	1.01	NA
Asic5	NA	NA	2.56	0.78	NA
Asl	0.56	1.93	3.3	0.09	0
Asna1	0.08	1.2	0.81	1.11	0
Asns	2.25	2.91	2.36	0.72	1.4
Asnsd1	NA	1.91	1.63	0.37	1.35
Aspa	0.53	1.58	0.8	1.15	2.42
Aspdh	NA	NA	2.53	1.11	2.4
Aspg	NA	NA	2.01	2.16	NA
Asph	0.34	0	1.82	2.23	0.91
Asphd1	NA	1.44	3.26	0.02	4.86
Asphd2	NA	NA	2.26	0.15	0.82
Aspm	NA	1.29	3.03	0	0.8
Aspn	1.22	0.8	0.41	0.46	0.81
Asprv1	NA	NA	0.82	1.57	0
Aspscr1	0	0	2.71	0	1.72
Asrgl1	0	0.84	1.31	0.54	2.2
Ass1	0.08	0	1.44	NA	0
Aste1	NA	2.32	2.24	1.65	3.95
Astl	1.99	NA	NA	1.02	NA
Astn1	0.56	2.06	1.42	2.05	0.92

Astn2	0.35	1.28	1.67	1.35	6.45
Asun	NA	0.45	1.38	2.11	NA
Asxl1	0.48	2.25	1.05	0	2.99
Asxl2	0.37	1.12	0.35	0	0
Asxl3	NA	NA	0.1	1.69	1.98
Asz1	5.87	NA	0.66	9.08	NA
Atad1	0.99	NA	1.24	0.78	1.38
Atad2	0.1	1.01	1.46	0	0.87
Atad2b	NA	1.85	0.17	0.43	0
Atad3a	0	1.67	0.51	1.95	0
Atad5	NA	1.67	0.96	0.82	0
Atat1	NA	1.16	1.77	0	NA
Atbf1	0	NA	NA	NA	NA
Atcay	3.48	NA	1.35	1.17	0.24
Ate1	0.55	NA	0.06	1.46	0
Atf1	0.14	3.15	2.52	0.06	0
Atf2	0	0.6	0.46	2.2	1.04
Atf3	0.07	0.58	1.63	1.17	11.87
Atf4	2.31	1.25	1.77	0	0
Atf5	0.57	0.86	0.75	NA	0
Atf6	0.5	0.1	0.56	1.56	0.95
Atf6b	NA	1.77	0	NA	0.15
Atf7	1.29	1.31	1.03	2.31	1.67
Atf7ip	1.3	0.64	0.55	1.47	1.4
Atf7ip2	0.36	2.6	1.21	2.05	3.09
Atg10	NA	1.63	0.8	1.86	3.12
Atg12	NA	1.99	1.58	0.81	0
Atg13	NA	0	0.57	6.12	NA
Atg14	NA	NA	NA	0	NA
Atg16l1	NA	0	0	1.06	3.17
Atg16l2	NA	NA	0.04	0.18	5.14
Atg2a	NA	0	0.86	0.09	0
Atg2b	NA	0.41	0.42	0	2.14
Atg3	NA	0	2.27	0.1	0
Atg4a	NA	2.65	0.99	0.9	0.58
Atg4b	NA	0.19	1.85	0.32	2.01
Atg4c	NA	NA	1.69	1.01	NA
Atg4d	NA	NA	1.37	1	1.11
Atg5	NA	1.16	1.66	0.07	3.61
Atg7	NA	0	1.22	0	0
Atg9a	NA	1.39	3.87	0	0.3

Atg9b	NA	NA	0.87	1.62	1.33
Athl1	NA	1.87	0.67	0	0
Atic	0.25	0.74	0.36	2.3	0
Atl1	NA	NA	2.92	1.27	1.74
Atl2	NA	NA	0.2	0.57	1.31
Atl3	NA	NA	0.87	1.17	0.69
Atm	0	0	0	NA	1.75
Atmin	NA	0.07	1.12	0.41	0
Atoh1	0.26	3.13	0	0.18	0
Atoh7	0.91	NA	0	1.63	NA
Atoh8	1.1	NA	1.59	0.75	2.44
Atox1	1.65	0	0.83	0.46	0
Atp10a	0.4	1.49	0.48	3.1	0.73
Atp10b	NA	0	1.1	0.65	1.51
Atp10d	1.26	0.95	0.4	0	3.53
Atp11a	0.03	0.73	0.32	0	0
Atp11b	0	0	1.25	1.35	0
Atp11c	0.53	NA	1.39	0	0.98
Atp12a	3.48	1.9	2.91	1.48	5.8
Atp13a1	NA	0	0.78	1.88	0
Atp13a2	NA	0.78	2.98	0	0
Atp13a3	NA	0.01	1.71	0	0.58
Atp13a4	NA	NA	0.84	0.13	NA
Atp13a5	NA	NA	0.97	0	0
Atp1a1	0.2	2.82	1.47	2.81	3.92
Atp1a2	1.12	0.58	0.52	0	1.6
Atp1a3	NA	1.19	1.05	0.68	2.15
Atp1a4	7.38	NA	1.29	0.01	NA
Atp1b1	0.06	0.94	0.34	2.48	2.11
Atp1b2	0.07	1.17	3.25	1.99	2.38
Atp1b3	0.98	1.81	1.94	0.9	0.25
Atp1b4	6.07	1.27	1.29	2.72	0
Atp2a1	0	0.82	1.24	0	0
Atp2a2	0.65	0.65	0.2	0	0.74
Atp2a3	0.38	0	2.38	0.15	0.54
Atp2b1	NA	2.26	0.07	0	2.29
Atp2b2	0.53	0.38	0.31	0.98	1.23
Atp2b3	0.47	1.53	1.38	0.76	7.27
Atp2b4	NA	0.73	1.02	0	0
Atp2c1	0	0.73	1.59	0.93	1.39
Atp2c2	NA	4.35	0	0.11	0

Atp4a	0	0	0.17	0.89	0
Atp4b	0	1.28	1.98	1.48	0
Atp5a1	0	0	1.16	0.03	0
Atp5b	0.23	1.43	0.58	0.14	0
Atp5c1	0.04	1.13	0.82	1.5	0
Atp5d	0.15	1.94	2.67	0.79	0
Atp5e	0	0.94	1.81	0.89	0
Atp5f1	0	0	1.53	1.1	3.51
Atp5g1	0.38	1.74	0.6	0.43	0
Atp5g2	0	0	0	0	0
Atp5g3	0.26	1.59	0.96	0	0.01
Atp5h	0.19	1.58	1.99	0	0
Atp5j	0	0	0.74	NA	1.28
Atp5j2	0.13	1.71	1.7	0.07	0.16
Atp5l	0	NA	NA	0.71	NA
Atp5o	0.29	0	0.19	0	NA
Atp5s	0.74	2.04	0.55	0	0
Atp5sl	NA	2.76	0.23	0.79	0
Atp6ap1	0.3	1.4	0.15	1.32	5.16
Atp6ap1l	NA	NA	NA	1.39	NA
Atp6ap2	0.41	1.13	2.49	0.78	0
Atp6v0a1	0	0	0.25	3.4	2.11
Atp6v0a2	0	0.83	0.81	1.46	0
Atp6v0a4	0.43	1.11	0	2.68	2
Atp6v0b	1.79	0	0.53	0.81	1.64
Atp6v0c	0	2.71	3.06	0.36	0.32
Atp6v0d1	0	1.32	3.26	1.64	1.11
Atp6v0d2	3.42	NA	1.49	0.61	NA
Atp6v0e2	NA	0	0.65	1.33	0
Atp6v1a	NA	2.66	1.07	1.97	3.39
Atp6v1b1	1.21	0.08	0	2.96	2.16
Atp6v1b2	0.02	0.75	2.04	0.63	3.3
Atp6v1c1	0.1	0	1.45	0.17	5.87
Atp6v1c2	0	NA	3.94	1.12	NA
Atp6v1d	1.12	0	0.9	0.97	1.46
Atp6v1e1	1.24	0	1.07	1.56	0.57
Atp6v1e2	0	NA	1.34	0.64	NA
Atp6v1f	1.11	2.2	1.7	0	0
Atp6v1g1	1.31	0	0.46	1.7	2.92
Atp6v1g2	0.18	3.2	0.83	NA	3.3
Atp6v1g3	0	NA	NA	2.47	NA

Atp6v1h	0	0.07	1.87	0.96	0
Atp7a	1.24	1.64	2.72	1.69	0
Atp7b	0.39	0.59	1	0.51	0
Atp8a1	0.65	0.89	1.09	1.33	0.06
Atp8a2	0.76	0	0.54	1.38	0.9
Atp8b1	0.34	1.37	2.17	0	0
Atp8b2	1.15	1.51	0.67	1.66	1.72
Atp8b3	0	1.53	0.32	0.19	3.35
Atp8b4	0	0.79	0.21	0.59	0.52
Atp9a	0	0.6	0.06	0	0
Atp9b	0.47	0.76	0.84	0	0
Atpaf1	4.3	NA	1.55	0.62	0
Atpaf2	0.05	0	1.31	0.71	1.69
Atpbd4	NA	NA	NA	NA	1.47
Atpif1	0.12	1.5	0.88	1.05	0
Atr	0.24	0	1.08	0	1.98
Atraid	NA	2.21	1.93	2.36	NA
Atrip	NA	NA	1.37	2.89	NA
Atrn	0.05	1.62	1.29	0	2.23
Atrnl1	0.3	1.9	1.05	0.37	2.31
Atrx	0.15	0.39	0.82	0	0.07
Atxn1	NA	0.59	2.07	1.06	0
Atxn10	NA	2.16	1.76	0.42	3.23
Atxn1l	NA	NA	0.01	NA	1.28
Atxn2	NA	0	0.94	1.4	0
Atxn2l	NA	0.27	0.02	1.74	1.06
Atxn3	NA	1.08	1.65	1.66	1.35
Atxn7	NA	0.86	0.87	1.73	0
Atxn7l1	NA	0.67	1.51	0.88	1.18
Atxn7l2	0	NA	0.82	2.12	0
Atxn7l3	NA	NA	3.58	1.68	2.97
Atxn7l3b	NA	1.27	0.95	0.39	NA
Atxn7l4	0	NA	NA	NA	NA
Auh	0.21	1.41	0.18	0.11	0
Aup1	0.49	1.89	1.49	2.5	2.01
Aurka	NA	0.32	2.66	0.39	1.62
Aurkaip1	NA	0	2.77	1.35	0
Aurkb	0.9	0.52	2.69	0.31	3.89
Aurkc	1.62	2	2.08	2.13	2.68
Auts2	NA	0.9	0.6	0	0
Aven	1.12	1.29	1.85	1.22	0

Avil	0	0.09	3.76	0	3.66
Avl9	NA	1	0.5	0	0
Avp	0	1.6	0.25	2.65	0.04
Avpi1	0.2	3.93	4.1	1.91	5.68
Avpr1a	0	0.73	1.2	1.1	0
Avpr1b	1.32	1.69	1.31	2.18	0
Avpr2	0.29	0.33	1.98	NA	0
Awat1	NA	NA	NA	0.76	NA
Awat2	NA	NA	NA	3.87	NA
Axdnd1	NA	NA	0	0.03	NA
Axin1	NA	0	3.88	1.39	NA
Axin2	2.59	NA	0	0	1.34
Axl	0	0.8	1.87	4.44	0
Axud1	0.09	NA	NA	NA	NA
Azgp1	1.69	1.62	0.5	2.3	3.68
Azi1	0.12	0.23	3.16	0.31	0
Azi2	0.71	1.8	3.01	NA	0
Azin1	NA	1.69	1.42	3.56	4.41
B2m	0.24	0.14	1.78	6.2	0.75
B3galnt1	NA	0.28	1.07	0.26	0
B3galnt2	NA	NA	0	2.28	0
B3galt1	0.47	0.56	0	0.98	2.57
B3galt2	0	0.77	0.59	0.91	0.18
B3galt3	1.06	NA	NA	NA	NA
B3galt4	0	3.84	1.19	1.25	0
B3galt5	0	0.16	0.92	2.66	0
B3galt6	0	NA	2.65	0.84	0.51
B3galtl	NA	NA	1.8	0.68	0.46
B3gat1	0.01	1.11	0.37	2.01	0.21
B3gat2	0	NA	0.82	0.53	0.69
B3gat3	2.44	0.82	2.1	1.45	1.02
B3gnt1	0	0	2.12	0.39	0.95
B3gnt2	NA	0.79	1.29	0	0.42
B3gnt3	3.84	1.51	2	0.47	0
B3gnt4	0	1.34	1.26	0	2.67
B3gnt5	0	NA	0.76	0.04	2.78
B3gnt6	0.21	NA	1.81	2.09	NA
B3gnt7	1.6	NA	0	0.25	0.51
B3gnt8	NA	NA	4.39	0.77	1.68
B3gnt9	NA	NA	3.6	1.03	0
B3gntl1	NA	2.5	1.52	1.15	1.6

B4galnt1	NA	1.21	3.17	1.01	2.15
B4galnt2	NA	NA	0.8	1.98	NA
B4galnt3	NA	NA	NA	2.27	NA
B4galnt4	NA	NA	0.77	3.13	0
B4galt1	0.89	0.05	3.24	1.73	0
B4galt2	0.85	2.4	0.79	NA	0
B4galt3	0	3.06	2.79	2.39	1.69
B4galt4	1.83	0	0.64	2.78	3.58
B4galt5	0	0	1.74	1.93	0
B4galt6	1.13	1.17	1.81	0.33	0
B4galt7	0.31	0	0.96	2.41	0
B9d1	NA	1.5	0.01	2.06	5.24
B9d2	NA	0	4.37	NA	0
Baalc	0.62	1.4	1.81	2.32	1.32
Baat	2.99	1.57	0	0.17	1.33
Babam1	NA	0	3.58	0	NA
Bace1	2.48	1.13	0.33	2.09	0.01
Bace2	1.33	0	0.46	1.44	0.06
Bach1	0.85	2.13	1.4	1.26	0
Bach2	3.02	0	2.43	1.31	1.86
Bad	0	0	0.58	NA	1.07
Bag1	0.22	0	1.04	1.44	1.69
Bag2	1.67	0.42	1.18	0.64	2.37
Bag3	0	2.14	1.84	0.08	0
Bag4	0	2.5	0.52	1.2	1.5
Bag5	0.71	0.44	0.15	0.93	NA
Bag6	NA	0	2.29	1.44	NA
Bahcc1	NA	2.93	0.96	1.47	6.45
Bahd1	NA	0	0.12	1	0
Bai1	1.02	2.46	2.46	1.28	1.86
Bai2	0.9	1.55	0.77	0	NA
Bai3	0.62	0.12	0.54	1.06	2.63
Baiap2	0	0.54	1.84	0	0
Baiap2l1	NA	NA	0.31	0.05	1.08
Baiap2l2	NA	NA	NA	0.97	NA
Baiap3	NA	0.94	1.79	0.21	2.25
Bak1	2.64	2.24	0.26	1.33	0.51
Bambi	0.28	0.74	2.5	0.23	2.06
Banf1	0.15	2.49	0.12	0	2.45
Banf2	NA	NA	1.2	0.94	1.23
Bank1	0.08	0	3.63	0	0

Banp	NA	0	4.57	3.98	1.49
Bap1	0	0.18	2.7	1.83	1.07
Bapx1	0.59	NA	NA	NA	NA
Bard1	0.37	1.54	3.09	0.74	1.43
Barhl1	1.49	NA	2.55	1.96	2.65
Barhl2	NA	NA	NA	2.45	NA
Barx1	0.18	4.14	0.33	1.47	0
Barx2	2.36	1.84	0.86	0.5	0.43
Basp1	0.59	0.46	1.21	0.59	NA
Bat2	2.89	NA	NA	NA	0
Bat2l	NA	NA	NA	NA	1.65
Bat3	2.76	NA	NA	NA	2.86
Bat4	0.24	NA	NA	NA	1.11
Bat5	0	NA	NA	NA	1.72
Batf	0.49	0.01	2.93	2.02	0
Batf2	NA	NA	1.91	1.05	0
Batf3	NA	0	1.09	0	4.19
Bax	1.53	1.61	1.59	1.92	0
Baz1a	0	0	0.24	0	0
Baz1b	0.24	0.39	0.54	0.06	0.01
Baz2a	0.46	0.77	0.54	1.07	0.94
Baz2b	NA	0.34	1.44	0.33	1.01
Bbc3	0.83	1.86	2.21	1.58	0.68
Bbox1	0.57	2.88	0.08	0	1.99
Bbs1	0	6.32	3.92	NA	2.3
Bbs10	NA	2.11	1.19	1.2	0
Bbs12	NA	NA	2.39	0.93	1.37
Bbs2	1.19	NA	2.21	0	2.41
Bbs4	0.02	1.01	0	0.75	0.87
Bbs5	NA	NA	1.94	0	2.24
Bbs7	2.66	0.19	0.74	0.85	0.65
Bbs9	NA	0	0.41	1.15	1.64
Bbx	0	1.17	1.34	1.48	1.76
Bcam	NA	1.43	0.05	0.07	5.18
Bcan	0.29	0.88	0.97	2.81	0
Bcap29	0.28	0	0.53	0.04	0
Bcap31	0	1.41	1.28	0.37	0.96
Bcar1	0.7	NA	1.4	1.25	0.68
Bcar3	0.47	1.82	0.53	2.94	0
Bcas1	1.34	0	0.93	5.3	1.41
Bcas2	0.86	1.15	0.42	0.66	0

Bcas3	0.51	0.23	2.24	0.17	0
Bcat1	0.51	0	3.78	0.59	0.64
Bcat2	0	1.77	1.04	3.79	1.18
Bccip	0	0	1.44	0.53	0
Bcdin3d	NA	NA	3.26	2.72	1.68
Bcdo2	2.48	NA	NA	NA	NA
Bche	1.44	0.82	0.8	0.46	1.51
Bckdha	0.23	0	6.3	3.64	0
Bckdhb	0	0	1.15	0.79	0
Bckdk	1.06	1.38	2.16	0.77	0.54
Bcl10	0.45	0.87	1.13	3.98	0.83
Bcl11a	0.55	0	1.93	0.83	1.77
Bcl11b	0.49	0	0.39	2.58	1.49
Bcl2	0.27	0.03	1.86	1.46	0.71
Bcl2l1	0.33	0	1.39	1.61	3.38
Bcl2l10	0.15	1.05	1.69	1.31	2.68
Bcl2l11	0.54	1.15	1.66	0.53	0.57
Bcl2l12	0	NA	0.52	0.1	0.6
Bcl2l13	1.27	0	0.41	1.13	0.97
Bcl2l14	0.38	1.37	0.74	3.37	0
Bcl2l15	NA	NA	0.1	3.11	0
Bcl2l2	0.9	0.83	1.73	0.79	0
Bcl3	1.77	1.49	2.85	0.15	0
Bcl6	0	0.05	2.28	0	0
Bcl6b	0	NA	1.67	0.25	0
Bcl7a	0.75	0	1.73	1.84	0.81
Bcl7b	0	2.05	2.01	1.27	0
Bcl7c	0.04	0.57	0.83	0.25	0.44
Bcl9	0	0	1.24	1.54	1.85
Bcl9l	0.99	NA	1.55	0	0
Bclaf1	0	0.64	1.13	0.87	0.87
Bcmo1	2.8	2.46	0	0.43	1.92
Bco2	NA	NA	2.92	4.04	0
Bcor	0.21	0	0.84	3.61	2.76
Bcorl1	0.26	2.27	1.61	2.51	0
Bcr	0.39	0	0.67	1.04	1.48
Bcs1l	0.31	2.5	3.12	0.81	6.31
Bdh1	NA	0	0.68	1.42	0.62
Bdh2	NA	0	1.68	0.54	0
Bdkrb1	0.97	1.62	0.56	0	6.58
Bdkrb2	0	0.42	0.82	2.63	1.94

Bdnf	0.25	1.92	1.68	0	0.8
Bdp1	NA	NA	1.93	2.5	0
Bean	1.82	NA	NA	NA	0.9
Bean1	NA	1.37	0.37	0.7	NA
Becn1	0.16	0.66	0.61	NA	0.89
Begain	NA	NA	NA	1.15	NA
Bend3	NA	NA	1.51	2.74	2.01
Bend4	NA	NA	0.36	1.89	0
Bend5	NA	0.14	1.03	1.41	0
Bend6	NA	NA	0	0.11	0
Bend7	NA	NA	1.29	0.44	0
Best1	NA	2.79	1.3	1.47	0
Best2	NA	0.71	0.21	0.99	0
Best3	NA	NA	NA	0.04	NA
Bet1	0	1.54	2.27	0.75	0
Bet1l	0.61	3.07	0.65	1.69	0
Bex1	NA	0.06	5.16	0	0
Bex2	NA	NA	3.76	1.89	4.73
Bex4	NA	1.08	0.41	0.88	2.29
Bfar	0.51	0	2.7	1.29	1.42
Bfsp1	2.66	0.97	1.15	1.67	NA
Bfsp2	1.45	2.81	0.3	1.8	0
Bglap	NA	NA	NA	1.05	NA
Bgn	0.2	0.77	2.17	0.26	0
Bhlha15	NA	NA	NA	1.29	NA
Bhlha9	NA	NA	NA	2.1	NA
Bhlhb2	0.44	NA	NA	NA	NA
Bhlhb3	1.2	NA	NA	NA	NA
Bhlhb4	0	NA	NA	NA	NA
Bhlhb5	0	NA	NA	NA	NA
Bhlhb9	0.13	0.23	1.47	1.4	3.23
Bhlhe22	NA	NA	0.55	0	0
Bhlhe23	NA	NA	0.76	1.65	0
Bhlhe40	NA	2.91	3.42	0.04	2.62
Bhlhe41	NA	0	0.44	1.65	2.01
Bhmt	0.75	1.82	0.31	1.62	3.23
Bhmt2	1.62	1.52	0.71	1.99	0.93
Bicc1	1.06	0.51	0.91	3.15	1.1
Bicd1	1.17	1.98	0.6	1.91	1.08
Bicd2	0.44	0.25	1.62	8.64	1.87
Bid	0.7	3.67	3.15	1.08	3.28

Bik	NA	0.9	4	0.69	1.84
Bin1	0	0	0.71	0.26	1.54
Bin2	NA	NA	NA	1.48	NA
Bin3	0	0.09	0.63	0	0
Birc2	0	2.28	4.56	0	0
Birc3	0.02	2.52	0.77	1.09	0.36
Birc4	0.76	NA	NA	NA	NA
Birc5	0.62	1.12	2.92	1.31	2.18
Birc6	0.26	NA	0.43	1.3	2.01
Birc7	0.9	2.38	0.92	1.57	2.44
Bivm	0	NA	1.36	0.23	0.88
Blcap	1.23	2.38	3.04	2.95	8.75
Blk	1.68	0.32	0.45	0.95	0
Blm	0.07	1.46	2.92	0	1.68
Blmh	0.86	0	1.65	0.91	0
Blnk	0.15	0	1.9	0	1.8
Bloc1s1	0	NA	NA	0	0
Bloc1s2	0.42	NA	0.82	0	0
Bloc1s3	0.28	NA	1.77	0.91	1.93
Bloc1s4	NA	NA	1.38	1.3	NA
Bloc1s5	NA	NA	0.74	NA	NA
Bloc1s6	NA	NA	0.08	0.63	NA
Blr1	0.29	NA	NA	NA	NA
Blvra	0.43	0	1.66	1.98	0.5
Blvrb	0.42	0.99	0.01	0.89	0
Blzf1	3.62	0.52	1.76	0.86	0
Bmf	0	NA	1.52	0.35	1.74
Bmi1	0	0.02	2	0.99	0
Bmp1	0.62	1.02	1.57	0.67	1.8
Bmp10	2.66	2.04	1.2	0.06	0
Bmp15	0.6	1.1	0.78	2.08	1.92
Bmp2	0.72	1.52	1.2	0	0
Bmp2k	0.8	1.2	1.23	0	1.64
Bmp3	0.74	NA	NA	0	NA
Bmp4	0.31	0.6	2.04	0	0.17
Bmp5	0	0.37	0.81	2.32	2.45
Bmp6	0	1.02	0.15	0.85	3.57
Bmp7	0.6	1	0	2.8	1.97
Bmp8a	0.76	0.6	1.16	1.12	0
Bmp8b	4.11	0.91	0.92	4.77	1.23
Bmper	2.89	NA	0	1.73	0.77

Bmpr1a	NA	1	0.41	1.03	1.77
Bmpr1b	0.44	0.28	0.71	0	1.13
Bmpr2	0	0.13	0.5	0.57	0.82
Bms1	NA	0	0.08	1.33	2.45
Bms1l	0	NA	NA	NA	NA
Bmx	0.74	0	0.77	0.86	0
Bnc1	0.55	1.46	2.92	0	2.37
Bnc2	1.63	1.85	0.5	2.59	0.88
Bnip1	0.38	1.57	0.37	0	0
Bnip2	0.01	0.22	0.8	0.89	0
Bnip3	0	1.61	3.45	2.37	0
Bnip3l	0.47	0.96	2.13	2.01	0
Bnipl	0.21	NA	0.6	0	0
Boc	0.37	NA	0.46	1.37	1.11
Bod1	NA	NA	2.2	0.09	0
Bod1l	NA	NA	NA	NA	0.64
Bok	1.35	2.42	0.86	2.43	0
Bola1	NA	0.42	1.17	2.25	0
Bola2	NA	NA	1.52	NA	0.25
Bola3	NA	NA	2.08	0	0
Boll	1.38	NA	0.45	0.8	0.93
Bop1	0.31	1.16	2.48	1.58	NA
Bora	NA	0.12	1.78	1.9	NA
Bpgm	0.19	1.26	1.61	1.43	0
Bphl	0	0	0.78	NA	0
Bpi	NA	NA	NA	1.92	NA
Bpifa1	NA	0.99	1.17	0.25	NA
Bpifa2	NA	NA	0.34	1.56	NA
Bpifa3	NA	NA	0	0.92	NA
Bpifb1	NA	NA	1.74	1.89	NA
Bpifb2	NA	NA	0	0.55	NA
Bpifb3	NA	NA	NA	1.03	NA
Bpifb4	NA	NA	NA	0.9	NA
Bpifb6	NA	NA	NA	0.72	NA
Bpifc	NA	NA	1.23	1.32	NA
Bpil1	0	NA	NA	NA	0
Bpil2	0.19	NA	NA	NA	NA
Bpil3	0	NA	NA	NA	NA
Bpnt1	0	2.53	1.76	0.16	1.32
Bptf	NA	0	1.04	1.66	0
Braf	0.77	0.22	1.13	2.15	0

Brap	0.84	2.56	0.31	0.69	0
Brat1	NA	NA	0.26	0.79	NA
Brca1	0	0.57	2.23	0.5	0
Brca2	0	2.41	2.16	0.27	0
Brcc3	NA	0.1	0	0.78	0
Brd1	1.87	0	1.5	1.3	1.58
Brd2	0.39	0	2.02	0.88	0.07
Brd3	0.63	0	0.98	1.13	0
Brd4	0.27	0	0.09	0.8	0
Brd7	0.81	0.22	0.23	0.28	NA
Brd8	0.19	1.13	1.35	0.15	0
Brd9	NA	0	3.7	0.11	2.33
Brdt	0.76	1.92	0	0.2	0
Bre	0.01	1.28	2.02	5.33	0.49
Brf1	1.39	1.78	1	0	0.56
Brf2	0	1.54	1.99	NA	3.64
Bri3	0.63	NA	0.86	0.09	0
Bri3bp	0.51	NA	1.53	7.67	0
Bricd5	NA	NA	NA	1.43	NA
Brip1	1.16	0	2.29	1.39	1.39
Brix1	NA	0.98	2.41	0	NA
Brk1	NA	NA	0.04	1.21	NA
Brms1	0	1.26	0.55	2.21	3.42
Brms1l	1.2	NA	0.51	0.44	0
Brox	NA	NA	1.67	0.4	NA
Brp44	NA	NA	NA	NA	0
Brp44l	0	NA	NA	NA	3.02
Brpf1	0.95	3.41	1.24	2.14	2.82
Brpf3	NA	NA	1.17	1.21	1.14
Brs3	1.36	1.16	1.64	2.77	0
Brsk1	NA	NA	0	1.23	NA
Brsk2	NA	1.22	0.53	1.57	0
Brunol4	0	NA	NA	NA	NA
Brunol6	0	NA	NA	NA	NA
Brwd1	NA	1.77	3.72	0.76	0
Brwd3	NA	NA	0.47	2.52	0
Bscl2	0.42	0.85	1.4	0	0.74
Bsdc1	NA	0.45	0.61	0.68	0
Bsg	1.98	0.56	0.34	0	1.18
Bsn	1.13	1.9	0.72	0.02	0
Bsnd	1.07	NA	0.84	0.97	NA

Bsph1	NA	NA	NA	0.83	NA
Bspry	0.79	1.91	0.81	1.6	0
Bst1	3.76	0	2.27	0.44	0
Bst2	0	0.51	2.35	0.05	0
Bsx	NA	NA	NA	0	NA
Btaf1	NA	2.01	0.7	0.85	0
Btbd1	0.37	0.17	0.87	1.06	3.62
Btbd10	NA	NA	3.2	0.66	NA
Btbd11	0.13	NA	0.14	2.67	1.81
Btbd12	1.32	NA	NA	NA	1.63
Btbd14a	1.74	NA	NA	NA	NA
Btbd14b	0.27	NA	NA	NA	NA
Btbd16	NA	NA	NA	1.18	NA
Btbd17	NA	NA	1.95	NA	0
Btbd19	NA	NA	1.75	NA	NA
Btbd2	0.11	0.17	1.21	0.52	2.06
Btbd3	0.39	0.27	1.7	1.28	0.36
Btbd6	0	NA	1.56	0.28	0
Btbd7	0.46	0.71	0.56	1.01	1.51
Btbd9	1.04	NA	1.93	0	0
Btc	2.57	1.74	1.16	0	2.59
Btd	0	0.84	3.71	0.73	0.62
Btf3	0.21	1.63	0.98	0	0
Btf3l4	NA	NA	1.61	0.93	0
Btg1	0	1.99	2.5	0	2.07
Btg2	0	1.76	0.99	1.9	0
Btg3	0	NA	NA	NA	NA
Btg4	0.29	1.96	4.62	1.75	4.38
Btk	0.63	1.7	1.53	14.06	0
Btla	0.75	NA	0.82	0.66	3.89
Btn1a1	0	0.63	0.14	0	0
Btn2a2	NA	NA	NA	2.85	NA
Btnl2	NA	1.6	1.59	0.71	0
Btnl9	NA	NA	0.96	1.33	1.44
Btrc	0	0.6	0.91	0	0.86
Bub1	1.57	0	2.43	0.83	0
Bub1b	0.57	0.3	2.1	NA	0.74
Bub3	0.6	1.08	0.61	0.9	0
Bud13	NA	NA	2.52	5.39	3.24
Bud31	NA	0	2	0.29	0
Bves	0	NA	0.57	1	0

Bxdc1	0	NA	NA	NA	NA
Bysl	0	2.2	1.46	2.7	2.05
Bzrap1	1.16	1.38	4.64	0	2.47
Bzrpl1	1.75	NA	NA	NA	NA
Bzw1	0.27	1.11	3.92	0	0
Bzw2	1.03	0	0	0.46	0
C1d	NA	0.41	1.1	2	0.66
C1galt1	1.02	3.43	1.66	1.53	2.96
C1galt1c1	NA	0	0.65	3.35	1.75
C1qa	0.69	3.23	1.81	0.6	0
C1qb	0.7	1.19	2.51	1.37	1.57
C1qbp	0.49	2.06	3.15	1.08	2.98
C1qc	NA	NA	2.53	1.35	1.88
C1qdc1	0	NA	NA	NA	NA
C1ql1	0	0	1.56	2.1	2.31
C1ql2	NA	NA	NA	1.41	NA
C1ql3	0	NA	NA	0.3	NA
C1ql4	NA	NA	NA	2.89	NA
C1qtnf1	0	1.03	0.51	1.51	5.3
C1qtnf2	0.09	NA	0	0.37	0
C1qtnf3	1.02	0.56	1.76	NA	NA
C1qtnf4	NA	NA	0	1.17	4.73
C1qtnf5	0.29	NA	NA	NA	NA
C1qtnf6	1.05	NA	0	1	1.03
C1qtnf7	1.39	NA	0	1.53	0
C1qtnf9	NA	NA	0.83	0.46	NA
C1r	0.47	NA	NA	NA	NA
C1rl	0.27	NA	NA	1.36	NA
C1s	0.3	2.2	9.95	1.12	NA
C2	0.45	1.93	0.72	0	1.36
C2cd2	NA	0	0.85	2.4	0
C2cd2l	NA	0	0.54	3.6	0
C2cd3	NA	0	1.64	1	1.25
C2cd4a	NA	NA	NA	1	NA
C2cd4b	NA	NA	4.51	0.57	NA
C2cd4c	NA	NA	0.61	0.71	NA
C2cd5	NA	0.64	2.67	1.45	NA
C3	0.23	0	0	1.18	0
C3ar1	2.3	2.98	3.14	0	1.66
C4b	NA	1.88	3.58	NA	NA
C5ar1	NA	3.71	2.29	2.5	6.86

C5ar2	NA	NA	NA	1.31	NA
C6	1.38	0.68	1.3	0.55	1.81
C8a	1.76	2.61	0.58	5.94	3.1
C8b	0.47	2.47	2.01	0.49	0
C8g	NA	0.56	2.6	2.68	5.15
C9	0.49	2.44	2.56	1.97	2.73
Caap1	NA	1.93	2.58	0.49	NA
Cab39	0	1.3	1.7	2.52	0
Cab39l	0.19	1.24	0.87	1.72	0
Cabc1	0	NA	NA	NA	0
Cabin1	0.72	0	1.09	1.51	4.51
Cables1	0.43	NA	0.7	0	0.84
Cables2	0.13	NA	4.65	8.06	1.64
Cabp1	0.93	1.81	0.69	0.82	4.47
Cabp2	1.31	3.32	0.33	2.82	0
Cabp4	0.89	NA	1.83	NA	NA
Cabp5	0.34	0	0	0	1.54
Cabp7	0	NA	0	0.67	0.38
Cabs1	NA	NA	2.16	0.1	NA
Cabyr	0.18	0.94	0.67	0.2	0
Cacfd1	NA	0.79	1.02	0	NA
Cachd1	NA	NA	0	2.74	2.29
Cacna1a	0.38	1.14	1.1	2.82	0
Cacna1b	0	1.07	0.51	0.69	0
Cacna1c	1.25	0	0.98	1.05	1.07
Cacna1d	0.66	0.7	1.5	NA	1.26
Cacna1e	0.57	0	0.94	0.89	1.89
Cacna1f	0.92	2.05	2.86	2.8	2.4
Cacna1g	0	1.43	0.36	1.64	0
Cacna1h	0.07	0.03	0.96	2.59	3.58
Cacna1i	NA	NA	NA	2.99	NA
Cacna1s	1.16	1.86	1.74	0	0
Cacna2d1	0.53	1.5	0.64	1.42	1.94
Cacna2d2	0.11	0	0.86	0	1.27
Cacna2d3	0	0	7.74	0.45	0
Cacna2d4	NA	NA	0	0	1.65
Cacnb1	0.3	0.47	0.96	3.04	0
Cacnb2	0.37	0.46	1.06	1.28	0
Cacnb3	1.42	1.78	1.49	1.44	7.8
Cacnb4	0	1.34	0.5	3.35	0
Cacng1	8.54	1.2	2.03	0.73	0

Cacng2	1.57	1.77	0.02	1.4	0
Cacng3	4.73	1.92	4.78	1.23	4.34
Cacng4	0	0.81	1.05	1.02	4.45
Cacng5	0.33	2.03	0.27	0.76	2.27
Cacng6	1.33	NA	1.3	4.17	1.23
Cacng7	1.29	NA	1.09	1.28	1.79
Cacng8	1.46	NA	0.36	NA	0
Cactin	NA	1.17	1.31	NA	NA
Cacul1	NA	NA	0.9	0.76	NA
Cacybp	0.3	1.93	2.05	1.08	0
Cad	0	0	0.79	2.44	0
Cadm1	NA	0.47	1.38	NA	0.51
Cadm2	NA	NA	0.58	2.41	NA
Cadm3	NA	0.68	0.98	5.77	1.7
Cadm4	NA	1.58	2.21	1.31	2.56
Cadps	0.13	2.24	1.2	1.16	2.04
Cadps2	0	0.6	4.98	0.6	1.86
Cage1	NA	NA	0.98	0	NA
Calb1	0.78	0.43	0.28	0	0
Calb2	1	1.29	0	1.52	6.03
Calca	0	0.33	1.48	0.5	0
Calcb	8.89	1.85	0.64	0.62	1.08
Calcoco1	NA	0.01	0.05	0.11	0.76
Calcoco2	NA	4.66	2.06	0.65	4.42
Calcr	2.99	0.7	0.96	1.33	8.44
Calcrl	4.36	0.91	0	8.54	1.22
Cald1	0.63	0.53	0.78	0.58	0.98
Calhm1	NA	NA	NA	3.34	NA
Calhm2	NA	0.87	0.87	2.28	0
Calm1	0.92	2.03	1.22	3.03	1.66
Calm2	0	1.34	2.3	2.5	NA
Calm3	0.02	0.22	0.88	0	0.52
Calml3	0	0	0.11	1.08	7.78
Calml4	0	2.91	1.8	1.02	0
Caln1	0.09	NA	0.58	0.27	6.6
Calr	0	1.44	0.76	2.52	0.87
Calr3	1.28	NA	0.68	4.16	NA
Calu	0	0.8	1.93	0.42	1.05
Caly	NA	1.54	2.92	1.13	1.42
Camk1	0	0	3.2	0.42	0
Camk1d	0	0	2.17	0	0

Camk1g	1.04	0.11	5.88	4.68	2.83
Camk2a	1	0.38	0.45	2.53	1.23
Camk2b	2.19	0.77	1.46	1.04	2.22
Camk2d	0.02	NA	1.13	3.52	0
Camk2g	0	1.49	0.41	3.08	NA
Camk2n1	NA	0	2.08	0.82	2.99
Camk2n2	NA	NA	3.47	0.96	0.81
Camk4	0.17	0	0.86	0	1.89
Camkk1	0	NA	2.32	1.56	0.93
Camkk2	0.39	0.61	2.82	2.1	2.8
Camkmt	NA	0	0.71	0.31	NA
Camkv	NA	1.58	0.15	1.18	2.74
Camp	2.14	0.48	1.73	0.24	0
Camsap1	0.38	0	1.87	NA	1.1
Camsap1l1	NA	NA	NA	NA	1.32
Camsap2	NA	0.47	0.95	0.43	NA
Camsap3	NA	NA	0.81	1.23	NA
Camta1	1.8	0	0.01	0.11	2.78
Camta2	0	2.46	3.79	1.12	2.17
Cand1	NA	0.65	0.25	0.22	0
Cand2	NA	0	1.9	1.75	3.27
Cant1	0.62	1.28	0.61	0.74	2.5
Canx	0.16	1.48	2.27	0.23	0
Cap1	0	0	2.49	0.35	0
Cap2	4.13	0.65	0.53	3.92	0.14
Capg	0.94	0	2.77	1.21	0
Capn1	0.4	1.7	0.95	0	0.53
Capn10	1.25	1.27	1.71	2.43	1.21
Capn11	NA	1.46	1.83	0.15	0
Capn12	1.9	NA	1.94	2.41	1.92
Capn13	NA	NA	2.52	3.14	0
Capn2	0.41	0.98	1.32	0	0.28
Capn3	0.57	1.8	0.3	NA	0
Capn5	0.04	0	1.28	1.08	1.83
Capn6	0.72	0.48	0.71	1.6	1.38
Capn7	0.32	0.02	3.29	0	0
Capn8	NA	NA	0.64	NA	0
Capn9	0.59	0.11	1.29	0	NA
Capns1	0.03	0	1.68	0	2.64
Capns2	0.65	NA	2.1	1.06	0.24
Caprin1	NA	0	1.11	0.06	1.59

Caprin2	NA	0	0.07	0.07	3.34
Caps2	0	NA	2.66	2.25	0.04
Capsl	NA	NA	1.04	0.79	0.57
Capza1	1.63	0	1.19	1.01	0
Capza2	0.26	0	6.32	1.01	0
Capza3	1.88	NA	1.06	2.21	3.07
Capzb	0.2	1.22	0.56	0.09	0.1
Card10	0	0.82	0.54	0	7.29
Card11	0.94	NA	1.86	0.61	1.91
Card14	0.34	2.88	0.63	0	3.32
Card6	0.31	NA	8.43	0.58	0
Carf	NA	0.61	1.74	1.52	NA
Carhsp1	0.65	1.56	1.02	1.97	0.54
Carkd	NA	0.92	1.2	0.71	0
Carkl	6.14	NA	NA	NA	NA
Carm1	0.53	0.14	0.91	NA	0.32
Carns1	NA	NA	0.82	7.23	NA
Cars	0.44	2.38	0.01	0.92	4.05
Cars2	NA	2.71	0.29	2.47	0
Cart1	0.32	NA	NA	NA	NA
Cartpt	NA	0.97	1.14	2.39	1.88
Casc1	0.3	NA	NA	0.29	NA
Casc3	0.3	0.19	0.35	4.4	0.03
Casc4	NA	NA	0.07	0	0
Casc5	NA	0.76	0.42	0	0.63
Casd1	NA	0.48	0.92	4.4	0
Cask	0.59	1.34	1.66	2.44	1.27
Caskin1	1.4	NA	0	NA	1.46
Caskin2	1.35	0.73	3.03	1.1	1.42
Casp1	0.48	0	3.23	0	0
Casp12	0.8	NA	NA	3.28	NA
Casp14	0	NA	0.33	0.36	0
Casp2	0	3.53	1.97	0	0
Casp3	0.21	1.44	0.45	0.81	0
Casp4	0.35	0.15	2.1	1	0
Casp6	0	1.15	1.4	1.63	0
Casp7	0.51	0.45	2.06	0.42	0.62
Casp8	0.29	1.47	1.58	0.55	1.6
Casp8ap2	0.5	2.33	0.93	1.11	3.47
Casp9	0	1	1.28	0	2.34
Casq1	0.66	0.6	1.53	3.59	5.1

Casq2	2.99	1.15	0.92	0.81	3.36
Casr	NA	0.64	0.21	1.2	0
Cass4	NA	2.91	0.57	1.11	1.8
Cast	0.42	1.5	2.14	1.35	0.9
Casz1	NA	1.13	0.18	0.78	0
Cat	0	0.79	2.83	2.44	0
Catsper1	0.09	NA	1.29	0.93	NA
Catsper2	0	NA	0.17	0.96	NA
Catsper3	7.39	NA	0.66	0.7	0.65
Catsper4	0.21	NA	NA	0.88	NA
Catsperb	NA	NA	NA	0.4	NA
Catsperd	NA	NA	0.87	2.02	NA
Cav1	1.87	1.03	1.5	0.92	0.97
Cav2	0.45	0.17	1.02	1.11	0.88
Cav3	2.67	0.99	1.2	3.04	0
Cbara1	1.41	NA	NA	NA	1.86
Cbfa2t2	NA	0	0.68	0	0.31
Cbfa2t3	NA	0.01	0.65	1.52	0
Cbfb	0.1	0.97	1.33	0.52	0
Cbl	0.57	0.68	1.14	1.29	0.75
Cblb	0.71	0	2.48	5.57	1.49
Cblc	1.08	1.02	0.63	5.3	0
Cbll1	3.31	0	0.92	4.78	1.47
Cbln1	0	1.33	0	1.77	2.03
Cbln2	0	NA	1.33	1.1	1.55
Cbln3	0.93	NA	0.95	1.05	0
Cbln4	0.67	NA	1.41	0.67	0.84
Cbr1	0.64	2.61	4.86	0.45	0
Cbr3	0.81	3.31	0	2.99	0
Cbr4	NA	0	0.91	0.99	3.42
Cbs	0	2.19	0.18	0.61	1.66
Cbwd1	NA	NA	0.62	NA	7.22
Cbx1	0	0	0.66	0.86	0
Cbx2	0	2.31	1.58	1.52	1.39
Cbx3	0.42	1.66	0.41	0.06	0
Cbx4	0	0.2	1.46	1.75	1.44
Cbx5	0.9	0.39	1.02	0	0
Cbx6	NA	0.35	3.46	NA	0.17
Cbx7	0	0	0.77	1.86	2.17
Cbx8	0.1	2.27	1.4	2.22	0.83
Cby1	NA	0	3.42	0.13	2.95

Cby3	NA	NA	0	NA	0
Cc2d1a	NA	1.09	2.12	0.8	0.53
Cc2d1b	NA	NA	1.91	2.04	0
Cc2d2a	NA	NA	0.66	2.36	0
Cc2d2b	NA	1.72	1.48	0.91	1.6
Ccar1	0.21	NA	1.37	0.39	0
Ccbe1	NA	NA	0.33	4.2	0
Ccbl1	0	0	0.66	3.44	0.57
Ccbl2	NA	1.42	0.54	NA	0.18
Ccbp2	4.66	1.01	1.08	0.22	1.23
Ccdc101	NA	1.3	1.08	1.61	NA
Ccdc102a	NA	NA	1.03	0.02	0.91
Ccdc103	NA	NA	0	NA	3.56
Ccdc104	NA	NA	0.21	0.84	8.84
Ccdc105	NA	NA	0	0.89	NA
Ccdc106	NA	0.98	1.06	3.13	2.48
Ccdc107	NA	NA	2.53	0.22	0
Ccdc108	NA	NA	1.78	0.38	0
Ccdc109a	NA	NA	NA	NA	1.73
Ccdc109b	NA	2.55	3.3	2.35	0
Ccdc11	NA	NA	2.47	0.09	NA
Ccdc110	NA	NA	1.62	4.46	1.75
Ccdc111	NA	NA	2.16	0.72	4.31
Ccdc112	NA	NA	2.48	0.99	0
Ccdc113	NA	NA	2.09	0	0
Ccdc114	NA	NA	2.84	2.53	0
Ccdc115	NA	NA	0.52	2.28	0
Ccdc116	NA	NA	2.94	3.33	NA
Ccdc117	NA	NA	1.62	0.06	0
Ccdc12	NA	NA	1.76	1.87	4.27
Ccdc120	NA	NA	3.85	3.81	0.64
Ccdc121	NA	NA	NA	1.53	NA
Ccdc122	NA	NA	2.25	1.07	NA
Ccdc123	NA	NA	NA	NA	2.46
Ccdc124	NA	NA	0	0	0
Ccdc125	NA	NA	0	0.85	3.43
Ccdc126	NA	NA	3.44	1.91	2.36
Ccdc127	NA	NA	1.27	0.57	0
Ccdc129	NA	NA	NA	0.47	NA
Ccdc13	NA	NA	2.11	1.13	NA
Ccdc130	NA	0.1	1.59	0.43	3.03

Ccdc132	NA	2.32	0.74	0.44	0
Ccdc134	NA	1.5	0.42	1.39	2.02
Ccdc135	NA	NA	NA	0.99	NA
Ccdc136	NA	NA	1.1	0	0
Ccdc137	NA	NA	0.18	1.23	0
Ccdc138	NA	NA	0.97	0.62	1.03
Ccdc14	NA	NA	1.13	0.48	0
Ccdc141	NA	NA	0.46	0.75	NA
Ccdc142	NA	NA	1.55	2.14	NA
Ccdc146	NA	NA	0.1	4.42	0
Ccdc147	NA	NA	NA	4.84	NA
Ccdc148	NA	NA	NA	0.93	NA
Ccdc149	NA	NA	NA	0.73	NA
Ccdc15	NA	NA	NA	0.66	NA
Ccdc150	NA	NA	0.45	8.98	4.18
Ccdc151	NA	NA	0.95	0.78	1.71
Ccdc153	NA	NA	2.11	0	1.53
Ccdc154	NA	NA	NA	0	NA
Ccdc155	NA	NA	NA	2.21	NA
Ccdc157	NA	NA	0	3.09	1.97
Ccdc158	NA	NA	1.17	5.12	1.16
Ccdc159	NA	NA	1.91	2.74	NA
Ccdc160	NA	NA	6.73	NA	NA
Ccdc166	NA	NA	NA	1.53	NA
Ccdc167	NA	NA	0.67	0.42	NA
Ccdc169	NA	NA	1.31	0	NA
Ccdc17	NA	NA	0.88	NA	0
Ccdc170	NA	NA	NA	0.57	NA
Ccdc171	NA	NA	2.52	0.55	NA
Ccdc172	NA	NA	1.61	1.42	NA
Ccdc173	NA	NA	0.11	0.64	NA
Ccdc174	NA	NA	2.45	NA	NA
Ccdc175	NA	NA	NA	0	NA
Ccdc176	NA	2.71	0.83	0.45	NA
Ccdc177	NA	1.7	0.59	1.43	NA
Ccdc178	NA	NA	NA	2.81	NA
Ccdc18	NA	NA	1.09	0.34	0.76
Ccdc181	NA	2.44	1	0	NA
Ccdc19	NA	0.51	1.87	0	2.21
Ccdc21	NA	NA	NA	NA	0
Ccdc22	NA	0.3	1.64	NA	1.46

Ccdc23	NA	NA	2.74	1.03	0.31
Ccdc24	NA	NA	1.64	0.63	0.35
Ccdc25	NA	0	1.34	0.16	3.46
Ccdc27	NA	NA	NA	2.23	NA
Ccdc28a	NA	1.16	1.08	0	1.02
Ccdc28b	NA	0.65	0	0.54	0.7
Ccdc3	0.59	NA	1.78	3.1	2.49
Ccdc30	NA	1.69	0.51	13.89	0
Ccdc33	NA	1.59	0.94	1.66	0.27
Ccdc34	NA	NA	0.82	1.05	2.19
Ccdc36	NA	NA	NA	0	NA
Ccdc37	NA	NA	NA	0.89	NA
Ccdc38	NA	NA	1.66	2.05	NA
Ccdc39	NA	NA	1.5	NA	0.83
Ccdc40	NA	1.47	2.33	0	0
Ccdc41	NA	0.68	1.64	1.02	2.41
Ccdc42	NA	NA	NA	2.98	NA
Ccdc43	NA	NA	0	1.36	0
Ccdc45	NA	NA	NA	NA	2.71
Ccdc46	NA	NA	NA	NA	0
Ccdc47	NA	0.22	0.43	0.83	0
Ccdc48	NA	NA	NA	NA	0
Ccdc5	1.84	NA	NA	NA	NA
Ccdc50	NA	NA	0.7	0.31	0
Ccdc51	NA	1.91	0.27	0.79	2.1
Ccdc52	NA	NA	NA	NA	0
Ccdc53	NA	1.46	0.83	1.86	1.24
Ccdc54	NA	NA	0	0.05	3.89
Ccdc55	NA	NA	NA	NA	5.08
Ccdc56	NA	NA	NA	NA	0.09
Ccdc57	NA	1.61	3.14	1.1	36.21
Ccdc58	NA	NA	1.79	1.69	1.01
Ccdc59	NA	0.9	2.55	NA	1.44
Ccdc6	NA	0.58	1.51	1.9	2.64
Ccdc60	NA	NA	NA	1.49	NA
Ccdc61	NA	NA	3.59	2.15	0
Ccdc62	NA	NA	1.58	0	2.2
Ccdc63	NA	NA	0	0.48	NA
Ccdc64	NA	1.05	0.95	0.74	1.96
Ccdc64b	NA	NA	0	0.53	0
Ccdc65	NA	NA	0.84	1.05	NA

Ccdc66	NA	NA	0.32	1.58	0
Ccdc67	NA	NA	2.34	2.57	NA
Ccdc68	NA	0.85	0.24	0.86	0
Ccdc69	NA	1.25	0.54	0.6	0
Ccdc7	NA	NA	1.26	0	NA
Ccdc70	NA	1.68	0.71	9.95	0
Ccdc71	NA	2.65	2.26	1.5	0
Ccdc71l	NA	NA	0.53	1.79	NA
Ccdc72	NA	NA	NA	NA	1.59
Ccdc73	NA	NA	NA	0	NA
Ccdc75	NA	NA	NA	NA	0
Ccdc76	NA	NA	NA	NA	2.14
Ccdc77	NA	NA	1.13	0.97	1.09
Ccdc78	NA	NA	NA	1.84	NA
Ccdc79	NA	NA	0.69	0.94	NA
Ccdc8	NA	NA	NA	2.84	NA
Ccdc80	NA	NA	0.79	0.84	0
Ccdc81	NA	1.74	0.68	0	0
Ccdc82	NA	1.26	0.04	0.07	1
Ccdc83	NA	NA	0.56	0.57	1.45
Ccdc84	NA	NA	0.98	0	2.94
Ccdc85a	NA	NA	1.29	0.92	0
Ccdc85b	NA	1.67	2.45	2.26	0.83
Ccdc85c	NA	NA	NA	0.38	NA
Ccdc86	NA	2.22	6.15	1.15	1.23
Ccdc87	NA	1.63	1.56	0.75	11.55
Ccdc88a	NA	0.14	4.7	1.46	0.29
Ccdc88b	NA	NA	3.6	1.41	0.57
Ccdc88c	NA	2.42	1.07	0.61	2.31
Ccdc89	NA	NA	2.22	3.48	NA
Ccdc9	0	1.51	1.38	7.07	0
Ccdc90a	NA	NA	NA	NA	3.2
Ccdc90b	NA	3.24	1.57	1.3	0
Ccdc91	NA	0.53	0.32	0.58	0
Ccdc92	NA	0	1.53	2.19	2.71
Ccdc93	NA	1.24	1.54	0.73	2.11
Ccdc94	NA	0	1.45	0	1.55
Ccdc96	NA	NA	0.44	1.54	1.27
Ccdc97	NA	NA	1.33	1.92	0
Ccdc99	NA	NA	NA	NA	7.72
Ccer1	NA	NA	0.79	0	NA

Cchcr1	NA	0.09	0.84	0.39	3.43
Ccin	NA	2.46	1.39	0.98	2.98
Cck	0	0.96	2.41	2.43	2.31
Cckar	2.36	0.38	0.27	0.33	0
Cckbr	3.17	0.61	0.33	1.21	2.94
Ccl1	1	2.05	1.4	1.2	6.55
Ccl11	0.61	1.3	2.74	0.37	0
Ccl17	0.65	1.07	4.57	1.56	2.56
Ccl19	0	1.2	0.87	NA	4.98
Ccl2	0.77	2.75	1.08	4.67	4.92
Ccl20	0	0	5.43	2.13	3.91
Ccl22	0	0.52	0.41	0	2.8
Ccl24	1.44	2.02	0.87	1.26	6.07
Ccl25	0	0.73	0.02	0.82	0
Ccl26	NA	NA	NA	0.96	NA
Ccl27	3.21	NA	NA	NA	NA
Ccl28	0	NA	0.87	4.39	0.16
Ccl3	0.23	NA	NA	1.46	NA
Ccl4	0.09	2.03	2.14	1.33	0
Ccl5	0	0.63	4.37	0.29	0
Ccl7	0.69	1.27	0	3.04	0
Ccl8	1.15	1.07	3.37	1.47	0
Ccm2	0.67	NA	2.74	0.5	0
Ccm2l	NA	NA	0.73	1.98	NA
Ccna1	0	0	0.76	3.08	4.42
Ccna2	0.23	1.42	3.32	0.57	1.62
Ccnb1	0.24	0.76	1.67	0.44	0.75
Ccnb1ip1	NA	0	1.17	0.58	0
Ccnb2	0.58	1.67	2.45	0.87	0.77
Ccnb3	0	NA	0	3.46	2.51
Ccnc	0.41	0.46	0.51	0	1.49
Ccnd1	0.58	2.02	1.58	1.57	1.7
Ccnd2	0.81	1.53	3.21	2.11	1.26
Ccnd3	0	1.08	0.08	0.19	0
Ccndbp1	0.86	NA	2.24	1.08	1.61
Ccne1	0	0.93	1.08	1.01	3.11
Ccne2	0.03	0	1.81	NA	3.72
Ccnf	0	0.09	2.96	0.22	0
Ccng1	0	1.63	1.54	0.48	0
Ccng2	0.13	0.65	1.54	0.23	1.8
Ccnh	0	0	1.16	0.93	0

Ccni	0.9	0	1	0.17	0.67
Ccnj	0	2.05	2.73	1.48	1.15
Ccnjl	NA	0	1.06	2.45	0
Ccnk	2.88	1.49	3.08	NA	NA
Ccnl1	0.21	2.11	1.96	0.43	0
Ccnl2	0.86	0.88	1.09	0.72	2.36
Ccno	NA	0.4	1.39	3.91	0
Ccnt1	1.07	1.34	1.64	0.41	0.73
Ccnt2	0.13	1.35	1.08	0.13	0.15
Ccny	NA	NA	1.05	2.11	0
Ccnyl1	NA	NA	4.3	1.13	2.79
Ccp110	NA	2.2	2.46	0.5	NA
Ccpg1	NA	0.5	1.95	0.43	2.39
Ccr1	0	0.94	1.3	0.34	0
Ccr10	NA	0.21	1.2	1.26	3.1
Ccr2	0.45	0.6	2.57	1.46	1.12
Ccr3	1.53	0	0	0	3.51
Ccr4	19.9	0.94	1.49	2.52	2.42
Ccr5	NA	2.63	3.41	0.94	0
Ccr6	0.92	0	4.53	0.93	1.81
Ccr7	0	0	0.07	2.45	1.8
Ccr8	0.93	1.57	1.47	0.81	0
Ccr9	0.73	0	0	1.64	0.97
Ccrk	1.7	NA	NA	NA	NA
Ccrl1	0	1.45	3.05	1.46	0
Ccrl2	9.76	0.27	0.17	1.78	0
Ccrn4l	0.04	2.13	1.31	0	NA
Ccs	0.16	0.33	2.56	1.81	0
Ccsap	NA	NA	2.72	1.08	NA
Ccser1	NA	NA	0.82	0.47	NA
Ccser2	NA	0	1.24	5.05	NA
Cct2	0	1.01	0.5	0.39	0
Cct3	0.67	0.77	0.21	0.88	1.36
Cct4	0.07	1.03	1.3	0.58	0
Cct5	0	1.98	2.22	1.56	0
Cct6a	0	1.99	1.16	1.19	2.97
Cct6b	0	0	1.73	1.89	3.73
Cct7	0	0.42	0.41	0.36	0
Cct8	0.33	0.52	0.48	0	0.77
Ccz1	NA	2.22	0.83	NA	NA
Cd101	NA	NA	NA	0	NA

Cd109	0.61	NA	2.39	0.19	0.83
Cd14	0.66	1.4	0.86	3.08	0
Cd151	0	1.72	1.74	0.62	0.67
Cd160	1.49	0	0.9	1.17	0
Cd163	0.66	0	6.28	0	2.68
Cd163l1	NA	NA	0.51	1.21	1.08
Cd164	1.38	1.37	1.63	0.36	0.58
Cd164l2	NA	NA	NA	0.81	NA
Cd177	NA	0.03	3.44	0.33	0
Cd180	NA	1.11	1.63	0	0
Cd19	0.47	0.1	0.08	1.85	0
Cd2	0.42	0	1.93	5.58	0
Cd200	0	0	0.53	1.45	2.41
Cd200r1	0	NA	0.65	NA	NA
Cd200r2	0.91	NA	NA	NA	NA
Cd207	0.05	1.87	0	2.75	4.61
Cd22	6.73	0.05	1.13	2.03	1.36
Cd226	1.49	0.74	2.18	0.18	0
Cd244	1.42	1.82	2.4	NA	0.12
Cd247	NA	0	0	7.74	2.78
Cd248	NA	1.79	2.12	0	2.53
Cd27	NA	0	0.11	1.21	0
Cd274	NA	NA	4.2	0	0.17
Cd276	NA	NA	0.72	0.85	0
Cd28	0.14	0	1.47	0.66	0
Cd2ap	0	1.3	0.88	1.24	0.57
Cd2bp2	0.34	0.31	0.07	0.89	0
Cd300a	NA	0.38	1.34	1.35	0
Cd300c	NA	NA	NA	0.5	NA
Cd300e	NA	NA	NA	0.91	NA
Cd300lb	NA	NA	1.95	0.58	NA
Cd300ld	NA	NA	NA	3.85	NA
Cd300lf	NA	NA	1.79	2.28	NA
Cd300lg	NA	NA	0.95	1.82	NA
Cd302	NA	2.54	2.65	NA	3.63
Cd320	NA	0	5.71	0.52	0.91
Cd33	1.67	1.86	1.13	0.4	16.05
Cd34	0.18	1.73	1.53	1.87	1.2
Cd36	0.18	2.57	5.65	1.83	0.28
Cd37	0.06	0	0.95	0	0
Cd38	0	1.54	1.76	3.16	0.22

Cd3d	0	0	3.32	0	2.45
Cd3e	1.4	0	0	3.19	0
Cd3eap	NA	4.04	2	1.78	2.09
Cd3g	1.1	0	2.26	0.57	0
Cd4	0	0	1.28	3.15	0
Cd40	NA	1.1	0.45	0.32	0
Cd40lg	NA	0	1.91	2.13	5.11
Cd44	0	0.01	2.09	1.45	0
Cd46	NA	1.77	1.96	0.13	3.93
Cd47	0	0.92	2.89	1.14	1.63
Cd48	0.22	2.95	1.55	1.2	2.17
Cd5	0.85	0	2.19	0	0.02
Cd52	0.83	2.77	0.49	0.21	4.51
Cd53	0.18	0	0.76	9.99	0
Cd55	NA	3.13	0.02	1.63	3.89
Cd5l	2.13	3.32	3.36	4.13	2.34
Cd6	0	0	0.44	0	3.35
Cd63	0.41	NA	NA	9.2	NA
Cd68	0.97	3.81	1.71	0.02	0.31
Cd69	0	0.81	1.06	3.1	0.02
Cd7	0.81	0	0.16	1.75	0
Cd70	NA	0	0.07	0.76	0
Cd72	0.98	0.07	0.31	3.47	0
Cd74	NA	0	1.26	2.01	0
Cd79a	0.33	0	1.69	2.21	NA
Cd79b	2.29	0.05	4.66	0.36	0
Cd80	2.59	1.56	1.5	0.47	0
Cd81	0.66	0	0.69	0	0
Cd82	NA	1.25	0.96	0.86	0
Cd83	0.09	1.91	1.63	0.91	0
Cd84	0.66	0.46	1.05	6.59	1.01
Cd86	0.55	1.03	1.63	1.52	3.63
Cd8a	3.37	0	0.22	0.21	2.01
Cd8b	1.33	NA	NA	NA	NA
Cd9	0.31	0.12	0.93	1.4	0.76
Cd93	NA	0.93	2.17	1.57	0
Cd96	1.24	0	1.08	1.96	1.83
Cd97	0.05	2.23	6.93	0.83	1.73
Cd99	NA	2.59	3.33	0.82	3.3
Cd99l2	NA	NA	1.62	0.77	2.53
Cda	0	0	0	0.12	0

Cdadc1	0.37	1.35	0.97	0.58	0
Cdan1	0.89	NA	0.52	2.6	NA
Cdc123	NA	0.18	1.84	0	0.07
Cdc14a	0.63	1.3	0.09	0	1.25
Cdc14b	0.59	0	1.31	0.74	1.91
Cdc16	0	0	0.43	1.53	4.21
Cdc20	0	0.72	4.63	0	1.58
Cdc20b	NA	NA	1.97	NA	3.05
Cdc23	0	0	0.83	0.94	1.14
Cdc25a	0	0	1.52	1.28	0
Cdc25b	1.13	0	3.38	0	0
Cdc25c	0.34	1.28	2.5	4.26	0
Cdc26	0.5	NA	0.82	0.79	1.7
Cdc27	0.36	0.67	0.83	0.81	0.52
Cdc2l2	0.15	NA	NA	NA	NA
Cdc2l5	0.49	NA	NA	NA	NA
Cdc34	0	0.96	2.13	4.09	NA
Cdc37	0.47	0.57	0.71	NA	0.3
Cdc37l1	NA	1.23	1.36	0.24	1.44
Cdc40	0	0.55	0.39	0.34	3.2
Cdc42	0.51	0.37	1.14	1.37	0
Cdc42bpa	1.17	1.28	0.22	1.57	0
Cdc42bpb	2.15	2.89	1.01	0	0
Cdc42bpg	NA	NA	2.74	0.53	NA
Cdc42ep1	0	1.26	2.95	1.82	0
Cdc42ep2	0	3.43	3.06	3.28	0
Cdc42ep3	0.97	0.52	0.28	0	9.08
Cdc42ep4	0.52	0	1.24	0.69	0
Cdc42ep5	1.45	NA	0.43	1.44	0
Cdc42se1	NA	0.97	0.93	1.98	2.65
Cdc42se2	NA	NA	2.55	0.96	3.35
Cdc45	NA	0	2.34	0.46	NA
Cdc45l	0	NA	NA	NA	NA
Cdc5l	0.34	0.75	0.69	0.4	0
Cdc6	0	0.26	3.26	2.9	1.82
Cdc7	0	0.54	0.84	0.46	0.12
Cdc73	NA	0.63	0.81	0	0
Cdca2	2.22	NA	3.38	0.64	0
Cdca3	0.37	0	2.22	0.46	2.74
Cdca4	0.45	0.03	1.72	1.66	0
Cdca5	0.41	NA	1.84	0.5	0

Cdca7	0.55	NA	2.3	1.67	0.98
Cdca7l	NA	NA	1.7	0	0
Cdca8	0	0.9	5.4	0.75	0.03
Cdcp1	NA	2.28	2.13	0.38	2.11
Cdcp2	NA	NA	NA	3.21	NA
Cdgap	0	NA	NA	NA	NA
Cdh1	0.59	0.81	2.57	1.84	1.57
Cdh10	NA	0.82	0.54	0	1.4
Cdh11	1.65	0.51	2.05	0.31	1.68
Cdh12	NA	NA	NA	3.28	NA
Cdh13	1.21	1.33	0.29	2.27	0.92
Cdh15	1.74	0.44	2.31	1.06	0
Cdh16	1.55	2.28	0.98	1.85	4.68
Cdh17	0	1.91	3.67	2.23	0
Cdh18	0.78	2.02	0.5	2.37	1.7
Cdh19	NA	NA	NA	3.33	NA
Cdh2	0.53	0.49	2.07	0	1.61
Cdh20	0.57	0	2.98	4.25	3.26
Cdh22	1.54	2.19	1.64	2.18	NA
Cdh23	0.04	NA	0	1.6	0
Cdh24	0.71	NA	NA	0.53	NA
Cdh26	NA	NA	3.11	0.64	5.14
Cdh3	0.41	0.65	0.14	0.44	2
Cdh4	0	0.8	0.43	2.37	0
Cdh5	0.86	0.72	0.63	0	1.2
Cdh6	1.59	1.07	0.91	2.96	4.48
Cdh7	0.21	1.77	0.98	1.12	2.97
Cdh8	0.13	0.54	0.56	0.58	1.67
Cdh9	2.84	1.6	0	1.39	0
Cdhr1	NA	1.2	0	0.96	NA
Cdhr2	NA	1.66	2.36	2.09	NA
Cdhr3	NA	NA	0	2.08	NA
Cdhr5	NA	1.49	1.21	3.6	NA
Cdip1	NA	0	0.92	1.44	NA
Cdipt	0.61	0	2.36	1.24	0
Cdk1	NA	0.72	2.36	0.75	NA
Cdk10	0.15	0	0.54	1.43	3.25
Cdk11b	NA	NA	NA	0.9	NA
Cdk12	NA	0.43	0.74	3.67	NA
Cdk13	NA	0.22	1.22	0	NA
Cdk14	NA	0	0.06	6.88	NA

Cdk15	NA	NA	NA	1.83	NA
Cdk16	NA	0.99	1.81	0.67	NA
Cdk17	NA	1.38	1.25	0.39	NA
Cdk18	NA	1.67	1.16	0.99	NA
Cdk19	NA	1.72	1.69	0.68	NA
Cdk2	1.28	0	1.71	0.72	0
Cdk20	NA	0.89	1.12	5.32	NA
Cdk2ap1	0.3	1.53	2.19	0	45.32
Cdk2ap2	NA	1.55	0.65	0	4.57
Cdk3	0.37	NA	NA	NA	0
Cdk4	0.27	2.46	1.03	0.41	0
Cdk5	0	2.04	0	1.32	2.39
Cdk5r1	0.53	0.5	0.57	0	0.15
Cdk5r2	0	2.5	0.16	1.23	0
Cdk5rap1	0.65	0	2.85	0	8.09
Cdk5rap2	0.71	2.16	0.39	0	0
Cdk5rap3	0.7	0	1.35	0.35	3.95
Cdk6	0.15	0.65	0.86	3.89	0.96
Cdk7	0	1.49	1.73	1.55	0
Cdk8	0.04	0.55	1.92	1.15	0
Cdk9	1.12	1.25	0.78	1.78	0.22
Cdkal1	0.87	0	1.16	0.25	0.05
Cdkl1	1.11	2.44	0.31	2.64	2.46
Cdkl2	0.59	0.97	0.34	1.77	0
Cdkl3	1.06	0.85	0.51	0.48	0
Cdkl4	NA	NA	NA	0.53	NA
Cdkl5	0.48	NA	NA	0	NA
Cdkn1a	0.37	2.7	0.78	4.61	0
Cdkn1b	1.04	0.07	2.55	1.61	0
Cdkn1c	0.27	0.96	1.45	3.1	0
Cdkn2a	0.44	1.91	3.16	2.21	3.04
Cdkn2aip	NA	0.31	1.97	0	0
Cdkn2aipnl	NA	NA	1.21	0.94	0
Cdkn2b	0.51	0.78	1.52	1.41	0.74
Cdkn2c	0.11	2.5	0.93	4.14	0
Cdkn2d	0.1	NA	NA	1.46	NA
Cdkn3	0	0.11	3.34	2.81	2.2
Cdnf	NA	NA	NA	1.6	NA
Cdo1	2.35	1.64	1.09	0.24	4.08
Cdon	0.67	0.08	0.46	0.96	1.74
Cdpf1	NA	NA	0	2.13	NA

Cdr2	3.67	0	0.49	0.74	0
Cdr2l	NA	1.65	0.59	1.17	1.83
Cdrt4	NA	NA	1.33	1.21	NA
Cds1	1.21	1.85	1.96	3.38	0
Cds2	2.06	2.23	2.62	21.32	1.15
Cdsn	NA	0.14	0.17	1.15	0
Cdt1	NA	1.78	1.52	1.35	0
Cdv3	0.19	0.31	1.25	3.17	0
Cdx1	0	0.43	2.06	0.65	0
Cdx2	0.07	1.26	1.74	0.49	0
Cdx4	2	2.42	2.7	1.82	0
Cdyl	0.24	0.09	1.48	0	0
Cdyl2	0.33	NA	1.49	0.07	NA
Ceacam1	0.65	0	0.29	1.13	0
Ceacam16	NA	NA	NA	2.65	NA
Ceacam19	NA	NA	NA	1.4	NA
Ceacam20	NA	NA	NA	0.6	NA
Ceacam5	NA	NA	NA	3.58	NA
Cebpa	0	1.55	0.23	1.2	0.22
Cebpb	0.33	0.3	2.53	0	0
Cebpd	0.17	0.38	0.96	5.39	0
Cebpe	0.18	NA	NA	1.14	NA
Cebpg	0	0.98	2.22	0.81	1.84
Cebpz	0.72	0.42	1.14	3.5	1.91
Cecr2	0.83	NA	0.13	4.24	1.11
Cecr5	0.17	0	2.5	1.66	0
Cecr6	0.15	NA	0.14	1	0.94
Ceecam1	0.37	NA	NA	NA	NA
Cel	2.01	1.99	1.9	0.15	2.07
Cela1	NA	NA	2.1	1.33	0.59
Cela2a	NA	0	1.13	0.56	3.08
Cela3b	NA	1.15	0.99	0.24	0
Celf1	NA	1.58	0.29	1.59	NA
Celf2	NA	0.31	0.61	1.06	NA
Celf3	NA	0	0.42	0	NA
Celf4	NA	NA	0.27	2.5	NA
Celf5	NA	NA	0.87	1.95	NA
Celf6	NA	NA	1.91	2.14	NA
Celsr1	0.07	0	2.15	0	0
Celsr2	2.12	0	2	1	0.98
Celsr3	0.44	2.48	0.74	1.41	0

Cend1	NA	0.61	0.78	0.84	0
Cenpa	0.31	0.87	5.59	1.67	1.53
Cenpb	0	1.23	0.69	1.39	4.38
Cenpc1	0.78	0	1	0.5	4.65
Cenpe	0	0.09	2.98	0.58	1.23
Cenpf	0.08	0.81	1.24	0	2.45
Cenph	0.11	NA	2.41	1.15	4.55
Cenpi	NA	0.31	3.87	1.39	2.87
Cenpj	0	0.34	0.83	0	0
Cenpk	NA	NA	0.35	NA	0
Cenpl	NA	NA	2.97	0	4.56
Cenpm	NA	1.01	2.79	5.87	1.29
Cenpn	NA	1.39	3.65	NA	0.07
Cenpo	NA	1.63	0.94	NA	0.65
Cenpp	NA	NA	NA	0.66	NA
Cenpq	NA	2.18	2.04	0.72	0.09
Cenpt	NA	1.39	2.72	0.16	0
Cenpv	NA	NA	1.6	0	0
Cenpw	NA	NA	2.91	0	NA
Centa1	0.43	NA	NA	NA	NA
Centa2	0	NA	NA	NA	NA
Centb1	0.8	NA	NA	NA	NA
Centb2	0.85	NA	NA	NA	NA
Centb5	0.51	NA	NA	NA	NA
Centd1	0.48	NA	NA	NA	NA
Centd2	0.46	NA	NA	NA	NA
Centg2	1.35	NA	NA	NA	NA
Centg3	0.01	NA	NA	NA	NA
Cep104	NA	0	1.75	1.49	NA
Cep110	NA	NA	NA	NA	1.74
Cep112	NA	0.17	0.07	1.94	NA
Cep120	NA	NA	1.95	0.65	0
Cep128	NA	NA	1.46	0	NA
Cep135	NA	2.08	0.47	0.27	0
Cep152	NA	2.76	1.95	0.84	0.63
Cep164	NA	0.24	2.42	0.09	0.85
Cep170	NA	1.21	1.03	0.7	0
Cep170b	NA	1.59	0	1.11	NA
Cep19	NA	NA	1.98	0.2	NA
Cep192	NA	0	0.89	0	1.69
Cep250	NA	0	3.01	0	0

Cep290	NA	0.67	1.21	NA	0
Cep350	NA	0.5	0.66	0.26	0.58
Cep41	NA	1.12	0.45	0.61	NA
Cep44	NA	NA	0.74	0.63	NA
Cep55	NA	0.58	1.96	0.84	1.53
Cep57	NA	0.12	2.18	0.91	3.35
Cep57l1	NA	NA	0.17	0.82	NA
Cep63	NA	0.13	0.59	0.1	0
Cep68	NA	0	0.56	1.09	0.77
Cep70	NA	0.28	1.38	1.37	0
Cep72	NA	0	2.51	0.05	1.25
Cep76	NA	1.01	0.56	5.25	0
Cep78	NA	NA	1.39	0.5	2.38
Cep85	NA	1.88	3.1	0	NA
Cep89	NA	NA	1.51	0.88	NA
Cep95	NA	NA	0.71	0.16	NA
Cep97	NA	2.95	0.73	0.93	0.7
Cept1	0.69	0	1.43	1.07	1.64
Cer1	0.15	0.31	0.69	0	0
Cercam	NA	NA	1.47	0.83	2.61
Cerk	0.39	0.34	0.95	0	NA
Cerkl	NA	NA	NA	0	NA
Cers1	NA	1.77	1.87	0	NA
Cers2	NA	3.15	2.76	1.45	NA
Cers3	NA	NA	0.8	0.62	NA
Cers4	NA	2.03	1.05	0.64	NA
Cers5	NA	NA	1.98	1.28	NA
Cers6	NA	1.76	1.31	1.01	NA
Ces1	0	NA	NA	NA	0
Ces2	3.12	NA	NA	NA	NA
Ces3	2.11	NA	NA	NA	1.59
Ces4a	NA	NA	0.93	0.94	NA
Ces5a	NA	NA	1.13	1.24	NA
Ces8	NA	NA	NA	NA	2.28
Cetn1	1.04	0	0.21	3.11	3.87
Cetn2	0.16	2.42	4.6	0.98	0
Cetn3	0.86	0.57	1.41	2.29	0
Cfb	NA	0.13	0.28	0.81	2.25
Cfc1	NA	NA	0.14	NA	2.44
Cfd	NA	1.08	1.38	2.49	7.09
Cfdp1	0.25	1.33	2.26	NA	3.4

Cfh	0	1.28	3.33	2.01	NA
Cfhr1	NA	1.28	2.89	2.67	NA
Cfhr2	NA	0.09	0.69	1.02	NA
Cfhr3	NA	NA	5.8	NA	NA
Cfi	1.41	2.05	0.71	0.52	NA
Cfl1	0.78	1.42	0.28	0	0.08
Cfl2	0.13	NA	0.65	0.8	1.45
Cflar	0	0.01	0.63	1.03	0
Cfp	NA	1.92	0.78	2.58	0
Cftr	0.45	0	0.74	0.52	1.04
Cga	1.13	0.46	1.96	1.66	3.47
Cggbp1	1.54	0	0.94	0.99	0.83
Cgn	0.3	NA	3.9	2.96	1.61
Cgnl1	NA	NA	0.36	3.94	0.08
Cgref1	0	0	1.06	1.74	0
Cgrrf1	NA	1.35	0.29	0.44	0
Ch25h	0.16	0.86	3.39	3.92	0
Chac1	NA	1.09	1.24	0.1	7.18
Chac2	NA	NA	0.25	0.51	3.9
Chad	1.09	2.22	1.7	0.16	0.09
Chadl	NA	NA	1.13	1.13	NA
Chaf1a	0.19	NA	NA	1.2	NA
Chaf1b	0.36	1.61	0.75	0.85	0.75
Champ1	NA	NA	0.52	5.14	NA
Chat	0.32	2.22	4.14	0.26	0
Chchd1	0.13	NA	0.35	0.56	0
Chchd10	NA	NA	0.95	0	0
Chchd2	NA	0	2.96	0.13	NA
Chchd3	0	0.15	1.08	0.2	0
Chchd4	0.03	NA	0.72	1.12	0
Chchd5	0.28	NA	0.81	0	0.53
Chchd6	NA	NA	1.62	0.15	0.99
Chchd7	0.73	1.22	0.77	1.12	1.09
Chchd8	NA	NA	NA	NA	0
Chd1	0	0	1.02	0.49	0
Chd11	0.43	0	2.28	0.66	0
Chd2	1.53	1.44	1.03	1.94	1.39
Chd3	0.52	0	2.71	1.24	0.77
Chd4	0.46	1.7	1.6	3.63	0.04
Chd5	0.08	0	1.37	0.03	0
Chd6	NA	NA	1.43	0	1.41

Chd7	2.1	0.71	2.18	NA	0
Chd8	1	0	1.63	0.43	0
Chd9	0	1.16	2.98	1.09	0
Chdh	0.29	NA	0.67	0.07	1.19
Chek1	0	0.25	1.03	0.38	0
Chek2	0.95	1.64	2.64	0.98	3.02
Cherp	2.64	0	2.15	NA	0
Ches1	0.49	NA	NA	NA	NA
Chfr	0.91	0.8	1.01	0	2.04
Chga	0.69	2.88	9.1	0	7.05
Chgb	2.29	2.08	0.29	1.17	0
Chi3l1	7.93	2.43	3.44	0.83	0.77
Chia	NA	0.36	0.76	6.07	5.11
Chic1	0	NA	0.1	0	0
Chic2	0.35	1.54	2.29	0	0
Chid1	NA	NA	1.7	0.09	0.79
Chit1	5.16	1.39	0	1.09	0
Chka	0.35	0.65	0.19	1.98	1.54
Chkb	1.37	0.24	4.98	NA	1.23
Chl1	0.65	0.61	0.88	2.12	0
Chm	0.44	1.05	0.8	0	1.35
Chml	0.25	0.78	0.75	0.53	2.69
Chmp1a	NA	3.36	1.14	1.28	0
Chmp1b	NA	0.71	1.02	0	4.66
Chmp2a	NA	0.45	2.71	2.72	0
Chmp2b	NA	0.31	0.07	0.51	0
Chmp3	NA	1.65	0.63	0.76	NA
Chmp4b	NA	NA	2.33	1.47	NA
Chmp4c	NA	NA	0.78	5.46	0
Chmp5	NA	1.29	1.27	1	1.24
Chmp6	NA	0.89	1.95	0	0.96
Chmp7	NA	0	0.35	0	6.14
Chn1	0.21	0.12	0	0	0.27
Chn2	0.42	0	1.4	NA	0
Chodl	0.22	1.91	3.57	1.08	0
Chordc1	0.4	2.64	1.1	0.95	3.46
Chp1	NA	1.77	1.23	1.89	NA
Chp2	NA	0.71	2.51	1.76	NA
Chpf	NA	1.34	0.87	1.53	0
Chpf2	NA	1.03	0.18	NA	1.04
Chpt1	2.31	1.85	1.79	0.73	0.82

Chrac1	6.64	NA	2.34	1.24	0
Chrd	9.53	2.5	1.02	NA	0
Chrdl1	0.36	0.02	0.66	0	1.64
Chrdl2	1.33	NA	1.25	1.71	0
Chrm1	0.25	NA	1.22	1.22	0
Chrm2	3	NA	NA	4.24	NA
Chrm3	0.63	1.97	0.82	0.52	0
Chrm4	0	0.92	1.59	1.35	0.12
Chrm5	2.29	NA	NA	2.33	NA
Chrna1	0.52	1.42	0.42	1.29	0
Chrna10	NA	NA	NA	1.03	NA
Chrna2	0.05	1.81	1.14	1.56	1.69
Chrna3	0.03	0.51	0.64	1.38	1.74
Chrna4	0	0.79	0.32	2.31	3.31
Chrna5	1.22	0.67	0.83	4.3	0
Chrna6	1.12	1.26	0	1.13	0
Chrna7	0.28	NA	NA	3.43	NA
Chrna9	0	0.96	0.62	2.64	1.81
Chrnb1	1.81	1.79	5.45	NA	4.08
Chrnb2	0.76	1.65	0.24	1.37	0
Chrnb3	1	1.64	0.75	0	2.67
Chrnb4	1.18	0.16	0.04	0.8	1.3
Chrnd	2.03	1.49	4.41	1.93	1.11
Chrne	1.04	3.28	0.88	NA	0
Chrng	0	1.43	2.39	0.7	1.98
Chst1	1.68	1.76	1.63	0.8	4.27
Chst10	1.42	0.15	1.35	0	1.95
Chst11	0.78	3.2	1.55	11.45	0
Chst12	1.07	0	2.13	5.82	2.05
Chst13	NA	NA	1	0.78	1.82
Chst14	NA	NA	0.79	4.55	0
Chst15	NA	1.41	1.44	0	0
Chst2	1.21	0.59	1.09	0.78	1.54
Chst3	0.8	0.58	2.32	1.37	0
Chst4	1.36	1.92	3.3	1.34	1.52
Chst5	0.72	2.46	1.81	0.85	0
Chst7	0.72	0	0.23	1.53	4.49
Chst8	2.3	0	0.78	0.57	9.06
Chst9	1.17	NA	1.81	4.35	0
Chsy1	NA	0	1.47	3.24	NA
Chsy3	NA	NA	0.86	0.8	0

Chtf18	1.02	NA	2.6	0.57	0
Chtf8	NA	NA	0.28	1.33	0
Chtop	NA	0.84	1.88	1.26	NA
Chuk	0.57	2.64	1.25	1.5	0
Churc1	0.46	NA	0.01	1.78	0.81
Chx10	1.87	NA	NA	NA	NA
Ciao1	NA	1.18	0.78	0	0
Ciapin1	0.29	1.42	2.46	0.72	2.44
Cib1	0.58	1.23	2.08	NA	0
Cib2	0.29	0.26	0	NA	1.88
Cib3	0.76	NA	2.99	7.1	NA
Cib4	NA	NA	0.53	0.47	0
Cic	1.18	0	1.45	0	0.49
Cidea	0.17	1.7	0.06	0.89	8.12
Cideb	NA	1.97	0.42	NA	0
Cidec	2.88	1.47	1	3.34	6.09
Ciita	NA	1.81	0.03	0.26	0.49
Cilp	0.77	1.02	0.58	1.87	0
Cilp2	2.8	NA	0.94	0.78	NA
Cinp	NA	2.31	0.48	0.05	0.82
Cir1	NA	3.64	1.66	1.21	0
Cirbp	0.3	0	2.22	0	4.26
Cirh1a	NA	NA	1.43	1.16	3.12
Cisd1	NA	2.48	0.98	0.18	0
Cisd2	NA	NA	1.54	0	2.96
Cisd3	NA	NA	4.01	NA	1.08
Cish	0.58	3.25	0.24	2.71	0
Cit	0.87	1.01	1.4	0	1.63
Cited1	1.52	1.52	1.86	1.63	0
Cited2	0.8	2	0.39	0	3.09
Cited4	0.39	NA	0.95	0.62	1.38
Ciz1	0	0	1.02	1.11	0
Ckap2	0.02	0.71	3.26	0.01	1.84
Ckap2l	NA	NA	1.9	0	0
Ckap4	0	0.15	2.72	0.96	0
Ckap5	NA	0	1.46	0	1.86
Ckb	1.52	1.25	5.37	1.69	1.49
Cklf	NA	0	2.32	NA	0.14
Ckm	0	0.46	0	1.59	1.67
Ckmt2	2.98	1.13	1.29	0.05	2.53
Cks1b	NA	0.22	2.14	0.75	0.56

Cks2	0	0.03	4.17	0.57	5.05
Clasp1	0.68	0.81	0.92	0	0.59
Clasp2	0.84	0.03	1.02	0	0
Clasrp	NA	1.99	2.86	6.72	NA
Clca1	1.46	1.29	0.3	1.38	0
Clca2	30.48	0.92	0.07	0.64	0
Clca3	0.91	NA	NA	NA	NA
Clca4	0.61	3.18	0	0.83	1.1
Clcc1	NA	0.59	1.22	0.97	2.79
Clcf1	NA	1.03	0.69	1.16	0
Clcn1	4.28	0	0.66	0.16	1.24
Clcn2	0	0.95	1.3	1.11	1.78
Clcn3	0.15	2.28	2.69	0.14	0
Clcn5	1.39	0	0.56	3.88	0.46
Clcn6	0	0.03	0.8	0	1.88
Clcn7	1.77	0.68	1.79	2.61	0.81
Clcnka	1.16	NA	NA	0.58	NA
Clcnkb	0	0.6	1.53	0.99	6.23
Cldn1	0	0	1.52	0.74	0
Cldn10	0.23	1.31	0.81	NA	NA
Cldn11	1.06	0.75	1.63	0.63	0
Cldn12	5.63	NA	2.84	2.8	1.1
Cldn14	0	0.73	5.08	2.36	0
Cldn15	0.78	1.76	0.62	2.2	0
Cldn16	2.06	0.32	0.55	1.89	1.84
Cldn17	1.94	NA	NA	1.52	NA
Cldn18	0	0.57	0.46	1.59	3.29
Cldn19	0.18	NA	0	1.16	NA
Cldn2	0.81	NA	0.77	NA	4.15
Cldn20	NA	NA	NA	0	NA
Cldn22	3.88	NA	NA	NA	NA
Cldn23	0	NA	1.39	1.75	1.75
Cldn24	NA	NA	NA	0	NA
Cldn3	1.5	1.85	0.82	1.98	0
Cldn4	0.56	2.66	3.53	0.8	0.86
Cldn5	0	1.72	2.24	1.03	3.54
Cldn6	0	0	0.8	4.38	1.77
Cldn7	0.85	0.89	0	0.71	2.61
Cldn8	0.47	1.75	0	1.82	0
Cldn9	0	0.66	1.68	1.96	5.05
Cldnd1	NA	NA	NA	NA	0

Cldnd2	NA	NA	0.26	1.96	0
Clec10a	NA	2.7	0.97	2.54	0.91
Clec11a	NA	0.11	0.36	1.38	0
Clec12a	NA	NA	NA	0	NA
Clec12b	NA	NA	0.56	0.19	0
Clec14a	NA	NA	1.63	0.69	0
Clec16a	NA	0	0.77	0	1.47
Clec18a	NA	NA	2.12	NA	0
Clec1a	NA	0	1.25	0	0
Clec1b	NA	1.58	1.56	2.74	0
Clec2d	NA	2.76	4.86	0	0
Clec2l	NA	NA	1.09	NA	0
Clec3a	NA	NA	NA	0.19	NA
Clec3b	NA	1.83	1.19	5.06	2.11
Clec4d	NA	NA	1.17	3.43	NA
Clec4e	NA	0.38	0.33	1.18	0
Clec4f	NA	NA	1.64	1.09	NA
Clec4g	NA	NA	0	0.78	NA
Clec5a	NA	0.21	0.7	1.61	0
Clec7a	NA	2.53	1.72	0.54	0
Clec9a	NA	NA	NA	2.95	NA
Clgn	3.38	2.32	0	0	0
Clhc1	NA	NA	3.9	1.35	NA
Clic1	0.76	0	1.66	0.17	0
Clic3	1.84	1.67	2.04	2.53	24.9
Clic4	0	2.34	5.1	0.8	0
Clic5	1.52	0.53	0.76	1.98	0.1
Clic6	0.09	NA	1.12	0.56	2.23
Clint1	NA	0.9	1.46	1.04	1.78
Clip1	NA	0.27	0.19	0	0.41
Clip2	NA	0	0	1.12	0
Clip3	NA	0.95	0.58	0.8	3.59
Clip4	NA	0.83	0.4	0	0
Clk1	0	NA	NA	1.17	NA
Clk2	0.84	0	1.2	2.01	5.42
Clk3	1.64	1.08	1.69	NA	0
Clk4	0.19	3.12	1.25	0	0.16
Clmn	0.3	1.01	0.87	0.12	0
Clmp	NA	NA	2.27	0.45	NA
Cln3	1.32	2.54	1.6	6.07	0
Cln5	0.75	0.27	1.3	1.52	5.06

Cln6	0	0.49	2.69	0	3.26
Cln8	0.32	1.13	2.73	2.18	0.15
Clnk	NA	NA	2.29	1.41	NA
Clns1a	0.73	1.14	0.24	1.81	0
Clock	0	0.14	0.02	0.51	1.45
Clp1	0.54	0.17	0	0.69	0.11
Clpb	NA	1.43	1.76	0.11	1.94
Clpp	0.09	0.43	1.58	0.15	4.57
Clps	0.43	0.89	0.32	0.78	3.9
Clpsl2	NA	NA	NA	0.91	NA
Clptm1	0.55	1.19	1.25	0.75	1.43
Clptm1l	NA	NA	2.34	2.32	2.83
Clpx	0.57	0.2	1.25	0	3.1
Clrn1	NA	NA	0.95	0.43	NA
Clrn2	NA	NA	NA	2.72	NA
Clrn3	NA	NA	1.16	2.39	3.01
Clspn	3.96	0	0.74	0.21	1.19
Clstn1	1.1	0	2.66	1.25	9.24
Clstn2	2.41	0.57	0.95	0	1.73
Clstn3	0	2.83	1.75	0.32	2.98
Clta	0.18	0.63	0.92	5.22	1.63
Cltb	0.02	0.61	0.32	0.66	0.41
Cltc	0	0.1	1.67	0	0
Clu	0.39	2.42	0	2.98	NA
Cluap1	NA	1.03	2.44	0	0.6
Cluh	NA	2.22	1.08	3.17	NA
Clvs1	NA	NA	0	1.31	NA
Clvs2	NA	NA	1.38	0.41	NA
Clybl	0.59	NA	2.02	2.17	0.56
Cma1	NA	2.15	0.03	0.76	2.63
Cmah	0	NA	NA	NA	0
Cmas	0.22	2.13	2.56	0.59	0
Cmbl	NA	NA	2.66	2.59	1.98
Cmc1	NA	NA	0.26	0.61	0
Cmc2	NA	1.28	3.34	0.22	NA
Cmip	NA	NA	0.62	1.23	NA
Cmklr1	2.14	1.22	0.86	3.94	1.66
Cmpk1	NA	0.54	2.1	0.49	0
Cmpk2	NA	NA	0.13	0.04	0
Cmss1	NA	NA	1.2	1.4	NA
Cmtm1	NA	NA	NA	0	0.06

Cmtm3	NA	NA	2.85	1.48	NA
Cmtm4	NA	NA	NA	0	NA
Cmtm5	NA	NA	0.88	1	1.5
Cmtm6	NA	0	3.5	1.03	0
Cmtm7	NA	NA	2.76	0.74	0
Cmtm8	NA	NA	2.23	0.94	0
Cmya1	0.15	NA	NA	NA	NA
Cmya3	1.25	NA	NA	NA	NA
Cmya5	0.16	NA	0.19	NA	0
Cnbd2	NA	NA	0.34	0.81	NA
Cnbp	NA	0.13	3.7	1.59	0
Cndp1	9.1	NA	0	0.72	0
Cndp2	0	1.9	0.9	0	0
Cnep1r1	NA	NA	2.13	0.65	NA
Cnfn	13.72	NA	0.65	0.47	0
Cnga1	0	2.05	0.09	NA	2.59
Cnga2	2.18	NA	NA	0.78	NA
Cnga3	1.11	NA	NA	1.43	NA
Cnga4	0.37	NA	2.01	3.48	NA
Cngb1	NA	1.72	0.69	1.47	NA
Cngb3	0.69	1.67	0.24	5.66	0
Cnih	0	1.93	2.09	0.05	0
Cnih2	NA	NA	3.08	1.52	NA
Cnih3	NA	2.66	0	2.81	0
Cnih4	NA	0	2.9	1.57	2.11
Cnksr1	1.56	0.88	0.24	0.09	0
Cnksr2	0	0.34	0.65	1.84	1.25
Cnksr3	NA	NA	0.88	0.42	1.98
Cnn1	0	3.01	2.23	1.77	0.23
Cnn2	0	0.43	1.45	0.21	0
Cnn3	1.35	1.19	1.76	0.55	NA
Cnnm1	0.24	1.26	1.06	1.56	2.25
Cnnm2	0.45	0.47	0.95	0	1.29
Cnnm3	0	0	0.94	1.33	0
Cnnm4	3.94	1.82	1.76	7.18	0
Cno	0.49	NA	NA	NA	2.1
Cnot1	NA	0	0.11	0	0.53
Cnot10	1.01	NA	0	0.72	0
Cnot11	NA	NA	1.35	7.63	NA
Cnot2	0	0.06	1.04	0.85	0.73
Cnot3	0.14	0.67	2.47	1.87	0

Cnot4	0.17	0.32	0.79	0.61	0
Cnot6	NA	0.76	1.33	0	0
Cnot6l	1.07	NA	0.57	1.47	0
Cnot7	0	0.63	0.79	0.84	3.2
Cnot8	0.23	1.33	0.94	0.48	3.46
Cnp	NA	2.09	1.36	1.92	1.51
Cnppd1	NA	0.51	0.24	3.25	NA
Cnpy1	NA	NA	1.38	1.21	NA
Cnpy2	NA	0.78	1.64	0.79	0.06
Cnpy3	NA	1.14	1.63	1.66	0
Cnpy4	NA	2.47	1.67	0.45	1.31
Cnr1	0.23	0.91	0	0.88	2.11
Cnr2	2.97	0.16	2.28	1.28	4.73
Cnrip1	NA	NA	0.65	0.8	0.97
Cnst	NA	NA	2.28	0.32	NA
Cntd1	NA	NA	0.22	0.45	0
Cntf	0.31	NA	NA	NA	NA
Cntfr	0.42	0	0	0.81	2.01
Cntln	NA	0	1.19	1.14	1.27
Cntn1	0.36	0.19	0.66	0.97	2.07
Cntn2	0.95	0.04	0	1.33	1.59
Cntn3	1.25	NA	1.27	0.01	3.02
Cntn4	0.76	NA	0.74	1.7	1.98
Cntn5	NA	NA	NA	5.42	NA
Cntn6	2.93	0.24	2.97	2.09	0.11
Cntnap1	1.26	2.68	0	1.05	0.19
Cntnap2	0.55	0.73	0.8	1.39	0.98
Cntnap3	NA	NA	NA	0.11	NA
Cntnap4	0.26	NA	1.26	1.71	2.72
Cntrob	NA	NA	2.92	0	0.1
Coa3	NA	2.02	1.1	2.67	NA
Coa4	NA	1.52	0.83	1.21	NA
Coa5	NA	NA	0.51	0.12	NA
Coa6	NA	NA	2.58	1.34	NA
Coasy	0.56	2.97	0.68	3.39	0.96
Cobl	0.51	1.35	0.67	1.71	1.92
Cobll1	3.67	1.57	1.18	0.99	1.22
Cobra1	NA	NA	NA	NA	0
Coch	0.08	3.16	0.47	0	2.09
Cog1	NA	NA	1.16	NA	0
Cog2	0.61	1.17	0.8	1.99	2.83

Cog3	1.31	NA	4.32	0.94	6.02
Cog4	3.56	0.29	0.91	0	2.44
Cog5	0.62	1.6	0.5	0.03	0
Cog6	0.66	NA	1	0.32	0
Cog7	0.58	0.73	1.9	0	0
Cog8	0.94	1.09	1.21	2.2	NA
Coil	0	0.78	0	1.31	0
Col10a1	2.95	0.05	1.21	0.1	0
Col11a1	0.22	1.52	0.62	1.64	0.01
Col11a2	0.82	1.66	2.31	2.21	0
Col12a1	0.82	NA	0.59	1.87	2.33
Col13a1	1.35	1.29	0	1.26	4.19
Col14a1	0.77	0.66	0.76	3.65	2.98
Col15a1	22.04	2.46	1.82	0	1.95
Col16a1	1	0	0.21	0.84	1.13
Col17a1	0.28	1.27	0	0.76	2.48
Col18a1	0.5	1.82	0.91	0	1.18
Col19a1	1.42	1.77	1.34	0	1.53
Col1a1	1	0.48	0.84	1.79	0.01
Col1a2	0.88	0.62	3.3	1.02	0.99
Col20a1	NA	NA	1.03	1.88	1.58
Col22a1	NA	NA	0.36	0.95	1.02
Col23a1	1.24	NA	0.69	1.85	0
Col24a1	0.29	NA	6.83	0.67	0
Col25a1	0.65	NA	0.52	1.16	1.24
Col26a1	NA	NA	2.36	1.98	NA
Col27a1	0.19	NA	0.57	0.27	0
Col28a1	NA	NA	NA	2.18	NA
Col2a1	0.09	0.85	3.94	2.84	0
Col3a1	1.44	0.89	2.09	1.17	1.07
Col4a1	2.96	0.51	0.8	0	1.67
Col4a2	3.14	0.4	2.09	0	0
Col4a3	2.35	0	0.42	0	3.07
Col4a3bp	0.22	0.02	0.4	0.34	1.51
Col4a4	4.98	0.71	0.1	5.63	0.1
Col4a5	0.57	0.01	0.09	5.72	1.02
Col4a6	0.61	1.05	0.7	0.65	2.67
Col5a1	0.27	0.93	1.86	0	1.43
Col5a2	0.49	0.61	1.04	0	2.1
Col5a3	1.78	1.36	2.5	2.59	2.72
Col6a1	0.57	0.47	2.79	0	3.16

Col6a2	0.45	0	1.64	0	1.08
Col6a3	1.43	0.72	0.87	0.85	3.27
Col6a5	NA	NA	0.8	0.87	NA
Col6a6	NA	NA	NA	0	NA
Col7a1	0.34	0	2.36	NA	0
Col8a1	0.53	0	0.12	2	0.04
Col8a2	2.29	1.83	1.27	1.15	5.25
Col9a1	0.35	1.45	1.03	1.41	0
Col9a2	0.53	2.08	0.54	1.21	0
Col9a3	4.19	1.84	0.94	NA	0.41
Colec10	NA	2.87	0	0.13	1.21
Colec11	1.32	2.34	0.2	1.21	0
Colec12	0.27	1.41	0.45	1.99	2.12
Colgalt2	NA	2.48	1.84	0.74	NA
Colq	NA	0	0	1.21	1.79
Commd1	0.44	NA	0.2	1.45	1.98
Commd10	0.34	1.03	0.83	0.8	0
Commd2	1.34	NA	1.06	0.7	1.56
Commd3	NA	0	1.74	NA	0
Commd4	3.71	0.77	1.41	1.76	0.64
Commd5	0.75	NA	0	0.15	0.08
Commd6	0.63	NA	0	1.63	2.41
Commd7	0.02	NA	2.25	0	NA
Commd8	0	2.33	2.37	0.13	0
Commd9	0	0.3	2.93	0	0
Comp	0.9	2.98	1.35	0	0
Comt	0.73	0.8	1.03	1.58	NA
Comtd1	0	NA	1.14	1.22	1.89
Copa	0.56	0.43	0.04	NA	0
Copb1	0	0.3	2.49	0.04	1.22
Copb2	0.25	0.37	2.55	0.18	0.94
Cope	0.3	1.02	0.56	1.58	0.86
Copg	0	NA	NA	NA	0.81
Copg1	NA	1.66	0.95	0	NA
Copg2	0.98	NA	0.61	NA	0.64
Coprs	NA	NA	2.34	1.41	NA
Cops2	0	2.18	0.32	0.67	0
Cops3	0	0.8	0.48	0.38	2.51
Cops4	0.18	1.14	0	0.61	0.85
Cops5	0	0.02	1.54	2.11	0
Cops6	0.69	1.17	0.54	1.5	1.44

Cops7a	0	1.56	0.71	0.69	0
Cops7b	0.78	0	1.4	1.17	0.81
Cops8	0.54	0.74	1.09	0	1.57
Copz1	1.55	0	0.69	0	0
Copz2	0.59	0.46	1.92	4.27	3.63
Coq10a	NA	NA	1.27	0.35	0
Coq10b	NA	0.27	2.25	1.12	0
Coq2	NA	0	1.58	0.89	3.47
Coq3	0	0	1.68	1.36	0.7
Coq4	2.18	3.27	1.28	11.58	0
Coq5	NA	NA	0.42	0.77	0
Coq6	0.11	0	0.55	0	2.94
Coq7	0.34	0	2	0.64	2.39
Coq9	NA	0	1.4	1.16	0.88
Corin	1.83	0	1.47	1.53	0
Coro1a	0.01	1.9	2.41	0	1.19
Coro1b	0.53	1.66	3.25	1.03	0
Coro1c	0.04	0.46	1.03	0.62	0
Coro2a	0.41	0.98	0.99	0.99	1.36
Coro2b	0.58	1.92	0.71	0.38	2.07
Coro6	1.01	NA	0.75	1.08	NA
Coro7	NA	0	0.6	1.3	0
Cort	0.97	2.28	4.82	NA	11.5
Cotl1	0	2.43	0.22	1.42	1.69
Cova1	1.45	NA	NA	NA	NA
Cox10	0.08	0.81	0.42	3.23	0
Cox11	0.65	0.27	1.48	1.18	0
Cox14	NA	NA	0.82	0.6	NA
Cox15	0.35	0.48	0.81	0.96	0
Cox16	NA	1.17	0	2.63	1.59
Cox17	1.69	0	1.87	1.02	1
Cox18	NA	NA	0.62	0.88	2.4
Cox19	NA	NA	2.28	0	0
Cox20	NA	0.21	1.4	NA	NA
Cox4i1	0.66	0.8	2.43	0.15	0
Cox4i2	3.42	NA	2.63	1.22	0.18
Cox4nb	NA	NA	NA	NA	0
Cox5a	0	0.71	1.11	0.39	0
Cox5b	0.33	0	1.58	0	0
Cox6a1	0.5	1.83	1.16	0	1.21
Cox6a2	0.5	0.89	0	1.95	2.69

Cox6b1	NA	0.02	2.07	0.42	0
Cox6b2	NA	NA	2.82	1.79	NA
Cox6c	1.62	1.01	0.83	4.31	0
Cox7a1	0.74	0.94	3.07	1.43	0.7
Cox7a2	0.15	1.16	2.01	1.26	0.57
Cox7a2l	0.14	3.26	2.14	0.75	0
Cox7b	0.41	NA	NA	0	NA
Cox7b2	NA	NA	0.76	0.98	0
Cox7c	0.62	1.82	2.38	NA	NA
Cox8a	1.16	1.8	3.46	0	1.21
Cox8c	0.95	NA	1.28	2.18	0.03
Cp	0.46	1.33	1.3	2.26	2.18
Cpa1	0.95	2.79	1.2	1.31	7.12
Cpa2	NA	1.52	0.07	1.21	1.84
Cpa3	0.64	0	0.03	0	2.51
Cpa4	1.06	1.56	0.21	0	0
Cpa5	1.39	NA	2.28	1.01	1.63
Cpa6	NA	NA	3.03	0	0
Cpb1	1.42	2.44	3.08	2.15	0.02
Cpb2	1.6	0.86	0.62	1.81	1.3
Cpd	0.26	0.09	3.07	0	0
Cpe	2.92	0.92	1.04	1.47	NA
Cpeb1	2.57	0.19	0.41	0	1.47
Cpeb2	0.72	NA	0.07	1.51	1.9
Cpeb3	0	1.15	0.19	2.63	0.63
Cpeb4	1.51	NA	1.63	1.38	3.08
Cped1	NA	1.23	1.01	1.56	NA
Cplx1	2.27	NA	0.1	3.52	0.92
Cplx2	2.13	0.89	0.03	1.38	0
Cplx3	NA	3.04	1.53	0.63	NA
Cplx4	NA	NA	NA	0.69	NA
Cpm	0	2.25	2.76	0.45	2.18
Cpn1	0.46	0.34	2.95	0.56	0
Cpn2	NA	1.44	0.92	1.33	0.21
Cpne1	0	2.29	0.38	NA	0.68
Cpne2	0.26	NA	1.52	1.32	0.23
Cpne3	0	0.56	4.11	0.54	1.24
Cpne4	1.27	NA	0.59	1.85	0
Cpne5	7.1	NA	0	0	3.37
Cpne6	0	1.36	4.86	0.61	5.99
Cpne7	0	2.26	1.38	1.15	1.24

Cpne8	0.27	NA	1	0	0
Cpne9	NA	NA	1.26	0.7	0
Cpo	2.11	NA	NA	NA	NA
Cpox	0.64	1.52	1.6	1.58	0
Cpped1	NA	0	1.35	0.2	0
Cpq	NA	1.39	1.36	1.2	NA
Cps1	0.28	2.03	0	1.69	4.58
Cpsf1	0.53	0	3.98	0	3.33
Cpsf2	0.13	NA	4.08	0.84	0.45
Cpsf3	0	NA	1.51	1.3	0
Cpsf3l	NA	0	0.16	NA	0
Cpsf4	2.96	0.86	1.17	1.11	0
Cpsf4l	NA	NA	NA	0.73	NA
Cpsf6	0	0.04	0.19	2.48	0
Cpsf7	NA	0	2.08	1.83	1.79
Cpt1a	1.14	1.45	1.62	1.14	0
Cpt1b	0.79	NA	NA	NA	NA
Cpt1c	1.58	NA	0.88	0.03	2.07
Cpt2	0.04	0.66	0.85	0	0
Cpvl	0.01	0.14	1.77	1.87	5.61
Cpxcr1	NA	NA	NA	1.7	NA
Cpxm1	4.03	NA	1.95	0.27	0
Cpxm2	1.24	NA	1.38	1.48	1.76
Cpz	0	NA	NA	2.09	3.04
Cr1l	NA	NA	0.51	1.17	3.25
Cr2	0.43	0	3.72	1.77	0.8
Crabp1	0.87	3.25	0	1.4	0
Crabp2	0.42	0	0	0	1.84
Cradd	1.11	0	1.42	1.15	0
Cramp1l	NA	NA	2.04	2.83	0
Crat	0.56	0.49	1.83	0.46	0.73
Crb1	0.47	0.03	0.9	0.85	2
Crb2	NA	NA	NA	0.51	NA
Crb3	10.95	NA	0	0.92	1.58
Crbn	0.54	3.48	0.69	2.8	2.06
Crcp	NA	0.88	1.1	0.96	NA
Crct1	NA	0.63	0.04	2.42	0
Creb1	0.39	0.91	1.95	0.79	0
Creb3	0.22	0.86	0.87	NA	0
Creb3l1	3.2	2.11	0	0.94	0
Creb3l2	0	1.44	0.88	1.61	1.37

Creb3l3	0.28	NA	1.91	1.32	1.42
Creb3l4	0	NA	2.04	1.24	5.47
Creb5	0.86	0	0.77	NA	1.47
Crebbp	0.38	0	1.15	1.8	0
Crebl1	1.86	NA	NA	NA	NA
Crebl2	NA	0	0.98	2.72	5.84
Crebrf	NA	NA	1.36	3.03	NA
Crebzf	NA	0.29	0.52	2.19	0
Creg1	NA	3	2	0.6	0.52
Creg2	6.47	NA	0.83	0.87	NA
Creld1	0.75	0.19	1.14	0.91	0.32
Creld2	NA	1.9	1.51	0.82	5.26
Crem	1.4	0.41	1.31	2	0
Crh	2.48	0	0	1	3.52
Crhbp	3.76	2.83	0	0	0.19
Crhr1	0	0.17	1.52	0.26	1.37
Crhr2	0	0.74	0.34	0	3.22
Crim1	0	1.26	0.41	2.52	2.29
Crip1	1.05	0.81	0.97	0.32	0
Crip2	1	0	2.04	2.31	0
Crip3	2.17	NA	0	0.97	2.3
Cript	NA	1.3	1.63	0.92	0.24
Crisp1	1.02	0.19	0	6.41	0
Crisp2	1.01	1.04	1.6	2.2	0
Crisp3	0	3.72	0.35	0.24	0
Crispld1	NA	NA	0.75	0.41	0
Crispld2	NA	3.25	1.04	0	0
Crk	0.61	0.18	1.03	0.08	2.9
Crkl	0	0.31	0.95	2.86	0.52
Crlf1	1.35	1.5	1.67	0.87	0
Crlf2	NA	3.32	2.69	1.61	0
Crlf3	0	0.59	0.69	0.8	2.69
Crls1	NA	NA	2.46	1.07	0
Crmp1	0	1.16	3.28	1.18	5.27
Crnkl1	0	1.14	1.33	0	4.75
Crnn	NA	NA	NA	2.61	NA
Crocc	NA	0.89	1.81	1.95	0.09
Crot	0	0.06	1.14	0.48	0.01
Crp	0.94	1.21	0.93	0.9	21.17
Crsp2	1.56	NA	NA	NA	NA
Crsp3	0	NA	NA	NA	NA

Crsp6	1.48	NA	NA	NA	NA
Crsp7	0.59	NA	NA	NA	NA
Crsp8	0.34	NA	NA	NA	NA
Crsp9	1.41	NA	NA	NA	NA
Crtac1	3.31	0.56	0.2	1.02	0.42
Crtam	0.54	0	2.57	0	6.42
Crtap	0.3	0.03	0.63	8.56	3.75
Crtc1	NA	0.21	0.97	1.09	0.6
Crtc2	NA	NA	1.87	2.13	1.76
Crtc3	NA	2.15	1.87	2.04	0.85
Crx	0.5	1.59	0.49	1.66	0.73
Cry1	0.32	2.2	2.82	3.61	6.36
Cry2	0.52	0	1.41	0.31	1.9
Cryaa	0	3.28	0.91	1.21	4.32
Cryab	2.67	0.78	1.07	NA	3.06
Cryba1	0	1.44	1.83	1.77	0
Cryba2	0	3.49	0.18	1.32	0.55
Cryba4	2.05	2.26	1.92	2.2	3.66
Crybb1	1.79	1.76	0.68	1.18	0
Crybb2	0	1.99	0.82	4.03	0
Crybb3	0.23	1.32	0	0.96	0
Crybg3	NA	1.51	1.11	0	3.82
Cryga	0.99	1.25	0.65	6.3	0
Crygb	0.82	1.77	0	NA	0
Crygc	0.64	0.14	1.17	1.07	NA
Crygd	1.93	3.17	0.94	1.86	0
Crygn	4.61	NA	0.18	1.63	NA
Crygs	1.18	NA	1.18	NA	2.59
Cryl1	0.24	0	1.58	0.87	0
Crym	0.81	1.54	1.05	0.14	0
Cryz	0.33	0.04	3.27	0.19	0
Cryzl1	0.04	1.01	2.38	1.11	0
Cs	0	2.41	0.88	1.92	0
Csad	1.13	1.4	1.17	2.23	1.77
Csda	1.2	NA	NA	NA	0.04
Csdc2	NA	2.13	0.21	4.5	0
Csde1	NA	0.58	1.68	0.66	1.95
Cse1l	0.09	0.45	1.56	0	0
Csf1	0.45	2.01	4.05	1.99	0
Csf1r	0.78	2.74	2.14	0.83	0
Csf2	0.02	0.71	1.06	2.01	1.52

Csf2ra	0	0	1.37	0.85	1
Csf2rb	NA	3.62	2.67	2.78	0
Csf3	1.02	0.61	3.7	0.55	0
Csf3r	0.37	1.44	1.46	0.73	0
Csgalnact1	NA	1.58	1.79	0.24	0
Csgalnact2	NA	0.1	2.92	1.39	2.14
Csh1	0.29	NA	NA	NA	NA
Csk	0	3.39	1.47	1.85	1.9
Csmd1	0	NA	0.5	1.36	2.76
Csmd2	0	NA	0.05	0	0
Csmd3	0.34	NA	1.04	1.01	2.26
Csn1s1	NA	1.49	0	2.6	2.68
Csn2	NA	3.91	0.95	1.11	2.63
Csn3	NA	0	0.43	1.8	0.93
Csnk1a1	0.67	0.4	0.77	0.59	0.64
Csnk1d	0.65	0.88	1.31	0	0
Csnk1e	0.25	0.82	0.08	NA	0.27
Csnk1g1	0.82	0	1.2	0.64	0.73
Csnk1g2	0.68	0.14	1.4	1.06	1.11
Csnk1g3	0.62	2.88	1.14	0.17	0
Csnk2a1	0.49	0.4	0.48	NA	0
Csnk2a2	0.32	1.12	1.98	1.24	1.75
Csnk2b	2.05	2.39	0.38	0	0
Cspg4	0	0.92	0.58	0	2.06
Cspg5	0.38	0.59	0.34	2.94	2.33
Cspp1	NA	0.1	0.63	0.07	0.13
Csrnp1	NA	NA	2.29	0.31	0
Csrnp2	NA	0.84	3.9	1.56	0.99
Csrnp3	NA	1.17	0.72	1.72	0.44
Csrp1	0	1.41	3.36	1.28	0
Csrp2	0.47	2.59	0.93	1.11	1.53
Csrp2bp	0.1	NA	1.05	NA	0
Csrp3	0.53	1.42	2.92	0.09	2.05
Cst11	0	NA	2.37	0.75	0
Cst3	3.13	2.62	2.58	10.62	0.06
Cst6	0	0.25	1.44	0	0
Cst7	0.34	0.72	0.29	0.85	0.29
Cst8	2.31	1.03	1.92	0.81	1.87
Cst9	0	NA	1.06	0.99	NA
Csta	NA	0	4.28	0	0
Cstb	0.26	0.53	0.6	0.11	0.18

Cstf1	0.52	0	0.95	3.44	0
Cstf2	0	0	2.35	1.57	0.77
Cstf2t	0.92	0.95	0.15	4.12	0
Cstf3	0.54	0	0.49	0	0.97
Cstl1	3.69	NA	NA	1.77	NA
Ctag2	NA	2.53	0	0.72	0
Ctage5	NA	0	2.9	1.09	1.23
Ctbp1	0	0	0.72	2.2	NA
Ctbp2	0.3	0.04	2.28	0	0.78
Ctbs	0.71	0.8	2.09	0.73	1.01
Ctc1	NA	0	1.16	0	NA
Ctcf	0.64	0	0.65	0	0
Ctcfl	NA	NA	NA	0.7	NA
Ctdnep1	NA	2.55	0.15	0.19	NA
Ctdp1	0.5	1.79	1.65	3.23	2.1
Ctdsp1	0.88	1.63	1.65	1.26	0
Ctdsp2	0.95	0	1.36	0	0.78
Ctdspl	0.08	1.4	1.3	1.55	6.89
Ctdspl2	NA	NA	0.5	1.27	0
Ctf1	1.09	1.47	0	2.22	2.46
Ctgf	0.99	1.25	2.36	0.94	0.77
Cth	0.47	1.11	0.11	1.55	0.88
Cthrc1	0	NA	3.11	0.61	2.41
Ctif	NA	0.69	0	0.72	NA
Ctla4	0.51	1.46	2.42	1.37	1.6
Ctnna1	NA	0.73	1.57	0	0
Ctnna2	NA	2.23	4.57	0.86	4.62
Ctnna3	NA	1.46	0	2.7	0
Ctnnal1	NA	2.1	0	0.25	0
Ctnnb1	NA	0.3	1.15	0	0
Ctnnbip1	NA	1.83	3.09	NA	1.36
Ctnnbl1	1.04	1.5	1.22	2.82	3.09
Ctnnd1	NA	2.15	0.37	NA	0.17
Ctnnd2	NA	1.23	2.26	1.68	NA
Ctns	0.35	0.58	2.34	0	1.86
Ctps	0.26	NA	NA	NA	3.84
Ctps2	0	1.88	0.28	1.1	0
Ctr9	NA	0.05	1.13	0.64	1.17
Ctrc	1.23	0	2.03	0.91	0.08
Ctrl	0.17	0.14	0.2	0.52	2.42
Ctsa	NA	0.85	1.68	0	0.7

Ctsb	0.73	1.08	1.26	NA	0.69
Ctsc	0.34	1.46	3.34	0.62	0
Ctsd	NA	1.41	0.51	3.39	0
Ctse	1.27	2.36	3.42	2.05	2.41
Ctsf	2.82	0.03	0.93	0.34	1.45
Ctsg	5.78	1.07	1.11	3.99	0
Ctsh	0.44	3.01	0.8	0.81	1.44
Ctsk	0	0.22	3.31	1.76	0.82
Ctso	1.4	0	2.21	3.34	0
Ctss	0.56	2.11	2.43	0.12	2.02
Ctsw	0	0.07	0.89	1.15	2.98
Ctsz	2.61	0.99	1.28	1.57	1.67
Cttn	0	1.23	0.94	0.38	0
Cttnbp2	1.78	NA	2.61	0	0
Cttnbp2nl	NA	NA	2.67	0	0.03
Ctu1	NA	NA	NA	1.1	NA
Ctu2	NA	NA	5.73	2.1	0.21
Ctxn1	NA	NA	1.56	1.58	3.26
Ctxn3	NA	NA	1.36	0.93	2.14
Cubn	1.23	2.32	1.64	0	2.39
Cuedc1	3.34	0.54	1.21	0.32	0.36
Cuedc2	0.41	0.05	1.95	NA	0
Cugbp1	0.33	NA	NA	NA	NA
Cugbp2	0	NA	NA	NA	NA
Cul1	0.48	0.27	1.64	0.2	1
Cul2	0.06	0.73	1.88	0.52	0
Cul3	0.99	0	1.6	0	0.87
Cul4a	0.5	0.18	0.06	0	0
Cul4b	1.4	1.83	1.69	1.03	0
Cul5	0.48	0.28	1.59	0.32	0
Cul7	0.6	0.43	1.51	0	0
Cul9	NA	0	1.5	1.65	2.07
Cuta	NA	0	2.34	0	0
Cutc	0	2.97	4.23	0	0
Cutl1	1.14	NA	NA	NA	NA
Cutl2	0.52	NA	NA	NA	NA
Cux1	NA	1.18	0.62	0	1.43
Cux2	NA	0	0.53	0.27	0
Cuzd1	0	2.22	0	0.48	0.75
Cwc15	NA	NA	1.46	1.27	0
Cwc22	NA	NA	0	0.52	3.37

Cwc25	NA	1.6	1.66	0.53	NA
Cwc27	NA	NA	2.34	1.16	NA
Cwf19l1	0	1.3	0	0.95	0
Cwf19l2	0	NA	0.98	0.2	2.99
Cwh43	NA	1.42	1.25	2.34	3
Cx3cl1	0.95	0.59	1.31	0.03	1.82
Cx3cr1	2.14	0.21	2.17	0.75	0
Cxadr	0.43	0.92	0.86	0.02	1.1
Cxcl1	0.18	1.1	0.78	NA	0
Cxcl10	0.09	0.75	0.43	0	0
Cxcl11	0	0	11.65	3.19	0
Cxcl12	0.03	1.22	1.13	1.06	0.8
Cxcl13	0	1.6	0.2	1.02	10.07
Cxcl14	0.58	1.52	1.08	3.58	0
Cxcl16	0.21	NA	2.5	2.2	0.04
Cxcl17	NA	NA	1.54	0.58	5.33
Cxcl2	0.18	0.75	0.79	1.79	0
Cxcl3	NA	0.63	0	2.1	4.19
Cxcl5	0.44	1.01	0.09	2.61	0.75
Cxcl9	0	0.78	0	1.4	0
Cxcr1	NA	NA	NA	0.86	NA
Cxcr2	NA	2.92	2.36	1.88	NA
Cxcr3	1.15	0	3.15	1.85	0
Cxcr4	0.1	0	2.77	1.64	0
Cxcr5	NA	0.06	0.94	0.06	0.05
Cxcr6	1.64	0	0.45	0.93	0.93
Cxcr7	NA	2.47	0.68	2.84	0
Cxxc1	0	0	0.2	0.5	0
Cxxc4	0	0.9	0.82	0.28	0
Cxxc5	0.46	NA	0.7	1.49	0
Cxxc6	0.59	NA	NA	NA	NA
Cyb561	1.22	1.36	2.84	2.73	0.12
Cyb561a3	NA	NA	1.19	14.13	NA
Cyb561d1	10.67	NA	0	1.1	0
Cyb561d2	0	1.4	0.17	0	0
Cyb5b	NA	0	0.53	0.18	0
Cyb5d1	NA	NA	2.06	0.83	NA
Cyb5d2	NA	NA	0.39	0.29	2.29
Cyb5r1	NA	0.89	4.19	0.64	0.98
Cyb5r2	NA	3.01	0	0.84	4.09
Cyb5r3	NA	1.45	2.99	1.47	0

Cyb5r4	NA	1.7	5.02	0.62	0.11
Cyb5rl	NA	NA	2.68	0	0
Cyba	0.8	0	2.33	0.3	0
Cybasc3	NA	NA	NA	NA	0
Cybb	0.99	0	2.14	1.74	0.36
Cybrd1	0.6	1.29	0.3	1.39	0.92
Cyc1	0.35	0.36	1.96	1.55	0
Cycs	0.36	2.21	0.76	0.04	0
Cyfip1	0.99	1.58	1.23	0	1.95
Cyfip2	0.53	0.81	1.26	0	2.76
Cygb	0	NA	0	0.63	3.83
Cyhr1	0.5	1.89	0.84	1.44	0
Cylc1	4.48	0	0.94	0.58	0
Cylc2	NA	0.31	2.29	3.28	0.24
Cyld	0.9	0.84	1.3	0.61	0.16
Cyp11a1	0.3	0.95	0.7	3.57	2.28
Cyp11b1	NA	1.2	2.48	0.24	0
Cyp11b2	2.37	4.78	1.55	1.02	1.94
Cyp17a1	0.92	1.24	0	1.55	0
Cyp19a1	1.53	1.68	0	3.99	0
Cyp1a1	0.36	0.15	2.46	2.49	3.21
Cyp1a2	1.75	1.51	0.43	2.11	1.16
Cyp1b1	0	0	1.17	1.52	1.49
Cyp20a1	0.68	0.94	3.19	0.46	0.16
Cyp24a1	1.61	1.61	1.35	0	3.77
Cyp26a1	0.77	2.52	0.68	0	0.63
Cyp26b1	0	2.09	3.07	1.19	0.8
Cyp26c1	NA	NA	NA	0.43	NA
Cyp27a1	0.12	1.01	1.86	0.91	2.83
Cyp27b1	0	0.82	2.49	4.17	4.01
Cyp2e1	1.06	0	1.66	1.64	3.78
Cyp2r1	1.34	0.79	2.59	1.16	3.18
Cyp2s1	0.68	NA	0	1.81	1.27
Cyp2u1	0	0.09	0.3	3.38	1.12
Cyp2w1	NA	3.03	3.73	1.16	4.66
Cyp39a1	0.91	2.51	2.26	1.73	3.03
Cyp46a1	1.24	0.46	1.37	0.55	4.71
Cyp4b1	0.22	1.61	1.05	1.42	1.52
Cyp4x1	0.29	NA	1.6	1.13	0.88
Cyp7a1	0.5	2.81	1.95	0.62	1.66
Cyp7b1	0	1.65	0.03	2.23	0.17

Cyp8b1	0.54	NA	0.78	0.64	1.4
Cyr61	0.94	0.97	1.39	0.46	1.76
Cys1	1.74	NA	0.13	2.78	0
Cysltr1	0.62	1.02	1.66	5.25	0
Cysltr2	4.1	0.89	2.32	1.92	0
Cystm1	NA	NA	2.85	0	NA
Cyth1	NA	0	1.06	0.69	1.3
Cyth2	NA	1.12	2.51	0.54	0
Cyth3	NA	1.25	1.1	1.58	0
Cyth4	NA	0.9	0.71	1.71	9
Cytip	NA	2.54	2.68	4.24	0
Cytl1	NA	1.24	9.23	2.81	2.77
Cytsa	NA	NA	NA	NA	0
Cytsb	NA	NA	NA	NA	0
Cyyr1	3.14	NA	0.34	1.84	9.01
D15wsu75e	0.93	NA	NA	NA	NA
D2hgdh	NA	NA	0.92	0	2.03
D4st1	0	NA	NA	NA	NA
Daam1	0.69	0.45	0	0	0
Daam2	4.87	0.1	0.34	0.82	0
Dab1	0.61	0.67	0.95	1.1	0.49
Dab2	0	1.32	1.61	1.81	1.4
Dab2ip	0.49	NA	2.93	1.58	0
Dach1	0.66	0	0.94	1.17	1.24
Dach2	0.62	NA	1.11	1.61	0.36
Dact1	0	3.25	2.13	2.21	0
Dact2	0.31	NA	0	0.87	NA
Dact3	NA	NA	0.56	1.38	0
Dad1	0.01	0	2.25	11.18	0
Dag1	0.74	0.24	0.29	0.53	0
Dagla	NA	2.04	0.73	0.96	0
Daglb	NA	NA	0.72	3.74	0.07
Dak	NA	0	2.13	0.51	0.25
Dalrd3	NA	2.88	1.75	NA	1.8
Dancr	NA	NA	3.07	NA	NA
Dand5	NA	NA	0.13	1.06	NA
Dao	NA	1.57	1.05	1.38	2.47
Dap	0.07	0.55	1.03	2.67	0
Dap3	0.06	1.32	3.03	1.06	2.36
Dapk1	0.61	0.17	5.38	3.61	0.68
Dapk2	2.67	2.23	0.08	5.26	0

Dapk3	0.9	0.02	0.68	0.78	1.47
Dapl1	NA	NA	1.95	0.01	0
Dapp1	1.05	2.48	2.75	0.66	0
Darc	NA	1.41	1.02	5.75	2.13
Dars	0.11	1.19	1.35	0.23	1.96
Dars2	NA	1.53	1.13	NA	1.65
Daw1	NA	NA	0	1.83	NA
Daxx	1.3	2.02	1.7	0.01	3
Dazap1	0	2.6	0.71	0.41	2.16
Dazap2	0	0	1.87	0	2.19
Dazl	1.61	0.1	1.36	0.79	2.96
Dbc1	NA	0	1.29	0.38	0
Dbf4	NA	0	2.75	0.72	0
Dbh	0.56	1.32	0.83	1.87	1.41
Dbi	0.46	0	0.66	1.77	0
Dbn1	0.66	1.46	1.94	1.79	0
Dbndd1	NA	1.87	2.47	0.63	1.5
Dbnl	0	NA	0.66	1.16	0
Dbp	0.02	0.56	2.58	NA	0.31
Dbpht2	NA	NA	1.09	NA	NA
Dbr1	0.74	1.27	0.76	0.85	1.71
Dbt	0.45	0.47	0.1	1.46	1.65
Dbx1	2.74	NA	NA	1.56	NA
Dbx2	2.92	NA	NA	0.15	NA
Dcaf10	NA	3.46	1.82	2.55	0
Dcaf11	NA	0.01	0	0.62	0
Dcaf12	NA	NA	0.81	0.61	1.67
Dcaf12l1	NA	NA	0	1.54	1.24
Dcaf12l2	NA	NA	NA	3	NA
Dcaf13	NA	2.34	3.82	0	2.39
Dcaf15	NA	0.27	0.57	2.69	5.46
Dcaf17	NA	0.99	0.74	0.65	1.59
Dcaf4	NA	0.49	2.25	0.26	0.7
Dcaf5	NA	NA	1.59	0.89	0
Dcaf6	NA	0.96	1.16	0.46	0
Dcaf7	NA	0	0.43	0	0
Dcaf8	NA	0	0.74	0	0
Dcakd	NA	0.82	1.19	3.13	0
Dcamkl1	0.74	NA	NA	NA	NA
Dcbld1	0.75	NA	1.19	0.6	0
Dcbld2	1.22	1.04	1.31	0	0

Dcc	0.29	0.43	0.43	0	0
Dcdc2	0	NA	NA	NA	NA
Dcdc2b	NA	NA	NA	NA	1.94
Dchs1	0.1	1.04	1.48	1.38	1.15
Dchs2	NA	NA	NA	0	NA
Dci	0.72	NA	NA	NA	0
Dck	0.46	1.98	2.3	0	0
Dclk1	NA	0.02	1.06	0.33	1.21
Dclk2	NA	1.79	0.66	0.57	1.72
Dclk3	NA	NA	0	4.32	0
Dclre1a	0	1.15	0.3	1.42	2.05
Dclre1b	0	0.91	1.62	0	0
Dclre1c	0	0.32	1.41	1.09	3.8
Dcn	0.38	0.64	0.04	1.06	2.49
Dcp1a	NA	1.36	1.24	0	0.14
Dcp1b	NA	NA	0.65	0.76	0
Dcp2	NA	0.79	1.08	1.46	0.7
Dcps	NA	0	0.55	2.03	1.8
Dcst1	NA	NA	1	0.6	NA
Dcst2	NA	NA	0.94	1.13	5.85
Dcstamp	NA	2.14	0.99	0.93	NA
Dct	0.34	0	0.5	0.34	0
Dctd	0.25	1.73	1.96	2	7.24
Dctn1	1	1.03	1.53	0	0.27
Dctn2	0	2.84	1.99	0.01	0
Dctn3	0.24	1.49	0.19	3.28	0
Dctn4	0	0.9	0.77	0.93	0
Dctn5	0.66	2.42	0.32	2.14	0
Dctn6	0.78	0	0.72	1.8	0.13
Dctpp1	NA	0.74	2.35	1.35	0
Dcun1d1	NA	2.05	1.39	0.41	0
Dcun1d2	NA	0.91	2.77	NA	0
Dcun1d3	NA	NA	2.29	0.43	0
Dcun1d4	NA	1.12	2.37	0.64	1.85
Dcun1d5	NA	NA	0.81	0.7	1.51
Dcx	0	0.41	0.53	2.3	3.35
Dcxr	1.11	0.35	2.8	3.44	0
Dda1	NA	1.18	0.74	0	1.19
Ddah1	0.32	2.3	0.92	0.83	1.57
Ddah2	0	1.63	1.92	0.71	6.69
Ddb1	0.76	0	0.95	0	2.12

Ddb2	0	0.35	2.67	1.35	2.5
Ddc	1.19	1.14	0.59	1.12	1.57
Ddef1	0.88	NA	NA	NA	NA
Ddhd1	0.13	NA	1.78	2.13	0
Ddhd2	0	0	0.95	0.32	0
Ddi1	NA	NA	0	2.66	1.29
Ddi2	NA	2.07	0.44	NA	0.38
Ddit3	0.49	0	4.84	NA	NA
Ddit4	0	1.21	0.47	0.81	1.99
Ddit4l	0.04	NA	0.37	6.57	0.91
Ddn	0	2.13	1.15	0	1.69
Ddo	NA	1.23	0.72	9.63	1.15
Ddost	0.47	0	0.51	1.4	0
Ddr1	1.08	0	0.91	0.9	1.43
Ddr2	0.79	1.05	1.04	0	2.33
Ddrgk1	NA	2.25	3.34	1.88	0
Ddt	1.12	2.48	3.76	NA	0
Ddx1	0.23	1.76	1.98	0.49	0
Ddx10	0	0	1.57	0.53	2.71
Ddx11	1.05	0	1.32	NA	1.24
Ddx17	NA	0.66	0.93	5.55	0.6
Ddx18	0.37	0	0.24	1.62	0
Ddx19a	NA	0	3.84	0.96	0
Ddx19b	NA	NA	0	0.27	1.13
Ddx20	0	NA	0.7	0.81	3.38
Ddx21	0.18	1.91	1.63	1.51	1.34
Ddx23	0	0.85	1.62	0.83	0.86
Ddx24	0.9	0	1.45	3.34	3.32
Ddx25	0.41	3.58	0.68	1.85	0
Ddx26b	NA	NA	1.13	0.87	4.31
Ddx27	0	0	0.02	0.96	2.34
Ddx28	0	0.84	0.14	4.01	0.94
Ddx31	0.52	1.49	0.74	2.25	0.53
Ddx39	0	NA	NA	NA	0
Ddx39b	NA	0.7	0.37	NA	NA
Ddx3x	0.83	1.57	0.5	0.24	0
Ddx3y	0.81	0.7	1.68	1.37	0.63
Ddx4	0.6	0.62	3.66	0	2.85
Ddx41	0.71	0	4.88	1.3	0
Ddx42	1.09	0	3.11	0.83	2.39
Ddx43	NA	1.68	1.67	NA	3.93

Ddx46	0.09	0	0.41	1.22	0
Ddx47	0.62	0	0.32	NA	9.89
Ddx49	NA	1.09	1.11	0.44	0.77
Ddx5	0.44	1.82	1.92	1.06	0
Ddx50	0.69	1.26	0.72	0.61	0
Ddx51	0	1.39	0.01	2.39	6.38
Ddx52	1.02	0.63	0.74	0.04	0.94
Ddx54	0.49	0.67	1.07	1.31	1.14
Ddx55	0	NA	0.07	3.26	0
Ddx56	0	0	1.44	0.68	2.47
Ddx58	0	0.3	4.16	0	0
Ddx59	NA	NA	0.35	3.03	1.45
Ddx6	0.68	0	0.73	0	0.84
Ddx60	NA	1.81	0.57	0	1.83
Deadc1	0.74	NA	NA	NA	NA
Deaf1	0.94	0	2.58	0.66	0.65
Decr1	0.05	3.7	2.21	1.66	0
Decr2	0	0.66	1.34	1.39	NA
Dedd	0	1.56	0.41	0.38	0
Dedd2	0	NA	3.63	0	0.55
Def6	0	0.94	1.38	2.43	2.97
Def8	NA	2.39	3.31	7.65	0
Defa4	NA	1.62	0	NA	0.89
Defb1	5.68	0	0	0	3.46
Defb4	NA	NA	NA	NA	2.69
Degs1	NA	0.23	2.31	1.06	0
Degs2	NA	NA	1.99	1.13	3.84
Dek	1.25	1.2	0.97	1.06	0
Dem1	NA	NA	NA	NA	1.23
Dennd1a	NA	0.75	1.31	1.56	2.4
Dennd1b	NA	1.03	0.47	2.05	1.47
Dennd1c	NA	0	0.76	4.22	1.04
Dennd2a	NA	0.99	2.61	1.21	0
Dennd2c	NA	NA	2.35	0.46	0.91
Dennd2d	NA	0	2.12	0.85	1.23
Dennd3	NA	0.49	1.35	1.04	0.33
Dennd4a	NA	1.47	0.21	0.75	0
Dennd4b	NA	2.41	0.57	1.64	3.62
Dennd4c	NA	0	2.73	1.06	0
Dennd5a	NA	1.13	1.77	1.93	0
Dennd5b	NA	0.18	0.66	2.05	0

Dennd6a	NA	NA	1.9	0	NA
Dennd6b	NA	2.02	0.43	2.34	NA
Denr	0.83	0.89	1.78	2.35	1.89
Depdc1	0.91	NA	NA	NA	NA
Depdc1b	0.16	NA	0.81	0	0
Depdc2	0	NA	NA	NA	NA
Depdc5	0.64	0	1.28	0	0.31
Depdc6	NA	NA	NA	NA	1.98
Depdc7	NA	NA	4.31	2.06	1.17
Deptor	NA	0.43	1.57	0.56	NA
Dera	NA	1.8	1.52	0.63	0
Derl1	NA	0.89	0.79	0.81	0
Derl2	NA	4.19	1.75	0	0.76
Derl3	NA	NA	0.38	1.13	0.9
Des	3.44	0.36	0.58	3.84	0
Desi1	NA	1.09	0.63	4.59	NA
Desi2	NA	0.49	2.34	1.37	NA
Det1	NA	0	0.54	4.79	1.95
Dexi	0.36	0	4.11	1.33	0
Dffa	0	0.08	1.9	0.05	0.41
Dffb	0	0	0.44	2.25	0
Dfna5	NA	2.97	1.63	0.58	0
Dgat1	1.57	2.06	0.99	0.2	0
Dgat2	3.08	NA	1.03	6.31	3.88
Dgat2l6	NA	NA	NA	1.11	NA
Dgcr14	NA	1.2	1.09	0.74	0.52
Dgcr2	0	0.93	1.48	2.16	0.59
Dgcr6	0.51	NA	NA	0.88	NA
Dgcr8	0.29	0	0.9	0	0
Dgka	0.27	0	2.39	0.34	0
Dgkb	6.42	0.05	1.41	1.41	0.63
Dgkd	0.88	0.04	2.77	3.73	0
Dgke	2.52	1.67	1.01	0.07	0.6
Dgkg	0.07	1.85	1.58	0.94	0
Dgkh	1.38	NA	1.86	3.23	NA
Dgki	NA	1.79	1.96	3.93	0
Dgkk	NA	NA	NA	1.02	NA
Dgkq	1.4	1.51	0.93	1.47	0
Dgkz	0.34	0	1.65	0.47	0
Dguok	0.08	2.75	2.33	0.88	1.85
Dhcr24	0.45	1.65	1.62	0	0

Dhcr7	0.14	2.17	1.5	0	1.84
Dhdds	0	1.17	0.12	0.03	2.07
Dhdh	NA	NA	0.2	0.98	0.74
Dhdpsl	NA	NA	NA	NA	0.78
Dhfr	0.33	1.14	3.04	NA	1.35
Dhh	1.73	NA	0.85	1.07	NA
Dhodh	0.12	1.17	2.49	1.45	1.59
Dhps	3.14	1.12	1.29	1.66	0
Dhrs1	0.27	3.15	0.92	0.44	1.07
Dhrs11	NA	0.41	0.4	1.35	0
Dhrs13	NA	NA	1.46	0.39	7.07
Dhrs2	0.97	1.39	0.81	0	0
Dhrs3	0	0	1.3	0.65	0
Dhrs4	0.69	NA	NA	NA	NA
Dhrs7	0.67	0	1.16	1.82	0
Dhrs7b	NA	2.04	2.02	0.55	0.71
Dhrs7c	NA	NA	2.18	2	0
Dhrs8	0.18	NA	NA	NA	NA
Dhrs9	0.13	NA	NA	0.43	NA
Dhrsx	0	NA	0.35	0.44	0
Dhtkd1	2.65	0.38	2.58	1.91	0
Dhx15	0.07	1.72	2.46	0.47	1.41
Dhx16	0	0	0.41	3.06	1.32
Dhx29	0	1.09	1.66	0.91	2.52
Dhx30	0.66	0	1.42	1.87	0.53
Dhx32	0.75	0	1.41	NA	0
Dhx33	0.03	NA	0.27	2.86	2.54
Dhx34	1.98	1.54	1.99	2.34	1.1
Dhx35	0	0	1.51	0.88	0.34
Dhx36	0.8	NA	1.51	1.11	1.29
Dhx37	0.91	NA	0.45	2.01	1.42
Dhx38	NA	2.02	0	2.4	1.05
Dhx40	0.67	0.4	2.97	2.12	0.77
Dhx57	NA	0	2.39	NA	0
Dhx58	NA	2.02	0.72	0.06	0
Dhx8	0	1.82	0.6	0	1.29
Dhx9	0	1.31	0.12	0.04	0
Diablo	0.12	1.64	0	NA	2.3
Dicer1	1.2	1.51	1.21	1.57	0.55
Dido1	0	0	0.64	0.65	0
Diexf	NA	0	0.01	1.19	NA

Dimt1	NA	0.66	3.65	1.13	NA
Dio1	0.55	4.01	0.78	0.69	4.92
Dio2	0	0	1.16	2.81	1.8
Dio3	1.29	NA	NA	0	NA
Dio3os	NA	NA	2.11	NA	0
Dip2a	NA	0.92	1.29	1.16	NA
Dip2b	NA	NA	0.75	0	0
Dip2c	NA	2.66	0.64	0	2.7
Diras1	0.28	NA	0.91	1.59	2.55
Diras2	1.27	2.25	1.19	3.83	1.26
Dirc2	3.54	NA	0.84	0	0
Dis3	NA	1.04	2.54	0.94	1
Dis3l	NA	NA	0	0.75	0
Dis3l2	NA	NA	0.74	0	1.3
Disc1	0	NA	NA	NA	NA
Disp1	0	NA	0.58	0.57	1.34
Disp2	1.12	NA	1.99	0.82	0.24
Dixdc1	0.65	0.45	0.02	0.74	0
Dkc1	NA	0.75	0.26	1.29	0
Dkk1	1.36	0	0.42	0.83	2.33
Dkk2	0.14	0.71	1.74	1.87	3.31
Dkk3	0.69	0.85	0.08	0.94	1.79
Dkk4	2.56	2.87	1.12	0.86	16.22
Dkkl1	NA	2.2	0.78	3.09	7.16
Dlat	0.43	0	0.51	1.03	0
Dlc1	0.62	0.01	0.76	1.03	2.82
Dld	1.08	0.1	1.71	0	1.16
Dlec1	NA	NA	NA	1.61	NA
Dleu2	4.56	0	5.4	1.76	2.05
Dleu7	NA	NA	0.92	1.41	2.16
Dlg1	NA	0.03	0.71	2.35	3.07
Dlg2	NA	1.25	0.99	0.92	0
Dlg3	NA	0.32	1.81	0.3	0.34
Dlg4	NA	1.85	0.38	0.11	2.2
Dlg5	1.62	0.01	0.41	1.29	0.8
Dlg7	0.22	NA	NA	NA	NA
Dlgap1	0.59	0.89	0.64	0.92	1.14
Dlgap2	1.62	0.5	1.12	1.16	0
Dlgap3	0	NA	0.07	0.78	0
Dlgap4	0.24	1.58	2.51	1.65	0
Dlgap5	NA	1.48	4.61	2.68	0.73

Dlk1	0.53	1.15	0	1.49	0
Dlk2	NA	0.91	2.42	0.79	1.57
Dll1	0	NA	4.43	0	2.59
Dll3	1.89	2.44	0.74	0.56	0
Dll4	1.6	NA	0.18	1.01	2.04
Dlst	0.61	0	1.56	0.79	NA
Dlx1	1.62	NA	3.89	0.78	0
Dlx2	0	0.07	3.29	0.96	8.1
Dlx3	0.25	NA	1.94	1.2	0.78
Dlx4	1.4	0.05	0.7	0.57	1.73
Dlx5	0	0.84	0.9	1.47	4.86
Dlx6	NA	2.41	0.66	0.36	0
Dlx6as	NA	NA	NA	NA	0
Dmap1	0	NA	2.31	0	4.55
Dmbt1	0.62	2.85	1.7	1.49	3.13
Dmbx1	NA	NA	2.77	0.59	2.41
Dmc1	NA	0.49	1.29	0.19	1.46
Dmd	0.19	0.35	0	0	2.69
Dmgdh	0	NA	1.01	3.01	1.04
Dmkn	NA	NA	0	3.63	0
Dmn	0.27	NA	NA	NA	NA
Dmp1	0.48	0.85	0.67	6.81	1.13
Dmpk	NA	1.24	2.14	1.23	0
Dmrt1	2.56	2.25	0	2.06	0
Dmrt2	0.04	NA	0.5	0.56	11.76
Dmrt3	7.67	NA	0.59	1.03	0
Dmrta1	1.91	NA	2.01	0	0
Dmrta2	2.51	NA	0.86	0.69	NA
Dmrtb1	0.09	NA	0.4	0	0
Dmrtc1b	NA	NA	NA	0	NA
Dmrtc2	NA	NA	1.9	1.5	0
Dmtf1	0	0.37	0.76	0.72	4.02
Dmtn	NA	0	0.35	0.98	NA
Dmwd	0.44	0.67	2.22	0.83	0
Dmxl1	NA	0.14	2.41	0.86	0
Dmxl2	NA	0.95	1.99	2.9	0
Dna2	NA	0.24	3.96	1.4	3.15
Dna2l	0	NA	NA	NA	NA
Dnaaf1	NA	0.78	1.44	0.38	NA
Dnaaf2	NA	0.11	1.3	1.26	NA
Dnaaf3	NA	NA	NA	0.39	NA

Dnah1	NA	NA	0.09	0.13	NA
Dnah10	NA	NA	NA	0.48	NA
Dnah11	NA	NA	1.84	NA	NA
Dnah12	NA	NA	4.16	0	NA
Dnah17	NA	0.15	4.99	0	NA
Dnah2	NA	0.98	0.69	2.63	NA
Dnah3	NA	NA	NA	0.1	NA
Dnah5	NA	NA	1.73	0	NA
Dnah6	NA	0.11	1.2	0.13	NA
Dnah8	NA	NA	0.11	1.56	NA
Dnah9	NA	0.55	0.71	1.44	NA
Dnai2	3.48	NA	NA	NA	NA
Dnaja1	0.61	3.09	1.42	0.02	0
Dnaja2	0	0.56	1.48	0.83	1.94
Dnaja3	0	0	0.83	0.82	1.49
Dnaja4	0.66	3.62	0.98	1.23	2.14
Dnajb1	0.54	1.85	0.63	0	0
Dnajb11	0	NA	0.75	0.42	2.58
Dnajb12	0	0.86	1.21	0	0
Dnajb13	NA	NA	0.95	0.25	1.47
Dnajb14	NA	0.31	1.56	0	2.82
Dnajb2	NA	1.21	1.59	0	0
Dnajb3	NA	NA	NA	1.58	NA
Dnajb4	0	1.35	1.1	1.57	1.2
Dnajb5	0.35	0.29	0.31	11.38	0.07
Dnajb6	0.25	2.73	1.08	0	0
Dnajb7	0.43	NA	0	2.73	NA
Dnajb8	0	NA	0	NA	2.53
Dnajb9	0.32	1.07	2.7	0.29	1.81
Dnajc1	0.23	0	1.87	1.29	0.29
Dnajc10	0.23	0.53	2.28	3.24	NA
Dnajc11	0	0	0	NA	0.93
Dnajc12	0.75	0.99	0.52	2.08	2.76
Dnajc13	0.71	0.64	0.16	0	1.42
Dnajc14	0.6	NA	2.78	2.91	0
Dnajc15	NA	0.93	1.68	0.71	0.05
Dnajc16	NA	0.56	0.43	2.51	0.47
Dnajc17	NA	0.64	0.46	NA	2.78
Dnajc18	NA	NA	0.63	0.62	0.66
Dnajc19	NA	NA	1.42	0.72	0
Dnajc2	NA	1.38	0.65	1.97	3.43

Dnajc21	NA	NA	1.16	0.47	0
Dnajc22	NA	1.18	0.74	1.53	1.44
Dnajc24	NA	2.17	2.63	0.9	0
Dnajc25	NA	NA	3.01	NA	1.7
Dnajc27	NA	NA	0.48	1.13	0.59
Dnajc28	NA	1.78	4.44	1.3	1.18
Dnajc3	8.56	0	2.72	0	1.4
Dnajc30	NA	NA	0.81	2.66	1.62
Dnajc4	0	1.2	1.27	0.61	0
Dnajc5	0.85	NA	0.74	0	0
Dnajc5b	1.18	NA	0.42	3.3	0
Dnajc5g	NA	NA	0.57	8.68	NA
Dnajc6	0.37	0.74	0.45	NA	0
Dnajc7	0.65	2.1	2.33	0.25	2.41
Dnajc8	0.09	0.9	3.8	0.77	2.18
Dnajc9	1.35	1.45	2.01	0.63	0
Dnal1	NA	NA	0.74	0.39	NA
Dnal4	NA	0.78	2.78	0.19	NA
Dnali1	1.72	0.4	0.38	0.85	0
Dnase1	0	3.06	0.13	1.11	2.8
Dnase1l1	0.62	3.05	2.31	NA	0
Dnase1l2	0.79	2.85	1.64	2.97	1.17
Dnase1l3	0.12	0.02	0.92	4.32	2.43
Dnase2b	0.39	1.24	5.19	0	1.92
Dncl2a	1.99	NA	NA	NA	NA
Dnd1	4.16	0	0.98	0.69	0
Dner	0.53	NA	0.27	0.34	1.11
Dnhd1	NA	NA	0.9	NA	5.42
Dnlz	NA	NA	4.51	1.5	0
Dnm1	NA	1.21	1.97	0	0.55
Dnm1l	0.14	1.62	1.16	0.61	0
Dnm2	0.17	0.28	0.8	NA	0
Dnm3	2.07	1.56	0.76	0.95	1.26
Dnm3os	NA	NA	0.46	NA	NA
Dnmbp	0.58	0	1.21	1.08	0
Dnmt1	0.11	0	1.73	0	0
Dnmt3a	0	0.24	0.27	0	0.68
Dnmt3b	0	1.16	0.79	2.68	1.54
Dnmt3l	1	1.32	1.27	1.44	13.12
Dnpep	0	0.6	0.79	1.48	0
Dnph1	NA	0.55	1.96	1.78	NA

Dntt	1.32	0.6	0.51	1.3	1.99
Dnttip1	0.62	NA	1.03	0.96	2.44
Dnttip2	NA	0	3.62	0.02	0.6
Doc2a	1.14	1.78	0.73	NA	0
Doc2b	1.18	1.31	0.9	0	0
Dock1	1.29	0.94	0.89	0.81	3.18
Dock10	0.97	0	1.58	0.52	0
Dock11	0.04	NA	0.76	0.07	2.48
Dock2	0.49	0	0.97	0	1.04
Dock3	1.56	1.59	1.16	0	0
Dock4	0.71	0	2.33	0.4	1.34
Dock5	0	0.29	2	1.78	0.84
Dock6	NA	0.49	0.42	0.53	2.88
Dock7	3.48	NA	0.96	0	0
Dock8	0.51	NA	0.95	1.11	0
Dock9	2.28	0.43	0.84	0	0
Dohh	NA	2.03	2.41	1.24	0
Dok1	0.99	1.06	0.14	2.4	1.34
Dok2	NA	0.92	0.79	0.88	2.9
Dok3	0.56	0	1.89	2.45	2.92
Dok4	5.28	1.02	0.23	0.28	0
Dok5	1.1	1.32	2.28	1.02	0
Dok6	NA	NA	NA	4.62	NA
Dok7	NA	NA	0.12	4.41	0
Dolk	NA	1.39	0.24	3.19	1.43
Dolpp1	0	0	0.99	0	2.53
Dom3z	0.3	0.37	0.8	1.22	0.8
Donson	0.07	1.74	2.45	1.04	NA
Dopey1	NA	0	0.9	1.84	0
Dopey2	NA	0.75	0.94	0.14	0.98
Dot1l	0	1.5	1.31	1.44	0
Dpagt1	0.2	3.56	0.25	6.95	0
Dpcd	NA	NA	0.49	0	NA
Dpcr1	NA	NA	NA	0	NA
Dpep1	4.57	1.16	0.58	7.57	0
Dpep2	0.23	NA	NA	0.2	NA
Dpep3	1.2	2.09	4.56	2.04	4.43
Dpf1	NA	1.75	1.7	1.7	0
Dpf2	1.22	0	2.01	0.71	0
Dpf3	0.88	0.02	0	0.91	1.37
Dph2	NA	0.05	1.82	1.82	2.87

Dph3	NA	NA	1.4	1.04	2.6
Dph5	NA	1.01	3.49	0.98	2.18
Dpm1	0.05	1.61	3.01	0.88	0
Dpm2	0.55	2.55	1.92	0.81	1.77
Dpm3	0.48	2.92	2.55	0	0
Dpp10	0.83	NA	1.02	0.63	2.15
Dpp3	0.41	0.88	0.9	0	4.12
Dpp4	4.79	0	0.76	2.62	2.61
Dpp6	0.38	0	0.11	1.14	0
Dpp7	0.09	NA	0.44	0	0
Dpp8	0.71	1.2	0.95	0.48	1.74
Dpp9	0	NA	0	0.11	0.96
Dppa2	0	NA	0.88	0.52	1.5
Dppa3	3.16	NA	0.97	3.25	12.78
Dppa4	0.74	3.72	1.3	2.19	1.24
Dppa5	2.38	NA	NA	NA	NA
Dpt	0.94	1.39	1.57	0.82	0
Dpy19l1	NA	0.71	0	3.91	0
Dpy19l2	NA	NA	1.75	0.67	0
Dpy19l3	NA	NA	0.21	0	0
Dpy19l4	NA	0.87	2.14	0.67	0
Dpy30	NA	1.79	1.06	0	0
Dpyd	3.18	1.14	0.69	0	0.33
Dpys	3.31	1.52	0.36	0	1.41
Dpysl2	0	0	1.93	0.08	0
Dpysl3	0.76	0.72	1.01	0	1.35
Dpysl4	0.17	0.78	0.92	0	0
Dpysl5	NA	NA	0.73	0	8.06
Dqx1	0.61	NA	1.63	0	0
Dr1	0	1.16	1.14	5.6	1.5
Dram1	NA	0	0.85	1.27	0
Dram2	NA	NA	2.12	2.17	1.92
Drap1	0.83	2.09	0.6	0.13	1.79
Draxin	NA	NA	0.91	0.52	NA
Drc1	NA	NA	0	2.35	NA
Drd1ip	1.91	NA	NA	NA	NA
Drd2	0.52	1.08	1.24	0.6	10.39
Drd3	0	1.07	0.48	0.19	0
Drd4	0.4	2.88	0.02	1.44	0
Drd5	0	NA	NA	0.41	NA
Drg1	0.34	0.07	0.99	1.16	0.04

Drg2	0.05	1.61	0.1	1.08	0
Drosha	NA	NA	NA	2.89	NA
Drp2	3.36	0.81	0.29	0	0
Dsc1	0	2.43	0.74	4.2	9.53
Dsc2	0	1.16	1.91	1.63	0
Dsc3	4.36	0.64	2.1	0.89	0.23
Dscam	2.32	0.5	0	0.66	0
Dscaml1	0	NA	0.36	1.08	0
Dscc1	NA	0	0.63	0.65	2.38
Dscr1	0.62	NA	NA	NA	NA
Dscr1l1	0	NA	NA	NA	NA
Dscr1l2	2.45	NA	NA	NA	NA
Dscr2	0.01	NA	NA	NA	NA
Dscr3	0	0	3.14	1.09	0
Dscr6	2.4	NA	NA	NA	NA
Dse	NA	0.06	1.3	0.53	2.95
Dsel	NA	NA	1.66	0.37	0.03
Dsg2	0	1.66	0.93	0.24	1.16
Dsg3	1.89	NA	NA	0.75	NA
Dsg4	0.49	NA	NA	1.34	NA
Dsn1	NA	1.14	3.3	0.68	4.99
Dsp	0.4	0	0.01	0	0.99
Dspp	0	0.28	0.93	0.1	1.18
Dst	0.15	0.47	1.23	4.12	0
Dstn	0.5	1.84	2.3	1.2	3.48
Dstyk	NA	0.92	2.26	0	0
Dtd1	NA	NA	1.25	1.62	NA
Dtd2	NA	NA	1.45	0.72	NA
Dtl	NA	0	1.37	0.68	0
Dtna	1.51	0.55	0	2.03	1.43
Dtnb	1.66	1.93	0.88	5.62	0.38
Dtnbp1	0.55	NA	1.32	0.44	0
Dtwd1	NA	1.53	3.08	1.37	0
Dtwd2	NA	NA	2.1	8.37	2.19
Dtx1	0.15	NA	0.02	0.59	0.6
Dtx2	0	1.23	2.34	4.32	8.43
Dtx3	0.02	1.38	0.94	3.53	NA
Dtx3l	NA	NA	4.09	0	0
Dtx4	0.1	2.93	0.84	2.21	3.71
Dtymk	0.08	1.27	1.42	0	0.48
Dullard	0	NA	NA	NA	NA

Duox1	0.91	1.23	2.79	3.3	0
Duox2	0.1	NA	NA	0	NA
Duoxa1	NA	NA	0.48	NA	NA
Duoxa2	NA	NA	1.3	0	3.42
Dupd1	NA	NA	NA	0.54	NA
Dus1l	NA	2.71	1.98	1.49	0
Dus2l	NA	1.09	0.78	NA	0
Dus3l	NA	NA	1.33	1.49	1.78
Dus4l	NA	0	0.91	0.39	0
Dusp1	0	1.02	2.33	0	0
Dusp10	0	1.62	0.8	0	0.72
Dusp11	0.24	1.25	1.43	0	1.25
Dusp12	0	0.83	0.82	1.93	0
Dusp13	0.98	0	8.43	0.65	0
Dusp14	0	0	0	0.21	1.23
Dusp15	0.34	NA	1.48	1.07	0
Dusp16	0.01	NA	0.68	0	0.89
Dusp18	0	NA	1.94	NA	0
Dusp19	1.05	NA	0.41	0.76	NA
Dusp2	0.43	2.17	1.12	2.39	2.42
Dusp21	NA	0.53	1.08	0.44	3.57
Dusp22	4.63	3.42	2.71	1.29	3.75
Dusp23	0	NA	2.59	0.34	2.52
Dusp26	NA	2.76	2.73	0.94	0
Dusp27	NA	NA	0.49	0.95	3.06
Dusp28	NA	NA	1.3	0.97	0
Dusp3	0.38	2.97	1.49	1.06	2.22
Dusp4	0	1.19	2	2.19	0
Dusp5	NA	2.46	1.85	1.34	0
Dusp6	1.4	1.88	0.39	0	0
Dusp7	0.58	0	3.01	2.78	0
Dusp8	4.2	1.63	1.24	2.69	1.42
Dusp9	1.38	1.34	1.46	3.32	0
Dut	NA	1.17	1.01	0.65	2.29
Dvl1	1.54	0	1.22	1.21	2.19
Dvl2	0	0	0.79	1.29	0
Dvl3	0.88	1.44	1.03	1.02	0
Dvwa	NA	NA	NA	NA	0.22
Dydc1	NA	NA	2.29	0.54	NA
Dydc2	NA	NA	2.72	NA	4.52
Dym	NA	1.16	1.32	0	1.39

Dync1h1	NA	0	0.55	0	0.33
Dync1i1	NA	2.52	1.55	0.46	0
Dync1i2	NA	0.21	3.11	0.17	1.5
Dync1li1	NA	0.97	1.45	0.57	6.55
Dync1li2	NA	0.55	1.19	0	0
Dync2h1	NA	2.7	1.32	0.4	0
Dync2li1	NA	0	1.4	4.32	NA
Dynll1	NA	1.39	1.85	1.36	0
Dynll2	NA	NA	1.03	0.71	2.19
Dynlrb1	NA	0.98	1.41	0.15	1.63
Dynlrb2	NA	NA	0.2	1.63	0
Dynlt3	NA	2	2.69	1.07	2.78
Dyrk1a	0	0.4	0.3	1.22	0
Dyrk1b	0	0	1.91	0.25	2.98
Dyrk2	NA	0	1.03	1.82	NA
Dyrk3	0	1.36	0.04	3.34	0
Dyrk4	0	NA	NA	0.94	NA
Dysf	3.48	0	0.49	1.96	7.33
Dyx1c1	1.08	NA	0.01	1.66	0
Dzank1	NA	4.1	0.34	0.84	NA
Dzip1	NA	1.04	0.54	0	0.73
Dzip1l	NA	NA	4.2	1.64	0
Dzip3	NA	0	0.62	0.78	0
E2f1	0.37	0.96	2.47	6.48	2.24
E2f2	0.25	1.38	2.38	1.63	2.5
E2f3	0.27	0	1.36	2.12	0
E2f4	NA	0.1	0.14	2.63	3.63
E2f5	0	0.26	1.31	0.48	0
E2f6	0	0.79	1.37	0.6	1.04
E2f7	1.65	NA	1.81	0.24	1.32
E2f8	NA	0.08	0.8	1.26	0.81
E4f1	0	0.13	2.53	1.38	22.35
Eaf1	0	NA	2.21	0	0.52
Eaf2	1.36	2.57	0.91	1.52	2.59
Eapp	NA	0.1	0.85	0.76	2.73
Ears2	NA	NA	0.02	1.92	0
Ebag9	0.23	2.34	0.21	0.93	7.46
Ebf1	1.17	NA	0.96	0	0.59
Ebf2	0.83	2.17	0.27	0.9	0
Ebf3	10.44	NA	0.09	0.81	1.19
Ebf4	NA	NA	0.41	3.35	2.42

Ebi2	1.13	NA	NA	NA	NA
Ebi3	0.36	0.48	4.13	1.83	0
Ebna1bp2	0.09	1.22	0.67	NA	2.42
Ebp	0.12	1.52	2.22	0.94	0
Ebpl	0.35	NA	0.24	1.98	4.76
Ecd	NA	0.57	0.83	0.44	0.33
Ece1	0.02	1.78	0.97	5.9	4.19
Ece2	0.93	0.32	0	2.19	1.92
Ecel1	0.36	1.37	1.49	1.32	0
Ecgf1	1.58	NA	NA	NA	NA
Ech1	0.2	1.18	0.39	4.12	6.8
Echdc1	0.17	0.65	1.63	1.6	0
Echdc2	NA	0	1.21	0.7	1.43
Echdc3	6.45	2.39	2.29	1.2	0.77
Echs1	0.43	2.84	3.12	0.35	0
Eci1	NA	1.74	1.62	0.72	NA
Eci2	NA	2.17	1.35	NA	NA
Ecm1	1.51	2.84	1.62	1.49	3.48
Ecm2	NA	0.32	1.08	0.06	3.44
Ecscr	NA	NA	2.49	NA	0
Ecsit	NA	0.92	2.16	0.74	0
Ect2	0.54	0	2.05	0	4.11
Eda	0.3	0	0.27	0.33	2.55
Eda2r	1.94	2.42	3.28	1.05	1.82
Edar	0	0	0.94	0.3	1.4
Edaradd	0.24	NA	0	2.44	NA
Edc3	NA	2.35	4.49	0	1.94
Edc4	NA	0	2.73	0	5.5
Eddm3b	NA	NA	NA	0.25	NA
Edem1	0.48	0	2.26	0.55	3.22
Edem2	NA	1.37	0.26	3.77	0
Edem3	NA	1.28	0.45	0.17	0.43
Edf1	1.64	2.37	0.65	0.33	2.76
Edg1	0	NA	NA	NA	NA
Edg2	1.05	NA	NA	NA	NA
Edg3	0.11	NA	NA	NA	NA
Edg4	0	NA	NA	NA	NA
Edg5	1.45	NA	NA	NA	NA
Edg6	3.72	NA	NA	NA	NA
Edg7	8.28	NA	NA	NA	NA
Edg8	3.73	NA	NA	NA	NA

Edil3	0	0.5	1.56	8.35	0
Edn1	0.25	0.31	2.33	0.82	0
Edn2	0.82	1.83	0.11	3.6	1.6
Edn3	0.95	1.05	2.19	1.6	0
Ednra	0.07	0.32	0.41	1.24	0
Ednrb	0.7	3.59	0.02	0.29	2.03
Eea1	0.62	1	2.9	1.63	2.65
Eed	0.82	0	0.33	2.98	2.5
Eef1a1	0.76	1.65	1.65	3.64	6.13
Eef1a2	1.06	0.83	1	1.84	2.24
Eef1b2	0.25	0.72	5.01	0.13	0
Eef1d	0.96	0.7	0	0.18	0.21
Eef1e1	0.32	0.9	2.14	0.7	0
Eef1g	NA	0	0.77	1.09	NA
Eef2	0.43	0	0.95	1.13	0
Eef2k	0	NA	1.15	0	0
Eefsec	0.53	NA	1.96	0	0
Eepd1	NA	NA	0	2.78	0.71
Efcab1	NA	1.03	0.83	1.69	1.69
Efcab10	NA	NA	0	0.35	NA
Efcab11	NA	0	1.16	0.83	NA
Efcab12	NA	NA	3.26	0.17	NA
Efcab14	NA	0	0.91	0.52	NA
Efcab2	NA	1.8	0	0.71	0
Efcab3	NA	NA	1.57	4.79	NA
Efcab4a	NA	NA	0.61	0.35	3.5
Efcab4b	NA	NA	NA	2.62	NA
Efcab5	NA	NA	NA	2.66	NA
Efcab6	NA	2.19	0.83	3.07	18.06
Efcab7	NA	NA	0.02	2.18	0.92
Efcbp1	0	NA	NA	NA	NA
Efcbp2	0.49	NA	NA	NA	NA
Efcc1	NA	1.71	0.45	1.43	NA
Efemp1	0.96	0.73	2.54	1.2	1.36
Efemp2	0	0	0.48	0.03	0.93
Efha1	NA	NA	NA	NA	0
Efha2	NA	NA	NA	NA	1.02
Efhb	NA	NA	1.75	2.03	1.54
Efhc1	1.04	0	0.41	14.64	0
Efhc2	0	1.2	1.21	1.47	0
Efhd1	0	2.19	0.45	2.31	2.69

Efhd2	0.04	3.23	1.39	5.8	0
Efna1	0.95	0.47	2.53	0.8	0
Efna2	1.4	1.15	0.71	0.57	0
Efna3	2.63	0.79	0.57	0.31	0
Efna4	0.59	1.61	1.33	4.27	3.75
Efna5	0	1.07	0.15	1.14	1.61
Efnb1	0	0.71	0.44	2.42	2.85
Efnb2	1.14	1.2	0.02	1.08	1.63
Efnb3	1.8	1.54	0.31	1.51	2.08
Efr3a	NA	1.78	1.41	1.42	0
Efr3b	NA	0.9	1.11	0	1.32
Efs	4.54	0.09	1.83	1.47	0
Eftud1	3.51	1.39	0.54	0.56	3.43
Eftud2	NA	NA	0.62	0.78	0
Egf	1.66	1.27	10.5	1.8	4.24
Egfl6	2.19	0.42	0.92	1.08	0
Egfl7	0.06	1.46	0	0.68	0
Egfl8	0.25	1.17	1.98	NA	NA
Egfl9	2.78	NA	NA	NA	NA
Egflam	NA	NA	0.04	1.28	2.34
Egfr	0.14	0.66	1.01	11.85	0
Egln1	0.43	1.84	1.3	0.18	0.32
Egln2	0.88	2.58	0.43	1.6	0.05
Egln3	0.38	1.15	1.08	0	0
Egr1	0	1.46	3.41	0.25	4.16
Egr2	0.64	1.44	1.54	0	0
Egr3	0.84	1.05	0.77	0.53	0
Egr4	1.27	2.22	0	1	0
Ehbp1	0	0.04	0.65	1.65	1.12
Ehbp1l1	NA	0.32	5.15	3.34	7.36
Ehd1	0.22	0.59	1.87	3.01	0
Ehd2	0	0.32	1.62	0	1.33
Ehd3	0.2	0.3	2.68	0	2.13
Ehd4	0.85	0	0.42	0.73	1.78
Ehf	0.03	0.22	0	2.06	0
Ehhadh	0.88	2.37	2.15	2.57	0.35
Ehmt1	0.71	1.22	2.02	2.81	0
Ehmt2	NA	0.02	1.27	0.57	0.12
Ei24	0.1	0	2.53	1.09	0
Eid1	NA	0.86	2.51	2.68	0
Eid2	NA	NA	1.5	1	2.01

Eid2b	NA	NA	NA	0.17	NA
Eid3	NA	NA	0.57	0.95	4.85
Eif1	NA	0.38	1.92	0	0.84
Eif1ad	NA	NA	2.83	1.29	1.03
Eif1ax	NA	1.44	1.6	0.84	0
Eif1ay	1.21	NA	NA	NA	NA
Eif1b	NA	1.61	0.13	0.87	3.21
Eif2a	NA	NA	2.17	1.05	0
Eif2ak1	0	1.35	0.89	0.78	0.5
Eif2ak2	NA	0.44	0.43	0	0
Eif2ak3	0.67	0	1.55	0	0.64
Eif2ak4	0.5	NA	0.82	1.06	0.91
Eif2b1	0.45	0	1.48	0.89	30.78
Eif2b2	1.85	0	2.12	1.21	0
Eif2b3	0.13	1.28	0.27	1.05	0.83
Eif2b4	0	0.66	0	1.69	0
Eif2b5	0	0	1.32	0.24	4.51
Eif2c1	0	NA	NA	NA	0
Eif2c2	1.13	NA	NA	NA	0
Eif2c3	0	NA	NA	NA	1.52
Eif2c4	0.68	NA	NA	NA	0.33
Eif2d	NA	0	0.98	0	NA
Eif2s1	0.53	0.62	0.67	0	1.75
Eif2s2	0.13	0.79	1.72	0	0.62
Eif3a	NA	2.01	0.36	0	0
Eif3b	NA	1.72	1.21	0.06	1.82
Eif3c	NA	NA	1.98	NA	0
Eif3d	NA	1	1.9	0	0
Eif3e	NA	1.05	2.53	2.38	3.99
Eif3f	NA	0.14	1.02	0	0.01
Eif3g	NA	2.3	0.68	1.38	0
Eif3h	NA	0	0.88	0	3.06
Eif3i	NA	1.84	0.52	1.87	0
Eif3j	NA	NA	NA	NA	0
Eif3k	NA	0	1.37	0	0
Eif3l	NA	1.3	0.3	0.85	0
Eif3m	NA	0.76	0.32	0.85	0
Eif3s1	0.2	NA	NA	NA	NA
Eif3s10	0.21	NA	NA	NA	NA
Eif3s2	0	NA	NA	NA	NA
Eif3s3	0.13	NA	NA	NA	NA

Eif3s4	0	NA	NA	NA	NA
Eif3s5	0.19	NA	NA	NA	NA
Eif3s6	0.87	NA	NA	NA	NA
Eif3s6ip	0.18	NA	NA	NA	NA
Eif3s7	0.91	NA	NA	NA	NA
Eif3s9	0.14	NA	NA	NA	NA
Eif4a1	0.37	2.1	0.47	1.12	0
Eif4a2	0	0	2.6	1.08	0
Eif4a3	NA	0.69	0.86	1.53	0
Eif4b	0.01	0	1.42	0	0
Eif4e	0.29	0.64	1.38	2.21	2.01
Eif4e1b	NA	NA	NA	1.58	NA
Eif4e2	NA	0.51	1.66	0.9	0
Eif4e3	NA	NA	1.68	0.93	0
Eif4ebp1	1.77	0.98	0.41	0.19	0
Eif4ebp2	0.57	0.42	1.28	2.97	0.7
Eif4ebp3	0.18	NA	NA	NA	NA
Eif4enif1	0.77	0.96	1.93	1.68	1.25
Eif4g1	0.1	0	0.06	1.83	1.59
Eif4g2	0.29	0.35	0.24	0	0.76
Eif4g3	1.29	0.77	0.74	1.24	0
Eif4h	NA	1.45	0.76	0.04	0
Eif5	0	1.29	1.75	0.36	0
Eif5a	0.4	1.62	0.38	1.4	1.17
Eif5a2	1.75	3.09	2.25	1.42	1.39
Eif5b	0.1	0.65	0.06	0.95	0
Eif6	NA	1.25	2.13	0	0.12
Ela1	2.18	NA	NA	NA	NA
Ela2	0	NA	NA	NA	NA
Ela3b	0.65	NA	NA	NA	NA
Elac1	0	1.14	1.55	0	1.11
Elac2	0.1	0.59	0.93	2.58	0.71
Elane	NA	0	1.09	0.83	0.65
Elavl1	0	0	0.03	0	0
Elavl2	1.19	0.35	0.84	0.72	1.74
Elavl3	0.57	2.14	0.03	1.83	2.03
Elavl4	0.71	1.5	1.58	1.41	0.82
Elf1	0.7	1.08	0.87	1.36	0.28
Elf2	0	0.53	0.49	0.25	0
Elf3	0.62	0.89	1.36	0.74	3.81
Elf4	0.54	1.2	0.02	0.99	0

Elf5	0.85	0.54	2.04	0.46	0
Elfn1	NA	NA	0.79	0.42	14.36
Elfn2	NA	NA	2.51	0	NA
Elk1	0	0.75	0.55	3.29	0
Elk3	0.2	0.03	4.92	NA	0
Elk4	0.96	0.82	0.37	0	1.91
Ell	0.83	0.81	1.68	3.24	0
Ell2	0	0.71	1.23	0.15	1.33
Ell3	0.78	NA	NA	0.43	NA
Elmo1	0.32	0	1.8	1.18	0
Elmo2	0.81	0.28	0.64	0.52	4.25
Elmo3	0.56	3.54	1.55	1.06	0
Elmod1	NA	NA	2.95	2.17	2.61
Elmod2	2.4	NA	0.84	0.39	0
Elmod3	NA	NA	2.89	0.56	NA
Elmsan1	NA	NA	0.33	0.62	NA
Eln	1.65	0.91	0.21	1.12	3.31
Elof1	NA	NA	5.49	3.16	0
Elovl1	0.03	1.41	1.46	5.41	3.08
Elovl2	0.08	1.78	2.29	0.87	1.43
Elovl3	0.2	NA	1.19	3.46	1.18
Elovl4	6.29	0	1.89	0.71	1.92
Elovl5	0.98	1.03	1.02	0.16	0.99
Elovl6	1.34	0.87	1.42	0.28	2.45
Elovl7	2.55	NA	0.86	0.72	0.2
Elp2	NA	NA	0.5	0	3.32
Elp3	0.21	0	0.38	0.5	1.88
Elp4	0	0.96	1.02	0.73	1.05
Elp5	NA	1.86	0	0.92	NA
Elp6	NA	0	0.33	0.85	NA
Eltd1	NA	1.78	0.05	2.46	1.79
Emb	0.87	NA	0.01	1.2	0
Emc1	NA	0	0.55	0	NA
Emc10	NA	NA	0.55	0.23	NA
Emc2	NA	0	1.27	1.06	NA
Emc3	NA	0.79	1.42	0	NA
Emc4	NA	NA	3.97	0.65	NA
Emc6	NA	1.33	1.47	1.03	NA
Emc7	NA	0.74	1.61	0.73	NA
Emc8	NA	0.94	1.23	2.23	NA
Emc9	NA	2.2	1.39	1.57	NA

Emcn	1.8	0.75	0.92	2.32	2.01
Emd	0.82	3.8	2.67	0.95	0
Eme1	NA	NA	0.73	0.95	0
Eme2	2.18	NA	1	0.25	NA
Emg1	NA	0.06	1.49	NA	2.01
Emid1	0.32	2.58	0	1.83	1.58
Emid2	NA	NA	NA	NA	1.59
Emilin1	0.17	1.74	1.79	0.89	0
Emilin2	0.74	0	1.57	0.95	0.74
Emilin3	2.43	NA	1.38	0.65	0
Eml1	0	2.24	1.04	3.73	NA
Eml2	0.48	0.72	2.6	0	0
Eml3	NA	0.15	1.61	2.16	0
Eml4	2.12	0	1.34	1.47	0.51
Eml5	0.51	NA	0.76	2	1.31
Eml6	NA	NA	1.29	0.27	0.7
Emp1	0.06	0	0	0.78	1.59
Emp2	0.58	0.67	1.46	0	0
Emp3	0.27	3.41	0.48	0	0.39
Emr1	0	4.37	3.39	1.2	3.36
Emr4	0.78	NA	NA	NA	NA
Emx1	0	1.96	2.21	0.09	0
Emx2	1.71	0.03	2.1	0	2.33
Emx2os	NA	NA	0	NA	0
En1	0.7	1.91	0	1.28	0
En2	0.22	1.48	0.6	2.4	4.54
Enah	0.27	0.88	0.9	1.61	0
Enam	0	NA	1.4	1.13	0.7
Enc1	0	1.22	7.49	0.96	0.04
Endod1	NA	0.93	0.55	0	0.61
Endog	0	0.11	0.8	NA	0
Endogl1	0.06	NA	NA	NA	NA
Endou	NA	1.4	1.05	1.92	NA
Endov	NA	NA	1.13	1.71	NA
Eng	1.3	0.75	0.48	1.49	0.89
Engase	NA	0	1.63	1.21	0
Enho	NA	NA	0.87	0.92	0
Enkd1	NA	NA	0.48	1.45	NA
Enkur	NA	NA	3.55	3.31	0
Eno1	0.33	1	0	0	0.42
Eno2	0.49	3.63	0	0.4	0

Eno3	0	1.07	0	2.14	1.98
Eno4	NA	NA	NA	0.9	NA
Enoph1	NA	3.06	1.83	0.14	0
Enox1	NA	0.82	0.58	0	0
Enox2	NA	0	1.55	1.18	0
Enpep	0	2.09	0.29	1.03	1.44
Enpp1	0.3	2.17	2.88	1.5	0.26
Enpp2	0.76	0	1.16	0	1.63
Enpp3	0	1.02	0.46	0.87	0.17
Enpp4	1.3	0.54	0.4	0.06	2.49
Enpp5	1.34	NA	0.25	1.86	2.02
Enpp6	0.9	NA	2.49	0.99	0.17
Enpp7	NA	NA	NA	1.32	NA
Ensa	0.74	0	0.89	0.42	0
Enthd1	NA	NA	2.21	0.8	NA
Enthd2	NA	NA	1.54	1.26	NA
Entpd1	0.72	0.01	2.86	1.21	0
Entpd2	1.35	2.53	1.71	0.37	2.94
Entpd3	1.03	3.93	2.7	4.02	2.24
Entpd4	0.85	0.46	3.09	3.78	0
Entpd5	0.79	0.03	0.98	1.36	0
Entpd6	0.82	0	0.98	0.75	0.1
Entpd7	1.31	0	0.21	0	2.49
Entpd8	1.38	NA	NA	1.01	NA
Eny2	NA	0	0	0	1.55
Eogt	NA	1.14	1.42	0.27	NA
Eomes	0.39	NA	0.74	1.95	0
Ep300	0.89	1.76	1.81	1.42	0
Ep400	0.4	0	1.08	1.52	0.92
Epas1	0	0.7	1.16	0	2.21
Epc1	0.84	NA	0.7	0	1.43
Epc2	1.09	NA	1.73	0.81	2.88
Epcam	NA	1.02	0.43	0.52	2.38
Epdr1	NA	NA	3.55	0.85	1.31
Epg5	NA	NA	NA	0	NA
Epgn	2.09	NA	NA	0.45	NA
Epha1	1.25	0	0.49	0.6	0.91
Epha10	NA	NA	1.99	0.61	0
Epha2	0	1.96	0	0	0
Epha3	0.65	0.58	0.39	1.55	1.78
Epha4	0.61	1.44	0.66	0.36	0

Epha5	0.24	1.12	0.52	0.64	0
Epha6	0.7	NA	2.6	0.06	3.76
Epha7	0.83	0.01	0.34	0	1.79
Epha8	0.65	NA	0	0.52	2.58
Ephb1	0.72	0	0	2.48	1.44
Ephb2	0.52	0.3	0.54	0	0
Ephb3	0	0.93	2.38	0.68	2.82
Ephb4	0.95	1.52	1.17	1.89	5.33
Ephb6	0.9	0	3.55	0.94	0
Ephx1	0	0.21	0.22	0.51	0.87
Ephx2	3.41	0	0.81	0.77	0.76
Ephx3	NA	1.32	3.42	1.11	0.98
Ephx4	NA	NA	0.25	2.05	NA
Epm2a	0	0.04	2.53	1.56	1
Epm2aip1	8.63	1.3	1.19	0.83	0.52
Epn1	0	2.23	0.96	0.2	1
Epn2	0	0.55	0.85	7.87	0.75
Epn3	1.02	0.63	1.1	1.74	0
Epo	0.74	NA	NA	1.96	NA
Epor	1.03	0	0.55	1.54	0.79
Eppb9	0	NA	NA	NA	NA
Eppin	NA	1.92	1.7	0.79	NA
Eppk1	0	1.54	0.15	0.68	0
Eprs	2.38	0	0.99	0	3.01
Eps15	0	0.18	2.6	1.06	2.98
Eps15l1	NA	0.55	0.9	0.04	0
Eps8	0	1.3	5.04	1.13	0
Eps8l1	0.31	1.45	0	0.57	0
Eps8l2	0.91	3.94	0.72	NA	2.31
Eps8l3	2.98	1.47	0.97	NA	0.64
Epsti1	NA	NA	0	10.64	0.43
Ept1	NA	NA	0.64	NA	NA
Epx	0.38	1.09	1.29	0.49	0
Epyc	NA	2.06	1.02	0.39	2.09
Eqtn	NA	NA	0.34	0	NA
Eraf	3.47	NA	NA	NA	NA
Eral1	0	0.21	0.79	0.64	0.44
Erap1	NA	0.53	1.61	3.9	1.93
Eras	0.31	NA	NA	1.54	NA
Erbb2	0.77	0	2.16	0	0
Erbb2ip	0.91	1.09	1.22	1	3.37

Erbb3	0.01	0.77	1.6	1.4	1
Erbb4	1.3	2	0	0	6.13
Erc1	NA	0.99	0.27	0.51	0.26
Erc2	NA	0.73	0.81	0.86	1.44
Ercc1	0	1.02	1.33	NA	0
Ercc2	0.8	0	1.96	1.84	1.55
Ercc3	2.17	1.08	1.26	0.8	3.14
Ercc4	0.12	0.35	0.61	0.44	0
Ercc5	0	0	1.54	2.06	2.16
Ercc6	0.18	NA	NA	NA	0
Ercc6l	NA	0.41	1.57	1.17	0
Ercc6l2	NA	NA	0.42	0.37	NA
Ercc8	NA	1.12	0	1.38	0.44
Ereg	0	0.47	0.17	0.31	1.95
Erf	0	0	0.14	1.12	0.73
Erg	0.18	0	0.59	0.92	0
Ergic1	NA	NA	1.98	0	1.1
Ergic2	NA	0	0.51	0.04	0
Ergic3	NA	0.33	0.26	0.89	1.83
Erh	0	0.56	1.49	0	0
Eri1	NA	NA	0	0.38	0
Eri2	NA	1.62	0.77	0.37	0.17
Eri3	NA	1.79	2.61	0.08	1.13
Erich1	NA	NA	NA	1.43	NA
Erlec1	NA	NA	2.35	1.08	0
Erlin1	NA	0	2.02	1.1	3.94
Erlin2	NA	1.03	1.38	0.46	0
Ermap	0.16	1.28	3.29	2.33	0
Ermn	NA	NA	0.92	0.07	0
Ermp1	NA	0	0.23	0	0
Ern1	0.31	0.6	0.65	0.86	0
Ern2	0	2.25	1.29	0.14	5.24
Ero1l	0	0	2.86	1.39	1.7
Ero1lb	NA	2.62	0.58	0.67	0.69
Erp27	NA	NA	0.14	2.01	1.71
Erp29	NA	0.8	0.36	0.9	0
Erp44	NA	0.78	2.04	0.94	0.18
Errfi1	NA	NA	1.98	0.25	0.23
Esam	NA	NA	0.95	0.36	0
Esco1	NA	NA	1.3	0	0
Esco2	NA	NA	1.75	0.35	1.15

Esd	0.93	0.57	3.02	4.22	0.92
Esf1	NA	1.66	5.07	2.52	2.03
Esm1	0.85	0.18	0.33	1.19	1.49
Espl1	0.37	2.38	2.41	1.83	1.81
Espn	0	0.27	1.26	1.49	0
Espnl	NA	NA	NA	1.09	NA
Esr1	0.99	0.19	1.62	0.11	1
Esr2	0	0.99	0.49	0	0
Esrp1	NA	0.74	0.57	2.7	0.76
Esrp2	NA	1.61	0.26	0.61	2.12
Esrra	0.7	1.05	0.66	1.94	0.99
Esrrb	0.44	1.45	0.44	5.27	0
Esrrg	0.74	0.64	0.21	0.59	0
Esx1	4.33	NA	2.46	1.93	NA
Esyt1	NA	0	0	1.31	0
Esyt2	NA	NA	1.96	0	2.26
Esyt3	NA	NA	2.52	4.09	1.82
Etaa1	NA	2.9	1.95	0.6	0
Etf1	0.6	0.25	1.52	0.9	0
Etfa	0	2.69	2.21	1.93	0
Etfb	0.36	0.89	1.59	0.04	2.33
Etfdh	1.59	0.54	0.51	1.75	0.03
Ethe1	1.51	0	1.12	0	0
Etnk1	0.06	1.49	0.45	0	0
Etnk2	0.55	0.34	1.43	1.02	0
Ets1	0	0.51	1.93	1.99	0.71
Ets2	0.18	0.22	1.15	5.52	0
Etv1	1.23	0.76	0.63	0.89	2.16
Etv2	NA	3.52	1.96	2.24	0
Etv3	0.37	0.66	3.57	0.6	1.17
Etv3l	NA	NA	NA	0	NA
Etv4	3.05	0	0	3.48	4.61
Etv5	0.76	0.57	1.75	1.71	2.44
Etv6	0.79	0.17	0.1	0.38	0.44
Eva1	1.07	NA	NA	NA	NA
Eva1a	NA	NA	2.86	1.22	NA
Eva1b	NA	1.35	2.76	NA	NA
Eva1c	NA	NA	0	2.18	NA
Evc	0.63	1.3	0.78	5.08	0
Evc2	2.1	NA	1.34	2.5	3.57
Evi1	0	NA	NA	NA	NA

Evi2a	0.05	0	1.77	NA	0
Evi2b	3.27	1.09	1.61	NA	3.02
Evi5	0	0.85	1.48	1.08	2.49
Evi5l	NA	NA	2.74	0	0
Evl	1.06	0	0.08	2.04	0
Evpl	2.33	0.63	0.96	1.26	5.44
Evx1	2.55	0.79	1.47	0.54	0.53
Evx2	0.55	NA	NA	1.33	NA
Ewsr1	NA	0.68	1.18	2.29	0.89
Exd1	NA	NA	0.83	0.96	NA
Exd2	NA	0	0.36	0.78	1.42
Exo1	0	0.45	1.41	1.15	0.09
Exo5	NA	2	1.17	0.96	NA
Exoc1	NA	0.55	0.68	0.22	1.22
Exoc2	NA	0.17	2.31	0.78	4.39
Exoc3	NA	0.75	1.54	NA	1.68
Exoc3l2	NA	NA	1.29	NA	NA
Exoc3l4	NA	NA	1.77	NA	NA
Exoc4	NA	NA	1.93	0.5	0.66
Exoc5	NA	1.1	1.02	0.28	1.39
Exoc6	NA	NA	0.58	0.11	0
Exoc6b	NA	0.21	1.14	0.76	0.76
Exoc7	1.52	1.36	1.11	0	0.15
Exoc8	1.04	NA	0.3	0	2.73
Exog	NA	0.14	1.49	0.94	1.18
Exosc1	1.88	0.91	1.67	0.5	0
Exosc10	0.32	0	0.76	2.88	0
Exosc2	0.24	0.6	1.19	1.3	1.66
Exosc3	0	NA	2.61	0.28	0
Exosc4	0.52	0	0.48	1.68	4.19
Exosc5	0.23	0.78	0.23	2.78	0.01
Exosc6	0	NA	1.17	NA	2.19
Exosc7	1.14	1.09	0.39	2.65	2.27
Exosc8	0.43	0	0.88	0.98	0
Exosc9	3.16	1.93	2.93	0.42	0.7
Exph5	NA	0	0.38	0.73	2.43
Ext1	0.33	0	0.15	1.48	0
Ext2	0.79	0	0.63	0	2.81
Extl1	0	2.84	0.73	0.18	2.64
Extl2	0.26	1.69	0.76	0.03	2.85
Extl3	1.1	1.72	0.53	1.67	1.44

Eya1	0.43	1.27	1.87	0.62	1.57
Eya2	1.16	2.16	1.72	1.99	2.03
Eya3	0	1.29	0.86	1.4	0
Eya4	0.76	0.81	0.45	1.36	1.68
Ezh1	1.05	0.13	1.4	0.27	0.34
Ezh2	0.31	0.38	2.23	0.19	0
Ezr	NA	0	0.89	0	0.85
F10	0.97	0.39	0.9	1.13	0
F11	0.5	0.86	1.3	0	0
F11r	0	0.86	0.62	NA	0.51
F12	0	2.02	0.06	0.7	2.97
F13a1	0.77	1.96	2.01	5.09	0.49
F13b	1.55	2	0.31	0.07	1.04
F2	2.04	2.7	0.76	1.97	5.75
F2r	0.48	0.13	1.3	3.95	1.63
F2rl1	1.85	1.86	0.59	1.85	1.34
F2rl2	1.95	1.05	0.63	1.63	0.61
F2rl3	0	2	2.66	0.7	0
F3	0.55	1.27	2.3	5.13	0.87
F5	0.24	4.24	2.37	1.71	0
F7	0.79	2.55	3.72	3	0
F8	0.61	3.42	1.38	2.9	0.86
F9	3.72	2.25	1.26	1.09	2.83
Fa2h	0.91	2.73	2.39	0.13	1.82
Faah	0.8	1.12	0.21	1.96	0.63
Fabp1	3.57	2.88	0.94	NA	12.99
Fabp12	NA	NA	NA	6.78	NA
Fabp2	2.32	2.58	1.15	3.74	1.45
Fabp3	1.73	0.94	2.05	0	3.53
Fabp4	0.84	1.33	1.66	4.33	2.62
Fabp5	NA	0.27	3.69	NA	3.8
Fabp6	1.05	1.2	3.23	2.02	0
Fabp7	0	1.5	0.5	0	4.84
Fadd	1.64	1.07	0.92	5.26	2.99
Fads1	0	1.01	3.06	0	0
Fads2	1.27	2.64	1.53	0.4	0.53
Fads3	0.19	1.14	0.28	0.73	0
Fads6	NA	NA	NA	0.67	NA
Faf1	0	0	0.73	0.99	1.47
Faf2	NA	0.79	1.64	0.53	1.93
Fah	0	1.99	0.59	0.48	0

Fahd1	0.86	NA	2.03	0.5	0
Fahd2a	NA	0	0.91	0.71	0
Faim	0.45	2	3.01	0.74	0
Faim2	2.33	0.32	0.9	0.47	0
Faim3	NA	0	0.4	0.71	0
Fam100a	NA	NA	NA	NA	3.73
Fam100b	NA	NA	NA	NA	0
Fam101a	NA	NA	0	2.33	4.39
Fam101b	NA	NA	0.61	2.26	0.17
Fam102a	NA	0	1.33	0.95	1.98
Fam102b	NA	NA	2.21	0	0
Fam103a1	NA	NA	0.71	NA	1.6
Fam104a	NA	NA	1.36	0	NA
Fam105a	NA	1.92	2.53	1.68	1.32
Fam105b	NA	NA	0.49	0.75	1.16
Fam107a	NA	0	0.46	0.79	0
Fam107b	NA	NA	0.48	4.83	0.11
Fam109a	NA	NA	1.61	2.12	1.81
Fam109b	NA	NA	2.78	0.39	3.54
Fam110a	NA	NA	4.16	3.79	1.78
Fam110b	NA	1.96	1.23	0	0
Fam110c	NA	NA	2.25	1.24	0
Fam111a	NA	1.03	1.83	1.92	0.06
Fam113a	NA	NA	NA	NA	1.92
Fam113b	NA	NA	NA	NA	0
Fam114a1	NA	0.16	0.89	2.38	1.12
Fam114a2	NA	3.1	1.92	0.35	0
Fam115a	NA	1.5	0.92	2.7	0.57
Fam115c	NA	NA	2.12	0.16	5.26
Fam116a	NA	NA	NA	NA	0
Fam116b	NA	NA	NA	NA	0
Fam117a	NA	0.02	1	0	0
Fam117b	NA	NA	0.74	1.64	0
Fam118a	NA	0.85	1.47	0.9	0
Fam118b	NA	NA	0.32	1.98	0
Fam119a	NA	NA	NA	NA	0
Fam120a	NA	0.46	0.48	0.22	0.89
Fam120aos	NA	NA	0.94	NA	NA
Fam120b	NA	NA	1.79	2.5	1.41
Fam120c	NA	1.42	0.6	1.89	1.28
Fam122a	NA	NA	0	0	3.87

Fam122b	NA	NA	0.91	0.74	4.15
Fam122c	NA	NA	1.54	0	0
Fam123a	NA	NA	NA	NA	3.66
Fam123c	NA	NA	NA	NA	0
Fam124a	NA	NA	2.87	NA	NA
Fam124b	NA	2.34	0	0	0
Fam125a	NA	NA	NA	NA	0
Fam125b	NA	NA	NA	NA	0
Fam126a	NA	NA	1.45	1.19	0
Fam126b	NA	NA	1.34	1.22	0
Fam128b	NA	NA	NA	NA	0
Fam129a	NA	0.45	0.92	0	0
Fam129b	NA	NA	0.83	0	3.81
Fam129c	NA	NA	1.94	0.27	0
Fam131a	NA	0.2	1.73	0.5	0
Fam131b	NA	1.05	2.16	1.62	1.82
Fam131c	NA	NA	0.96	1.1	0
Fam132a	NA	NA	1.93	1.76	1.28
Fam132b	NA	NA	NA	0.13	NA
Fam133b	NA	NA	NA	NA	0
Fam134a	NA	0.41	0.94	1.06	0
Fam134b	NA	0.98	0.98	0.72	0.15
Fam134c	NA	0	0.49	0	0.01
Fam135a	NA	1.35	0.74	1.16	0
Fam135b	NA	NA	0.25	0.78	1.76
Fam136a	NA	1.33	2.92	1.37	2.43
Fam13a	NA	0.94	1.31	0.28	1.44
Fam13b	NA	1.59	0.19	0.43	2.98
Fam13c	NA	0.83	0.67	0.69	0
Fam149a	NA	1.25	0.97	2.61	0
Fam150a	NA	NA	NA	1.31	NA
Fam150b	NA	NA	NA	0	NA
Fam151a	NA	NA	0	NA	NA
Fam151b	NA	NA	NA	1.56	NA
Fam154a	NA	NA	1.18	1.16	0.5
Fam154b	NA	NA	0	0	NA
Fam155a	NA	0.01	0.59	NA	6.65
Fam158a	NA	NA	NA	NA	6
Fam159a	NA	NA	NA	1.12	NA
Fam159b	NA	NA	NA	1.08	NA
Fam160a1	NA	NA	0.85	0.48	2.21

Fam160a2	NA	NA	0.62	1.54	0
Fam160b1	NA	NA	1.14	0	1.73
Fam160b2	NA	0	2.33	0	0.79
Fam161a	NA	NA	0.55	0.15	1.61
Fam161b	NA	NA	NA	0.83	NA
Fam162a	NA	0.86	0.38	2.44	0
Fam162b	NA	NA	3.31	1.31	2.71
Fam163a	NA	1.9	1.6	1.52	0
Fam163b	NA	NA	NA	1.52	NA
Fam164a	NA	NA	NA	NA	0
Fam164c	NA	NA	NA	NA	1.71
Fam165b	NA	NA	NA	NA	0
Fam166a	NA	NA	1.16	0.2	0
Fam167a	NA	NA	1.56	2.45	0
Fam167b	NA	NA	2.35	1.27	0
Fam168a	NA	0.01	1.22	0.85	1.53
Fam168b	NA	0	1.76	1.52	0
Fam169a	NA	0.04	0.93	1.48	2.09
Fam169b	NA	NA	1.35	1.28	NA
Fam170a	NA	NA	1.16	5.28	NA
Fam170b	NA	NA	1.35	1.11	NA
Fam171a1	NA	0	2.16	0	0
Fam171a2	NA	NA	1.25	0	0
Fam171b	NA	NA	1.84	0.18	1.46
Fam172a	NA	1.81	0.97	0.75	0.65
Fam173a	NA	2.8	1.94	0.57	0
Fam173b	NA	NA	1.78	0.87	0.88
Fam174a	NA	NA	0.28	0.87	0
Fam174b	NA	2.23	0.51	1.69	0
Fam175a	NA	NA	1.96	0.41	1.96
Fam175b	NA	1.15	1.02	0.27	0
Fam176a	NA	NA	NA	NA	0
Fam176b	NA	NA	NA	NA	0
Fam178a	NA	1	1.3	0	0
Fam178b	NA	NA	0	1.43	0
Fam179a	NA	NA	1.38	1.42	0.59
Fam179b	NA	0.47	1.24	2.38	2.5
Fam180a	NA	NA	NA	0.02	NA
Fam181a	NA	NA	NA	1.08	0
Fam181b	NA	NA	0.02	1.44	0
Fam183b	NA	NA	NA	1.5	NA

Fam184a	NA	1.68	0	2.07	0
Fam184b	NA	NA	0.76	3.56	0.8
Fam185a	NA	NA	0.86	0.71	1.95
Fam186b	NA	NA	NA	3.93	NA
Fam187b	NA	NA	0.37	0.23	0
Fam188a	NA	0.41	1.11	0.12	0
Fam188b	NA	NA	1.35	0	2.58
Fam189a1	NA	2.22	0.07	0.93	3.92
Fam189a2	NA	1.06	0	0.2	1.35
Fam189b	NA	0.55	4.13	0.3	0
Fam18b	NA	NA	NA	NA	0
Fam190a	NA	NA	NA	NA	3.04
Fam192a	NA	2.03	1	3.56	0
Fam193a	NA	0	1.91	1.33	NA
Fam193b	NA	0.56	1.87	0.92	NA
Fam194a	NA	NA	0.37	0.53	NA
Fam195a	NA	NA	0.39	0.76	NA
Fam195b	NA	NA	1.2	1.27	NA
Fam196a	NA	NA	0	1.22	NA
Fam198a	NA	NA	NA	2.55	NA
Fam198b	NA	3.24	2.14	2.8	NA
Fam199x	NA	NA	2.43	0.09	NA
Fam19a1	NA	NA	0	0.58	0.64
Fam19a2	NA	NA	0	0.71	0
Fam19a3	NA	NA	NA	0.84	NA
Fam19a4	NA	NA	0.27	0	0
Fam19a5	NA	NA	0.29	1.2	0
Fam203a	NA	0	0.21	1.14	NA
Fam207a	NA	NA	2.47	1.16	NA
Fam20a	NA	NA	0	1.82	0.94
Fam20b	NA	0.36	1.1	6.78	1.37
Fam20c	NA	NA	4.07	0.22	2.04
Fam210a	NA	NA	1.3	1.07	NA
Fam210b	NA	NA	0.48	0.73	NA
Fam211a	NA	NA	0.4	1.54	NA
Fam211b	NA	2.34	0.59	1.37	NA
Fam212a	NA	NA	3.25	0.11	NA
Fam212b	NA	0.44	0.05	0.93	NA
Fam213a	NA	NA	1.8	0	NA
Fam213b	NA	NA	4.31	1.65	NA
Fam214a	NA	NA	1.25	2.2	NA

Fam214b	NA	2.33	3.17	0.22	NA
Fam216a	NA	0.08	2.26	NA	NA
Fam216b	NA	NA	1.63	5.37	NA
Fam217a	NA	NA	0.88	1.78	NA
Fam217b	NA	NA	1.37	0.1	NA
Fam219a	NA	NA	0.7	1.16	NA
Fam219b	NA	NA	2.42	8.71	NA
Fam220a	NA	NA	1.41	1.36	NA
Fam221a	NA	NA	0.7	0	NA
Fam221b	NA	NA	2.02	0.71	NA
Fam222a	NA	NA	2.6	1.1	NA
Fam222b	NA	0.67	0.12	2.12	NA
Fam227a	NA	NA	NA	0	NA
Fam227b	NA	NA	1.42	0.73	NA
Fam228a	NA	NA	1.04	0.81	NA
Fam229a	NA	NA	0.45	NA	NA
Fam229b	NA	NA	1.89	1.89	NA
Fam24a	NA	NA	NA	1.61	NA
Fam26d	NA	NA	0	1.46	NA
Fam26e	NA	NA	2.26	0	0.06
Fam26f	NA	NA	1.56	0.8	2.66
Fam32a	NA	2.28	0.87	3.15	4.75
Fam35a	NA	2.02	1.7	0.76	0
Fam36a	NA	NA	NA	NA	0.02
Fam38a	NA	NA	NA	NA	0
Fam38b	NA	NA	NA	NA	0
Fam3a	NA	1.76	1.18	1.03	0.1
Fam3b	NA	NA	0	2.93	0.32
Fam3c	NA	0.02	1.58	0.61	0
Fam40a	NA	NA	NA	NA	2.68
Fam40b	NA	NA	NA	NA	0.04
Fam43a	NA	NA	3.25	1.08	0
Fam43b	NA	NA	0.52	0.66	NA
Fam45a	NA	3.93	2.75	0.34	2.4
Fam46a	NA	2.9	0.47	0	1.61
Fam46b	NA	NA	2.22	2.1	0
Fam46c	NA	0.75	0.74	0	0.1
Fam46d	NA	NA	NA	0.65	NA
Fam47a	NA	NA	NA	NA	0
Fam47c	NA	NA	0.92	0.21	NA
Fam47e	NA	2.87	0.88	0.68	NA

Fam48a	NA	NA	NA	NA	0.21
Fam49a	NA	1.47	0.56	2.99	0.2
Fam49b	NA	0	2.92	0.17	0
Fam50a	NA	2.03	2.44	2.33	0
Fam50b	NA	4	0	0.8	3.17
Fam53a	NA	NA	2.46	3.51	0
Fam53b	NA	0	1.21	2	0
Fam53c	NA	0.27	1.27	1.78	0
Fam54a	NA	NA	NA	NA	0
Fam54b	NA	NA	NA	NA	0
Fam55c	NA	NA	NA	NA	2.26
Fam55d	NA	NA	NA	NA	6.09
Fam57a	NA	0.92	0.11	0.4	11.18
Fam57b	NA	NA	0.22	6.56	0
Fam59a	NA	NA	NA	NA	1.1
Fam59b	NA	NA	NA	NA	2.36
Fam5b	NA	2.94	1.69	1.64	4.36
Fam5c	NA	0.93	2.43	0.87	0
Fam60a	NA	0.18	4.18	1.18	0
Fam63a	NA	0.27	1.72	0.81	0.5
Fam63b	NA	2.25	0.01	1.05	0
Fam64a	NA	1.65	2.99	1.08	0
Fam65a	NA	0	1	NA	1.19
Fam65b	NA	0.86	0.06	1.19	0
Fam65c	NA	NA	0.57	1.15	0
Fam69a	NA	0	1.09	1.11	0
Fam69b	NA	NA	1.05	1.9	4.47
Fam69c	NA	NA	NA	1.79	NA
Fam70a	NA	NA	NA	NA	0.82
Fam71a	NA	NA	0.31	0.28	NA
Fam71b	NA	NA	0	10.33	NA
Fam71d	NA	NA	0.16	0.05	NA
Fam71e1	NA	NA	1.86	NA	1.26
Fam71e2	NA	NA	NA	0.9	NA
Fam71f1	NA	NA	2.27	0.44	1.51
Fam71f2	NA	NA	NA	1.44	NA
Fam72a	NA	NA	2.32	0	NA
Fam73a	NA	NA	2.66	0	0
Fam73b	NA	NA	0.81	0	3.73
Fam76a	NA	1.42	0.44	2.07	0
Fam76b	NA	NA	1.72	1.01	0

Fam78a	NA	NA	0.63	2.54	0
Fam78b	NA	NA	0.62	0	1.69
Fam81a	NA	NA	0.4	0.85	0.13
Fam81b	NA	NA	0.71	NA	0.86
Fam82a1	NA	NA	NA	NA	1.3
Fam82a2	NA	NA	NA	NA	2.08
Fam82b	NA	NA	NA	NA	0
Fam83a	NA	NA	NA	0.44	NA
Fam83b	NA	NA	NA	1.82	NA
Fam83c	NA	NA	NA	1.01	NA
Fam83d	NA	NA	2.72	0.11	0.07
Fam83e	NA	0	0.27	1.3	2.29
Fam83f	NA	NA	0.13	1.16	0
Fam83g	NA	NA	0.91	NA	NA
Fam83h	NA	NA	0.72	1.89	1.16
Fam84a	NA	NA	0.93	2.52	0
Fam84b	NA	NA	1.14	2.67	0.54
Fam89a	NA	NA	1.5	1.25	0
Fam89b	NA	NA	NA	2.84	NA
Fam92b	NA	NA	NA	0	NA
Fam96a	NA	NA	0.96	0.79	0
Fam96b	NA	0	2.33	0	0
Fam98a	NA	0.1	1.24	0.94	0
Fam98b	NA	NA	1.97	0.72	3.22
Fam98c	NA	NA	2.48	NA	0
Fanca	0.93	0.52	1.5	0.96	2.72
Fancb	NA	NA	1.05	0.8	1.37
Fancc	0.08	0.76	0.46	0.27	1.46
Fancd2	0	NA	1.4	0	0.93
Fancd2os	NA	NA	1.67	NA	NA
Fance	0.04	0	1.42	0.62	0.79
Fancf	NA	0.78	0.52	1.55	1.6
Fancg	0	0	1.67	0	0.19
Fanci	NA	0	0.87	0	0
Fancl	0	1.16	0.55	1.54	0
Fancm	NA	NA	0.85	0.18	0.13
Fank1	0	NA	0.62	0	0.76
Fap	2.11	0.06	0.05	0	0
Far1	NA	NA	1.92	2.12	0
Far2	NA	2.36	1.53	3.28	0
Farp1	1.08	1.11	0.93	0	0

Farp2	1.53	0.65	0.98	0.07	0.61
Fars2	NA	0.07	0.32	0	0.09
Farsa	NA	0.62	0.73	1.46	0
Farsb	NA	NA	1.31	NA	0.26
Farslb	0.4	NA	NA	NA	NA
Fas	NA	0.25	2.54	0	0
Fasn	0.21	0.52	1.83	0	0
Fastk	0	1.49	0.5	1.53	3.88
Fastkd1	NA	1.61	0.3	2.32	4.52
Fastkd2	NA	0.03	1.13	1.41	1.84
Fastkd3	NA	0.77	0.83	0.66	0
Fastkd5	NA	3.4	1.77	0.83	2.16
Fat1	NA	0.51	2.92	3.52	2.33
Fat2	NA	1.77	0	0.61	0
Fat3	NA	NA	2.83	0.23	3.7
Fat4	NA	1.43	3.04	1.04	0
Fate1	NA	NA	NA	0.76	NA
Fau	2.58	1.87	1.64	1.61	NA
Faxc	NA	NA	0.64	0.79	NA
Fbf1	1.43	NA	0.85	1.48	4.14
Fbl	0.26	3.01	0.98	0.07	3.75
Fblim1	NA	NA	0.72	3.37	2.34
Fbll1	NA	NA	0.1	0	0
Fbln1	0.27	0.92	1.23	0.4	2.6
Fbln2	0	1.28	0.39	1.23	0
Fbln5	1.69	1.45	0.09	0.26	1.75
Fbln7	NA	NA	0.64	0.84	0.96
Fbn1	0.77	0	0.89	0	1.31
Fbn2	0.71	0	1.54	3.1	2.02
Fbp1	5.44	3.3	4.56	0.52	0
Fbp2	0	0.71	2.62	1.25	12.36
Fbrs	NA	0.43	1.08	0.63	0
Fbrsl1	NA	NA	0.05	0.76	2.9
Fbs1	0.6	NA	NA	NA	NA
Fbxl10	0	NA	NA	NA	NA
Fbxl11	0.58	NA	NA	NA	NA
Fbxl12	0	0	1.32	0.78	2.12
Fbxl13	0	NA	1.79	0	NA
Fbxl14	0.92	3	0.51	3.28	7.16
Fbxl15	0	3.25	0.83	0.82	1.44
Fbxl16	0.19	NA	0.36	0	0.96

Fbxl17	1.19	NA	1.93	1.4	0.05
Fbxl18	0.14	0	0.71	1.81	2.31
Fbxl19	6.58	NA	0.57	1.11	2.31
Fbxl2	3.49	1.68	3.18	0.67	0.86
Fbxl20	0	NA	1.66	0.01	0
Fbxl21	0.48	NA	0.16	1.33	NA
Fbxl22	NA	NA	NA	1.76	NA
Fbxl3	NA	NA	0.38	0	0
Fbxl4	2.15	4.69	2.66	2.73	6.87
Fbxl5	0.27	0.36	0.12	0	0
Fbxl6	0.27	1	0.44	NA	3.28
Fbxl7	0.41	0.17	0.36	0.19	0
Fbxl8	0.9	3.5	4.66	2.85	0
Fbxo10	NA	NA	1.94	NA	0
Fbxo11	0.51	0	2.89	0.93	0.6
Fbxo15	1.71	NA	1.32	1.07	0.11
Fbxo16	2.87	NA	1.51	0.13	0
Fbxo17	1.07	1.31	0.72	1.96	NA
Fbxo18	0.46	NA	0.94	0.46	2.61
Fbxo2	4.45	0	0.92	NA	0
Fbxo21	0.43	0	0.08	3.32	0
Fbxo22	0.76	0.98	1.38	NA	1.29
Fbxo24	0	0.71	1.67	0.81	0.13
Fbxo25	2.06	NA	0.4	0	0
Fbxo27	2.11	NA	1.29	1.55	0
Fbxo28	0.6	0.67	1.08	0.57	1.71
Fbxo3	0.55	1.14	0.16	0.56	0
Fbxo30	0.09	NA	1.2	0.53	1.28
Fbxo31	0.39	NA	2.33	0	0.52
Fbxo32	0.94	NA	2.3	0.4	0.47
Fbxo33	NA	NA	2.87	0.88	1.26
Fbxo34	0.62	0.08	1.83	4.81	0
Fbxo36	1.42	NA	2.96	0.48	0
Fbxo38	0.21	1.59	0.74	0.24	0.21
Fbxo39	NA	NA	NA	1.26	NA
Fbxo4	0	1.32	0.51	0.99	0.18
Fbxo40	0	NA	NA	0.84	NA
Fbxo41	NA	0	0.35	0.5	0
Fbxo42	0.55	0.68	1.17	0	0
Fbxo43	0	NA	2.53	0	0
Fbxo44	0.97	NA	0	1.23	2.78

Fbxo45	NA	NA	1.38	1.94	0
Fbxo46	1.22	0	1.42	1.04	0.93
Fbxo47	NA	NA	NA	1.41	NA
Fbxo48	NA	NA	NA	0.24	NA
Fbxo5	0	0.36	2.67	0	3.68
Fbxo6	NA	NA	1.23	3.83	0
Fbxo7	1.7	0	1.37	0	3.38
Fbxo8	0.49	NA	1.91	0.41	3.6
Fbxo9	0.8	1.5	2.32	0.42	1.37
Fbxw10	NA	NA	2.2	NA	0
Fbxw11	0.13	0.77	0.72	0.08	1.91
Fbxw2	0.28	1.03	0.66	1.51	0
Fbxw4	0.28	0	1.04	1.02	0
Fbxw5	0.76	NA	1.91	0.59	2.18
Fbxw7	0.19	2.06	1.61	0	1.63
Fbxw8	NA	NA	0.27	0.24	1.16
Fbxw9	0	NA	3.19	1.18	1.78
Fcamr	0.58	NA	2.71	3.29	0
Fcer1a	4.7	0	0.58	4.78	0
Fcer1g	2.12	0	1.27	0	0
Fcf1	NA	1.32	0	4.23	0.98
Fcgbp	NA	0	2.97	NA	0
Fcgr2b	1.08	0.43	1.7	3.02	1.72
Fcgrt	0.08	0.34	3.18	1.32	2.28
Fcho1	0	0	1.69	0.04	1.46
Fcho2	NA	NA	2.41	0.71	1.19
Fchsd1	0.45	NA	2.43	0	0
Fchsd2	0.52	0.23	0.29	1.36	0.91
Fcmd	1.2	NA	NA	NA	NA
Fcrl1	NA	NA	0.43	2.37	0
Fcrl3	2.29	NA	NA	NA	NA
Fcrl5	NA	NA	NA	1.37	NA
Fcrl6	NA	NA	NA	0.59	NA
Fcrla	NA	NA	0.41	1.51	1.17
Fcrlb	NA	NA	NA	0.06	NA
Fdft1	1.23	0	1.23	0	0
Fdps	1.19	0.06	1.36	2.62	1.4
Fdx1	0	0.03	0.34	0.64	0
Fdx1l	NA	NA	NA	1.32	NA
Fdxacb1	NA	NA	NA	1.19	NA
Fdxr	0.18	1.33	0.65	4.8	0.95

Fech	0.24	1.02	0.62	0.52	0
Fem1a	0.05	NA	0.53	1.84	0
Fem1b	1.78	0.5	5.81	0	0
Fem1c	0.74	0	2.92	0	6.12
Fen1	0.44	1.06	1.43	0.79	0.77
Fendrr	NA	NA	2.11	NA	NA
Fer1l3	0.28	NA	NA	NA	NA
Fer1l4	1.99	2.88	1.51	1.51	0
Ferd3l	0.96	NA	1.57	2.5	NA
Fermt1	NA	NA	NA	0	NA
Fermt2	NA	0.78	0	0.03	1.36
Fermt3	NA	NA	0.3	0.89	0
Fes	0	0.02	0.55	1.23	0
Fetub	0.72	1.53	1.16	5.43	1.38
Fev	3.14	1.77	4.02	0.88	0
Fez1	0.86	0	0	2.66	2.57
Fez2	0	3.24	1.81	0.75	0
Fezf1	NA	NA	NA	1.1	NA
Fezf2	NA	3.53	0.54	2.24	0
Ffar1	NA	NA	NA	0.33	NA
Ffar2	NA	0.5	2.51	1.55	0
Ffar3	NA	NA	NA	1.04	NA
Ffar4	NA	NA	4.09	0.38	NA
Fga	0	1.85	1.81	2.94	10.85
Fgb	0.7	1.35	1.63	7.53	10.62
Fgd1	2.92	0.09	1.16	NA	0
Fgd2	0.94	2.65	3.68	2.72	0.21
Fgd3	0.77	NA	1.06	2.01	0.93
Fgd4	0.58	NA	0.2	1.3	0
Fgd5	1.96	NA	2.55	1.9	0
Fgd6	0.76	1.14	2.5	4.26	0.51
Fgf1	0.64	1.68	1.66	0	0
Fgf10	0	NA	0	0.76	7.42
Fgf11	1.45	NA	1.86	0.19	0
Fgf12	1.38	0.98	0.32	0	0
Fgf13	1.03	2.18	1.56	0.05	2.31
Fgf14	0.34	1.25	0.75	4.87	0
Fgf16	2.58	0.39	1.99	0.83	3.27
Fgf17	3.39	3.22	0.52	2.02	0
Fgf18	0.24	1.49	0.86	2.6	6.05
Fgf2	4.2	0	0.9	NA	0

Fgf20	0.62	2.21	0.52	1.13	0
Fgf21	1.85	1.99	1.12	1.72	0
Fgf22	0.87	0.27	1.1	1.21	1.02
Fgf23	1.79	3.19	2.16	1.02	1.98
Fgf3	1.98	1.8	0.23	1.16	0
Fgf4	0	0	0	1.86	0.69
Fgf5	0.36	0.06	1.09	0	0
Fgf6	2.2	0.92	2.38	3.47	4.02
Fgf7	0	1.6	0.57	0	0
Fgf8	2.7	1.51	0.04	1.49	0
Fgf9	2.52	0.26	0.89	0.22	0
Fgfbp1	0.55	1.35	0	0.87	0
Fgfbp3	NA	NA	2.18	0	1.2
Fgfr1	0.62	1.14	2.21	2.16	0.64
Fgfr1op	0.84	1.22	3.08	0.74	1.1
Fgfr1op2	0.8	NA	0.81	0.03	0
Fgfr2	0.58	0.41	0.78	1.16	1.08
Fgfr3	0.49	0.12	0.59	2.41	1.68
Fgfr4	0.61	0.79	0.29	0	0
Fgfrl1	1.08	NA	0.49	1.45	NA
Fgg	0.58	2.03	1.19	0.89	7.32
Fggy	NA	2.34	1	1.01	1.76
Fgl1	1.44	1.16	2.79	2.48	4.18
Fgl2	0.96	1.07	1.03	1.56	0
Fgr	1.39	0.74	2.11	1.92	0
Fhad1	NA	NA	1.21	0	NA
Fhdc1	NA	NA	0.11	1.3	2.97
Fhit	0.05	0	0.7	0.78	0
Fhl1	1.85	0	1.75	0.87	0
Fhl2	0.93	2.34	2.14	0.66	0
Fhl3	0.83	2.48	2.77	2.34	13.61
Fhl5	1.26	1.33	2.02	0.63	2.39
Fhod1	0.24	0.8	1.88	4.34	1.22
Fhod3	NA	1.08	0.91	0	0
Fibcd1	0.79	NA	0.51	1.36	3.83
Fibin	NA	NA	0	1.6	3.05
Fibp	0.6	0	1.06	1.53	0
Ficd	NA	1.71	1.52	0	0
Fig4	NA	2.51	1.32	0	0
Figf	2.7	1.04	1.54	2.16	4.31
Figla	0.49	NA	0	0.65	NA

Fign	0	NA	1.02	0	1.27
Fignl1	1.84	NA	0.88	1.11	4.23
Fignl2	NA	NA	NA	1.61	NA
Filip1	NA	NA	0.94	1.16	0.02
Filip1l	NA	1.3	1.98	0	0
Fip1l1	0.37	0	1.02	0.24	0
Fis1	NA	2.27	0.64	0.16	0
Fitm1	NA	NA	0.82	1.05	0.93
Fitm2	NA	NA	0.78	2.41	0.72
Fiz1	0.08	NA	3.85	0.63	0
Fjx1	0	1.82	0.75	4.04	0
Fkbp10	1.4	1.32	1.34	NA	0
Fkbp11	0.47	0	0.96	0.38	0
Fkbp14	3.19	0.07	0.83	0.91	0.6
Fkbp15	NA	0	1.02	0	0.56
Fkbp1a	0	0.94	0.36	0	1.92
Fkbp1b	NA	2.02	1.19	1.45	5.25
Fkbp2	0.08	2.08	0.85	0.38	2.56
Fkbp3	2.09	1.74	0	NA	0.33
Fkbp4	0	1.19	0.5	3.08	1.55
Fkbp5	0.64	0.44	0.75	NA	0
Fkbp6	0	1.07	0.27	2.26	0
Fkbp7	0.3	NA	0.64	0.76	0
Fkbp8	1.03	0.61	0.28	6.56	1.27
Fkbp9	0.74	0.26	0.98	2.61	1.36
Fkbpl	1.11	0	0.76	0.77	2.17
Fkhl18	0	NA	NA	NA	NA
Fkrp	2.05	0.55	0.75	2.19	0.57
Fktn	NA	0	2.57	3.74	1.82
Flad1	NA	0.6	0.06	0	0
Flcn	NA	1.86	2.23	5.19	1
Flg	NA	0.96	0.51	NA	0.63
Flg2	NA	NA	NA	0	NA
Fli1	0.06	0	2.08	1.06	0.64
Flii	NA	1.55	1.71	0	1.68
Flna	0.43	2.56	3.86	13.81	1.17
Flnb	0.51	0	0.19	0	0.59
Flnc	NA	1.49	0.87	2.61	4.69
Flot1	1.42	0.02	1.17	0.06	0
Flot2	0.41	0.37	0.97	0.77	0
Flrt1	0	0.83	2.41	2.08	0

Flrt2	1.81	0	9.75	1.96	0
Flrt3	1.87	1.65	0.89	0	0
Flt1	1.13	0.21	2.38	1.28	1.73
Flt3	1.22	1.55	0.25	0	3.28
Flt4	0	0.69	1	2.86	0.9
Flywch1	NA	NA	1.28	1.9	2
Flywch2	NA	NA	1.84	1.03	3.31
Fmn1	NA	NA	0.6	0.33	1.87
Fmn2	0	NA	0.75	2.12	1.37
Fmnl1	1.75	0.24	1.5	1.76	0
Fmnl2	0.44	NA	2.52	1.38	1.18
Fmnl3	0.42	NA	1.48	1.86	0
Fmo1	0.67	2.58	0	0	4.53
Fmo2	1.16	0.99	0	1.12	1.69
Fmo3	0.58	6.11	1.95	0.07	1.69
Fmo4	0	0.34	0	1.71	2.63
Fmo5	1.12	1.37	1.89	2.87	0.28
Fmod	4.11	1.9	1.33	0	0
Fmr1	0.71	0.74	1.03	0.9	1.21
Fmr1nb	NA	NA	1.66	1.38	NA
Fn1	0	0.92	0.59	0	3.36
Fn3k	0	1.03	0.09	1.29	0
Fn3krp	NA	0	1.29	0.57	0
Fnbp1	0.98	0	3.82	0	0
Fnbp1l	NA	0.5	0.73	0.14	0.19
Fnbp4	0.94	0	1.7	0.66	1.78
Fndc1	NA	NA	0.17	2.81	1.61
Fndc3a	NA	0.25	0.88	0.84	0
Fndc3b	NA	0	1.53	1.43	0
Fndc4	0.38	1.63	2.09	1.3	0
Fndc5	0	NA	0.83	0.83	2.59
Fndc7	NA	NA	0.02	0	0
Fndc8	NA	0	1.1	3.22	0
Fndc9	NA	NA	2.6	2.3	NA
Fnip1	NA	2.32	0.39	0	1.68
Fnip2	NA	NA	1.03	1.81	0
Fnta	0.04	0.24	0.98	0	NA
Fntb	0.68	0	1.11	0.83	0
Focad	NA	0	1.27	0	NA
Folh1	1.12	0.79	1.75	0	1.08
Folr1	0.07	2.18	1.95	1.42	0

Folr2	0.4	1.6	1.21	2.26	0
Fopnl	NA	NA	1.92	1.47	NA
Fos	0	1.39	0.68	0.95	0
Fosb	0.4	0	2.34	1.03	2.43
Fosl1	0	0.23	3.4	NA	0
Fosl2	0.04	0.42	0.8	1.93	0
Foxa1	2.26	0.42	1.17	0.7	0
Foxa2	2.46	1.4	2.26	0.9	0
Foxa3	0	NA	1.15	0.93	1.45
Foxb1	0.3	2.03	0.16	0.57	0
Foxb2	0	NA	NA	0.85	NA
Foxc1	0	0	1.05	0	0
Foxc2	1.71	1.98	2.08	0.97	0
Foxd1	0	1.35	1.95	0.57	2.38
Foxd2	0	1.7	1.68	1.22	0
Foxd3	1.43	0.77	0.4	3.5	0
Foxd4	1.79	NA	0.88	NA	NA
Foxe1	NA	NA	NA	1.32	NA
Foxe3	1.56	1.61	0	0.85	6.62
Foxf1	NA	0.23	0.52	0.88	NA
Foxf2	0.93	0.76	1.18	2.66	1.11
Foxg1	NA	1	0	NA	3.31
Foxh1	1.77	0.76	0.03	0.66	1.37
Foxi1	0.49	0.06	0	1.45	4.03
Foxi2	NA	NA	NA	2.56	NA
Foxj1	2.94	0.24	1.34	1.53	0
Foxj2	0	0	3.02	0.87	2.06
Foxj3	NA	0	1.89	1.67	0.52
Foxk1	0.65	NA	1.07	1.23	0.5
Foxk2	NA	0.76	0.49	1.5	0.14
Foxl1	0.51	4.52	1.68	2.03	0
Foxl2	0	1.64	0.04	2.18	0.96
Foxm1	0.78	1.34	0.85	NA	0
Foxn1	4.04	1.09	0	1.21	2.81
Foxn2	0.22	0.06	1.03	0.16	2.05
Foxn3	NA	0	2.67	1.11	0
Foxn4	0	NA	1.46	2.72	0
Foxo1	NA	0	0.75	3.56	0
Foxo3	NA	1.21	1.41	1.69	0
Foxo4	NA	0.24	1.7	3.08	2.62
Foxp1	0.46	NA	0.06	2.47	0

Foxp2	0.14	NA	0.66	0.91	NA
Foxp3	0.57	1.69	1.57	NA	0
Foxp4	0.48	NA	0.83	1.08	0
Foxq1	0.55	NA	0.71	0.41	0.77
Foxr1	NA	NA	NA	0.32	NA
Foxr2	NA	NA	NA	1.09	NA
Foxred1	NA	NA	0.13	1	0
Foxred2	NA	1.06	1.35	1.42	3.01
Foxs1	NA	NA	0.59	0.52	0
Fpgs	1.09	0.04	1.13	1.66	0.17
Fpgt	0.88	1.97	2.3	NA	1.41
Fpr1	0.09	0.38	5.06	7.02	0
Fpr2	NA	1.74	7.13	NA	0
Fpr3	NA	1.52	0.42	0.75	0
Fprl1	1.05	NA	NA	NA	NA
Fra10ac1	NA	NA	0.52	0.44	NA
Frag1	2.02	NA	NA	NA	NA
Frap1	0	NA	NA	NA	NA
Fras1	0.42	0.99	0.45	0.77	2.77
Frat1	1.46	3.46	1.4	0	4.49
Frat2	0.18	3.92	2.28	1.19	0
Frem1	NA	NA	1.94	2.85	0.19
Frem2	NA	NA	1.96	0.05	4.7
Frem3	NA	NA	NA	0.28	NA
Freq	0.27	NA	NA	NA	NA
Frg1	4.32	1.48	1.16	1.64	0
Frk	1.11	1.22	0.62	2.98	0
Frmd3	0	NA	0.88	0.06	2.84
Frmd4a	0	0.23	1.06	1.74	12.32
Frmd4b	0	0.79	1.84	0.97	0
Frmd5	NA	NA	0.59	0.37	1.76
Frmd6	NA	NA	2.14	0	0
Frmd7	NA	NA	NA	1.56	NA
Frmd8	NA	2.51	1.25	0	0.49
Frmpd1	0	1.88	0.67	0	0
Frmpd3	NA	NA	1.83	1.6	2.08
Frmpd4	NA	NA	NA	1.49	NA
Frrs1	NA	NA	1.93	2.57	NA
Frrs1l	NA	NA	0	1.59	NA
Frs2	1	1.79	1.05	0.4	1.32
Frs3	0	1.14	0	1.47	4.84

Fry	NA	0.09	0.91	0	0.66
Fryl	NA	0.01	0.89	0	2.24
Frzb	1.46	1.62	0.16	0	2.95
Fscb	NA	NA	NA	0	NA
Fscn1	0.73	1.41	0.72	1.76	0
Fscn2	0	3.47	0.09	2.26	0
Fscn3	0	1.64	2.41	0.26	4.91
Fsd1	2.98	1.13	2.19	0	10.13
Fsd1l	NA	NA	NA	0.14	NA
Fsd2	NA	NA	0.29	0.74	0.33
Fshb	0	0.25	0	1.13	1.43
Fshr	2.3	0	0.87	1.15	0.17
Fsip1	1.54	NA	1.28	6.55	NA
Fsip2	0.56	NA	NA	0.68	NA
Fst	0.4	1.26	1.4	NA	0
Fstl1	0.07	0.79	0.82	NA	0.02
Fstl3	0.44	3.76	1.68	0	3.8
Fstl4	NA	2.4	0.35	0	6.89
Fstl5	3.75	NA	0.95	3.88	2.08
Ftcd	0.22	2.38	1.27	0.71	1.57
Fth1	0.79	0	3.66	0	0
Fthl17	2.35	NA	NA	3.54	NA
Ftmt	1.01	NA	NA	8.11	NA
Fto	NA	1.74	1.02	0.55	2.17
Ftsj1	NA	0	0	0.46	0
Ftsj2	0.34	0	4.24	2.33	0
Ftsj3	0.01	1.12	1.09	0.96	3.04
Ftsjd1	NA	NA	0.66	2.22	0
Ftsjd2	NA	0.95	1.26	4.04	0
Ftx	NA	NA	1.39	NA	NA
Fubp1	0.59	0	0.31	0.5	1.12
Fubp3	0.19	3.25	0.04	0.98	0
Fuca1	9.19	2.16	2.42	1.37	0
Fuca2	NA	NA	2	1.01	0
Fuk	0.15	NA	1.84	2.65	1.84
Fundc1	NA	NA	1.27	0.75	0
Fundc2	0.49	NA	1.44	0.97	0
Fuom	NA	NA	1.25	0.38	NA
Furin	0.54	1.69	4.21	1.55	0
Fus	NA	1.63	0.11	0.03	1.48
Fusip1	1.18	NA	NA	NA	NA

Fut1	3.67	2.4	2.85	0.04	0
Fut10	0.78	NA	0.92	3.47	1.28
Fut11	0.38	NA	1.65	NA	1.91
Fut2	0	0.16	0.66	0.34	1.03
Fut4	7.74	0	1.46	0	4.07
Fut7	0	0.31	1.76	NA	3.34
Fut8	0.13	0	1.39	3.76	2.67
Fut9	1	0.62	1.74	2.04	1.68
Fuz	NA	0	0.66	1.5	0.7
Fvt1	0.24	NA	NA	NA	NA
Fxc1	2.4	NA	NA	NA	0
Fxn	NA	0	1.1	0	0.32
Fxr1	NA	0	0.43	NA	0.28
Fxr2	NA	1.21	0.34	1.1	0.61
Fxyd1	0.31	2.24	2.03	0.73	2.35
Fxyd2	0.1	0.74	1.24	0.91	1.32
Fxyd3	1.7	0.55	0.31	NA	6.14
Fxyd4	0	NA	0.56	1.28	0.56
Fxyd5	0.7	3.57	0.83	NA	8.35
Fxyd6	0.96	0.9	1.1	0.91	1.84
Fxyd7	0.75	0	2.37	1.82	0
Fyb	1.04	0.01	5.3	4.72	2.37
Fyco1	0	0	1.14	0	1.68
Fyn	0	0	2.74	0.55	5.24
Fyttd1	NA	NA	1.81	1.62	0.06
Fzd1	0.57	1.23	1.08	1.72	0.59
Fzd10	3.54	1.04	0.78	1.26	0
Fzd2	0	1.42	0.98	2.33	0
Fzd3	1.95	1.84	0.34	2.75	1.31
Fzd4	0.63	0.94	0.86	2.43	0
Fzd5	0	0.8	1.2	2.62	0
Fzd6	0	0.74	1.89	0	0
Fzd7	1.04	0.23	0	1.05	3.01
Fzd8	0.23	0.12	0.5	1.61	0
Fzd9	0	0.81	0	1.28	0
Fzr1	0.3	2.15	0.82	1.07	0
G0s2	0	0	1.4	0	0
G2e3	NA	NA	1.82	1.15	0
G3bp1	NA	1.03	0.02	1.9	1.78
G3bp2	NA	0.13	0.89	0.02	0
G6pc	0	2.71	0	0.82	2.07

G6pc2	1.5	1.14	7.7	1.48	2.2
G6pc3	0.75	0.08	1.11	1.34	0
Gaa	0.43	1.13	1.5	0.87	0.34
Gab1	0.76	0.97	3.02	0.77	0
Gab2	0	1.49	0.78	2.5	0
Gab3	0.3	NA	2.53	0	0.84
Gabarap	0	2.09	0.56	0	0.45
Gabarapl1	0.03	0.55	0.46	1.27	1.7
Gabarapl2	0.39	2.15	1.4	0.14	5.54
Gabbr1	0.39	0.9	0.74	1.88	2.24
Gabbr2	NA	NA	NA	0	NA
Gabpa	0.33	0.92	0.32	0.5	1.4
Gabpb1	NA	0.27	1.65	0.51	0
Gabpb2	0	NA	0.61	0.87	NA
Gabra1	2.58	2.1	0.44	1.63	1.56
Gabra2	0	0.39	0.97	0.99	1.27
Gabra3	3.95	1.09	0	4	0.44
Gabra4	0.54	1.34	0	0.64	0
Gabra5	1.29	1.35	0.95	0.99	2.1
Gabra6	1.15	0.44	2.58	0.75	3.3
Gabrb1	1.31	0.04	2.07	1	1.98
Gabrb2	0	1.28	0.4	0.02	0
Gabrb3	0.42	0.06	0.71	5.96	1.89
Gabrd	0	2.37	0.8	1.06	1.47
Gabre	2.53	1.55	0.9	3.8	3.32
Gabrg1	0.57	NA	0.87	0.77	0.98
Gabrg2	0.3	2.14	0.82	0	0.98
Gabrg3	0.78	1.12	1.97	2.38	1.63
Gabrp	1.72	1.24	0.57	3.56	2.87
Gabrq	0.62	1.22	1.6	2.32	2.43
Gabrr1	0.76	1.12	0.81	4.24	2.19
Gabrr2	0	2.64	3.38	0.91	3.06
Gabrr3	NA	NA	NA	0.71	NA
Gad1	0.42	1.2	0	1.56	2.83
Gad2	0	0.38	0.67	3.63	0
Gadd45a	0.19	1.04	0.45	0.03	0
Gadd45b	0.14	0.01	1.98	2.3	0
Gadd45g	0.67	0.16	1.2	1.6	0.03
Gadd45gip1	0	0.81	1.82	0	1.2
Gadl1	2.13	NA	1.76	1.28	NA
Gak	0.24	0	0.22	2.24	1.67

Gal	2.56	2.43	1.21	3.13	0
Gal3st1	1.07	2.31	2.17	0.9	0
Gal3st2	NA	NA	0.32	NA	NA
Gal3st3	NA	NA	NA	0.55	NA
Gal3st4	NA	3.89	1.21	0.48	1.14
Galc	0	0	0.68	0.86	0
Gale	0.24	0.4	1.71	1.08	0
Galk1	0.07	2.71	1.68	0.82	1.19
Galk2	0.01	2.24	1.96	0.27	1.05
Galm	0.59	NA	2.01	0.63	0
Galns	0	0.05	1.13	2.46	2.67
Galnt1	0	2.8	2.91	0.67	4.11
Galnt10	0.18	1.18	1.76	3.1	0.26
Galnt11	0.49	0.76	0.81	0.12	2.1
Galnt12	1.57	0	2.09	0.06	1.82
Galnt13	4.1	NA	0.47	3.5	0
Galnt14	2.16	3.83	0.83	1.09	7.59
Galnt15	NA	NA	0.94	1.38	NA
Galnt16	NA	NA	0.67	0.53	NA
Galnt18	NA	NA	0.61	2.51	NA
Galnt2	0.86	3.25	0.6	0.02	0.66
Galnt3	0.49	0	0.52	0	1.04
Galnt4	0.54	NA	0.71	0.87	1.33
Galnt5	0.42	NA	NA	1.12	NA
Galnt6	0.65	0	3.69	1.01	0
Galnt7	0.25	0.76	1.69	0	1.48
Galnt9	0.55	NA	1.32	1.74	13.2
Galntl1	1.32	NA	NA	NA	3.49
Galntl2	1.21	NA	NA	NA	1.35
Galntl4	3.36	NA	NA	NA	0
Galntl5	NA	NA	0	14.43	2.15
Galntl6	NA	NA	1.22	0.62	NA
Galp	2.56	NA	NA	2.23	NA
Galr1	1.98	0.75	0.42	2.89	1.15
Galr2	0.39	2.61	3.29	3.49	3.27
Galr3	1.01	2	1.17	NA	0
Galt	0.12	0	1.44	3.27	4.46
Gamt	0.24	0	2.21	1.73	2.46
Gan	1.64	1.91	2.18	0	0
Ganab	5.28	0	1.27	0	0
Ganc	NA	NA	1.1	1.01	0

Gap43	1.35	0	3.68	1.03	2.46
Gapdh	NA	0	0.43	2.21	0
Gapdhs	NA	0.69	0.29	3.39	0
Gapt	NA	NA	NA	1.42	NA
Gapvd1	NA	0.89	1.64	4.85	0
Gar1	NA	2.58	3.16	1.01	2.29
Garem	NA	1.65	0.68	0	NA
Gareml	NA	NA	2.47	1.17	NA
Garnl1	2.05	NA	NA	NA	NA
Garnl3	0.51	1.17	2.35	0	0
Gars	0.68	1.53	2.47	0	2.54
Gart	0.05	0.53	0.05	1.34	0.25
Gas1	1.59	0.09	1.15	0.89	1.26
Gas2	0.78	0.99	1.39	0.3	1.94
Gas2l1	1.25	1.4	0.98	0.8	0.63
Gas2l2	NA	NA	0.19	1.84	NA
Gas2l3	0	NA	4.26	0.06	1.04
Gas5	0.51	NA	1.89	0	0
Gas6	0.4	1.64	2.78	3.56	0.49
Gas7	0.67	0	1.94	4.58	0.09
Gas8	0.51	0.85	0	NA	0
Gast	10.31	1.39	2.91	1.7	0
Gata1	0.62	0.71	1.63	1.31	1.27
Gata2	0.83	0.06	1.1	0.7	1.32
Gata3	6.94	0	1.98	0.8	2.07
Gata4	0.89	0.28	0.62	0.71	1.65
Gata5	0.48	NA	0.54	1.11	0
Gata6	0.09	0.35	1.94	0.21	0
Gatad1	NA	0.1	1.7	1.07	2.3
Gatad2a	NA	0	2.28	1.28	0.71
Gatad2b	NA	NA	0.61	1.08	1.42
Gatc	NA	0.74	1.75	2.37	0
Gatm	0	2.79	0.81	1.62	0.16
Gats	1.07	NA	NA	NA	NA
Gatsl2	NA	NA	NA	1.18	NA
Gatsl3	NA	NA	2.57	0	NA
Gba	0	NA	NA	1.09	NA
Gba2	0.87	NA	1.29	0	3.03
Gbas	0	0.98	1.33	1.76	0
Gbe1	0	0.96	2.09	0.55	0
Gbf1	0.09	0	2.2	0	1.25

Gbgt1	0	NA	3.2	1.82	1.71
Gbl	0	NA	NA	NA	NA
Gbp1	2.53	NA	NA	NA	0
Gbp2	0	0.19	2.73	0.81	0.02
Gbp3	NA	NA	1.52	0	0.14
Gbp4	0	NA	NA	1.85	NA
Gbp5	0	NA	NA	1.03	NA
Gbp6	NA	NA	0.37	NA	NA
Gbp7	NA	NA	NA	0.4	NA
Gbx1	NA	2.83	2.03	1.36	1.71
Gbx2	0.75	4.4	1.88	0	0
Gc	0.11	2.53	0.96	4.75	8.61
Gca	0.26	0.73	2.38	0.76	0
Gcat	0	0	1.76	2.26	0.09
Gcc1	0.68	1.27	0.38	0	0
Gcc2	NA	0	0.69	0	0
Gcdh	1.08	0	1.6	0.42	0
Gcet2	1.96	NA	NA	NA	0.02
Gcfc2	NA	0.89	1.44	0.71	NA
Gcg	0.63	1.86	3.59	2.31	7.04
Gcgr	2.85	1.87	0.21	0.65	3.22
Gch1	0	0.17	2.32	0.53	0
Gchfr	NA	1.89	0	0.67	0
Gck	2.32	1.62	0.71	0	2.91
Gckr	1.97	2.04	1.1	0.85	1.69
Gclc	0	1.1	2.15	1.39	2.84
Gclm	0.99	2.88	1.23	0.95	1.94
Gcm1	0.87	0.78	0.1	1.15	0.65
Gcm2	0.69	0	0.43	1.77	1.21
Gcn1l1	0.38	0	1.11	0	2.89
Gcn5l2	0.43	NA	NA	NA	NA
Gcnt1	0	0	2.4	0.11	2.06
Gcnt2	0	0.04	0.56	2.62	0
Gcnt3	0.39	0.82	0.71	1.26	3.31
Gcnt4	NA	NA	NA	0	NA
Gcnt7	NA	NA	0.58	1.16	3.07
Gcom1	NA	NA	NA	NA	0.49
Gcs1	0.08	NA	NA	NA	NA
Gcsam	NA	NA	0.09	1.05	NA
Gcsh	NA	NA	NA	NA	0
Gda	0.29	NA	2.07	0	1.66

Gdap1	0	0.95	0.7	0.36	0.45
Gdap1l1	0.31	1.77	1.34	3.22	0
Gdap2	1.76	0	0.66	0.12	0
Gde1	NA	1.5	0.59	1.21	2.35
Gdf1	2.3	NA	NA	NA	NA
Gdf10	0.77	2.05	2.62	0.18	5.46
Gdf11	4.72	0.02	0	0	0.61
Gdf15	0.54	0.63	2.36	0.55	NA
Gdf2	1.24	2.59	2.1	5.38	4.44
Gdf3	1.13	0.53	0.01	0.96	2
Gdf5	1.88	1.76	2.11	NA	3.61
Gdf6	0	NA	NA	4.17	NA
Gdf7	1.3	NA	NA	2.34	NA
Gdf8	1.38	NA	NA	NA	NA
Gdf9	2.59	1.3	0.45	0.83	0
Gdi1	0.11	1.6	1.26	0.28	2.71
Gdi2	0	0.76	1.46	1.46	0
Gdnf	1.02	1.29	0.29	0.89	2.27
Gdpd1	NA	NA	1.13	1.66	0
Gdpd2	1.96	2.19	2.17	0.34	1.71
Gdpd3	NA	0.07	0.35	0.06	0
Gdpd4	NA	NA	NA	1.14	NA
Gdpd5	NA	2.13	1.77	1.49	3.84
Gdpgp1	NA	NA	NA	0.5	NA
Gem	0	1.97	1.37	7.38	0
Gemin2	NA	0.96	0.33	0.73	NA
Gemin4	0.34	0	3.24	3.23	2.78
Gemin5	0	NA	0.36	2.68	1.6
Gemin6	0	0.41	1.12	0.78	4.24
Gemin7	1.46	2.89	4.62	1.17	0.91
Gemin8	NA	0.1	0.43	0.51	0.53
Gen1	NA	NA	2.65	0.44	0.06
Get4	NA	NA	2.1	0.22	NA
Gfap	0.66	0.93	0.11	0	0
Gfer	1.35	1.22	1.1	NA	0.94
Gfi1	0.66	0	2.05	2.28	3.27
Gfi1b	2.46	0	0.68	1.12	0.85
Gfm1	NA	0.9	0.67	0.47	1.23
Gfm2	NA	NA	0.72	0.45	0
Gfod1	NA	0.95	2.14	2.36	0
Gfod2	NA	2.69	1.52	3.9	0

Gfpt1	0.72	0.87	1.53	0	0
Gfpt2	0.68	1.81	0.47	1.62	0
Gfra1	1.7	1.22	1.16	0	0
Gfra2	2.41	0.79	0.69	1.51	0
Gfra3	1.77	1.83	2.02	0.72	NA
Gfra4	1.99	0.91	3.87	1.21	5.75
Gfral	NA	NA	NA	1.63	NA
Gga1	0	0.84	0.35	1.52	1.01
Gga2	0.03	0.07	2.86	2.67	0
Gga3	0.74	0	3.91	3.16	0.63
Ggact	NA	NA	3.91	1.59	NA
Ggct	NA	1.2	0.07	0.13	2
Ggcx	0	3.84	0.16	0.07	0.16
Ggh	1.32	0	0.28	1.03	0
Ggn	0	NA	2.71	0.75	6.85
Ggnbp1	NA	NA	NA	1.01	NA
Ggnbp2	NA	0.58	1.46	0.26	1.56
Ggps1	0.02	2.09	0.88	0.72	0
Ggt1	0	0.52	0.17	NA	0.08
Ggt5	NA	0.5	2.73	1.36	0
Ggt6	NA	NA	6.27	0.63	0
Ggt7	NA	NA	1.42	0.47	0.85
Ggta1	NA	NA	NA	NA	1.08
Ggtl3	0.76	NA	NA	NA	NA
Ggtla1	0	NA	NA	NA	NA
Ghdc	NA	NA	0.9	1.21	8.56
Ghitm	3.23	2.29	0.63	1.34	0
Ghr	1.75	1.7	0.9	1.04	3.39
Ghrh	1.29	2.02	1.74	3.16	3.73
Ghrhr	0.28	1.49	0	1.19	0
Ghrl	1.56	NA	2.38	1.57	5.28
Ghsr	0.85	0.7	4.1	1	0
Gid4	NA	1.86	0.7	2.43	NA
Gid8	NA	0.19	0.95	0	NA
Gif	0.7	2.35	1.5	0.54	3.56
Gigyf1	NA	NA	2.93	0	1.55
Gigyf2	NA	0.53	0.39	0	0
Gimap1	NA	NA	1.27	1.29	NA
Gimap4	NA	0.09	0.3	1.76	0.66
Gimap5	NA	0	2.23	1.49	2.58
Gimap6	NA	0	0	3.52	2.89

Gimap7	NA	NA	0.53	0.79	0
Gimap8	NA	NA	0.39	1.92	0
Gin1	NA	2.37	1.04	1.05	4.05
Ginm1	NA	NA	1.42	0.37	NA
Gins1	NA	0.86	0.96	0	1.44
Gins2	NA	0.4	1.82	NA	2.93
Gins3	NA	0.19	1.89	0.87	0
Gins4	NA	2.02	0.73	0	2.83
Gip	1.83	1.21	1.18	0.99	3.26
Gipc1	NA	0.23	1.26	1.28	0
Gipc2	NA	2.22	2.94	0.98	1.06
Gipc3	NA	NA	3.28	0.87	0
Gipr	NA	2.6	1.57	1.01	0
Git1	NA	1.91	0.99	1.28	1.51
Git2	0	0.99	1.04	0	0.88
Gja1	0.97	1.87	0.91	1.18	0
Gja10	3.89	NA	0.91	1.47	NA
Gja12	0.9	NA	NA	NA	NA
Gja3	0.24	1.65	0.1	1.18	0
Gja4	3.34	1.11	1.7	0	0
Gja5	1.14	2.41	1.14	1.09	5.19
Gja7	0.46	NA	NA	NA	NA
Gja8	0.73	3.45	0.41	3.3	0
Gja9	0.52	NA	NA	NA	NA
Gjb1	0	2.24	0.32	2.32	1.54
Gjb2	0	NA	3.6	0.41	4.54
Gjb3	1.42	1.18	1.81	4.74	0
Gjb4	0.92	0.65	0	4.37	5.61
Gjb5	1.36	2.34	1.78	0.12	2.34
Gjb6	1.24	NA	0.62	0.16	0
Gjc1	2.37	0.34	1.37	3.23	1.15
Gjc2	NA	1.26	0	1.19	0
Gjc3	NA	NA	NA	1.74	NA
Gjd2	NA	1.27	2.13	1.75	1.09
Gjd3	NA	NA	NA	1.09	NA
Gjd4	NA	NA	1.71	0	2.73
Gje1	1.1	NA	NA	NA	NA
Gk2	4.81	1.41	2.22	0.02	0
Gk5	NA	NA	2.03	0.18	1.21
Gkap1	4.09	NA	0.44	1.19	2.11
Gkn1	2.63	1.15	0.26	0.7	11.09

Gkn2	NA	NA	4.95	1.58	1.08
Gla	0.65	0	0.69	0.58	0
Glb1	0.28	0.45	1.11	0.95	0
Glb1l	0.04	0.55	1.08	2.25	0.19
Glb1l2	NA	2.08	0.49	0	0
Glb1l3	NA	NA	1.11	2.01	NA
Glcci1	0.79	NA	1.73	0.03	0
Glce	0.06	0.81	1.29	0.97	1.32
Gldc	2.1	0.93	0.86	1.33	0
Gldn	NA	NA	NA	0	NA
Gle1	NA	0.98	0.87	1.17	0.81
Gle1l	1.65	NA	NA	NA	NA
Glg1	0.75	0	1.09	0	0
Gli1	NA	1.12	0.88	1.87	0
Gli2	0.45	0.37	0.89	1.71	1.72
Gli3	0.9	1.26	1.15	0.24	0
Glipr1	0	0.92	0.86	NA	0
Glipr1l1	NA	NA	1.62	2.05	0
Glipr1l2	NA	NA	NA	0	NA
Glipr2	NA	NA	1.02	2	1.28
Glis1	2.11	NA	7.88	1.38	0.4
Glis2	0.37	NA	0.75	0.74	2.66
Glis3	NA	NA	0.27	0.69	0.2
Glmn	0.41	1.25	2.72	0.68	0
Glo1	0.38	0.24	1.75	1.11	0
Glod4	NA	2.76	0.73	0.82	3.07
Glod5	NA	NA	0.7	0	0
Glp1r	0.9	2.1	0	0	0
Glp2r	0.89	NA	NA	0	NA
Glra1	0	1.24	1.7	0.82	0
Glra2	1.22	2.2	0	0.55	2.57
Glra3	0.64	0.1	1.67	0.25	2.82
Glra4	NA	NA	NA	0.65	NA
Glrb	0.91	0.65	0	3.98	3.19
Glrx	NA	0	1.55	0	0.01
Glrx2	0.46	0	0.84	1.27	0
Glrx3	NA	0.59	0.42	1.77	0.17
Glrx5	NA	1.95	3.75	0.9	0
Gls	1.65	0.33	0.88	0	0.61
Gls2	0	0	0	0.28	0.47
Glt1d1	NA	NA	NA	0.43	NA

Glt25d1	0.06	NA	NA	NA	1.73
Glt25d2	4.22	NA	NA	NA	3.16
Glt6d1	NA	NA	NA	1.64	NA
Glt8d1	NA	0.61	0.83	0	0
Glt8d2	NA	1.69	0.49	0	1.19
Gltp	0.73	2.12	2.78	1.03	1
Gltpd1	NA	1.44	3.49	0.52	1.02
Gltpd2	NA	NA	NA	0.83	NA
Gltscr1	1	0	3.33	1.86	0.09
Gltscr1l	NA	0.02	0.69	0	NA
Gltscr2	0	0	0.76	1.27	1.97
Glud1	0.38	1.56	1.56	NA	0
Glul	1.19	0.04	3.24	1.04	0.75
Gluld1	2.38	NA	NA	NA	NA
Glyat	0	0.97	0	0.8	1.58
Glycam1	0	NA	NA	NA	NA
Glyctk	NA	NA	0.71	0	0
Glyr1	NA	1.97	2.55	0.12	1.09
Gm2a	1.21	1.63	0.59	0.99	2.54
Gmcl1	NA	0.34	1	1.93	0
Gmds	0.34	0.54	0.11	0.7	0
Gmeb1	0.64	0.48	0.39	0.91	0
Gmeb2	0.57	1.49	1.09	3.52	1.38
Gmfb	0	0	2.14	0.82	0
Gmfg	2.01	0	0.85	1.34	0.35
Gmip	0.7	1.22	5.19	1.86	0.18
Gml	NA	1.69	3.58	0.87	NA
Gmnn	0.01	0	2.03	0.04	0
Gmppa	0.47	0.92	0.33	0.41	0
Gmppb	0.67	1.39	1.12	0.82	1.79
Gmpr	0.98	0.52	0.96	0	NA
Gmpr2	0.44	2.42	0.02	0.39	1.28
Gmps	0.1	0	1.51	0.06	0
Gna11	0	1.37	0.96	1.77	2.78
Gna12	1.24	0.09	0.18	0	0.82
Gna13	0.01	0.9	1.39	0.78	0.46
Gna14	3.27	1.29	0.77	1.72	1.59
Gna15	0.68	4.36	2.1	1.94	5.41
Gnai1	0.57	0.72	0.39	2.63	0
Gnai2	0	1.76	2.74	0.19	0
Gnai3	2.84	0	3.28	1.03	0

Gnal	0.6	0.36	0.48	0.45	0
Gnao1	NA	0.76	1.2	3	1.75
Gnaq	0.97	0.79	0.68	0	0
Gnas	0.09	0	0.62	0	0.56
Gnat1	1.41	1.47	0.34	0.39	5.96
Gnat2	0.61	2.46	0.67	1.17	2.32
Gnaz	NA	0	1.12	0.29	0
Gnb1	0.69	1.03	0.59	0.37	0
Gnb1l	0.22	2.12	3.52	NA	0
Gnb2	0.42	2.84	2.39	0.58	0
Gnb2l1	NA	0	1.75	0.15	0
Gnb3	0	1.57	0.55	0.96	0
Gnb4	0.2	NA	3.37	0.68	3.24
Gnb5	0.28	1.65	1.8	0.86	0
Gne	0.51	0.16	2.29	1.23	0
Gng10	0.79	0	3.14	NA	2.17
Gng11	0.83	0.84	3.28	0.39	0.69
Gng12	0.81	0.99	0.02	0	0.59
Gng13	1.22	1.9	0	NA	0
Gng2	0.53	NA	0.6	0.86	2.25
Gng3	2.32	0	1.04	NA	0
Gng4	0	1.02	0.66	1.1	0
Gng5	0.72	1.86	1.81	0	3.15
Gng7	0.1	1.48	1.67	1.11	0.92
Gng8	1.17	NA	2.15	2.82	2.89
Gngt1	0.29	1.74	0.32	2.47	0
Gngt2	0.34	NA	2.55	NA	2.72
Gnl1	NA	1.05	2.26	1.28	0
Gnl2	0	0.77	4.63	0.79	7.13
Gnl3	NA	1.45	1.06	1.26	5.92
Gnl3l	NA	0.67	0	0.01	1.18
Gnmt	0.34	2.45	0.89	0	2.32
Gnpat	0.17	3.73	1.53	1.26	2.8
Gnpda1	0.06	1.38	2.46	NA	14.09
Gnpda2	0.75	NA	1.87	0.68	1.45
Gnpnat1	0	NA	2.62	0.68	0
Gnptab	NA	0.09	1.8	3.25	1.22
Gnptg	NA	NA	0.94	0.01	2.99
Gnrh1	NA	NA	NA	0.67	NA
Gnrhr	0	0.85	0.83	0.88	2.2
Gns	0.73	0	0.87	1.04	0

Golga1	0	1.35	0.74	0.87	0
Golga2	0.65	0.89	1	1.17	2.59
Golga3	0.09	1.37	0.43	0	0
Golga4	0	0.65	1.86	2.22	0
Golga5	0.59	1.95	2.31	0.61	1.04
Golga7	1	3.23	4.4	1.57	5.59
Golga7b	NA	NA	1.51	3.05	0.68
Golgb1	0.48	0.76	1.41	0	1.31
Golim4	NA	0	0.89	0.72	1
Golm1	NA	1.48	1.99	1.12	2.64
Golph2	5.52	NA	NA	NA	NA
Golph3	0.3	0.72	2.51	0	0.87
Golph3l	NA	1.4	1.05	0.11	0
Golph4	0	NA	NA	NA	NA
Golt1a	NA	NA	1.63	11.37	3.51
Golt1b	NA	1.1	0.51	0.83	0
Gon4l	NA	0	2.61	0	1.35
Gopc	1.32	NA	0.76	NA	0
Gorab	NA	NA	0	1.35	0
Gorasp1	0.48	1.63	1.1	1.21	1.65
Gorasp2	1.01	1.08	1.98	0	0
Gosr1	0.36	0.38	1.13	0.56	0
Gosr2	1.08	0.74	0	0.73	1.07
Got1	0	0	3.36	0.84	1.51
Got1l1	NA	NA	0	1.37	NA
Got2	0	0.96	0.34	1.09	0
Gp1ba	5.08	0	1.61	1.97	0
Gp1bb	0	1.89	0.65	NA	0
Gp2	NA	1.63	1.2	0.63	8.29
Gp5	3.62	0	0.79	2.36	0.21
Gp6	0.23	2.21	2.25	NA	0.03
Gp9	0	1.41	0.81	4.82	0
Gpa33	0.09	0	0.9	1.1	0
Gpaa1	0.25	0.84	1.1	9.86	0.24
Gpam	1.2	NA	0	0	0.12
Gpank1	NA	NA	0.98	0	NA
Gpat2	NA	NA	2.67	0	1.12
Gpatch1	NA	0	0	0.23	0
Gpatch11	NA	NA	1.71	0.45	NA
Gpatch2	NA	0	0.37	0	0
Gpatch2l	NA	0	1.72	0.3	NA

Gpatch3	NA	1.24	0	0.02	0.92
Gpatch4	NA	1.11	1.06	1.04	0
Gpatch8	NA	0	0.33	0	0.04
Gpbar1	1.92	NA	NA	0.56	NA
Gpbp1	NA	NA	1.41	0	3.72
Gpbp1l1	NA	1.15	2.37	1.68	6.35
Gpc1	0.57	0.92	1.97	0.35	0
Gpc2	1.83	NA	0.93	0.91	0.62
Gpc3	0.34	1.46	0.32	0.92	0
Gpc4	2.98	0.86	1.58	1.23	1.95
Gpc5	0.33	1.53	1.91	0.84	0
Gpc6	1.84	NA	0.04	0	1.19
Gpcpd1	NA	NA	1	0.37	NA
Gpd1	1.51	0.8	2.06	0.73	2.44
Gpd1l	NA	0	4.35	0.79	0
Gpd2	0	0.68	1.69	1.02	0.35
Gper	NA	NA	NA	NA	1.41
Gpha2	1.17	NA	1.12	1.58	0
Gphb5	0	NA	NA	0.02	NA
Gphn	0.84	0	1.87	0.35	0.21
Gpiap1	0	NA	NA	NA	NA
Gpihbp1	NA	NA	0	0	1.52
Gpkow	0	0.54	0.85	1.01	1.99
Gpld1	0.81	0.08	0.88	2.28	1.85
Gpm6a	0.45	0.7	0.17	1.06	0.52
Gpm6b	0.06	0.46	0.25	0.72	0
Gpn1	NA	0.72	1.17	1.05	0
Gpn2	NA	1.47	1.42	2.08	NA
Gpn3	NA	4.25	0.56	0.95	6.3
Gpnmb	0.89	0.68	1.34	0.59	0
Gpr1	0.3	2.3	1.01	4.28	1.23
Gpr101	NA	NA	0.12	0.95	NA
Gpr103	0.83	NA	NA	NA	NA
Gpr107	NA	0	1.21	0.14	0
Gpr108	0.9	NA	2.12	1.04	0
Gpr109b	1.06	NA	NA	NA	NA
Gpr110	0.29	1.14	0.34	0.02	0
Gpr111	NA	NA	NA	0.89	NA
Gpr112	NA	NA	NA	0.01	NA
Gpr113	NA	NA	NA	0	NA
Gpr114	NA	NA	2.37	2.31	3.69

Gpr115	0	NA	2.62	NA	1.46
Gpr116	0	0.91	0.55	0	0
Gpr119	0.37	NA	NA	1.15	NA
Gpr12	0	1.31	0.88	2.23	0
Gpr120	0.37	NA	NA	NA	NA
Gpr123	0.72	NA	0.63	1.17	1.08
Gpr124	0.67	2.6	1.37	0.35	0
Gpr125	0.9	0.75	0.56	3.66	0
Gpr126	0	1.24	0.77	3.06	0
Gpr128	4.37	NA	NA	1.29	NA
Gpr132	1.13	0.75	1.27	0	0
Gpr133	3.63	NA	3.76	0	1.91
Gpr135	0.63	1.02	0.68	0.27	2.13
Gpr137	NA	0.4	0	0.18	3.16
Gpr137b	NA	1.42	1.99	3.36	0
Gpr137c	NA	NA	0.1	1.48	1.14
Gpr139	NA	NA	NA	0.66	NA
Gpr141	2.18	NA	NA	0	NA
Gpr142	0.85	NA	NA	1.17	NA
Gpr143	0.57	2.54	9.67	1.63	2.83
Gpr146	0.87	NA	0.25	1.78	0.66
Gpr149	0.54	NA	NA	0.6	NA
Gpr15	NA	2.74	0.01	0.6	3.76
Gpr150	2.56	NA	0	0.39	2.09
Gpr151	0.75	NA	NA	1.46	NA
Gpr152	2.45	NA	NA	0.94	NA
Gpr153	2	0.1	4.01	0.04	4.85
Gpr155	1.78	NA	0.72	1.55	0
Gpr156	0.68	NA	NA	0.05	NA
Gpr157	NA	NA	NA	0.25	NA
Gpr158	0.16	NA	0	2.16	2.25
Gpr160	0	NA	0.26	1.52	6.87
Gpr161	NA	0.6	1.14	3.98	0
Gpr162	NA	1.96	2.86	2.86	2.55
Gpr17	NA	0	1.89	0.31	9.6
Gpr171	0	2.31	2.26	0	4.26
Gpr172b	3.92	NA	NA	NA	1.2
Gpr173	0.92	1.52	0	0	1.93
Gpr174	NA	NA	0.55	5.22	0
Gpr176	NA	2.32	2.38	0	7.66
Gpr179	NA	NA	NA	1.05	NA

Gpr18	1.54	0	0.21	0.1	0
Gpr180	NA	NA	3.06	1.93	0
Gpr182	NA	2.44	1.94	2.63	0
Gpr183	NA	0	2.29	4.78	0
Gpr19	0.45	0	9.14	0	0
Gpr20	1.34	2.51	12.34	0.94	0
Gpr21	3.22	NA	NA	1.97	NA
Gpr22	0	1.43	1.83	15.36	0
Gpr23	0	NA	NA	NA	NA
Gpr25	NA	3.93	0.21	0.67	0.45
Gpr26	1.55	NA	0.72	2.98	0
Gpr27	0.97	0	1.26	0.99	0
Gpr3	1.06	1.51	2.57	1.55	0.17
Gpr30	0	NA	NA	NA	NA
Gpr34	0.14	NA	2.77	0.04	0
Gpr35	0	1.96	0.54	1	2.09
Gpr37	3.06	0.8	1.48	17.03	1.63
Gpr37l1	1.61	0.45	0	1.97	4.98
Gpr39	0.55	2.25	0.61	NA	0
Gpr4	1.04	1.9	0.1	0.49	3.32
Gpr44	0	NA	NA	NA	0
Gpr45	0.78	0.85	0.09	1.1	0.68
Gpr50	0.44	1.48	0.8	0.69	1.81
Gpr52	NA	NA	NA	1.7	NA
Gpr55	NA	NA	NA	4.51	NA
Gpr56	0	0	0.57	1.43	1.96
Gpr6	1.29	0.77	0.79	1.03	3.73
Gpr61	0.55	NA	0	2.14	0
Gpr62	NA	NA	0	1.17	NA
Gpr63	2.94	1.24	0.42	13.74	2.04
Gpr64	0.07	0	0.24	0.47	1.89
Gpr65	0.55	0	2.8	1.64	0
Gpr68	0.57	2.41	4.82	0.72	3.86
Gpr75	0	0.06	1.98	NA	2.61
Gpr77	0	NA	NA	NA	NA
Gpr81	0	NA	NA	NA	2.58
Gpr82	2.64	NA	NA	0.62	NA
Gpr83	1.69	NA	1.45	0.02	0
Gpr84	0.45	NA	3.02	0.56	0
Gpr85	1.84	1.99	2	0	1
Gpr87	0.89	1.2	0.64	1.96	3.89

Gpr88	0	1.78	1.33	0	0
Gpr97	0.39	0.14	1.02	0.54	0
Gpr98	NA	0.92	1.28	0	0
Gprasp1	6.73	0	2.04	NA	3.91
Gprasp2	3.89	NA	NA	0	0
Gprc5a	NA	2.79	0	1.24	2.09
Gprc5b	0.47	1.32	1.25	0	1.42
Gprc5c	1.18	1.67	1.23	0	1.47
Gprc5d	1.36	3.17	0.2	2.35	3.33
Gprc6a	0.75	NA	0	1.58	NA
Gprin1	1.18	NA	0.47	0.92	0
Gprin2	NA	0	1.04	0	3.11
Gprin3	NA	NA	1.39	1.65	NA
Gps1	0.29	0.07	0.27	0	0
Gps2	0.44	0	2.31	0	NA
Gpsm1	1.85	NA	0.5	0.72	0.29
Gpsm2	NA	0.03	1.38	0.22	0
Gpsm3	1.12	1.31	1.55	1.16	0
Gpsn2	0	NA	NA	NA	NA
Gpt	NA	2.36	3.87	1.17	0.95
Gpt2	0.73	NA	1.73	1.27	1.19
Gpx1	0.42	0.77	0.92	0	0
Gpx2	0.06	1.14	0.27	0.07	6.97
Gpx3	0	1.55	4.8	6.76	0
Gpx4	0.85	0.3	0.53	0	0
Gpx5	0.88	1.71	0.33	0	1.11
Gpx6	0	NA	NA	0.73	NA
Gpx7	2.79	1.11	0.77	2.96	3.2
Gpx8	NA	NA	0.79	0	0
Gramd1a	NA	NA	5.21	3.45	0
Gramd1b	NA	1.03	2.15	0	2.26
Gramd1c	NA	0	1.2	0.28	3.49
Gramd2	NA	NA	NA	2.79	NA
Gramd3	NA	0	2.21	0.35	1.87
Gramd4	NA	0	2.09	2.44	2.62
Grap	0.5	0.67	0.18	1.93	1.68
Grap2	0.93	0.2	0.93	0.48	0
Grasp	0.09	NA	0.05	0.31	2.21
Grb10	0.74	1.9	0.42	0	2.36
Grb14	0.27	1.01	0.51	0.64	0
Grb2	0.32	0	0.81	1.49	0

Grb7	1.21	2.23	0.07	1.28	2.59
Greb1	0	0	0.39	0.7	9.69
Greb1l	NA	1.01	0	1.43	NA
Grem1	1.22	1	4.04	0	3.1
Grem2	0	1.87	0.44	1.77	0
Grhl1	NA	NA	2.56	1.15	NA
Grhl2	NA	0.93	0	0	1.75
Grhl3	NA	NA	0.71	2.25	0
Grhpr	0.39	0.03	0	1.81	0
Gria1	0.72	0.82	0.94	0	0
Gria2	0.88	0.34	0.38	1.29	0.63
Gria3	0.61	0.17	1.76	1.15	7.96
Gria4	1.53	1.01	0.51	1.31	0
Grid1	0.76	NA	0.05	0.11	2.47
Grid2	0	0.94	0.01	3.4	0
Grik1	0.29	0.43	0.81	0	2.04
Grik2	1.94	0.69	0.48	3.28	0
Grik3	0	0	0.61	1.13	0
Grik4	0	1.01	0.93	2.09	0
Grik5	0.79	0	1.9	0.93	0
Grin1	1.48	0.57	0.03	0.44	0
Grin2a	0	1.31	1.36	2.75	2.92
Grin2b	0.35	0	0.07	0	3.29
Grin2c	1.48	2.29	0.83	1.94	3.38
Grin2d	0.51	1.06	1.21	5.52	0
Grin3a	3.07	NA	0.63	0.1	0
Grin3b	1.48	NA	2.39	NA	0
Grina	0	1.38	5.01	1.5	0
Grinl1a	1.64	NA	NA	NA	0
Grip1	0.66	1.55	0.62	1.15	0
Grip2	NA	1.74	0	1.77	0
Gripap1	NA	NA	1.24	0.97	0.59
Grk1	2.32	1.96	0	0.94	NA
Grk4	NA	0.64	1.72	0.74	2.24
Grk5	NA	0	0.31	0.01	3.98
Grk6	NA	1.51	0.08	3.83	0
Grlf1	NA	NA	NA	NA	0
Grm1	0.27	1.19	0.16	1.79	NA
Grm2	0.23	2.24	1.02	0.36	1.37
Grm3	1.28	1.48	0.75	1.45	0
Grm4	NA	0.76	0.05	0	6.88

Grm5	0.61	0	0.91	0	0
Grm6	NA	NA	NA	2.19	NA
Grm7	0	1.01	0.72	0.38	0
Grm8	1.27	0.81	0.76	0.79	2.84
Grn	0.26	1.21	1.75	0	3.8
Grp	0.09	1.32	1.99	0	3.22
Grpel1	2.53	1.51	6.28	2.74	0
Grpel2	0.21	NA	0	2.46	0
Grpr	1.17	0.76	0.65	3.92	2.38
Grrp1	NA	NA	NA	NA	0
Grsf1	0	0	0.54	0.56	0
Grtp1	2.97	4.12	0.36	0.16	0
Grwd1	0	1.55	1.05	1.6	0.26
Gsap	NA	1.91	1.28	2.52	NA
Gsc	2.2	NA	0.27	3.67	NA
Gsc2	NA	0.21	4.54	1.19	0
Gscl	4.13	NA	NA	NA	NA
Gsdm1	2.03	NA	NA	NA	NA
Gsdma	NA	NA	NA	5.91	NA
Gsdmc	NA	NA	0.03	0.05	9.88
Gsdmd	NA	0	0.19	0.5	1.22
Gsdmdc1	0	NA	NA	NA	NA
Gse1	NA	0	1.59	0	NA
Gsg1	1.35	1.09	0.57	0	4.04
Gsg1l	NA	NA	0	0	1.96
Gsg2	0.6	NA	4.36	12.77	1.04
Gsh1	0.78	NA	NA	NA	NA
Gsh2	2.11	NA	NA	NA	NA
Gsk3a	NA	0.78	2.01	1.3	4.94
Gsk3b	0.74	2.13	1.38	1.31	1.69
Gskip	NA	NA	2.05	0.74	NA
Gsn	0.54	0.74	1.17	0.33	0
Gspt1	0.48	1.02	0.41	0.31	1.59
Gspt2	0.09	1.57	1.71	0	0
Gsr	0.76	0.37	2.54	2.72	3.6
Gss	0	0	1.61	0.16	0
Gsta1	0.28	NA	NA	NA	NA
Gsta2	0	NA	NA	NA	NA
Gsta3	1.4	3.08	1.26	4.13	3.06
Gsta4	0.16	2.02	0.36	2.11	1.25
Gstcd	NA	1.4	1.74	1.14	0

Gstk1	0.67	2.7	3.21	1.57	0.05
Gstm1	0.53	0	1.31	0	0.57
Gstm2	0	1.06	1.31	0.02	1.56
Gstm3	0	0.8	1.12	1.64	1.3
Gstm4	0.06	0	2.42	4.26	0
Gstm5	1.03	1.38	8.02	NA	1.8
Gsto1	1.68	0	3.01	1.01	0.6
Gsto2	NA	NA	1.12	0.92	0
Gstp1	0.75	0	0.06	0.68	0
Gstt1	0.96	1.95	0.87	1.41	1.26
Gstt2	0.5	4.29	1.96	NA	2.14
Gstz1	0.31	2.39	1.85	NA	0.33
Gsx1	NA	NA	0	0.27	0
Gsx2	NA	NA	0.57	0.95	1.26
Gtdc1	NA	1.4	0.94	0	0
Gtdc2	NA	NA	1.38	0	NA
Gtf2a1	0.41	1.33	2.58	0.49	1.02
Gtf2a1l	NA	NA	1.19	NA	NA
Gtf2a2	0.99	0	0.95	0	1.1
Gtf2b	0.09	0	1.89	2.64	0
Gtf2e1	0.61	1.8	0.98	0.73	0
Gtf2e2	0	3.36	1.18	0.82	0
Gtf2f1	0.65	1.58	0.03	0.86	1.4
Gtf2f2	0.8	1.7	3.18	0.85	0.88
Gtf2h1	0.44	1.61	1.29	1.19	0
Gtf2h2	0.26	NA	2.53	NA	0.11
Gtf2h3	0.16	3.06	3.24	0.36	1.31
Gtf2h4	0.18	1.77	0.39	0.91	0
Gtf2h5	NA	0	0.46	0	0
Gtf2i	0.66	0.09	0.82	3.14	3.64
Gtf2ird1	0.27	0	2.04	3.2	2.21
Gtf2ird2	NA	NA	1.66	NA	4.08
Gtf3a	0.33	0	2.19	0	0
Gtf3c1	1.15	0	0.46	1.44	0
Gtf3c2	0.28	0	0	2.23	2.27
Gtf3c3	NA	0.44	0.61	0.28	0
Gtf3c4	0.36	0	1.04	1.56	1.3
Gtf3c5	0	1.54	0.7	0.01	2.81
Gtf3c6	NA	NA	2.74	0.27	0
Gtpbp1	0	1.34	0	2.92	2.41
Gtpbp10	NA	2.08	2.02	1.06	0

Gtpbp2	0.12	1.37	1.82	1.56	0
Gtpbp3	0	0.67	0.39	0.85	0
Gtpbp4	0.07	1.6	2.9	0.76	2.17
Gtpbp5	2.8	NA	NA	NA	0
Gtpbp6	NA	0	0.98	1.22	0
Gtpbp8	NA	0.23	1.13	0.19	5.09
Gtse1	0.12	2.04	1.42	1.71	0
Gtsf1	NA	NA	2.91	0.86	0.88
Gtsf1l	NA	NA	1.81	1.48	0
Guca1a	0.44	1.38	2.5	1.7	0
Guca1b	1.98	0.53	0.64	1	0
Guca2a	0.24	0	0	1.16	0
Guca2b	0.13	0.08	1.67	1.11	0
Gucd1	NA	NA	1.62	1.31	NA
Gucy1a2	0	2.5	0.48	2.48	0
Gucy1a3	0.7	1.74	0.61	0.67	0.03
Gucy1b2	0.56	NA	NA	0.84	NA
Gucy1b3	0.06	1.61	0.85	1.07	0.01
Gucy2c	0.7	1.76	0.12	2.71	0
Gucy2d	NA	NA	NA	4.53	NA
Gucy2e	0	NA	NA	NA	NA
Gucy2f	0.29	NA	NA	4.74	NA
Guf1	NA	0	1.01	0.26	2.79
Guk1	0.73	1.45	1.2	0	0.82
Gulp1	0.7	0.28	0.24	2.03	2.09
Gusb	NA	1.19	0.84	2.23	0
Gvin1	NA	NA	NA	NA	0.83
Gxylt1	NA	NA	2	2.7	NA
Gxylt2	NA	NA	0.83	5.06	NA
Gyltl1b	0.06	NA	1.98	2.21	3.15
Gypa	0	1.47	0	1.31	3.04
Gypc	0.41	0	2.33	1.89	0.36
Gys1	0	1.15	1.57	0.97	0
Gys2	1.41	2.94	1.3	3.57	1.34
Gzf1	NA	NA	0.96	0.71	0.61
Gzma	1.25	0.87	2.06	0	3.34
Gzmb	1.69	2.08	2.32	0.57	9.51
Gzmk	0	0.68	0.91	0.68	4.16
Gzmm	1.18	0	3.88	2.96	0
H19	0.33	NA	0.58	NA	0
H1f0	0.85	2.33	1.94	1.03	0.87

H1fnt	NA	NA	1.62	2.65	0
H1foo	6.02	NA	0.81	2.45	NA
H1fx	0	0	0	1.35	0
H2afb1	NA	3.85	0.55	0.69	NA
H2afj	NA	0.71	0.4	2	1
H2afv	0.48	0	4.23	0	0
H2afx	NA	1.13	2.92	1.13	2.47
H2afy	0.24	0.02	0.15	0.96	0
H2afy2	NA	0.74	0	1.72	1.16
H2afz	0.37	0.86	1.94	0.26	0
H2bfm	NA	NA	0.08	0.7	NA
H3f3b	0.6	0.24	0.73	1.1	0
H6pd	1.24	0.85	1.36	0	3.2
Haao	1.5	3.4	0.92	2.25	1.53
Habp2	0.84	1.59	0.9	0.51	0.01
Habp4	0	0.6	1.11	0.96	0.74
Hace1	2.45	NA	0.29	0.3	0
Hacl1	NA	NA	2.22	0.69	0
Hadh	NA	0	1.52	1.1	0
Hadha	0.61	0	0.4	0.57	4.55
Hadhb	NA	0.99	0.34	2.61	0.99
Hagh	0.14	0.64	3.35	1.72	0
Haghl	0	NA	1.95	3.86	1.93
Hak	1.3	NA	NA	NA	NA
Hal	6.47	0.95	2.44	3.89	1.26
Hamp	5.74	2.28	1	0.82	8.62
Hand1	0.52	1.81	0.05	1.16	2.14
Hand2	1.53	2.2	0	2.33	2.24
Hao1	0.78	2.42	0.29	3.05	5.12
Hao2	NA	0.13	1.01	0	0
Hap1	0.16	0.66	0.85	0.19	2.54
Hapln1	0.5	0.46	1.02	3.66	1.25
Hapln2	1.99	0.02	0	1.04	0
Hapln3	0.62	NA	0	0.66	1.71
Hapln4	0	NA	3.52	6.17	0.1
Harbi1	NA	NA	1.98	0.71	3.72
Hars	0.32	2.54	1.05	0.61	0
Hars2	0.97	0.12	1.3	0	1.75
Has1	1.24	0.05	0.46	0.97	3.46
Has2	2.6	0.21	0.82	1.45	1.13
Has3	6.03	NA	1.41	NA	3.72

Hat1	0.3	0.01	5.03	0.76	0
Haus1	NA	NA	1.89	0.02	2.9
Haus2	NA	1.11	0.07	0.66	1.21
Haus3	NA	3.11	1.48	NA	0
Haus4	NA	2.93	2.87	1.38	1.41
Haus5	NA	0	1.86	1.47	0.79
Haus6	NA	0.29	0.8	0.53	0
Haus7	NA	2.39	1.88	0	NA
Haus8	NA	NA	2.67	0.93	0.85
Havcr1	1.85	1.08	0.54	0	0
Havcr2	0.12	NA	2.55	0.89	0
Hax1	NA	1.01	3.63	0	0
Hbegf	NA	0.17	2.35	0.28	1.68
Hbp1	0.19	0.91	1.56	NA	0
Hbq1	0.63	NA	NA	NA	NA
Hbs1l	0.57	0.83	1.2	1.9	1.37
Hbxip	3.77	NA	NA	NA	4.06
Hccs	0.48	0.19	1.22	0.48	0
Hcfc1	0.08	0	0.81	1.6	0.65
Hcfc1r1	0	0	1.04	NA	0
Hcfc2	NA	0.72	0.77	0.21	1.2
Hck	0	0.62	4.53	1.95	0
Hcls1	0	3.06	2.15	0	0
Hcn1	0	NA	2.01	0	NA
Hcn2	2.19	1.39	1.76	1.01	0
Hcn3	0.04	NA	1.41	1.29	2.83
Hcn4	3.49	NA	NA	0.16	NA
Hcrt	3.52	0.12	2.78	0.5	4.74
Hcrtr1	2.88	2.14	2.11	0	NA
Hcrtr2	19.42	0.09	1.28	0.73	0
Hcst	0	NA	2	1.92	2.35
Hdac1	0.52	1.13	1.81	0.79	0
Hdac10	NA	NA	1.69	2.58	1.56
Hdac11	0	1.69	3.22	2.06	1.94
Hdac2	0	0.91	1.3	0.93	3.21
Hdac3	0	0	2	2.28	3
Hdac4	NA	0	1.74	1.66	1.43
Hdac5	0.4	0	2.01	0.4	0.22
Hdac6	1.09	0.98	1.89	0.73	3.16
Hdac7	NA	2.96	1.59	1.86	0.89
Hdac7a	0	NA	NA	NA	NA

Hdac8	0	NA	0.64	0.72	3.05
Hdac9	0.58	0	0.29	2.36	0
Hdc	0.89	4.37	0.65	0.67	10.11
Hddc2	NA	2.24	1.35	0	0
Hddc3	NA	NA	0.78	0.81	0
Hdgf	0.54	1.75	0.14	2.93	4.91
Hdgfl1	NA	NA	1.06	1.11	3.36
Hdgfrp2	NA	NA	0.57	0.89	NA
Hdgfrp3	0.08	1.2	1.47	NA	0.63
Hdhd1a	NA	NA	NA	NA	0
Hdhd2	NA	NA	1.22	0.69	0
Hdhd3	0	0.31	3.32	0	0
Hdlbp	1.93	0.47	0.19	1.83	0.83
Hdx	NA	NA	NA	1.9	NA
Heatr1	NA	1.19	0.68	0.85	0
Heatr2	NA	0	0.53	NA	1.69
Heatr3	NA	1.49	2.23	0	0
Heatr5a	NA	NA	1.34	1.37	0
Heatr5b	NA	NA	0.99	1.32	2.08
Heatr6	NA	1.02	1.11	2.07	3.18
Heatr7a	NA	NA	NA	NA	1.13
Heatr7b2	NA	NA	NA	NA	2.31
Hebp1	0	1.43	2.88	2.36	0
Hebp2	0.97	1.85	1.95	1.67	1.18
Heca	NA	0.83	1.32	1.16	0.08
Hectd1	0.14	NA	1.27	1.09	3.27
Hectd2	0.35	NA	1.7	1.1	0.86
Hectd3	NA	0.63	1.17	0	0
Hecw1	NA	1.22	0.4	0.34	1.96
Hecw2	NA	NA	0.82	2.31	0.99
Heg1	NA	0	4.84	0.74	0
Hel308	0	NA	NA	NA	NA
Helb	0.83	NA	0.36	0	NA
Hells	0.34	1.48	2.25	0	0.11
Helq	NA	NA	1.38	0.17	0.68
Helt	0	NA	NA	0.96	NA
Helz	1.11	0	1.27	0.88	0.1
Helz2	NA	NA	1.13	0.12	NA
Hemgn	0.35	NA	1.33	1.62	0.17
Hemk1	1.4	0	0.56	3.66	0
Henmt1	NA	NA	0	0	NA

Hepacam	NA	NA	1.13	NA	2.05
Hepacam2	NA	NA	2.04	0	0.56
Heph	0.46	0.02	3.77	0.79	0.06
Hephl1	NA	NA	NA	0.96	NA
Herc1	0.08	0	1.25	0	1.41
Herc2	0.7	0.68	0.56	0	0.83
Herc3	1.1	2.68	0.37	2.06	3.87
Herc4	NA	0	1.9	0.89	0.09
Herc5	1.82	NA	NA	NA	0.06
Herc6	NA	1.82	1.85	0	NA
Herpud1	0.48	0.02	3.21	0.17	2.83
Herpud2	NA	NA	2.71	1.81	1.22
Hes1	1.63	0.97	3.44	0	0.78
Hes2	6.92	0.78	0.06	0.79	0
Hes3	0	NA	NA	2.8	NA
Hes5	0	NA	0.94	1.49	1.48
Hes6	0.11	NA	2.44	2.16	1
Hes7	4.64	NA	8.03	0.74	0
Hesx1	4.1	1.75	4.88	0.66	4.15
Hexa	0	0	0.62	0.46	0
Hexb	0	1.93	2.35	1.5	0
Hexdc	NA	NA	1.55	0.6	0.17
Hexim1	NA	0.4	1.01	0.83	1.64
Hexim2	NA	NA	0	2.95	NA
Hey1	0	3.17	2.64	0.24	0
Hey2	5.51	1.57	0.99	1.18	3.97
Heyl	0	1.47	0.58	1.61	0
Hfe	2.05	1.28	2.01	0	2.62
Hfe2	NA	NA	1.51	1.59	0.3
Hfm1	NA	NA	NA	0.19	NA
Hgd	0.54	0.03	1.53	0	2.83
Hgf	0	0	1.18	2.2	0
Hgfac	0.57	2.44	0.49	3.56	1.43
Hgs	0	1.29	1.08	3.49	4.8
Hgsnat	NA	0.71	1.03	0.36	0
Hhat	NA	0	1.69	1.17	1.36
Hhatl	NA	NA	0.17	1.32	0
Hhex	0.19	3.5	1.68	0.83	3.8
Hhip	0.27	NA	0.72	0	1.22
Hhipl1	NA	NA	0.7	1.4	NA
Hhipl2	NA	0.97	2.06	3.19	0

Hhla1	NA	NA	NA	0.13	NA
Hiat1	0	NA	2.82	0.58	2.47
Hiatl1	NA	NA	0.95	0.47	0
Hibadh	0.65	NA	0.98	0.86	1.25
Hibch	0	0.98	0.06	0.96	0
Hic1	0.51	0	1.09	2.37	0
Hic2	0.91	0.94	0.31	3.04	0.19
Hid1	NA	NA	0.37	0.55	NA
Hif1a	0.15	1.29	2.08	1.23	0
Hif1an	0.87	1.5	0.85	0.23	0.6
Hif3a	0	0.98	0	0.15	1.7
Higd1a	NA	1.52	2.44	2.07	0
Higd1b	NA	1.92	0.74	2.81	0
Higd2a	NA	0	0.92	0	0
Hilpda	NA	1.35	2.07	7.12	NA
Hils1	0.78	NA	1.68	2.29	0
Hinfp	NA	1.09	2.03	2.75	NA
Hint1	0.22	0.67	1.92	0	0
Hint2	0.34	NA	0.7	0.89	0
Hint3	0	NA	2.45	2.13	0
Hip1	0.65	0.01	1.32	0	3.3
Hip1r	2.09	0	0.43	0	NA
Hip2	0	NA	NA	NA	NA
Hipk1	0.29	1.33	0.81	1.4	0
Hipk2	0.56	0	3.15	1.04	0
Hipk3	0	0.72	0.8	0.46	0
Hipk4	NA	NA	NA	2.09	NA
Hira	1.27	0	0.67	2.76	1.42
Hirip3	NA	1.16	2.52	0.35	2.97
Hist1h1a	0.91	NA	NA	1.61	NA
Hist1h1b	1.53	NA	NA	2.15	NA
Hist1h1c	0.07	1.82	1.74	3.42	0.75
Hist1h1d	0	NA	NA	0.99	NA
Hist1h1e	0.66	2.46	0.02	NA	0
Hist1h1t	1.11	3.54	2.8	0.98	3.27
Hist1h2aa	6.32	NA	NA	0.78	NA
Hist1h2ab	2.43	NA	NA	1.32	NA
Hist1h2ac	1.04	NA	NA	7.12	NA
Hist1h2ad	1.13	NA	NA	NA	NA
Hist1h2ae	0.93	NA	NA	1.46	NA
Hist1h2ag	0.84	NA	NA	NA	NA

Hist1h2ah	2.57	NA	NA	NA	NA
Hist1h2ai	0.92	NA	NA	NA	NA
Hist1h2ak	2.13	NA	NA	16	NA
Hist1h2ba	5.12	NA	NA	0.7	NA
Hist1h2bb	0.72	NA	NA	0.8	NA
Hist1h2bc	0.17	2.16	1.01	1.91	0.66
Hist1h2be	0.18	0	0	0	5.53
Hist1h2bf	0.66	NA	NA	NA	NA
Hist1h2bg	0.92	NA	NA	2.32	NA
Hist1h2bh	1.18	NA	NA	0.62	NA
Hist1h2bj	0.27	NA	NA	NA	NA
Hist1h2bk	0.05	NA	NA	NA	NA
Hist1h2bl	0.36	NA	NA	NA	NA
Hist1h2bm	0.68	NA	NA	1.02	NA
Hist1h2bn	0.15	NA	NA	2.89	NA
Hist1h3a	NA	3.76	0.93	15.57	NA
Hist1h3b	2.39	NA	NA	NA	NA
Hist1h3c	1.22	NA	NA	NA	NA
Hist1h3d	0.25	3.21	0.76	3.57	NA
Hist1h3e	1.09	NA	NA	NA	NA
Hist1h3f	0.64	4.3	0	0.92	1.7
Hist1h3g	2.23	NA	NA	NA	NA
Hist1h3h	0.81	2	3.08	NA	NA
Hist1h3i	3.9	1.43	0.41	4.04	NA
Hist1h4a	0.84	NA	NA	NA	NA
Hist1h4b	0.85	1.69	1.24	NA	NA
Hist1h4c	0	NA	NA	0.92	NA
Hist1h4d	2.03	NA	NA	1.22	NA
Hist1h4f	0.53	NA	NA	1.7	NA
Hist1h4h	0.18	2.3	1.15	0.97	1.29
Hist1h4i	0.2	NA	NA	0.51	NA
Hist1h4j	NA	1.29	0.49	NA	1.39
Hist1h4k	0	NA	NA	NA	NA
Hist2h2ab	0.48	NA	NA	1.5	NA
Hist2h2ac	1.1	NA	NA	NA	NA
Hist2h2be	1.17	3	0.7	0	7.1
Hist3h2a	2.37	1.25	2.06	0.5	0.36
Hist3h2bb	0	NA	NA	NA	NA
Hist4h4	NA	NA	NA	1.02	NA
Hivep1	0.51	1.31	1.67	1.06	0
Hivep2	0	0.08	1.82	2.17	0

Hivep3	1.02	1	1.41	0	0.78
Hjurp	NA	0.64	2.56	0	1.99
Hk1	0.67	0.43	3.3	0.01	0
Hk2	0.26	1.83	3.05	0	1.2
Hk3	0.98	0	1.13	0.99	0
Hkdc1	NA	0	0.59	2.55	2.17
Hlcs	0	0.97	0.46	2.82	1.61
Hlf	0.98	0.7	0.93	0	0
Hltf	NA	1.78	0	0.5	2.05
Hlx	NA	0.88	1.42	0.49	0
Hlx1	1.26	NA	NA	NA	NA
Hlxb9	2.12	NA	NA	NA	NA
Hmbox1	NA	0.91	1.66	0.9	0.97
Hmbs	0.13	1.77	0.79	1	0
Hmcn1	NA	NA	0.12	0	2.55
Hmg20a	0	0	0.9	0.56	0
Hmg20b	0.59	0.02	1.54	0.12	1.82
Hmga1	0.43	NA	NA	0	NA
Hmga2	0.56	0.77	0.44	1.59	0.78
Hmgb1	NA	1.48	0.92	1	0
Hmgb2	2.97	0.01	1.52	0	0
Hmgb3	0.6	1.57	1.96	1.03	0
Hmgb4	NA	NA	1.3	4.78	18.09
Hmgcl	0	0.7	0.8	0.26	0
Hmgcll1	2.41	NA	0.62	1.39	0
Hmgcr	0.74	0.59	0.83	0.01	0
Hmgcs1	0.47	0.71	0.94	0	0.53
Hmgcs2	1.03	1.88	1.22	1.18	2.07
Hmgn1	NA	0	1.01	0	0
Hmgn2	0.47	NA	NA	1.66	7.42
Hmgn3	1.08	2	1.97	1.63	7.09
Hmgn5	NA	0.56	2.22	0.95	NA
Hmgxb3	NA	0	0.09	2.48	1.29
Hmgxb4	NA	0.46	0.28	0.44	0
Hmha1	NA	0.09	2.71	0.03	3.29
Hmmr	0.74	0	2.41	2.53	0
Hmox1	1.16	2.32	1.53	0	5.56
Hmox2	0.12	3.11	3.4	NA	0
Hmx1	0	1.42	2.55	1.8	0
Hmx2	NA	NA	4.95	0	0
Hmx3	1.3	NA	NA	1.72	NA

Hn1	0.53	2.55	1.23	0.96	1.35
Hn1l	NA	0.87	2.23	0.67	0.96
Hnf1a	NA	2.19	1.89	0.95	0
Hnf1b	NA	0.81	0.87	1.36	0
Hnf4a	2.24	0.92	0.79	0.76	2.42
Hnf4g	1.02	0.31	1.05	0.63	0.67
Hnmt	0	0	0.99	0	2.64
Hnrnpa0	NA	2.61	2.73	0	0
Hnrnpa1	NA	1.9	1.53	NA	0
Hnrnpa2b1	NA	2.42	0.45	2.52	0.03
Hnrnpa3	NA	1.83	0	0.66	1.55
Hnrnpab	NA	2.2	1.22	0.04	0
Hnrnpc	NA	0.97	0.36	0.72	0
Hnrnpd	NA	0	1.19	0	0
Hnrnpf	NA	1.85	0.4	0.51	2.66
Hnrnph1	NA	1.67	0.15	0	0
Hnrnph2	NA	0.2	1.43	0.67	0
Hnrnph3	NA	0.12	1.97	2.62	0.48
Hnrnpk	NA	1.89	5.68	0.08	0
Hnrnpl	NA	0.64	1.4	1.62	1.89
Hnrnpm	NA	0.57	0.82	NA	0
Hnrnpr	NA	1.47	0.31	0.35	1.92
Hnrnpu	NA	0.99	0.87	0.01	0
Hnrnpul1	NA	0	4.09	0.23	0
Hnrnpul2	NA	0.03	0.47	0	1.13
Hnrpa0	0.07	NA	NA	NA	NA
Hnrpa1	0	NA	NA	NA	NA
Hnrpa2b1	0	NA	NA	NA	NA
Hnrpab	0.06	NA	NA	NA	NA
Hnrpc	1.45	NA	NA	NA	NA
Hnrpd	3.09	NA	NA	NA	NA
Hnrpdl	0	NA	NA	NA	0
Hnrpf	0	NA	NA	NA	NA
Hnrph1	0.04	NA	NA	NA	NA
Hnrph2	2.26	NA	NA	NA	NA
Hnrpk	0.18	NA	NA	NA	NA
Hnrpl	3.19	NA	NA	NA	NA
Hnrpll	NA	NA	NA	NA	1.57
Hnrpm	0.4	NA	NA	NA	NA
Hnrpr	0.81	NA	NA	NA	NA
Hnrpu	1.09	NA	NA	NA	NA

Hnrpul1	0.91	NA	NA	NA	NA
Hnt	0.43	NA	NA	NA	NA
Hoga1	NA	NA	4.9	0	NA
Homer1	0.91	1.29	3.89	1.26	0.89
Homer2	0.54	1.53	0.89	0.6	0
Homer3	0	3.25	1.65	NA	1.08
Homez	0	NA	0	NA	0.7
Hook1	0.77	0.38	0.93	0	0
Hook2	0.57	0	1.9	4.6	0.07
Hook3	0	NA	0.87	0	1.23
Hopx	NA	1.75	0.35	0.7	8.09
Hormad1	NA	NA	0.53	0	NA
Hormad2	NA	NA	2.33	0.65	0
Hoxa1	0.86	1.51	2.11	0	1.43
Hoxa10	0.46	0	0.98	0	0
Hoxa11	0.52	1.3	0.84	1.33	3.15
Hoxa11as	NA	NA	NA	NA	3.78
Hoxa13	1.15	NA	1.79	3.29	0
Hoxa2	0.79	1.99	0	0	8.61
Hoxa3	0.65	0.24	0	0.82	1.51
Hoxa4	1.42	0.53	1.34	0.39	2.6
Hoxa5	0.74	1.01	0	0.9	2.9
Hoxa6	0.64	NA	NA	2.45	NA
Hoxa7	0.4	3.3	3.3	0	0
Hoxa9	0.66	NA	NA	0	0.13
Hoxb1	1.53	1.47	0.03	0.53	4.67
Hoxb13	3.84	2.1	0	0	0
Hoxb2	0.05	2.75	1.09	0	0
Hoxb3	0.45	0	1.92	0.06	0.79
Hoxb4	0.57	NA	2.23	0.9	2.16
Hoxb5	2.82	1.37	2.25	0	3.13
Hoxb6	0.52	0	1.43	0.97	0
Hoxb7	0.86	2.05	2.8	0.02	3.14
Hoxb8	2.26	0.35	1.22	0.07	NA
Hoxb9	0.27	0	0.65	0.41	0
Hoxc10	0	1.29	1.94	7.12	2.14
Hoxc11	NA	NA	NA	1.89	NA
Hoxc12	0	NA	NA	1.32	NA
Hoxc13	3	0.14	0.16	1.01	0
Hoxc4	0.9	1.66	0.04	NA	0
Hoxc5	0.06	0.59	1.47	1.41	1.65

Hoxc6	0	1.13	1.17	0.48	0
Hoxc8	1.46	0.93	1.9	0.57	2.87
Hoxc9	1.29	NA	2.11	0	1.3
Hoxd1	2.11	2.44	0.71	2.15	4.25
Hoxd10	0.4	2.01	0.88	2.09	0
Hoxd11	0.98	0.92	0.65	0.94	0.27
Hoxd12	0	0.84	0.95	1.51	0
Hoxd13	0.58	1.13	0.42	1.94	0.04
Hoxd3	4.98	0.98	0.03	0.09	2.93
Hoxd4	1.02	NA	1.66	0	0
Hoxd8	0.02	NA	1.52	1.18	1.33
Hoxd9	0.84	2.18	0	1.66	1.51
Hp	1.95	0	1.12	2.54	0
Hp1bp3	1.02	2.51	1.97	0.53	0
Hpca	1.84	2.8	0.95	1.82	0.99
Hpcal1	0	2.61	1.95	1.24	0
Hpcal4	0.96	0.44	0.99	0	2.62
Hpd	2.22	2.48	0	2.86	7.29
Hpdl	NA	NA	0.94	1.8	2.28
Hpgd	NA	0.84	2.19	4.28	4.89
Hpgds	NA	2.13	3.74	0.39	1.37
Hpn	2.65	2.36	1.98	1.17	0.05
Hps1	0.12	0.8	1.12	0.72	1.06
Hps3	0	NA	0.59	0.98	3.19
Hps4	0.45	0	3.92	1.53	0.02
Hps5	0	0.06	3.14	0.92	3.5
Hps6	0.03	1.68	1.04	3.5	0
Hpse	0	3.06	0.44	0.46	0
Hpse2	NA	NA	NA	0.01	NA
Hpx	NA	1.04	0.28	1.37	10.88
Hr	0.6	1.8	2.53	0.66	0
Hrasls	2.98	1.02	0	2.6	1.22
Hrasls3	3.96	NA	NA	NA	NA
Hrasls5	NA	NA	2.36	0.8	0.26
Hrb	0.53	NA	NA	NA	NA
Hrbl	1.12	NA	NA	NA	NA
Hrc	2.22	0.39	0	2.63	6.27
Hrct1	NA	NA	0.95	0.42	3.04
Hrg	0.78	1.24	1.98	0	4.55
Hrh1	0.91	1.28	0.21	1.82	1.37
Hrh2	0.47	0.52	0.4	1.11	0

Hrh3	3.3	0	1.13	1.39	0
Hrh4	0.76	1.32	1.43	0.48	0
Hrk	NA	1.03	1.58	2.03	0
Hrnr	1.27	NA	NA	1.78	NA
Hrsp12	0	2.34	2.73	1.45	1.69
Hs1bp3	0	1.8	1.34	2.28	0.73
Hs2st1	0.07	1.2	0.85	0	1.29
Hs3st1	0	1.43	2.24	1.17	0.15
Hs3st2	3.05	1.13	0.12	1.43	1.07
Hs3st3a1	0	1.55	0.1	2.18	4.1
Hs3st3b1	0.24	1.74	0.42	0.79	1.16
Hs3st4	NA	NA	0.78	NA	1.8
Hs3st5	NA	NA	0.52	0	NA
Hs3st6	NA	NA	1.4	0.52	10.67
Hs6st1	1.58	0.63	4.63	0.89	NA
Hs6st2	0.42	NA	0.84	0.21	0.52
Hs6st3	2.55	NA	1.23	0.28	5.05
Hsbp1	0	3.87	1.28	0	0.2
Hsbp1l1	NA	NA	0	NA	NA
Hscb	NA	NA	2.06	0.02	0
Hsd11b1	0.12	2.13	1.61	0.24	0
Hsd11b2	0	1.37	0	1.32	8.78
Hsd17b1	0	0.02	0	1.01	1.34
Hsd17b10	NA	1.56	0.66	0	0
Hsd17b11	0.37	0.77	0.77	1.55	1.07
Hsd17b12	0	1.78	1.45	0	1.03
Hsd17b13	5.39	NA	NA	0	NA
Hsd17b14	NA	1.76	0.76	2.81	1.1
Hsd17b2	2.82	2.17	1.44	0.44	0
Hsd17b3	0.65	0.58	0.62	1.62	4.54
Hsd17b4	1.36	0	1.12	0.72	0.08
Hsd17b6	NA	0.73	0.74	2.58	1.5
Hsd17b7	1.69	0.88	1.8	NA	1.77
Hsd3b1	4.14	0.76	0.29	0.43	2.98
Hsd3b2	1.03	1.33	0.4	1.89	0
Hsd3b7	0	NA	1.51	1.09	1.49
Hsdl1	NA	NA	4.33	1.72	0.78
Hsdl2	NA	3.27	0.79	1.74	0
Hsf1	1.98	1.48	1.21	0	1.22
Hsf2	0.65	0	1.23	1.06	0
Hsf2bp	0	1.2	0	2.3	1.01

Hsf4	0	3.34	0.28	NA	0
Hsf5	NA	NA	1.25	0	1.53
Hsfy2	NA	NA	0.51	1.76	NA
Hsh2d	4.22	NA	1.67	1.43	NA
Hsp90aa1	NA	1.59	0.42	0.8	0
Hsp90ab1	NA	0	0.52	1.63	0.54
Hsp90b1	NA	1.5	1.44	0.11	1.25
Hspa12a	0.67	1.11	0.88	0	0
Hspa12b	0.39	NA	0.74	0.84	2.82
Hspa13	NA	0.26	1.21	0	0
Hspa14	0.68	0.18	0.32	0.93	2.15
Hspa1a	0.68	0.71	1.99	1	3.67
Hspa1b	0.47	1.31	1.6	NA	NA
Hspa1l	1.02	1.03	1.97	0	5.62
Hspa2	1.38	0.91	2.28	2.05	0
Hspa4	0	0.44	0.74	0	0
Hspa4l	NA	0.65	1.08	0.3	2.24
Hspa5	7.93	1.89	0.96	0	1.43
Hspa8	0	2.16	0	0.73	0
Hspa9	NA	0.46	0.9	1.99	0
Hspb1	0.64	1.14	1.77	5.74	1.39
Hspb11	NA	0.17	2.5	0.09	0
Hspb2	0.31	1.8	0.85	NA	3.23
Hspb3	0.83	1.27	0.05	2.3	0
Hspb6	NA	1.97	3.25	2.1	4.15
Hspb7	2.72	1.11	0.12	1.29	1.94
Hspb8	1.14	1.51	0.43	0	1.4
Hspb9	3.8	NA	1.82	0.98	2.19
Hspbap1	0	0.9	1.13	1.82	2.89
Hspbp1	NA	1.77	1.34	1.21	0
Hspc171	0.4	NA	NA	NA	NA
Hspd1	0.01	1.44	0.68	0.67	1.16
Hspe1	0	0.29	0.43	0.38	1.05
Hspg2	0.91	1.27	0.94	NA	0
Hsph1	NA	1.96	1.23	0.57	0
Hszfp36	0.31	NA	NA	NA	NA
Htatip	0.67	NA	NA	NA	NA
Htatip2	0	2.37	0.75	0	0.8
Htatsf1	0.19	0.48	2.15	1.2	1.58
Htf9c	1.22	NA	NA	NA	NA
Htr1a	0.24	1.65	0.96	0.7	3.24

Htr1b	0	0.83	0.29	3.83	4.33
Htr1d	16.79	1.49	0	0.36	3.11
Htr1f	2.79	1.5	0.68	2.43	0
Htr2a	2.1	NA	NA	0.17	NA
Htr2b	0.11	2.62	0.32	0.29	0
Htr2c	2.48	0	1.14	0	1.37
Htr3a	1.83	2.28	1.85	5.63	3.37
Htr3b	0.86	2.35	0	0.69	0
Htr4	0	0	0.01	1.44	0
Htr5a	0	0.04	1.46	2.73	2.39
Htr6	2.09	1.93	1.43	0.59	2.2
Htr7	0	1.09	0.39	0.89	0.1
Htra1	NA	0.07	2.72	1.43	0
Htra2	NA	0	0.82	NA	3.06
Htra3	1.7	NA	0.85	0.04	1.75
Htra4	NA	NA	0	0.4	NA
Htt	NA	0.82	1.09	0	0
Hunk	2.19	0	2.54	0.69	0
Hus1	0.62	0.73	0.43	1.99	2.15
Hus1b	0.2	NA	NA	2.6	NA
Huwe1	NA	0.37	0.92	1.83	0.12
Hvcn1	NA	NA	0.7	NA	0
Hyal1	0.28	NA	NA	NA	NA
Hyal2	0.41	4.77	0.9	2.63	0
Hyal3	0	1.54	5.59	NA	4.64
Hyal4	0	2.47	2.2	1.13	0
Hydin	2.64	1.15	0.43	NA	1.54
Hyi	NA	1.46	0.54	0.14	1.03
Hyls1	NA	NA	0.83	0.36	0
Hyou1	0	1.82	0.34	0	2.11
Hypk	NA	2.08	0.55	0.45	NA
Iah1	NA	NA	2.11	0	0
Iapp	2.52	0.97	1.07	0.46	1.46
Iars	0.25	2.54	5.74	1.3	0
Iars2	NA	0	1.9	0.18	5.44
Iba57	NA	2.06	0.03	0.82	NA
Ibrdc2	1.2	NA	NA	NA	NA
Ibrdc3	0	NA	NA	NA	NA
Ibsp	1.77	1.06	0.76	0.94	0
Ibtk	0	0.5	1.65	0	3.64
Ica1	0	0.71	1.76	0.67	0

Ica1l	NA	NA	2.64	6.27	2.29
Icam1	0.17	0.59	3.3	1.95	0
Icam2	0.25	0	1.73	0.11	0
Icam4	0	1.34	0.36	0.68	2.57
Icam5	0	4.74	0.34	1.14	0
Ick	0.58	0	0.77	1.47	0
Icmt	0.24	0.86	1.03	NA	0
Icos	0.54	0	0.13	2.44	0.96
Ict1	0.07	1.36	0	0.51	0
Id1	NA	1.3	4.3	0.39	0.09
Id2	NA	2	1.22	0.26	3.15
Id3	NA	0	0.15	1.13	2.43
Id4	NA	0.61	0.64	0	1.95
Ide	1.66	0.19	1.57	0	0
Idh1	0.12	2.16	1.27	2.75	2.6
Idh2	0	0	1.01	0.63	0
Idh3a	0	1.15	0.03	0.6	0
Idh3b	0.16	0	2.82	0.99	0
Idh3g	0.33	0	0.54	1.48	0
Idi1	0.74	0	1.57	1.05	1.59
Idi2	NA	NA	2.04	0.26	1.71
Idnk	NA	NA	0.81	1.44	NA
Ido1	NA	0.43	4.18	0.38	10.69
Ido2	NA	NA	0.98	1.04	NA
Ids	0.31	0.18	1.93	0.63	0
Idua	0.83	0.23	0.02	0	0
Ier2	0.86	2.86	1.79	2	0
Ier3	0.4	0.55	2.08	0.02	0
Ier3ip1	NA	1.25	0.93	0.54	0
Ier5	4.18	1.15	1.14	2.22	0
Ier5l	0.27	NA	1.75	1.24	1.16
Iffo1	NA	0.22	0.95	1.52	0
Iffo2	NA	NA	0.18	1.89	0
Ifi16	0.1	NA	NA	NA	NA
Ifi27l1	NA	NA	1.95	0.31	0
Ifi30	5.86	0	0.76	1.45	0.06
Ifi35	0.06	0.05	1.49	0	0
Ifi44	NA	0.4	0.04	0	0
Ifi44l	NA	0.85	1.59	0	NA
Ifih1	0.06	0.86	0.73	0	0
Ifit1	0	2.17	1.05	0	0

Ifit2	0.01	1.33	1.12	0	0
Ifit3	0	1.35	1.97	0	0
Ifitm1	0	0.2	0.3	0.55	0.31
Ifitm10	NA	NA	0.78	0.92	NA
Ifitm2	0.08	1.17	0.66	0.17	0
Ifitm3	0	0.44	0.82	0.77	0
Ifitm5	1.1	NA	NA	1.06	NA
Ifltd1	NA	NA	1.55	7.36	NA
Ifna1	1.42	2.04	1.61	NA	3.39
Ifna13	1.62	NA	NA	1.64	NA
Ifna14	0.77	NA	NA	0	NA
Ifna2	0.79	2.29	0.98	0	1.67
Ifna4	0.87	1.6	1.94	NA	2.6
Ifna5	0.92	3.13	2.18	NA	2.86
Ifna6	0.64	NA	NA	0	NA
Ifna7	0.58	NA	NA	NA	NA
Ifnar1	0.22	0	1.29	0.46	0
Ifnar2	0.28	0	0.81	0.61	2.34
Ifnb1	0	2.37	2.07	1.99	0
Ifne	NA	NA	NA	6.55	NA
Ifng	0	0.43	0	0.27	5.84
Ifngr1	0.53	0	3.23	0.35	0
Ifngr2	0.13	0.4	1.57	0.42	0
Ifnk	0.32	NA	NA	0.33	NA
Ifnl3	NA	NA	NA	0	NA
Ifnlr1	NA	NA	0.5	2.23	NA
Ifrd1	0.56	1.45	1.84	0	1.89
Ifrd2	0	0	1.41	1.58	1.31
Ifrg15	1.06	NA	NA	NA	NA
Ift122	NA	0.05	0.77	0.34	0.42
Ift140	NA	0.68	1.82	0	1.34
Ift172	1.33	NA	1.7	0	2.15
Ift20	NA	2.64	3.44	0.79	0
Ift27	NA	0.44	1.66	1.2	NA
Ift43	NA	NA	2.11	0.38	NA
Ift46	NA	2.26	1.19	0	NA
Ift52	NA	4.01	0.67	1.41	1.12
Ift57	NA	0	0.19	0.18	2.18
Ift74	NA	2.06	0.59	1.37	0
Ift80	NA	NA	0.01	1.19	0
Ift81	NA	0	0.13	0.67	0.56

Ift88	NA	0	3.28	0.22	0
Igbp1	0.31	1.76	0.82	0.79	2.09
Igdcc3	NA	NA	0.64	2.63	0
Igdcc4	NA	NA	3.09	0	0
Igf1	0.26	0.51	1.83	1.25	2.3
Igf1r	0	0.63	0.51	1.19	0
Igf2	1.15	NA	NA	NA	NA
Igf2as	2.29	NA	NA	NA	6.8
Igf2bp1	0.49	NA	0.61	1.14	1.58
Igf2bp2	NA	2.09	1.32	0.75	0.14
Igf2bp3	0	1.28	1.68	0.44	0
Igf2r	1.9	0.05	0.74	0	0
Igfals	0.29	3.66	4.5	1.74	7.06
Igfbp1	2.93	0.88	1.75	1.21	4.8
Igfbp2	2.51	3.42	0.86	4.49	0.91
Igfbp3	0.85	0	1.08	0	0.72
Igfbp4	0.88	0.96	2.94	0.38	0
Igfbp5	0.56	0	0.9	0.41	1.34
Igfbp6	1.33	4.36	0.26	0	1.66
Igfbp7	0	0.66	1.97	0.15	1.08
Igfbpl1	3.44	NA	0.09	1	0
Igfl3	NA	NA	NA	0.04	NA
Igflr1	NA	2.69	1.31	2.18	NA
Igfn1	NA	NA	NA	1.57	NA
Ighg1	NA	0.62	2.64	NA	0
Ighm	NA	0.13	1.04	0.04	NA
Ighmbp2	1.21	0.76	0.54	0.02	0
Igj	1.51	1.17	1.91	0.09	2.99
Igk	NA	0	4.01	NA	NA
Igkc	NA	NA	NA	0.39	NA
Igll1	NA	0.56	3.19	1.7	0.21
Iglon5	NA	NA	0.1	1.42	3.15
Igsf1	0.9	0	0.35	0.93	0
Igsf10	0.45	NA	0.02	0.26	2.01
Igsf11	0	NA	0.97	1.5	0
Igsf21	NA	NA	1.91	1.42	0
Igsf3	1.43	0.66	0.78	3.2	0.06
Igsf5	NA	NA	0.98	1.15	NA
Igsf6	0.81	0.02	2.12	4	0
Igsf8	2.74	NA	0.71	0	0.03
Igsf9	4.02	NA	1.17	1.71	0

Igsf9b	NA	NA	NA	0.86	NA
Ihh	0.93	2.25	1.9	2.49	2.45
Ihpk1	0.89	NA	NA	NA	NA
Ik	0	2.48	0.87	1.08	2.51
Ikbip	NA	NA	0.67	0.47	NA
Ikbkap	0.41	0	0.24	1.4	0
Ikbkb	0.59	0	1.54	1.99	0
Ikbke	NA	1.03	1.86	0.9	0.05
Ikbkg	0	2.99	4.34	1.08	1.16
Ikzf1	NA	0	1.01	2.07	1.03
Ikzf2	NA	1.52	0.49	1.43	0
Ikzf3	NA	1.73	0	3.13	1.5
Ikzf4	NA	0.62	1.2	1.27	0
Ikzf5	NA	0.41	0.76	0.01	0
Il10	0.11	0	4.34	3.46	0
Il10ra	0.22	0.73	1.71	0.92	2.17
Il10rb	0.06	0	2.31	1.65	0
Il11	0.06	1.14	1.28	3.68	3.32
Il12a	3.73	1.93	2.22	0.01	0.1
Il12b	0	1.1	1.23	1.81	0
Il12rb1	0.93	1.29	1.62	0.97	0.68
Il12rb2	1.06	0	1.86	1.22	6.14
Il13	0.93	2.74	1.32	0	0
Il13ra1	0.69	0.96	5.2	1.88	0
Il13ra2	0.55	0.98	4.07	0	7.92
Il15	0.35	1.41	0.95	0	6.82
Il15ra	0.07	0	0	9.62	0.31
Il16	0	0	0.28	4.03	0
Il17a	NA	0.77	0.31	1.45	5.46
Il17b	2.53	0	0.52	0.96	0
Il17c	1.63	NA	NA	0.86	NA
Il17d	2.61	NA	0.45	2.4	0
Il17f	0.74	NA	NA	5.24	NA
Il17ra	NA	0.32	3.1	1.36	0
Il17rb	2.96	1.3	2.3	2.87	3.21
Il17rc	0.33	0.82	1.62	1.04	0
Il17rd	2.59	NA	0.6	0	0.74
Il17re	0	NA	0.47	1.28	1.47
Il18	0.59	0.14	1.14	1.74	0
Il18bp	0	1.84	0.38	2.91	2.98
Il18r1	0.72	0.01	3.22	0.53	4.4

Il18rap	1.39	0.05	1.21	0	0
Il19	0.38	NA	NA	0.79	NA
Il1a	0.3	0.85	2.18	1.97	0
Il1b	0.5	0.41	0.07	1.13	0
Il1f10	0.93	NA	0.32	1.26	0
Il1f5	1.56	NA	NA	NA	2.52
Il1f6	1.54	NA	NA	NA	0.94
Il1f8	2.86	NA	NA	NA	1.82
Il1f9	4.39	NA	NA	NA	0.07
Il1r1	0.44	0	1.33	1.53	3.04
Il1r2	2.44	0.28	2.2	0	0
Il1rap	0	0.85	1.34	0	0
Il1rapl1	0.65	1.93	0	0	2.25
Il1rapl2	0.51	0.83	0	0.87	2.07
Il1rl1	0.62	0.18	0.42	0.54	0
Il1rl2	2.41	1.24	2.24	0.05	0
Il1rn	0.04	0.2	1.69	0.36	0
Il2	2.21	0.37	1.99	0	1.53
Il20	1.13	NA	6.09	0.23	3.41
Il20ra	NA	0	1.14	1.82	3.92
Il20rb	NA	NA	1.69	0.76	0.6
Il21	0.4	3.26	1.42	2.13	0
Il21r	0	0	0.84	0.22	2.48
Il22	1.67	NA	NA	NA	NA
Il22ra1	0.75	NA	NA	0.54	NA
Il22ra2	1.88	NA	0	0.45	2.97
Il23a	0	2.11	0.41	0.68	0.51
Il23r	3.75	NA	NA	3.51	NA
Il24	0.61	0	2.44	0.27	4.06
Il25	NA	1.11	1.14	1.39	0
Il27	0	NA	NA	2.12	NA
Il27ra	0	0.54	1.4	0	0.73
Il28ra	0.54	NA	NA	NA	7.06
Il2ra	0	1.77	3.41	0.72	1.92
Il2rb	0	0	0.23	0.13	3.54
Il2rg	1.54	1.19	0.47	5.52	0
Il3	0.29	1.51	2.7	1.83	1.84
Il31	NA	NA	NA	1.74	NA
Il31ra	0	NA	2.1	2.1	0.02
Il33	NA	1.5	0	1.42	0.26
Il34	NA	NA	0	0.11	6.22

Il3ra	1.97	1.91	0.47	0.17	0.1
Il4	4.47	1.11	1.36	0.96	0
Il4i1	0.99	NA	NA	NA	NA
Il5	0.79	0.25	4.75	0	0
Il5ra	1.29	0	0.25	6.96	0.04
Il6	0.41	2.89	1.92	0.4	0
Il6st	0.63	0.25	1.45	2.38	0
Il7	1.18	0.97	3.85	1.36	2.67
Il7r	0	0	2.14	1.44	2.41
Il8	NA	0.68	1.61	1.83	NA
Il8ra	0.51	NA	NA	NA	NA
Il8rb	0.01	NA	NA	NA	NA
Il9	0	1.31	3.98	1.97	0
Il9r	0.87	2.05	2.31	0.7	1.33
Ildr1	NA	NA	1.36	2.36	0
Ildr2	NA	NA	0.57	0.44	NA
Ilf2	0	1.44	0.11	0.61	0
Ilf3	0.2	0	1.29	1.61	0
Ilk	0	2.32	4.1	1.38	0
Ilkap	NA	0.12	1.25	NA	1.7
Ilvbl	0	0.06	0	0	0.97
Immp1l	NA	NA	0	0	0
Immp2l	0.39	NA	1.23	0.94	0.19
Immt	0.73	0	0	0	1.64
Imp3	NA	0	1.8	0.69	0
Imp4	NA	1.02	0.25	1.59	2.89
Impa1	2.16	0.8	1.17	0	0.5
Impa2	0	0.01	0.22	0.85	4.37
Impact	0.4	0	0.35	1.15	0
Impad1	NA	1.96	0.67	0.39	0
Impdh1	0.34	4	0.45	2.26	0
Impdh2	0.32	0.87	0.16	0.51	0
Impg1	2.24	1.38	0.81	0.31	0.12
Impg2	0.85	NA	NA	0.77	NA
Ina	1.3	0.98	2.09	1.93	1.99
Inadl	NA	0	1.28	0	0.51
Inca1	NA	NA	NA	1.88	NA
Incenp	3.65	0.77	1.32	0	1.56
Indo	0	NA	NA	NA	NA
Inf2	NA	0.05	0.02	0.94	1.4
Ing1	0	0.36	0.21	1.08	0

Ing2	NA	1.1	2.42	2.3	0
Ing3	0.4	0.7	0.24	0.31	1.59
Ing4	1.04	1.54	1.01	0.65	0.14
Ing5	0	NA	0.75	1.48	0.78
Inha	1	1.87	0.91	4.93	4.01
Inhba	0	0.02	0.17	0.3	0.88
Inhbb	0.41	0	1.96	0.02	NA
Inhbc	0.54	1.79	0.12	0	0
Inhbe	1.27	1.46	1.59	0	2.53
Inip	NA	NA	0.91	0.51	NA
Inmt	NA	NA	0.44	0.43	5.11
Ino80	NA	NA	0.96	1.75	0
Ino80b	NA	0.62	3	1.28	0.06
Ino80c	NA	NA	1.16	0.69	1.35
Ino80d	NA	1	0.5	1.24	2.16
Ino80e	NA	NA	0	1.28	2.48
Inpp1	0.21	1.74	1.66	0.8	4.27
Inpp4a	0.4	0.35	1.71	0	2.25
Inpp4b	1.61	0	0.61	1.74	1.38
Inpp5a	0	0.74	5.72	0	0
Inpp5b	0.97	0	1.04	0	0
Inpp5d	0.88	0	2.2	1.83	0
Inpp5e	1.02	0	4.22	2.49	1.61
Inpp5f	0	0.21	3.36	0	0.23
Inpp5j	NA	2.6	3.95	1.72	3.47
Inpp5k	NA	0	2.62	0.47	0
Inppl1	0.48	1.73	3.14	1.44	1.13
Insc	NA	NA	0	1.47	1.66
Insig1	0.75	1.24	1.83	0.11	0
Insig2	0	0.73	2.13	1.55	2.2
Insl3	1.05	NA	NA	NA	NA
Insl5	0.02	3.29	1.41	0.71	3.06
Insl6	1.72	1.82	1.73	0	0
Insm1	0	1.04	0.85	0.91	1.91
Insm2	0	NA	0.84	0	1.1
Insr	1	1.17	1.14	0	0
Insrr	2.14	0	0.24	NA	5.43
Ints1	NA	0.44	1.4	1.09	0
Ints10	NA	NA	0.69	0.56	0.23
Ints12	NA	0.57	2.14	0.08	3.57
Ints2	NA	NA	1.91	1.44	2.77

Ints3	NA	1.36	0.33	1.76	0
Ints4	NA	NA	1.89	0.7	1.02
Ints5	NA	1.33	0	1.44	1.59
Ints6	NA	1.44	1.94	0	1.11
Ints7	NA	0	0.84	0.44	1.34
Ints8	NA	1.24	0	0.18	0
Ints9	NA	0	0.3	0	1.07
Intu	NA	NA	0.38	0.82	0.64
Invs	0.11	1.32	0.03	0	0.96
Ip6k1	NA	0.74	1.26	0.04	0
Ip6k2	NA	2.66	0.57	0	0.32
Ip6k3	NA	NA	2.91	0.1	2.56
Ipcef1	NA	0.76	0	1.89	4.1
Ipmk	1.45	NA	1.71	0	0.94
Ipo11	0.01	NA	2.14	0.02	0.99
Ipo13	0.11	0	0.82	1.36	3.62
Ipo4	0.35	2.01	2.43	2.33	0.76
Ipo5	NA	1.04	0.33	0.17	1.82
Ipo7	1.38	1.27	0.59	0.68	3.43
Ipo8	0.42	0.73	0.73	0	0
Ipo9	0.46	0	1.51	0.1	1.09
Ipp	1.15	3.95	4.25	NA	0.52
Ippk	NA	2.58	2.99	NA	0
Ipw	NA	1.92	0.66	NA	0.27
Iqca	0.77	NA	NA	NA	NA
Iqcb1	NA	0.79	4.54	0.95	2.65
Iqcc	2.8	3.03	1.94	2.41	3.24
Iqcd	0	NA	0.68	5.54	NA
Iqce	0	1.5	1.08	1.94	0
Iqcf1	3.03	NA	0.35	3.71	NA
Iqcf3	1.62	NA	1.04	NA	0
Iqcf4	NA	NA	0	NA	NA
Iqcf5	NA	NA	0.82	NA	8.64
Iqcg	NA	0.78	0.2	1	0
Iqch	NA	0.98	1.83	5.02	0
Iqck	NA	0.97	0	0.32	0
Iqgap1	0	0.3	0.48	0	0
Iqgap2	NA	0.15	1.65	0	0
Iqgap3	NA	NA	1.75	0	2.98
Iqsec1	NA	2.7	0	1.22	0
Iqsec2	NA	0.09	1.27	0.4	1.84

Iqsec3	0.42	2.49	1.24	0.91	0
Iqub	NA	NA	0.01	0.18	NA
Iqwd1	0	NA	NA	NA	NA
Irak1	0.68	0	1.74	NA	0
Irak1bp1	0	NA	0.81	0.82	4.4
Irak2	0	NA	0.76	0.38	0
Irak3	0.05	0	1.71	0.5	0
Irak4	0.79	0	5.65	0.35	0.9
Ireb2	1.01	0.12	0.79	0.65	0
Irf1	0	1.48	1.23	0.69	0
Irf2	0.66	1.03	2.71	1.24	0.05
Irf2bp1	1	1.04	2.01	0.89	0.13
Irf2bp2	0	NA	1.61	1.64	1.94
Irf2bpl	NA	NA	4.03	0.29	NA
Irf3	0	0	0	0	2.03
Irf4	0.87	0.52	0.97	0	0
Irf5	0.37	0.89	0.24	2.13	0
Irf6	0.84	1.72	1.01	5.55	2.53
Irf7	0.91	0.01	0.36	2.47	0
Irf8	NA	1.92	3.12	0.73	3.16
Irf9	NA	1.23	5.88	0	0
Irg1	NA	NA	NA	0.18	0
Irgq	NA	1.18	0.72	0.73	1.37
Irs1	0	0	0.54	1.27	0.72
Irs2	NA	1.26	1.09	0.86	0
Irs4	1.33	0.96	0	2.29	0.87
Irx1	2.24	NA	0.69	1.32	2.9
Irx2	1.33	NA	1.49	0.88	NA
Irx3	0.97	NA	2.55	0	1.74
Irx4	5.65	1.89	0.84	3.87	12.53
Irx5	0	1.55	1.44	2.18	1.95
Irx6	0.73	NA	NA	2.35	NA
Isca1	NA	1.98	0.84	1.28	0
Isca2	NA	NA	0.86	1.26	0
Iscu	NA	0.62	2.34	0	0.92
Isg15	NA	1	0.23	0.03	NA
Isg20	0.01	1.15	0.53	8.06	1.71
Isg20l2	NA	1.14	2.92	3.04	0.12
Isgf3g	1.16	NA	NA	NA	NA
Isl1	0.37	1.51	0.72	0	0
Isl2	1.51	NA	0.96	0.55	0

Islr	1.56	1.44	0.99	3.4	0
Islr2	NA	NA	0	1.36	0
Ism1	NA	NA	0.42	NA	0.5
Isoc1	NA	2.4	2.05	0.16	0
Ist1	NA	1.17	1.27	0.29	NA
Isx	NA	NA	1.42	0	2.47
Isy1	NA	NA	0.27	0.79	0.83
Isyna1	3.57	3.02	2.28	0	1.25
Itch	2.2	0.69	1.85	0.05	0
Itfg1	NA	2.71	0.45	0.79	0.75
Itfg2	NA	0	0.41	0.96	0
Itfg3	NA	NA	1.08	0.02	0
Itga1	0	0.06	3.75	NA	NA
Itga10	0.98	1.68	1.4	0.06	0
Itga11	0.91	NA	NA	0.06	NA
Itga2	0.73	0.52	0.67	0	1.21
Itga2b	5.83	1.44	1.29	0.36	0
Itga3	4.92	1.19	0	2.06	0
Itga4	0.43	0.01	2.25	NA	0
Itga5	0	2.01	1.47	2.14	4.44
Itga6	2.65	0	2.43	0.97	1.04
Itga7	1.5	3.97	0.89	1.08	0
Itga8	1.28	1.88	1.92	0	1.48
Itga9	0	1.22	0.92	2.97	0.97
Itgad	NA	NA	NA	1.55	NA
Itgae	0.71	2.19	1.14	1.16	4.05
Itgal	0.4	0.23	2.27	1.49	5.68
Itgam	0.37	0	0.77	1.44	0
Itgav	0.54	0	0.93	0	0
Itgax	0.48	1.33	0.29	1.85	1.07
Itgb1	1.32	0.37	0.77	0.64	0.72
Itgb1bp1	1.23	0.59	0.52	0.41	1.98
Itgb1bp2	1.07	1.01	1.73	0	0
Itgb1bp3	0.57	NA	NA	NA	2.82
Itgb2	2.5	2.3	0.92	0	1.25
Itgb3	0.91	1.26	2.18	0	0.9
Itgb3bp	NA	0.45	1.57	NA	0
Itgb4	0.96	1.47	0	2.17	0
Itgb4bp	0.45	NA	NA	NA	NA
Itgb5	1.56	0.96	0.82	1.45	1.86
Itgb6	0.22	0.53	0.66	0.24	0

Itgb7	1.05	0	3.65	0.43	0
Itgb8	0	0.7	3.9	0	0
Itgbl1	0.58	0.93	0.56	0.61	5.11
Itih1	1.24	1.24	0.94	1.37	4.57
Itih2	0.27	0.08	0	1.95	2.13
Itih3	1.93	1.71	0.83	2.57	4.25
Itih4	1.64	2.2	1.22	1.11	5.82
Itih5	0	1.16	0.39	1.69	0
Itk	0.64	0	0.76	1.41	2.29
Itln1	NA	NA	0.5	NA	0
Itln2	1.13	NA	NA	NA	NA
Itm2a	0	0.13	1.99	1.25	0
Itm2b	0.46	0.91	0.75	4.47	0.68
Itm2c	0.13	4.17	1	1.56	3.94
Itpa	0.44	2.17	1.64	0.74	4.42
Itpk1	0	0.81	1.96	0.27	1.25
Itpka	4.98	2.1	2.82	2.76	0
Itpkb	0.46	0	0.41	2.73	1.39
Itpkc	2.72	0	1.64	1.21	0
Itpr1	0.38	0	2.05	1.08	0.63
Itpr2	2.14	1.45	0.83	1.66	0.98
Itpr3	1.1	0	1.8	0	0.55
Itprip	NA	NA	1.78	NA	0
Itpripl1	NA	NA	1.74	1.09	2.79
Itpripl2	NA	NA	0.36	1.63	0
Itsn1	0.17	1.16	1.26	0	0.98
Itsn2	NA	0.93	1.05	1.14	0
Ivd	0.43	0.88	0.96	0.11	1.05
Ivl	1.95	1.29	6.51	0	0
Ivns1abp	5.35	1.12	0.89	1.13	2.21
Iws1	NA	NA	1.7	2.57	0
Ixl	0.24	NA	NA	NA	NA
Iyd	NA	NA	1.06	0.77	0.29
Izumo1	NA	NA	0	2.49	NA
Izumo2	NA	NA	5.53	0.54	NA
Izumo4	NA	1.89	2.4	3.41	NA
Jag1	0	0	1.13	2.02	0
Jag2	5.65	1.06	1.04	1.08	0
Jagn1	NA	NA	1.66	2.44	0
Jak1	0	0	1.05	0	1.92
Jak2	0.26	0.94	2.25	0.79	0.41

Jak3	0	0	1.66	1.21	0
Jakmip1	NA	NA	1.43	4.49	1.02
Jakmip2	NA	2.06	1.98	0.18	0.12
Jakmip3	NA	NA	0.5	1.22	0
Jam2	0.26	0.72	0.66	1.3	1.13
Jam3	0.69	1.38	0.51	3.29	1.27
Jarid1a	0	NA	NA	NA	NA
Jarid1b	0	NA	NA	NA	NA
Jarid1c	0.62	NA	NA	NA	NA
Jarid1d	0.63	NA	NA	NA	NA
Jarid2	0	0.6	3.15	0	1.65
Jazf1	NA	NA	4.24	1.3	3.64
Jdp2	NA	NA	0	1.17	2.91
Jhdm1d	NA	0.21	2.42	1.65	0.99
Jkamp	NA	NA	1.26	0.93	0
Jmjd1a	0	NA	NA	NA	NA
Jmjd1b	0.13	NA	NA	NA	NA
Jmjd1c	0.53	1.52	1.17	0	2.36
Jmjd2a	0.93	NA	NA	NA	NA
Jmjd2b	0.52	NA	NA	NA	NA
Jmjd2c	0	NA	NA	NA	NA
Jmjd4	NA	0	1.62	NA	0.68
Jmjd5	NA	NA	NA	NA	2.21
Jmjd6	NA	0.94	1.77	1.09	2.96
Jmjd7	NA	0.43	0.55	NA	NA
Jmjd8	NA	NA	0.28	0	0
Jmy	0.77	NA	0.8	0	0
Josd1	NA	1.81	2.31	1.59	0
Josd2	NA	NA	2.95	1.19	0.43
Jph1	1.72	NA	2.76	2.89	2.59
Jph2	0.44	2.37	0	0.75	1.53
Jph3	0.39	1.33	0.8	0.84	2.9
Jph4	0	NA	0	3.89	7.82
Jpx	NA	NA	1.91	NA	NA
Jrk	0.41	0.07	0.39	0.68	0
Jsrp1	NA	NA	2.75	1.85	7.64
Jtb	1.24	2.6	1.76	0	0
Jtv1	0	NA	NA	NA	NA
Jub	0.78	NA	NA	NA	4.91
Jun	0.07	2.19	2.63	0.27	1.91
Junb	0.81	1.09	2.18	0.15	0

Jund	NA	0	0.97	0	0
Jup	4.12	0.22	0.3	0	0.99
Kalrn	NA	0.51	0.4	2.67	0
Kank1	NA	0.65	0.34	0	0.78
Kank2	NA	1.88	1.76	0	1.75
Kank3	NA	1.53	1.3	1.42	3.31
Kank4	NA	NA	1.31	1.11	9.02
Kansl1	NA	NA	1.9	2.53	NA
Kansl1l	NA	0	1.06	0	NA
Kansl2	NA	0.05	1.78	3.91	NA
Kansl3	NA	0.94	1.25	3.19	NA
Kars	0.5	1.74	1.39	0.93	NA
Kat2a	NA	0.24	0	0.3	0
Kat2b	NA	0.74	0.13	1.65	0.76
Kat5	NA	1.86	1.28	0.32	0
Kat6a	NA	0.91	0.68	1.66	NA
Kat6b	NA	0	0.88	0	NA
Kat7	NA	0	0.87	0	NA
Kat8	NA	1.19	1.9	NA	NA
Katna1	0	1.61	8.2	0	5
Katnal1	0.44	NA	0.76	1.14	0
Katnal2	NA	NA	0.18	1.25	1.48
Katnb1	0	0.09	1.02	0.25	0
Katnbl1	NA	1.73	1.14	NA	NA
Kazald1	3.22	1.69	1.8	1.47	0.88
Kazn	NA	0.67	0.59	1.84	NA
Kbtbd10	NA	NA	NA	NA	4.69
Kbtbd11	NA	0.46	0	1.79	2.16
Kbtbd12	NA	NA	0.97	0.77	NA
Kbtbd13	NA	NA	NA	1.12	NA
Kbtbd2	0.84	1.87	2.06	0.69	0
Kbtbd3	0	NA	2.47	0.7	0
Kbtbd4	1.5	1.16	1.29	NA	1.2
Kbtbd5	0.63	NA	NA	NA	NA
Kbtbd7	NA	NA	1.72	0	0
Kbtbd8	NA	NA	0.49	1.3	1.17
Kbtbd9	0.98	NA	NA	NA	NA
Kcmf1	0.71	1.97	1.62	3.66	0.89
Kcna1	0.33	0.86	0.7	1.47	0.9
Kcna10	2.23	NA	NA	3.66	NA
Kcna2	1.63	2.13	0.8	1.13	2.99

Kcna3	0.05	0	1.13	0.9	1.85
Kcna4	1.75	1.33	0.86	2.23	1.08
Kcna5	1.27	1.61	0.87	1.97	1.39
Kcna6	0	1.72	1.76	2.96	1.68
Kcna7	0.61	NA	3.56	1.94	2.25
Kcnab1	0	0.8	0.77	0.06	1.06
Kcnab2	1.07	0.22	1.07	0	1.2
Kcnab3	0	2.39	1.31	0.28	0
Kcnb1	0.05	1.49	0	0.35	0.39
Kcnb2	NA	NA	NA	0.65	NA
Kcnc1	0.9	0.17	0.01	2.88	0.82
Kcnc2	NA	1.42	1.4	0.18	2.74
Kcnc3	1.47	0.96	0	1.93	0
Kcnc4	0	1.32	0.74	2.11	1.95
Kcnd1	1.24	2.66	0.32	0.62	4.22
Kcnd2	0.91	0	0.47	0.82	1.57
Kcnd3	0.19	0.3	1.66	0.82	1.19
Kcne1	NA	2.07	0	1.9	5.39
Kcne1l	0.67	1.41	0	2.07	0
Kcne2	0.65	1.08	0.67	1.2	1.62
Kcne3	0.76	NA	1.43	3.05	0
Kcne4	0.59	0	1.67	1.95	0
Kcnf1	0	1.01	1.7	2.31	8.21
Kcng1	NA	0	1.65	2.04	1.93
Kcng2	NA	1.92	1	2	0
Kcng3	0.36	NA	NA	1.97	NA
Kcng4	0.26	NA	2.14	1.09	NA
Kcnh1	0	1.82	1.26	2.09	2.54
Kcnh2	1.64	1.16	1.18	1.98	NA
Kcnh3	1.5	NA	2.03	0.93	0.77
Kcnh4	NA	NA	NA	0.07	NA
Kcnh5	NA	NA	0.68	1.43	0
Kcnh6	NA	NA	NA	7.39	NA
Kcnh7	0	NA	0.7	5.94	0
Kcnh8	1.26	NA	1.16	5.58	NA
Kcnip1	3.22	0.91	0.02	0.89	2.74
Kcnip2	0	1.53	0.11	0.31	1.1
Kcnip3	NA	NA	0.26	0.3	0
Kcnip4	0	NA	0.8	4.34	1.26
Kcnj1	1.13	0.8	0	0	2.07
Kcnj10	1.05	1.57	2.13	3.46	0

Kcnj11	0.95	NA	0	0	1.45
Kcnj12	0	2.81	1.86	NA	1.4
Kcnj13	NA	1.12	0.24	4.74	0
Kcnj14	0.46	2.5	2.97	1.96	4.58
Kcnj15	1.14	0	0.95	1.17	0
Kcnj16	0.11	0	1.08	2.68	2.73
Kcnj2	0	0	3.47	0	0
Kcnj3	0.65	0.85	0.37	1.49	3.23
Kcnj4	2.33	0.93	0.21	0.68	6.42
Kcnj5	3.48	1.02	0.39	0.6	2.08
Kcnj6	0.58	0.98	1.17	0.74	1.68
Kcnj8	0	1.04	0.79	1.91	0
Kcnj9	0.71	0.29	0	0.53	2.73
Kcnk1	0	0.38	1.45	1.73	1.13
Kcnk10	NA	3.02	2.23	1.4	0
Kcnk12	1.04	0	1.75	1.83	2.32
Kcnk13	1.27	0.88	1.42	0.1	3.4
Kcnk15	NA	1.3	0.56	1.26	12.6
Kcnk16	1.13	NA	2.88	0.05	1.72
Kcnk18	NA	NA	NA	0.94	NA
Kcnk2	0.34	1.67	5.82	0.54	4.93
Kcnk3	0.63	1.16	0.01	1.23	0
Kcnk4	0.47	NA	0.04	0.98	3.18
Kcnk5	0.44	2.74	0.36	1.59	3.54
Kcnk6	0	NA	0	0.39	0.47
Kcnk7	7.99	2.01	0.92	0.84	0
Kcnk9	NA	NA	NA	1.45	NA
Kcnma1	0.97	0	0.9	0	0
Kcnmb1	1.53	1.11	0	0.02	1.39
Kcnmb2	0.21	0.35	0.2	1.38	0
Kcnmb3	NA	NA	NA	3.94	NA
Kcnmb4	0.86	0.09	1.1	2.03	NA
Kcnn1	0.9	2.64	1.47	1.44	0
Kcnn2	0.39	1.82	1.75	1.7	0
Kcnn3	1.72	1.1	0.39	0.15	0
Kcnn4	0.58	0.14	1.6	4.16	0.1
Kcnq1	0.43	1.01	4.46	1.38	0.03
Kcnq1ot1	NA	NA	0.58	NA	0.95
Kcnq2	0.4	0.15	0.53	0.54	1.42
Kcnq3	1.83	1.36	2.53	1.46	0
Kcnq4	0.61	2.71	1.58	2.78	0

Kcnq5	2.8	NA	0.65	0	0.28
Kcnrg	3.06	NA	NA	3.05	NA
Kcns1	0.6	1.1	4.25	0.54	0
Kcns2	0	NA	1.29	0.1	2.79
Kcns3	0	0.67	1.19	1.03	0
Kcnt1	NA	NA	0.97	0.66	NA
Kcnt2	NA	NA	0	1.81	2.47
Kcnu1	NA	NA	1.16	NA	4.86
Kcnv1	0.92	0.72	0.45	0.52	1.3
Kcnv2	4.38	0.25	1.54	0.92	2.59
Kcp	NA	NA	1.05	1.36	0
Kctd1	2.03	NA	0.51	0.49	0.65
Kctd10	0.38	NA	1.27	0	3.35
Kctd11	4.34	NA	1.28	NA	0
Kctd12	0.82	0.34	0.55	1.49	8.42
Kctd13	0.72	1.98	1.14	2.15	1.47
Kctd14	NA	1.23	0.8	0.12	1.73
Kctd15	0.74	0.95	0	3.08	0.7
Kctd16	3.45	NA	1.9	0.9	0
Kctd17	NA	0.64	1.71	1.75	1.1
Kctd18	NA	NA	1.75	0.55	4.03
Kctd19	NA	NA	NA	0.79	NA
Kctd2	0.65	2.13	1.61	0.7	1.4
Kctd20	NA	1.4	0.4	0	1.2
Kctd21	NA	NA	3.26	NA	0.51
Kctd3	0.79	1.22	0	1.85	1.65
Kctd4	0	NA	1.91	2.07	0
Kctd5	0	1.29	0.84	1.11	0
Kctd6	0.33	NA	4.74	0.13	0.22
Kctd7	0	0.2	1.16	0	0
Kctd8	0.62	NA	NA	0.6	NA
Kctd9	0.5	1.88	3.47	0.59	0
Kdelc1	1.23	3.01	1.57	2.13	0.73
Kdelc2	NA	NA	1.88	0	0.02
Kdelr1	0	1.76	1.09	0.33	0
Kdelr2	0	1.09	0.68	2.88	0
Kdelr3	0.64	1.23	1.41	0.94	0.7
Kdm1a	NA	0	1.48	0.01	NA
Kdm1b	NA	NA	1.71	2.34	NA
Kdm2a	NA	0.37	1.24	0.46	0
Kdm2b	NA	NA	3.24	1.45	0

Kdm3a	NA	0.08	1.12	0.13	3.3
Kdm3b	NA	0.84	1.16	0	0
Kdm4a	NA	0	0.94	0	1.17
Kdm4b	NA	2.91	1.45	0.46	1.56
Kdm4c	NA	0	0.79	0	0
Kdm4d	NA	2.67	1.01	1.06	13.88
Kdm5a	NA	1.04	0.37	0	0.49
Kdm5b	NA	0.52	0.64	0	1.27
Kdm5c	NA	0.74	0.25	0	0.72
Kdm5d	NA	1.45	2.37	3.95	0
Kdm6a	NA	0.6	1.79	0	0
Kdm6b	NA	2.97	3.46	1.45	0
Kdm8	NA	0.04	1.43	0.77	NA
Kdr	0.51	1.54	1.34	1.23	0
Kdsr	NA	1.56	2	0.44	0
Keap1	1.01	0.8	1.87	1.46	0
Kel	1.28	1.32	0.21	1.76	1.61
Kera	4	0.08	0.69	0.73	1.55
Khdrbs1	0.25	0.94	0.72	0.96	0.1
Khdrbs2	0.14	0.79	1.3	1.96	2.62
Khdrbs3	0.51	2.41	1.68	1.09	0.07
Khk	0.14	0.7	2.07	0.7	2.05
Khnyn	NA	0.87	0.2	0	NA
Khsrp	0	0	0.11	1.28	1.15
Kidins220	NA	1.09	0.72	0	0.9
Kif11	0	0.27	1.9	0.74	1.36
Kif12	0.54	NA	0.14	0.01	0
Kif13a	0.52	0	0.89	0.22	0.74
Kif13b	0.17	0	0.44	0.01	3.25
Kif14	1.79	0	0.43	0.13	0
Kif15	NA	1.29	0.64	0.01	5.71
Kif16b	NA	1.44	0.24	0	0.35
Kif17	0.53	1.46	0	0	0
Kif18a	0.18	1.43	0.74	0.69	0
Kif18b	NA	0.05	1.96	0	3.29
Kif1a	0.64	1.24	0.15	0	0
Kif1b	0.53	0	1.18	1.79	0
Kif1c	0.71	1.25	0.58	2.07	2.17
Kif20a	2.31	0.84	3.48	NA	0
Kif20b	NA	1.25	1.87	0.91	0.39
Kif21a	0.32	NA	0.55	4.03	0

Kif21b	0.4	0	0.59	1.34	1
Kif22	0.16	1.39	4.38	0.03	2
Kif23	0.57	0.88	0.98	0	0.56
Kif24	1.49	1.97	0.6	0	0.82
Kif26a	NA	NA	1.32	0.43	2.23
Kif26b	NA	0.01	0.48	0.23	3.89
Kif27	0.27	NA	1.19	3.53	0.77
Kif2a	1.1	0.46	0.75	0.84	0
Kif2b	0	NA	0.05	3.97	5.92
Kif2c	1.96	0	2.31	0	0
Kif3a	0.26	1.02	1.58	0	0
Kif3b	0	1.19	1.01	0.63	1.82
Kif3c	1.25	2.91	2.01	NA	0
Kif5a	0.7	0.44	0.24	0	0
Kif5b	0.59	0	1.87	0	0.92
Kif5c	1.15	0	0.83	0.71	0.69
Kif6	NA	NA	0.28	1.37	5.66
Kif7	0.29	NA	2.33	0.55	1.39
Kif9	0.47	NA	2.48	1.13	NA
Kifap3	0	0	1.69	0.26	0
Kifc1	1.09	NA	NA	0	0
Kifc2	0.75	NA	0.71	1.54	0
Kifc3	1.14	3.39	1.44	2.22	12.86
Kin	0.34	2.88	1.06	0.67	1.09
Kir3dl1	1.11	NA	NA	NA	NA
Kirrel	NA	1.1	1.48	9.01	3.19
Kirrel2	0.47	NA	NA	0.29	NA
Kirrel3	3.39	NA	0.42	1.09	0
Kiss1	0.2	NA	NA	1.29	NA
Kiss1r	NA	NA	5.67	0.85	5.07
Kit	1.86	1.56	1.25	1.11	0.65
Kl	0	1.38	1.17	3.28	0
Klb	NA	NA	0.71	0.92	0.13
Klc1	NA	1.06	0.56	0.03	1.05
Klc2	0.36	0	1.61	0	0
Klc3	NA	NA	1.65	0.95	0
Klc4	NA	NA	2.74	1.15	0.79
Klf1	1.18	0.52	2.5	0.24	0
Klf10	NA	2.72	3.2	0	0
Klf11	NA	0	0.66	1.73	1.67
Klf12	0.47	0	0.74	0	1.16

Klf13	0	0.88	1.08	5.21	1.73
Klf14	NA	NA	0	2.33	NA
Klf15	3	2.66	0.91	0.8	1.21
Klf16	3.18	NA	0	2.23	0.89
Klf17	NA	NA	0.35	2.47	NA
Klf2	0.19	0	1.98	0.9	0.16
Klf3	1.19	0	1.22	NA	0
Klf4	0.75	1.72	2.99	0.73	1.97
Klf5	0.5	0.14	0.6	3.23	0
Klf6	NA	0.12	1.67	1.18	0
Klf7	1.56	0.37	0.27	0	0
Klf8	1.86	NA	NA	4.08	NA
Klf9	NA	0.63	1.19	0.39	0.57
Klhdc1	0	NA	0.76	0.75	NA
Klhdc10	NA	0	1.26	0	0
Klhdc2	0.26	1.11	1.55	0.01	5.09
Klhdc3	0.85	0.91	0.68	0.01	2.24
Klhdc4	0	1.99	0.38	2.2	0.27
Klhdc5	NA	NA	NA	NA	0
Klhdc7a	NA	NA	1.68	1.38	NA
Klhdc7b	NA	NA	NA	0.74	NA
Klhdc8a	NA	2.59	0.26	0.18	1.61
Klhdc8b	NA	NA	0.68	1.78	4.39
Klhdc9	NA	NA	1.43	0	1.22
Klhl1	2.96	0.3	0.93	0	2.93
Klhl10	2.15	NA	0.21	8.21	NA
Klhl11	0.75	2.12	0.39	2.52	0
Klhl12	0	1.2	3.59	0.66	0
Klhl13	2.83	NA	1.62	3.08	1.2
Klhl14	NA	NA	2.21	0	0
Klhl15	0.65	NA	0.52	3.07	0.95
Klhl17	0	NA	5.3	0	0
Klhl18	0	0.14	0.65	2.28	1.17
Klhl2	0.66	0.59	2.56	2.31	0
Klhl20	NA	0.46	0.4	0	0
Klhl21	NA	0.06	1.5	0.78	0
Klhl22	NA	0	0	0.02	0
Klhl23	NA	1.86	0	NA	0
Klhl24	NA	1.01	0.51	0.52	1.94
Klhl25	NA	0.22	0.82	1.89	2.01
Klhl26	NA	2.28	0.57	0.1	0.84

Klhl28	NA	0	1.39	1.15	0
Klhl29	NA	0.49	2.17	1.32	3.07
Klhl3	NA	NA	NA	0	NA
Klhl30	NA	NA	NA	1.63	NA
Klhl31	NA	NA	NA	0.57	NA
Klhl32	NA	NA	0	0.43	NA
Klhl33	NA	NA	NA	1.53	NA
Klhl34	NA	NA	NA	1.08	NA
Klhl35	NA	0.24	1.68	1.39	0
Klhl36	NA	1.62	1.24	0.33	0.07
Klhl38	NA	NA	NA	3.56	NA
Klhl4	1.55	1.21	0.65	1.72	0
Klhl40	NA	NA	0.37	1.96	NA
Klhl41	NA	1.2	1.76	0	NA
Klhl42	NA	NA	1.17	2.81	NA
Klhl5	0.66	0	0.77	0.31	2.44
Klhl6	0.89	NA	2.6	3.63	4.41
Klhl7	0.29	0.41	1.52	0.12	0
Klhl8	2.5	NA	0.4	0.57	1.62
Klhl9	NA	0	0.38	0	0
Klk1	0.38	1.14	1.06	NA	1.18
Klk10	0.7	0.63	0.94	1.36	5.25
Klk11	0.73	0	1.6	2.14	0
Klk12	4.07	0.53	0.95	2.01	3.58
Klk13	8.72	NA	NA	1.25	NA
Klk14	1.19	NA	NA	1.83	NA
Klk15	1.26	NA	NA	2.36	NA
Klk4	0.4	NA	3.16	0.67	4.85
Klk5	0.92	1.93	2.68	0.78	0
Klk6	1.11	1.05	0.03	2.12	0
Klk7	2.21	0.88	1.66	0.83	0
Klk8	0.69	2.21	1.65	NA	0
Klk9	1.86	NA	1.98	1.08	NA
Klkb1	2.79	2.26	3.66	NA	0.62
Klra1	0.4	NA	NA	NA	0.42
Klrb1	NA	NA	NA	0.83	NA
Klrc1	3.31	NA	NA	0	NA
Klrc2	0	1.96	1.32	NA	NA
Klrc3	0.34	0.94	1.86	3.17	2.02
Klrd1	0.82	0	1.05	0.61	1.46
Klrg1	0	0	2.52	1.59	4.41

Klrg2	NA	NA	0.21	4.92	0
Klrk1	0.62	0.77	3.03	0.55	7.95
Kmo	1.57	2.71	1.01	NA	1.68
Kmt2a	NA	0	0.46	0.68	NA
Kmt2b	NA	1.85	1.31	1.34	NA
Kmt2c	NA	NA	1.91	1.29	NA
Kmt2d	NA	0.19	1.72	1.39	NA
Kmt2e	NA	NA	1.85	1.3	NA
Kncn	NA	NA	NA	4.12	NA
Kndc1	NA	NA	0.89	2.24	1.9
Kng1	0.81	1.84	0	2.86	3.41
Knop1	NA	1.44	1.41	0.95	NA
Kns2	0.38	NA	NA	NA	NA
Knstrn	NA	NA	1.59	0.09	NA
Kntc1	0.07	0.38	3.06	0.11	1.33
Kpna1	1.16	1.5	1.31	0.5	1.83
Kpna2	0.29	1.12	1.55	0	0.88
Kpna3	0.67	1.64	0.38	0.21	0
Kpna4	4.02	0	0.36	0.21	0
Kpna6	0.76	1.02	0.71	0	0
Kpna7	NA	NA	NA	2.4	NA
Kpnb1	0.52	1.32	0.43	1.8	0
Kprp	NA	NA	NA	1.61	NA
Kptn	0.56	2.99	0.61	1.37	1.99
Kras	NA	0	0.14	NA	0
Krba1	NA	NA	2.68	1.36	0
Krcc1	NA	0.64	1.44	0.92	1.63
Kremen1	NA	NA	0.06	0.83	0
Kremen2	1.6	NA	NA	1.69	NA
Kri1	NA	2.14	0.71	NA	5.35
Krit1	NA	0.05	0.9	1.13	0
Krr1	NA	0.89	1.6	NA	0
Krt1	NA	2.06	0.12	2.09	0
Krt10	NA	0.22	1.09	0	6.88
Krt12	NA	2.37	0.86	1.11	2.44
Krt13	NA	1.12	0	1.29	0
Krt14	NA	0.15	0.32	3.93	2.4
Krt15	NA	1.44	1.07	1.3	0
Krt16	NA	1.07	1.63	2.18	2.02
Krt17	NA	0.72	1.71	3.64	1.51
Krt18	NA	2	0.74	1.11	0.36

Krt19	NA	1.22	1.61	1.51	0
Krt2	NA	0.84	0.78	0.06	0
Krt20	0.35	1.51	2.26	0.99	9.17
Krt222	NA	NA	0.91	0.62	1.27
Krt23	NA	0.7	2.8	1.92	0
Krt24	NA	1.95	1.29	2.67	4.95
Krt25	NA	NA	0.81	0.01	0
Krt26	NA	NA	NA	1.48	NA
Krt27	NA	NA	0	2.8	0
Krt28	NA	NA	NA	0	NA
Krt31	NA	0.96	0	0.5	1.77
Krt32	NA	0.48	3.75	2.24	0
Krt33a	NA	1.1	2.71	0.92	0
Krt33b	NA	1.55	0.29	0	2.63
Krt34	NA	0.48	2.99	2.36	0
Krt35	NA	0.43	1.92	0.46	0
Krt36	NA	0.39	1.4	0.57	4.8
Krt39	NA	NA	NA	1.52	NA
Krt4	NA	0.66	0.42	3.32	2.59
Krt40	NA	NA	NA	0.69	NA
Krt5	NA	1.02	0.42	0.82	1.72
Krt6a	NA	1.02	0	0.54	1.68
Krt6b	NA	0.51	4.48	1.85	0
Krt7	NA	1.1	0.14	3.7	10.19
Krt71	NA	NA	0	1.88	0
Krt72	NA	NA	1.54	0.36	NA
Krt73	NA	NA	0.98	4.05	1.96
Krt74	NA	NA	NA	0	NA
Krt75	NA	0.55	0.1	4.06	0
Krt76	NA	0.3	0.53	3.38	0.28
Krt77	NA	NA	0	2.07	0.77
Krt78	NA	NA	0	1.35	NA
Krt79	NA	NA	0.02	0.87	NA
Krt8	NA	0.6	0.92	0.22	0
Krt80	NA	NA	2.16	0.87	4.32
Krt81	NA	0.21	1.6	NA	4.16
Krt82	NA	NA	1.79	1.6	NA
Krt84	NA	0.87	4.01	1.36	3.4
Krt85	NA	1.2	0	1.4	NA
Krt86	NA	NA	NA	1.14	0
Krt9	NA	NA	NA	0.68	NA

Krtap1-5	NA	NA	4.31	0	4.74
Krtap10-10	NA	NA	NA	2.1	NA
Krtap10-4	NA	NA	NA	0.84	NA
Krtap11-1	NA	NA	2.34	0.51	NA
Krtap12-1	2.43	NA	NA	1.36	NA
Krtap13-1	2.32	NA	1.35	0.63	NA
Krtap17-1	NA	NA	0.03	1.29	0
Krtap19-1	NA	NA	1.19	19.39	NA
Krtap19-3	NA	NA	0.26	2.07	NA
Krtap19-4	NA	NA	NA	0	NA
Krtap19-5	NA	NA	NA	0	NA
Krtap2-4	NA	0.65	0	NA	4.27
Krtap20-2	NA	NA	NA	3.65	NA
Krtap21-1	0	NA	NA	2.24	NA
Krtap24-1	NA	NA	NA	2.13	NA
Krtap26-1	NA	NA	NA	0.81	NA
Krtap3-1	0	NA	0.92	0.86	0
Krtap3-2	1.45	NA	3.67	NA	0.04
Krtap3-3	0.5	NA	1.26	0	0
Krtap4-1	NA	NA	1.77	1.62	3.99
Krtap4-2	NA	NA	0.35	0.76	NA
Krtap4-6	NA	NA	1.66	NA	0
Krtap4-7	2.41	0.93	0	1.81	9.18
Krtap5-1	2.45	NA	NA	0.32	NA
Krtap5-2	NA	NA	2.76	0.74	NA
Krtap5-3	NA	NA	NA	0.76	NA
Krtap5-4	0.71	NA	NA	0.77	NA
Krtap5-5	NA	NA	NA	1.49	NA
Krtap6-1	3.08	NA	NA	0.03	NA
Krtap6-2	0	NA	NA	2.01	NA
Krtap6-3	0.21	NA	NA	0.48	NA
Krtap7-1	NA	NA	2.81	1.67	NA
Krtap8-1	0	NA	0.22	1.96	NA
Krtap9-3	NA	NA	0	0.53	0.21
Krtcap2	1.1	NA	3.58	0	0
Krtcap3	1.66	NA	1.34	0.73	0.78
Krtdap	NA	NA	4.17	0.65	1.13
Ksr1	NA	0.11	0.79	0	0
Ksr2	NA	NA	NA	0.71	NA
Ktelc1	NA	NA	NA	NA	0
Kti12	NA	NA	0.56	0.95	0

Ktn1	0.7	0.31	1.05	0.4	0
Kxd1	NA	0.77	1.14	0	NA
Ky	0.21	NA	0.85	1.06	0.21
Kynu	0	2.21	0.86	0	1.99
L1cam	0.92	0.73	0.71	1.11	0
L1td1	NA	0	2.03	3.98	1.7
L2hgdh	NA	1.71	1.37	0.8	0
L3hypdh	NA	NA	0	0.94	NA
L3mbtl	0.27	NA	NA	NA	NA
L3mbtl1	NA	NA	NA	0.17	NA
L3mbtl2	1.09	NA	0	0.98	3.04
L3mbtl3	1.31	NA	0.3	1.33	0
L3mbtl4	NA	NA	NA	0.9	NA
Lacc1	NA	NA	1.51	0.93	NA
Lace1	0.72	NA	1.5	0.71	NA
Lactb	0.42	NA	1.64	1.05	3.31
Lactb2	1.96	1.24	2.86	3.25	0
Lad1	0	0.96	2.05	2.48	0
Lag3	0	3.47	0	1.15	0
Lage3	NA	0.08	3.22	0.02	0.11
Lair1	0.85	0.68	3.31	0.4	1.02
Lalba	1.44	0.69	0	3.92	0
Lama1	2.63	2.91	0.29	1.02	0
Lama2	0.56	1.23	2.34	0.66	0
Lama3	0.71	1.17	1.22	0	0
Lama4	0.24	0.71	0.58	0	0
Lama5	0	0.52	0.51	0.57	2.23
Lamb1	NA	1.08	0.07	5.44	NA
Lamb2	0.86	2.32	10.89	0	0
Lamb3	0.07	2.91	0	1.12	0
Lamc1	0	0.69	1.72	0	1.19
Lamc2	0.41	0	0.75	0	2
Lamc3	0.44	0.55	0.9	1.82	0
Lamp1	1.75	0.92	1.18	2.26	1.91
Lamp2	0.47	0.04	1.78	0	0
Lamp3	7.79	1.55	0.81	0	NA
Lamp5	NA	1.33	6.63	2.62	NA
Lamtor1	NA	NA	0.41	0.6	NA
Lamtor2	NA	1.57	0.6	1	NA
Lamtor3	NA	0	2.06	0.98	NA
Lamtor4	NA	NA	2.2	0.83	NA

Lamtor5	NA	0	1.6	0.49	NA
Lancl1	0	0	1.26	1	2.41
Lancl2	0.59	0.98	2.12	0	0.57
Lancl3	NA	NA	5.42	2.6	NA
Lap3	7	0.61	2.65	0.7	1.09
Laptm4a	0.6	4.15	2.76	0	2.98
Laptm4b	0.97	0.53	0	1.32	1.51
Laptm5	0.15	1.56	0.72	6.5	0.1
Large	1.09	1.22	2.4	1.87	1.35
Larp1	NA	2.41	0.67	1.36	1.74
Larp1b	NA	NA	1.29	0.91	0
Larp4	NA	0.82	0.19	0.82	0
Larp4b	NA	0.84	1.08	0	2.48
Larp6	NA	0.66	0.81	0.46	0
Larp7	NA	0.02	0.85	0.82	1.06
Lars	0	2.33	0.66	0.32	2.27
Lars2	0	0.5	0.5	0.8	0.8
Las1l	NA	1.17	1.64	1.45	0
Lasp1	0	2.03	0.47	1.37	0
Lass1	0.12	NA	NA	NA	NA
Lass2	0	NA	NA	NA	0
Lass4	0.37	NA	NA	NA	0
Lass5	0.57	NA	NA	NA	0.78
Lass6	0.61	NA	NA	NA	1.98
Lat	0.95	NA	NA	0.99	NA
Lat2	NA	1.83	1.42	1.5	1.27
Lats1	0.78	1.56	0.47	0.96	0
Lats2	0	NA	0.36	2.47	1.77
Lax1	NA	0	1.89	1.33	2.4
Layn	NA	NA	0.51	1.36	1.06
Lba1	0	NA	NA	NA	NA
Lbh	0.71	0	1.15	2.88	0
Lbp	0.23	0.64	0.87	0.98	0
Lbr	0.08	1.43	3.69	0.06	1.94
Lbx1	NA	2.01	1.25	0.06	0
Lbx2	NA	NA	NA	3.36	NA
Lca5	NA	NA	0.14	0.94	1.64
Lca5l	NA	NA	1.59	0	3.08
Lcat	0.41	0.76	0.35	NA	1.42
Lce1b	NA	NA	0.52	1.98	NA
Lce1c	NA	NA	NA	0	NA

Lce1d	NA	NA	NA	1.29	NA
Lce1e	NA	NA	NA	1.63	NA
Lce1f	NA	NA	NA	0	NA
Lce3a	NA	NA	NA	1.35	NA
Lce3b	NA	NA	NA	0.96	NA
Lce3c	NA	NA	NA	1.11	NA
Lck	4.58	0	0.1	4.84	2.38
Lclat1	NA	NA	1.95	1.23	0
Lcmt1	0.6	0	2.17	2.29	0
Lcmt2	0	1.28	0.9	1.08	0
Lcn10	NA	NA	NA	2.36	NA
Lcn12	NA	NA	0	0.33	5.8
Lcn2	0.92	1.66	2.09	1.45	0
Lcn6	NA	NA	NA	3.16	NA
Lcn8	NA	NA	0.81	1.25	3.16
Lcn9	NA	NA	NA	3.61	NA
Lcor	NA	NA	0.78	0	0.7
Lcorl	NA	NA	0.63	0	0
Lcp1	0.6	2.79	1.3	1.95	0
Lcp2	0	1.49	0.84	1.58	0
Lct	NA	0.15	0.91	0.38	7.09
Lctl	0.55	NA	NA	0.68	NA
Ldb1	0.85	2.72	1.31	6.17	1.3
Ldb2	0.91	1.22	0.85	1.57	1.5
Ldb3	0.29	0.45	0.83	0.89	1.83
Ldha	NA	0	1.66	2.37	0
Ldhal6b	0.98	1.89	1.78	0	8.71
Ldhb	NA	0.59	0.31	0	3.66
Ldhc	NA	0.19	0.21	0	0
Ldhd	0.9	NA	3.93	0.77	1.3
Ldlr	0.46	0.02	1.75	0	4.18
Ldlrad1	NA	NA	NA	0.32	NA
Ldlrad2	NA	NA	NA	1.1	NA
Ldlrad3	NA	NA	3.15	2.2	5.07
Ldlrad4	NA	0.57	0.29	0.37	NA
Ldlrap1	NA	0	2.05	3.19	0
Ldoc1	NA	3.64	2.14	4.42	1.25
Ldoc1l	NA	NA	0.55	0	4.44
Leap2	NA	NA	0	0.07	NA
Lect1	0.22	3.66	0.25	0.43	2.7
Lect2	1.73	1.09	0	1.36	1.59

Lef1	0	0	0.16	0	0
Lefty1	NA	1.12	0	0.82	1.33
Lefty2	NA	2.58	1.43	0.64	0
Lekr1	NA	NA	0.98	1.4	NA
Lelp1	NA	NA	0.79	1.69	0
Lemd1	1.59	NA	0	1.98	4.72
Lemd2	0.12	NA	2.71	0	0
Lemd3	NA	1.2	0	0	0.25
Lenep	1.25	NA	NA	NA	3.79
Leng1	0.99	NA	0	0.78	1.8
Leng4	0	NA	NA	NA	NA
Leng8	0.17	NA	0.06	0.03	2.04
Leng9	1.92	NA	3.85	NA	NA
Leo1	NA	NA	0.69	0.09	3.5
Lep	1.87	2.14	1.18	1.43	0
Lepr	1.45	0	0.29	NA	0
Lepre1	0	1.22	2.79	0.51	0
Leprel1	0.82	1.48	0.52	1.3	0
Leprel2	0.21	1.86	1.45	NA	NA
Leprel4	NA	0.27	1.01	0.23	NA
Leprot	NA	0.83	1.66	1.09	0
Leprotl1	0	0.03	1.28	0	2.63
Letm1	0.15	0.08	1.01	0.37	0.52
Letm2	NA	NA	0.32	0.25	NA
Letmd1	0	0	0.82	1.4	0
Lfng	0	0.23	1.23	1.05	0
Lgals1	0.83	0	2.6	1.91	1
Lgals12	1.19	NA	1.83	1.56	0.1
Lgals2	0.86	0.45	0.82	1.83	2.55
Lgals3	0.1	3.41	0.29	1.13	0
Lgals3bp	0	1.7	1.78	0.16	0
Lgals4	1.07	0.95	3.15	NA	1.83
Lgals7	0	NA	NA	NA	NA
Lgals8	0.73	0.16	0.45	NA	0
Lgals9	0.59	2.03	3.49	1.84	0.81
Lgalsl	NA	2.05	4.06	0.33	NA
Lgi1	2.74	1.22	2.49	2.05	3.04
Lgi2	1.07	0.3	0.76	0.6	0
Lgi3	3.27	NA	0.66	4.74	0.81
Lgi4	1.26	NA	4.95	1.09	0
Lgmn	0.53	2.66	1.38	2.44	1

Lgr4	NA	1.4	1.16	0.06	0
Lgr5	NA	1.16	0.09	0.52	3.17
Lgr6	0	NA	0.08	0.4	2.31
Lgsn	NA	NA	NA	1.46	NA
Lgtn	0.06	NA	NA	NA	1.8
Lhb	1.09	3.04	0.24	1.04	9.16
Lhcgr	0.57	1.62	0	4.08	0
Lhfp	0	3.03	2.93	0.87	0
Lhfpl1	NA	NA	NA	1.88	NA
Lhfpl2	0.65	0	2.78	2.27	0
Lhfpl3	NA	NA	1.33	2.19	1.27
Lhfpl4	NA	NA	0	0.68	2.33
Lhfpl5	NA	NA	1.43	0	NA
Lhpp	NA	0	0.81	0.48	0.88
Lhx1	2.46	0.14	1.59	0	5.06
Lhx2	0	0	2.47	2.1	3.32
Lhx3	0.18	1.28	1.53	0.73	2.18
Lhx4	0.43	NA	1.41	1.07	NA
Lhx5	4.24	1.85	0.2	0	4.16
Lhx6	2.62	1.19	0.02	0.82	0
Lhx8	0.82	NA	0	0.87	NA
Lhx9	0.22	NA	0.54	1.25	NA
Lias	0.15	0.32	0	0.87	1.71
Lif	0.09	0.74	0.1	0.87	2.45
Lifr	0.39	1.89	0.94	2.97	1.1
Lig1	1.16	0	4.17	0.48	6.6
Lig3	0	1.24	0.47	0	0.81
Lig4	1.13	1.05	1.55	1.14	2.75
Lilra5	NA	NA	NA	0	NA
Lilrb3	NA	0.21	4.21	NA	0
Lilrb4	0.96	0.51	0.81	NA	NA
Lim2	0.34	1.05	1.22	0.54	5.34
Lima1	NA	2.43	0.63	3.78	1.98
Limch1	NA	0.96	0.18	0	2.19
Limd1	0.62	0.92	1.1	NA	1.62
Limd2	NA	1.52	2.43	NA	0.7
Lime1	NA	0	1.04	0.89	5.6
Limk1	1.28	0.98	0.54	1.79	0
Limk2	0.57	0	0.28	3.8	0
Lims1	0.31	0.82	2.19	0.25	0
Lims2	1.34	1.43	1.62	NA	2.54

Lin28	0.63	NA	NA	NA	0.21
Lin28a	NA	0.27	0	0.81	NA
Lin28b	NA	NA	2.27	1.83	0
Lin37	NA	3.38	0.63	2.42	0
Lin52	NA	NA	1.38	0.95	0
Lin54	NA	NA	1.3	0.4	2.48
Lin7a	0.43	2.64	0.3	1.18	0
Lin7b	1.86	1.02	1.06	NA	0.11
Lin7c	1.22	0.94	1.11	0.18	2.29
Lin9	NA	NA	1.1	0.39	0.86
Lingo1	NA	NA	0.46	1.61	0
Lingo2	NA	NA	0	1.5	1.53
Lingo3	NA	NA	2.89	0.82	NA
Lingo4	NA	NA	NA	1.16	NA
Lins	NA	1.23	0.6	1.02	NA
Lipa	NA	0	0.35	1.28	1.76
Lipc	0.9	0.08	0	2.66	0.55
Lipe	0.39	0.24	1.68	0	0
Lipf	0.41	1.56	1.36	0.61	2.06
Lipg	0.82	0.99	0.75	1.57	0
Liph	0.42	NA	0.46	3.24	0.12
Lipi	NA	NA	0	0.7	3.89
Lipk	NA	NA	NA	0.83	NA
Lipl2	0	NA	NA	NA	NA
Lipl3	3.98	NA	NA	NA	NA
Lipm	NA	NA	NA	0.24	NA
Lipn	NA	NA	NA	1.59	NA
Lipt1	NA	0.07	2.23	0	8.51
Lipt2	NA	NA	NA	1.8	NA
Litaf	0.09	1.96	2.04	0.46	0
Lix1	0.71	NA	0.78	1.01	0
Lix1l	NA	NA	1.39	0.35	1.85
Llgl1	NA	1.54	1.33	1.26	2.89
Llgl2	NA	0	2.5	0.05	NA
Llph	NA	0	2.96	0.23	0.36
Lman1	0.83	1.08	0.16	0.99	0
Lman1l	1.22	2.09	1.67	2.95	1.78
Lman2	0.42	2.4	1.02	0.63	2.38
Lman2l	0	1.15	1.3	0.84	1.37
Lmbr1	0.66	NA	0.93	0	0
Lmbr1l	NA	0	0.45	0	4.22

Lmbrd1	NA	2.32	2.88	1.15	0
Lmbrd2	NA	NA	NA	1.06	NA
Lmcd1	0	1.27	0.6	0	0
Lmf1	NA	0	0.07	0	0.7
Lmf2	NA	0.01	1.18	2.73	1.02
Lmln	0.5	NA	0.68	0	1.71
Lmna	0.1	0.97	0.15	1.22	0
Lmnb1	0.53	0.03	5.96	0	0
Lmnb2	0.89	0.26	0.77	0	1.78
Lmo1	1.38	0.59	2.4	2.76	3.99
Lmo2	0.22	2.45	5.68	1.47	2.21
Lmo3	1.21	2.05	1.32	0.96	0
Lmo4	0.91	0.17	2.66	2.81	1.55
Lmo6	0.71	NA	NA	NA	NA
Lmo7	1.55	1.46	0.31	0.69	1.36
Lmod1	0.47	1.27	0.24	0.77	0
Lmod2	NA	NA	0	1.45	0
Lmod3	4.61	NA	5.17	2.64	1.99
Lmtk2	2.39	1.51	4.5	0.38	0
Lmtk3	NA	NA	0.88	1.19	NA
Lmx1a	0.28	NA	0	0.84	NA
Lmx1b	4.52	1.42	1.14	0.75	3.83
Lnpep	0	1.1	0	2.41	1.1
Lnx1	2.33	NA	0	1.84	0.65
Lnx2	0	NA	1.48	2.71	1.93
Loh11cr2a	0.63	NA	NA	NA	NA
Loh12cr1	2.25	NA	0	1.08	1.01
Lonp1	NA	0.07	3.48	0	0
Lonp2	NA	0	0.22	0.22	1.05
Lonrf1	NA	NA	1.93	0.83	NA
Lonrf2	NA	NA	1.12	1.54	0.89
Lonrf3	NA	0.02	2.92	1.38	0.74
Lor	0.75	0.99	0.06	3.18	2.27
Lox	1.03	0.54	1.2	0.78	0
Loxhd1	0	NA	NA	2.16	NA
Loxl1	0.23	2.11	1.48	0	0
Loxl2	0.73	0.95	1.54	0	1.32
Loxl3	0.81	1.81	2.69	NA	0
Loxl4	0.84	NA	1.54	1.35	0
Lpar1	NA	0.93	0.76	2.06	0.67
Lpar2	NA	1.75	0.88	1.72	0

Lpar3	NA	0.93	1.77	0.81	0
Lpar4	NA	0.49	1.84	0.99	0
Lpar5	NA	NA	NA	1.39	NA
Lpar6	NA	2.44	0.77	0.57	0
Lpcat1	NA	0.39	0.77	4.38	2.46
Lpcat2	NA	NA	0.46	1	0
Lpcat3	NA	1.05	0.18	0.73	1.49
Lpcat4	NA	0	1.17	2.24	0
Lpgat1	NA	0.04	1.23	2.01	0.51
Lphn1	0	0	0.54	1.61	0
Lphn2	0	0.72	1.07	1.72	NA
Lphn3	0.29	0	0	0.53	0
Lpin1	0	0	2.44	0.08	0
Lpin2	0.29	0	1.66	0	0.61
Lpin3	0	NA	1.85	NA	2.56
Lpl	0.31	1.41	5.87	5.58	3.12
Lpo	0.07	1.9	1.15	1.5	3.45
Lpp	0	0.93	1.8	0.76	1
Lpxn	0.3	0	1.91	0	0
Lrat	1.3	1.1	1.21	1.27	1.3
Lrba	0.37	0	0.6	0	0
Lrch1	NA	1.25	0.98	0	0.86
Lrch2	3.94	NA	0.88	3.44	0.53
Lrch3	0	1.2	1.08	0	0.24
Lrch4	0.6	1.17	0	NA	0.98
Lrdd	0.96	NA	NA	NA	3.77
Lrfn1	NA	NA	0.47	0.85	0.66
Lrfn2	0.06	NA	3.74	1.08	0
Lrfn3	0.23	0	0.9	4.64	1.01
Lrfn4	0	1.06	1.25	2.88	0
Lrfn5	0.13	NA	1.53	1.77	2.51
Lrg1	0	NA	3.04	1.73	0
Lrguk	NA	NA	0	0.83	0
Lrif1	NA	1.37	2.74	2.39	NA
Lrig1	2.41	0	1.21	1.1	0.52
Lrig2	1.15	0	1.31	0	0
Lrig3	1.03	NA	0	16.61	0
Lrit1	NA	2.11	0.38	0.68	1.59
Lrit2	NA	NA	NA	2.02	NA
Lrit3	NA	NA	NA	0.55	NA
Lrmp	0.02	1.29	1.63	1.72	1.86

Lrp1	0.11	0.63	1.54	0	0.29
Lrp10	0.31	2.87	1.19	1.13	0
Lrp11	0.15	NA	0	1.24	0
Lrp12	1.15	0.48	0.78	1.11	1.81
Lrp1b	0.82	1.72	0.89	1.05	0
Lrp2	0.96	0.89	0.26	0.93	1.69
Lrp2bp	NA	0.6	0.06	2.54	2.79
Lrp3	NA	5.65	0.88	1.17	0
Lrp4	1.86	1.4	2.32	2.62	2.98
Lrp5	3.56	0.27	2.41	0.78	2.69
Lrp6	1.83	1.42	0.4	5.93	0
Lrp8	0.02	0.72	1.72	2.32	0.72
Lrpap1	0.11	0	6.71	0	0.28
Lrpprc	0.44	0	0.1	0	3.79
Lrr1	NA	NA	3.43	2.29	NA
Lrrc1	0.41	0.31	0	0.72	2.03
Lrrc10	0	NA	NA	2.38	NA
Lrrc14	NA	2.2	2.36	NA	0
Lrrc14b	NA	NA	NA	1.12	NA
Lrrc15	0	1.6	0.24	3.24	1.81
Lrrc16	0.4	NA	NA	NA	NA
Lrrc16a	NA	0	2.09	5.29	0
Lrrc16b	NA	NA	2.34	0.3	0
Lrrc17	0.75	0.28	0.78	1.92	0
Lrrc18	NA	NA	0.81	1.89	NA
Lrrc19	0	2.07	0.62	0.76	0.05
Lrrc2	0.55	1.71	0.89	5.85	0.77
Lrrc20	0.45	0.9	0.7	0.58	1
Lrrc21	0.31	NA	NA	NA	NA
Lrrc22	0	NA	NA	NA	NA
Lrrc23	NA	0.97	1.94	2.71	2.01
Lrrc24	NA	NA	NA	0.59	NA
Lrrc25	0.8	NA	NA	1.49	NA
Lrrc26	NA	NA	NA	1.86	NA
Lrrc27	NA	NA	2.57	1.02	0
Lrrc28	0.04	NA	0.59	0.9	0.58
Lrrc29	1.79	NA	NA	0.01	NA
Lrrc3	0	0.42	0.9	1.23	1.75
Lrrc31	NA	0.53	1.59	NA	0
Lrrc32	NA	NA	NA	1	NA
Lrrc33	NA	NA	0.75	0.65	0

Lrrc34	NA	NA	2.58	1.6	2.07
Lrrc36	NA	1.09	2.13	0.9	0
Lrrc38	NA	NA	0.35	0.86	NA
Lrrc39	NA	NA	0.23	5.11	0.65
Lrrc3b	10.86	NA	1.4	2.3	0.59
Lrrc4	0.54	NA	0.21	0.12	0.97
Lrrc40	NA	0.8	1.78	0.92	0
Lrrc41	NA	0.63	2.26	2.53	0.33
Lrrc42	NA	2.02	2.08	0.45	0.79
Lrrc43	NA	NA	NA	0.28	NA
Lrrc45	NA	NA	2.61	0.77	0.14
Lrrc46	NA	NA	1.15	1.14	0
Lrrc47	NA	0	0.57	2.07	0
Lrrc48	NA	1.1	0.76	0.38	0
Lrrc49	NA	1.2	0.76	0.64	1.58
Lrrc4b	2.13	NA	0.59	0.65	NA
Lrrc4c	NA	NA	3.61	1.55	0
Lrrc50	NA	NA	NA	NA	0
Lrrc52	NA	NA	0.86	1.39	NA
Lrrc55	NA	NA	NA	0.73	NA
Lrrc56	NA	NA	0.28	2.97	1.73
Lrrc57	NA	NA	0.08	0.05	2.12
Lrrc58	NA	NA	1.81	0.96	0
Lrrc59	NA	2	1.13	0.93	0.9
Lrrc6	0.25	0	0.72	0	0
Lrrc61	NA	2.79	3.24	2.88	2.91
Lrrc63	NA	NA	1.56	2.59	NA
Lrrc66	NA	NA	1.89	1.47	NA
Lrrc68	NA	NA	NA	NA	0
Lrrc69	NA	NA	0	0	4.65
Lrrc7	NA	NA	NA	0.65	NA
Lrrc71	NA	NA	1.35	NA	NA
Lrrc73	NA	NA	2.86	1.6	NA
Lrrc8a	NA	NA	4.9	1.24	0
Lrrc8b	NA	NA	NA	1.74	NA
Lrrc8c	NA	NA	1.05	2.3	2.16
Lrrc8d	NA	3.02	2.82	0	7.65
Lrrc8e	NA	1.58	0.83	0.27	0
Lrrc9	NA	NA	NA	1.5	NA
Lrrcc1	NA	NA	0.11	0.51	1.04
Lrrd1	NA	NA	NA	0.27	NA

Lrrfip1	0	0.78	0	0.39	2.78
Lrrfip2	0.44	0.97	2.18	0.9	0
Lrriq1	NA	NA	NA	0.26	NA
Lrriq2	0	NA	NA	NA	NA
Lrriq3	NA	NA	0.97	1.8	1.87
Lrrk1	2.51	0.07	0.46	1.92	0.86
Lrrk2	NA	NA	1.48	0.67	1.97
Lrrn1	0.71	NA	1.4	0	0
Lrrn2	0.29	0.57	0.25	1.17	0
Lrrn3	0.87	0	2.14	1.49	4.22
Lrrn4	NA	NA	2.52	4.32	NA
Lrrn4cl	NA	NA	1.24	1.89	NA
Lrrtm1	0	NA	0.31	0	1.94
Lrrtm2	4.14	1.21	0.6	0.91	0.13
Lrrtm3	2.71	NA	NA	1.69	NA
Lrrtm4	0.7	2.3	0.63	1.79	0.28
Lrsam1	0.45	NA	0.37	0	0
Lrtm1	NA	0.27	0.61	0.13	2.15
Lrtm2	NA	NA	2.49	1.42	NA
Lrwd1	NA	NA	2.1	0.94	1.58
Lsamp	0.6	0.68	0.06	0.48	2.26
Lsg1	NA	3.31	0.99	1.24	3.78
Lsm1	0	2.28	0.69	1.31	0
Lsm10	1.2	NA	2.8	1.29	3.95
Lsm11	1.76	NA	0.78	4.67	0.82
Lsm12	NA	1.17	0.12	2.39	0
Lsm14a	NA	0	1.62	0	2.54
Lsm14b	NA	0	1.39	0.69	1.59
Lsm2	0.46	1.35	1.47	0	3.29
Lsm3	0.75	2.04	2.7	0	0
Lsm4	0.21	1.59	0.01	0.66	0
Lsm5	1.54	0.75	1.49	0	0
Lsm6	1.42	1.52	1.09	0	1.18
Lsm7	0.09	0.88	2.92	0.37	2.06
Lsm8	0.11	NA	NA	NA	NA
Lsmd1	NA	NA	1.57	0	0.05
Lsmem1	NA	NA	NA	0	NA
Lsp1	0.05	1.76	4.03	1.95	0
Lsr	NA	1.99	0.84	2.59	0
Lss	2.33	1.35	1.26	0.02	1.11
Lst1	0.69	1.41	3.22	0.81	0

Lta	1.21	0.23	3.37	0	1.74
Lta4h	0.4	0.74	1.82	0.55	0
Ltb	2.89	0	1.84	0.85	1.13
Ltb4dh	1.68	NA	NA	NA	NA
Ltb4r2	0.07	0	0	0.93	0
Ltbp1	0.47	1.63	0.67	0	0
Ltbp2	0	1.59	1.67	0.67	0
Ltbp3	0.45	0	1.51	0.88	0
Ltbp4	0.51	1.03	2.62	0.73	1.37
Ltbr	0.15	0.56	0.28	3.19	4.02
Ltc4s	2.33	0.26	1.37	NA	0.83
Ltf	0.76	1.54	2.79	1.32	0
Ltk	1.06	0	1.41	2.51	0.94
Ltn1	NA	0.76	0.78	0	NA
Ltv1	NA	NA	1.71	1.77	1.44
Luc7l	0.12	0	0.32	0.7	0
Luc7l2	0.45	0	1.2	1.58	1.45
Luc7l3	NA	0.21	0.61	0.75	0
Lum	1.81	0	0.65	0.6	0.88
Lurap1	NA	NA	1.65	0.78	NA
Lurap1l	NA	NA	0.37	0.42	NA
Luzp1	0.72	2.63	1.09	1.2	0
Luzp2	0.82	2.63	1.23	2.6	0
Lxn	0.32	0	0.97	1.25	0
Ly6d	0.17	1.58	5.6	2.15	1.68
Ly6e	2.94	1.33	3.84	0.05	0.92
Ly6g5b	3.28	NA	NA	3.96	NA
Ly6g5c	0	NA	NA	0	NA
Ly6g6c	2.18	1.34	0.78	1.2	1.91
Ly6g6d	1.08	4.13	0.42	0.16	3.57
Ly6g6e	0	1.36	0.65	0.43	21.54
Ly6g6f	NA	NA	NA	1.06	NA
Ly6h	0.35	1.33	2.91	1.33	NA
Ly6k	NA	NA	0	1	0
Ly75	0	1.67	1.11	3.56	3.11
Ly86	0.21	1.14	0.74	0.24	0
Ly9	1.88	0	0.52	1.79	0
Ly96	1.3	0	1.33	0.18	0
Lyar	0	NA	4.01	2.49	3.14
Lyg1	NA	NA	2.17	1.22	0
Lyg2	NA	NA	1.26	2.12	NA

Lyl1	0	0.54	4.61	0.15	0
Lyn	3.19	0.25	7.23	1.47	NA
Lynx1	0.57	NA	0	0	1.82
Lypd1	NA	0.66	1.68	0.2	1.58
Lypd2	NA	NA	NA	1.48	NA
Lypd3	NA	0.69	2.42	1.43	5.38
Lypd4	NA	NA	1.1	0	2
Lypd5	NA	NA	0.32	1.25	0
Lypd6	NA	NA	0.72	0.49	2.33
Lypd6b	NA	NA	0.2	1.87	10.33
Lypd8	NA	NA	0.5	NA	NA
Lypla1	0.4	0	1.21	0.76	1.69
Lypla2	0.1	0.61	0.91	0.31	1.49
Lypla3	0.24	NA	NA	NA	NA
Lyplal1	0	NA	1.53	8.61	0
Lyrm1	NA	2.41	1.06	3.18	0
Lyrm2	NA	0	2.01	0.8	1.09
Lyrm4	NA	0	1.18	0	0
Lyrm5	NA	NA	2.31	1.22	1.94
Lyrm7	NA	NA	1.83	4.26	16.64
Lyrm9	NA	0.56	0.46	0.12	NA
Lysmd1	NA	NA	0.28	1.22	3.25
Lysmd2	NA	NA	1.83	0.28	0
Lysmd3	NA	NA	1.18	0.47	1.34
Lysmd4	NA	NA	2.2	0.71	0.52
Lyst	0.24	0	0.72	0.22	0
Lyve1	NA	2.35	1.85	1.57	1
Lyzl1	1.9	NA	NA	0.67	NA
Lyzl4	2.36	NA	0	2.19	9.49
Lyzl6	0.34	0.14	4.73	0.97	0
Lzic	0	NA	0.24	1	0
Lztfl1	0.44	1.16	1.04	0.73	0
Lztr1	2.57	0.38	2.75	1.59	3.26
Lzts1	0	NA	NA	0.45	NA
Lzts2	0	NA	1.69	1.15	NA
Lzts3	NA	0	7.4	3.46	NA
M1ap	NA	NA	1.98	12.86	NA
M6pr	0.98	1.72	0.52	NA	4.49
M6prbp1	0	NA	NA	NA	NA
Maats1	NA	NA	0.98	0.94	NA
Mab21l1	0	0.17	1.43	1.3	0

Mab21l2	0.56	1.15	0	2.56	1.72
Mab21l3	NA	NA	1.14	2.04	NA
Macc1	NA	NA	1.5	1.9	11.92
Macf1	0	0.72	0.95	0	1.96
Macrod1	NA	0	1.86	0.97	3.7
Macrod2	NA	NA	1.22	0.05	0.44
Mad1l1	0	0	0.34	0	0
Mad2l1	0.08	0.63	2.01	1.2	0
Mad2l1bp	0.84	4.39	1.72	0	4.09
Mad2l2	0.89	NA	1.49	2.15	2.23
Madcam1	2.92	4.36	0.96	1.38	1.37
Madd	0	0	2.1	0	2.25
Maea	1.5	1.21	0.87	0.45	0
Mael	NA	NA	0.17	3.08	0
Maf	0	1.19	3.44	0.68	0
Maf1	0.06	NA	0.89	1.01	1.88
Mafa	3.27	NA	NA	1.65	NA
Mafb	0.31	0.14	8.28	3.11	1.51
Maff	0	1.56	1.24	2.75	0
Mafg	0.72	0	1.01	3.09	0
Mafk	0	1.18	1.54	NA	1.93
Mag	1.29	0.36	1.17	1.07	0
Magea1	0	NA	NA	NA	NA
Magea10	NA	NA	NA	0.18	NA
Magea2	0.92	NA	NA	NA	NA
Magea3	3.65	NA	NA	NA	NA
Magea4	2.46	2.5	2.78	1.3	1.52
Magea5	0.91	0.54	2.11	5.76	0
Magea6	0.08	0	0.59	0.53	1.81
Magea8	0.79	0	1.02	0.28	NA
Magea9	0.88	NA	NA	NA	NA
Mageb1	0	NA	NA	NA	NA
Mageb18	NA	NA	NA	1.61	NA
Mageb2	0.04	NA	NA	NA	NA
Mageb3	0.57	0	0.92	0.23	3.72
Mageb4	NA	NA	NA	0	NA
Mageb5	2.54	NA	NA	NA	NA
Maged1	0.64	1.39	2.28	0	0
Maged2	1.38	0	1.33	0.23	0
Magee1	2.43	NA	1.46	0.47	0
Magee2	NA	NA	NA	0.97	NA

Magefl	NA	0	4.13	2.71	9.47
Mageh1	0	0	1.89	1.38	4.67
Magel2	0	1.94	0.95	1.05	0
Magi1	0.72	0.88	0.42	2.25	1.12
Magi2	NA	0.77	2.27	0.6	0
Magi3	NA	NA	1.95	0	0.88
Magix	NA	3.21	0.11	0	0
Magmas	0.36	NA	NA	NA	NA
Magoh	0.28	0.01	0.42	0	1.21
Magohb	NA	2.46	1.36	0.61	0
Magt1	NA	0	1.54	0.2	0.83
Mak	0.3	0.61	1.72	1.76	0
Mak10	0.35	NA	NA	NA	NA
Mak16	NA	0.08	2.24	NA	0
Mal	0.37	1.8	0.21	3.17	2.55
Mal2	0.23	NA	1.73	1.86	0
Malat1	NA	NA	1.97	0.98	0.63
Mall	NA	0.08	1.15	1.27	0
Malsu1	NA	NA	1.16	1.48	NA
Malt1	1.06	0.95	3.91	0	2.68
Mamdc1	0.25	NA	NA	NA	NA
Mamdc2	1.3	NA	1.03	0	0.63
Mamdc4	NA	NA	5.74	1.43	0.9
Maml1	2.89	0	1.44	1.22	0
Maml2	NA	NA	3.33	0.52	0
Maml3	NA	NA	NA	1.38	NA
Mamld1	NA	0.41	1.42	1.32	3.3
Mamstr	NA	NA	0	1.17	NA
Man1a2	NA	0.71	0.81	0.09	0
Man1b1	NA	0	1.02	1.02	0
Man1c1	0.41	2.57	1.99	0.99	0
Man2a1	0.46	2.56	1.96	0.96	6.71
Man2a2	0	1.29	0.79	0.98	1.18
Man2b1	1.16	0	1.01	1.41	2.62
Man2b2	0.21	0	2.01	0.19	1.44
Man2c1	6.22	0	0.58	0.71	1.37
Manba	0.05	0.66	2.63	0.05	0
Manbal	0.76	NA	1.4	0.22	0
Manea	0.59	1.37	1.38	0.88	0
Maneal	NA	NA	0.49	1.35	NA
Manf	NA	0.19	1.48	1.01	1.28

Mansc1	5.64	4.11	2.71	3.04	3.33
Maoa	0.58	0	0	0.61	3.08
Maob	1.32	2.33	1.68	1.89	1.49
Map10	NA	NA	0.62	0.61	NA
Map1a	NA	1.26	2.21	0	NA
Map1b	NA	0.46	0.68	3.79	NA
Map1lc3a	0.48	NA	2.05	0.91	0
Map1lc3b	0.64	1.28	0.38	1.71	0
Map1s	NA	1.29	0	1.25	NA
Map2	NA	1	2.01	1.81	NA
Map2k1	2.88	1.39	2.52	NA	0.59
Map2k1ip1	0.16	NA	NA	NA	NA
Map2k2	0	0.9	1.34	0.7	0.11
Map2k3	0.53	0.63	1.22	0	0.08
Map2k4	0.74	1.58	2.11	0.14	2.03
Map2k5	0	0	0.92	0	0.75
Map2k6	0.63	3.37	4.78	0.77	0
Map2k7	0.33	0.64	0.47	NA	0.88
Map3k1	0	1.7	2.43	1.12	0
Map3k10	0.54	0.83	0.93	2.33	0
Map3k11	2.48	1.47	1.5	1.58	0
Map3k12	0	1.23	0.37	2.13	0
Map3k13	0.97	NA	NA	1.59	NA
Map3k14	0	0	1.69	NA	2.77
Map3k15	NA	NA	NA	0.9	NA
Map3k19	NA	NA	NA	2.24	NA
Map3k2	0	0.32	0.52	0.55	0.04
Map3k3	0	0.53	1.69	NA	3.39
Map3k4	0	0	0.93	0.31	0
Map3k5	1.36	0.39	1.25	0.52	0
Map3k6	1.05	1.2	2.31	1.16	0.65
Map3k7	0.69	0.77	1.03	0.42	0.93
Map3k7cl	NA	3.76	9.28	4.79	NA
Map3k7ip1	0.05	NA	NA	NA	NA
Map3k7ip2	0	NA	NA	NA	NA
Map3k8	0	1.58	0.71	1.88	0
Map3k9	2.2	1.1	1.2	1.94	0
Map4	NA	0.43	0.06	0	NA
Map4k1	0.27	0	0.1	1.5	0
Map4k2	0.52	0.1	1.35	1.68	0.63
Map4k3	1.42	0.71	0.82	10.88	0

Map4k4	1.33	0.71	0	0.74	0
Map4k5	0.42	1.03	2.12	2.32	0
Map6	NA	NA	0.06	1.62	NA
Map6d1	NA	0	0.94	1.17	0.8
Map7	NA	0	1.88	1.71	NA
Map7d1	NA	3.23	0.33	0.54	NA
Map7d2	NA	NA	0.08	3.04	NA
Map9	NA	0.71	0	0.66	NA
Mapbpip	0.09	NA	NA	NA	NA
Mapk1	0.06	1.98	0.65	0.07	0.55
Mapk10	0	0.99	1.05	0.36	1.83
Mapk11	0.92	1.58	2.31	2.22	0
Mapk12	1.17	NA	0.79	0.97	1.47
Mapk13	0	0.29	1.48	2.46	0
Mapk14	0.26	1.5	1.53	0.47	0
Mapk15	NA	NA	2.72	3.64	4.5
Mapk1ip1l	NA	0.55	0.49	2.69	0
Mapk3	0	0.84	0.99	1.42	0
Mapk4	2.19	1.01	1.38	2.58	0
Mapk6	1.13	1.16	1.18	5.2	1.68
Mapk7	0	1.34	1.34	0.9	0
Mapk8	0.47	0.37	0.94	0	0.08
Mapk8ip1	NA	1.42	1.13	1.25	1.98
Mapk8ip2	1.09	0	0.4	1.14	1.72
Mapk8ip3	0.21	1.78	1.04	0.23	0.49
Mapk9	0	0	1.24	0.01	1.87
Mapkap1	0	0	0.9	0.78	1.79
Mapkapk2	0.52	1.85	0.9	0.92	0
Mapkapk3	0.77	0.8	3.68	1.85	1.9
Mapkapk5	3.46	2.82	0.17	1.2	3.81
Mapkbp1	1.39	1.14	1.19	0.43	1.69
Mapksp1	NA	NA	NA	NA	0.72
Mapre1	0	0.82	1.27	0.66	0
Mapre2	0	0	1.2	0.96	0
Mapre3	0.48	1.3	1.5	0.97	0
Mapt	0.14	0.8	0.88	7.18	0
41699	NA	2.51	1.38	2.15	NA
41700	NA	2.82	2.09	2.21	NA
41699	NA	0.34	2.38	0.2	1.65
41708	NA	NA	0.44	0.78	0
41709	NA	NA	1.35	0.54	0

41700	NA	0.74	1.55	1.19	0
41701	NA	0.86	0	0	2.7
41702	NA	NA	0.58	1.23	0.81
41703	NA	1.75	1.78	0.72	1.44
41704	NA	0.59	1.97	0	1.22
41705	NA	0.86	2.24	0.02	0.13
41706	NA	0.09	1.72	0	0.28
41707	NA	NA	1.76	2.24	1.87
Marcks	0.45	1.35	1.69	0	0
Marcksl1	NA	2.79	0.89	1.98	11.49
Marco	0.32	0	0	0.51	0
Mark1	0.52	0	0.42	1.37	0.18
Mark2	1.7	0.49	0.75	1.45	0
Mark3	0	3.87	1.76	0.64	NA
Mark4	0	0.74	0.43	1.53	0.22
Mars	NA	1.57	0.93	0	0
Mars2	NA	NA	1.87	1.13	0.12
Marveld1	0	NA	0.85	1.88	0.78
Marveld2	0.49	NA	1.93	0.34	2.68
Marveld3	0.61	NA	0.66	0.24	0
Mas1	0.79	0.02	0.05	1.54	4.47
Masp1	0.41	0.79	0.36	1.62	0
Masp2	0	1.45	1	NA	1.14
Mast1	14.82	1.48	0.28	0.19	0
Mast2	0.56	1	0.59	1.52	0
Mast3	0.31	0.62	1.68	0.94	0.26
Mast4	NA	0.77	0.67	3.6	0.69
Mastl	0.65	NA	2.76	1.73	0
Mat1a	0	2.27	1.74	0.13	1.15
Mat2a	0.52	1.36	1.04	0.17	0
Mat2b	0.35	0.46	0	4.11	1.76
Matk	0.08	0	9.7	0.06	3.19
Matn1	0	0.65	4.75	NA	0
Matn2	0.67	1.15	1.21	3.75	0
Matn3	0	1.65	0.61	0.76	1.82
Matn4	1.03	0	2.14	2.78	0
Matr3	0.14	0.51	1.87	NA	0
Mau2	NA	0.71	0.61	0	NA
Mavs	NA	0.16	0.74	2.42	0
Max	0.69	0	0.09	1.04	NA
Maz	0.56	0	3.9	1.96	0

Mb	0	0.63	0	3.08	3.12
Mb21d1	NA	NA	3.58	0.62	NA
Mb21d2	NA	NA	3.44	7.42	NA
Mbd1	0.39	1.23	1.49	4.11	0
Mbd2	0.52	0.57	0.1	1.9	0
Mbd3	0.02	0.69	1.19	2.42	1.36
Mbd3l1	0.73	NA	1.57	2.99	NA
Mbd3l2	NA	NA	0.63	NA	NA
Mbd4	0.35	2.05	0	NA	2.43
Mbd5	0	0	0.98	1.24	0
Mbd6	NA	NA	1.94	1.14	1.02
Mbip	0	0.98	0	1.12	2.75
Mbl2	1.46	2.41	0.89	4.49	2.3
Mblac1	NA	NA	1.75	1.56	NA
Mblac2	NA	NA	0.17	0	0
Mbnl1	2.17	0.54	2.09	0.98	0
Mbnl2	0.68	0.18	0.37	3.01	0
Mbnl3	0.01	0	0.97	0.27	1.39
Mboat1	NA	NA	1.99	1.15	1.04
Mboat2	NA	3.21	2.51	1.44	2.17
Mboat4	NA	NA	NA	1.04	NA
Mboat7	NA	1.26	0.35	3.19	0
Mbp	0	0.96	0.66	1.14	0.61
Mbtd1	0.56	1.67	0.53	0.3	0
Mbtps1	0.06	0	0	3.41	0
Mbtps2	0	0	1.3	NA	1.83
Mc1r	0	0.01	1.28	0.34	0
Mc2r	2.52	1.14	0.23	0	4.37
Mc3r	0	1.26	0.52	2.13	4.45
Mc4r	0.87	NA	NA	0.2	NA
Mc5r	0.62	2.06	0.01	1.43	0
Mcam	2.24	0.42	0.87	0.98	1.52
Mcart1	0.1	NA	NA	NA	0
Mcat	NA	1.82	0.85	0	1.21
Mcc	0.48	0.73	0.1	0.57	1.01
Mccc1	0	0	0.36	0.86	6.11
Mccc2	0.36	0	1.26	0.48	1.56
Mcee	0.17	NA	0.44	5.29	3.25
Mcf2	1.78	0	0.21	2.72	1.8
Mcf2l	1.63	0	1.37	2.17	0
Mcfd2	0	0.98	1.8	0.54	0

Mchr1	NA	1.38	0.41	5.25	1.83
Mcl1	1.08	1.59	0.33	0.5	0
Mcm10	0	2.26	0.64	1.42	0
Mcm2	0.49	0	2.51	0.27	0
Mcm3	0.26	0	1.67	0	0
Mcm3ap	1.52	0.83	2.47	0	0.07
Mcm4	0	0.38	3.12	0	0
Mcm5	0.24	0.77	2.28	0	1.75
Mcm6	0.05	0	1.29	2.67	0.72
Mcm7	0.68	0	2.03	0.06	3.32
Mcm8	3.1	NA	0.8	0.62	0
Mcm9	NA	1.44	0.38	0	0
Mcmbp	NA	2.13	0.9	0	NA
Mcmdc1	3.17	NA	NA	NA	NA
Mcmdc2	NA	NA	NA	3.34	NA
Mcoln1	1.94	0.39	0.27	1.85	1.76
Mcoln2	0.01	NA	1.71	4.74	2.45
Mcoln3	0.96	2.93	1.55	1.9	0
Mcph1	0.99	0.48	2.2	0.2	0
Mcrs1	0	2.02	1.13	0.74	2.75
Mctp1	NA	2.56	0.27	0	0
Mctp2	NA	0	0.76	1.63	0
Mcts1	0.7	0	1.21	0	0
Mcu	NA	NA	0.4	1.79	NA
Mcur1	NA	3.09	0.35	0.52	NA
Mdc1	NA	0	2.54	0.41	0.77
Mdfi	0	2.05	1.22	0.67	0
Mdfic	NA	2.94	1.55	1.72	0
Mdga1	0.37	NA	0.01	1.98	0
Mdga2	NA	NA	1.73	0	0.31
Mdh1	0.21	1.71	0.04	3.82	0
Mdh1b	0.61	NA	0.24	1.26	2.56
Mdh2	0	2.4	0.72	0.41	2.22
Mdk	1.21	3.49	6.36	0	0
Mdm1	0.52	0.63	0.04	0.49	0
Mdm2	0.06	0.88	0.94	0.24	0.1
Mdm4	1.2	0.77	0.74	0	0.8
Mdn1	1.38	2.19	0.5	7.53	3.93
Mdp1	NA	NA	0.62	0	0
Mds1	4.06	NA	NA	NA	NA
Me1	NA	1.03	3.89	1.03	NA

Me2	0.34	1.83	1.6	0.52	0.18
Me3	NA	0	2.45	0.97	3.21
Mea1	0	0.78	1.31	NA	3.01
Meaf6	NA	0	1.86	0.73	0
Mecom	NA	0.27	0.52	0.43	1.46
Mecp2	0	0	1.59	1.69	0.76
Mecr	NA	1.05	0.68	0	0
Med1	NA	0.01	0	1.45	1.05
Med10	NA	NA	0.12	1.22	NA
Med11	NA	NA	2.74	1.15	1.37
Med12	NA	1.84	1.98	2.38	0
Med12l	NA	NA	0.3	0	0.32
Med13	NA	1.22	0.59	0.48	0
Med13l	NA	0.58	1.41	1.86	1.53
Med14	NA	0.37	0.17	1.03	0
Med15	NA	1.31	0.31	0	3.55
Med16	NA	1.37	1.14	0	3.95
Med17	NA	0.24	0.49	0.44	1.54
Med18	NA	2.53	1.9	1.1	0
Med19	NA	NA	1.16	0.16	0.81
Med20	NA	1.02	1.87	0.72	0
Med21	NA	0	2.2	0.1	4.36
Med22	NA	1.03	2.48	2.26	0
Med23	NA	0.98	1.53	1.18	0
Med24	NA	0	0.03	1.45	0
Med25	NA	0	0.65	1.59	0
Med26	NA	NA	0.78	0.66	1.55
Med27	NA	1.18	1.62	0.77	1.26
Med28	NA	2.28	2.34	3.05	0
Med29	NA	NA	1.16	0.66	3.62
Med30	NA	NA	0.64	1.32	0.16
Med31	NA	3.32	2.02	NA	1.62
Med4	NA	0.78	0.14	0.88	3.75
Med6	0.36	0.41	1.27	0.5	0
Med7	NA	0.14	2.87	0.88	4.19
Med8	NA	0.06	0.01	2.25	0.57
Med9	NA	2.09	2.94	4.43	1.09
Medag	NA	NA	1.51	1.69	NA
Mef2a	1.05	0	3.81	1.06	0
Mef2b	1.49	3.89	4.09	NA	NA
Mef2c	0	0.16	1.77	1.19	0

Mef2d	0	1.81	0.86	1.9	0.82
Mefv	0.56	4.63	2.07	0.55	0
Meg3	NA	0.57	0.62	2.77	0
Megf10	NA	NA	0.58	0.09	0.8
Megf11	NA	NA	0.48	0.38	NA
Megf6	NA	0	0.36	0.71	0
Megf8	NA	0.81	1.85	0.79	2.2
Megf9	NA	0.49	0.82	1.41	0.22
Mei1	0.51	NA	1.72	1.95	0
Meig1	0.42	NA	2.84	0	0
Meiob	NA	NA	0.71	2.02	NA
Meis1	0.93	0	0.93	0	0
Meis2	NA	0.86	6.13	1.19	1.24
Meis3	NA	NA	0.64	0.79	0.18
Melk	1.28	0.47	1.73	0.84	1.91
Memo1	NA	NA	NA	0	NA
Men1	1.85	0	0.23	4.47	3.55
Meox1	0.94	4.14	0.36	1.03	0.24
Meox2	1.23	0.09	0	2.39	0.01
Mep1a	0	1.04	1.5	0.41	0
Mep1b	1.35	2.16	1.79	0.08	5.1
Mepce	NA	0	0.78	1.41	0
Mepe	2.03	0.38	2.28	0	1.33
Mertk	0	0.24	3.3	0	0
Mesdc1	1.29	NA	2.14	1.66	0.48
Mesdc2	2.64	NA	1.19	1.06	0.12
Mesp1	3.51	NA	2.14	1.26	0
Mesp2	0.18	NA	2.08	0.09	NA
Mest	0.36	0	0.38	1.86	0
Met	0.08	0.53	2.27	1.75	2.56
Metap1	0	0	0.72	0.76	3.44
Metap1d	NA	NA	0.25	4.01	NA
Metap2	0	0.76	0.6	0.6	0
Metrn	0.98	1.25	1.56	0.65	0.93
Metrnl	NA	NA	0.9	1.21	1.23
Mett10d	NA	NA	NA	NA	3.57
Mett5d1	NA	NA	NA	NA	0.31
Mettl1	0.83	1.98	0.09	2.85	0
Mettl10	NA	1.14	0.52	0.45	3.17
Mettl11a	NA	NA	NA	NA	4.75
Mettl13	NA	0	0.85	3.74	0

Mettl14	NA	NA	0.76	0.81	0
Mettl15	NA	NA	0.87	0.49	NA
Mettl16	NA	0	0	0.85	NA
Mettl17	NA	0	0.12	1.57	NA
Mettl18	NA	2.39	0	0.93	NA
Mettl20	NA	NA	1.05	1.77	NA
Mettl21a	NA	NA	1.49	1.13	NA
Mettl21c	NA	NA	NA	0.75	NA
Mettl21d	NA	1.29	1.64	0.51	NA
Mettl22	NA	1.66	0.28	1.21	NA
Mettl23	NA	NA	1.09	1.21	NA
Mettl24	NA	NA	NA	1.31	NA
Mettl25	NA	NA	2.79	0.72	NA
Mettl3	0.23	0	2.86	NA	0
Mettl4	0.73	0.1	2.65	1.25	0
Mettl5	NA	0.4	2.14	0.55	0
Mettl6	NA	NA	0.51	1.81	2.58
Mettl7b	NA	NA	1.2	2.26	2.07
Mettl8	NA	0	1.03	2.37	0.9
Mettl9	NA	0	2.99	0.61	0
Mex3a	NA	NA	0.4	NA	0
Mex3b	NA	NA	4.38	1.88	4.63
Mex3c	NA	0	1.39	1.6	0.89
Mex3d	NA	1.38	2.53	1.95	1.92
Mfap1	0.37	NA	NA	NA	NA
Mfap2	0.96	1.35	0.57	1.9	2.7
Mfap3	0.72	1.41	1.54	0.72	2.28
Mfap3l	NA	0.67	2.29	1.05	0
Mfap4	4.08	2.19	1.53	1.22	4.95
Mfap5	5.67	0.48	0.81	1.54	1.69
Mff	NA	1.55	2.23	0.97	0
Mfge8	1.02	0.27	3.1	0.35	4.87
Mfhas1	0.61	0	0	1.54	6.61
Mfi2	0.09	1.14	1.73	0.91	1.3
Mfn1	0.55	1.07	1.01	0.76	1.47
Mfn2	0.62	0.77	0.05	0	0.09
Mfng	0	0	4.19	0.52	NA
Mfrp	2.06	NA	NA	NA	NA
Mfsd1	NA	1.16	3.64	0.64	3.08
Mfsd10	NA	0.75	2.72	3.07	3.46
Mfsd11	NA	1.83	3.6	2.01	0

Mfsd12	NA	2.87	1.53	2.23	NA
Mfsd2a	NA	NA	1.08	1.86	NA
Mfsd2b	NA	2.01	2.73	1.12	NA
Mfsd3	NA	NA	1.18	NA	1.58
Mfsd4	NA	NA	0	0.33	0.88
Mfsd5	NA	1.86	1	2.33	0
Mfsd6	NA	2.3	2.33	0	3.64
Mfsd6l	NA	NA	0	2.02	NA
Mfsd8	NA	NA	1.88	0.03	0
Mfsd9	NA	1.37	0.1	1.28	0.13
Mga	0	0	1.22	2.45	0
Mgam	0	0.23	6.26	0	0
Mgarp	NA	NA	0.89	2.89	NA
Mgat1	0.37	3.31	0.69	2.47	0
Mgat2	0.47	1.02	2.37	1.1	0
Mgat3	0.92	1.4	1.38	1.9	3.26
Mgat4a	0.55	0.62	1.22	1.36	2.81
Mgat4b	0.44	2.19	2.33	0.97	2.56
Mgat4c	NA	0.98	0.38	1.75	1.28
Mgat5	0	1.29	1.09	4.69	0
Mgat5b	NA	NA	0.53	1.85	9.13
Mgea5	0.13	0.99	1.08	1.52	0.25
Mgll	1.37	1.03	0.04	0	0.49
Mgme1	NA	NA	0.43	0.11	NA
Mgmt	0	0	3.56	1.2	0
Mgp	NA	1.57	0.5	5.53	0.07
Mgrn1	0.29	0.24	0.27	1.55	0.18
Mgst1	0.48	NA	0.93	1.69	0.65
Mgst2	0.4	2.38	1.98	2.27	4.04
Mgst3	0.35	2.13	2.13	0.76	0.74
Mia	NA	0.77	0.91	0.8	NA
Mia2	3.21	1.36	0	0.7	0.02
Mia3	0.61	0.66	0.88	0.12	0.09
Miat	NA	NA	1.69	NA	NA
Mib1	0	NA	1.04	0	0
Mib2	NA	NA	1.88	0.38	0
Mical1	NA	2.81	2	0.06	1.29
Mical2	NA	2.09	2.46	1.92	1.7
Mical3	1.14	0	1.03	0	0
Micalcl	NA	NA	NA	2.05	0.32
Micall1	NA	0	2.49	0.99	0

Micall2	NA	0.79	0	0.77	0
Micu1	NA	1.55	1.12	1.24	NA
Micu2	NA	1.99	2.05	5.18	NA
Micu3	NA	NA	2.53	0.52	NA
Mid1	0.94	0.23	0.94	1.88	0
Mid1ip1	0.13	0	0.36	0	0
Mid2	0.05	1.35	0.59	0.56	2.27
Midn	0.06	NA	1.68	1.58	0
Mien1	NA	NA	1.26	0.69	NA
Mier1	NA	NA	0.89	0.71	0
Mier2	NA	0.1	3.82	2.35	1.76
Mier3	NA	NA	4.37	0.95	3.94
Mif	2.08	1.42	0.02	15.69	2.63
Mif4gd	NA	NA	1.49	2.68	8.47
Miip	NA	2.36	1.38	1.87	1.7
Milr1	NA	0	3.04	0.59	NA
Mina	0	0	0.3	NA	0
Mink1	NA	1.17	0.43	0.85	0.89
Minos1	NA	NA	0.56	0.13	NA
Minpp1	0.24	0.85	0.13	0	0
Mios	NA	0.91	1.81	0.75	0.77
Miox	NA	NA	1.75	1.93	0
Mip	0.76	1.57	0.98	0.82	0
Mipep	0	0	2.16	0	1.85
Mipol1	0.05	NA	0.76	NA	0.57
Mir100	NA	NA	NA	2.35	NA
Mir106a	NA	NA	NA	0.95	NA
Mir106b	NA	NA	NA	2.03	NA
Mir107	NA	NA	NA	1.17	NA
Mir10a	NA	NA	NA	0	NA
Mir10b	NA	NA	NA	0.6	NA
Mir125a	NA	NA	NA	0.49	NA
Mir126	NA	NA	NA	0	NA
Mir127	NA	NA	NA	0.87	NA
Mir128-1	NA	NA	NA	0.5	NA
Mir128-2	NA	NA	NA	0.81	NA
Mir129-1	NA	NA	NA	0	NA
Mir129-2	NA	NA	NA	1.18	NA
Mir130a	NA	NA	NA	0	NA
Mir130b	NA	NA	NA	1.41	NA
Mir132	NA	NA	NA	0.64	NA

Mir133b	NA	NA	NA	2.46	NA
Mir134	NA	NA	NA	2.85	NA
Mir136	NA	NA	NA	3.5	NA
Mir137	NA	NA	NA	0.37	NA
Mir138-1	NA	NA	NA	0.78	NA
Mir138-2	NA	NA	NA	0.79	NA
Mir139	NA	NA	NA	1.09	NA
Mir140	NA	NA	NA	0.67	NA
Mir141	NA	NA	NA	3.27	NA
Mir142	NA	NA	NA	0	NA
Mir144	NA	NA	NA	0.82	NA
Mir148a	NA	NA	NA	5.34	NA
Mir148b	NA	NA	NA	0	NA
Mir149	NA	NA	NA	1.01	NA
Mir150	NA	NA	NA	0	NA
Mir152	NA	NA	NA	0.5	NA
Mir154	NA	NA	NA	5	NA
Mir155	NA	NA	NA	0	NA
Mir15a	NA	NA	NA	0	NA
Mir15b	NA	NA	NA	3.02	NA
Mir16	0.01	NA	NA	NA	NA
Mir16-1	NA	NA	NA	2.3	NA
Mir16-2	NA	NA	NA	1.01	NA
Mir17hg	NA	NA	2.13	0.62	NA
Mir181c	NA	NA	NA	1.56	NA
Mir182	NA	NA	NA	3.04	NA
Mir183	NA	NA	NA	0.7	NA
Mir184	NA	NA	NA	1.34	NA
Mir185	NA	NA	NA	2	NA
Mir186	NA	NA	NA	4.24	NA
Mir187	NA	NA	NA	1.27	NA
Mir188	NA	NA	NA	0	NA
Mir191	NA	NA	NA	3.47	NA
Mir192	NA	NA	NA	0	NA
Mir194-1	NA	NA	NA	0	NA
Mir194-2	NA	NA	NA	1.28	NA
Mir195	NA	NA	NA	1.38	NA
Mir199b	NA	NA	NA	1.84	NA
Mir200a	NA	NA	NA	0.61	NA
Mir200b	NA	NA	NA	4.82	NA
Mir200c	NA	NA	NA	0.18	NA

Mir202	NA	NA	NA	0	NA
Mir203	NA	NA	NA	0.88	NA
Mir204	NA	NA	NA	1.32	NA
Mir206	NA	NA	NA	0.25	NA
Mir208a	NA	NA	NA	3.95	NA
Mir21	NA	NA	NA	0.08	NA
Mir210	NA	NA	NA	0.76	NA
Mir211	NA	NA	NA	1.12	NA
Mir212	NA	NA	NA	1.22	NA
Mir215	NA	NA	NA	1.45	NA
Mir216a	NA	NA	NA	0.67	NA
Mir217	NA	NA	NA	1.66	NA
Mir218-1	NA	NA	NA	25.61	NA
Mir218-2	NA	NA	NA	1.21	NA
Mir219-1	NA	NA	NA	2.6	NA
Mir219-2	NA	NA	NA	0.04	NA
Mir221	NA	NA	NA	0.98	NA
Mir222	NA	NA	NA	0.63	NA
Mir224	NA	NA	NA	1.41	NA
Mir22hg	NA	0.93	1.97	NA	NA
Mir23a	NA	NA	NA	0.27	NA
Mir23b	NA	NA	NA	0.66	NA
Mir24-2	NA	NA	NA	1.33	NA
Mir25	NA	NA	NA	0.9	NA
Mir26b	NA	NA	NA	0.66	NA
Mir27a	NA	NA	NA	0	NA
Mir27b	NA	NA	NA	0	NA
Mir28	NA	NA	NA	0.84	NA
Mir296	NA	NA	NA	0.8	NA
Mir299	NA	NA	NA	0.31	NA
Mir29a	NA	NA	NA	1.85	NA
Mir29c	NA	NA	NA	0	NA
Mir302a	NA	NA	NA	1.69	NA
Mir302b	NA	NA	NA	2.28	NA
Mir30a	NA	NA	NA	1.4	NA
Mir30b	NA	NA	NA	0.58	NA
Mir30d	NA	NA	NA	0.42	NA
Mir30e	NA	NA	NA	0	NA
Mir31	NA	NA	NA	0	NA
Mir32	NA	NA	NA	0.52	NA
Mir326	NA	NA	NA	1.78	NA

Mir330	NA	NA	NA	0.17	NA
Mir331	NA	NA	NA	1.12	NA
Mir339	NA	NA	NA	1.09	NA
Mir34a	NA	NA	NA	1.82	NA
Mir34b	NA	NA	NA	0.55	NA
Mir375	NA	NA	NA	0.15	NA
Mir376c	NA	NA	NA	0.11	NA
Mir377	NA	NA	NA	0	NA
Mir382	NA	NA	NA	1.87	NA
Mir412	NA	NA	NA	0	NA
Mir425	NA	NA	NA	1.24	NA
Mir431	NA	NA	NA	0.15	NA
Mir455	NA	NA	NA	1.53	NA
Mir485	NA	NA	NA	0.34	NA
Mir494	NA	NA	NA	0.63	NA
Mir495	NA	NA	NA	6.04	NA
Mir503	NA	NA	NA	0	NA
Mir504	NA	NA	NA	0.55	NA
Mir671	NA	NA	NA	1.28	NA
Mir675	NA	NA	NA	0.62	NA
Mir7-2	NA	NA	NA	1.59	NA
Mir711	NA	NA	NA	1.25	NA
Mir9-1	NA	NA	NA	2.14	NA
Mir9-3	NA	NA	NA	1.26	NA
Mir93	NA	NA	NA	1.44	NA
Mir96	NA	NA	NA	1.04	NA
Mir98	NA	NA	NA	1.28	NA
Mir99a	NA	NA	NA	0.89	NA
Mir99b	NA	NA	NA	0.28	NA
Mirlet7b	NA	NA	NA	2.6	NA
Mirlet7d	NA	NA	1.02	0.86	NA
Mirlet7e	NA	NA	NA	1.02	NA
Mirlet7g	NA	NA	NA	1.98	NA
Mirlet7i	NA	NA	NA	1.13	NA
Mis12	NA	2.02	1.69	0.32	0
Mis18a	NA	0	2.61	2.09	NA
Mis18bp1	NA	1.06	1.12	1.06	NA
Misp	NA	1.75	2.19	1.09	NA
Mitd1	NA	NA	0.69	NA	0.52
Mitf	0.13	0	2.6	1.18	0.04
Mixl1	2.22	NA	2.89	1.77	0

Mki67	0.72	0.7	3.76	0	0
Mki67ip	0	NA	1.4	1.13	0
Mkks	1.12	1.9	0.21	0	1.4
Mkl1	1.26	1.07	0.22	1.78	0
Mkl2	1.78	0	1.94	0.66	1.62
Mkln1	0	1.87	0.71	0	1.05
Mknk1	0.34	1.97	1.59	0	4.33
Mknk2	0.16	0.64	2.28	1.13	3.46
Mkrn1	0	1.05	0.97	2.87	1.53
Mkrn2	0.03	0.83	1.9	0.85	0
Mkrn3	0	1.04	0.59	0.31	NA
Mks1	NA	0.86	0.69	0.91	0
Mkx	NA	NA	1.82	0.63	0.06
Mlana	0	0.81	1.71	1.87	0
Mlc1	1.8	0.22	0	0	7.33
Mlec	NA	0.79	0.69	4.42	1.56
Mlf1	0	0.45	2.61	3.86	1.4
Mlf1ip	0	1.13	2.22	NA	2.15
Mlf2	0.18	0	3.72	0	0
Mlh1	0.19	0.02	0.88	1.14	0
Mlh3	0.71	1.49	0.31	4.34	1.1
Mlip	NA	2.03	0.87	6.68	NA
Mlkl	NA	NA	1.72	0	0
Mll	0.47	NA	NA	NA	NA
Mll2	NA	NA	NA	NA	0.39
Mll3	NA	NA	NA	NA	0.47
Mll5	1.8	NA	NA	NA	0.59
Mllt1	0	1.6	0.86	3.45	0.68
Mllt10	1.02	0.61	0.09	0.84	0.61
Mllt11	NA	0	0.93	0.7	0
Mllt3	0.91	0	1.78	0.49	3.12
Mllt4	0.46	0.66	0.74	2.36	0
Mllt6	0	NA	0.88	1.64	0
Mllt7	2.52	NA	NA	NA	NA
Mlph	4.06	0.79	0.92	2.1	2.25
Mlst8	NA	0.64	0.02	1.47	0
Mlstd1	3.8	NA	NA	NA	NA
Mlx	NA	0	3.3	0.15	1.23
Mlxip	NA	1.34	0.55	1.5	1.61
Mlxipl	NA	3.07	2.5	1.03	1.39
Mlycd	0.06	0.48	1.21	0	0

Mlze	0.31	NA	NA	NA	NA
Mmaa	0.97	NA	1.25	0	0
Mmab	0.25	NA	1.37	0.85	0.35
Mmachc	NA	1.89	0.42	0.79	0
Mmadhc	NA	0	3.41	0.55	0
Mmd	0	0	0.55	1.45	0.59
Mmd2	0.39	NA	0.73	1.79	2.12
Mme	0	3.56	3.76	1.41	0
Mmel1	NA	NA	2.07	0.89	NA
Mmgt1	NA	NA	2.37	0.57	3.96
Mmp10	0.32	1.2	2.44	0	0
Mmp11	2.92	1.69	2.33	1.63	4.92
Mmp12	0.83	1.02	0	1.56	1.06
Mmp13	0.33	0.12	1.15	0.64	1.44
Mmp14	0	0.7	2.58	0.49	0
Mmp15	1.08	1.93	1.01	1.05	1.39
Mmp16	0	0.59	0.62	3.29	1.33
Mmp17	1.44	0.32	6.83	4.2	0
Mmp19	0.49	1.49	1.94	1.7	3.01
Mmp2	0	1.25	2.33	0.8	0
Mmp20	0	1.18	1.17	0	3.13
Mmp21	0.26	NA	NA	0.81	NA
Mmp24	0.12	0.76	0.58	0.99	0
Mmp25	0.52	NA	NA	0.84	NA
Mmp27	NA	NA	NA	1.1	NA
Mmp28	0.89	NA	NA	1.47	NA
Mmp3	1.79	0	1.98	0.84	9.3
Mmp7	0	2.23	0.26	1.6	4.36
Mmp8	1.32	0.68	0	1.62	0
Mmp9	0.69	0.36	3.71	0.94	0
Mmrn1	0	1.34	2.13	3.3	1.48
Mmrn2	0.48	0.78	0.47	1.73	1.6
Mms19	NA	0	0	NA	0
Mms19l	0	NA	NA	NA	NA
Mms22l	NA	NA	NA	0.14	NA
Mn1	NA	1.49	3.65	0	0
Mnat1	0.4	1.59	2.81	0	0
Mnd1	NA	NA	0.2	NA	0
Mnda	NA	1.47	1.65	0.72	NA
Mnf1	NA	NA	0.35	0	NA
Mns1	0.15	2.91	0.86	1.35	0

Mnt	0	0.67	2.51	0.64	0
Mnx1	NA	0.91	3.01	0.95	2.39
Moap1	0.5	NA	NA	NA	NA
Mob1a	NA	0.12	2.08	0.1	NA
Mob1b	NA	NA	2.27	2.14	NA
Mob2	NA	NA	2.32	0.61	NA
Mob3a	NA	NA	4.62	1.72	NA
Mob3b	NA	1.83	1.8	0	NA
Mob3c	NA	NA	1.4	0	NA
Mob4	NA	0.93	2.1	1.51	NA
Mobk1b	0.25	NA	NA	NA	NA
Mobkl1a	NA	NA	NA	NA	0
Mobkl1b	NA	NA	NA	NA	0.65
Mobkl2a	NA	NA	NA	NA	1.22
Mobkl2b	1.46	NA	NA	NA	1.77
Mobkl2c	0	NA	NA	NA	0
Mobkl3	NA	NA	NA	NA	0
Mobp	0.19	0.8	0.89	0.23	0
Mocos	1.44	1.43	1.11	0.23	0
Mocs1	2.36	1.46	1.92	0.72	0.32
Mocs2	1.05	0.88	1.8	0.65	0
Mocs3	0	0.58	2.07	1.51	1.12
Mog	0.98	0.54	0	1.98	0
Mogat1	0.5	NA	2.57	3.05	NA
Mogat2	0	0.79	1.51	2.84	4.46
Mogs	NA	2.65	1.01	7.6	0
Mon1a	NA	NA	1.61	2.26	5.49
Mon1b	NA	1.72	0.43	NA	0
Mon2	NA	0.71	1.91	0	0.36
Morc1	NA	0.35	0.42	0	0.57
Morc3	NA	0.99	0.99	0.93	0.26
Morc4	NA	0	0.96	1.85	1.41
Morf4l1	0	0.57	3.61	1.04	0.6
Morf4l2	0.27	1.77	1.61	0.61	0.3
Morn1	NA	1.4	0	0.04	0
Morn2	NA	NA	3.6	1.6	4.32
Morn3	NA	NA	1.62	2.72	5.06
Morn4	NA	NA	0.2	0.47	0.83
Morn5	NA	NA	1.36	1.66	0
Mos	0.42	2.65	0.4	1.98	6.32
Mosc2	NA	NA	NA	NA	2.27

Mospd1	0	1.28	1.04	0.39	0
Mospd2	0.98	0.09	2.34	0.65	1
Mospd3	0.01	2.85	0.3	0	0
Mov10	0.51	NA	1.44	0.3	1.41
Mov10l1	0	NA	0.19	0.7	1.27
Moxd1	2.02	2.36	1.25	1.91	1.23
Mpc1	NA	1.92	1.75	1.75	NA
Mpc2	NA	1.98	1.09	0	NA
Mpdu1	0	0	0.25	0	1.91
Mpdz	0.41	0.77	0.05	0	0
Mpeg1	NA	NA	3.28	2.13	0
Mpg	0.88	1.38	0.57	0.62	2.34
Mphosph1	0.01	NA	NA	NA	NA
Mphosph10	0	1.88	0.57	0.36	2.32
Mphosph6	0.43	1.44	2.23	0.63	0.23
Mphosph8	NA	1.04	0.66	NA	0
Mphosph9	0	0	0.54	1.15	0
Mpi	NA	1.13	1.93	0.62	3.18
Mpl	0	0.93	1.52	1.26	1.47
Mplkip	NA	NA	2.21	1.02	NA
Mpnd	NA	NA	1.18	0.66	1.44
Mpo	0	0.28	0.02	0.87	1.13
Mpp1	0	2.18	1.38	0.64	2.36
Mpp2	1.37	0.89	2.25	1.18	4.17
Mpp3	0	2.89	0.81	1.83	3.54
Mpp4	0.36	NA	1.57	0	NA
Mpp5	1.42	0.66	4.29	0.66	1.82
Mpp6	0.44	1.18	0.43	2.55	0.69
Mpp7	0.2	NA	0	1.5	0.72
Mppe1	0.05	0.16	0.37	1.18	0
Mpped1	NA	1.8	2.06	0.93	2.04
Mpped2	NA	0.56	1.19	0.37	1.57
Mprip	NA	0	0.63	2.4	0.63
Mpst	3.77	3.01	1.53	1.18	0.55
Mpv17	0.33	0	3.81	1.56	0
Mpv17l	1.06	NA	1.88	0.9	NA
Mpv17l2	NA	NA	1.42	4.48	2.75
Mpz	0.58	1.16	1.62	3.08	2.77
Mpzl1	0.87	0.87	2.02	1.01	1.68
Mpzl2	NA	0.77	0.54	0.88	0
Mpzl3	NA	NA	2.49	1.46	0

Mr1	0.02	1.82	0.77	3.42	1.55
Mrap	NA	NA	0.75	1.07	NA
Mrap2	NA	NA	1.15	1.25	2.51
Mras	0.46	2.64	1.38	1.4	0.07
Mrc1	0.71	1.1	1.58	1.17	NA
Mrc2	0.54	0.87	1.59	0.61	1.69
Mre11a	0.67	0.4	1.09	0.69	0.93
Mreg	NA	2.38	0.08	1.21	4.15
Mrfap1	NA	NA	1.5	1.26	0
Mrgbp	NA	1.11	1.34	0	NA
Mrgprd	2.02	NA	NA	0.21	NA
Mrgpre	NA	NA	NA	0.57	NA
Mrgprf	0.38	NA	1.58	2.03	1.04
Mrgprg	NA	NA	NA	0	NA
Mrgprx1	NA	NA	NA	0.8	NA
Mrgprx2	NA	NA	NA	0.29	NA
Mri1	NA	NA	1.35	1.15	0
Mrm1	NA	1.51	1.66	1.39	0
Mro	0	NA	1.12	1.29	2.31
Mroh1	NA	NA	0.6	0	NA
Mroh2a	NA	NA	1.45	4.39	NA
Mroh2b	NA	NA	1.41	0	NA
Mroh5	NA	NA	NA	1.17	NA
Mroh8	NA	NA	NA	1.59	NA
Mroh9	NA	0.39	0	1.54	NA
Mrp63	0.55	1.22	0.67	NA	0
Mrpl1	0.17	NA	0.59	1.6	0
Mrpl10	0.89	NA	2.5	0.91	0
Mrpl11	0.19	0.15	1.3	1.12	0
Mrpl12	0.01	1.86	0.65	0.24	0.01
Mrpl13	0	0	0.76	0	2.03
Mrpl14	1.08	NA	2.84	0.39	1.58
Mrpl15	0	0.74	1.02	1.35	0
Mrpl16	0.77	1.61	1.26	1.3	0
Mrpl17	0	0	0.49	0.83	1.89
Mrpl18	0.03	1.74	2.17	1.99	0.32
Mrpl19	0	1.25	0.35	1.08	2.08
Mrpl2	0.57	2.39	0.07	3.16	4.35
Mrpl20	0.53	1.5	1.07	1.41	0.94
Mrpl21	0	NA	0.3	0	3.18
Mrpl22	0.4	0	2.14	0.04	0

Mrpl23	0	0	0.46	1.66	2.59
Mrpl24	0.51	0.86	0.18	NA	2.56
Mrpl27	0.25	NA	0.62	0.65	6.64
Mrpl28	0.75	1.92	2.35	0	2.16
Mrpl3	0.13	1.15	1.93	0.84	3.69
Mrpl30	1.21	NA	0.27	1.25	4.81
Mrpl32	0.28	NA	0.96	1.14	0
Mrpl33	0.57	0.09	0.77	0	0
Mrpl34	0.2	0.79	2.12	0.66	0
Mrpl35	0	0.13	0.34	0.92	0
Mrpl36	0.4	NA	0.8	1.05	0
Mrpl37	0.22	NA	0.3	0.52	0
Mrpl38	0	NA	0	2.33	0
Mrpl39	0.72	1.36	0.03	0.7	0
Mrpl4	0.89	3.18	0.61	1.83	1.07
Mrpl40	1.08	1.11	0.4	0.8	0
Mrpl41	NA	0.4	0.85	1.91	0
Mrpl42	NA	0.42	0.79	0	0
Mrpl43	0.42	NA	1.99	0.99	0
Mrpl44	0.47	0.88	2.8	2.47	3.06
Mrpl45	0	NA	0.4	1.1	2.28
Mrpl46	0.83	2.17	1.61	1.2	0
Mrpl47	NA	NA	0.21	0.94	0.02
Mrpl48	0.23	1.76	0	2.17	3.11
Mrpl49	0.3	0	1.61	0.59	0
Mrpl50	0.67	NA	2.37	1.23	0
Mrpl51	0.1	NA	1.33	0.33	0
Mrpl52	1.04	1.5	1.63	0.09	0.58
Mrpl53	0	NA	NA	NA	NA
Mrpl54	0.47	NA	1.77	1.75	4.64
Mrpl55	0.33	NA	2.59	0.38	1.59
Mrpl9	0	0.49	1.2	0.75	2.56
Mrps10	0	0.99	1.02	0.69	0
Mrps11	0.7	1.73	1.46	0	2
Mrps12	0.04	0.92	2.16	NA	0.07
Mrps14	3.24	0.3	0.52	0.78	0
Mrps15	0.35	0	1.02	0	0
Mrps16	0.08	3.62	0.29	0.89	0
Mrps17	0.28	NA	NA	1.36	NA
Mrps18a	1	0.4	0.66	NA	1.42
Mrps18b	0.82	2.3	2.49	0.13	2.22

Mrps18c	0.17	1.38	0.06	NA	2.16
Mrps2	0.12	1.99	0.66	0.83	0
Mrps21	0	NA	3.27	0	0
Mrps22	0.35	0	3.59	0	0
Mrps23	0.25	NA	1.1	0.82	0
Mrps24	0.38	NA	1.54	1.79	0
Mrps25	0.16	NA	1.16	1.51	0
Mrps26	0.26	NA	1.12	0.24	0
Mrps27	0	2.26	1.28	1.21	0
Mrps28	0.06	0	1.44	0.53	3.57
Mrps30	0	1.29	1.64	0.89	4.14
Mrps31	0.72	1.23	1.4	0.72	0
Mrps33	0.34	0.6	1.88	0.92	0
Mrps34	0.05	1.54	5.8	3.48	0
Mrps35	0.57	0.82	1.14	1.46	0
Mrps36	0.7	NA	2.17	NA	0
Mrps5	0.19	NA	0.84	1.16	0.52
Mrps6	0.78	NA	0.87	0	3.79
Mrps7	0.07	1.46	0	0.32	3.07
Mrps9	0.29	NA	0.64	2.1	3.96
Mrrf	0	NA	0.99	0.04	5.6
Mrs2	NA	0.8	1.63	0.36	0
Mrto4	NA	2.35	0.66	2.12	2.29
Mrvi1	0.3	NA	2.03	0.65	0
Ms4a1	NA	0	1.15	0	0
Ms4a10	2.18	NA	NA	0.91	NA
Ms4a13	NA	NA	NA	1.09	NA
Ms4a15	NA	NA	0.37	0.7	NA
Ms4a2	1.36	0.85	0.27	2.29	1.23
Ms4a3	1.06	0.5	0.6	4.98	0
Ms4a4a	NA	NA	NA	0.06	NA
Ms4a5	0.84	NA	NA	0	NA
Ms4a7	0	NA	2.39	NA	0
Msantd1	NA	NA	0.58	0.62	NA
Msantd2	NA	0	0.69	0.47	NA
Msantd3	NA	NA	2.32	0.22	NA
Msantd4	NA	NA	1.23	0.99	NA
Msc	0	0	1.62	1.5	0
Msgn1	0.73	NA	NA	2.17	NA
Msh2	0	0	1.43	0	1.51
Msh3	0	1.18	0.66	0.6	0.91

Msh4	3.88	1.1	2.06	NA	0
Msh5	0.61	NA	NA	NA	NA
Msh6	0.11	0	3.49	NA	0.18
Msi1	NA	1.05	0.6	2.45	0.53
Msi2	NA	NA	0.66	1.3	0
Msl1	NA	1.74	1.5	1.13	0
Msl2	NA	2.02	2.74	2.76	3.2
Msl3	NA	3.43	2	0.01	1.07
Msln	4.02	1.43	0.26	2.39	0.68
Msmb	0	2.09	1.28	0.66	0
Msmp	NA	NA	NA	3.68	NA
Msn	0.21	2.63	0.4	0.06	0.38
Msr1	1.12	0.29	1.28	0.87	1.55
Msra	0	0.04	0.65	5.18	0
Msrb1	NA	0.02	2.74	2.28	NA
Msrb2	NA	4.85	1.29	3.95	0
Msrb3	NA	NA	1.51	0.84	1.4
Mss51	NA	NA	3.31	0.43	NA
Mst1	0.23	1.43	5.89	0.11	0
Mst1r	0	3.26	0.52	1.49	1.52
Mstn	NA	NA	NA	4.55	NA
Msto1	NA	NA	1.91	NA	3.42
Msx1	0.34	1.93	0.29	0	0
Msx2	0.11	0.49	1.18	2.44	1.66
Mt3	1.95	1.31	1.47	1.45	0
Mt4	0.35	0.58	0.53	2.72	2.72
Mta1	3.35	0	0.03	2.07	0
Mta2	0	0.39	2.06	0.59	2.15
Mta3	0.89	NA	0.99	0.47	0.31
Mtac2d1	0	NA	NA	NA	NA
Mtap	0	0.78	0.64	NA	0
Mtbp	0.06	NA	2.69	0.59	4.56
Mtch1	1.44	0	2.87	0.86	0
Mtch2	0	0.86	0.68	0.27	0
Mtcp1	0	NA	0.94	NA	0.26
Mtdh	NA	0.39	1.21	0.81	2.66
Mterf	0.4	1.72	0.72	0.75	0
Mterfd1	NA	0	0.73	0	3.35
Mterfd2	NA	1.18	0.25	NA	4.59
Mterfd3	NA	NA	1.45	1.43	0
Mtf1	1.33	0	0.89	0	0

Mtf2	0.12	0.88	1.03	1.01	1.1
Mtfmt	NA	NA	1.72	1.68	2.24
Mtfp1	NA	NA	0.39	2.23	NA
Mtfr1	NA	0.84	0.48	0.98	0.73
Mtfr1l	NA	NA	1.46	0.98	NA
Mtfr2	NA	NA	2.76	0.74	NA
Mtg1	NA	0.51	0.41	2.78	1.07
Mthfd1	0.2	0	1.19	0.92	0
Mthfd1l	NA	NA	1.61	0	0
Mthfd2	1.71	2.76	2.09	0.81	0.59
Mthfd2l	NA	2.89	0.64	0.61	0.61
Mthfr	4.44	0.31	0.61	0	0.16
Mthfs	5.56	0	2.65	NA	0.07
Mthfsd	NA	0	0.44	2.68	0
Mtif2	0.81	0.07	1.88	1.45	0
Mtif3	NA	NA	0.36	1.38	0
Mtl5	0	1.63	0.97	1.38	1.28
Mtm1	0.37	0.59	0.19	2.14	1.87
Mtmr1	1	0.81	0	0.72	1.56
Mtmr10	NA	2.62	0.62	2.01	0.35
Mtmr11	NA	1.34	1.46	0.18	1.58
Mtmr12	NA	1.51	1.27	0	4.32
Mtmr14	NA	0.57	6.28	0.29	0.41
Mtmr2	0	1.79	0.15	0.34	0
Mtmr3	0.26	1.38	1.32	0	2.18
Mtmr4	0.49	1.22	5.26	0	0.27
Mtmr6	3.97	0.39	0.97	0.85	1.48
Mtmr7	1.74	0.78	1.77	0.75	0.18
Mtmr9	0.5	0.56	0.97	0	0
Mtnr1a	0	2.19	0.07	0	3.46
Mtnr1b	2.16	NA	NA	0	NA
Mto1	2.42	0.13	1.2	0.58	1.72
Mtor	NA	0.45	1.2	0	0.7
Mtpap	NA	0	0.59	NA	0
Mtpn	0.51	NA	1.46	NA	1.31
Mtr	0	0	0	0	0
Mtrf1	2.92	NA	NA	1.01	NA
Mtrf1l	0.26	2.2	0.03	0	3.29
Mtrr	0.61	0.81	0.43	1.8	1.67
Mtss1	0.42	0.48	2.37	1.71	1
Mtss1l	NA	2.47	1.68	1.2	NA

Mttp	11.57	1.56	1.17	0	2.68
Mtus1	1.18	1.77	1.57	3.8	0
Mtus2	NA	0.44	0.13	1.72	NA
Mtx1	0.24	1.25	2.17	19.78	1.99
Mtx2	0	2.63	1.61	0.86	3.53
Mtx3	NA	NA	0.82	0.92	0
Muc1	0.78	0	6.33	0.31	0
Muc13	NA	3.64	0.99	0.42	0.74
Muc15	NA	NA	0.15	1.65	3.77
Muc16	NA	0.54	0.56	NA	6.38
Muc2	NA	2.1	0.53	0.85	2.71
Muc20	0.12	NA	0.43	1.51	1.46
Muc3	NA	NA	0.1	NA	NA
Muc4	0.56	0.9	0.81	1.6	2.02
Muc5ac	NA	1.56	0.55	NA	0
Muc5b	NA	2.13	0.74	1.11	0
Muc6	0.44	NA	NA	0.02	NA
Mucdhl	3.3	NA	NA	NA	NA
Mucl1	NA	NA	3.46	0	NA
Mudeng	NA	NA	NA	NA	0
Mul1	NA	0.08	0.73	0.08	0.7
Mum1	0.26	0.67	3.44	1.96	0
Mum1l1	1.49	NA	0.26	0.81	0.44
Murc	NA	NA	1.56	0.4	1
Mus81	0	0.71	1.72	0.4	0.55
Musk	0	0.49	1.08	2.11	2.65
Mustn1	0.39	1.51	1.18	3.06	0.91
Mut	1.45	1.79	0.99	0.04	0
Muted	0	NA	NA	NA	0
Mutyh	0	0	1.57	1.36	0
Mvb12a	NA	NA	0.15	0.94	NA
Mvb12b	NA	0.79	1.91	2.3	NA
Mvd	1.51	1.12	3.3	0.79	0
Mvk	0.09	1.91	3.06	0.64	0
Mvp	0.14	0.73	1.44	0.02	0
Mx1	0.26	3.08	1.62	0	0
Mx2	0.14	3.25	0	0.22	0
Mxd1	NA	0.83	1.91	2.07	0
Mxd3	0	0	2.41	0.29	4.42
Mxd4	0	0	0	0.41	0
Mxi1	0.31	1.85	2.01	1.2	0

Mxra7	NA	1.26	0.15	1.22	0.63
Mxra8	NA	0	0.83	0.96	1.39
Myadm	0.27	NA	0.84	0	0.04
Myadml2	NA	NA	1.52	1.8	2.25
Myb	0	1.07	0.28	2.85	0.57
Mybbp1a	0.03	2.11	0.73	NA	2.27
Mybl1	NA	1.39	0.92	0.36	2.35
Mybl2	1.01	0.69	1.39	0.61	0
Mybpc1	NA	0.63	0.5	1.13	2.43
Mybpc2	0	1.18	0.17	0.71	1.33
Mybpc3	0	0	0.9	1.24	0
Mybph	3.33	1.24	1.25	1.36	0
Mybphl	NA	NA	NA	0.74	NA
Myc	0	1.89	2.08	0	8.79
Mycbp	0.23	1.88	0.42	NA	0.9
Mycbp2	NA	0	0.71	1.32	1.67
Mycbpap	0	NA	1.5	1.69	0.3
Mycl1	NA	0	0.85	1.54	0
Mycn	NA	0.92	0.38	3.49	1.46
Myct1	1.28	2.55	0.34	0.19	1.26
Myd88	0.38	0.77	5.03	0	0
Myef2	0.52	1.21	2.32	0.29	1.53
Myeov2	NA	NA	0.2	0.69	0
Myf5	1.44	0	0.92	0.42	0
Myf6	2.47	0.04	5.46	0.95	4.51
Myh1	1.49	1.18	0.83	1.25	2.33
Myh10	0.38	0.61	1.49	0	4.13
Myh11	0.27	0.33	1.17	0.3	0.13
Myh13	NA	NA	NA	0.6	NA
Myh14	3.12	1.11	0.98	0.71	0
Myh15	NA	NA	NA	0	NA
Myh2	4.45	1.03	1	2.23	4.93
Myh3	1.34	3.36	4.1	1.71	0.53
Myh4	0.04	0.81	2.71	1.04	0
Myh6	0.92	1.57	0	NA	0
Myh7	1.19	1.17	0.23	0.56	0
Myh7b	NA	0.72	0.04	1.16	0
Myh8	0	1.26	1.08	2.53	2.65
Myh9	1.02	0	1.27	0	0
Myl1	0.59	1.1	1.01	0.77	1.72
Myl10	NA	1.76	1.31	1.3	3.23

Myl12a	NA	0	2.44	NA	NA
Myl12b	NA	0.92	0.9	0.22	0.13
Myl2	0.73	2.3	1.52	1.71	0
Myl3	1.29	1.48	2.21	1.24	0
Myl4	0	0.48	0.18	1.35	0
Myl6	1.2	2.77	3.19	0.31	2.98
Myl6b	NA	3.52	0	0	3.33
Myl7	1	0.84	0	7.89	4.4
Myl9	1.12	1.51	6.76	0	0
Mylc2pl	0.89	NA	NA	NA	NA
Mylip	1.07	3.03	2.05	2.59	0
Mylk	0.26	0.35	0.63	0.59	0
Mylk2	0	NA	4.42	0	0
Mylk3	NA	1.73	1.33	0.78	2
Mylk4	NA	NA	0.56	0	NA
Mylpf	2.85	0.75	2.14	0.72	0
Mynn	0	2.1	0.67	0	0.95
Myo10	0.17	0.48	0.29	0	1.67
Myo15b	NA	0.59	2.67	0.85	0.96
Myo16	NA	1.47	0	3.11	2.58
Myo18a	0.75	0	0.7	1.33	NA
Myo18b	0.42	NA	2.45	1.28	NA
Myo19	NA	1.01	1.82	2.57	0
Myo1a	0.8	0.01	4.92	0	4.62
Myo1b	0.62	0.99	1.54	1.11	1.14
Myo1c	2.71	0.58	0.8	1.86	0.54
Myo1d	0	1.1	2.05	0.25	0.07
Myo1e	0.44	0	2.9	0	0.79
Myo1f	0.82	2.64	0.98	1.41	0
Myo1g	0.77	NA	1.85	1.05	0
Myo1h	NA	NA	2.58	NA	NA
Myo3a	0	1.37	1.87	6.55	7.11
Myo3b	0.01	NA	1.45	0.66	NA
Myo5a	0.63	1.08	0.48	0	0
Myo5b	4.14	NA	0.17	1.15	0.24
Myo5c	0.6	0	2.14	0.37	0.49
Myo6	0	0.69	0.47	NA	0
Myo7a	0.49	1	1.23	0.7	0
Myo7b	2.25	1	0.98	0.53	2.18
Myo9a	1.47	0	0.88	0.01	1.85
Myo9b	0	0.57	1.76	1.14	0

Myoc	0.55	1.31	0.23	2.13	2.05
Myocd	0.05	NA	1.02	0.64	4.54
Myod1	0	0	0.44	1.84	3.56
Myof	NA	0	0.95	1.92	0.05
Myog	5.56	2.96	0	1.09	5.27
Myohd1	0.72	NA	NA	NA	NA
Myom1	0	0.54	2.39	1.39	0
Myom2	0	1.22	0.49	0.7	1.31
Myom3	0.38	NA	1.09	1.22	9.16
Myot	NA	0.2	2.64	1.01	0
Myoz1	1.08	1.12	0.34	0.7	2.46
Myoz2	NA	0.47	0.04	1.22	0
Myoz3	2.21	0.25	1.6	0.66	0
Mypn	NA	NA	0.92	2.54	1.75
Mypop	NA	NA	0.95	1.99	0
Myrf	NA	1.78	2.84	0	NA
Myrfl	NA	NA	1.72	0	NA
Myrip	0.58	2.21	0.49	0	0
Mysm1	NA	NA	0.92	0.13	0.13
Myst1	0.98	NA	NA	NA	0
Myst2	1.9	NA	NA	NA	1.28
Myst3	1.59	NA	NA	NA	0
Myst4	0.38	NA	NA	NA	0
Myt1	2.04	1.54	0.69	0	0
Myt1l	0.49	0.84	0.39	1.7	8.07
Myzap	NA	NA	NA	0.62	NA
Mzb1	NA	0.59	2.06	1.29	NA
Mzf1	NA	0	1.36	1.44	3.21
Mzt1	NA	NA	0.95	NA	NA
N4bp1	NA	0.62	1.73	0.91	0
N4bp2	NA	NA	0	0	1.7
N4bp2l1	NA	0.41	2.45	0	1.66
N4bp2l2	NA	0.39	0.39	0	0
N4bp3	NA	1.5	0.99	2.03	NA
N6amt1	NA	0.3	0.92	0.08	0.83
N6amt2	NA	NA	1.28	0.65	0.16
Naa10	NA	1.46	1.11	NA	NA
Naa11	NA	1.16	1.6	0.99	NA
Naa15	NA	1.34	0.92	0.77	NA
Naa16	NA	0.01	1.62	1.02	NA
Naa20	NA	NA	1.63	0	NA

Naa25	NA	NA	0.76	0.67	NA
Naa30	NA	NA	1.28	0.94	NA
Naa35	NA	0	1.6	0	NA
Naa38	NA	0	0.51	0.76	NA
Naa40	NA	0	0.4	0	NA
Naa50	NA	1.93	1.3	0.67	NA
Naa60	NA	1.67	1.08	0.81	NA
Naaa	NA	2.03	0.56	1.31	0
Naalad2	0.64	0	0	0.59	0
Naaladl1	NA	1.91	1.41	2.4	1.48
Naaladl2	NA	NA	3.18	0.38	NA
Nab1	0	0.23	3.3	1.72	0
Nab2	0	1.21	0.12	0.02	0.57
Nabp1	NA	0	2.01	0.33	NA
Nabp2	NA	1.21	2.95	0	NA
Naca	0.36	2.08	0.87	0	0
Nacad	NA	1.08	0	0.7	0
Nacc1	NA	NA	0.41	4.65	1.07
Nacc2	NA	1.15	0.78	1.94	0
Nadk	NA	3.01	3.11	2.54	0
Nadk2	NA	NA	1.26	3.56	NA
Nadsyn1	NA	6.08	0.92	2.37	1.5
Nae1	NA	0.2	1.7	0	NA
Naf1	NA	NA	2.9	3.29	1.76
Naga	0	1.13	1.64	0	0
Nagk	0	0.97	1.75	0.82	0.97
Naglu	0	0.07	0.66	0	2.26
Nagpa	0	0.63	0.14	0.44	0
Nags	0.52	NA	1.07	0.52	0
Naif1	NA	NA	0.88	0.64	0
Nalcn	NA	NA	1.79	0.31	NA
Nampt	NA	0.09	3.32	1.43	0
Nanog	0.34	1.2	1.6	NA	4.24
Nanos1	6.67	NA	0.42	1.19	1.62
Nanos2	0	NA	NA	0.89	NA
Nanos3	2.38	NA	NA	1.35	NA
Nanp	NA	NA	3.23	0.94	0
Nans	0.08	0	0.95	1.81	2.38
Nap1l1	0.41	0	0.69	0.93	0
Nap1l2	0.01	3.92	3.34	1.62	0
Nap1l3	1.61	1.65	0.94	1.07	0

Nap1l4	0.1	1.07	1.64	0.43	2
Nap1l5	0	NA	2.02	1.45	9.84
Napa	1.54	0.04	0.86	7.71	1.77
Napb	0.57	NA	0.81	0.46	1.91
Napepld	NA	NA	1.54	0.79	1.49
Napg	0.62	0.01	0.82	0	2.3
Naprt1	NA	NA	1.98	1.92	1.41
Napsa	0.55	NA	0.36	2.32	0.75
Narf	NA	2.17	0.12	1.19	5.39
Narfl	0	1.89	1.28	0.67	3.06
Narg1	0.12	NA	NA	NA	NA
Narg2	0.55	0	1.39	2.37	0
Nars	0	2.2	2.03	0.97	0
Nars2	NA	1.75	1.01	0.84	1.65
Nasp	1.66	0.96	0.77	0.81	0
Nat1	0	1.11	0.38	0.12	0.78
Nat10	NA	0.6	1.23	2.98	2.41
Nat14	NA	NA	1.9	0.52	0.78
Nat15	NA	NA	NA	NA	0
Nat2	0	2.63	0.52	1.28	3.42
Nat5	0.34	NA	NA	NA	NA
Nat6	0	0	0.87	0.45	1.11
Nat8	NA	0.27	0	1.34	0
Nat8l	NA	NA	3.3	0.31	1.22
Nat9	NA	1.45	1.46	0.3	0
Nav1	0.87	NA	1.79	0	0.76
Nav2	13.06	0.64	1.16	2.39	1.33
Nav3	NA	0.63	0.72	2.51	0
Nbas	NA	1.83	0.17	0	0.9
Nbea	0.34	0	0.67	1.32	0.41
Nbeal1	NA	NA	0.71	0.58	NA
Nbeal2	NA	1.75	1.68	1.23	0
Nbl1	0.7	0.26	1.66	2.08	0
Nbn	0.54	0	2.91	1.56	0
Nbr1	0.69	0.94	0.86	3.19	6.98
Ncald	0	0	0.13	1.19	0
Ncam1	0	0.49	0.66	1.03	0
Ncam2	0.62	2.57	1.09	0.46	0.84
Ncan	NA	0.69	0.58	2.22	3.85
Ncapd2	NA	0	5.17	0	0
Ncapd3	NA	0	1.64	0.11	1.77

Ncapg	NA	0.15	1.29	NA	1.51
Ncapg2	NA	0.17	2.91	1.24	2.51
Ncaph	NA	0.2	3.55	0.33	0
Ncaph2	NA	0.17	1.5	1.22	1.15
Ncbp1	NA	1.12	1.58	0.09	0.84
Ncbp2	0.11	0.86	2.35	0.83	0
Nccrp1	NA	NA	NA	0.86	NA
Ncdn	0.82	0.85	2.44	1.65	1.42
Nceh1	NA	NA	0.88	0.99	0
Ncf1	4.16	NA	NA	NA	NA
Ncf2	0.14	0.92	1.29	0	1.52
Ncf4	0.96	0.16	2.76	0.66	0
Nck1	0.68	0.75	2.07	0.78	4.05
Nck2	NA	0	0.66	0.38	0
Nckap1	0.21	1.98	1.92	2.5	0.26
Nckap1l	NA	1.72	0.07	1.8	3.89
Nckap5	NA	NA	0	0.69	0
Nckap5l	NA	NA	0.23	0	1.49
Nckipsd	0.75	0.53	2.39	0	2.23
Ncl	0	1.75	0.96	1.93	2.06
Ncln	NA	1.67	1.78	0	0.8
Ncmap	NA	NA	NA	0	NA
Ncoa1	0.33	0.25	0.92	2.03	0.48
Ncoa2	0.89	0.71	0.68	3	0
Ncoa3	0.18	1	2.03	3.16	0.9
Ncoa4	0.84	NA	NA	2.88	NA
Ncoa5	0.57	NA	0.24	0.18	0
Ncoa6	0.3	0.94	0.63	1.7	3.42
Ncoa7	NA	NA	0.26	2.69	1.25
Ncor1	0.83	0	0.52	1.74	0.57
Ncor2	0	0.08	1.05	0.79	1.18
Ncr1	3.32	1.36	1.8	5.85	0
Ncrna00081	NA	NA	NA	NA	0.56
Ncrna00085	NA	NA	NA	NA	0
Ncrna00086	NA	NA	NA	NA	1.71
Ncs1	NA	1.12	0.29	3.1	NA
Ncstn	0.72	0.76	1.04	0	0
Ndc80	NA	1.65	1.76	0	3.8
Nde1	0.52	1.45	1.28	0.74	0.76
Ndel1	0.07	0.99	0.75	0	0
Ndfip1	0	0.06	2.49	2.17	NA

Ndfip2	0.71	NA	1.34	0.81	0.04
Ndn	1.44	0.18	2.28	2.6	1.22
Ndnf	NA	0.81	0.74	0.44	NA
Ndnl2	0.78	NA	0.28	1.94	0
Ndor1	7.52	2.2	0	3.71	2.27
Ndp	NA	1.25	0.38	1.04	0
Ndrg1	0.99	0.9	0.14	4.24	1.71
Ndrg2	3.4	0	0	2.41	0.19
Ndrg3	0.1	0	1.83	1.37	2.64
Ndrg4	0.54	2.07	2.29	0	1.33
Ndst1	0.01	1.07	0.88	1.52	2.07
Ndst2	0.8	0	0.6	5.85	0
Ndst3	0.55	1.52	0.57	1.97	0
Ndst4	0.79	0.89	0.08	13.9	2.22
Ndufa1	0	1.69	1.56	NA	1.7
Ndufa10	NA	0	0.84	1.43	NA
Ndufa11	0.2	NA	0.41	0.92	0
Ndufa12	NA	NA	2.17	5.89	0
Ndufa13	NA	1.08	0.68	NA	0
Ndufa2	0.59	1.3	1.33	0.12	3.06
Ndufa3	0.86	1.25	0	0	0
Ndufa4	0.11	2.87	3.03	0.12	0
Ndufa4l2	NA	0.36	1.15	0.41	0
Ndufa5	0.26	1.58	1.51	0.72	0
Ndufa6	0.03	5.01	2.99	1.7	0
Ndufa7	0.4	0.86	0	0	0
Ndufa8	0.5	0	1.14	2.85	0
Ndufa9	0.35	0.11	0.46	0	0
Ndufab1	0	1.71	1.07	1.07	0
Ndufaf1	0	5.33	0	1.25	0
Ndufaf2	NA	NA	2.46	0.95	3.42
Ndufaf3	NA	2.6	1.52	0.74	3.07
Ndufaf4	NA	1.15	2.65	3.01	0
Ndufaf5	NA	1.98	2.65	1.34	NA
Ndufaf6	NA	NA	2.47	2.05	NA
Ndufaf7	NA	0.33	2.6	2.34	NA
Ndufb10	0.14	NA	1.99	0	0
Ndufb11	NA	1.04	1.18	0	0
Ndufb2	0	0	1.14	0	0
Ndufb3	1.16	0	1.16	0	0
Ndufb4	NA	3.1	2.39	0	0

Ndufb5	0.26	0	1.29	0	0
Ndufb6	NA	0	0.44	0.95	0.96
Ndufb7	0	0	0.06	0	1.75
Ndufb8	0.34	1.47	1.12	0	0
Ndufb9	0	NA	2.41	0	0
Ndufc1	0.78	0.19	1.37	10.75	0.18
Ndufc2	2.1	0	1.91	1.64	0.69
Ndufs1	7.45	1.37	0.35	0	2.33
Ndufs2	0.07	1.8	1.85	1.98	0
Ndufs3	0.14	0	1.51	1.93	0
Ndufs4	0.15	0	1.33	1.09	1.67
Ndufs5	0.44	NA	NA	NA	0
Ndufs6	NA	0	1.33	0.03	0
Ndufs7	0.85	1.25	4.5	3.81	0.71
Ndufs8	0.32	0.98	0.24	0.1	0
Ndufv1	0.27	1.31	0.41	0.65	0
Ndufv2	0	0	2.32	1.98	3
Ndufv3	NA	NA	0.56	4.28	0
Neat1	NA	0.05	3.19	NA	0.82
Neb	0.96	0.68	2.24	0	0
Nebl	0.73	0.68	0.1	1.25	1.02
Necab1	NA	NA	1.68	0.46	0.94
Necab2	NA	1.06	2.19	1.53	7.6
Necab3	NA	0.17	0.03	1.96	0
Necap1	NA	0.82	1.76	0.39	2.1
Necap2	NA	0.53	1.63	0.07	0.88
Nedd1	0.83	NA	0.65	0.57	2.37
Nedd4	1.27	1.99	0.48	2.02	0.22
Nedd4l	0.43	0.75	1.47	1.27	0
Nedd8	0	1.43	2.17	0	0
Nedd9	0.46	1.68	1.77	1.18	0
Nefh	0	0.08	2.91	3.07	0
Nefl	0.65	0.03	0.64	NA	0
Nefm	NA	1.45	0.35	0.64	0
Negr1	0.74	NA	0	0.96	0
Neil1	1.03	1.76	1.82	0.13	2.49
Neil2	3.39	NA	NA	0.58	NA
Neil3	NA	0	1.71	0.73	0
Nek1	0.47	0.93	0.19	1.77	0.53
Nek10	NA	NA	NA	0	NA
Nek11	1.2	2.5	0.44	0.96	0.41

Nek2	0	0.31	1.55	0.27	2.06
Nek3	0	0.4	1.03	0.62	2.68
Nek4	0	0.15	0.93	0.08	0
Nek5	NA	NA	NA	0	NA
Nek6	0.49	NA	0.22	4.06	0.11
Nek7	1.2	1.54	1.2	0.74	3.66
Nek8	0	NA	2	0.9	NA
Nek9	6.9	1.67	0.22	0	0
Nelf	0.91	NA	NA	NA	0
Nelfa	NA	0	2.29	NA	NA
Nelfb	NA	0	2.74	0	NA
Nelfcd	NA	0	1.15	0.66	NA
Nelfe	NA	2.31	3.53	0.98	NA
Nell1	NA	1.26	0.91	1.04	1.44
Nell2	0	0	1.15	0	0
Nemf	NA	0.56	1.22	0	NA
Nenf	NA	1.14	3.12	0	0
Neo1	0.73	1.91	1.15	1.88	0
Nes	0	1.24	5.2	2.24	0
Net1	0	0	1.05	0.68	3.55
Neto1	0.32	NA	1.15	2.4	NA
Neto2	0	0	0	0.06	0.09
Neu1	0	0	1.37	1.51	0.01
Neu2	2.45	0.01	1.95	0.07	2.72
Neu3	0.02	0.2	1.14	1.48	2.13
Neu4	1.19	NA	NA	0.51	NA
Neurl	2.45	NA	NA	NA	NA
Neurl1b	NA	NA	3.13	NA	0
Neurl2	NA	NA	3.6	2.11	0
Neurl3	NA	NA	0.12	1.23	0.63
Neurl4	NA	NA	2.27	0.74	0.01
Neurod1	0.9	0.74	1.34	0.03	1.45
Neurod2	1.83	1.33	0.98	3.08	0
Neurod4	0	0.45	0.05	0.24	2.57
Neurod6	0.09	1.39	1.69	1.24	0
Neurog1	1.31	1.74	0.72	1.21	0
Neurog2	0	2.2	2.05	0.9	0
Neurog3	0.62	0.85	0.78	2.7	1.68
Nexn	0.62	NA	0	0.24	1.09
Nf1	0.44	0	1.29	1.77	0
Nf2	0.36	0.63	1.03	2.02	0

Nfam1	NA	NA	1.9	2.79	0
Nfasc	0.25	0	0.94	0.52	1.53
Nfat5	0.24	2.03	0	2.36	0.24
Nfatc1	0.51	0.05	1.5	0.67	0
Nfatc2	0.74	NA	0.71	0.96	0.43
Nfatc2ip	0.38	0	0.53	1.43	0
Nfatc3	0	0	1.05	1.02	0
Nfatc4	1.28	1.2	0	0.14	0
Nfe2	2.71	2.46	4.43	1.91	0
Nfe2l1	0.93	1.03	3.04	0	3.26
Nfe2l2	0.87	0.31	1.25	0.67	0
Nfe2l3	3.27	1.3	0.11	1.23	1.24
Nfia	0	NA	1.1	0.25	0.67
Nfib	0.5	0.67	0.01	0.38	1.26
Nfic	0.41	0.02	1.94	1.54	0
Nfil3	0.06	0.13	0.67	0.4	NA
Nfix	0.39	1.51	1.15	0.47	1.16
Nfkb1	0	1.41	1.46	8.66	0
Nfkb2	0.1	0.63	0.9	1.71	0
Nfkbia	0	0.56	1.26	1.61	0
Nfkbib	0.93	0.78	0.4	1.19	0
Nfkbid	NA	NA	0.53	4.44	0
Nfkbie	0.32	1.59	2.09	0.97	0
Nfkbil1	0.93	0.64	1.81	1.96	0
Nfkbil2	NA	NA	NA	NA	4.23
Nfkbiz	0.25	NA	0.69	1.25	0
Nfrkb	0.76	0	0.74	1.86	1.28
Nfs1	0.13	0	0.81	0	0
Nfu1	NA	0.03	0.75	4.2	2.24
Nfx1	0.46	0	0.58	2.77	0
Nfxl1	NA	NA	3.6	0.24	1.61
Nfya	1.46	0.59	0.77	0	0
Nfyb	0.57	0.78	1.84	0.13	2.78
Nfyc	0	1.24	0.17	0.67	1.39
Ngb	3.18	1.37	0.5	0.48	0.63
Ngdn	NA	1.21	0.92	1.8	1.38
Ngef	1.93	NA	1.33	3.35	3.54
Ngf	NA	1.73	0.46	0	1.6
Ngfb	0	NA	NA	NA	NA
Ngfr	0.09	2.21	0.77	1.93	0
Ngfrap1	0.88	1.32	1.93	0.26	1.86

Ngly1	0	2.15	2.62	0.13	4.16
Ngrn	NA	2.24	1.7	1.26	0
Nhedc2	NA	NA	NA	NA	1.63
Nhej1	NA	0	0.7	0	0.05
Nhlh1	1.37	0	0.64	0	0
Nhlh2	2.32	0.55	1.3	2.81	0
Nhlrc1	2.01	NA	NA	0.58	NA
Nhlrc2	0.38	0	0.95	0.92	0.86
Nhlrc3	NA	NA	1	0.58	0.05
Nhlrc4	NA	NA	NA	1.75	NA
Nhp2	NA	2.68	1.15	0.75	2.37
Nhp2l1	NA	2.16	0.54	1.52	0
Nhs	NA	NA	NA	2.04	NA
Nhsl1	NA	NA	0.49	1.54	0
Nhsl2	NA	NA	2.4	NA	NA
Nicn1	0	NA	2.22	0.84	0
Nid1	0.4	0.06	1.57	1.25	0
Nid2	0.57	0	6.36	NA	0.18
Nif3l1	0	1.75	1.97	NA	3.46
Nim1	NA	NA	NA	0.34	NA
Nin	1	0.3	2.56	0	0
Ninj1	0	1.01	1.09	1.55	0
Ninj2	0	0.55	1.85	0.05	NA
Ninl	NA	0	3.47	0.61	1.01
Nip7	NA	1.39	1.88	1.65	2.61
Nipa1	0	NA	2.83	NA	0
Nipa2	NA	1.6	4.21	0.86	0
Nipal1	NA	NA	0.17	0.13	0
Nipal2	NA	0.94	0.1	1.02	0
Nipal3	NA	0	2.23	0	0
Nipal4	NA	NA	1.78	0.35	1.05
Nipbl	NA	0.63	0.76	1.23	0
Nipsnap1	1.25	0	3.23	0.66	1.07
Nipsnap3a	NA	NA	1.36	2.72	0
Nipsnap3b	0.61	1.82	1.9	0.59	3.26
Nisch	0.04	0.6	3.63	1.66	2.27
Nit1	0.46	2.49	1.54	0.14	0.57
Nit2	0.15	0	1.52	1.19	8.12
Nkain1	NA	0	0.75	3.26	1.65
Nkain2	NA	NA	0	1.48	0.22
Nkain3	NA	NA	NA	1.98	NA

Nkain4	NA	NA	3.03	1.25	0
Nkap	NA	NA	0	0.95	1.81
Nkapl	NA	NA	0.22	1.16	2.01
Nkd1	0.44	NA	0.06	2.11	NA
Nkd2	0.8	NA	0.72	0.58	0
Nkg7	0	1.1	1.1	3.37	2.81
Nkiras1	0	NA	1.46	0	0
Nkiras2	0.89	1.72	1.03	NA	0.53
Nkpd1	0.23	NA	0.92	1.65	NA
Nkrf	NA	0.94	3.91	2.38	0
Nktr	0.29	1.17	0.42	0	2.04
Nkx1-2	1.52	NA	NA	0.79	NA
Nkx2-1	NA	0.64	1.05	2.82	0
Nkx2-2	0	2.8	2.81	0.65	2.83
Nkx2-3	0.08	NA	0.99	0.93	NA
Nkx2-4	NA	NA	NA	0.44	NA
Nkx2-5	4.84	1.79	0.16	1.06	0
Nkx2-6	2.16	NA	NA	0.38	NA
Nkx3-1	3.49	1.77	3.2	1.05	1.03
Nkx3-2	NA	1.49	0.26	1.56	0
Nkx6-1	1.15	2.53	2.41	1.89	NA
Nkx6-2	0.67	NA	0.11	NA	0.01
Nkx6-3	NA	NA	1.65	2.48	NA
Nle1	NA	0.96	0	3.42	0
Nlgn1	0.08	1.12	2.1	0.87	0.13
Nlgn2	0.35	NA	1.49	1.14	4.77
Nlgn3	0	1.22	1.18	1.12	NA
Nlk	0	1.13	2.15	4.02	0.85
Nln	0	NA	0.88	0	0
Nlrc3	NA	NA	1.57	1.15	2.73
Nlrc4	NA	NA	NA	1.9	NA
Nlrc5	NA	NA	0	0.62	1.83
Nlrp10	NA	NA	0	2.86	NA
Nlrp12	NA	NA	1.5	NA	0
Nlrp14	NA	NA	2.88	2.11	NA
Nlrp2	NA	NA	NA	0.86	NA
Nlrp3	NA	0.55	0.76	2.79	0
Nlrp5	NA	NA	1.57	1.34	NA
Nlrp6	NA	NA	0.48	1.02	NA
Nlrx1	NA	0.33	2.61	0	0
Nmb	1.38	0.48	2.01	3.52	0

Nmbr	2.09	2.9	0.11	0.91	2.72
Nmd3	NA	0.64	0.87	0.94	1.33
Nme1	0.29	0.27	0.25	NA	NA
Nme2	0.53	NA	NA	NA	NA
Nme3	1.1	2.63	1.51	2.75	0
Nme4	0.62	1.3	2.52	0	2.1
Nme5	0	1.87	7.72	0	0
Nme6	0	1.67	2.17	1.53	1.42
Nme7	0.47	0.46	1.33	0.8	0.67
Nme8	NA	2.74	0	7.64	NA
Nme9	NA	NA	NA	0	NA
Nmi	0.16	0	0.86	1.58	0.35
Nmnat1	NA	NA	1.19	0.59	2.57
Nmnat2	NA	0	1	5.13	7.79
Nmnat3	NA	NA	1.55	3.22	0
Nmral1	NA	NA	1.02	0.94	2.9
Nmrk1	NA	0.81	1.06	0	NA
Nmrk2	NA	1.9	0.5	0	NA
Nms	NA	NA	NA	2.13	NA
Nmt1	1.08	0	0.34	0.03	0
Nmt2	0	0	0.5	0.88	1.43
Nmu	1.25	1.71	0.78	1.42	3.43
Nmur1	NA	0.98	2.94	1.2	3.49
Nmur2	0.7	NA	2.26	1.59	2.98
Nnat	0.21	0.7	0.38	1.53	0
Nnmt	0	1.04	0.47	1.88	1.45
Nnt	0.08	0	2.13	0	0
Noa1	NA	NA	2.63	2.08	NA
Nob1	NA	NA	1.25	1.64	4.3
Nobox	NA	NA	0	0.15	0.03
Noc2l	NA	1.4	0.28	2.34	0.6
Noc3l	NA	1.42	2.81	1.07	3.16
Noc4l	NA	1.54	2.06	2.5	1.01
Nod1	NA	2.21	3.6	1.58	4.16
Nod2	NA	NA	NA	4.76	NA
Nodal	0.14	1.35	0.15	1.88	2.03
Nog	2.85	NA	2.97	0.67	5.4
Nol1	0.92	NA	NA	NA	NA
Nol10	NA	1.49	0.49	1.12	0.87
Nol11	NA	1.61	0.66	0.62	2.54
Nol12	NA	0.07	3.19	1.76	1.61

Nol3	0.21	2.54	1.14	NA	0
Nol4	1.25	1.36	0.13	1.92	1.65
Nol5a	0.27	NA	NA	NA	NA
Nol6	0	1.12	0.75	1.81	0.98
Nol7	0.42	0.76	3.11	0.83	1.56
Nol8	0	0	0.82	1.67	2.27
Nol9	NA	0	0.72	3.76	0
Nola1	0.23	NA	NA	NA	NA
Nola2	0.3	NA	NA	NA	NA
Nola3	0	NA	NA	NA	NA
Nolc1	0.54	0.72	1.98	1.39	1.89
Nom1	NA	NA	NA	1.44	NA
Nono	0.77	0	0	0.48	0
Nop10	NA	0	2.2	0.21	2.5
Nop14	NA	2.7	0.54	1.36	0
Nop16	NA	0.97	1.34	1.75	0
Nop2	NA	2.16	0.6	1.13	1.91
Nop56	NA	0.85	0.52	NA	1.29
Nop58	NA	NA	2.02	0.99	2.58
Nop9	NA	NA	0	0	NA
Nope	0.53	NA	NA	NA	NA
Nos1	3.58	0.73	0.57	1.66	1.12
Nos1ap	NA	0	0.91	0.03	0
Nos2	NA	1.67	0.39	1.75	0
Nos3	0.65	1.6	0.01	1.6	0
Nosip	0.71	0	0.69	0	0
Nostrin	NA	NA	0.96	1.37	3.03
Notch1	0.93	2.15	1.15	0	1.42
Notch2	0	1	1.33	0	0
Notch3	1.17	1.25	0	2.5	1.21
Notch4	0.41	0.16	0.98	NA	1.34
Notum	NA	NA	3.15	0.64	0
Nov	0.81	1.71	1.22	4.33	0.72
Nova1	0.22	0.64	0.6	3.91	2.04
Nova2	NA	0.48	0.67	0.05	0
Nox1	0	NA	NA	1.81	NA
Nox3	0.52	NA	NA	2.4	NA
Nox4	0.11	2.07	1.06	0.75	1.16
Noxa1	0	NA	NA	NA	NA
Noxo1	1.78	NA	2.66	2.04	4.01
Noxred1	NA	NA	0	0.17	NA

Npas1	1.61	3.03	3.75	0.79	0
Npas2	0.55	0.61	3	1.61	0
Npas3	0.49	0.93	0	0.62	1.13
Npas4	NA	NA	1.46	0.78	0.07
Npat	0.19	0	1.67	0	0
Npb	0.89	NA	3.56	0.88	7.53
Npbwr1	NA	NA	NA	0.54	NA
Npc1	0.07	0	0.93	0	0
Npc1l1	1.29	0.89	0	0.95	1.94
Npc2	0.57	0.06	0.79	0.02	0
Npdc1	1.85	1.33	2.08	1.14	5.69
Npepl1	0.16	2.13	0.67	0.57	0
Npepps	0	0.19	1.49	0	0.89
Npff	0.77	4.31	1.77	0.4	2.41
Npffr1	NA	NA	NA	1.24	NA
Npffr2	NA	NA	1.63	1.3	4.74
Nphp1	0.52	1.18	2.75	0.94	1.07
Nphp3	0.79	NA	1.5	NA	0
Nphp4	1.33	0.25	2.23	0.8	0.92
Nphs1	3.31	3.64	1.4	1.2	7.58
Nphs2	0.37	0.87	0.02	1.41	1.48
Npl	1.82	1.33	2.14	0.14	1.96
Nploc4	NA	2.33	3.18	0	1.61
Npm1	NA	1.74	3.42	0	3.4
Npm2	1.57	NA	NA	2.57	NA
Npm3	1.97	NA	NA	NA	NA
Npnt	0.92	NA	0.37	0	1.05
Nppa	NA	0.73	1.17	2.48	0
Nppb	4.32	0.03	5.08	2.13	0
Nppc	2.78	3.27	0.79	1.56	3.6
Npr1	0.58	2.28	1.44	1.06	3.81
Npr2	0.1	0	1.38	1.42	1.91
Npr3	1.15	0.74	0.42	0.46	0
Nprl2	NA	0.09	0.15	3.35	NA
Nprl3	NA	0	0.29	0	NA
Npsr1	NA	NA	NA	1.28	NA
Nptn	NA	0.19	1.79	0.93	0
Nptx1	0.33	0.02	3.18	2.3	0
Nptx2	2.52	0.67	0.87	2.06	5.42
Nptxr	0	NA	NA	NA	NA
Npvf	NA	1.98	2.09	1.57	0.56

Npw	NA	NA	NA	2.23	NA
Npy	0.69	1.02	1.79	0.77	0
Npy1r	0.4	0.37	0.81	1.02	2.27
Npy2r	NA	1.44	1.54	0.36	0
Npy4r	NA	1.61	0	0.58	NA
Npy5r	1.43	1.44	0.48	1.52	1.72
Npy6r	NA	2.2	1.62	0	2.25
Nqo1	0.29	1.6	0.41	2.67	0
Nqo2	1.95	4.62	3.54	0	1.4
Nr0b1	0.24	0.8	0	0.7	0
Nr0b2	0.51	2	1.12	0.97	1.89
Nr1d1	2.01	0	1.16	0.64	NA
Nr1d2	0.04	0	1.45	1	0
Nr1h2	0.28	1.77	0.02	2	0
Nr1h3	0.12	0.05	1.8	1.37	0
Nr1h4	0.08	2.46	0.48	0	2.19
Nr1i2	0.49	1.52	0.41	0.45	2.29
Nr1i3	6.97	2.29	0.23	3.48	1.72
Nr2c1	0	0	0.55	2.92	0.03
Nr2c2	2.33	1.18	1.07	3.82	0.13
Nr2c2ap	NA	NA	0	0.47	1.18
Nr2e1	0.43	2.02	0	1.69	0
Nr2e3	0.57	1.57	0.59	2.4	3.08
Nr2f1	0	0	1.73	2.42	NA
Nr2f2	2.09	0.47	0.6	1.6	0
Nr2f6	0.13	1.8	1.27	1.6	1.14
Nr3c1	0.63	0.24	0	1.3	2.83
Nr3c2	0.43	0	0.51	0.59	0
Nr4a1	0	0.99	1.88	1.24	1.09
Nr4a2	1	0.09	1.25	0.06	0.74
Nr4a3	0	0.64	0.47	0.59	0
Nr5a1	0.81	0.74	0.1	1.53	3.06
Nr5a2	1.01	0.96	0.34	0.77	2.24
Nr6a1	0.58	1.37	0.84	0	1.75
Nrap	0.31	1.26	1.42	NA	0
Nrarp	NA	NA	0.27	2.88	0
Nras	0.45	2.46	3.08	NA	1.19
Nrbf2	0	1.29	1.33	0.85	0
Nrbp1	NA	1.59	0.64	0	2.69
Nrbp2	NA	NA	0.88	0.17	0
Nrcam	NA	0.86	0.6	0	1.33

Nrd1	0.14	1.78	2.17	2.85	0
Nrde2	NA	2.24	0.64	0	NA
Nrep	NA	0	0.69	1.2	NA
Nrf1	0.82	0	0.55	0.85	0.7
Nrg1	0	0.65	1.22	1.64	5.09
Nrg2	2.01	1.07	0.44	0.74	1.18
Nrg3	1.59	NA	0.85	2.47	3.97
Nrg4	2.2	NA	1.23	3.65	0.05
Nrgn	0	0	0.72	0.79	0.91
Nrip1	0	1.25	1.36	0	1.74
Nrip2	0.24	1.68	0.44	2.15	0
Nrip3	0.78	1.29	0.62	1.29	0
Nrk	0.99	NA	0.39	0.94	1.61
Nrl	0.52	0.96	0.95	NA	2.52
Nrm	0.06	NA	1.12	0.94	2.08
Nrn1	0.57	2.37	1	1.14	3.74
Nrn1l	NA	NA	NA	0.98	NA
Nrp1	NA	0.74	0.87	0.93	2.26
Nrp2	0.49	1.05	0.86	2.27	0
Nrsn1	NA	NA	2.46	0.25	1.87
Nrsn2	NA	0.89	0.9	NA	0
Nrtn	1.48	1.87	2.04	1.26	0
Nrxn1	0.21	0.32	0.56	0	1.22
Nrxn2	1.06	1.18	0.31	0.44	0.87
Nrxn3	0.11	0.37	0.45	1.09	0
Nsa2	NA	0	1.77	0	NA
Nsbp1	1.13	NA	NA	NA	NA
Nsd1	0.29	0.83	0.61	1.99	0.07
Nsdhl	0.03	1.04	1.25	0.51	0.6
Nsf	1.52	0.23	1.08	0.85	2.05
Nsfl1c	0.51	0.71	2.22	0	0.55
Nsg1	NA	0	2.37	0.56	NA
Nsl1	NA	0	0.61	0.86	0.49
Nsmaf	1.17	0.99	2.56	1.01	0
Nsmce1	NA	NA	1.24	1.31	3.13
Nsmce2	NA	NA	0.53	0.86	2.06
Nsmce4a	NA	0.28	0.74	1.21	0
Nsmf	NA	0	2.31	0.84	NA
Nsun2	1.54	NA	1.64	1.4	3.17
Nsun3	2.7	0	0.98	NA	0
Nsun4	NA	NA	0	0.78	0.61

Nsun5	NA	0.32	0	2.51	2.68
Nsun6	NA	2.32	0.6	0.74	0
Nsun7	NA	2.72	1.43	1.65	0
Nt5c	0.19	1.64	0.63	0.75	1.11
Nt5c1a	NA	NA	NA	0.9	NA
Nt5c1b	0	NA	0.72	3.92	2.23
Nt5c2	0.69	0.32	1.65	1.4	0
Nt5c3	0.25	NA	NA	NA	1.64
Nt5c3b	NA	NA	7.82	0	NA
Nt5c3l	NA	NA	NA	NA	1.41
Nt5dc1	NA	NA	2.53	1.77	3.23
Nt5dc2	NA	0.74	3.71	NA	0
Nt5dc3	NA	0.9	0.51	4.47	3.69
Nt5e	1.62	0	0.37	0.89	2.77
Nt5m	0.62	0.98	0.16	0.55	0
Ntan1	0	2.36	1.26	0	0
Ntf3	2.54	0.75	0.63	1.41	2.95
Ntf5	0.09	NA	NA	NA	NA
Nthl1	1.38	1.78	0.76	0.26	0
Ntm	NA	0.38	1.79	5.61	1.69
Ntmt1	NA	NA	0.71	NA	NA
Ntn1	0	1.29	0.6	6.15	0
Ntn3	NA	0	8.35	1.03	7.67
Ntn4	1.48	NA	1.62	0.87	0
Ntn5	NA	NA	2.44	0.33	NA
Ntng1	0.87	0.76	0.05	1.85	0
Ntng2	0	NA	0	0	0
Ntpcr	NA	NA	1.25	0	NA
Ntrk1	0	1.03	2.15	NA	13.75
Ntrk2	1.06	0.59	0.55	1.24	0
Ntrk3	0.04	0.9	0.03	0	1.64
Nts	0.63	2.66	2.51	0	2.71
Ntsr1	13.04	3.98	0.73	0.13	8.29
Ntsr2	0	1.07	3.52	0	17.04
Nuak1	NA	2.43	2.83	0	3.24
Nuak2	NA	1.17	0.13	0.25	NA
Nub1	NA	NA	1.12	1.77	0.1
Nubp1	0.63	1.52	0.07	0.47	0
Nubp2	0	1.59	0.43	1.04	0.64
Nubpl	NA	2.86	0	0.27	2.97
Nucb1	0.21	2.62	1.57	0	1.92

Nucb2	1.07	0.98	1.19	0.41	2.68
Nucks1	NA	0.03	0.78	0.85	0
Nudc	0.14	1.34	1	2	1.39
Nudcd1	NA	NA	0	0.51	1.43
Nudcd2	NA	NA	0.23	0.68	0
Nudcd3	NA	0	0.62	0	0.11
Nudt1	0.1	1.04	2.14	0.06	0
Nudt10	0.91	NA	1.04	1.46	0
Nudt11	0	0	0	2.57	0.96
Nudt12	0	NA	2.41	0.91	1.37
Nudt13	2.33	0.4	0.89	0.36	0.98
Nudt14	0	NA	3.05	1	0.53
Nudt15	0.72	1.55	0.64	0.58	0
Nudt16	NA	NA	1.28	3.53	0.97
Nudt16l1	NA	NA	1.89	0	0
Nudt17	NA	NA	0.51	0.73	2.08
Nudt18	NA	2.65	1.3	0.6	0.99
Nudt19	NA	NA	2.12	NA	0
Nudt2	0.99	0.15	4.29	0	0
Nudt21	NA	1.09	0.97	1.13	0
Nudt22	NA	NA	2.03	4.69	0
Nudt3	0.86	2.38	1.63	NA	NA
Nudt4	0.11	0.23	1.8	1.71	4.84
Nudt5	0.09	NA	1.21	3.79	0
Nudt6	0	1.22	0.5	0.8	0.97
Nudt7	NA	1.42	1.03	0.58	1.25
Nudt8	0.2	NA	NA	0.69	NA
Nudt9	0.33	0.15	0.24	1.05	1.96
Nuf2	NA	NA	2.4	0.49	1.27
Nufip1	0	1.75	1.52	0.71	1
Nufip2	NA	NA	2.27	1.96	0.88
Nuggc	NA	NA	NA	3.3	NA
Numa1	0.17	0	1.91	0	1.78
Numb	0.78	1.77	0.54	3.84	0
Numbl	0.57	NA	2.06	1.81	0.8
Nup107	0.65	0	1.23	0.68	0
Nup133	0.41	0	0.87	0	0
Nup153	1.15	0	0.71	1.21	1.88
Nup155	0.48	0	1.16	0	0
Nup160	0.7	1.4	0.64	6.13	0
Nup188	2.84	0.84	0.65	2.47	0.19

Nup205	0.75	0.58	1.98	0	1.47
Nup210	1.14	0	0.44	2.54	3.22
Nup210l	NA	NA	0.42	0	0
Nup214	NA	1.18	0.38	1.05	0.09
Nup35	0.69	NA	3.25	2.46	3.16
Nup37	0	0.12	1.24	1.83	0
Nup43	0.59	1.54	1.01	1.9	0
Nup50	0.3	0.49	1.67	0.98	1.03
Nup54	0.75	0.46	1.44	0.03	0.04
Nup62	0.87	1.36	1.45	NA	0
Nup62cl	NA	NA	NA	9.1	NA
Nup85	NA	0.45	1.27	1.37	0
Nup88	0	2.21	1.07	0	0
Nup93	0.04	0	0.08	0.26	1.76
Nup98	0.59	1.18	0.55	4.09	0
Nupl1	0.5	1.41	0.32	NA	0.03
Nupl2	0	0.58	0.14	0.43	0.94
Nupr1	0.56	0.51	0	0.87	3.22
Nupr1l	NA	NA	NA	2.6	NA
Nus1	NA	1.08	2.36	1.24	0
Nusap1	0	1.48	0.97	0.99	0.57
Nut	0	NA	NA	NA	NA
Nutf2	0.25	1.24	1.31	2.05	0
Nutm1	NA	NA	0	1.17	NA
Nvl	0.76	2	1.32	0.84	1.12
Nwd1	NA	NA	NA	0.25	NA
Nxf1	0.6	2.23	2.51	3.94	0.65
Nxf2	0.77	2.24	1.44	NA	0
Nxf3	NA	1.83	0.15	0.72	2.47
Nxn	0.51	2.16	1.68	0.38	0
Nxnl1	NA	NA	0	0.45	NA
Nxnl2	NA	NA	1.55	1.6	NA
Nxpe2	NA	NA	NA	0	NA
Nxpe3	NA	1.43	2.09	1.27	NA
Nxpe4	NA	1.17	2.28	1.61	NA
Nxph1	1.65	NA	0	3.5	1.1
Nxph2	3.88	NA	0.61	2.81	0
Nxph3	0.56	0.51	1.46	0.79	3.02
Nxph4	0	1.68	1.39	0.93	6.36
Nxt1	0	3.36	1.52	2.18	0
Nxt2	1.66	0	1.01	0.76	0.26

Nyap1	NA	NA	0.25	1.24	NA
Nyap2	NA	NA	NA	1.61	NA
Nynrin	NA	1.22	0.27	1.29	NA
Nyx	0.83	3.61	0.94	1.2	0
Oaf	NA	NA	2.33	1.09	0.86
Oard1	NA	1.38	0.67	2.35	NA
Oas2	0.71	3.54	1.95	1.12	0.1
Oas3	0.24	1.31	3.31	0	0
Oat	0	1.46	1.09	1.01	0
Oaz1	1.81	3.35	0.51	NA	4.54
Oaz2	0	0	3.59	1.13	0.97
Oaz3	5.94	0	0.99	0	0
Obfc1	0	0.79	1.06	0.4	3.62
Obfc2a	NA	NA	NA	NA	0
Obfc2b	NA	NA	NA	NA	1.87
Obp2a	NA	0.88	0.98	0.62	NA
Obp2b	NA	NA	NA	0.86	NA
Obscn	0.39	1.18	2.67	1.24	0
Obsl1	NA	0.68	0.1	0.14	0
Oc90	NA	NA	NA	0.31	NA
Oca2	NA	1.35	2.5	0	0
Ocel1	NA	1.11	1.95	0.29	0
Ociad1	NA	NA	2.16	0	2.56
Ociad2	NA	NA	0	1.08	0
Ocln	1.62	NA	NA	0.28	1.52
Ocm	0.95	NA	NA	1.32	NA
Ocrl	NA	0.51	0.32	0	1.57
Ocstamp	NA	NA	NA	0	NA
Odam	NA	0.22	0	0	1.91
Odc1	1.44	0	4.26	1.24	2.13
Odf1	3.95	0.69	1.56	1.38	1.74
Odf2	0.46	0	0.81	0	1.55
Odf2l	NA	NA	0.17	0.85	1.07
Odf3	0	NA	0.79	3.15	0
Odf3b	NA	NA	0.39	0.58	0.8
Odf3l1	NA	NA	NA	1.41	NA
Odf3l2	NA	NA	NA	0	NA
Odf4	2.03	NA	0.69	0.77	NA
Odz1	0.59	NA	NA	NA	3.27
Odz2	NA	NA	NA	NA	0.55
Odz3	NA	NA	NA	NA	0.55

Odz4	NA	NA	NA	NA	1.31
Ofcc1	0	NA	NA	0.11	NA
Ofd1	0	3.11	0.55	0.49	3.89
Ogdh	0	0.11	0.03	2.92	1.1
Ogdhl	NA	NA	NA	0	NA
Ogfod1	NA	0	0.55	1.27	0
Ogfod2	NA	2.16	2.16	0.91	4
Ogfod3	NA	1.48	2.21	NA	NA
Ogfr	0.32	1.29	0.1	1.83	0
Ogfrl1	0	0.39	3.25	1.14	2.81
Ogg1	0.89	1.17	0.58	1.33	0
Ogn	1.93	1.7	0.66	2.6	0
Ogt	1.02	0.59	1.11	0	0.98
Oip5	NA	1.2	2.14	NA	2.88
Oit3	1.02	NA	0.48	0	2.31
Ola1	NA	1.04	0.1	0.02	0
Olah	NA	2.33	0.2	1.29	0
Olfm1	1.14	2.16	2.11	0	3.92
Olfm2	NA	NA	0.14	0.58	0
Olfm3	1.29	NA	0.72	5.3	NA
Olfm4	NA	0.98	0.73	1.04	0
Olfml1	0	3.55	0.87	0	0
Olfml2a	0.63	2.17	1.7	0.85	2.98
Olfml2b	0	0	2.14	1.23	0
Olfml3	0.43	0.98	2.47	0.21	0
Olig1	2.54	NA	4.44	0.91	0.35
Olig2	0.7	1.04	1.23	2.11	0.31
Olig3	0.95	NA	0.91	0.61	NA
Olr1	1.71	1.19	1.85	1.51	0
Oma1	NA	NA	1.87	0.78	0
Omd	1.76	0.06	1.07	0.51	2.39
Omg	1.33	3.98	0.68	0.15	0
Omp	0.35	1.74	0.71	1.11	3.57
Onecut1	0.54	2.02	0.26	1.21	0
Onecut2	0.65	1.32	0.54	2.19	1.9
Onecut3	NA	NA	3.74	NA	3.15
Ooep	NA	NA	0.58	NA	1.39
Opa1	0.06	0.32	0.86	0.59	NA
Opa3	NA	1.2	1.62	0.86	1.09
Opalin	NA	NA	0	0.17	0
Opcml	NA	0.49	0	1.28	2.47

Ophn1	1.02	0.65	2.88	0.6	0.56
Oplah	1.03	2.89	4.42	NA	0
Opn1sw	3.33	0.7	1.28	2.77	1.61
Opn3	0.35	2.19	0.58	0.13	3.19
Opn4	0	NA	1.64	0.42	0
Opn5	0	NA	NA	0	NA
Oprd1	0.45	0.17	0	0.98	3.28
Oprk1	0	1.87	0.07	10.73	3.7
Oprl1	NA	0.14	0.97	1.28	1.77
Oprm1	0.89	1.2	0	1.66	0
Oprs1	1.07	NA	NA	NA	NA
Optc	1.68	NA	0.4	1.8	1.34
Optn	1.04	0.82	0.72	1.25	3.91
Orai1	NA	NA	1	1.78	0
Orai2	NA	1.48	2.05	1.71	3.53
Orai3	NA	1.96	1.64	2.09	0
Oraov1	0	NA	1.42	0.76	0
Orc1	NA	1.38	2.82	0.45	NA
Orc1l	3.35	NA	NA	NA	2.05
Orc2	NA	0.42	0.54	1.49	NA
Orc2l	3.81	NA	NA	NA	0.2
Orc3	NA	0	0.68	0.56	NA
Orc3l	0	NA	NA	NA	0
Orc4	NA	1.65	1.67	0.85	NA
Orc4l	0.15	NA	NA	NA	0
Orc5	NA	2.12	1.35	0.57	NA
Orc5l	0.26	NA	NA	NA	2.96
Orc6	NA	1.55	0.67	0.5	NA
Orc6l	0.1	NA	NA	NA	0.64
Orm1	0.21	1.14	2.21	0.91	0
Orm2	1.26	0.42	1.23	1.58	NA
Ormdl1	NA	NA	0.67	0.57	2.95
Ormdl2	0	0	0.91	NA	0
Ormdl3	0.03	NA	0.5	2.37	3.09
Os9	NA	0.23	0.12	0	1.61
Osbp	0	0	3.08	0	0
Osbp2	1.59	0	1.36	2.7	0
Osbpl10	2.84	0.74	0.51	3.31	NA
Osbpl11	0	0.53	2.11	2.38	0.53
Osbpl1a	1.05	2.09	2.04	2.23	2.16
Osbpl2	0.09	0.57	2.88	0.07	2.04

Osbpl3	0.55	0	0.8	0.29	1.89
Osbpl5	0.97	NA	2.37	1.8	1.15
Osbpl6	0.63	NA	0.51	1.77	2
Osbpl7	1	0	1.4	0.93	0
Osbpl8	0	3.56	2.02	1.37	1.21
Osbpl9	0.28	0.88	1.21	0.61	0
Oscar	0.23	NA	0	0.54	NA
Oscp1	NA	NA	3.09	0.04	NA
Osgep	0.2	0	0.26	0.84	0
Osgepl1	0	0	1.01	1.62	2.1
Osgin1	NA	0	2.04	2.26	4.65
Osgin2	NA	0.87	1.53	0.51	2.33
Osm	0.36	0.14	3.24	0	0
Osmr	0.38	0.44	0.42	1.49	1.43
Osr1	0.2	NA	2.9	2.4	0
Osr2	0.81	1.95	0.83	0.69	0
Ost4	NA	NA	0.55	NA	NA
Ostc	NA	NA	0.66	1.06	0
Ostf1	0.06	0	1.98	1.21	0.99
Ostm1	0.6	2.34	2.49	2.4	0
Ostn	0.22	NA	NA	0	NA
Otc	1.94	0.47	0	2	3.04
Otoa	0	NA	NA	0	NA
Otof	0.53	1.56	0.8	1.44	2.3
Otog	0.2	NA	NA	NA	NA
Otogl	NA	NA	NA	0.87	NA
Otol1	NA	NA	NA	1.16	NA
Otop1	1.82	NA	NA	1.48	NA
Otop2	3.38	NA	2.85	1.33	7.09
Otop3	0.51	NA	NA	1.73	NA
Otor	5.48	0.24	4.01	0	1.6
Otos	1.44	NA	0	2.02	3.88
Otp	0.64	NA	1.15	0.91	0
Otub1	1.88	1.71	0.11	0.63	0
Otub2	0.26	1.89	2.13	0	0
Otud1	NA	NA	3.27	1.15	0.17
Otud3	NA	0	6.37	1.2	8.08
Otud4	NA	0.1	0.66	0.78	1.29
Otud5	NA	NA	0.02	0	0.01
Otud6a	NA	NA	NA	1.41	NA
Otud6b	NA	NA	0.99	0.29	1.95

Otud7a	NA	NA	0.83	1.4	NA
Otud7b	NA	2.51	0.11	2.8	0
Otx1	0.31	NA	1.71	3.27	5.62
Otx2	0.45	NA	0.87	0	0
Ovca2	1.72	1.25	2.02	2.35	NA
Ovch2	NA	NA	NA	2.14	NA
Ovgp1	0.26	0.78	1.08	0.16	0
Ovol1	2.38	1.02	0.88	1.77	2.02
Ovol2	NA	0.44	1.3	0.07	2.07
Oxa1l	0	0	2.33	0.61	0
Oxct1	2.22	0	1.34	0	0.96
Oxgr1	NA	NA	0.05	0.46	NA
Oxld1	NA	2.92	1.3	0.67	NA
Oxnad1	NA	NA	0.01	0.6	0
Oxr1	0.24	2.06	1.52	0.44	0
Oxsm	NA	0.82	0.74	0	0
Oxsr1	NA	1.05	1.45	0.13	0
Oxt	2.13	1.23	0.68	0.71	3.47
Oxtr	0.08	0.69	3.32	2.69	2.15
P2rx1	1.38	2.73	0.27	1.4	0.61
P2rx2	0.47	1.42	5.1	1.11	0
P2rx3	0.13	0.38	0.47	2.58	0
P2rx4	0	0.74	2.29	1.21	0.63
P2rx5	0.54	3.68	0	0.09	0
P2rx6	NA	1.71	0.57	0.39	0
P2rx7	1.99	0.83	1.78	3.01	4.09
P2rxl1	1.48	NA	NA	NA	NA
P2ry1	0.14	2.3	0.06	4.42	1.22
P2ry10	0.21	0	0	1	2.67
P2ry12	3.13	NA	1.06	0.89	0
P2ry13	0.16	1.18	3.72	2.48	0
P2ry14	0.83	2.88	3.02	1.74	1.05
P2ry2	0.4	1.54	2.49	3.26	0
P2ry4	0.7	1.5	2.36	1.37	6.41
P2ry6	0.49	1.4	2.28	6.83	6.75
P4ha1	0.66	0.71	1.81	0.53	1.83
P4ha2	0.24	2.4	0.28	0.24	0.82
P4ha3	1.61	NA	4.27	0.68	0
P4hb	0.8	2.16	1.53	1.34	0
P4htm	NA	0	1.48	0	0
P518	3.52	NA	NA	NA	NA

Pa2g4	0.17	1.63	0	0.13	0
Pabpc1	0.74	0.76	2.12	2.07	0
Pabpc1l	NA	NA	0	0	2.22
Pabpc4	NA	0	1.09	1.64	0
Pabpc4l	NA	NA	0.37	NA	0
Pabpc5	0	NA	0.41	2.27	0
Pabpn1	0	0.13	0.7	0.05	0
Pacrg	1.45	0.74	1.77	0	0
Pacrgl	NA	NA	0.34	0	0
Pacs1	0	0.12	2.03	0.72	0.43
Pacs2	NA	0	1.18	0.51	0
Pacsin1	0.47	NA	0.46	0	0.5
Pacsin2	1.53	0.51	2.38	0.14	NA
Pacsin3	0.57	1.55	0.58	0	2.28
Padi1	0.36	0.07	0.38	0.1	0
Padi2	1.2	2.85	1.16	0.88	5.09
Padi3	0.67	0.59	0.91	1.22	1.61
Padi4	0.83	0	1.81	2.48	0
Padi6	0.1	NA	NA	0.43	NA
Paf1	NA	0.39	1.71	0.33	0
Pafah1b1	0.47	0.25	1.16	0	0.14
Pafah1b2	0.24	0.01	1.28	0.63	0
Pafah1b3	0	0.19	1.29	NA	0.24
Pafah2	0	0	1.5	0.88	0
Pag1	NA	NA	1.54	1.63	0.47
Pah	0	2.5	2.14	1.95	2.5
Paics	0.12	0	0.74	0	0
Paip1	2.08	0.46	0.95	0.41	0
Paip2	0	NA	2.71	0	0
Paip2b	NA	0.03	1.31	0.1	0
Pak1	0	1.44	0.65	1.53	0.76
Pak1ip1	0.84	1.27	2.52	2.8	0
Pak2	0	0.9	0.02	2.12	0.68
Pak3	1.15	0.51	0.55	0.74	3.24
Pak4	0.88	1.26	0.88	1.3	3.17
Pak6	NA	0.73	0.86	0.9	0
Pak7	0.49	0.57	0.06	1.67	1.56
Palb2	NA	0	2.67	0.92	3.47
Pald1	NA	NA	2.13	1.42	NA
Palld	NA	0.59	0.07	0.15	0
Palm	0.43	1.2	0.43	1.75	1.31

Palm2	1	NA	0.88	NA	NA
Palm3	NA	NA	NA	2.21	NA
Palmd	2.32	1.12	2.88	3.21	0
Pam	0	0	0.87	0	0
Pam16	NA	1.54	4.57	NA	NA
Pamci	0.92	NA	NA	NA	NA
Pamr1	NA	1.61	0.82	5.45	0
Pan2	NA	0	0.24	0	4.72
Pan3	NA	NA	2.73	1.3	0
Pank1	2.16	NA	1.13	0.9	0.73
Pank2	1.01	2.02	0	1.17	0
Pank3	0.45	0.87	1.86	0	1.47
Pank4	0.91	0	1.33	0	1.45
Panx1	0.63	2.15	1.43	0.66	1.93
Panx2	NA	NA	0	0.57	1.75
Panx3	0.16	NA	1.3	5.11	1.59
Paox	0.43	0.84	2.41	1.31	NA
Papd1	0	NA	NA	NA	NA
Papd4	1.94	NA	0.85	2.71	0
Papd5	NA	NA	1.25	0.46	0
Papd7	NA	0.75	0.89	0.39	NA
Papln	0.93	NA	0	0.01	0
Papola	0	0.78	1.6	2.06	0
Papolb	0.83	0.74	1.89	0.52	6.21
Papolg	0.71	1.76	1.95	0.69	0
Pappa	10.78	0.05	0.09	NA	1.48
Pappa2	0.29	0	3.41	1.18	0
Papss1	0	0.68	0.89	4.01	0.14
Papss2	1.17	0	1.42	5	2.57
Paqr3	0.42	2.98	2.54	1.93	5
Paqr4	0	0	3.04	NA	1.37
Paqr5	NA	0.38	0.84	1.24	1.26
Paqr6	0.02	0.6	0.89	1.01	4.27
Paqr7	NA	NA	1	2.71	1.16
Paqr8	NA	NA	0	1.68	2.94
Paqr9	0.56	NA	0.77	0	NA
Parc	3.48	NA	NA	NA	NA
Pard3	1.01	0.78	0.14	5.65	0
Pard3b	NA	NA	0.48	0.52	0
Pard6a	0.06	3.27	0	0.72	0
Pard6b	0.64	0.7	2.53	0.4	0

Pard6g	0.85	NA	1.01	0	1.53
Parg	0.04	0.02	1.68	NA	0
Park2	0	1.23	0.7	0	0
Park7	0.12	1.04	3.36	1.38	0
Parl	NA	0.08	1.48	0.49	0
Parm1	NA	0.06	0.99	2.01	NA
Parn	0	0	1.16	2.58	0
Parp1	NA	0	2.22	0	0
Parp10	NA	NA	0.66	0	NA
Parp11	NA	2.11	0.72	0.62	0.75
Parp12	NA	2.43	2.34	0	0
Parp14	0.43	NA	2.6	0	0
Parp16	0	0	2.48	3.84	0
Parp2	NA	0	1.09	0	0
Parp3	NA	1.3	1.56	1.94	0
Parp4	NA	0.89	1.02	1.58	1.7
Parp6	0.38	0.05	1.34	2.05	1.94
Parp8	0.2	0.9	0.63	0.98	1.05
Parp9	NA	NA	1.3	NA	0.34
Parpbp	NA	1.27	1.21	NA	NA
Pars2	NA	NA	0.93	1.72	1.22
Parva	0.88	0.71	1.01	1.54	1.68
Parvb	0.6	1.18	2.8	1.5	0
Parvg	0.28	NA	0.75	1.45	1.96
Pask	0	0	1.91	0	0
Pate2	NA	NA	NA	1.13	NA
Patl1	NA	NA	1.2	0.23	0
Patl2	NA	NA	1.81	4.08	0
Patz1	NA	0	1.77	1.39	0
Pawr	1.12	0.45	1.11	8	3.79
Pax1	2.38	0.73	0.99	1.17	1.62
Pax2	0	NA	NA	0.72	NA
Pax3	1	0.31	0.72	2.04	0
Pax4	0.4	1.81	0.65	1.67	4.56
Pax5	5.05	1.1	2.03	0	4.97
Pax6	0.06	0.59	0.49	0	1.14
Pax7	0	0	0.32	1.15	2.16
Pax8	0.77	0.83	1.07	1.36	1.96
Pax9	0.35	1.01	0.07	3.25	0
Paxbp1	NA	0.22	0.43	0	NA
Paxip1	5.96	0	0.83	1.2	2

Pb1	0.4	NA	NA	NA	NA
Pbdc1	NA	NA	0.18	1.75	NA
Pbef1	0.25	NA	NA	NA	NA
Pbk	0.48	0	3.02	1.32	1.13
Pbld	NA	NA	NA	NA	0
Pbrm1	NA	1.3	2.58	0	0
Pbx1	0.36	1.38	0.33	0	0
Pbx2	0.22	0.7	0.68	2.7	0.54
Pbx3	0	2.06	2.93	1.63	0
Pbx4	0.09	NA	0.51	1.24	5
Pbxip1	0.02	0	1.54	0	4.92
Pcaf	0.97	NA	NA	NA	NA
Pcbd1	NA	3.22	0	2.97	0.12
Pcbd2	NA	NA	1.16	2.4	0.01
Pcbp1	0.26	2.38	1.46	0.84	1.2
Pcbp2	0	0	1.17	0.02	0
Pcbp3	0	0.86	1.14	0	0
Pcbp4	1.32	0	2.58	1.68	0
Pcca	0	0.26	0.23	1.85	0.92
Pccb	0.31	0.09	1.27	0.72	1.66
Pcdh1	NA	1.03	1.31	1.43	4.43
Pcdh10	0.17	NA	0.82	3.83	0
Pcdh11x	0	1.49	0.92	NA	0
Pcdh12	2.46	1.09	0.31	0.66	1.78
Pcdh15	0.45	NA	0.43	0.56	NA
Pcdh17	NA	1.11	0	1.22	1.26
Pcdh18	1.24	NA	0.81	0.95	0
Pcdh19	NA	NA	0.51	0.59	2.13
Pcdh20	0	NA	0.2	1.8	1.51
Pcdh21	0	NA	NA	NA	NA
Pcdh7	0.38	0.56	2.54	0	0
Pcdh8	1.01	3.55	1.06	1.63	0
Pcdh9	0.46	0.17	0.67	0	0.11
Pcdha1	1.94	NA	NA	NA	NA
Pcdha10	5.38	3.84	1.56	NA	0
Pcdha11	4.14	NA	NA	NA	NA
Pcdha12	0.83	NA	NA	NA	NA
Pcdha2	0.45	NA	NA	NA	NA
Pcdha3	2.55	NA	NA	NA	NA
Pcdha5	0	NA	NA	NA	NA
Pcdha6	0.44	NA	NA	NA	NA

Pcdha7	0.44	NA	NA	NA	NA
Pcdha8	0	NA	NA	NA	NA
Pcdhac1	1.22	NA	1.05	NA	NA
Pcdhac2	1.36	NA	2.07	NA	NA
Pcdhb1	1.94	1.7	0.98	1.46	2.28
Pcdhb10	2.56	NA	1.45	2.65	0
Pcdhb11	1.32	0.07	0	0.91	0
Pcdhb12	1.8	1.11	2.54	3.23	1.95
Pcdhb13	0.35	0	2.9	2.27	4.56
Pcdhb14	1.5	NA	1.36	0.46	1.09
Pcdhb15	1.4	NA	0	4.07	2.12
Pcdhb16	0.29	NA	1.09	1.52	2.05
Pcdhb17	13.37	0.67	0.26	1.11	4.72
Pcdhb18	3.49	NA	2.12	0.35	2.92
Pcdhb2	0.44	NA	3.37	4.78	0
Pcdhb3	1.54	2.21	1.9	9.09	0
Pcdhb4	4.64	NA	0.58	3.69	0
Pcdhb5	0.94	NA	3.09	0	1.85
Pcdhb6	0	0	0.57	4.42	6.97
Pcdhb7	0	NA	1.83	4.72	3.27
Pcdhb8	0.73	0.9	2.29	0	0
Pcdhb9	1.72	NA	0.09	2.93	0
Pcdhga1	0.91	NA	NA	NA	NA
Pcdhga10	1.92	NA	NA	NA	NA
Pcdhga2	0.72	NA	NA	NA	NA
Pcdhga3	1.04	NA	NA	NA	NA
Pcdhga4	0.78	NA	NA	NA	NA
Pcdhga5	2.34	NA	NA	NA	NA
Pcdhga6	1.13	NA	NA	NA	NA
Pcdhga7	0.96	NA	NA	NA	NA
Pcdhga8	0	NA	NA	NA	NA
Pcdhga9	1.51	NA	NA	NA	NA
Pcdhgb1	3.06	NA	NA	NA	NA
Pcdhgb2	0.84	NA	NA	NA	NA
Pcdhgb4	0.1	NA	NA	NA	NA
Pcdhgb5	1.77	NA	NA	NA	NA
Pcdhgb6	0.72	NA	NA	NA	NA
Pcdhgb7	0	NA	NA	NA	NA
Pcdhgc3	0	NA	NA	NA	NA
Pcdhgc4	0	NA	3.41	NA	2.45
Pcdhgc5	0.47	NA	NA	NA	NA

Pced1a	NA	NA	0.73	2.12	NA
Pced1b	NA	NA	0	0	NA
Pcf11	NA	0.56	1.83	0	1.71
Pcgf1	NA	0.6	0.87	0.34	0
Pcgf2	NA	0.75	1.32	0.01	0
Pcgf3	NA	0.87	0.31	0.71	0.38
Pcgf5	NA	NA	1.63	1.72	1.93
Pcgf6	NA	NA	1.71	1.1	3.91
Pcid2	NA	2.57	0.17	0.32	0
Pcif1	NA	1.36	1.85	1.81	0.31
Pck1	0.49	2.03	1.65	0.72	1.25
Pck2	NA	1.03	3.12	0.6	0
Pclo	NA	0.81	0.49	1.26	2.33
Pcm1	0.64	0.01	1.41	0.16	1.26
Pcmt1	0	1.83	1.58	0.45	0.57
Pcmtd1	NA	NA	1.34	0.66	0
Pcmtd2	NA	0	1.73	2.58	2.46
Pcna	0	0.14	0.68	NA	0
Pcnp	0.69	1.91	3.87	1.65	0
Pcnt	NA	0	1.96	0	0.6
Pcnx	0.43	0	1.71	1.49	0
Pcnxl2	NA	0	0.41	0.01	0.66
Pcnxl3	0.75	NA	2.4	1.4	2.66
Pcnxl4	NA	0.17	0.78	2.98	NA
Pcolce	0	1.37	0.82	1.42	0.66
Pcolce2	0	0	1.3	2.05	2.08
Pcoln3	1.05	NA	NA	NA	NA
Pcp2	0.63	NA	1.46	NA	0.01
Pcp4	0.78	1.39	1.59	3.02	0
Pcp4l1	2.05	NA	0.84	0	1.2
Pcqap	1.58	NA	NA	NA	NA
Pcsk1	0.4	0.77	1.46	3.67	0.07
Pcsk1n	0.03	0.63	0.84	1.05	0
Pcsk2	0.66	0.83	0.59	1.31	0
Pcsk4	0	NA	2.46	1.05	0
Pcsk5	0.82	1.35	0.45	0	2.17
Pcsk6	NA	1.12	1.55	4.59	2.39
Pcsk7	0.74	0	1.79	1.56	3.36
Pcsk9	0	NA	1.04	1.53	4.12
Pctk1	1.51	NA	NA	NA	NA
Pctk2	7.76	NA	NA	NA	NA

Pctk3	0	NA	NA	NA	NA
Pctp	0	2.24	0.28	0.56	2.52
Pcyox1	0.37	0	0.4	0.07	0
Pcyox1l	NA	0	0.44	1.7	5.19
Pcyt1a	0.45	0	0.22	1.51	0
Pcyt1b	3.78	1.39	0.86	0.9	1.07
Pcyt2	NA	0.12	3.18	0	1.1
Pdap1	1.6	1.5	0.88	1.06	0.14
Pdc	0.01	1.55	3.12	0.66	1.41
Pdcd1	1.91	2.25	1.11	1.49	4.87
Pdcd10	0.49	0	2.4	0.57	0.51
Pdcd11	0.78	0.72	1.84	2.64	1.46
Pdcd1lg2	0	1.09	1.68	11.26	2.93
Pdcd2	0.26	0.19	1.03	0	0
Pdcd2l	NA	NA	1.15	0.34	0
Pdcd4	0.32	0	1.17	0.37	0
Pdcd5	0.05	0	3.33	0.79	0
Pdcd6	1.9	0.87	4.52	NA	0.83
Pdcd6ip	0.29	0	2.83	0.24	1.79
Pdcd7	0	NA	1.5	0	0.65
Pdcl	1.07	0.55	1.15	0.7	0
Pdcl2	0.58	NA	3.95	0.78	0
Pdcl3	0	NA	NA	0.53	NA
Pddc1	NA	NA	2.07	0	0
Pde10a	0.49	0.56	1.36	2.37	1.18
Pde11a	1.86	1.08	0.68	2.58	0
Pde12	NA	1.77	1.42	1.24	0.32
Pde1a	0.3	0	0.03	1.77	0
Pde1b	0.51	0	3.73	0.9	0
Pde1c	0.49	0.93	0.97	2.15	0
Pde2a	0.58	1.99	0.37	0	0.07
Pde3a	1.3	1.46	0.36	1.88	0
Pde3b	0	0	3.32	4.03	0.89
Pde4a	0.32	1.4	1.2	0.54	0
Pde4b	0	0.67	1.25	0.53	0
Pde4c	0.59	NA	NA	NA	NA
Pde4d	0.55	0.13	0.31	1.52	3.63
Pde4dip	NA	0	2.06	4.48	0
Pde5a	0.71	1.39	2.05	0.04	0
Pde6a	1.52	0.74	3.06	2.31	8.94
Pde6b	2.11	0.74	0.41	0.91	0

Pde6c	1.21	0.3	2.47	0.19	0
Pde6d	0.26	1.59	1.5	0.31	1.33
Pde6g	1.08	1.24	0.11	0.9	0
Pde6h	1.15	1.26	3.07	3.5	0
Pde7a	0.76	NA	1.87	0.02	0
Pde7b	1.18	0.79	0.61	3.35	0.82
Pde8a	2.05	1.66	0.74	1.92	1.59
Pde8b	3.28	1	0.92	2.63	1.69
Pde9a	0.44	0	0.68	0	1.73
Pdgfa	0.5	1.3	3.11	4.54	0
Pdgfb	0.27	0.78	0.77	0.7	2.57
Pdgfc	1.21	0.37	2.93	2.36	0
Pdgfd	0.21	0.15	0.68	2.41	1.62
Pdgfra	1.08	0.51	1.11	2.13	1.05
Pdgfrb	0.54	1.41	2.36	1.67	0.56
Pdgfrl	0.76	2.67	0.35	0.41	0
Pdha1	0.02	1.28	2.45	NA	1.69
Pdha2	0	1.67	0.48	0.06	0
Pdhb	0.44	0	1.65	0	NA
Pdhx	0	1.67	2	0.22	4.2
Pdia2	NA	0.4	0.02	1.01	5.72
Pdia3	NA	1.61	0.88	1.66	3.13
Pdia4	NA	1.66	3.17	NA	0
Pdia5	NA	0	1.47	0	2.41
Pdia6	NA	0.86	1.19	1.58	0
Pdik1l	NA	NA	1.19	3.7	0
Pdilt	NA	NA	1.42	3.68	NA
Pdk1	1.66	1.75	0.09	0.77	1.03
Pdk2	0	0.35	0.55	0.85	1.42
Pdk3	0.65	0.09	3.53	0	0
Pdk4	1.16	0.82	2.32	0.82	0
Pdlim1	0.47	0	1.1	0	NA
Pdlim2	1.16	3.76	2.55	0.7	0.45
Pdlim3	4.69	1.43	1.37	2.33	0
Pdlim4	0.55	1.07	1.75	0	3.9
Pdlim5	NA	0.24	0.64	4.08	0
Pdlim7	0.66	1.98	1.86	2.16	0
Pdp1	NA	1.4	1.68	0	0
Pdp2	NA	NA	3.46	2.58	0.66
Pdpk1	0.78	0.78	0.52	0.16	1.54
Pdpn	NA	0.79	1.42	6.05	0

Pdpr	NA	1.1	1.42	0.56	NA
Pdrg1	0.31	NA	0.98	1.78	0
Pds5a	NA	0	1.44	0	1.83
Pds5b	NA	2.05	0.69	0	0.03
Pdss1	NA	0	1.09	1.03	0
Pdss2	NA	0.44	0.36	1.87	0
Pdx1	NA	1.13	1.07	1.45	1.78
Pdxdc1	NA	0	0.03	0.1	0
Pdxk	0	0.21	1.02	0.03	0
Pdxp	0	NA	1.02	2.95	0.57
Pdyn	0.69	1.47	0.96	0.69	4.32
Pdzd11	NA	NA	0.32	1.68	0
Pdzd2	NA	0.82	0.7	0	0.71
Pdzd3	NA	1.5	0.65	1.55	0
Pdzd4	NA	NA	0.78	0	0.7
Pdzd7	NA	NA	NA	1.08	NA
Pdzd8	NA	0.14	1.11	0	1.2
Pdzd9	NA	NA	0.7	0.1	NA
Pdzk1	0	2.09	0.8	NA	2.11
Pdzk1ip1	NA	0.37	2.55	0.77	0
Pdzrn3	2.9	0	1.55	0.84	0
Pdzrn4	NA	1.13	0	1.14	0
Pea15	0.52	NA	NA	NA	NA
Pear1	NA	NA	0.03	2.03	1.42
Pebp1	NA	1.04	0.36	0.68	0
Pebp4	NA	NA	0.8	2.02	0
Pecam1	0	0	0	1.59	4.4
Peci	0	NA	NA	NA	5.51
Pecr	0	2.39	2.14	0.87	3.14
Pef1	NA	3.9	1.49	1.78	0
Peg10	0.25	1.15	0.28	0.62	0
Peg3	0	0.48	0.28	NA	1.86
Peli1	0.33	1.34	2.85	0.52	0
Peli2	0	1.27	0.12	2.21	2.7
Peli3	NA	NA	NA	0.25	NA
Pelo	0.12	1.25	1.04	0	1.43
Pelp1	NA	0	2.96	1.99	1.71
Pemt	0.47	0	0	0	0
Penk	NA	1.62	0.48	1.97	0
Peo1	0	NA	NA	NA	NA
Pepd	NA	1.1	1.4	3.11	0

Per1	0.6	1.7	0.49	0.05	0.11
Per2	0.02	1	0.08	NA	0
Per3	0.03	0.28	0.64	NA	0
Perld1	0	NA	NA	NA	NA
Perp	2.71	0	1.31	2.64	0.48
Perq1	0	NA	NA	NA	NA
Pes1	0.48	1.87	2.2	0.91	0
Pet100	NA	NA	2.2	NA	NA
Pet112	NA	1.65	0.08	0.8	NA
Pet112l	0	NA	NA	NA	1.85
Pet117	NA	NA	NA	1.96	NA
Pex1	1.64	0.5	1.64	1.42	1.84
Pex10	NA	0.32	0.72	NA	0.1
Pex11a	0.49	0.97	0.37	1.58	0
Pex11b	0.2	0.21	1.12	2.8	2.91
Pex11g	NA	NA	4.1	4.6	NA
Pex12	3.16	1.34	2.43	1.71	0
Pex13	0.68	0	1.6	0	0
Pex14	0.81	0	1.23	1.21	0
Pex16	2.32	0.61	0.53	0	0.56
Pex19	0.44	1.14	0.41	0.8	0
Pex2	NA	0.02	1.59	0	NA
Pex26	NA	0.13	5.11	2.67	0.69
Pex3	0.87	0	0.03	0.62	1.18
Pex5	0	0	0.47	0.84	3.08
Pex5l	NA	1.07	0.57	3.06	0.88
Pex6	0.32	2.14	0.78	0.15	0.65
Pex7	0	2.29	1.85	1.52	2.08
Pf4	NA	0.88	2.66	2.86	0.31
Pfas	NA	0	0	2.14	0
Pfdn1	24.74	0.61	3.2	0.13	0
Pfdn2	1.46	0.45	8.21	0.81	0
Pfdn4	0.42	0.52	1.09	0.3	0
Pfdn5	1.06	1.54	1	2.66	1.88
Pfkfb1	2.9	1.4	2.95	1.67	1.83
Pfkfb2	0	1.55	0.42	0.44	3.23
Pfkfb3	0.15	0	1.7	0	0
Pfkfb4	NA	0.05	0.95	0.82	0
Pfkl	0.93	1.33	0.49	0.76	1.62
Pfkm	0	1.46	2.08	0	1.7
Pfkp	0	1.36	1.2	0.2	1.44

Pfn1	0	2.28	1.1	1	0
Pfn2	0.28	0	2.57	0.83	0.7
Pfn3	2.62	NA	NA	NA	NA
Pfn4	4.36	NA	0.16	0.53	0
Pftk1	0.46	NA	NA	NA	NA
Pga5	0	NA	NA	NA	NA
Pgam1	NA	1.28	2.15	NA	0
Pgam2	1.68	0.57	0.06	0	1.26
Pgam5	NA	NA	0.79	0.63	NA
Pgap1	NA	1.65	0.75	0	0.19
Pgap2	NA	2.23	0.32	0.66	0
Pgap3	NA	3.49	2.41	2.49	2.94
Pgbd1	NA	NA	3.08	2.72	15.41
Pgbd5	2.5	1.72	1.04	2.66	1.71
Pgc	0	2.01	1.4	1.4	0
Pgcp	3.19	NA	NA	NA	2.55
Pgd	0	0.36	2.17	0.21	0
Pgf	0.35	0.87	2.65	2.1	3.83
Pggt1b	2.69	0.63	1.59	0	0.55
Pgk1	0.94	0	1.92	0.05	0
Pgk2	2.78	0	2.19	2.42	0
Pgls	0	1.59	1.96	0.37	0
Pglyrp1	0.25	0.35	2.43	0.19	0
Pglyrp2	0	NA	1.7	1.31	1.6
Pglyrp3	2.44	NA	NA	0.92	NA
Pglyrp4	0.57	NA	NA	1.07	NA
Pgm1	0	1.03	0	1.99	0
Pgm2	0.51	NA	0.77	0.21	1.2
Pgm2l1	0.55	NA	3.43	0	0
Pgm3	0.2	1.73	2.64	0.19	0
Pgm5	1.87	1.59	2.56	1.79	NA
Pgp	NA	2	0.7	0.74	0.9
Pgpep1	0.7	1.86	2.32	0.69	0
Pgpep1l	NA	NA	NA	2.45	NA
Pgr	0.56	1.7	0.77	5.2	2.36
Pgrmc1	0.42	0.25	2.78	1.49	1.85
Pgrmc2	0.03	0	2.19	2.23	0
Pgs1	NA	0	1.87	2.42	0
Phactr1	NA	0.62	0.9	0.67	0
Phactr2	0.04	0.12	0.97	1.42	0
Phactr3	0.48	NA	4.06	1.96	0.48

Phactr4	0.48	0	0.86	0.43	0
Phax	NA	NA	1.69	2.51	0
Phb	0	1.11	1.32	0.71	0.61
Phb2	NA	1.85	1.82	0.78	0
Phc1	0.28	0	1.76	NA	0
Phc2	0.35	1.54	2.05	1.89	0
Phc3	0	0.83	0.26	0.6	0
Phca	0.06	NA	NA	NA	NA
Phex	1.09	0.85	0.66	0.37	4.93
Phf1	0	0	6.12	0.41	0
Phf10	0.1	3.38	1.86	0.18	0
Phf11	0.13	NA	NA	NA	NA
Phf12	0.77	NA	2.44	1.82	0
Phf13	2.54	NA	1.12	0.74	1.58
Phf14	NA	1.2	0.09	0.03	0.5
Phf15	2.38	0	0.29	1.67	1.67
Phf16	0	1.62	2.12	0.9	0
Phf17	0.47	0	1.17	6.68	0
Phf19	0.26	NA	0.94	4.83	0
Phf2	5.89	0	0.64	0.49	0.88
Phf20	NA	0.07	0.65	0	0
Phf20l1	NA	1.71	1.11	0.84	1.36
Phf21a	NA	1.96	1.16	1.13	0
Phf21b	NA	NA	1.89	1.42	NA
Phf23	NA	NA	1.21	1.3	1.21
Phf3	0	0.24	0.94	0	1.07
Phf5a	0.75	NA	0.76	1.15	0
Phf6	0.37	NA	1.26	0.87	1.09
Phf7	1.78	0.86	1.45	0.21	0
Phf8	NA	0.71	0.8	0	0
Phgdh	0	0.79	2.34	0	0.53
Phgdhl1	0.02	NA	NA	NA	NA
Phgr1	NA	NA	0	NA	NA
Phip	0.48	0.21	0.53	0	0.05
Phka1	0.6	1.78	2.22	0	1.78
Phka2	0.49	1.8	0.92	0.03	1.27
Phkb	0.37	0	2.44	3.73	0
Phkg1	9.37	0.98	0.33	0	2.31
Phkg2	0.36	0.05	0.03	NA	1.12
Phlda1	0.01	0.77	2.36	8.7	0
Phlda2	0	3.44	3.64	0	9.63

Phlda3	0.45	3.16	0.64	3.27	0
Phldb1	NA	0.3	0.4	0.97	3.37
Phldb2	1.5	NA	0.54	4.73	0.89
Phldb3	NA	NA	NA	0.53	NA
Phlpp1	NA	1.34	2	1.79	0
Phlpp2	NA	0	1.15	NA	1.78
Phospho1	0.3	NA	NA	1.4	2.63
Phospho2	NA	NA	1.4	1.18	0
Phox2a	0	1.07	0.33	NA	0
Phox2b	0	1.27	0.78	0.38	0
Phpt1	0.28	NA	2.58	0.7	0
Phrf1	NA	NA	0.52	0	1.45
Phtf1	0.93	5.01	0.69	0.61	2.52
Phtf2	0	1.4	1.19	0.95	2.12
Phyh	2.89	0	1.41	2.57	2.76
Phyhd1	NA	NA	2.06	2.28	2.16
Phyhip	0.41	2.08	2.05	7.11	0
Phyhipl	0.5	NA	0.8	NA	0
Pi15	0.29	1.57	2.19	1.31	1.75
Pi16	0.31	NA	1.31	1.76	0
Pi4k2a	NA	0.81	0.53	0	0.02
Pi4k2b	0.63	NA	1.16	0.7	1.64
Pi4ka	NA	0	0.77	0	0
Pi4kb	NA	0	0.42	0	0
Pianp	NA	NA	1.03	2.87	NA
Pias1	0.62	1.09	0.74	0.58	0.65
Pias2	NA	0.23	0.39	0.47	0.36
Pias3	0.52	0.91	0.94	1.47	3.73
Pias4	0.61	0.81	0.78	4.16	0.96
Pib5pa	1.76	NA	NA	NA	NA
Pibf1	NA	0.66	0.53	NA	0
Picalm	0.5	2.33	1.75	0.38	0
Pick1	NA	2.09	1.49	2.24	0.14
Pid1	NA	2	1.42	1.02	0.18
Piezo1	NA	3.06	3.44	0.8	NA
Piezo2	NA	0.86	0.53	0.75	NA
Pif1	NA	NA	1.05	2.19	0.39
Pifo	NA	NA	NA	0	NA
Piga	0.4	0.21	1.03	1.16	1.15
Pigb	0.84	0.73	0.5	NA	6.08
Pigc	1.9	0.54	1.4	0.9	0.69

Pigf	0	0	1.14	NA	0
Pigg	NA	0	1.41	0	0.78
Pigh	0	0.69	1.42	NA	0
Pigk	0.1	1.9	1.41	1.48	0
Pigl	0	0	1.33	0.87	0
Pigm	2.27	NA	0	0	0
Pign	0.03	0.7	1.17	0.02	0
Pigo	0.23	0	2.41	0.21	0.13
Pigp	NA	0.28	1.99	0.88	0
Pigq	0	1.24	1.95	1.41	1.45
Pigr	0.33	0.33	1.25	0.17	1.17
Pigs	0	NA	0.2	0	0
Pigt	0.67	1.19	1.14	0.98	0
Pigu	NA	NA	1.09	1.3	0
Pigv	NA	1.26	1.18	1.46	2.05
Pigw	0.71	NA	NA	1.47	NA
Pigx	NA	NA	0.87	0.39	0.75
Pigy	NA	NA	NA	NA	0.58
Pigz	NA	1.28	1.28	2.21	0.14
Pih1d1	NA	1.69	1.9	3.85	2.43
Pih1d2	NA	NA	0	0.62	0.09
Pih1d3	NA	NA	1.46	0.53	NA
Pik3ap1	0.25	NA	4.29	0.09	0.03
Pik3c2a	0.18	1.21	0.93	0.08	2.14
Pik3c2b	NA	NA	NA	1.27	NA
Pik3c2g	0.45	0.13	1.29	1.86	1.93
Pik3c3	0.72	4.33	1.26	0	0
Pik3ca	0.84	1.48	3.57	3.38	0
Pik3cb	0.84	1.81	3.05	0	4.95
Pik3cd	0.74	0.82	2.48	0.3	0.74
Pik3cg	0.18	0	1.04	0.65	0
Pik3ip1	NA	0	1.29	3.63	2.67
Pik3r1	0.28	0	3.97	0.91	0
Pik3r2	0	2.18	1.4	1.19	3.27
Pik3r3	1.09	0	0.1	0	1.74
Pik3r4	0	0.74	0.9	1.64	0.7
Pik3r5	NA	2.08	2.58	1.03	0
Pik3r6	NA	NA	3.2	2.02	NA
Pik4ca	1.31	NA	NA	NA	NA
Pik4cb	0.69	NA	NA	NA	NA
Pikfyve	NA	1.07	1.51	0	0

Pilra	0.59	4.47	3.24	0.77	0
Pilrb	0.62	NA	NA	NA	NA
Pim1	0.5	3.13	2.37	0	0
Pim2	0	0.05	2.57	0	0
Pim3	0.16	NA	1.55	1.05	2.57
Pin1	1.88	1.01	0.96	0	3.09
Pin4	0.22	0.08	1.17	0.65	0
Pink1	0	1.36	1.42	1.83	NA
Pinlyp	NA	0.6	1.81	0.86	NA
Pinx1	0.35	NA	4.76	1.86	NA
Pion	NA	NA	NA	NA	2.53
Pip	2.16	2.46	1.48	0.78	1.66
Pip4k2a	NA	0.45	1.07	0.4	0
Pip4k2b	NA	0	0.42	1.72	0
Pip4k2c	NA	0	1.19	1.37	0
Pip5k1a	0.01	0.07	0.8	0.81	0.66
Pip5k1b	1	1.27	0.62	0.78	0.11
Pip5k1c	0.48	1.23	0.79	0.37	0
Pip5k2a	0.84	NA	NA	NA	NA
Pip5k2b	0	NA	NA	NA	NA
Pip5k2c	0	NA	NA	NA	NA
Pip5k3	0	NA	NA	NA	NA
Pip5kl1	1.01	NA	0.75	1.66	0
Pipox	4.9	2.76	1.19	0.33	2.1
Pir	0	2.31	2.29	2.78	1.72
Pirt	NA	NA	0	4.83	NA
Pisd	NA	1	1.51	1.21	NA
Pithd1	NA	NA	0.85	1.96	NA
Pitpna	NA	1.17	0.58	0.02	1.24
Pitpnb	0.61	1.46	1.23	0.75	0
Pitpnc1	0.95	0	2.33	3.69	1.05
Pitpnm1	0.39	0.88	2.61	1.6	0.85
Pitpnm2	0	NA	0	1.98	0.75
Pitpnm3	NA	0.85	0.72	0.11	0
Pitrm1	0	0	0	0.18	0
Pitx1	0	0.69	0.48	2.26	0
Pitx2	0.4	1.3	1.99	0	1.11
Pitx3	2.32	1.55	1.26	1.18	0
Piwil1	7.46	4.46	0.75	1.55	0
Piwil2	0.32	1.02	0	1.42	0
Piwil4	NA	NA	2.17	1.45	0

Pja1	0.66	0	2.53	0.32	2.33
Pja2	0.33	0.02	1.98	2.84	3
Pkd1	1.6	1.16	2.07	0.91	0.82
Pkd1l1	4.42	NA	NA	0.23	NA
Pkd1l2	0.72	NA	3.1	3.01	3.53
Pkd1l3	0.37	NA	NA	1.68	NA
Pkd2	1.5	2.17	1.36	2.34	0.3
Pkd2l1	NA	0	0	0.21	0.86
Pkd2l2	0.33	1.39	0.47	NA	0
Pkdcc	NA	NA	0.81	0.25	NA
Pkdrej	0	1.41	0.3	1.33	1.61
Pkhd1	0.43	NA	2.14	3.1	0
Pkhd1l1	1.45	NA	1.09	0	NA
Pkia	0.45	1.48	0.45	0.89	1.33
Pkib	0.67	NA	1.49	1.13	1.04
Pkig	0.77	0.12	0	0.8	0
Pklr	2.71	1.22	1	1.67	2.21
Pkm	NA	2.3	0.39	0.01	NA
Pkm2	0.99	NA	NA	NA	0
Pkmyt1	NA	2.8	1.72	2.84	4.46
Pkn1	0.29	1.08	3.4	0	2.62
Pkn2	0	1.38	1.34	0.02	0
Pkn3	0	NA	1.09	0.61	0.25
Pknox1	0.15	1.17	2.02	0	0
Pknox2	0.73	0.29	1.54	1.24	2.53
Pkp1	1.39	0.35	0	3.48	0
Pkp2	0.65	0.43	4.6	0.3	0.37
Pkp3	1.1	1.7	1.49	1.81	0
Pkp4	1.09	0	1.77	2.63	4.27
Pla1a	0.64	2.12	1.62	0.04	2.92
Pla2g10	3.09	2.3	0.59	1.12	0
Pla2g12a	0	2.21	1.89	0.99	0.93
Pla2g12b	0	NA	0	1.4	1.89
Pla2g15	NA	0	0.25	1.08	0
Pla2g16	NA	1.78	1.21	0.64	0
Pla2g1b	0	0.09	0.22	1.55	0
Pla2g2a	1.43	0.97	0.68	0.14	0
Pla2g2c	NA	NA	NA	2.31	NA
Pla2g2d	0.74	0.24	0	0.66	2.53
Pla2g2e	0.92	1.04	1.73	1.06	9.24
Pla2g2f	1.11	1.62	0	1.01	2.6

Pla2g3	1.65	0.59	1.56	0	0
Pla2g4a	0	0	2.93	2.08	0
Pla2g4b	0	0	0.66	NA	NA
Pla2g4c	NA	1.54	0.71	0	1.84
Pla2g4d	NA	NA	0.95	1.45	NA
Pla2g4f	NA	NA	0	4.49	16.86
Pla2g5	7.95	1.35	2.3	2.04	5.5
Pla2g6	1.37	0.58	0.49	0.88	0
Pla2g7	0.49	3.08	2.1	NA	0
Pla2r1	NA	1.6	0.45	0.18	1.24
Plaa	0	0.55	1.08	0.5	0
Plac1	2.64	0.73	0.8	2.13	3.37
Plac1l	NA	NA	0.1	0.6	NA
Plac8	0	0	1.26	0.88	0
Plac8l1	NA	NA	0	0	0.94
Plac9	0.25	NA	NA	NA	NA
Plag1	0.38	1.03	2.02	1.05	1.53
Plagl1	0.06	1.04	1.43	NA	0.85
Plagl2	0	0.76	1.08	1.61	0
Plat	0	0.71	1.07	0.84	2.36
Plau	0.33	0.2	1.68	1.42	0
Plaur	0.14	0.79	4.1	0.06	0
Plb1	NA	NA	0.81	1.27	0.3
Plbd1	NA	0	0.98	2.36	NA
Plbd2	NA	NA	0.71	0	1.97
Plcb1	0.33	0	0.27	2.12	1.2
Plcb2	2.71	2.08	1.66	2.22	0
Plcb3	0.69	1.1	1.6	1.38	0
Plcb4	2.05	0.84	0.48	1.82	0.04
Plcd1	0	0	2.2	0.22	0
Plcd3	0	NA	1.97	2.37	0
Plcd4	0	NA	0.01	NA	3.65
Plce1	2.9	0.34	1.48	0.58	2.61
Plcg1	0	0	0.01	0	0.84
Plcg2	0.31	2.61	2.27	1.24	0.32
Plch1	NA	0	0.74	2.34	0
Plch2	NA	0.78	1.19	2.24	0
Plcl1	2.22	1.18	0.18	2.5	0.72
Plcl2	1.04	0.54	1.55	0.62	2.27
Plcxd1	NA	0	0.8	0.94	1.15
Plcxd2	NA	NA	0.87	1.47	3.75

Plcxd3	0	NA	1.93	3.13	2.24
Plcz1	0.05	NA	1.44	1.91	0.95
Pld1	0	0.18	1.54	1.1	0
Pld2	0.83	1.42	4.6	0	2.61
Pld3	0.41	0	0.66	0	4.66
Pld4	NA	NA	1.32	1.72	0
Pld5	NA	NA	NA	0.57	NA
Pld6	NA	NA	1.37	1.25	1.64
Pldn	0.21	NA	NA	NA	3.37
Plec	NA	0.74	1.17	0	NA
Plec1	0.3	NA	NA	NA	NA
Plek	0.26	0.97	0.64	1.43	0
Plek2	1.45	1.61	1.78	3.76	0
Plekha1	NA	1.56	1.45	1.13	3.81
Plekha2	0.52	1.83	1.19	0.75	0
Plekha3	4.08	NA	3.23	1.02	0.05
Plekha4	0	1.4	0.94	1.31	0
Plekha5	0.78	0.82	1.49	0	0.54
Plekha6	0.55	0.39	2.72	1.22	1.17
Plekha7	0	NA	0.93	0	1.36
Plekha8	NA	NA	1.53	0.83	0
Plekhb1	0.5	1.06	1.52	3.35	2.34
Plekhb2	0	0.53	0.92	0	1.22
Plekhc1	0	NA	NA	NA	NA
Plekhd1	NA	NA	NA	1.93	NA
Plekhf1	0	0	4.15	0	0
Plekhf2	0	1.45	0.74	5.6	2.57
Plekhg1	1.21	NA	0.81	0	4.81
Plekhg2	NA	NA	1.19	0.07	0
Plekhg3	NA	0.98	1.66	1.75	0.05
Plekhg4	NA	NA	0	2.28	0
Plekhg5	NA	NA	0.34	0.42	1.23
Plekhg6	NA	1.11	2.13	0	0
Plekhh1	0.42	NA	0.99	0.13	0.58
Plekhh2	4.22	NA	0.71	0	2.06
Plekhh3	NA	3.11	2.59	1.27	0
Plekhj1	0.01	1.85	1.18	16.67	1.87
Plekhk1	2.45	NA	NA	NA	NA
Plekhm1	0.19	1.06	1.29	1.39	0
Plekhm2	0.78	0.75	1.77	1.28	0
Plekhm3	NA	NA	1.08	1.06	3.62

Plekhn1	NA	NA	0.39	6.38	0
Plekho1	NA	0.05	0.62	3.29	0
Plekho2	NA	0	0.21	1.93	0
Plekhs1	NA	2.31	2.39	0.37	NA
Plg	0.16	1.04	0.6	0	2.72
Plgrkt	NA	1.28	2.29	0.87	NA
Plin	3.08	NA	NA	NA	NA
Plin1	NA	NA	NA	2.17	NA
Plin2	NA	0.85	0.93	1.48	0
Plin3	NA	2.1	1.56	0.52	0
Plin4	NA	NA	1	1.59	0
Plin5	NA	NA	1.07	NA	0
Plk1	0.17	1.18	2.89	0	0
Plk1s1	NA	0	0.54	2.06	NA
Plk2	0.84	0	1.34	0	0
Plk3	0.82	0.23	1.21	NA	0
Plk4	0	0	0.86	0.71	0
Plk5	NA	NA	0.85	NA	NA
Pllp	NA	0.84	1.19	0.56	5.93
Pln	6.06	0.67	1.47	8.26	2.52
Plod1	0.12	0	2.52	1.81	1.56
Plod2	0	0	1.96	1.53	2.6
Plod3	1.14	3.19	0.89	0.52	0
Plp1	NA	2.19	0.52	0.95	0
Plp2	0.06	0.55	1.4	0	0
Plrg1	0.29	NA	1.24	0	3.14
Pls1	NA	2.01	0.67	0.9	0
Pls3	0.81	0.21	2.19	0.23	1.59
Plscr1	0.59	0.1	2.2	0	0
Plscr2	1.34	0.44	1.23	0.07	0
Plscr3	7.7	2.06	0.88	NA	0
Plscr4	0.77	1.23	2.82	0.79	0
Pltp	1.01	1.27	0.27	NA	7.4
Plunc	0	NA	NA	NA	2.79
Plvap	0.63	0	1.05	0.78	0
Plxdc1	2.11	2.8	0.65	2.13	0.35
Plxdc2	1.96	NA	2.42	0	2.89
Plxna1	0	0.56	0.44	1.59	0.67
Plxna2	0.56	0.68	2.33	3.6	1.39
Plxna3	0.89	2.19	0.71	0.27	1.32
Plxna4	NA	NA	2.31	0	0

Plxnb1	2.36	0.82	0.17	0	0
Plxnb2	0.56	0.63	1.72	1.17	1.58
Plxnb3	2.06	1.89	2.74	1.29	0
Plxnc1	1.32	0.05	4.24	1.2	0.1
Plxnd1	0.49	0	0.78	0	0
Pm20d1	NA	NA	2.17	0	0.79
Pm20d2	NA	NA	1.53	0	NA
Pmaip1	0.75	0.53	3.73	1.75	3.17
Pmch	NA	0.17	2.39	NA	0
Pmel	NA	3.6	0.13	0.17	NA
Pmepa1	NA	1.54	2.42	2.35	0
Pmf1	0.51	1.07	1.31	0.37	0
Pmfbp1	2.78	0.09	0.06	0.75	2.92
Pml	0.34	1.38	0.44	0	0
Pmm1	0	1.33	1.39	0.53	2.9
Pmm2	0	0.56	0.02	0.4	0
Pmp2	NA	3.02	1.39	2.38	0
Pmp22	0.32	0.22	0.65	2.28	0.65
Pmpca	0.16	2.92	1.95	0.07	2.23
Pmpcb	3.4	1.96	1.68	0.63	2.44
Pms1	0	0	1.3	0.47	0
Pms2	0.56	NA	NA	0.76	NA
Pmvk	0.05	1.9	2.9	1.76	0
Pnck	5.98	NA	0.66	1.21	1.75
Pnkd	NA	NA	0.48	0.13	0.55
Pnkp	0	2.74	2.16	0	2.61
Pnldc1	NA	NA	0.49	0.02	NA
Pnlip	0.82	0	0.33	4.28	1.7
Pnliprp1	2.59	2.77	1.28	1.23	3.47
Pnliprp2	0	1.12	0.93	0.44	2.99
Pnma1	0	0	0.1	1.8	0
Pnma2	2.03	0.42	0.31	1.28	2.61
Pnma3	5.49	0	0.33	1.82	2
Pnma5	NA	NA	NA	5.59	NA
Pnmal1	NA	1	1.01	0.98	0
Pnmal2	NA	NA	1.45	NA	2.35
Pnmt	1.3	2.06	0.96	0.15	2.13
Pnn	0.03	0.65	0	0.3	0
Pno1	NA	1.49	0.6	0.26	0
Pnoc	0.7	2.68	2.19	2.23	5.47
Pnp	NA	0.38	0	3.55	NA

Pnpla1	NA	NA	3.34	2.49	NA
Pnpla2	0.29	0.74	2.09	1.58	0.06
Pnpla3	NA	2.81	0	0	2.09
Pnpla5	2.23	NA	1.71	0.28	3.55
Pnpla6	NA	2.46	1.9	1.34	0.82
Pnpla7	NA	NA	1.72	0.74	0
Pnpla8	NA	NA	2.35	1.54	0
Pnpo	NA	0.12	2.92	0.85	0
Pnpt1	0.3	NA	2.23	0	0
Pnrc1	0	1.42	1.15	1.71	0
Pnrc2	0.54	0.28	0.99	0.06	0
Poc1a	NA	NA	2	1.62	NA
Poc1b	NA	3.27	2.2	NA	NA
Poc5	NA	NA	0.82	0.63	NA
Podn	0.91	NA	1.97	0.07	0
Podnl1	NA	NA	NA	1.42	NA
Podxl	0.87	0.87	0.83	1.05	1.69
Podxl2	0	0	1.09	0.43	2.36
Pof1b	0.83	0.04	1.43	1.29	3.23
Pofut1	0.42	1.16	2.33	0	0.73
Pofut2	0.5	0.69	1.82	1.52	0
Pogk	1.94	1.66	0.64	2.47	2.05
Poglut1	NA	0.82	0.83	1.29	NA
Pogz	0	0	4.47	1.72	0.85
Pola1	NA	0.89	2.33	0.17	1.55
Pola2	0.22	0	1.39	2.37	0
Polb	2.7	1.79	0.55	0.02	0.81
Pold1	0.44	0.02	2.79	0	0
Pold2	0.17	1.98	1.76	0.24	0
Pold3	0.35	1.22	1.9	0.77	0
Pold4	0	3.54	0.9	0.86	0
Poldip2	0	2.58	0.17	1.04	0
Poldip3	0	0.6	0.19	0	0.86
Pole	0.19	1.75	3.25	0	0
Pole2	0	0.1	0.97	0.86	0
Pole3	0.05	1.12	1.86	1.62	0
Pole4	0.05	NA	1.29	1.15	1.48
Polg	0	0	2.07	NA	0.49
Polg2	0.53	0.26	2.82	0	0
Polh	1.13	0.95	1.43	0.11	0
Poli	0	3.76	0.96	0.61	2.42

Polk	1.1	NA	1.28	1.89	0
Poll	0.27	0	3.48	0.64	0.94
Polm	9.72	0	0.34	0.32	0.56
Poln	2.76	NA	1.29	1.86	1.43
Polq	0	1.18	2.59	0.37	15.15
Polr1a	NA	NA	0.52	3.02	1.38
Polr1b	NA	1.51	0.72	0.18	3.15
Polr1c	NA	0	1.4	0.74	1.77
Polr1d	NA	1.75	2.69	1.11	0
Polr1e	NA	0.89	1.9	0.8	0
Polr2a	0.6	0.84	1.06	0	0
Polr2b	2.08	0.83	3.07	0.64	3.24
Polr2c	6.49	0.5	2.63	0.71	2.88
Polr2d	NA	1.61	0.12	1.27	8.97
Polr2e	0	0	0.02	0.93	5.85
Polr2f	NA	0	0.28	0	0
Polr2g	0.21	1.51	1.77	2.93	0
Polr2h	1.14	2.09	2.46	0.32	4.58
Polr2i	0.23	1.82	1.83	0	0
Polr2j	0.32	0	1.63	0.06	0
Polr2k	0.36	0.02	1.61	0	0
Polr2l	0.08	NA	NA	0.8	NA
Polr2m	NA	NA	1.19	0.8	NA
Polr3a	0.27	NA	1.65	4.93	0.28
Polr3b	0	0	0.54	0.26	0
Polr3c	1.99	0	0.02	0.59	4.29
Polr3d	0.97	1.36	0.5	1.55	1.49
Polr3e	0.45	1.68	0.55	1.68	0
Polr3f	0.44	0.85	0.7	NA	0
Polr3g	0	1.25	0.34	0.11	0.56
Polr3gl	NA	NA	1.78	0	0
Polr3h	0	NA	2.22	0	0.79
Polr3k	0	0.52	0.89	NA	0.19
Polrmt	1.03	0.18	2.89	0.55	0
Pols	0.84	NA	NA	NA	NA
Pom121	NA	1.4	5.22	NA	0.03
Pom121l12	NA	NA	2.04	NA	NA
Pom121l2	NA	1.87	2.08	0.8	3.27
Pomc	NA	2.65	4.26	0.88	1.95
Pomgnt1	NA	0.19	0.11	3.05	1.53
Pomp	NA	0	2.99	0	2.01

Pomt1	0.04	0	2.88	2.57	0
Pomt2	0	2.35	0.6	0.75	2.4
Pon1	1.38	1.47	1.01	0.75	2.79
Pon2	0	0	0.83	0.32	0
Pon3	3.54	2.22	1.02	1.64	1.85
Pop1	0.58	1.89	0	1.32	0
Pop4	0	0.45	1.3	0.52	2.27
Pop5	0.68	0	0.97	2.98	2.09
Pop7	2.79	1.36	2.44	0	0
Popdc2	0.12	1.08	1.11	0	0
Popdc3	3.64	2.64	3.43	1.33	3.71
Por	0	0	2.96	2.05	0
Porcn	2.23	1.71	1.68	0	1.79
Postn	0.51	0.59	1.21	1.34	0
Pot1	0.12	NA	NA	NA	NA
Pou1f1	NA	0	1.23	0.95	0.1
Pou2af1	0.23	0.04	2.57	2.18	2.28
Pou2f1	0.48	1.6	0.63	2.99	0.62
Pou2f2	0.18	1.71	2.36	2.12	0.3
Pou2f3	1.07	1.45	1.55	1.23	7.13
Pou3f1	1.83	0.04	3.39	2.89	NA
Pou3f2	0.06	1.63	0.73	1.17	5.3
Pou3f3	0.24	1.38	3.38	5.16	0
Pou3f4	0.42	1.51	2.93	2.54	0
Pou4f1	0.11	0.48	1.29	0.07	0
Pou4f2	0.11	2.5	1.06	0.68	2.49
Pou4f3	1.03	2.13	0.99	1.66	0
Pou5f1	0.33	NA	NA	NA	NA
Pou5f2	NA	NA	0.35	0.58	NA
Pou6f1	1.2	0	0.88	2.45	1.55
Pou6f2	3.34	1.98	1	1.59	0
Pp2d1	NA	NA	NA	1.03	NA
Ppa1	NA	1.38	2.19	0	6.64
Ppa2	NA	4.08	2.61	0	NA
Ppan	0.24	1.22	1.75	NA	0
Ppap2a	0.88	1.53	1.28	0.58	1.7
Ppap2b	0.21	1.93	2.3	1.08	1.3
Ppap2c	1.5	0.95	2.42	6.2	1.45
Ppapdc1a	NA	NA	0	1.51	2.65
Ppapdc1b	NA	NA	1.57	0.18	7.05
Ppapdc2	NA	NA	1.35	NA	0

Ppapdc3	NA	NA	1.68	0.89	0
Ppara	5.83	1.8	0.63	0	0.49
Pparbp	0.28	NA	NA	NA	NA
Ppard	0.55	1.22	2.36	0	0.05
Pparg	0.29	2.12	0.94	0.96	6.29
Ppargc1a	0.79	2.31	0.75	0	1.15
Ppargc1b	0.64	NA	0.56	1.8	NA
Ppat	0	0	0.88	1.01	0.72
Ppbp	NA	1.23	1.27	5.68	0.41
Ppcdc	NA	2.19	0.52	0.69	0.93
Ppcs	NA	3.31	1.59	1.29	0
Ppdpf	NA	0.66	1.04	0	0
Ppef1	NA	NA	NA	2.79	NA
Ppef2	0.58	2	0.6	0.31	1.2
Ppfia1	0.66	0.88	0.83	4.51	0.61
Ppfia2	NA	0.99	0.11	1.52	0
Ppfia3	NA	0	4.62	1.48	0
Ppfia4	0	1.13	0.05	2.27	2.4
Ppfibp1	0.65	0.66	0.06	0.71	0
Ppfibp2	0.49	0.57	0.02	2.29	2.06
Pphln1	NA	NA	0.89	0.66	1.42
Ppia	0.08	1.51	1.48	0.1	0
Ppib	0	1.97	6	NA	0
Ppic	0.01	0.98	1.6	0.31	1.84
Ppid	1.23	NA	NA	0.78	NA
Ppie	12.66	1.34	1.57	1.68	4.32
Ppif	0	0.06	1.83	1.1	2.31
Ppig	0	0	0.82	0.75	1.03
Ppih	0.2	0.98	1.54	1.13	0.21
Ppil1	0.47	NA	2.2	0.12	2.35
Ppil2	0.47	0	0.43	0	1.64
Ppil3	0	1.2	0.97	0.04	0
Ppil4	0.34	NA	3.07	0.86	2.51
Ppil5	2.15	NA	NA	NA	1.69
Ppil6	3.9	1.3	0.49	2.34	0
Ppip5k1	NA	0.08	1.22	3.81	NA
Ppip5k2	NA	1.74	0	1.06	NA
Ppl	0.02	1.63	1.61	0.02	1.8
Ppm1a	0.97	1.3	2.51	3.8	0.98
Ppm1b	0.02	2.5	1.65	1.69	0
Ppm1d	0.54	2.18	1.43	0.17	NA

Ppm1e	0	4.13	0	2.11	0
Ppm1f	0	1.77	1.9	4.66	0
Ppm1g	1.41	0	0	0.5	0
Ppm1h	0.46	1.54	1.43	2.26	0
Ppm1j	NA	NA	1.52	0	0.17
Ppm1k	NA	NA	0.93	0.75	2.73
Ppm1l	0.14	NA	0.56	0.41	1.89
Ppm1m	NA	NA	1.31	2.4	3.41
Ppm1n	NA	NA	3.35	0.9	NA
Ppme1	NA	1.07	1.09	0.04	1.58
Ppox	0	0.67	1.14	0	0
Ppp1ca	0	0	3.55	0.77	0
Ppp1cb	0.88	0.57	5.19	0.74	0.66
Ppp1cc	0.42	0.65	2	1.76	5.53
Ppp1r10	0	NA	NA	0.02	2.76
Ppp1r11	0.81	1.95	0	1.9	0.3
Ppp1r12a	0.61	0	2.21	0.02	0
Ppp1r12b	NA	0	0.57	0	1.45
Ppp1r12c	0	NA	0.72	0.59	0
Ppp1r13b	0.1	0	3.79	0	1.19
Ppp1r13l	NA	1.56	1.27	1.45	1.42
Ppp1r14a	0.71	NA	0	1.07	0
Ppp1r14b	0.14	0.01	1.23	0.51	3.41
Ppp1r14c	0.91	NA	0	3.4	7.57
Ppp1r14d	0.87	0.25	0	1.7	7.76
Ppp1r15a	NA	2.51	1.96	0.86	0
Ppp1r15b	0.48	NA	0.2	2.07	1.47
Ppp1r16a	3.06	NA	0.9	1.43	0.3
Ppp1r16b	0.84	0	0.54	1.48	2.65
Ppp1r17	NA	3.67	1.18	0	NA
Ppp1r18	NA	NA	1.19	0.68	NA
Ppp1r1a	NA	1.93	2.33	1.18	0
Ppp1r1b	0.61	NA	3.48	0.47	0.81
Ppp1r1c	0.3	NA	3.21	1.04	2.34
Ppp1r2	0.78	0.37	1.52	1.39	2.55
Ppp1r21	NA	NA	2.08	0.05	NA
Ppp1r26	NA	2.54	2.74	0.56	NA
Ppp1r27	NA	NA	1.28	1.82	NA
Ppp1r32	NA	NA	1.73	1.03	NA
Ppp1r35	NA	NA	0	5	NA
Ppp1r36	NA	NA	1.3	0.3	NA

Ppp1r37	NA	1.61	2.04	3.94	NA
Ppp1r3a	1.17	0.56	0.47	1.47	2.13
Ppp1r3b	0.35	NA	0.58	0.84	0
Ppp1r3c	2.23	1.06	4.32	0	0
Ppp1r3d	NA	0.28	1.78	2.14	0
Ppp1r3e	0.05	NA	1.05	19.77	0
Ppp1r3f	1.06	NA	1.45	3.39	0
Ppp1r42	NA	NA	NA	0.34	NA
Ppp1r7	1.05	0.73	0.84	0.83	1.35
Ppp1r8	0.48	0.04	1.85	1.16	2.97
Ppp1r9a	0.92	1.25	0.53	0	0.15
Ppp1r9b	1.19	NA	NA	1.26	NA
Ppp2ca	0.35	0.75	2.42	0.07	1.04
Ppp2cb	0	0.45	0.11	NA	NA
Ppp2r1a	0.39	2.38	0.32	0	1.86
Ppp2r1b	0.28	0.1	1.53	0.85	0
Ppp2r2a	0.67	1.87	1.22	0.35	0
Ppp2r2b	NA	0	0.96	2.44	0.73
Ppp2r2c	NA	NA	0.4	0.72	1.48
Ppp2r2d	0.65	1.35	0.97	0.7	0.09
Ppp2r3a	0.37	0	0.79	1.37	1.34
Ppp2r3c	NA	0	1.87	0.94	1.89
Ppp2r4	0.98	1.61	0.48	8.66	0.89
Ppp2r5a	1	1.39	2.53	1.15	0
Ppp2r5b	6.4	2.37	0.66	0.35	2.34
Ppp2r5c	0.48	0.66	0.25	0.12	1.53
Ppp2r5d	0.59	0	2.37	0.57	2.59
Ppp2r5e	0	0.93	1.07	0	3.12
Ppp3ca	0.29	1.03	0.85	0.31	0.24
Ppp3cb	0	1.27	2.51	1.26	0
Ppp3cc	0	0	2.48	0.59	0.86
Ppp3r1	0	1.31	1.77	0.5	1.63
Ppp3r2	0.06	NA	2.42	1.12	0.16
Ppp4c	0	1.43	0	1.07	0
Ppp4r1	0.3	1.51	1.63	6.59	1.8
Ppp4r1l	NA	NA	NA	NA	0
Ppp4r2	1.25	1.5	1.54	0.53	0
Ppp4r4	NA	1.49	0.4	0	3.63
Ppp5c	1.29	0.85	2.45	1.55	0
Ppp6c	0.56	2.08	1.36	1.07	0
Ppp6r1	NA	0	4.57	1.3	NA

Ppp6r2	NA	0	0	0	NA
Ppp6r3	NA	1.46	0.09	0	NA
Pppde1	NA	NA	NA	NA	2.16
Pppde2	NA	NA	NA	NA	1.17
Pprc1	0	2.47	0	2.4	3.26
Ppt1	1.75	0.74	1.72	0.66	0
Ppt2	0.29	3.06	0.59	NA	1.42
Pptc7	NA	NA	2.71	0.89	0
Ppwd1	NA	0	0	1.19	0
Ppy	0	1.08	1.09	0	2.99
Ppyr1	0	NA	NA	NA	6.64
Pqbp1	0.16	1.01	0.26	0.83	0
Pqlc1	NA	0.53	0.02	1.08	1.88
Pqlc2	0.68	2.19	0.59	0.97	2.11
Pqlc3	NA	0.51	2.09	1.22	1.04
Pradc1	NA	NA	2.12	1.96	NA
Praf2	NA	2.21	0.3	2.36	0
Pram1	NA	NA	3.85	2.38	11.49
Prame	NA	0.49	5.95	2.26	2.52
Pramef12	NA	0.81	0	2.5	0
Prap1	1.33	NA	0.72	2.01	2.42
Prc1	1.01	0.4	0.5	0.05	1.84
Prcc	1.34	1.48	3.55	0	0.94
Prcp	NA	1.92	2.3	0.82	2.48
Prdm1	0.87	2.7	1.09	4.03	3.16
Prdm10	NA	1.31	0.81	0.57	1.36
Prdm11	NA	2	2.04	0.48	0
Prdm13	1.32	NA	NA	2.94	NA
Prdm14	NA	2.26	1.46	1.82	2.03
Prdm15	3.31	NA	1.15	0.34	0.66
Prdm16	0.8	0.9	0.84	0.46	2.4
Prdm2	1.09	0	3.56	0	0.58
Prdm4	0.23	1.05	1.42	1.8	2.64
Prdm5	0	1.85	1.26	2.14	2.37
Prdm6	NA	NA	0.66	0.87	0
Prdm8	0	4.07	2.33	1.44	0
Prdm9	NA	0.82	0.79	1.51	2.64
Prdx1	0.99	0	0.27	0	0.14
Prdx2	0.29	0.76	0.83	0.81	1.27
Prdx3	0.72	1.58	2.06	1.12	0.4
Prdx4	0.54	0	1.43	2.04	0

Prdx5	2.04	NA	0.16	NA	3.76
Prdx6	1.21	0.86	0.27	0	1.23
Preb	0.02	1.91	0.95	0.83	0
Prei3	0	NA	NA	NA	NA
Prelid1	NA	NA	0.88	0.83	0.55
Prelid2	NA	NA	0.68	2.74	0.23
Prelp	0.22	0.77	0.97	1.43	1.75
Prep	0.38	0.08	0.18	0.62	1.97
Prepl	NA	0	0.56	0.68	0
Prex1	NA	NA	3.35	0	0
Prex2	NA	0	1.17	1.18	0.66
Prf1	0	1.65	1.01	1.75	10.08
Prg2	0.98	0.49	1.48	1.37	0
Prg3	2.7	0.72	1.23	0.82	0
Prg4	1.99	1.31	0	0	5.53
Prh1	1.84	NA	NA	NA	NA
Prhoxnb	NA	NA	NA	1.44	NA
Prickle1	0.01	NA	0.82	0	0.5
Prickle2	3.89	NA	0.47	0	0
Prickle3	NA	0.14	0.36	4.57	1.73
Prim1	0	0.94	2.13	0.55	2.55
Prim2	NA	0	1.02	0.34	0.49
Prima1	0.45	NA	1.33	2.1	6.76
Prkaa1	0.96	0.83	2.1	0.8	3.41
Prkaa2	0	2.21	2.13	0	0
Prkab1	0	1.63	1.54	1.3	1.78
Prkab2	0	2.81	1.56	0	1.29
Prkaca	0	1.2	0.14	NA	0.65
Prkacb	0.68	0.15	1.11	0.61	1.66
Prkag1	5.34	0.45	4.49	2.04	1.81
Prkag2	NA	0	0.76	0	0.86
Prkag3	0	NA	4.58	1.37	0
Prkar1a	0.49	0.32	1.92	0.43	0.54
Prkar1b	1.24	0.68	0.79	1.6	0
Prkar2a	0.1	0	0.91	1.14	1.17
Prkar2b	0.68	1.84	2.45	3.35	0.78
Prkca	0	0.35	0.18	0	0.56
Prkcb	NA	0	0.76	1.96	0.81
Prkcd	0.41	0.27	3.51	1.47	0
Prkcdbp	1.58	0.47	2.92	0	1.22
Prkce	0.38	2.1	0.02	1.32	0

Prkcg	NA	2.38	0.66	1.89	NA
Prkch	1.66	0	2.15	0.88	2.51
Prkci	0	0.43	1.11	2.03	0
Prkcq	0.47	0	0.72	1.21	4.15
Prkcsh	0.33	0	0.56	0	0
Prkcz	1.16	0	0.82	0.23	0
Prkd1	NA	0.47	0	1.47	0
Prkd2	NA	0.2	0.4	0	0
Prkd3	NA	0	0.89	4.19	1.86
Prkdc	0	0	0.67	0	0.97
Prkg1	0.32	0	0.85	0	0
Prkg2	NA	0	0.56	1.62	0.97
Prkra	NA	1.55	0.35	0.19	0
Prkrip1	0.81	1.68	1.12	0.81	2.41
Prkrir	0.02	1.03	3.36	4.02	3.86
Prkx	0.39	1.91	1.34	4.72	0
Prl	0	0	0	1.11	0
Prlh	NA	NA	NA	0.13	NA
Prlhr	NA	NA	NA	2.3	NA
Prlr	0.27	0.43	0.72	0.62	3
Prm1	1.27	0.75	0.51	5.37	1.33
Prm2	1.04	2.03	0.67	NA	0
Prm3	2.53	NA	2.09	0	0
Prmt1	NA	2.45	0.11	3.17	2.93
Prmt10	NA	NA	1.05	0.74	0
Prmt2	NA	1.65	2.27	NA	0
Prmt3	NA	0	0.25	1.08	0
Prmt5	NA	1.56	0	1.01	2.81
Prmt6	NA	NA	0.81	1.23	0
Prmt7	NA	0	0.7	2.42	2.68
Prmt8	NA	0	0.48	2.61	1.18
Prnd	0	2.28	2.11	2.22	7.34
Prnp	0.47	0.13	0.33	1.87	2.02
Proc	1.9	0.57	2.98	1.55	0.78
Proca1	NA	NA	0	0.22	0
Procr	3.55	1.41	2.9	1.84	1.7
Prodh	5.62	0.39	1	1.25	1.84
Prodh2	0.48	2.28	0.84	2.58	0
Prok1	0.94	NA	NA	1.29	NA
Prok2	2.13	NA	0.97	0.97	0
Prokr1	NA	NA	NA	0.63	NA

Prokr2	NA	NA	0.02	0	1.33
Prol1	NA	2.94	0.45	0.62	0.99
Prom1	1.33	1.62	1.79	0.6	0
Prom2	1.91	NA	0	2.15	1.68
Prop1	1.34	1.94	0.12	4.17	0
Pros1	1.25	3.95	1.38	0	1.55
Prosapip1	0.89	NA	NA	NA	1.34
Prosc	0	0.71	0.18	0.77	0
Proser1	NA	1.48	0.77	1.49	NA
Proser2	NA	NA	0	1.42	NA
Prox1	3.21	1.21	0.26	1.14	0
Prox2	NA	NA	1.57	0.54	0.09
Proz	1.27	1.21	0	0.6	0
Prpf18	NA	1.3	0.9	0.71	0
Prpf19	NA	2.09	1.6	2.44	0
Prpf3	0.64	1.58	1.61	0.84	0.02
Prpf31	0	1	0.95	NA	0
Prpf38a	NA	NA	7.3	0.52	1.95
Prpf38b	NA	0.69	0.94	1.17	1.29
Prpf39	0.19	1.21	0.87	0	1.68
Prpf4	0.07	0	0	0.94	3.77
Prpf40a	NA	0	0.4	0.29	0
Prpf40b	NA	NA	0.31	NA	3.42
Prpf4b	0.23	0.12	0.32	0.62	0
Prpf6	NA	1.2	0.4	0	2.61
Prpf8	1.51	0	0.04	0	3.13
Prph	NA	0.81	1.76	0.83	0
Prph2	1.75	2.85	0	8.45	7.72
Prps1	0.25	0	1.11	0.6	3.46
Prps1l1	NA	1.6	0.81	5.6	8.91
Prps2	1.19	0.54	1.81	0.53	2.28
Prpsap1	0	0	2.27	0.75	2.26
Prpsap2	0	0	2.22	1.16	0
Prr11	NA	0	2.58	0.9	0.39
Prr12	NA	NA	0.81	1.08	0
Prr13	NA	0.07	1.44	1.8	0
Prr14	NA	1.78	0.38	2.02	0.3
Prr14l	NA	2.07	0.8	0.54	NA
Prr15	NA	NA	0.37	2.74	0
Prr15l	NA	1.75	0	2.62	NA
Prr16	NA	0	0.21	5.5	2.21

Prr18	NA	NA	0.79	1.1	4.16
Prr19	NA	NA	NA	1	NA
Prr23a	NA	NA	NA	0.23	NA
Prr24	NA	NA	1.88	NA	NA
Prr3	1	0	1.28	1.36	1.29
Prr5	NA	0	0.17	NA	NA
Prr5l	NA	0	0.16	1.56	4.22
Prr7	NA	NA	NA	0.99	NA
Prr9	NA	NA	0.13	NA	11.99
Prrc1	NA	0.76	0.83	0.23	0
Prrc2a	NA	0.93	1.04	1.22	NA
Prrc2b	NA	0	1.69	1.12	NA
Prrc2c	NA	0	0.54	2.26	NA
Prrg1	NA	1.57	1.21	0.51	1.15
Prrg2	0.72	0	0	0.62	0
Prrg3	NA	1.53	1.3	2.96	1.88
Prrg4	3.38	1.91	1.11	0.77	NA
Prrt1	NA	NA	1.47	0.97	2.67
Prrt2	NA	NA	3.37	0	NA
Prrt3	NA	NA	1.2	0.45	NA
Prrx1	0.05	0.38	0.85	2.4	0
Prrx2	3.15	0.93	0.7	0.14	4.88
Prss12	0.24	1.46	1.26	0.75	0
Prss16	0	3.9	3.53	0.7	0
Prss2	3.11	0.73	0.36	1	0.69
Prss21	0.73	0	1.47	1.17	3.21
Prss22	0.59	0.72	2.7	1.71	1.58
Prss23	NA	0	1.9	0	1.16
Prss27	NA	NA	1.26	1.2	NA
Prss3	NA	0	1.15	1.26	21.56
Prss33	0.85	NA	NA	0.34	NA
Prss35	0.66	NA	1.61	0.44	0.92
Prss36	NA	NA	2.4	1.79	NA
Prss37	NA	NA	0	1.33	NA
Prss38	NA	NA	NA	2.09	NA
Prss41	NA	NA	NA	1.46	NA
Prss42	NA	NA	NA	0	NA
Prss45	NA	NA	NA	1.24	NA
Prss48	NA	NA	NA	7.21	NA
Prss50	NA	2.34	0.69	1.58	NA
Prss53	NA	1.03	0.06	1.15	NA

Prss54	NA	NA	0.48	0.28	NA
Prss55	NA	NA	0.29	2.96	NA
Prss58	NA	NA	0.88	2.94	NA
Prss7	0.53	NA	NA	NA	NA
Prss8	2.12	1.53	0	1.26	0
Prssl1	0	NA	NA	NA	NA
Prtg	NA	NA	0	2.18	0
Prtn3	0	0.03	4.87	1.1	1.19
Prune	NA	0	0.39	2.74	1.24
Prune2	NA	0	0.84	0.92	2.5
Prx	1.13	2.44	1.34	2.29	2.58
Psap	1.88	1.98	1.71	0	5.42
Psapl1	NA	NA	1.45	1.21	NA
Psat1	0.29	1.38	2.24	1	0.97
Psca	1.14	2.08	1.36	1.36	1.61
Pscd1	0.02	NA	NA	NA	NA
Pscd2	0.89	NA	NA	NA	NA
Pscd3	0.78	NA	NA	NA	NA
Pscd4	0.05	NA	NA	NA	NA
Pscdbp	0.98	NA	NA	NA	NA
Psd	NA	1.02	0.56	1.74	0.11
Psd2	0.73	NA	1.41	0.19	2.57
Psd3	NA	0.64	1.18	0	1.36
Psd4	1.25	0	1.37	1.33	0.96
Psen1	0	0	0.74	0	0
Psen2	0.12	0.69	0.54	4.38	1.14
Psenen	NA	1.95	4.02	0	1.73
Psip1	1.67	0	1.95	1.83	0
Pskh1	NA	0	0.54	0	0.17
Psma1	0.64	0.03	1.36	6.93	1.33
Psma2	0	0	0.54	1.1	2.08
Psma3	0	0	1.57	2.16	0
Psma4	0.49	0	1.88	NA	0.97
Psma5	0.19	0.83	1.05	0.26	0
Psma6	1.63	0	1.02	0.81	1.07
Psma7	0	0	3.17	0	0
Psma8	NA	NA	2.3	2.42	7.26
Psmb1	0.73	0	0.92	0.04	1.07
Psmb10	0.63	0.7	2.58	0	1.38
Psmb11	NA	NA	NA	0.6	NA
Psmb2	0.03	1.11	1.28	1.86	5.5

Psmb3	0.55	0	0.7	0.08	1.95
Psmb4	0.87	0	2.1	1.04	0.14
Psmb5	3.49	0	1.18	9.44	1.7
Psmb6	1.8	0	1.91	0	1.74
Psmb7	0.32	0	0.33	0.35	0
Psmb8	0.5	0.21	0.53	0.71	0
Psmb9	0.52	0.78	2.21	2.8	0.55
Psmc1	1.04	0.01	1.39	0.98	0
Psmc2	0	0.7	0.13	1.14	0
Psmc3	0.44	1.27	0.16	1.83	0
Psmc3ip	3.28	0.07	2.95	0.86	0.43
Psmc4	0.17	1.77	0.46	0.02	0
Psmc5	0.47	2.01	1.78	1.28	0
Psmc6	0.5	0.11	1.91	0.43	0
Psmd1	0.08	0.94	1.13	0.45	0
Psmd10	1.22	1.15	3.57	0	3.5
Psmd11	0.69	0.75	1.57	1.85	2.05
Psmd12	0.35	0.18	1.69	2.39	3.41
Psmd13	0.12	0.84	1.48	0	0.19
Psmd14	0.43	0.36	3.38	0.86	2.66
Psmd2	0.09	0.97	1.02	0.36	1.85
Psmd3	0.31	2.49	0.05	1	4.35
Psmd4	0.6	1.09	0.93	1.3	1.11
Psmd5	0.01	1.6	1.62	0	0
Psmd6	NA	0	2.48	0.79	0.51
Psmd7	1.14	0.86	0.07	4.42	3.67
Psmd8	0.66	0.64	3.35	0	3.71
Psmd9	0.48	1.27	0.6	0.57	0.8
Psme1	0.57	1.5	3.26	0	3.39
Psme2	0.39	0.48	1.54	2.48	2
Psme3	0.28	0.69	0	0.84	1.37
Psme4	0.6	0.44	0.1	0	0
Psmf1	0	0.01	0.51	1.01	0
Psmg1	NA	1.57	3.86	0	0
Psmg2	NA	0	0.94	0	3.54
Psmg3	NA	NA	0.36	3.28	2.37
Psmg4	NA	NA	1.88	1.7	0.72
Psors1c2	1.5	1.22	0.85	1.84	1.86
Pspc1	1.2	0.14	0.27	0	1.29
Psph	1.12	2.39	2.1	0.88	2.13
Pspn	0	1.96	4.2	0.33	0

Psrc1	NA	1.04	0.24	2	0.41
Pstk	NA	NA	0.54	0.03	0
Pstpip1	0.69	2.24	3.83	0	0
Pstpip2	0.23	0	1.68	1.29	0
Ptafr	0.76	1.35	1.81	0.59	2.13
Ptar1	NA	NA	1.02	0.55	0
Ptbp1	0.36	0	1.1	0	0
Ptbp2	0.46	0.28	1.4	0.41	0
Ptbp3	NA	2.25	2.78	0	NA
Ptcd1	NA	1.14	0.97	0.77	1.02
Ptcd2	0	0	0	0.85	0.1
Ptcd3	NA	0	0	0.18	2.09
Ptch1	0.22	0.36	0.87	1.72	0.53
Ptch2	2	0.99	1.18	0.01	0
Ptchd1	3.56	NA	0.03	0	2.39
Ptchd2	NA	NA	1.02	2.21	1.61
Ptchd3	NA	NA	NA	0.7	NA
Ptchd4	NA	NA	7.02	1.47	NA
Ptcra	0.19	0.04	2.84	0.97	2.56
Ptdss1	1.96	0	1.77	2.08	0
Ptdss2	0	1.58	0.5	1.56	0.68
Pten	1.11	0.05	0.17	0.47	0
Pter	0.51	1.61	0.72	NA	4.07
Ptf1a	3.41	NA	NA	0.85	NA
Ptgdr	3.06	0.87	0.82	1.46	2.49
Ptgds	2.23	1.46	1.12	1.74	1.56
Ptger1	0.5	0	1.77	1.25	0
Ptger2	0	1.55	1.03	0	0
Ptger3	0.14	0.57	0.71	5.47	1.23
Ptger4	0	1.76	2.22	0.78	2.78
Ptges	0.25	0	0.57	1.78	0
Ptges2	0	2.17	1.46	1.51	0
Ptges3	NA	1.4	1.15	0.15	2.07
Ptgfr	0.6	1.15	0	1.66	0
Ptgfrn	2.15	NA	0.01	1.23	0.14
Ptgir	0.51	1.58	10.46	0.77	1.64
Ptgis	2.1	0.88	0	0.87	2.36
Ptgr1	NA	NA	1	2.33	2.25
Ptgr2	NA	NA	1.17	1.03	0
Ptgs1	0.01	0.97	1.05	0	0.47
Ptgs2	0	2.68	2.19	0.06	0

Pth	1.33	1.73	0	2.86	6.3
Pth1r	NA	3.07	0.63	1.1	0
Pth2	NA	NA	1.68	1.17	NA
Pth2r	NA	1.97	0.02	0	2.03
Pthlh	1.24	0.54	1.09	1.04	1.85
Pthr1	0.06	NA	NA	NA	NA
Pthr2	2.29	NA	NA	NA	NA
Ptk2	0.72	0.01	1.02	1.98	0
Ptk2b	2.79	1.53	1.16	0.36	0
Ptk6	3.88	1.74	0	0.88	0
Ptk7	0	1.24	1.02	0	0
Ptk9	1.02	NA	NA	NA	NA
Ptma	0.19	3.41	0.86	0.13	NA
Ptms	NA	1.28	3.92	0.76	0
Ptn	0.23	0.24	0.6	2.12	0
Ptov1	0	0	0.41	0.78	0
Ptp4a1	1.14	1.33	2.49	0.53	0
Ptp4a2	0.66	0.46	1.87	0	0
Ptp4a3	0.89	0	1.02	3.22	0.02
Ptpdc1	NA	NA	1.51	4.63	0.56
Ptpla	0	2.09	3.93	1.05	1.44
Ptplad1	NA	0.17	0.31	0.59	0
Ptplad2	NA	NA	2.63	2.35	0
Ptplb	0.27	2.82	1.44	0.2	5.68
Ptpmt1	NA	2.25	2.01	1.87	0
Ptpn1	0	0.63	2.9	9.88	0
Ptpn11	0.1	0.28	1.06	1.52	0.69
Ptpn12	0	0.82	3	0.29	0
Ptpn13	0.33	0.33	0.46	1.11	0.52
Ptpn14	0.28	1.7	1.04	0	1.8
Ptpn18	0.82	0.11	2.28	1.99	0
Ptpn2	0.25	0.01	4.87	0.72	0
Ptpn20	12.12	NA	NA	NA	NA
Ptpn21	1.3	0.81	0.63	2.26	2.07
Ptpn22	NA	1.14	0.78	0.95	2.06
Ptpn23	1.45	NA	3.69	0	2.61
Ptpn3	2.41	2.88	2.3	1.89	1.65
Ptpn4	0.34	0	0.84	0.45	5.59
Ptpn5	3.18	NA	0.5	1.85	2.18
Ptpn6	NA	2.56	3.99	0.21	1.39
Ptpn7	0	NA	NA	0.16	NA

Ptpn9	0.73	3.33	2.64	1.24	1.82
Ptpra	1.15	0	0.1	NA	0
Ptprb	0.64	1.39	0.67	3.01	1.47
Ptprc	0.61	1.1	2.12	1.27	0.18
Ptprcap	3.03	0	2.02	NA	0
Ptprd	0.77	0.68	0.3	0	2.18
Ptpre	0	2.06	0.65	1.76	6.07
Ptprf	1.02	1.06	0.46	1.58	0
Ptprg	1.48	0.18	0.72	3.14	0
Ptprh	1.95	NA	NA	0.71	NA
Ptprj	0.27	1.07	0.71	1.05	0.14
Ptprk	0	2.74	0.53	1.05	0.54
Ptprm	0.58	0.69	2.72	0	0.05
Ptprn	0.18	1.95	2.16	0.1	1.02
Ptprn2	0	0.07	1.44	0.86	0
Ptpro	0	0.9	0.96	0	0
Ptprq	7.32	NA	NA	NA	NA
Ptprr	0.44	1.09	1.9	0.25	2.5
Ptprs	1.2	1.19	2.66	0	1.11
Ptprt	0.22	1.07	0	0.63	2.92
Ptpru	1.98	0.99	1.98	0	3.1
Ptprv	0.88	NA	NA	NA	NA
Ptprz1	1.1	0	1.35	1.24	0
Ptrf	0.65	1.39	0.94	0.64	0
Ptrh1	NA	NA	2.52	NA	1.91
Ptrh2	NA	2.11	2.64	1.76	0
Ptrhd1	NA	NA	1.99	0	NA
Pts	0.14	1.96	1.64	2.67	0.28
Pttg1	0.77	1.18	1.1	NA	0.7
Pttg1ip	0.39	2.23	3	0.6	3.76
Ptx3	0	0.04	1.03	0	0
Ptx4	NA	NA	NA	0.15	NA
Puf60	NA	0	0.74	0.71	3.24
Pum1	0.65	0	1.82	2.42	2.87
Pum2	0.06	0.26	1.07	2.86	2.11
Punc	0.44	NA	NA	NA	NA
Pura	2.23	0	1.28	2.01	4.21
Purb	0	NA	0.73	1.29	0.63
Purg	0.55	0.85	2.05	1.11	1.69
Pus1	0	1.87	1.7	4.48	0
Pus10	NA	NA	0	0.31	0.35

Pus3	0.13	2.4	1.39	0.9	2.41
Pus7	NA	1.99	0.89	1.03	NA
Pus7l	NA	0.25	1.05	1.36	1.2
Pusl1	NA	NA	NA	1.03	NA
Pvalb	0.8	0.85	0.56	2.37	6.12
Pvr	0.26	1.05	0.59	0.73	2.26
Pvrl1	0.38	0.99	0.39	0.64	0
Pvrl2	0	1.22	3.56	0.62	0
Pvrl3	0	0.97	0.42	1.32	2.15
Pvrl4	0.27	NA	2.89	1.09	0
Pvt1	1.79	0.91	0.16	NA	1.4
Pwp1	NA	1.25	0.06	0.81	8.43
Pwp2	NA	1.1	1.8	3.85	2.56
Pwwp2	1.21	NA	NA	NA	NA
Pwwp2a	NA	NA	1.36	2.36	2.83
Pwwp2b	NA	NA	1.73	2.53	0.47
Pxdc1	NA	3.12	1.01	1.53	NA
Pxdn	NA	0.86	2.07	1.3	0
Pxk	NA	NA	2.25	1.14	0
Pxmp2	0	2.69	1.01	4.94	0.64
Pxmp3	0.49	NA	NA	NA	0
Pxmp4	0	0	2.04	0.82	0.54
Pxn	0	2.92	3.01	1.95	0.01
Pxt1	1.17	NA	0.12	2.14	NA
Pycard	0	0.9	0.83	0.58	0
Pycr1	0.68	2.61	0	0.77	1.65
Pycr2	0	NA	0.6	3.29	NA
Pycrl	0.1	1.5	1.98	2.55	2.05
Pygb	0.21	0	2.28	0	1.36
Pygl	0.53	0.18	2.19	0	0
Pygm	3.08	1.28	0.62	0.16	0
Pygo1	0	1.1	2.72	2.08	2.24
Pygo2	0.05	NA	0.12	0.87	0.42
Pyhin1	NA	1.1	0.85	0.06	0.71
Pyroxd1	NA	1.41	0.68	0.56	3.12
Pyroxd2	NA	NA	0.86	NA	0.45
Pyurf	NA	NA	NA	0	NA
Pyy	0.44	2.24	0.02	1.01	0
Pzp	0.76	0.32	0	1.81	4.95
Qars	0	0	0.76	1.12	0
Qdpr	0.1	0	0.98	1.74	1.1

Qpct	0	4.09	1.41	5.32	6.77
Qpctl	2.44	1.86	0.97	0.01	0
Qprt	5.03	2.57	1.01	2.15	0
Qrfp	NA	NA	NA	1.86	NA
Qrfpr	NA	NA	1.98	0	NA
Qrich1	NA	1.22	0.41	0.83	1.94
Qrich2	NA	NA	3.97	0.49	3.28
Qrsl1	1.64	0	0.81	1.13	0
Qscn6	0.05	NA	NA	NA	NA
Qscn6l1	0.75	NA	NA	NA	NA
Qser1	NA	1.48	0.85	1.93	0.37
Qsox1	NA	0	1.79	NA	0
Qsox2	NA	NA	4.32	1.87	0
Qtrt1	0	2.18	0.76	2.78	0.69
Qtrtd1	0.56	1.65	1.56	1.55	2.1
R3hcc1	NA	2.29	3.26	0.3	6.32
R3hcc1l	NA	0	0	0.03	NA
R3hdm1	NA	0.13	0.92	0.8	1.07
R3hdm2	NA	2.33	1.31	0.6	2.52
R3hdm4	NA	0	1.04	1.36	NA
R3hdml	NA	NA	NA	1.19	NA
Rab10	1	NA	1.9	0.85	0
Rab11a	0.48	1.99	1.72	2.23	0
Rab11b	2.94	0.65	1.1	1.47	0
Rab11fip1	0.64	2.11	0.37	0.55	4.18
Rab11fip2	1.61	0.67	0.55	0.8	0
Rab11fip3	0	1.52	0.77	1.84	0.95
Rab11fip4	3.37	1.17	0.29	1.87	1.95
Rab11fip5	0.87	2.82	2.28	1.42	2.58
Rab12	0.17	NA	2.29	0.15	0.63
Rab13	2	2.84	0.72	0.87	0.14
Rab14	0.04	0.45	0.75	1.64	0
Rab15	1.34	3.39	0	6.27	5.3
Rab17	0.19	2.57	4.31	2.51	0
Rab18	0.47	NA	1.6	0.91	1.66
Rab19	NA	NA	NA	1.23	NA
Rab1b	NA	2.03	1.03	0.65	1.92
Rab20	0.79	0.02	2.31	2.97	0
Rab21	0.83	1.89	2.1	0.03	0
Rab22a	0	0.59	0.55	0.4	1.14
Rab23	0	1.04	0.36	0.82	0.36

Rab24	0	NA	3.03	0.8	0
Rab25	0.19	0.56	0.3	0.37	6.95
Rab26	1.18	1.41	2.09	1.34	3.02
Rab27a	0.27	0	0.05	1.64	0
Rab27b	0.25	1.55	0.81	0.33	0
Rab28	0.18	0.11	1.58	0.01	2.4
Rab2a	NA	0	0.78	0.92	1.18
Rab2b	0.49	NA	1.46	0.61	0
Rab30	NA	2.53	0.1	0.67	1.18
Rab31	NA	0	2.05	2.09	0.26
Rab32	1.84	0	5.44	0.76	0
Rab33a	NA	0.29	1.05	3.19	2.12
Rab33b	0	0.66	2.07	0	4.89
Rab34	0.16	NA	3.12	1.4	0
Rab35	0	1.09	0.52	2.04	2.38
Rab36	1.65	1.94	0.06	1.15	0.07
Rab37	0.36	NA	0.75	1.99	0
Rab38	0	0.19	2.17	1.39	2.99
Rab39	2.19	NA	NA	NA	NA
Rab39b	2.99	NA	0.63	0.96	0
Rab3a	2.01	2.16	1.03	0.14	3.92
Rab3b	1.23	1.9	0.32	0.71	0
Rab3c	0.32	NA	0.33	0	0.93
Rab3d	0	0.39	1.41	0.12	1.82
Rab3gap1	NA	1.04	0.11	0.61	0
Rab3gap2	NA	1.42	1.26	0	0
Rab3il1	0	1.29	1.47	1.2	1.32
Rab3ip	0	NA	2.08	0.77	0
Rab40b	1.02	0.62	1.18	1.03	0.59
Rab40c	0.21	2.49	1.24	0.88	1.26
Rab42	NA	NA	2.85	0	NA
Rab43	NA	NA	0.79	2.24	1.72
Rab44	NA	NA	NA	1.96	NA
Rab4a	0	0	2.4	NA	1.76
Rab4b	0.87	0.64	1.49	1.72	6.5
Rab5a	0	1.18	1.87	1.1	0
Rab5b	0.21	0.03	0.58	0.52	0.73
Rab5c	0	3.28	1.9	0.03	1.98
Rab6a	NA	0.92	2.81	1.03	NA
Rab6b	0	0.91	1.87	2.25	0
Rab6ip1	0.52	NA	NA	NA	NA

Rab7l1	0.45	0.92	0.86	0	1.33
Rab8a	1.67	1.32	2.63	0.4	1.22
Rab8b	2.17	0	1.94	1.05	0
Rab9b	1.28	NA	1.54	1.62	0.12
Rabac1	0.28	3.28	2.89	1.02	2.38
Rabep1	1.57	0.88	1.24	0.93	1.47
Rabep2	0.74	0.16	2.24	0.33	0
Rabepk	NA	1.08	0	2.88	0.62
Rabgap1	0.54	0.34	0.59	0	2.16
Rabgap1l	1.17	0.43	1.19	1.91	0
Rabgef1	0.84	NA	3.07	0.52	NA
Rabggta	0.18	1.69	1.46	1.17	0
Rabggtb	1.06	0.83	4.51	0.27	0
Rabif	0.15	0.87	1.37	0.56	0.64
Rabl2a	0.13	NA	NA	NA	NA
Rabl3	0	0.92	0.27	1.06	0
Rabl4	0	NA	NA	NA	NA
Rabl5	1.47	1.12	0.97	2.27	0.74
Rabl6	NA	0.45	1.66	3.05	NA
Rac1	0.64	0.66	0.57	1.26	0.83
Rac2	0.93	1.16	0.99	4.05	0
Rac3	0.79	2.68	2.09	1.17	0
Racgap1	0.83	1.02	2.16	0.2	0
Rad1	0.3	0.22	1.63	2.33	0
Rad17	0	0	1.38	0.36	2.8
Rad18	1.07	NA	1.18	0.5	0.91
Rad21	4.46	0.47	4.73	0.4	1.18
Rad23a	0.18	0.83	2.14	NA	0
Rad23b	0.01	0.61	1.58	1.49	0.04
Rad50	0.62	0	1.59	0	2.04
Rad51	0.36	0.12	1.2	0.13	0
Rad51ap1	0	0.21	1.89	0.03	0.84
Rad51b	NA	1.05	0.57	0.14	NA
Rad51c	0.26	0.95	1.37	0	2.05
Rad51d	NA	0.08	0.25	1.72	NA
Rad51l1	0.41	NA	NA	NA	0
Rad51l3	0	NA	NA	NA	1.23
Rad52	1.38	1.67	0.65	0.69	0.9
Rad54b	NA	0.18	2.06	NA	0.17
Rad54l	0.31	0.41	2.36	0.26	1.88
Rad54l2	NA	0	0.49	0	0.8

Rad9a	NA	0.32	1.18	0.83	NA
Rad9b	0.22	NA	1.81	1.61	NA
Radil	NA	NA	1.29	1.45	4.51
Rae1	1	0.77	0.85	1.39	0.1
Raet1e	0.29	NA	1.8	1.39	NA
Raf1	0.29	1.37	2.01	2.16	2.72
Rag1	0	1.04	1.65	NA	0.72
Rag1ap1	NA	NA	NA	NA	0.59
Rag2	2.76	0.18	0.61	0	0
Rage	0.34	NA	NA	NA	NA
Rai1	0	1.3	0.56	1.82	0.76
Rai14	0.21	1.4	3.38	2.04	1.49
Rai16	0.67	NA	NA	NA	NA
Rai2	3.34	0.59	2.38	0	0.09
Rala	0.03	0	2.71	1.1	3.14
Ralb	0.34	0	1.54	1.16	0
Ralbp1	0.31	1.53	0.81	0.24	0.24
Ralgapa1	NA	0.3	0.81	0.89	NA
Ralgapa2	NA	NA	1.81	0	NA
Ralgapb	NA	0.39	2.64	0	NA
Ralgds	0.2	2.25	0.73	1.21	0.04
Ralgps1	0.76	0	0.57	0	0
Ralgps2	NA	3.01	0.84	0.1	0
Raly	0	0.09	0.2	NA	0.26
Ralyl	NA	0.67	0.54	2.05	3.06
Ramp1	1.61	0.85	1.01	1.9	2.4
Ramp2	2.32	0.89	2.27	1.63	2.39
Ramp3	0.82	1.85	1.4	0	NA
Ran	0.62	1.51	0.9	0.03	4.14
Ranbp1	0	1.27	1.97	0	0.11
Ranbp10	0.66	0.29	0.74	1.35	0
Ranbp17	0.8	1.6	0.16	0.02	0.78
Ranbp2	0.21	0.94	1.47	0	0
Ranbp3	NA	0.68	0.49	1.34	0.91
Ranbp3l	NA	NA	0.48	1.13	NA
Ranbp6	0	0.13	0.99	2.17	0
Ranbp9	0	0.61	1.95	1.67	NA
Rangap1	0.09	0.13	0.75	0	0.74
Rangnrf	0.34	NA	NA	NA	NA
Rangrf	NA	2.49	1.83	NA	0
Rap1a	3.78	1.3	0	0	0

Rap1b	0	1.43	3.22	0.13	NA
Rap1gap	NA	1.48	0.86	1.13	0
Rap1gap2	NA	3.06	0.63	26.37	NA
Rap1gds1	0	0.84	0.77	1.97	1.61
Rap2a	NA	0.75	2.81	1.76	0
Rap2b	0	0.53	1.2	0	0
Rap2c	0.32	1.07	3.33	1.2	NA
Rapgef1	0.39	0.81	1.45	0.42	0.89
Rapgef2	NA	0.94	2.52	14.52	0
Rapgef3	2.45	1.15	0.87	2.18	1.38
Rapgef4	0.2	1.88	0.64	1.08	0
Rapgef5	NA	0.4	2.12	1.14	2.16
Rapgef6	1.25	NA	1.3	0.3	1.58
Rapgefl1	NA	0	2.25	0.1	2.53
Raph1	NA	NA	1.31	1.2	1.39
Rapsn	1.16	2.97	3.1	0.78	2.33
Rara	0	1.17	1.89	1.81	0
Rarb	0	0.6	2.58	0.14	0
Rarg	2.04	0.85	1.4	1.07	0
Rarres1	0.37	0.59	2.57	2.17	0
Rarres2	0	1.37	0.89	NA	1.45
Rars	0.18	1.27	1.85	0.64	0
Rars2	NA	0.2	0.75	0.55	0
Rasa1	0.64	0.96	3.11	5.35	3.37
Rasa2	2.4	0.78	4.53	0.68	0
Rasa3	1.74	0	1.5	2.75	0
Rasa4	0.02	2.04	1.65	0.81	NA
Rasal1	1.57	2.87	2.72	0	0
Rasal2	NA	0.82	0.73	3.57	0
Rasal3	NA	NA	0	NA	2.16
Rasd1	3.53	NA	1.11	1.2	0.43
Rasd2	1.26	NA	1.59	0	0.22
Rasef	0.42	NA	0.94	0.71	NA
Rasgef1a	0	NA	1.39	0.61	0.26
Rasgef1b	0.01	NA	2.43	0	NA
Rasgef1c	NA	NA	1.41	0	5.56
Rasgrf1	0.32	0.55	0.51	1.24	2.41
Rasgrf2	0.7	NA	0	2.06	0
Rasgrp1	0.36	0	1.21	0	2.19
Rasgrp2	0.58	0.26	0.39	0.49	0
Rasgrp3	0	0.11	2.45	31.05	1.4

Rasgrp4	0.1	NA	0	1.79	0
Rasip1	0.92	2.44	0	1.34	0
Rasl10a	NA	2.17	0	0	0
Rasl10b	2.45	NA	2.14	1.25	3.65
Rasl11a	NA	NA	3.43	0	0
Rasl11b	1.9	1.52	0.17	0	0.27
Rasl12	1.33	0.77	1.46	0.44	0
Rassf1	0.1	1.57	0.09	NA	0
Rassf10	NA	NA	0.28	0	NA
Rassf2	0.63	0.72	3.59	2.88	0.73
Rassf3	1.33	NA	2.73	0.95	NA
Rassf4	0.3	0.61	1.98	2.26	0.87
Rassf5	0.55	NA	3.12	2.1	0
Rassf6	0.74	NA	1.23	1	0.59
Rassf7	NA	0.52	3.8	0.83	0
Rassf8	NA	0.49	2.25	1.26	1
Rassf9	NA	1.08	1.9	0.15	2.97
Raver1	NA	NA	0	0.64	2.96
Raver2	NA	NA	NA	0.67	NA
Rax	0.58	1.02	0	8.2	5.24
Rb1	0	1.21	1.47	1.91	0
Rb1cc1	0.91	1.06	0.63	0.2	0
Rbak	1.67	NA	1.86	0	4.77
Rbbp4	1.68	0.05	0	NA	0
Rbbp5	2.09	1.76	1.48	16.89	0
Rbbp6	0.55	0	1.65	0	0.77
Rbbp7	0.07	0	0.64	1	3.53
Rbbp8	NA	0.02	0.77	0.59	3.47
Rbbp8nl	NA	NA	NA	0.96	NA
Rbbp9	0.16	1.29	2.26	0.76	0.92
Rbck1	NA	3.69	2.4	0.44	0
Rbfa	NA	0	2.08	1.18	NA
Rbfox1	NA	0.76	0.48	0.9	NA
Rbfox2	NA	0.02	0.08	0	NA
Rbfox3	NA	NA	1.02	1.23	NA
Rbks	0.92	0	2.88	NA	1.56
Rbl1	0	1.34	0.69	0	3.01
Rbl2	0	0	1.75	NA	1.64
Rbm10	1.44	1.06	0.43	0.76	1.62
Rbm11	1.55	NA	0.86	0	0.57
Rbm12	0.38	0.99	0	NA	0

Rbm12b	NA	NA	NA	NA	0.49
Rbm14	0	0.48	1.62	2.63	0
Rbm15	0.03	0.34	0.1	0	0
Rbm15b	NA	0	0	1.55	0
Rbm16	0	NA	NA	NA	0
Rbm17	0.14	0	1.02	0.56	2.31
Rbm18	0.09	NA	0.98	0.55	2.11
Rbm19	0.74	2.32	1.58	0.74	0
Rbm20	NA	NA	1.11	NA	0
Rbm22	0	0.91	0	0.96	1.96
Rbm24	NA	NA	1.44	1	0.02
Rbm25	NA	0.77	1.22	0.61	0.99
Rbm26	NA	0.48	0.71	0.42	0
Rbm27	NA	NA	0.9	2.13	1.32
Rbm28	0.03	0	1.38	0.88	1.94
Rbm3	0.29	0.7	1.03	0.76	0
Rbm33	NA	NA	1.03	1.22	0
Rbm34	NA	0.98	0	0	1.49
Rbm38	NA	0.25	2.83	NA	0
Rbm39	NA	0.73	0.35	0.4	0.89
Rbm4	0.17	0.56	0.61	5.35	0
Rbm41	NA	0.31	0.47	0.39	1.07
Rbm42	NA	0.69	0.24	1.83	0.03
Rbm43	NA	NA	3.12	0	2.24
Rbm44	NA	NA	NA	0	NA
Rbm45	NA	NA	0.49	0.75	0.93
Rbm46	NA	NA	1.48	NA	NA
Rbm47	NA	0.01	2.35	0.31	0
Rbm48	NA	0.18	3.2	0.75	NA
Rbm4b	NA	0	0.68	0.44	1.42
Rbm5	0.65	0.39	1.47	0	0.16
Rbm6	0	0	0.54	1.09	1.02
Rbm7	2.03	0.93	2.33	0.71	2.38
Rbm8a	NA	1.01	1.85	0.61	1.55
Rbm9	0.74	NA	NA	NA	0
Rbms1	0.45	0	4.78	4.3	0
Rbms2	0.63	1.46	0	2.16	0
Rbms3	0.7	1.1	1.24	0.99	0.02
Rbmx	0	1.4	0.71	NA	2.61
Rbmx2	0.32	0.76	1.49	0.85	3.1
Rbmxl1	NA	NA	0.56	0.62	NA

Rbmxl2	NA	1.61	1.48	2.4	NA
Rbmy1a1	2.73	NA	NA	NA	NA
Rbp1	0.36	1.63	1.39	4.53	0
Rbp2	0.38	NA	9.4	4.26	0
Rbp3	2.71	1.11	1.04	1.98	0
Rbp4	5.9	2.38	0.92	1.15	8.76
Rbp7	0.26	NA	3.41	3.43	7.43
Rbpj	NA	0.35	0.03	0.29	0
Rbpjl	NA	0	1.56	1.38	0
Rbpms	0.59	0.78	2.07	16.63	1.75
Rbpms2	0	NA	1.23	1	1.61
Rbx1	0.65	0	1.32	0	0
Rc3h1	NA	NA	3.54	1.56	1.62
Rc3h2	NA	0.51	0.79	0.4	0.52
Rcan1	NA	0.93	1.64	0.72	1.12
Rcan2	NA	0.81	0.47	0.49	1.72
Rcan3	NA	0	1.14	NA	0
Rcbtb1	4.52	1.71	0.93	0.45	0
Rcbtb2	NA	1.06	0.88	0.18	1.99
Rcc1	NA	1.85	0.84	NA	NA
Rcc2	NA	NA	0.54	1.33	5.55
Rccd1	NA	NA	0.41	1.61	NA
Rce1	0.05	0.67	3.89	NA	2.63
Rchy1	0.06	3.32	1.27	NA	0
Rcl1	0.65	1.62	2.38	1.59	0
Rcn1	NA	0.93	0.97	0.97	4.13
Rcn2	0.6	0.77	0.21	1.06	0
Rcn3	14.38	2.03	2.3	3.08	0
Rcor1	2.08	0.52	0.27	0.2	0.61
Rcor2	NA	NA	1.2	0.19	3.22
Rcor3	0	0.75	1.4	0.59	0
Rcsd1	NA	NA	0.2	0	1.54
Rcvrn	1.08	1.24	0.74	1.64	2.49
Rd3	NA	NA	1.22	1.77	1.6
Rdbp	0.72	NA	NA	NA	0.84
Rdh10	0.26	NA	0.6	0	0
Rdh11	0.51	0.8	1.23	0	2.28
Rdh12	0.62	NA	1.05	0.74	1.12
Rdh13	1.05	NA	2.03	1.88	1.35
Rdh14	0.39	1.54	0.89	0.21	2.17
Rdh16	NA	1.32	0	0.02	2.35

Rdh5	0.75	1.81	1.15	1.23	0
Rdh8	NA	NA	NA	2.87	NA
Rdhe2	0.88	NA	NA	NA	NA
Rdm1	NA	NA	2.9	1.27	1.16
Rdx	1.35	1.5	0.92	NA	0.62
Rec8	NA	0	0.3	4.19	3.35
Rec8l1	5.11	NA	NA	NA	NA
Reck	2.83	1.26	1.49	0	5.16
Recql	0	1.17	1.53	NA	1.98
Recql4	1.47	2.32	1.31	1.18	3.14
Recql5	0.86	0.89	0.68	1.76	0.13
Reep1	NA	0.33	1.45	2.04	1.08
Reep2	NA	3.79	3.02	1.63	1.61
Reep3	NA	NA	4.34	0.93	2.05
Reep4	NA	3.02	0	5.2	0
Reep5	NA	0	3.33	0.06	0.45
Reep6	NA	NA	1.07	1.81	2.16
Reg3a	1.42	3.7	0.81	0.86	1.16
Reg3g	0	NA	1.96	0.56	2.95
Reg4	0	NA	0.94	0.8	0
Rel	0.21	0.72	2.82	0.75	0.3
Rela	0	2.46	2.56	0.48	0
Relb	0.03	1.12	2.58	5.27	0
Rell1	NA	NA	0.98	0	0
Rell2	NA	NA	1.32	NA	NA
Reln	0.77	2.68	1.51	0	1.55
Relt	NA	NA	2.28	2.11	0
Rem1	0.27	0.21	3.54	0.85	3
Rem2	1.13	NA	1.14	0.78	1.39
Renbp	0.19	2.74	3.97	1.4	0.5
Rep15	NA	NA	NA	0.55	NA
Repin1	0.39	0	0.22	1.5	2.32
Reps1	0	0	0	13.03	1.09
Reps2	0	1.95	1.16	0.88	4.09
Rer1	NA	1.24	1.81	0.64	1.88
Rere	0.03	1.42	1.51	0.92	1.13
Rerg	0.77	NA	0.94	2.12	1.99
Rergl	NA	NA	NA	1.55	NA
Resp18	0	NA	NA	0.89	NA
Rest	2.3	0.42	1.46	2.56	0
Ret	2.94	0.6	0.1	1.88	0

Retn	1.44	0	1.57	0.48	0
Retnlb	0.61	NA	0.55	2.08	0
Retsat	NA	0	1.74	1.34	3.4
Rev1	NA	0	0.95	NA	1.73
Rev3l	1	0.11	0.98	0.49	0
Rexo1	NA	NA	0.75	2.05	0
Rexo2	NA	1.41	1.07	0.13	0
Rexo4	NA	0	1.4	1.2	0.09
Rfc1	NA	0	1.55	0.79	0
Rfc2	0.23	1.2	2.58	1.06	0
Rfc3	0	2.06	2.05	0.42	4.7
Rfc4	0	1.65	1.38	2.2	3.58
Rfc5	0.4	0	3.02	0.6	0
Rfesd	NA	NA	0.39	0.35	1.58
Rffl	NA	NA	0.31	0	0
Rfk	1.22	2.31	1.54	1.58	0
Rfng	0.01	1.86	2.14	2.14	0.71
Rfpl4b	NA	NA	NA	3.46	NA
Rft1	NA	NA	NA	0.09	NA
Rftn1	NA	0	1.92	0	0
Rftn2	NA	NA	0	0.76	1.28
Rfwd2	NA	NA	2.54	0.8	2.6
Rfwd3	NA	0.05	2.33	0	1.52
Rfx1	0.49	2.46	0.86	0	0.02
Rfx2	1.14	2.04	0.73	1.19	2.57
Rfx3	0	1.19	0.7	0.51	0
Rfx4	0.11	NA	1.93	0.12	2.63
Rfx5	0.54	0	2.02	0.7	1.59
Rfx6	NA	NA	NA	3.75	NA
Rfx7	NA	2.11	0.76	2.18	0
Rfx8	NA	NA	NA	1.63	NA
Rfxank	7.04	2.46	1.54	NA	2.13
Rfxap	0	1.16	0	0.97	0
Rfxdc1	0.07	NA	NA	NA	NA
Rg9mtd1	0.62	NA	NA	NA	0
Rg9mtd2	0.81	NA	NA	NA	2.21
Rg9mtd3	0.19	NA	NA	NA	1.3
Rgag1	NA	NA	NA	0.85	NA
Rgag4	NA	NA	3.59	1.55	2.59
Rgcc	NA	1.59	4.97	2.42	NA
Rgl1	0.56	0	3.99	0.72	0

Rgl2	0	4.37	4.85	1.4	1.48
Rgl3	NA	NA	0.75	1.49	6.83
Rgma	3.34	NA	0	1.31	0
Rgmb	0	NA	0.99	4.47	0.18
Rgn	0.96	2.01	0.84	0	5.76
Rgnef	0.45	NA	NA	NA	1.72
Rgp1	NA	0.84	4.06	NA	0
Rgr	0	0	1.69	3.73	0
Rgs1	1.1	0.87	2.63	0.12	0
Rgs10	0.49	1.3	2.9	2.83	0
Rgs11	0	2	0.97	2.79	0
Rgs12	0.88	0.76	0.55	0.81	0
Rgs13	0	1.1	3.91	16.04	0.19
Rgs14	0.84	1.24	0.55	0.94	0
Rgs16	0.06	0.89	2.04	1.63	1.73
Rgs17	1.48	1.65	2.65	0.35	0.91
Rgs18	0	NA	0.85	1.98	0.33
Rgs19	0	0	1.6	1.83	0.63
Rgs2	1.53	1.6	1.41	0	1.05
Rgs20	0	1.07	0.65	0.96	0
Rgs21	NA	NA	NA	4.96	NA
Rgs22	NA	NA	NA	0.97	NA
Rgs3	0.41	0.69	0.72	1.03	0.5
Rgs4	0.1	0.42	0.12	1.93	2.36
Rgs5	0.51	0.9	0.53	0.51	1.97
Rgs6	1.44	0.76	0.61	3.34	0
Rgs7	0	0.05	1.06	0	3
Rgs7bp	NA	NA	1.56	2.19	1.06
Rgs8	NA	NA	3.16	2.73	8.15
Rgs9	0	0	0.27	1.05	0.9
Rgs9bp	0.25	NA	0.39	1.8	NA
Rgsl2	1.96	NA	NA	NA	NA
Rhag	1.47	1.16	1.32	0.94	1
Rhbdd1	NA	NA	0.7	1.21	1.63
Rhbdd2	NA	NA	0	1.18	0.95
Rhbdd3	NA	1.88	1.64	2.18	0
Rhbdf1	2.31	3.2	0.48	1.05	3.25
Rhbdf2	NA	0	1.01	2.44	0
Rhbdl1	NA	2.95	0.55	1.72	14.29
Rhbdl2	NA	NA	0.31	0	NA
Rhbdl3	NA	NA	0.72	1.37	0

Rhbg	1.2	0.05	1.82	0.54	14.86
Rhcg	1.17	2.65	0.8	0	5.05
Rhd	NA	2.44	0.04	2.74	1.6
Rheb	1.7	0.37	2.36	1.02	NA
Rhebl1	1.62	NA	0	0.43	NA
Rhno1	NA	NA	0.15	1.34	NA
Rho	18.48	0.79	0.01	2.06	0
Rhoa	0	1.26	0.12	0	0
Rhob	0.35	2.14	2.84	0.19	0
Rhobtb1	2.1	2.13	0.59	0	0
Rhobtb2	0.59	0	1.41	2.85	1.14
Rhobtb3	0.04	0.58	0.07	0.77	0.01
Rhoc	0.73	2.33	1.3	0	0.37
Rhod	NA	1.16	2.51	1.41	0
Rhof	0.39	0	3.86	NA	0
Rhog	1.66	0.21	2.15	7.5	0
Rhoh	1.38	0	1.21	0.2	0
Rhoj	0	NA	1.39	2.81	1.86
Rhoq	3.16	1.26	1.26	0.51	0.06
Rhot1	0.62	1.28	1.14	1.44	0.52
Rhot2	0.97	0	5.5	2.79	0
Rhou	1.39	NA	2.92	1.45	1.71
Rhov	0.61	NA	3.86	1.55	0
Rhpn1	0	NA	1.94	0	0
Rhpn2	0.76	NA	2.5	1.21	0.1
Ribc1	0.89	NA	0.53	1.79	0
Ribc2	0.9	0.95	1.04	0.57	0
Ric3	NA	0	1.06	2.03	1.19
Ric8b	NA	0	0.44	0.75	1.37
Rictor	NA	NA	0.77	1.02	0.59
Rif1	NA	1.25	1.28	0.27	0
Riiad1	NA	NA	1.91	0	NA
Rilp	NA	NA	1.66	1.87	1.32
Rilpl1	NA	NA	0.36	0	0.39
Rilpl2	NA	NA	2.17	0.59	0
Rimbp2	NA	0.73	0.69	0.7	3.78
Rimbp3	NA	NA	NA	1.31	NA
Rimkla	NA	NA	1.11	0.87	0
Rimklb	NA	NA	0.75	1.5	0
Rims1	0	0.07	0.27	1.1	1.74
Rims2	0.75	0.59	0.9	0	1.76

Rims3	0.94	0.41	0.97	0	0.53
Rims4	0.97	NA	1.27	1.45	1.07
Rin1	0.82	1.46	1.01	2.49	0
Rin2	0	2.07	4.17	0	NA
Rin3	0.77	1.49	3.01	1.52	0
Ring1	0.57	0	0.22	1.04	0
Rinl	NA	NA	NA	5.93	NA
Rint1	NA	0.08	0.36	0.82	0
Riok1	0.7	NA	1.5	1.04	1.47
Riok2	1.22	0.81	2.3	0.67	2.65
Riok3	0.72	0.97	1.51	1.98	0
Ripk1	1.45	1.69	1.17	0.47	0
Ripk2	0	0.57	3.98	0.82	4.64
Ripk3	0.23	NA	1.89	1.34	0.44
Ripk4	3.25	0.53	2.45	0.75	0
Ripk5	1.44	NA	NA	NA	NA
Ripply1	NA	NA	NA	0.74	NA
Ripply2	NA	NA	0.54	0.75	2.84
Ripply3	NA	0	1.92	2.84	NA
Rit1	0.1	4.27	0.6	2.58	0.71
Rit2	0.17	0.68	0.75	0.34	0
Rkhd1	0	NA	NA	NA	NA
Rkhd2	1.34	NA	NA	NA	NA
Rkhd3	1.32	NA	NA	NA	NA
Rlbp1	1.24	0.72	1.04	0.71	0
Rlf	NA	0.93	0.52	1.56	0
Rlim	NA	1.56	0.43	0.72	0
Rln1	1.65	5.16	1.25	2.12	1.52
Rln3	1.03	NA	NA	3.46	NA
Rltpr	NA	NA	0.7	1.13	0.82
Rmdn1	NA	0.55	0.79	1.61	NA
Rmdn2	NA	NA	1.26	0.87	NA
Rmdn3	NA	2.29	0.05	0.57	NA
Rmi1	NA	2.25	1.34	0.02	0
Rmi2	NA	NA	0	0.07	NA
Rmnd1	NA	2.41	1.95	NA	0
Rmnd5a	NA	0.36	0.38	0.1	0
Rmnd5b	NA	1.21	1.64	0.54	2.05
Rmrp	NA	NA	NA	0	NA
Rmst	NA	NA	1.07	NA	2.79
Rn7sk	NA	NA	NA	0	NA

Rnase1	3.84	0.99	0	0.85	0
Rnase10	NA	NA	NA	0.94	NA
Rnase12	NA	NA	NA	0.38	NA
Rnase13	NA	NA	NA	3.33	NA
Rnase2	0	NA	NA	NA	NA
Rnase4	0.74	0.34	2.34	0.36	0.48
Rnase6	0.1	0.62	1.21	3.11	0
Rnase9	6.86	NA	NA	0.74	NA
Rnaseh1	0.46	1.13	0.54	0.53	0
Rnaseh2a	0.14	0	2	0.67	0
Rnaseh2b	NA	2.14	0.68	0.98	0.03
Rnaseh2c	NA	NA	2.21	0	1.21
Rnasek	NA	NA	1.55	0	1.49
Rnasel	0	0	2.01	0.51	0
Rnasen	NA	NA	NA	NA	0
Rnaset2	1.06	NA	NA	NA	NA
Rnd1	0	1.26	1.53	1.45	0
Rnd2	NA	2.31	1.64	0.59	1.68
Rnd3	NA	0.34	1.76	0.26	5.71
Rnf10	0	0.39	0.69	0	0
Rnf103	0.71	0.11	0	0	0
Rnf11	0	1.47	2.07	0.57	1.22
Rnf111	1.02	0.65	2.05	0.78	0.61
Rnf112	NA	NA	0.03	0.31	0.56
Rnf114	NA	0.76	0.09	0	1.24
Rnf115	NA	0.96	1	0	2.77
Rnf12	2.38	NA	NA	NA	NA
Rnf121	0.54	1.83	0.36	6.3	2.69
Rnf122	1.6	3.11	2.11	0.11	1.14
Rnf123	0.61	0.59	0.33	1.56	1.59
Rnf125	0.78	1.39	0.19	1.39	6.22
Rnf126	0.02	0.22	4.39	1.6	NA
Rnf128	0	1.56	3.62	0.15	0.64
Rnf13	1.68	1.3	1.25	0.73	0
Rnf130	0.21	0	1.24	1.24	0.4
Rnf133	2.57	NA	0	1.85	0
Rnf135	NA	NA	3.01	1.19	0
Rnf138	0.22	2.77	1.57	0.53	1.49
Rnf139	1.41	1.3	1.39	0	2.3
Rnf14	0.84	2.35	0.41	1.56	2.44
Rnf141	0.31	0.84	0	0.88	2.12

Rnf144	2.19	NA	NA	NA	NA
Rnf144a	NA	1.01	0.94	0	0
Rnf144b	NA	NA	1.95	0	1.93
Rnf145	NA	NA	1.7	0.89	1.3
Rnf146	0	1.78	2.1	1.65	0.59
Rnf148	0.15	NA	3.72	1.8	6.44
Rnf149	1.02	NA	1.99	0	0
Rnf150	1.07	NA	1.18	1.95	1.74
Rnf151	3.21	NA	1.89	0	0
Rnf152	NA	NA	0.8	1.63	NA
Rnf157	2.13	NA	3.45	2.3	1.74
Rnf160	NA	NA	NA	NA	0
Rnf165	NA	NA	0.06	1.83	0.91
Rnf166	0	NA	1.58	1.32	0
Rnf167	NA	1.97	2.51	1.1	0
Rnf168	NA	NA	0.9	0.63	NA
Rnf169	NA	NA	2.74	0	4.36
Rnf17	0	0.62	0.41	1.52	0
Rnf170	NA	1.46	1.05	0.78	0
Rnf180	NA	NA	1.15	3.47	0
Rnf181	NA	NA	0.98	1.26	0.17
Rnf182	NA	NA	0	1.57	1.01
Rnf183	NA	NA	0	0.07	2.79
Rnf185	NA	0.35	1.55	0.98	0
Rnf186	NA	0.86	8.38	0	1.42
Rnf187	NA	0.14	1.48	2.59	0.86
Rnf19	0.38	NA	NA	NA	NA
Rnf19a	NA	1.55	2.77	0	5.26
Rnf19b	NA	1.54	2.14	0.92	0
Rnf2	9.5	2.12	0.52	0.36	0
Rnf20	1.5	NA	0	0.33	0
Rnf207	NA	NA	1.31	0.35	2.81
Rnf208	NA	1.84	0	0.33	5.09
Rnf212	NA	NA	NA	0.56	NA
Rnf213	NA	NA	0.75	0	0.19
Rnf214	NA	NA	1.33	0.89	0
Rnf215	NA	NA	0.6	0.89	1.48
Rnf216	NA	0	1.26	0	2.02
Rnf217	NA	NA	1.15	0	1.71
Rnf219	NA	1.17	0.63	0.19	1.23
Rnf220	NA	0.95	0.37	7.32	0

Rnf222	NA	NA	NA	1.17	NA
Rnf24	0	0	0.83	0	0
Rnf25	0.5	1.43	1.77	0.51	4.92
Rnf26	0.13	NA	1.92	0.55	0
Rnf31	0.06	2.6	1.83	1.31	0
Rnf32	0.77	2.04	1.78	1.36	1.88
Rnf34	0.27	1.73	1.43	0	2.32
Rnf38	0	0	2.27	0.68	1.37
Rnf39	NA	1.19	0.01	1.78	0
Rnf4	0.75	1.92	0.59	0.63	4.26
Rnf40	0.11	1.41	1.54	0.43	0.86
Rnf41	1.93	0.72	0.09	0	1.29
Rnf43	NA	1.14	1.23	2.95	0
Rnf44	0.18	0	1.14	1.52	5.74
Rnf5	NA	0	1.93	NA	0.15
Rnf6	0.32	0.81	0.07	0.77	1.14
Rnf7	0.4	0	1.69	2.49	1.14
Rnf8	1.76	1	1.44	0.89	NA
Rnft1	NA	2.53	4.55	0.66	0
Rnft2	NA	4.9	2.26	0	1.32
Rngtt	0	0.5	0.74	0.62	0.12
Rnh1	0	2.77	0	NA	0
Rnls	NA	1.85	0.5	4.45	1.76
Rnmt	0	0.05	1.01	0.91	0
Rnmtl1	NA	0.33	1.24	0.76	0
Rnpc2	0.68	NA	NA	NA	NA
Rnpc3	NA	NA	0.59	1.8	0
Rnpep	0.89	3.93	5.36	0	0
Rnpepl1	0.93	0.64	1.3	0.9	0.08
Rnps1	0.15	2.09	1.17	0.04	0
Rnu12	NA	NA	NA	0	NA
Robld3	NA	NA	NA	NA	0
Robo1	0.4	1.99	0	0	0.98
Robo2	1.65	NA	0.45	0	3.78
Robo3	4.14	2.02	2.01	0.27	0.66
Robo4	0	2.56	1.99	1.61	0
Rock1	0.93	0.38	1.35	1.3	0.69
Rock2	1.6	2.66	0.96	0	0.77
Rod1	0.6	NA	NA	NA	1.14
Rogdi	NA	1.26	1.79	0	3.36
Rom1	2.69	3.88	1.4	0.43	0

Romo1	NA	NA	3.02	0	3.03
Ropn1	0	NA	0.04	NA	0.39
Ropn1l	2.66	NA	0.61	2.97	0
Ror1	0.33	0.33	0.71	0.6	1.46
Ror2	1.55	0.46	3.3	1.4	2.88
Rora	0.3	0.52	0.53	3.66	0
Rorb	0	1.65	0.98	0	0
Rorc	1.57	0	1.54	0.2	0
Ros1	0.26	1.86	3.47	4.16	0
Rp1	NA	NA	0.01	0.93	2.33
Rp1l1	NA	NA	0.3	0.28	NA
Rp9	NA	NA	2.2	1.12	0
Rpa1	0.3	0	1.38	1.51	0
Rpa2	0.85	0.51	3.7	1.59	1.58
Rpa3	0.17	2.31	1.55	1.44	7.75
Rpain	NA	0.35	1.87	5.46	0
Rpap1	0	0	2.04	2.57	0
Rpap2	NA	0.45	1.27	0.22	1.37
Rpap3	NA	1.58	4.77	0.62	0
Rpe	1.84	0.1	0.03	0.64	5.17
Rpe65	0.59	2.27	5.15	0	0
Rpf1	NA	1.81	1.09	1.14	NA
Rpf2	NA	NA	2.41	0.85	NA
Rpgr	0.21	1.1	1.01	0.02	0
Rpgrip1	0	0	0	NA	0.77
Rpgrip1l	NA	NA	NA	0	NA
Rph3a	3.37	0.61	0	1.76	1.68
Rph3al	2.27	2.32	1.42	1.14	1.23
Rpia	0.29	0.49	2.74	0.98	0
Rpl10	1.03	0.22	0.13	1.97	0
Rpl10a	0.36	0	2.28	1.89	NA
Rpl10l	NA	NA	NA	8.28	NA
Rpl11	0.29	1.04	2	0	0
Rpl12	0.63	0	1.31	0	3.3
Rpl13	0	0	2.92	0.75	0
Rpl13a	0.44	0	1.58	NA	3.04
Rpl14	0	2.56	1.93	0.04	0
Rpl15	0.27	0.61	0.98	0	0
Rpl17	0	0.4	0.97	0.47	0
Rpl18	NA	0	1.15	0	0
Rpl18a	NA	NA	NA	0	NA

Rpl19	0.6	0	4.59	0	0
Rpl21	NA	0.87	1.98	NA	2.12
Rpl22	0.51	0	2.15	0	0
Rpl22l1	NA	NA	1.12	NA	0
Rpl23	0.89	0	0.85	NA	1.98
Rpl23a	0.17	1.27	1.43	0	0
Rpl24	NA	1.09	2.65	NA	NA
Rpl26	0.27	1.74	2.87	0	0
Rpl27	0	2.05	0.97	0	NA
Rpl27a	0.54	2.28	0.92	0.94	0
Rpl28	0.69	0	1.6	0	NA
Rpl29	0.34	NA	NA	0.55	NA
Rpl3	0.21	0.96	1.06	1.66	0
Rpl30	1.16	NA	NA	0	NA
Rpl31	0.95	0.93	0.87	0	5.24
Rpl32	0	0	2.3	NA	7.39
Rpl34	NA	1.12	3.51	0	0
Rpl35	NA	0.77	0.97	0	3.78
Rpl35a	1.31	NA	NA	NA	NA
Rpl36	0.41	0	2.46	0.11	NA
Rpl36a	0.86	0.92	2.26	NA	8.96
Rpl36al	0.22	0.85	1.39	NA	0
Rpl37	0.41	0	1.33	NA	1.51
Rpl37a	0.02	0	1.22	NA	0
Rpl38	0.28	0.72	1.93	0	NA
Rpl39	0	NA	NA	NA	0
Rpl39l	NA	0.59	2.39	3.44	0.14
Rpl3l	1.21	0.69	3.94	0.59	18.68
Rpl4	0.05	0.05	0.28	1.08	0
Rpl41	0.57	1.6	0.63	0	0
Rpl5	0.04	0	2.24	0	NA
Rpl6	0	1.05	1.5	1.14	0
Rpl7	0.82	1.45	1.01	0	0
Rpl7a	0.09	1.95	1.01	0	3.33
Rpl7l1	NA	NA	2.82	0.87	0
Rpl8	0.41	0	0.99	0.76	0
Rpl9	0	0.95	3.26	0.07	0
Rplp0	0	0.81	1.49	0.17	0
Rplp1	NA	1.85	2.46	0.45	5.45
Rplp2	0.63	0.28	0.91	1.38	0
Rpn1	0.23	2.05	1.46	0	1.84

Rpn2	NA	1.55	2.52	3.2	0
Rpp14	0.85	1.76	0.53	1.6	0
Rpp21	0.36	NA	NA	NA	NA
Rpp25	0	1.18	1.39	1.82	1.44
Rpp25l	NA	NA	0.59	3.82	NA
Rpp30	0.34	0.9	1.53	0.72	0
Rpp38	0	NA	NA	1.01	NA
Rpp40	2.3	0.89	0.09	2.2	0.76
Rpph1	0.84	NA	NA	NA	NA
Rprd1a	NA	1.34	1.34	0.69	0
Rprd1b	NA	NA	0.58	1.09	0
Rprd2	NA	0	0.44	0	0.04
Rprm	NA	2.08	0.41	0.31	2.71
Rprml	NA	NA	0.22	2.25	NA
Rps10	0.51	1.6	2.01	0	0
Rps11	0.54	0.75	1.06	0	0
Rps12	0.63	1.46	1.74	NA	0
Rps13	0	1.68	2.62	0.04	NA
Rps14	0.52	0	0.66	0.18	0
Rps15	0	0	1.11	0.19	0
Rps15a	0	1.09	0.56	0	0
Rps16	0.71	0	0.22	NA	0
Rps17	0.43	1.06	1.83	NA	0
Rps18	1.62	0	1.71	0	3.45
Rps19	0.67	0	0.97	0.14	0
Rps19bp1	NA	NA	2.03	1.37	0
Rps2	NA	1.68	1.32	0	0
Rps20	0.9	1.75	3.36	0	0
Rps21	0.43	1.05	0.48	0.63	0
Rps23	0.17	0	1.04	0.47	NA
Rps24	0.06	1.03	2.53	0.16	0
Rps25	0.68	2.86	0.83	0	1.57
Rps26	0.45	1.5	3.42	0	0
Rps27	0.62	1.17	1.1	NA	0.8
Rps27a	0.42	4.31	2.18	0	NA
Rps27l	0.14	0	1.09	0	0.16
Rps28	0.56	0.92	3.93	0.51	NA
Rps29	1.02	2.38	1.65	1.97	NA
Rps3	0.1	1.25	0.47	0	0
Rps4x	0.52	0	0.72	0	7.05
Rps4y2	NA	NA	NA	0.62	NA

Rps5	0.29	0	0.94	0	0
Rps6	0.2	0.7	1.01	0	0
Rps6ka1	0.04	0.76	2.96	0	0
Rps6ka2	0	1.84	0.67	0.71	0.47
Rps6ka3	0.37	0.5	2.45	0.37	1.33
Rps6ka4	0	2.41	0	1.22	1.37
Rps6ka5	0.13	0	2.05	0.28	0
Rps6ka6	1.72	1.36	1.02	1.13	0
Rps6kb1	0.39	0.6	0	0.78	1.36
Rps6kb2	0.16	2.79	1.01	0.34	1.78
Rps6kc1	1.77	3.15	3.25	0	0
Rps6kl1	1.21	NA	0.58	0.99	5.94
Rps7	0.27	2.23	1.11	0	0
Rps8	0.45	0	1.81	0.07	0
Rps9	NA	0	0.77	0.56	0
Rpsa	NA	0	0.11	NA	0
Rptn	0.61	NA	0.43	0.43	NA
Rptor	NA	NA	0.78	1.09	0
Rpusd1	NA	NA	1.06	0.37	0
Rpusd2	NA	0	0	4.64	0.64
Rpusd3	NA	NA	0.41	0	0
Rpusd4	NA	NA	2.44	1.36	0
Rqcd1	1.22	1.02	2.39	0.84	0
Rrad	0.32	1.27	2.78	2.01	0
Rraga	4.15	4.22	1.68	1.96	4.71
Rragb	NA	NA	NA	1.92	NA
Rragc	0	0.34	0.9	0.6	0
Rragd	0.45	0	0.19	0.63	0
Rras	0.32	0	2.23	0.7	0
Rras2	0.58	0	3.29	0.38	0
Rrbp1	0.15	0.45	0.58	0	0
Rreb1	NA	0.53	0.52	12.55	0.16
Rrh	1.94	0.93	0.3	0.15	0.06
Rrm1	0.29	0.67	1.65	0.18	0
Rrm2	1.49	0.89	2.18	0.51	0.18
Rrm2b	1.99	NA	1.61	0.91	0
Rrn3	0.38	2.63	1.56	0.78	0
Rrnad1	NA	4.15	2.7	0.54	NA
Rrp1	NA	2.83	0.33	2.7	1.05
Rrp12	NA	1.32	0.98	2.68	1.4
Rrp15	NA	2.02	0.43	1.46	4.56

Rrp1b	NA	1.56	1.08	0.8	1.29
Rrp36	NA	NA	2.26	1.05	NA
Rrp7a	NA	1.15	1.01	NA	1.72
Rrp8	NA	0.26	4.77	1.1	0.05
Rrp9	NA	1.65	0.41	1.66	0.53
Rrs1	0.85	2.85	1.41	2.7	0
Rs1	NA	0.2	0.42	0.98	2.65
Rsad1	NA	0	0.64	0.31	3.06
Rsad2	NA	1.51	1.31	0.11	2.01
Rsbn1	0.4	2.33	1.11	0	NA
Rsbn1l	0.92	NA	NA	0.35	NA
Rsf1	NA	0.89	1.69	1.04	0.96
Rsg1	NA	1.44	0.23	0.97	NA
Rshl1	7.71	NA	NA	NA	NA
Rshl2	0.39	NA	NA	NA	NA
Rshl3	1.64	NA	NA	NA	NA
Rsl1d1	NA	1.04	1.09	1.95	0
Rsl24d1	NA	0	1.94	0.42	0
Rsph1	NA	NA	1.53	2.09	0.88
Rsph4a	NA	NA	0.26	2.14	0
Rsph6a	NA	1.87	1.64	0.94	NA
Rsph9	NA	NA	1.25	0.94	3.87
Rspo1	NA	NA	0.36	1.45	13.14
Rspo2	NA	NA	1.04	1.12	NA
Rspo3	NA	NA	0.89	0.42	0
Rspo4	NA	NA	1.87	1.16	0.61
Rspry1	NA	NA	0.95	0.85	3.16
Rsrc1	NA	1.68	0.78	0.95	0.94
Rsrc2	NA	0.41	1.04	0.57	1.19
Rsu1	0	0.53	0.05	1.34	0.61
Rtbdn	NA	NA	1.97	0.76	0.29
Rtca	NA	0	0	1.24	NA
Rtcd1	0.43	NA	NA	NA	3.53
Rtdr1	0.24	1.27	1.21	3.32	3.53
Rtel1	NA	1.22	1.19	1.77	0
Rtf1	NA	0.58	1.34	0.53	0.86
Rtfdc1	NA	0.05	0.81	1.45	NA
Rtkn	0.91	NA	1.14	0	0
Rtkn2	NA	NA	0.41	0.65	3.62
Rtl1	0	NA	NA	NA	NA
Rtn1	0	0	2.15	1.45	7.34

Rtn2	2.43	1.92	1.57	2.67	7.16
Rtn3	0.56	3.14	1.07	4.18	0
Rtn4	0.23	0.72	0.72	0.22	0
Rtn4ip1	0	NA	0	1.04	0
Rtn4r	1.37	NA	0	0.78	0
Rtn4rl1	0	NA	0.83	1.2	2.36
Rtn4rl2	2.83	NA	0.94	1.3	3.81
Rtp1	NA	NA	NA	0.74	NA
Rtp2	NA	NA	NA	0.82	NA
Rtp3	NA	NA	1.28	1.51	NA
Rtp4	NA	0.38	1.51	1.93	0.47
Rttn	0.47	NA	1.14	0	0
Rufy1	1.5	0.14	1.53	0.42	0.11
Rufy2	0.66	1.1	0	0.56	0.91
Rufy3	NA	0.19	1	1.42	0
Rufy4	NA	NA	NA	3.14	NA
Rundc1	0	NA	1.54	0.64	0
Rundc3a	NA	0.56	0.76	1.09	2.2
Rundc3b	NA	1.7	1.4	0.85	0.75
Runx1	0.09	0.91	0	3.35	0
Runx1t1	NA	0.85	0.67	0	0.76
Runx2	2.75	0.24	0.49	2.12	0
Runx3	0.86	0	1.44	0.62	0
Rusc1	0.41	0.17	0.86	0.12	0.74
Rusc2	0.22	1.29	1.37	0.94	1.29
Rutbc1	6.23	NA	NA	NA	NA
Rutbc2	0.5	NA	NA	NA	NA
Rutbc3	0	NA	NA	NA	NA
Ruvbl1	0	2.8	0.39	0.66	9.07
Ruvbl2	0.4	2.04	0.64	0.9	3.03
Rwdd1	1.03	0	0.63	0	2.93
Rwdd2	0.87	NA	NA	NA	NA
Rwdd2a	NA	1.92	2.02	0.35	2.97
Rwdd2b	NA	3.1	0.44	1.22	2.22
Rwdd3	7.91	0.87	0.97	0.45	2.83
Rwdd4a	NA	NA	NA	NA	0
Rxfp1	NA	NA	0.66	3.96	0
Rxfp2	NA	NA	1.71	0.76	NA
Rxfp3	NA	NA	NA	2.32	NA
Rxfp4	NA	NA	NA	1.36	NA
Rxra	0.99	0.84	1.12	2.2	0.59

Rxrb	0.02	0.59	0	1.27	1.74
Rxrg	0.18	3.03	0	1.48	2.6
Rybp	1.29	0.79	0.9	0.22	0.44
Ryk	0.25	0.08	0.11	1.09	1.81
Ryr1	0.68	0.67	0.5	0.52	2.9
Ryr2	0.31	0	0.44	1.15	0
Ryr3	0.48	0.21	0.95	0	1.6
S100a1	2.93	2.83	2.35	1.49	0
S100a10	0.31	0.49	0	0	0
S100a11	0.36	0.6	1.99	0.02	0
S100a13	0.03	0.28	4.05	0.47	1.87
S100a14	1.05	1.41	0.21	0.12	1.34
S100a16	0.53	NA	1.47	1.09	1.01
S100a2	NA	NA	NA	0.86	NA
S100a3	0.65	1.7	0.14	3.18	6.41
S100a4	0.26	2.37	1.4	2.28	5.05
S100a5	1.8	3.18	0.4	0	0
S100a6	0.49	0.11	3.37	0.44	0
S100a7a	NA	NA	NA	1.25	NA
S100a8	0.52	0.58	5.83	0.7	1.24
S100a9	0.44	0.1	5.02	0	0
S100b	0	1.66	1.2	1	0.81
S100g	2.38	2.6	0.64	1.25	2.47
S100pbp	NA	0.1	1.4	0.7	0
S100z	NA	NA	NA	0.92	NA
S1pr1	NA	0.07	4.72	1.44	2.52
S1pr2	NA	0.24	1.37	1.94	0.76
S1pr3	NA	NA	0.51	NA	0.88
S1pr4	NA	3.78	1.76	1.15	1.49
S1pr5	NA	0.05	0.75	1.4	0
Saa1	0	NA	NA	NA	NA
Saa2	0.4	0.74	1.19	0.6	NA
Saa4	0.78	0.74	0.98	1.51	3.5
Saal1	NA	NA	0.3	0.72	3.2
Sac3d1	NA	1.26	0.09	3.74	0
Sacm1l	2.15	0.25	1.2	0.51	0
Sacs	NA	0.21	1.27	0	0
Sae1	NA	0	1.01	0.87	0.68
Safb	NA	0.2	0.51	1.64	0.31
Safb2	NA	0.02	1.77	3.5	2.61
Sag	0	2.65	0.68	0.93	0

Sall1	0.97	0.99	3.03	1.23	0
Sall2	0	3.41	2.34	2.78	0
Sall3	0.33	NA	0	1.87	0
Sall4	0.69	NA	0.64	0.21	2.54
Samd1	NA	NA	0	0.16	NA
Samd10	2.03	NA	1.82	0.91	0.23
Samd11	1.13	NA	0.71	NA	NA
Samd12	NA	NA	0.02	1.5	3.25
Samd14	NA	3.61	1.07	0.97	1.94
Samd3	NA	NA	NA	0	NA
Samd4b	NA	2.25	0.7	1.4	0
Samd5	NA	NA	0.49	1.03	1.75
Samd7	1.15	NA	NA	2.88	NA
Samd8	0.61	NA	1.23	6.92	2.68
Samd9l	NA	NA	1.71	0	0.2
Samhd1	0.39	0.8	0.37	NA	0
Samm50	NA	0.15	1.03	0.21	0.05
Samsn1	0.06	0	0.72	1.96	0
Sap130	NA	0	1.01	0.78	0
Sap18	0	1.78	1.56	0	0
Sap25	NA	NA	NA	1.5	NA
Sap30	0.56	0.09	0.84	0.96	1.77
Sap30bp	NA	0.78	0.91	0.64	0.18
Sap30l	NA	2.81	1.09	1.17	NA
Sapcd1	NA	0	1.28	NA	NA
Sapcd2	NA	NA	1.73	1.43	NA
Saps1	NA	NA	NA	NA	5.7
Saps2	NA	NA	NA	NA	1.52
Saps3	NA	NA	NA	NA	0.18
Sar1a	NA	0.63	1.35	0.38	1.1
Sar1b	NA	1.73	1.51	0.62	0.83
Sardh	1.4	2.05	1.54	1.14	1.63
Sarnp	NA	NA	1.88	1.08	NA
Sars	NA	0.08	1.02	0.5	0
Sars2	NA	0	0.83	1.01	3.41
Sart1	1.45	0.29	0.1	NA	1.03
Sart3	0.99	0	1.06	0.93	4.62
Sash1	1.73	1.07	2.19	0.81	0.78
Sash3	NA	1.06	0.65	0.63	1.08
Sass6	NA	NA	1.14	NA	0
Sat1	0	0	1.47	0.94	0

Sat2	0	NA	0	6.38	0
Satb1	0.37	0	1.44	2.17	2.62
Satb2	1.03	1.86	0.55	0.83	1.78
Satl1	NA	NA	NA	3.38	NA
Sav1	0	1.1	0.22	0.59	NA
Saysd1	NA	0.14	1.32	1.45	NA
Sbds	1.38	NA	NA	1.12	NA
Sbf1	0.53	0	0.42	1.06	0.78
Sbf2	NA	NA	1.44	0.64	1.13
Sbk1	NA	NA	1.7	0.95	0
Sbk2	NA	NA	NA	0.44	NA
Sbno1	2.07	0	0.52	0	0.41
Sbno2	NA	0.53	1.8	0.44	0
Sbsn	0.75	NA	0.97	3.76	0
Sbspon	NA	1.07	0.94	1.2	NA
Sc4mol	0.56	NA	NA	NA	1.22
Sc5d	NA	1.36	0.57	0.71	NA
Scaf1	NA	NA	0	0.77	0.13
Scaf11	NA	0.85	0.36	0.01	NA
Scaf4	NA	1	0.12	0.9	NA
Scaf8	NA	0	3	0	NA
Scai	NA	0.65	2.58	0	0
Scamp1	0.81	0.99	1.45	0	2.04
Scamp2	0	1.64	0.82	0.79	3.01
Scamp3	0.57	0	1.01	0.61	0
Scamp4	0.05	NA	NA	NA	NA
Scamp5	0	0.27	0.81	1.71	0
Scand1	0.58	0	0.75	0.4	2.23
Scand3	NA	NA	NA	NA	0.36
Scap	0.58	0	1.5	1.22	0
Scaper	NA	1.08	1.85	0	0
Scara3	1.14	0.87	0.23	2.17	2.37
Scara5	NA	NA	0.72	1.61	1.58
Scarb1	0	1.18	1.92	2.43	4.28
Scarb2	0.5	0.04	2.4	7.19	0.57
Scarf1	NA	0.93	1.67	0.87	0
Scarf2	1.02	NA	0.57	0.46	0.73
Scarna13	NA	NA	NA	0.39	NA
Scarna17	NA	NA	NA	0	NA
Scarna8	NA	NA	NA	0	NA
Sccpdh	NA	0	1.35	0.16	7.19

Scel	1.79	0.27	1.76	0.8	1.55
Scfd1	0.68	0.08	1.52	0.97	1
Scfd2	0	NA	0.95	0.06	2.07
Scg2	0.22	0.77	0.44	2.78	0
Scg3	0.02	2.9	12.18	7.5	0
Scg5	NA	0.87	5.08	0.18	0
Scgb1a1	0.99	1.19	0	1.25	0
Scgb2b2	NA	NA	NA	0	NA
Scgb3a1	0.16	NA	0.61	1.53	3.92
Scgb3a2	0.8	NA	0.71	1.85	0.43
Scgn	0.34	0.72	0.96	0	6.15
Schip1	0.67	NA	NA	NA	0
Scimp	NA	NA	NA	0.7	NA
Scin	0	2.41	0.23	0.6	1.27
Sclt1	NA	NA	0.18	0.49	3.33
Scly	0	0.53	1.25	1.25	0
Scmh1	0.08	0	1.29	3.27	0
Scml2	0.97	2.07	1.02	0	NA
Scml4	0.88	NA	0	1.51	NA
Scn10a	0	1.86	3.24	1.31	4.54
Scn11a	3.31	1.48	0.51	1.5	0
Scn1a	0.61	1.79	0.1	1.34	0.33
Scn1b	1.25	2.79	1.23	2.62	0
Scn2a	0.7	NA	NA	NA	NA
Scn2b	NA	0.58	0.55	2.11	0
Scn3a	0.05	0.86	1.02	1.01	0
Scn3b	0.42	0.83	1.99	2.74	1.24
Scn4a	0	3.08	1.54	0.66	0
Scn4b	NA	NA	0.71	2.14	2.95
Scn5a	2.3	1.9	1.1	0.95	0
Scn7a	0.93	0.29	0.8	0.1	1.05
Scn8a	0.85	1.37	0	0.14	0
Scn9a	0.55	0.45	1.35	0.38	0.91
Scnm1	0	2.37	3.9	2.55	2.31
Scnn1a	2.63	0.06	0	0.61	5.5
Scnn1b	0.9	0.81	2.33	0.68	0.63
Scnn1g	2.07	2.05	0.73	4.15	3.68
Sco1	0	NA	3.22	0.38	0
Sco2	NA	1.14	1.97	0.68	0
Scoc	0	NA	3.34	1.38	0
Scotin	0.15	NA	NA	NA	NA

Scp2	0.27	0	0.85	2.27	0
Scp2d1	NA	NA	0.9	1.06	NA
Scpep1	0	0	0.15	1.8	0
Scrg1	0.39	1.12	2.1	0.33	0
Scrib	0.33	0.47	0	1.39	0
Scrn1	0.38	0.84	0.67	0	2.71
Scrn2	1.32	NA	1.34	1.51	7.26
Scrn3	0.69	2.94	1.94	1.68	0.41
Scrt1	1.14	1.33	1.97	0.4	2.78
Scrt2	NA	NA	NA	0.73	NA
Sct	3.55	0	0.75	1.08	4.3
Sctr	0	1.35	1.53	0.63	0
Scube1	0.84	NA	1.74	0.16	2.85
Scube2	1.22	1.19	0	1.38	0
Scube3	NA	1.9	2.44	0.72	0
Scye1	0.21	NA	NA	NA	NA
Scyl1	0.75	NA	1.52	3.19	0
Scyl2	NA	1.45	0.56	0.01	0
Scyl3	NA	1.2	1.49	0.46	0
Sdad1	1.64	2.31	0.14	1.07	0
Sdc1	1.02	1.63	0.75	1	2.57
Sdc2	0.37	0.89	1.12	0.59	0.9
Sdc3	0.3	0.05	1.37	0	2.46
Sdc4	0.25	1.94	0	4.58	6.68
Sdcbp	0.69	1.54	1.21	0.83	0
Sdcbp2	0.31	NA	0.02	1.9	0
Sdccag1	0	NA	NA	NA	0
Sdccag10	0	NA	NA	NA	NA
Sdccag3	NA	1.14	0.12	1.13	0.59
Sdccag8	0.5	NA	2.38	0.48	0
Sde2	NA	NA	0.81	0	NA
Sdf2	0	4.29	1.06	0.86	0
Sdf2l1	0.05	2.06	0.9	1.01	2.68
Sdf4	0.5	1.02	3.19	0.67	0
Sdha	0.18	0	1.05	0	1.61
Sdhaf1	NA	1.85	3.72	NA	1.73
Sdhaf2	NA	NA	5.82	0.47	2.65
Sdhb	0.41	0	2.12	0.76	0.16
Sdhc	0	1.04	0.06	0.21	1.24
Sdhd	0.53	0.79	0.07	0.55	0
Sdk1	1.14	NA	1.02	2.12	1.93

Sdk2	1.58	1.16	1.19	2.16	0
Sdpr	0.28	0	1.06	0	0
Sdr16c5	NA	NA	0.96	0.89	2.44
Sdr39u1	NA	0	0.25	1.38	2.1
Sdr42e1	NA	NA	3.26	0.21	1.04
Sdr9c7	NA	NA	1.94	1.48	NA
Sds	1.25	1.54	1.59	2.05	0.57
Sdsl	0	NA	1.6	0	0.12
Sebox	NA	NA	0	1.05	NA
Sec1	NA	NA	NA	NA	2.38
Sec11a	NA	1.85	2.76	0	0
Sec11c	NA	NA	1.47	1.2	0
Sec13	NA	1.65	1.27	1.45	1.65
Sec14l1	0	2.21	2.04	2.21	2.69
Sec14l2	1.58	2.19	1.12	0.48	1.48
Sec14l3	NA	NA	NA	1.89	NA
Sec14l4	0.11	0.39	0	0.2	0
Sec14l5	NA	0.31	0	1.64	0
Sec15l2	0.29	NA	NA	NA	NA
Sec16a	NA	2.19	2.59	0	0
Sec16b	NA	NA	1.25	NA	1.82
Sec22a	NA	0.5	0.49	0.81	0.25
Sec22b	NA	0.68	1.96	1.45	0
Sec22c	NA	NA	2.17	6.93	0.81
Sec23a	1.13	1.35	1.99	1.7	0.28
Sec23b	0.61	0.98	1.48	1.29	NA
Sec23ip	0.69	1.08	2.96	0.15	0
Sec24a	NA	1.62	0.95	0.98	0
Sec24b	0.28	1.17	1.18	1.3	NA
Sec24c	0.15	0	1.62	4.12	3.58
Sec24d	0.95	0.08	1.21	2.57	1
Sec31a	NA	1.27	1.35	NA	6.42
Sec31b	NA	NA	NA	0.16	NA
Sec61a1	NA	1.23	0.11	0	0
Sec61a2	0.36	1.56	0.96	0.3	1.53
Sec61b	0.27	0	0.05	0.62	0.44
Sec61g	1.22	1.64	3.53	0	0
Sec62	NA	1.47	1.64	0.62	0.21
Sec63	0.94	1.53	1.38	0.6	2.24
Secisbp2	1.94	0	1.65	0.1	2.44
Secisbp2l	NA	0.55	1.05	1.76	0

Sectm1	2.44	NA	NA	NA	NA
Sehl1	0.23	0.73	0	1.09	0
Sel1l	NA	0.13	0.16	0.16	0
Sel1l2	NA	NA	1.37	NA	1.64
Sel1l3	NA	1.03	0.14	1.73	NA
Sele	0.05	0.15	0.85	4.01	4.7
Selenbp1	0.72	1.47	2.39	NA	2.99
Selk	1.12	NA	2.66	0	0.85
Sell	0	0	0.52	2.06	0
Selm	NA	NA	1.66	0.83	0
Selp	0.45	0.39	1.03	2.99	0
Selplg	NA	1.73	0.09	1.69	0
Selrc1	NA	1.1	0.74	0.5	NA
Sema3a	0	1.23	0.57	0	2.29
Sema3b	0.17	1.13	1.94	1.85	2.7
Sema3c	0.67	0.64	0.3	1.43	0.68
Sema3d	0.49	0.48	4.51	3.04	1.5
Sema3e	0.27	1.32	1.15	1.2	1.11
Sema3f	0.13	0.69	0	0.77	3.82
Sema3g	NA	2.51	0.81	1.48	0
Sema4a	0	0.9	0.29	0.98	0.03
Sema4b	0.44	NA	4.91	0.35	1.65
Sema4c	1.75	1.89	1.21	2.38	0
Sema4d	1.35	1.49	3.12	0.64	2.73
Sema4f	0	0.92	0.57	0	0
Sema4g	1.06	1.43	0.16	0	2.09
Sema5a	1.01	0.19	0.48	1.74	2.29
Sema5b	0.75	NA	2.53	0	0.9
Sema6a	0.83	1.34	0.58	7.53	0
Sema6b	0.06	1.27	0.67	0.31	0
Sema6c	0	1.28	0.84	0.01	2.3
Sema6d	0.18	1.25	1.04	2.04	1.09
Sema7a	0	1.28	1.07	1.83	1.23
Semg1	NA	NA	NA	NA	0
Senp1	2.48	NA	1.13	2.07	0.21
Senp2	0.58	1.6	1.19	0.35	0
Senp3	0.48	1.96	0	0.68	1.32
Senp5	0.63	0.54	1.24	0.66	1.4
Senp6	0	0.25	0.19	0.04	2.59
Senp7	NA	1.44	2.52	0.31	0
Senp8	0	NA	1.73	2.7	0

41897	NA	1.77	0.34	1.24	0
Sephs1	0.53	0	1.03	0.39	0
Sephs2	0.84	2.03	0.56	2.12	5.51
Sepn1	1.67	NA	1.22	0.68	9.48
Sepp1	0	1.85	3.32	1.39	3.12
Sepsecs	NA	NA	1.21	0.72	0
41883	NA	NA	0	1.19	NA
41892	NA	0.63	0.54	0.3	NA
41893	NA	0.59	0.47	0.28	NA
41894	NA	NA	0.42	1.74	NA
41896	NA	NA	NA	1.32	NA
41884	NA	1.88	5.93	0.35	NA
41885	NA	NA	0	0.73	NA
41886	NA	1.6	2.24	2.56	NA
41887	NA	1.83	0.35	NA	NA
41888	NA	0	0.67	0.86	NA
41889	NA	0.71	0.97	0.84	NA
41890	NA	0.61	0.13	1.26	NA
41891	NA	0	0.43	0	NA
Sepw1	1.11	2	4.95	0.47	0
Sepx1	0.43	NA	NA	NA	0
Serac1	1.56	NA	0.03	1.07	0
Serbp1	NA	0	1.39	0.12	0
Serf2	1.93	1.42	0.61	NA	0
Sergef	NA	1.61	1.46	2.16	0
Serhl	0.93	NA	NA	NA	NA
Serinc1	NA	2.51	1.76	0.27	1.27
Serinc2	NA	1.36	0	1.14	0
Serinc3	NA	1.27	1.55	0	2.11
Serinc4	NA	1.57	0.49	2.7	NA
Serinc5	NA	0	2.08	0	NA
Serp1	NA	0.48	1.82	0.94	1.99
Serp2	NA	NA	0.61	1.09	2.75
Serpina1	0	NA	NA	NA	NA
Serpina10	3.82	1.11	0.45	0.21	2.68
Serpina11	0.32	NA	NA	0.16	NA
Serpina12	0.29	NA	1.76	1.87	NA
Serpina5	1.68	0.1	1.64	0.44	0
Serpina6	4.62	1.31	1.83	1.67	4.07
Serpina7	0.8	1.01	0	0.81	5.46
Serpina9	2.66	NA	2.57	3.85	NA

Serpinb10	NA	NA	NA	5.64	NA
Serpinb11	0.92	NA	1.23	1.43	NA
Serpinb12	3.61	NA	0	1.24	NA
Serpinb13	0.19	NA	NA	2.32	NA
Serpinb2	0.39	0	0.74	0	0
Serpinb5	5.31	0.98	0.1	0.27	1.38
Serpinb6	0	NA	NA	NA	NA
Serpinb7	2.17	0.75	0.88	1.26	0
Serpinb8	0.24	2.82	0.94	0.69	1.71
Serpinb9	0	1.4	2.35	1.8	0.13
Serpinc1	1.09	3.38	0.26	0.45	5.06
Serpind1	1.33	1.45	1.37	1.63	0
Serpine1	0.43	1.75	3.79	0.49	9.96
Serpine2	1.6	0.16	3.06	0.85	0
Serpinf1	0	0	2.23	1.87	0
Serpinf2	1.13	2.03	0.57	1.74	2.87
Serping1	0.58	1.66	0.35	0.7	2.58
Serpinh1	0.69	0.93	2.01	2.03	0
Serpini1	NA	1.71	0.45	0.87	0.89
Serpini2	1.76	4.69	0.84	0	0
Sertad1	0.65	NA	3.68	0.8	2.68
Sertad2	0	0	2.93	1.24	1.58
Sertad3	2.46	1.08	1.78	0.08	0
Sertad4	7.16	NA	0.62	1.92	1.87
Sertm1	NA	NA	0	13.71	NA
Sesn1	0.1	0.15	2.66	0.03	NA
Sesn2	1.24	NA	2.14	10.84	5.21
Sesn3	1.47	NA	3.21	0.47	0.71
Sestd1	0.01	NA	3.7	0	0.21
Set	0.65	2.03	1.68	0.2	0
Setbp1	1.51	NA	NA	0.28	NA
Setd1a	NA	0.26	0	1.05	0.01
Setd1b	NA	0	1.82	1.1	0
Setd2	NA	0	0.64	0.97	1.95
Setd3	NA	2.05	1.02	0.94	0.7
Setd4	NA	0.56	1.8	0.57	0
Setd5	NA	1.53	0.59	0	0
Setd6	NA	0	0.11	3.65	0
Setd7	NA	NA	1	0.48	0.97
Setd8	NA	0	0.69	2.06	0.03
Setdb1	0.86	0.65	1.49	0.69	0.22

Setdb2	NA	NA	0.51	0.74	3.3
Setmar	0	2.92	1.6	0.41	15.61
Setx	NA	0.02	1.98	0.01	0.14
Sez6	0	NA	1.23	1.13	0
Sez6l	1.37	0.38	0.5	0.77	1.2
Sez6l2	NA	1.8	2.38	2.33	10.32
Sf1	NA	0.45	0.5	2.19	1.07
Sf3a1	0.47	0.45	0.39	3.01	0.05
Sf3a2	0	1.69	3.44	0	0
Sf3a3	0	2.33	0.05	0.27	0
Sf3b1	0.38	0.08	0.77	0.02	0.74
Sf3b2	0.84	0	1.03	0	0
Sf3b3	0.22	0	0.35	5.36	1.49
Sf3b4	0.91	1.56	0.47	2.59	2.36
Sf3b5	0.16	0.3	1.41	0.32	4.43
Sf4	0.73	NA	NA	NA	1.94
Sfi1	NA	0	0.65	0.46	0
Sfmbt1	0.92	0	1.74	2	1.85
Sfmbt2	0.44	NA	1.21	3.11	0.67
Sfn	0.55	1.78	2.67	2.09	0
Sfpq	0	0	0.01	0.85	0.73
Sfr1	NA	NA	1.61	3.17	NA
Sfrp1	0.58	0.04	0.43	1.11	2.08
Sfrp2	0.4	NA	0.25	2.99	0
Sfrp4	1.79	1.41	0	0.77	5.54
Sfrp5	0.49	1.39	1.09	3.28	0
Sfrs1	0.15	NA	NA	NA	0
Sfrs10	0.32	NA	NA	NA	NA
Sfrs11	NA	NA	NA	NA	1.87
Sfrs12	NA	NA	NA	NA	0.8
Sfrs12ip1	NA	NA	NA	NA	0
Sfrs14	0	NA	NA	NA	0
Sfrs15	NA	NA	NA	NA	2.43
Sfrs16	1.53	NA	NA	NA	0.59
Sfrs18	NA	NA	NA	NA	1.45
Sfrs2	0.14	NA	NA	NA	0
Sfrs2ip	1.25	NA	NA	NA	0
Sfrs3	0.07	NA	NA	NA	0
Sfrs4	NA	NA	NA	NA	0.02
Sfrs5	0.64	NA	NA	NA	1.25
Sfrs6	0.17	NA	NA	NA	0

Sfrs7	0	NA	NA	NA	1.27
Sfrs8	2.69	NA	NA	NA	1.5
Sfrs9	0.66	NA	NA	NA	0
Sfswap	NA	0.47	1.27	1.49	NA
Sft2d1	NA	NA	0.39	0.97	0
Sft2d2	NA	0	0.3	0	1.18
Sft2d3	NA	NA	1.81	1.18	2.71
Sftpb	4.52	0	0.79	1.03	3.46
Sftpc	4.01	1.08	1.28	0.96	1.73
Sftpd	2.72	1.38	1.23	2.63	2.12
Sfxn1	0.93	2.64	0.74	1.02	0.36
Sfxn2	0	NA	0.97	0.88	1.65
Sfxn3	0	0	5.63	0	0.42
Sfxn4	0	NA	1.73	1.19	0
Sfxn5	0.28	NA	1.39	1.92	3.47
Sgca	4.36	1.51	1.58	2.54	4.15
Sgcb	1.84	0.73	1.48	0.71	0
Sgcd	0.2	1	0.4	4.5	4.27
Sgce	1.95	0.22	2.73	0.63	0.12
Sgcg	0	1.06	1.33	NA	0
Sgcz	2.77	NA	NA	0.71	NA
Sgip1	NA	NA	0.55	1.44	0
Sgk	0.45	NA	NA	NA	NA
Sgk1	NA	3.12	3.74	0.07	1.98
Sgk2	0.71	2.06	0.99	0	3.81
Sgk3	0.51	NA	3.26	0.4	0
Sgms1	NA	0.81	1.97	5.09	0
Sgms2	NA	NA	1.05	0	2.58
Sgol1	0.07	NA	1.76	0.65	1.81
Sgol2	0.57	NA	2.99	0.7	0
Sgpl1	0.02	0.68	0.03	0.28	0.3
Sgpp1	0.97	0	3.88	3.68	0
Sgpp2	NA	NA	0.85	0	0
Sgsh	0.27	1.9	4.21	NA	0
Sgsm1	NA	NA	0.97	1.23	0
Sgsm2	NA	0	1.35	0	0
Sgsm3	NA	0	2.84	1.04	2.19
Sgta	3.54	3.01	4.2	1.05	1.51
Sgtb	0.25	NA	2.89	0.07	0.53
Sh2b1	NA	0	1.58	1.52	0
Sh2b2	NA	0.32	1.35	1.32	0

Sh2b3	NA	0	7.65	0.28	NA
Sh2d1a	2.94	0.13	0.52	2.34	NA
Sh2d2a	0.93	0	1.66	1.07	0
Sh2d3c	0.9	1.44	0.62	0.61	0
Sh2d4a	1.76	0.93	1	0.98	0
Sh2d4b	2.15	NA	1.81	0	NA
Sh2d5	NA	NA	1	6.37	0.08
Sh2d6	NA	NA	1.75	2.31	0.22
Sh2d7	NA	NA	NA	0.6	NA
Sh3bgr	2.43	0.39	0.72	0.64	0.03
Sh3bgrl	1.07	0.66	0.71	0.66	0
Sh3bgrl2	2.33	NA	0.47	0	0
Sh3bgrl3	0.06	0.85	0.75	0	0.71
Sh3bp1	0.03	1.1	1.29	1.4	0
Sh3bp2	0.11	0.4	2.36	2.14	0.55
Sh3bp4	0.07	1.89	2.57	3.9	1.08
Sh3bp5	0.44	1.3	0.1	0.7	0
Sh3bp5l	NA	NA	2.28	7.69	0
Sh3d19	1.34	1.06	1.15	4.5	1.96
Sh3d21	NA	2.77	2.29	3.26	NA
Sh3gl1	0.06	1.84	2.28	0	0.07
Sh3gl2	1.03	1.61	0.85	1.19	1.97
Sh3gl3	4.14	0.32	0.24	0.06	0
Sh3glb1	0	2.56	2.06	1.47	14.29
Sh3glb2	0.25	0.3	1.63	2.35	0
Sh3kbp1	1.47	NA	2.69	0.8	0.45
Sh3pxd2a	NA	0.34	2.06	0.74	0
Sh3pxd2b	NA	NA	1.99	3.86	1.78
Sh3rf1	NA	NA	0.93	1.56	1.26
Sh3rf2	1.89	NA	1.99	1.23	2.89
Sh3rf3	NA	NA	1.97	0.37	0
Sh3tc1	4.86	2.08	1.46	1.98	1.09
Sh3tc2	NA	0.61	1.83	2.95	1.69
Sh3yl1	2.1	0	4.36	1.27	0
Shank1	NA	3.69	8.45	0	0
Shank2	0.25	1.27	1.15	1.5	3.83
Shank3	0.88	NA	1.03	1.91	2.24
Sharpin	NA	0.57	0.92	0	1.86
Shb	0	1.06	0.19	NA	1.52
Shbg	0.63	0.87	0	1.23	0
Shc1	0	0.56	0.17	0.16	0.81

Shc2	0.4	NA	NA	0.45	NA
Shc3	2.02	1.47	1.65	0	2.53
Shc4	NA	NA	0.35	2.67	5.1
Shcbp1	2.29	0.18	1.95	0.06	1.63
Shcbp1l	NA	0.17	0	0.35	NA
Shd	8.94	NA	4.11	2.01	0
She	NA	NA	0	4.46	3.17
Shf	NA	NA	5.26	0.48	0
Shfm1	NA	1.25	0.08	0	4.01
Shh	0.2	1.52	1.28	1.71	1.37
Shisa2	NA	NA	5.71	0	1.25
Shisa3	NA	NA	0.34	0.41	0.01
Shisa4	NA	NA	2.35	1.03	3.1
Shisa5	NA	NA	2.81	0.03	0.83
Shisa6	NA	NA	0.71	0.72	NA
Shisa7	NA	NA	0.74	1.52	NA
Shisa9	NA	NA	2.25	1.63	NA
Shkbp1	0	NA	1.39	2.6	0.08
Shmt1	0.7	0.6	1.26	1.57	0.55
Shmt2	0	0	0.34	0.71	1.07
Shoc2	NA	0.74	1.78	3.49	0
Shox2	0.37	0	1.39	0.81	0
Shpk	NA	1.56	1.88	0.57	1.39
Shprh	0	NA	0.86	0	0
Shq1	NA	0	0.05	0.79	2.22
Shroom1	NA	NA	0.99	3.4	0
Shroom2	NA	1.17	0	4.38	2.61
Shroom3	NA	NA	0.59	1.48	0
Shroom4	NA	NA	0.06	3.07	0.46
Si	0.75	NA	NA	NA	0.07
Siae	NA	NA	0.2	0.92	0.49
Siah2	0	1.62	2.52	0.18	3.59
Siah3	NA	NA	NA	0.96	NA
Sidt1	NA	0	0.63	0.56	2.26
Sidt2	NA	1.34	2.46	1.07	2.48
Sigirr	NA	0	1.17	1.93	0
Siglec1	NA	1.45	0.34	1.58	2.1
Siglec10	0.56	NA	NA	NA	NA
Siglec15	NA	NA	NA	0.87	NA
Siglec5	0.01	0	1.79	0.94	4.37
Sigmar1	NA	1.17	0.79	0	0

Sik1	NA	0.98	0.89	0.97	0.98
Sik2	NA	1.01	2.37	1.48	0.7
Sik3	NA	0.54	0.12	1.95	NA
Sike1	NA	1.42	0.38	0.68	0
Sil1	NA	3.04	3.95	0	0.98
Sim1	0.93	0	0.52	0.22	0
Sim2	0.75	0.79	0.75	0.1	0
Simc1	NA	NA	0.45	1.17	NA
Sin3a	0.55	NA	1.64	2.94	1.34
Sin3b	0.58	0	0.37	2.65	1.16
Sip1	0.03	NA	NA	NA	0
Sipa1	0.95	3	2.91	NA	2.24
Sipa1l1	0	0.9	0.27	0.41	0
Sipa1l2	0.32	NA	1.64	0.11	0
Sipa1l3	NA	3	3.33	0.86	1.93
Sirpa	NA	0.29	2.92	0.4	0
Sirt1	0.98	0.1	1.74	0	1.73
Sirt2	2.07	2.63	2.53	1.23	5.4
Sirt3	0.79	0	1.69	2.06	0
Sirt4	0.49	0	1.33	1.08	0
Sirt5	0.71	1.36	2.33	2.28	0
Sirt6	2.73	0	0	1.91	0
Sirt7	0.22	2.56	2.48	1.73	1.46
Sit1	NA	0	0.4	0.52	0
Siva1	NA	1.13	1.33	0.32	0
Six1	1.75	0.96	2.99	0	1.88
Six2	0.33	1.27	2.42	0.74	3.45
Six3	1.86	1.24	0.92	0	0
Six4	0.03	NA	0	0	0.72
Six5	0.29	0	2.21	1.5	0
Six6	0	2.5	0.28	0.03	2.36
Ska1	NA	0.4	5.5	0	0.68
Ska2	NA	NA	1.93	0	NA
Ska3	NA	NA	1.95	0.75	NA
Skap1	NA	0	0	0.71	1.71
Skap2	NA	1.61	0.91	1.96	0
Ski	0.16	1.11	2.62	0.64	0.21
Skida1	NA	NA	0.3	4.3	NA
Skil	0	0	1.56	0.07	0
Skiv2l	0.98	0.1	2.54	0.84	2.63
Skiv2l2	NA	0.36	1.22	0	0

Skor1	NA	NA	NA	4.53	NA
Skor2	NA	NA	NA	1.71	NA
Skp1a	1.31	NA	NA	NA	NA
Skp2	1.14	0.13	1.49	0.41	0
Sla	0.7	0.21	0.03	0.98	0
Sla2	0.53	NA	1.56	0.85	0
Slain1	NA	NA	1.42	4.69	2.16
Slain2	NA	NA	0.74	0.75	0
Slamf1	0	0	4.09	0.53	0.68
Slamf6	2.62	NA	0	2.99	NA
Slamf7	0.05	1	0.92	0.59	5.42
Slamf8	1.12	0.6	1.78	NA	2.01
Slamf9	0.42	NA	1.11	5.81	NA
Slbp	5.6	0	2.03	0.76	0
Slc10a1	0.44	0	0.76	1.11	0.11
Slc10a2	1.78	0.91	4.23	0	2.44
Slc10a3	0	2.56	2.51	NA	0
Slc10a4	NA	NA	NA	0	NA
Slc10a5	NA	NA	NA	0	NA
Slc10a6	NA	NA	NA	1.45	NA
Slc10a7	NA	NA	0	0	0
Slc11a1	0	0.4	0.01	1.38	0
Slc11a2	0.2	0.71	3.54	0.05	0
Slc12a1	0.56	1.48	0.4	3.4	2.09
Slc12a2	0	1.37	2.79	2.52	1.48
Slc12a3	0	1.29	0.41	1.14	2.63
Slc12a4	NA	1.33	3.23	0.48	2.06
Slc12a5	2.63	1.07	0.89	1.4	2.42
Slc12a6	0.48	1.69	0.68	0.8	0.63
Slc12a7	0	0.56	2.1	1.2	0
Slc12a8	0.08	1.16	1.06	0.81	0
Slc12a9	0.27	0	3.49	1.47	1.71
Slc13a1	0	1.46	1.18	0	0.99
Slc13a2	1.79	0.68	1.85	0.04	3.88
Slc13a3	0.49	0.63	1.06	2.16	0
Slc13a4	3.18	1.52	0	2.26	0
Slc13a5	3.41	NA	1.85	2.44	0.82
Slc14a1	0.23	2.69	0.14	1.48	1.08
Slc14a2	3.71	1.82	0.86	0.57	1.96
Slc15a1	2.24	1.15	0.81	0.96	0
Slc15a2	0.43	1.56	0.2	0	1.16

Slc15a3	1.16	2.38	1.28	NA	0
Slc15a4	0.16	NA	0.26	0	0
Slc15a5	NA	NA	NA	1.44	NA
Slc16a1	0	0.66	1.72	0.79	0.6
Slc16a10	0.39	0	0.5	0.1	0.24
Slc16a11	3.59	NA	2.41	0.58	3.05
Slc16a12	NA	NA	NA	1.19	NA
Slc16a13	0.01	NA	NA	10.14	NA
Slc16a14	0.75	NA	1.4	0.13	0
Slc16a2	1.64	0.47	0.75	0	0.83
Slc16a3	NA	0.67	2.06	1.37	0
Slc16a4	1	1.01	0.17	0.84	0.14
Slc16a5	0	1.18	0.72	1.61	0
Slc16a6	0.38	0.12	1.69	1.47	10.91
Slc16a7	1.24	2.92	1.77	2.57	0
Slc16a8	1.66	0.98	1.07	1.99	2.37
Slc16a9	1.52	NA	3.66	1.32	1.6
Slc17a1	2.17	0.1	4.13	0.48	0
Slc17a2	1.59	1.76	0.9	1.06	1.91
Slc17a3	0.62	1.55	1.1	2.24	3.38
Slc17a4	NA	NA	NA	0.46	NA
Slc17a5	0	2.08	1.64	0	0
Slc17a6	1	0	1.29	0	0
Slc17a7	1.61	1.61	0.82	1.68	0
Slc17a8	0	NA	NA	0	NA
Slc17a9	NA	0.51	1.36	0.43	0.65
Slc18a1	0.92	0.98	0.95	1.6	2.09
Slc18a2	0	0.61	0.45	1.36	0
Slc18a3	NA	2.04	0	1.01	21.82
Slc18b1	NA	NA	0.34	0	NA
Slc19a1	0	1.32	0	2.16	0.77
Slc19a2	2.26	0.79	0	0.99	0
Slc19a3	0.42	1.28	0	1.26	2.92
Slc1a1	0.72	0.89	0	0	0
Slc1a2	2.27	1.39	0.2	2.19	1.5
Slc1a3	1.32	0	1.06	0.42	1.94
Slc1a4	0.25	0.55	1.98	0.94	0
Slc1a5	0	1.18	1.58	3.1	0.58
Slc1a6	1.78	2.66	2.96	2.8	0
Slc1a7	3.65	0.14	2.27	0.3	0
Slc20a1	0.71	0.08	0.52	0.95	0.62

Slc20a2	0.63	0	1.28	2.27	0.68
Slc22a1	0.81	2.25	1.31	0.93	1.51
Slc22a12	0.83	NA	0	1.08	1.39
Slc22a13	0	2.03	0	0.85	1.97
Slc22a14	NA	3.31	0.07	3.81	0
Slc22a15	NA	NA	0.18	1.47	2.75
Slc22a16	0.57	NA	2.9	0	1.1
Slc22a17	0	1.84	2.07	0.04	1.35
Slc22a18	0.56	1.22	1.14	0.19	1.74
Slc22a2	0.07	0.12	0.14	0.79	1.83
Slc22a20	NA	NA	NA	0	NA
Slc22a23	NA	NA	1.65	0.67	0
Slc22a3	6.48	4.16	0	0.82	0
Slc22a4	0	4.56	1.66	0.16	1.53
Slc22a5	0.57	1.13	0.04	1.4	0
Slc22a6	0.7	0	0.05	0	5.75
Slc22a7	0	1.54	1.76	0.69	0
Slc22a8	0	1.3	0.83	1.67	0
Slc22a9	7.41	NA	NA	NA	0
Slc23a1	0	NA	0.12	0.78	0
Slc23a2	1.89	0.71	0.25	2.67	0
Slc23a3	0.67	NA	1.41	0	7.67
Slc24a1	0	0.3	0.29	1.67	1.03
Slc24a2	0.6	0.88	0.03	1.86	2.12
Slc24a3	0.52	0.45	0.53	1.21	0
Slc24a4	0.33	NA	0.92	2.16	0
Slc24a5	2.61	NA	NA	2.97	NA
Slc24a6	0.63	2.44	2.16	0.94	1.47
Slc25a1	0	1.88	4.64	2.12	0
Slc25a10	0.28	3.59	1.83	0	2.4
Slc25a11	0	0.05	0.07	1.48	1.15
Slc25a12	0.97	0.6	1.22	1.86	0
Slc25a13	0.02	0.06	3.04	0	1.26
Slc25a14	0.2	0.72	0.87	1.57	1.41
Slc25a15	0.53	0	1.89	3.21	0
Slc25a16	0	1.28	2.58	1.78	0
Slc25a17	0	0	0.77	1.34	0.6
Slc25a18	0.29	NA	0.21	0	0
Slc25a19	0.05	NA	0.71	1.91	1.63
Slc25a2	0.59	NA	0	0.39	1.25
Slc25a20	0	0	0	0.72	4.31

Slc25a21	3.81	NA	NA	0.53	NA
Slc25a22	0.55	1.51	4.38	5.2	3.2
Slc25a23	NA	1.72	3.98	2.27	0
Slc25a24	0.24	0.46	0.78	0.79	0.73
Slc25a25	1.14	NA	0.02	4.11	0
Slc25a26	0.19	NA	0	0.95	0
Slc25a27	0.14	NA	0.39	0	1.07
Slc25a28	0.84	0.11	1.95	0	0
Slc25a29	9.77	NA	0.91	1.13	1.17
Slc25a3	0.41	0.65	4.46	0.55	2.12
Slc25a30	1.39	0.27	0.78	0	0
Slc25a31	NA	0	0.81	0	0.19
Slc25a32	NA	0	5.1	1.97	0
Slc25a33	NA	NA	3.27	1.34	0
Slc25a34	NA	NA	0.67	1.58	7.08
Slc25a35	NA	NA	0.37	2.32	0
Slc25a36	NA	0.19	1.94	0.98	0
Slc25a37	NA	1.02	0.74	2	2.27
Slc25a38	NA	0	1.76	0.78	0
Slc25a39	NA	NA	1.09	0.31	0
Slc25a4	0.11	0.71	0.29	0.16	1.38
Slc25a40	NA	4.04	1.19	0.45	0
Slc25a41	NA	NA	2.74	1.12	NA
Slc25a42	NA	1.99	0.23	1.39	0
Slc25a43	NA	NA	NA	2.62	NA
Slc25a44	NA	1.47	1.94	1.84	2.06
Slc25a45	NA	NA	1.35	0.18	0
Slc25a46	NA	2.22	1.31	0.83	0
Slc25a47	NA	NA	1.86	1.41	NA
Slc25a48	NA	NA	0.37	0.85	NA
Slc25a5	0.28	3.18	1.48	0.77	0
Slc25a51	NA	NA	1.8	0.84	NA
Slc25a53	NA	NA	0.44	0	NA
Slc26a1	1.06	1.37	1.04	3.22	1.13
Slc26a10	NA	0.04	0.13	1.05	1.31
Slc26a11	1.1	NA	3.12	6.07	0
Slc26a2	0.41	1.61	1.68	4.72	0
Slc26a3	0.73	0.72	0.45	12.87	2.26
Slc26a4	3.21	0.05	0.62	0.16	0.01
Slc26a5	NA	NA	NA	1.83	NA
Slc26a6	0	2.44	0.82	5.56	0

Slc26a7	0.32	NA	0.84	NA	1.15
Slc26a8	0	NA	0	4.19	0
Slc26a9	0.24	NA	NA	2.39	NA
Slc27a1	0.28	NA	2.24	0.75	4.24
Slc27a2	1.97	1.95	1.03	1.41	1.67
Slc27a3	0	1.46	1.81	2.56	0
Slc27a4	3.39	NA	0.36	0	0.06
Slc27a5	0	0.42	1.81	0.42	1.21
Slc27a6	4.73	NA	NA	0.99	NA
Slc28a1	NA	NA	NA	1.93	NA
Slc28a2	1.24	NA	NA	1.87	NA
Slc28a3	0.53	1.78	1.01	1.51	0
Slc29a1	0	0	0	2.54	2.64
Slc29a2	1.47	2.51	0.03	2.49	0.34
Slc29a3	0.22	0	0.35	1.78	0.88
Slc29a4	2.77	NA	0.69	0	NA
Slc2a1	0.6	2.68	1.99	0	2.73
Slc2a10	1.06	1.29	0.44	1.47	1.71
Slc2a12	2.18	NA	0.13	0.75	0
Slc2a13	NA	NA	0	1.72	0.59
Slc2a2	0	2.5	3.09	1.02	2.89
Slc2a3	0.55	2.68	0.5	0	0
Slc2a4	0	1.35	0.19	1.16	4.42
Slc2a5	0.86	0.13	2.42	0.82	3.61
Slc2a6	0.07	1.05	1.56	0.67	0.49
Slc2a7	NA	NA	NA	0.39	NA
Slc2a8	0	0.84	2.1	0.35	1.49
Slc2a9	0.68	3.47	2.82	2.12	1.71
Slc30a1	0	1.75	1.58	2.14	0.06
Slc30a10	NA	1.02	0.6	2.38	3.65
Slc30a2	1.99	NA	0.08	1.69	1.14
Slc30a3	0.9	2.26	0.76	0.9	1.1
Slc30a4	0.72	1.85	4.75	0.57	0
Slc30a5	0.83	0.41	1.87	0.66	0
Slc30a6	0.51	2.34	2.14	1.08	2.57
Slc30a7	0.85	NA	2.32	0	1.52
Slc30a8	0.65	NA	1.56	1.44	0
Slc30a9	0.63	1.77	2.49	5.11	1.98
Slc31a1	1.24	0	2.38	1.22	2.99
Slc31a2	0.04	0.66	1.96	1.12	1.12
Slc32a1	NA	NA	0.73	0.86	0

Slc33a1	1.92	1.47	3.48	1.45	0
Slc34a1	0	1.35	0.75	0.13	10.13
Slc34a2	1.7	0.7	0.28	0	1.31
Slc34a3	1.29	NA	2.4	0.47	NA
Slc35a1	0.51	3.07	1.63	1.49	NA
Slc35a2	1.64	0.35	0.68	0.48	0
Slc35a3	0.14	0.94	0.65	0.56	2.02
Slc35a4	0.14	NA	0.62	2.57	1.62
Slc35a5	0	0.07	0.75	1.9	0
Slc35b1	0	0.17	1.31	0.33	0
Slc35b2	NA	NA	1.22	0	1.2
Slc35b3	0.32	NA	2.81	1.18	3.21
Slc35b4	3.11	NA	1.4	0.81	0.11
Slc35c1	0.02	1.63	3.56	2.99	0
Slc35c2	0.18	0.8	1.1	4.63	1.34
Slc35d1	0.48	0	0.19	5.73	1.07
Slc35d2	NA	0.83	4.43	1.69	3.66
Slc35d3	0.22	NA	2.79	0.55	5.05
Slc35e1	NA	1.03	1.73	0	7.76
Slc35e2	NA	1.41	3.51	8.28	0
Slc35e3	0.23	0.1	0.7	1.16	0.17
Slc35e4	0.08	NA	0.54	6.45	1.47
Slc35f1	0.88	NA	0.58	0	0.34
Slc35f2	2.39	0	0.57	0.86	NA
Slc35f3	2.27	NA	0.33	2.72	2.79
Slc35f4	0.82	NA	2.09	1.67	2.1
Slc35f5	NA	0	1.05	0	0
Slc35f6	NA	1.26	2.52	1.29	NA
Slc35g1	NA	NA	2.41	0.33	NA
Slc35g2	NA	NA	NA	0	NA
Slc35g3	NA	NA	1.59	2.66	NA
Slc36a1	0.33	0.85	2.08	3.08	1.11
Slc36a2	0.94	NA	NA	1.08	NA
Slc36a3	0.33	NA	NA	0	NA
Slc36a4	0.47	NA	3.13	1.69	3.74
Slc37a1	1.18	0.38	1.63	1.18	0.71
Slc37a2	NA	NA	1.9	4.91	0
Slc37a3	1.19	NA	1.67	0.1	0
Slc37a4	0	1.28	1.04	0.82	1.71
Slc38a1	0.9	0	1.35	2.02	0
Slc38a10	NA	0.71	0.9	0	0.3

Slc38a11	NA	NA	NA	2	NA
Slc38a2	0	0.53	1.91	1.7	0
Slc38a3	2.1	2.26	0.1	1.29	0
Slc38a4	0.98	2.79	0.51	2.52	2.85
Slc38a5	0.68	NA	0	0	0.35
Slc38a6	NA	2.13	0.94	1.74	NA
Slc38a7	NA	0.99	0.61	0.97	0
Slc38a8	NA	NA	NA	1.73	NA
Slc38a9	NA	NA	2.17	1.08	0.2
Slc39a1	0.51	2.9	2.25	1.21	0
Slc39a10	0.17	NA	2.03	0.27	0
Slc39a11	0.02	NA	1.15	0.73	7.04
Slc39a12	NA	NA	0.97	0.7	NA
Slc39a13	0.99	NA	4.79	3.25	0.46
Slc39a14	1.44	2.01	0.85	2.33	1.02
Slc39a2	2.48	2.42	0.02	0	4.49
Slc39a3	0	NA	0.14	1.32	1.14
Slc39a4	0.3	0.77	3.27	0.91	3.3
Slc39a5	1.08	NA	1.07	0.59	0
Slc39a6	1.26	0	2.46	0.87	0
Slc39a7	2.82	NA	NA	0	2.54
Slc39a8	2.85	0	4.34	0.24	0
Slc39a9	1.34	0.56	0.48	0.85	0
Slc3a1	1.22	1.56	0	NA	0
Slc3a2	1.48	1.85	2.17	1.06	1.24
Slc40a1	0.19	NA	2.19	4.28	2.42
Slc41a1	0	NA	0.66	1.61	2.18
Slc41a2	2.88	NA	1.6	0.2	2.72
Slc41a3	1.31	0	1.13	1.77	0
Slc43a1	0.65	0	5.89	0.26	0.62
Slc43a2	NA	NA	2.47	1.5	3.3
Slc43a3	0.31	1.29	2.95	0.89	6.79
Slc44a1	NA	2.05	0.68	0	0
Slc44a2	NA	NA	0.61	0.79	0
Slc44a3	NA	NA	0	0.06	1.25
Slc44a4	NA	1.71	4.04	2.39	0
Slc44a5	NA	NA	NA	0.6	NA
Slc45a1	NA	NA	1.23	2.78	0
Slc45a2	NA	0.43	0.47	0.48	3.67
Slc45a3	NA	NA	0.11	1.11	0
Slc45a4	NA	NA	2.06	0	0.82

Slc46a1	NA	NA	0.39	1.49	0
Slc46a2	NA	NA	1.88	1.27	0
Slc46a3	NA	2.87	1.12	0	0.4
Slc47a1	NA	0	1.83	1.41	0.78
Slc47a2	NA	NA	NA	0.22	NA
Slc48a1	NA	0.29	2	0.65	0
Slc4a1	0.76	0.99	0.27	0.64	4.79
Slc4a10	2.03	1.77	1.55	1.97	2.54
Slc4a11	0.98	NA	3.49	4.12	0
Slc4a1ap	0	1.24	1.28	0.88	0.22
Slc4a2	1.12	1.52	1.97	0.78	2.69
Slc4a3	0.69	1.02	0.63	0.95	0
Slc4a4	0.06	0.52	0.38	0.03	1.91
Slc4a5	NA	0.66	0.91	8.57	3.04
Slc4a7	0.41	0.66	0.04	0	0
Slc4a8	2.07	0.81	0.68	0.14	0.56
Slc4a9	0.41	NA	3.98	0	5.42
Slc50a1	NA	4.34	0.25	0	NA
Slc51a	NA	NA	0.09	0	NA
Slc51b	NA	NA	1.65	2.04	NA
Slc52a2	NA	0	1.02	12.93	NA
Slc52a3	NA	NA	0.99	4.08	NA
Slc5a1	0	1.26	1.12	1.5	2.2
Slc5a10	0.67	NA	0.54	0.09	0
Slc5a11	0.68	NA	0.98	0	0
Slc5a12	2.25	0.21	0.7	1.32	0
Slc5a2	0.89	1.56	1.41	NA	0
Slc5a3	0	0.76	0.89	1.09	2.14
Slc5a5	0	0.58	0	2.25	0
Slc5a6	0.03	0	4.11	2.07	1.92
Slc5a7	0	1.98	0.96	0.31	1.29
Slc5a8	0	NA	3.07	1.42	0
Slc5a9	0.06	NA	0.93	1.53	0
Slc6a1	0.85	3.34	1.7	1.96	0
Slc6a11	NA	1.89	0.41	2.57	2.2
Slc6a12	0.2	3.17	0	1.07	0
Slc6a13	1.39	2.14	1.55	1.78	0
Slc6a14	1.46	0.05	1.32	0.43	1.13
Slc6a15	0.37	1.79	2.25	1.03	0
Slc6a16	NA	0	2.93	1.25	0
Slc6a17	2.32	NA	1.08	0.31	0

Slc6a18	3.63	NA	0.15	0.52	4.03
Slc6a19	0.39	NA	0.74	0.2	0
Slc6a2	0.05	0.94	0.32	2.99	2.31
Slc6a20	0	NA	NA	NA	NA
Slc6a3	1.13	0.17	1.91	0.67	0
Slc6a4	0.58	1.14	1.28	0.64	0
Slc6a5	0	1.33	1.54	0.02	0
Slc6a6	1.03	0.39	1.78	2.48	0
Slc6a7	1.04	0.08	0.7	3.25	0
Slc6a8	0	0.84	1.53	0.07	1.74
Slc6a9	0.45	1.21	1.52	0.85	2.51
Slc7a1	0.08	0.75	2.59	2.11	0.6
Slc7a10	0.85	1.79	1.52	NA	3.56
Slc7a11	0	0.23	0.38	1.6	1.22
Slc7a13	1.74	NA	1.2	0	2.78
Slc7a14	NA	NA	1.22	2.62	0.63
Slc7a2	0.74	1.36	0.97	1.78	0
Slc7a3	0.3	NA	1.53	3.92	0.87
Slc7a4	1.15	0.37	4.78	0	3.63
Slc7a5	0.52	1.31	1.32	1.88	NA
Slc7a6	0	0	1.21	NA	2.17
Slc7a6os	NA	NA	1.34	0.74	0
Slc7a7	0	2.87	1.47	NA	0
Slc7a8	0.55	1.97	3.23	0	1.68
Slc7a9	1.23	0	3.12	1.64	0
Slc8a1	0.69	0.27	0.12	0	0.72
Slc8a2	0.71	1.36	0	0.46	3.27
Slc8a3	0	NA	0.05	0.83	NA
Slc9a1	0.13	1.76	1.98	1.28	1.07
Slc9a2	0.71	3.04	1.02	0.94	0
Slc9a3	0	2.05	0	1.18	0
Slc9a3r1	0.78	2.76	0.85	0.43	2.68
Slc9a3r2	0.47	1.63	6.43	2.04	0
Slc9a4	0	NA	NA	1.95	NA
Slc9a5	NA	1.71	0.94	2.44	1.18
Slc9a6	0.16	1.4	1.89	0	0
Slc9a7	0	NA	NA	0	NA
Slc9a8	0.8	1.29	0.56	0	0
Slc9a9	0	NA	3.06	0.73	0
Slc9b1	NA	NA	0.66	0.11	NA
Slc9b2	NA	NA	1.38	1.12	NA

Slc9c1	NA	NA	NA	0	NA
Slco1c1	1	1.83	2.72	0	0
Slco2a1	1.3	0.82	0.82	3.26	1.86
Slco2b1	0.09	0.35	1.24	1.72	0
Slco3a1	0.34	1.08	2.78	4.07	1.31
Slco4a1	1.05	1.72	0.67	2.31	4.64
Slco4c1	0	1.01	1.25	1.77	0
Slco5a1	1.39	0.65	0	0	2
Slfn5	0	NA	1.54	15.65	0.83
Slfnl1	NA	NA	0.39	0.35	NA
Slirp	NA	0	3.33	NA	NA
Slit1	4.16	1.18	2.01	NA	2.29
Slit2	0.7	1.36	1.15	4.14	2.72
Slit3	1.1	0.54	0.63	0	0
Slitrk1	0.61	NA	2.32	0	1.38
Slitrk2	1.51	NA	0.62	1.19	1.12
Slitrk3	0.66	0.94	5.92	0.93	0
Slitrk4	0.13	NA	0.35	1.44	0.39
Slitrk5	2.71	1.52	0.52	1.73	4
Slitrk6	1.9	NA	2.7	2.65	2.39
Slk	NA	0.06	1.99	0	2.01
Slmap	0.31	NA	0.65	1.83	1.22
Slmo1	NA	0	0.99	2.83	0
Slmo2	NA	1.66	2.1	0.1	8.41
Sln	1	0.33	0	0.92	0
Slpi	0.54	0	0.57	0.31	0
Sltm	NA	0.51	1.51	1.15	0
Slu7	NA	NA	1.13	1.12	0.34
Slurp1	NA	1.72	2.08	0.56	0
Slx4	NA	NA	4.21	1.12	NA
Slx4ip	NA	NA	1.4	0.28	NA
Smad1	0.51	1.98	3.68	1.53	1.43
Smad2	0.89	0.43	2.27	0.58	5.98
Smad3	1.58	0.64	0.65	0	0
Smad4	0.83	0.82	2.08	1.4	2.11
Smad5	0.65	0.66	1.19	1.52	4.23
Smad6	0	1.32	1.92	1.61	2.09
Smad7	0	0	1.26	2.19	0
Smad9	0.4	1.53	0.27	0	NA
Smagp	NA	0	4.78	2.06	1.53
Smap1	1.87	0.65	1.62	NA	0

Smap2	NA	NA	1.48	1.26	0
Smarca1	1.55	0.99	2.59	0	0
Smarca2	7.08	0	1.65	0	2.2
Smarca4	0.14	1.23	0.91	0	0
Smarca5	2.37	0.75	1.23	1.94	2.01
Smarcad1	0.12	NA	0.62	0.12	0
Smarcal1	0	0	1.37	0	2.63
Smarcb1	0.81	0.97	0.49	0.57	0.56
Smarcc1	0	0	1.74	5.18	0
Smarcc2	NA	0	1.57	0	0
Smarcd1	0.82	0.78	0.68	0	0.76
Smarcd2	0.35	0.05	0.49	NA	3.89
Smarcd3	1.14	1.09	1.1	1.81	0
Smarce1	0.54	0	1.57	0.14	1.66
Smc1a	NA	0.95	0.4	0	1.61
Smc1b	NA	NA	1.29	1.12	NA
Smc2	NA	0.71	0.06	0	1.45
Smc3	NA	0.1	0.02	0.79	0
Smc4	NA	0.97	3.83	1.63	0
Smc5	NA	0.11	0	0.92	0
Smc6	NA	1.16	0.3	0.54	0
Smchd1	NA	1.41	0.07	0.04	0.56
Smco2	NA	NA	NA	1.75	NA
Smco3	NA	NA	1.8	0.25	NA
Smco4	NA	1.19	1.74	3.21	NA
Smcp	NA	1.19	0	0.63	2.14
Smcr7	NA	NA	3.78	1.39	0
Smcr7l	NA	2.7	0.23	0.45	0
Smcr8	NA	NA	1	0.66	2.73
Smdt1	NA	NA	4	0	NA
Smek1	NA	0	0.79	0.28	0
Smek2	NA	0.25	1.06	0.41	1.18
Smg1	NA	0.96	0.39	0	0.02
Smg5	NA	1.29	1.1	5.18	0
Smg6	NA	0.25	0.55	0	0
Smg7	NA	1.08	0.59	3.4	0
Smg8	NA	1.55	0.95	0.38	NA
Smg9	NA	0	1.09	3.38	NA
Smim1	NA	NA	0	NA	NA
Smim11	NA	NA	2.64	0.99	NA
Smim12	NA	NA	1.95	0	NA

Smim13	NA	NA	1.29	NA	NA
Smim14	NA	2.53	0.98	0.34	NA
Smim15	NA	NA	2.96	1.34	NA
Smim19	NA	NA	0.92	1.16	NA
Smim20	NA	NA	0.71	2.18	NA
Smim3	NA	NA	2.17	1.36	NA
Smim4	NA	NA	2.93	0.62	NA
Smim5	NA	NA	0.68	3.17	NA
Smim6	NA	NA	0.46	NA	NA
Smim7	NA	1.16	1.1	0.46	NA
Smim8	NA	0.64	1.91	1.18	NA
Smn1	0	NA	NA	NA	NA
Smndc1	NA	1.12	1.89	1.12	1.44
Smo	0.85	1.71	1.7	0.84	1.42
Smoc1	1.92	NA	1.85	1.06	1.49
Smoc2	0.26	NA	1.37	0.62	1.75
Smox	0	0.61	1.24	0.32	0
Smpd1	0.28	0.79	0	4.49	0.01
Smpd2	0.04	2.74	1.27	0	9.19
Smpd3	0	0	0.77	3.24	0
Smpd4	NA	NA	NA	3.44	NA
Smpdl3a	0	3.66	1.36	1.03	4.24
Smpdl3b	0.7	1.51	2.41	1.58	8.4
Smpx	3.56	1.21	0.42	5.1	0
Smr3a	NA	1.03	0	1.33	0
Sms	NA	0	3.5	NA	3.23
Smtn	1	1.99	2.04	1.94	0
Smtnl1	NA	NA	0.41	NA	0
Smtnl2	NA	NA	2.38	0.54	1
Smu1	0	0.82	1.17	0.85	0.85
Smug1	0	4.09	1.62	0	0
Smurf1	0.28	0.86	3.02	0.81	0
Smurf2	0.34	1.53	1.16	0.02	3.19
Smyd1	0.19	NA	0	2.68	1.49
Smyd2	0	2.57	1.53	0.7	1.66
Smyd3	0.76	0	1.75	0.54	2.13
Smyd4	NA	NA	0.88	4.16	1.12
Smyd5	0	0	0.03	1.2	0.83
Snag1	2.14	NA	NA	NA	NA
Snai1	0.87	0.77	1.01	0.33	0.24
Snai2	0.94	0.07	0.02	1.41	1.1

Snai3	0	NA	0	1.02	NA
Snap23	0	0.74	1.93	0.97	0
Snap25	0	0.8	1.1	1.2	0
Snap29	0.12	2.12	0.72	0.74	0
Snap47	NA	NA	3.12	NA	0
Snap91	0.27	1.28	1.25	1.22	0
Snapap	0.17	NA	NA	NA	NA
Snapc1	1.08	0.07	3.49	0.73	2.36
Snapc2	0	1.95	0.85	2.41	1.27
Snapc3	0	0.61	0.97	0	2.66
Snapc4	0.86	0	1.63	1.53	0.03
Snapc5	0.14	0.29	0.76	2.67	0
Snapin	NA	NA	1.01	NA	0.75
Snca	0	0.94	1.11	2.83	1.29
Sncaip	0	1.44	1.41	4.07	0
Sncb	0	1.78	1.43	0.76	2
Sncg	1.46	1.36	0.19	4.24	2.37
Snd1	NA	1.94	0	0	2.44
Sned1	0.57	1.28	1.37	1.22	4.2
Snf1lk	0	NA	NA	NA	NA
Snf8	NA	0.1	0.1	0	2.54
Snhg1	NA	NA	2.39	NA	2.56
Snhg10	NA	NA	0.76	NA	0.35
Snhg11	NA	NA	1.62	NA	0
Snhg12	NA	NA	0.17	NA	0
Snhg3	NA	0.89	3.03	NA	2.22
Snhg4	NA	NA	0	NA	NA
Snhg5	NA	NA	0.59	NA	NA
Snhg6	NA	NA	2.96	NA	4.84
Snhg7	NA	NA	0.76	NA	0
Snhg8	NA	NA	4.36	NA	0
Snip1	1.02	1.14	0.9	1.15	4.87
Snn	0.23	0.86	0.96	NA	0
Snora16a	NA	NA	NA	0.44	NA
Snora19	NA	NA	NA	0.52	NA
Snora20	NA	NA	NA	0	NA
Snora21	NA	NA	NA	0.04	NA
Snora23	NA	NA	NA	0.29	NA
Snora24	NA	NA	NA	0.13	NA
Snora28	NA	NA	NA	4.7	NA
Snora2b	NA	NA	NA	0	NA

Snora3	NA	NA	NA	0	NA
Snora30	NA	NA	NA	2.11	NA
Snora31	NA	NA	NA	0	NA
Snora33	NA	NA	NA	0.13	NA
Snora41	NA	NA	NA	0	NA
Snora44	NA	NA	NA	0.79	NA
Snora52	NA	NA	NA	0.21	NA
Snora5c	NA	NA	NA	2.56	NA
Snora61	NA	NA	NA	1.16	NA
Snora62	NA	NA	NA	0.3	NA
Snora69	NA	NA	NA	1.25	NA
Snora70	NA	NA	NA	0.69	NA
Snora74a	NA	NA	NA	0.78	NA
Snora75	NA	NA	NA	0.52	NA
Snora7a	NA	NA	NA	0.07	NA
Snord104	NA	NA	1.47	0.64	1.78
Snord123	NA	NA	NA	NA	4.01
Snord14e	NA	NA	NA	0.71	NA
Snord15a	NA	NA	NA	0.82	NA
Snord15b	NA	NA	NA	0.71	NA
Snord1c	NA	NA	NA	0.39	NA
Snord32a	NA	NA	NA	0.02	NA
Snord33	NA	NA	NA	0.06	NA
Snord34	NA	NA	NA	1.55	NA
Snord35a	NA	NA	NA	0	NA
Snord37	NA	NA	NA	3.31	NA
Snord38a	NA	NA	NA	2.07	NA
Snord42b	NA	NA	NA	0	NA
Snord49a	NA	NA	NA	4.39	NA
Snord49b	NA	NA	NA	0.79	NA
Snord52	NA	NA	NA	2.26	NA
Snord53	NA	NA	NA	0.74	NA
Snord55	NA	NA	NA	0.32	NA
Snord57	NA	NA	NA	0	NA
Snord58b	NA	NA	NA	0	NA
Snord61	NA	NA	NA	0.82	NA
Snord68	NA	NA	NA	0.53	NA
Snord7	NA	NA	NA	3.11	NA
Snord73a	NA	NA	NA	3.82	NA
Snord8	NA	NA	NA	0.52	NA
Snord82	NA	NA	NA	0	NA

Snord89	NA	NA	0.57	NA	0
Snord95	NA	NA	NA	0.64	NA
Snord96a	NA	NA	NA	2.33	NA
Snph	0.3	0	0.41	NA	0.79
Snrk	1.12	0.47	2.04	0.66	0
Snrnp200	NA	0.11	1.03	0	0
Snrnp25	NA	1.91	1.86	0.72	0
Snrnp27	NA	0.01	2.29	1.75	0
Snrnp35	NA	1.11	0.97	3.27	1.78
Snrnp40	NA	2.1	0	0.74	4.38
Snrnp48	NA	NA	0.81	0.87	2.18
Snrnp70	NA	0.17	1.08	2.84	1.2
Snrp70	0.62	NA	NA	NA	NA
Snrpa	0.32	2.71	0.17	0	1.91
Snrpa1	0	1.43	1.07	1.84	3.06
Snrpb	0.6	3.01	0.19	0.76	0
Snrpb2	0.65	0.33	2.12	1.3	2.58
Snrpc	NA	0.87	1.04	0	2.14
Snrpd1	0.25	0.25	2.26	1.18	1.47
Snrpd2	0.21	0.17	2.24	NA	0
Snrpd3	0.25	0.45	0.38	NA	1.47
Snrpe	0.74	1.05	0.07	1.37	0
Snrpf	0.24	2.01	0.24	3.69	0
Snrpg	0	1.19	1.04	0	0
Snrpn	0	1.38	2.11	NA	1.78
Snta1	0.67	0.97	3.53	2.43	1.96
Sntb1	0.88	1.52	1.32	1.17	2.62
Sntb2	0	0.86	1	0	0
Sntg1	0.8	1.43	0.21	0	0
Sntg2	1.09	1.77	0.87	0	0
Sntn	NA	NA	NA	0	NA
Snupn	NA	0.1	1	1.28	0
Snurf	NA	1.52	0.41	NA	NA
Snw1	NA	0.82	0.46	1.12	0.49
Snx1	0	0.7	0.79	1.07	1.34
Snx10	0	0.71	2.17	0.77	0
Snx11	0.76	2.22	2.62	0	0
Snx12	0	NA	0.94	1.1	1.26
Snx13	0.27	0	0.99	0.18	0
Snx14	0.54	NA	0.71	0	5.61
Snx15	0	2.04	1.51	0.92	1.23

Snx16	0.49	0.45	1.41	0.28	0
Snx17	0.1	1.51	2.21	0.66	0
Snx18	NA	NA	1.97	1.12	3.63
Snx19	0	0.68	0.06	0	0.57
Snx2	0	2.39	1.7	0.97	0
Snx20	NA	NA	0.56	0.66	0.98
Snx21	NA	NA	1.57	NA	2.04
Snx22	NA	NA	0.02	0.82	4.72
Snx24	0	0.97	2.26	0.34	0
Snx25	0.93	NA	0.58	0.94	0.5
Snx26	1.33	NA	NA	NA	NA
Snx27	0.28	0	0.77	0.87	1.33
Snx29	NA	1.41	0.36	0	1.6
Snx3	0	2.38	1.51	0	3.33
Snx30	NA	NA	0.68	0	0
Snx31	NA	NA	1.01	0.52	2.63
Snx32	NA	NA	2.1	2.12	1.04
Snx33	NA	NA	0.11	0	0
Snx4	0	1.29	1	0.42	1.7
Snx5	0	0	0.76	0.98	0
Snx6	0.74	0	0.55	0.7	0.37
Snx7	0.43	0	0.85	1.53	1.97
Snx8	0.05	NA	2.54	2.41	0
Snx9	1.2	NA	2.33	5.71	0
Soat1	0.01	2.48	2.15	1.24	0.78
Soat2	0	1.28	4.19	1.99	4.97
Sobp	NA	1.62	0.9	0	4.56
Socs1	0	0.91	1.94	1.39	0
Socs2	0.64	1.84	2.4	0.7	1.48
Socs3	0.09	0.46	3.9	0	0
Socs4	0.81	NA	1.53	1.31	0
Socs5	0.5	1.6	2.09	3.66	0
Socs6	0	0.64	2.67	2.42	2.2
Socs7	1.33	1.21	0.44	0.99	0.93
Sod1	0	1.27	1.96	1.46	0.01
Sod2	0.07	0.1	1.63	0.94	0
Sod3	0	0.21	2.08	8.24	0
Soga1	NA	1.88	1.53	1.86	NA
Soga2	NA	0.99	0.5	0.86	NA
Soga3	NA	NA	0.04	0.45	NA
Sohlh1	NA	NA	0.91	1.82	NA

Sohlh2	NA	0.88	1.02	0	0
Solh	0.38	0	0.36	0.86	0
Son	0.4	1.24	0.74	1.36	0.8
Sorbs1	0.68	1.13	1.34	0	1.03
Sorbs2	NA	1.26	0.57	0.47	2.64
Sorbs3	NA	0	3.73	0.51	1.95
Sorcs1	0.19	NA	0.01	3.31	3.04
Sorcs2	1.6	NA	1.32	0	0
Sorcs3	0.15	1.55	1.31	1.08	0
Sord	NA	0	2.55	0.56	0
Sorl1	1.66	1.01	0.99	0.07	2.57
Sort1	0	1.57	0.8	0	0
Sos1	0.03	0.43	0.77	0.88	1.03
Sos2	0	0	3.2	2.27	0
Sost	0.88	NA	1.07	2.07	0
Sostdc1	0.71	0.46	1.31	0	0.81
Sowaha	NA	NA	0.57	2.15	NA
Sowahb	NA	NA	NA	0.69	NA
Sowahc	NA	0	2.25	1.05	NA
Sowahd	NA	NA	NA	0.67	NA
Sox1	2.03	0.25	0	0.89	0
Sox10	2.09	0.55	0.77	0.2	1.38
Sox11	0.48	1.05	1.23	1.2	2.11
Sox12	0.52	0	0.45	1.42	0.24
Sox13	3.4	0	0.39	1.63	1.37
Sox14	0	2.08	0	0.28	1.59
Sox15	0.96	2.23	0.21	NA	0
Sox17	0.16	0.24	0.12	0.24	1.93
Sox18	1.63	1.6	1.87	0.85	0.9
Sox2	0	0.22	0.05	0	0
Sox21	1.24	1.18	1	1.66	1.86
Sox2ot	NA	NA	NA	NA	0.94
Sox3	0	0.93	0.34	1.14	0
Sox30	0	1.85	2.82	2.31	0
Sox4	0.6	0.5	0.02	1.74	0.63
Sox5	0.24	0.2	0.22	2.49	0
Sox6	0.71	NA	0.48	1.2	0.59
Sox7	0.47	NA	0.47	2.48	0.12
Sox8	1.79	NA	4	1.54	1.7
Sox9	4.2	1	0.74	0.83	0
Sp1	0	0.19	3.18	1.76	1.45

Sp100	0.06	1.97	0.24	0	0
Sp110	NA	0	1.1	0	0
Sp140	NA	NA	NA	0.95	NA
Sp2	0.93	0.84	0.76	1.38	7.3
Sp3	0	1.21	2.67	0	1.04
Sp4	0	0.18	0.83	1.58	0.91
Sp5	2.36	NA	1.9	4.11	0
Sp6	0.07	NA	1.36	2.49	0.6
Sp7	0	NA	1.53	1.47	NA
Sp8	0	NA	0.89	1.96	3.89
Sp9	NA	NA	NA	1.62	NA
Spa17	0.65	1.54	0.95	NA	0.02
Spaca1	NA	1.28	2.11	0	0
Spaca3	0	NA	0.05	1.15	0.26
Spaca4	NA	NA	1.5	0.67	3.28
Spaca7	NA	NA	1.21	1.08	NA
Spag1	0	2.9	1.24	1.25	1.68
Spag11	1.54	NA	NA	NA	NA
Spag11a	NA	NA	NA	0.5	NA
Spag16	0	3.15	0.52	0.87	0.52
Spag17	NA	NA	0.93	0.03	0
Spag4	0.07	1.04	0	0.92	4.6
Spag4l	4.23	NA	NA	NA	NA
Spag5	0	0.74	2.58	NA	5.13
Spag6	0.03	0.76	2.17	1.06	7.71
Spag7	0.51	0.06	0.67	0	6.56
Spag8	NA	2.15	1.46	2.19	0.05
Spag9	0.7	0.57	0.42	0	0.46
Spam1	0.12	1.33	1.26	0.77	2.4
Sparc	1.84	0	0	2.42	1.21
Sparcl1	0.09	1.44	1.11	0.36	0
Spast	NA	1.86	1.43	0.7	1.52
Spata1	3.23	NA	NA	NA	0
Spata13	0.07	NA	1.28	7.73	NA
Spata16	NA	NA	1.21	0	0
Spata17	NA	NA	0.31	0	0.85
Spata18	NA	NA	1.16	1.13	0.56
Spata19	NA	NA	0.74	2.52	NA
Spata2	5.07	0.67	0.68	1.5	0
Spata20	NA	NA	NA	0.57	NA
Spata21	NA	NA	0.79	0.6	NA

Spata22	NA	NA	NA	0.87	NA
Spata24	NA	NA	2.88	0.11	NA
Spata25	NA	NA	1.18	1.72	NA
Spata2l	NA	3.96	0.17	0.71	0
Spata3	NA	NA	0	1.51	2.12
Spata4	2.53	NA	2.25	0.14	3.27
Spata5	2.28	NA	1.44	1.61	0.7
Spata5l1	2.8	0.89	0.08	NA	0.24
Spata6	0.25	1.35	1.25	0.73	0.58
Spata7	NA	2.98	0	0.73	1.36
Spata9	NA	NA	1.61	2.23	1.29
Spatc1	NA	NA	1.16	1.48	0
Spatc1l	NA	NA	1.56	1.41	NA
Spats1	1.9	NA	NA	3.94	0
Spats2	0	0.37	0.96	0.82	0
Spats2l	NA	0.59	1.1	0.82	0
Spc24	NA	NA	3.46	2.23	2.24
Spc25	NA	1.27	1.45	0.81	2.58
Spcs1	NA	0.62	1.05	NA	0
Spcs2	NA	NA	4.62	1.25	1.27
Spcs3	NA	0.83	2.36	0.57	0
Spdef	9.81	1.74	1.49	0.02	0
Spdl1	NA	1.65	2.22	1.44	NA
Spdya	NA	NA	1.32	8.76	0.71
Specc1	NA	NA	1.12	9.57	NA
Specc1l	NA	0.81	1.33	1.22	NA
Spef1	NA	1.55	0.68	1.96	1.58
Spef2	NA	NA	0.81	0.97	0
Speg	NA	1.06	0.51	0.51	1.66
Spem1	NA	NA	3.18	2.38	6.29
Spen	NA	1.37	1.45	4.81	0
Spert	NA	NA	1.44	4.95	NA
Spesp1	0.33	NA	0.38	3.2	0
Spg11	NA	0.29	1.16	0	0.75
Spg20	0	0.09	0.62	0.43	0
Spg21	0.32	1.93	0.9	1.91	0
Spg3a	2.75	NA	NA	NA	NA
Spg7	0.96	0	0.81	0	4.09
Sphk1	0	1.45	1.78	1.11	0
Sphk2	0.14	1.21	0.6	0	0.82
Sphkap	NA	NA	0	1.18	2.07

Spib	0.25	0.05	0.97	1.46	0
Spic	1.92	NA	4.28	7.12	NA
Spice1	NA	2.06	0	0.34	NA
Spin1	NA	1.1	2.47	0.39	2.48
Spin4	NA	NA	1.59	2.06	0
Spink14	NA	NA	NA	1.32	NA
Spink2	0	0.75	0.2	1.45	0
Spink4	0	3.42	0.04	2.64	2.17
Spink5	0	1.42	0.13	1.18	0
Spink6	NA	NA	NA	0.45	NA
Spink7	NA	NA	NA	0.6	NA
Spinlw1	0.44	NA	NA	NA	4.2
Spint1	0.78	2.4	0.15	2.77	0.87
Spint2	1.21	2.32	0	0.53	0
Spint3	NA	NA	NA	2.64	NA
Spint4	NA	NA	NA	0.15	NA
Spire1	0	NA	3.09	5.35	0.17
Spire2	0.5	NA	0.67	3.99	0.31
Spn	0.75	0.2	2.45	0.72	1.39
Spns1	NA	0	2.47	0.99	0
Spns2	NA	NA	0.06	1.56	0.94
Spns3	NA	NA	0.22	0.33	0
Spo11	3.41	2.36	0.99	0.54	2.57
Spocd1	NA	NA	NA	0.77	NA
Spock1	0	0.5	0.61	1.39	4.41
Spock2	0.42	1.74	1.85	4.53	0
Spock3	NA	0.02	0	2.78	0
Spon1	4.38	1.12	0.05	0.26	0
Spon2	0	2.73	0.8	0.37	1.46
Spop	2.63	1.01	0.52	0.05	0
Spopl	NA	NA	0.88	0.7	0
Spp1	0.81	4.6	3.11	0.56	0
Spp2	2.3	1.67	0.49	0.8	0.84
Sppl2a	NA	NA	1.89	0	NA
Sppl2b	NA	0.12	0.05	2.21	NA
Sppl2c	NA	NA	NA	1.25	NA
Sppl3	0	NA	0	0.82	NA
Spr	0.14	2.45	1.39	1.43	2.77
Spred1	0	NA	2.05	1.16	0.07
Spred2	0.54	0.89	0.93	2.71	2.22
Spred3	NA	NA	2.63	0.8	NA

Sprn	0.24	NA	2.35	2.36	0
Sprr1a	0.19	0.35	0	2.64	4.69
Sprr1b	9.89	0.49	0	1.25	0
Sprr2a	6	NA	NA	NA	NA
Sprr2b	0	NA	NA	4.52	NA
Sprr2d	0	NA	NA	0.49	NA
Sprr2e	0.57	NA	NA	2.39	NA
Sprr2f	1.15	NA	NA	1.29	NA
Sprr2g	2.51	NA	NA	1.85	NA
Sprr3	5.8	0.11	0.91	0.55	0
Sprr4	1.8	NA	NA	2.79	NA
Sprtn	NA	NA	1.48	NA	NA
Spry1	0.65	1.75	2.25	3.23	NA
Spry2	0	0.9	1.58	2.08	0
Spry3	NA	NA	1.98	2.18	3.18
Spry4	0	0.72	1.58	2.32	1.12
Spryd3	NA	NA	1.7	2.7	0.55
Spryd4	NA	NA	0.26	3.23	1.47
Spryd7	NA	1.41	2.7	1.35	NA
Spsb1	NA	0.94	0.98	0.75	0
Spsb2	NA	NA	1.75	1.06	0
Spsb3	NA	2.44	2.32	0.55	0
Spsb4	NA	NA	1.59	4.75	9.07
Spta1	NA	1.86	1.52	0.14	NA
Sptan1	NA	0	0.65	0	NA
Sptb	NA	1.44	0.36	1.8	NA
Sptbn1	NA	0.39	1.81	0	NA
Sptbn2	NA	2.61	0.47	0.7	NA
Sptbn4	NA	2.21	1.4	2.76	NA
Sptlc1	0.29	0.22	0.36	0.48	1.15
Sptlc2	0	0	0.32	0.83	0
Sptlc3	NA	0.57	1.92	1	0
Sptssa	NA	2.81	1.64	0.92	NA
Sptssb	NA	NA	0	2.46	NA
Spty2d1	NA	NA	1.1	0.52	0.83
Spz1	1.24	NA	0.88	2.37	0
Sqle	0.32	0.55	3.11	0.12	1.99
Sqrdl	0.5	0	1.62	2.09	0
Sqstm1	1.36	1.2	1.38	0.28	0.5
Sra1	0.71	NA	2.8	2.46	1.54
Srbd1	NA	2.65	1.97	0.81	1.94

Src	0	0.58	1.87	1.22	1.88
Srcap	NA	0.24	2.2	0	0.08
Srcin1	NA	NA	0	1.37	NA
Srcrb4d	0.22	NA	1.88	1.11	1.02
Srd5a1	0.98	2.19	0.35	1.49	0
Srd5a2	0.88	3.05	1.98	0.83	0
Srd5a2l	0.6	NA	NA	NA	NA
Srd5a2l2	2.22	NA	NA	NA	NA
Srd5a3	NA	0.6	1.05	0.16	0.56
Srebf1	3.43	0	0.25	0	0.7
Srebf2	2.69	0.27	2.54	0.93	0
Srek1	NA	0.28	1.03	0.53	NA
Srek1ip1	NA	1.59	1.25	1.28	NA
Srf	0.35	1.06	2.88	1.81	0
Srfbp1	NA	NA	1.27	0.35	1.99
Srgap1	3.47	NA	1.06	0	3.25
Srgap2	0.14	0.22	1.1	1.42	1.66
Srgap3	NA	1	0.94	0	2
Srgn	NA	0.01	1.1	3.11	0
Sri	0.41	0.02	0.21	0.81	3.31
Srl	NA	NA	0.55	0.83	0.13
Srm	0.28	2.35	2.17	0.45	0
Srms	0	NA	1.35	0.45	1.54
Srp14	NA	1.4	0.87	0.1	0
Srp19	0.71	0	1.49	0.38	0
Srp54	0.76	NA	NA	NA	NA
Srp68	0	NA	0.78	0.47	0
Srp72	0	1.33	2.91	0.2	1.87
Srp9	1.02	1.19	1.6	0.35	0
Srpk1	0.22	3.77	2.41	0.89	0
Srpk2	0.1	0.24	0.8	NA	1.33
Srpk3	NA	1.82	2.45	0	1.3
Srpr	1.79	1.59	1.18	0.08	0.23
Srprb	0	0	0.23	0.85	0
Srpx	0.12	1.71	3.07	6.47	2.43
Srpx2	0.43	1.7	2.09	0.41	2.45
Srr	0.06	0	0.52	0.31	0.75
Srrd	NA	0.95	1.05	0.9	0.18
Srrm1	0.05	1.14	0.8	0	1.38
Srrm2	0.07	0	0.95	4.83	1.33
Srrm3	NA	NA	0	0.6	NA

Srrm4	NA	NA	0	0.29	NA
Srrt	NA	0.69	0.38	0	0.02
Srsf1	NA	0	0.59	0.95	NA
Srsf10	NA	0.02	2.08	0.58	NA
Srsf11	NA	0.3	1.43	0	NA
Srsf12	NA	NA	1.51	0.54	NA
Srsf2	NA	1.3	1.06	0	NA
Srsf3	NA	1.36	0.02	0.71	NA
Srsf4	NA	1.21	2.37	0.64	NA
Srsf5	NA	1.66	0.25	NA	NA
Srsf6	NA	0.15	1.71	0.92	NA
Srsf7	NA	0	0.89	0.34	NA
Srsf9	NA	2.34	2.13	1.74	NA
Srxn1	NA	NA	0.88	1.09	2.01
Sry	3.42	1.03	0.05	0.14	0
Ss18	0.27	0.64	1.51	6.46	0
Ss18l1	0.46	0.37	0.78	0.89	0
Ssb	0.29	1.25	1.68	0	0
Ssbp1	0.67	0.76	0.58	0.23	0
Ssbp2	0	0	0.93	1.37	0
Ssbp3	0	0.04	0.64	1.28	0
Ssbp4	0.58	NA	4.98	0.19	1.73
Ssc5d	NA	NA	0.9	0.8	NA
Ssfa2	1.85	0.13	1.17	1.62	0
Ssh1	0	2.22	0.8	0	0
Ssh2	0	NA	2.31	0.96	0
Ssh3	0.82	1.23	0.81	2.73	1.02
Ssmem1	NA	NA	1.52	3.37	NA
Ssna1	NA	1.93	1.89	1.89	0
Sspn	2.26	0.07	0	2.07	0.51
Sspo	NA	NA	1.1	1.98	0
Ssr1	0	0.81	1.3	0.57	0
Ssr2	1.58	0	1.09	4.19	0.64
Ssr3	0	1.41	1.68	0.87	0
Ssr4	0.67	0.73	4.25	2.43	0
Ssrp1	0.46	0	0.02	1.34	0
Sssca1	0.95	1.54	0.48	4.38	1.78
Sst	1.26	0.46	0	3.49	0
Sstr1	1.54	2.28	0.61	2.46	0
Sstr2	0	2.15	2.12	1.49	5.15
Sstr3	0.52	2.91	2.23	0	0

Sstr4	0	1.09	0.5	1.7	1.39
Sstr5	NA	0	0.66	0.85	4.41
Ssu72	NA	NA	0.62	0.51	1.41
Ssx2ip	1.11	1.67	0.91	1.97	0.05
Ssx9	0.36	NA	NA	NA	NA
St13	0	1.41	0.19	NA	2.68
St14	1.17	1.59	2.24	1.09	0
St18	0.25	0.48	1.48	3.58	1.58
St3gal1	NA	0.96	0.06	0.76	1.08
St3gal2	NA	2.78	0.87	2.17	3.37
St3gal3	NA	NA	0.77	0.91	2.03
St3gal4	NA	0	0.39	2.41	0
St3gal5	NA	0.37	3.03	0.5	0
St3gal6	NA	0	0.75	NA	1.76
St5	0.29	1.47	2.2	0.94	1.73
St6gal1	1.27	0.02	2.57	2.28	0
St6gal2	0	NA	0.53	1.59	0
St6galnac1	NA	NA	0	0	3.05
St6galnac2	NA	0.76	1.07	NA	0
St6galnac3	NA	NA	0	3.06	2.81
St6galnac4	NA	1.86	0.88	2.18	0.63
St6galnac5	NA	1.71	0.76	0.4	0
St6galnac6	NA	NA	1.4	0.24	4.59
St7	0.79	0.21	1.94	NA	0.51
St7l	0	1.37	0.56	0.84	0.83
St8sia1	NA	0.17	1.84	0.38	1.12
St8sia2	NA	1.46	0.4	7.89	0
St8sia3	NA	0.85	0.95	3.83	1.78
St8sia4	NA	0.97	1.32	0.65	1.8
St8sia5	NA	1.78	0.17	2	0
St8sia6	NA	NA	NA	1.52	NA
Stab1	0.51	0.15	4.58	0.03	0
Stab2	0.68	0.38	1.26	2.91	2.89
Stac	0.96	3.49	1.01	0.71	2.62
Stac2	1.24	NA	1.01	2.34	0.64
Stac3	NA	NA	1.72	0.3	1.72
Stag1	0	1.34	0.68	0.28	0.23
Stag2	1.61	2.68	0.56	0.2	0
Stag3	0.73	0	2.56	3.23	3.77
Stam	0.37	0.18	1.97	0.19	0
Stam2	0.09	0	0.63	0.55	0

Stambp	0.78	1.41	1.4	1.38	0
Stambpl1	NA	1.44	1.94	2.85	1.11
Stap1	NA	0.34	1.41	0	NA
Stap2	NA	1.05	1.12	0.73	0
Star	0	0.1	1.44	4.35	0.68
Stard10	0.4	NA	0.1	1.39	3.14
Stard13	2.97	0.61	0.67	3.17	0
Stard3	0.62	2.23	0.07	1	3.45
Stard3nl	0.43	NA	3.95	0.84	2.24
Stard4	1.25	NA	0.41	1.26	0
Stard5	0.3	0	0	0.45	2.24
Stard6	0.19	NA	1.69	1.26	NA
Stard7	0.88	1.67	0.8	1.17	3.61
Stard8	0.95	1.41	1.1	0	5.37
Stard9	NA	NA	1.43	0	1.63
Stat1	0.41	0.61	1.97	0	0.18
Stat2	0	0	3.98	1.02	0
Stat3	0.3	2.94	2.11	0	0
Stat4	1.81	0	1.89	3.19	2.32
Stat5a	0.03	1.29	0.55	1.62	4.17
Stat5b	0	0.67	0.43	1.36	1.8
Stat6	1	2.48	0.03	1.24	4.46
Statip1	0	NA	NA	NA	NA
Stau1	0.33	0.14	1.33	1.6	0
Stau2	0	1.46	1.5	2.67	0
Stbd1	NA	NA	3.15	0.19	0.59
Stc1	0	1.17	1.23	0.22	1.94
Stc2	0	0.86	0.18	0	2.51
Stch	0.74	NA	NA	NA	NA
Steap1	NA	1.08	0.01	0.39	2.13
Steap2	3.8	NA	1.04	1.54	0.18
Steap3	NA	0	1.58	1.43	0
Steap4	NA	0.53	0.87	2.11	0
Stil	NA	0.63	4.63	0.38	0.05
Stim1	0.59	0	2.55	8.78	0.34
Stim2	0.67	NA	0.81	1.23	4.19
Stip1	0.38	1.3	0.27	0.47	0
Stk10	1.45	0.1	0.54	1.9	0
Stk11	0	0.95	1.13	1.23	2.13
Stk11ip	1	NA	1.66	0.44	NA
Stk16	1.04	0.2	0.6	NA	0

Stk17b	0.56	2.04	0.76	0	0
Stk19	0.2	1.21	0.8	0	0
Stk24	0.36	0.17	2.05	1.63	0.06
Stk25	0.66	0	0.98	NA	3.46
Stk3	0.52	1.47	1.03	0.8	1.14
Stk31	0.69	NA	2.62	0	1.9
Stk32a	0.71	NA	2.13	2.53	NA
Stk32b	0.99	0	1.34	0	2.11
Stk32c	2.99	NA	0.19	1.97	0
Stk33	0.52	NA	1.24	2.37	2.54
Stk35	0	NA	0.91	5.27	1.53
Stk36	NA	NA	0.22	4.3	1.04
Stk38	0.92	0.03	1.7	0.69	0.03
Stk38l	3.26	1.95	1.62	1.85	0
Stk39	0	2.04	0.39	1.29	3.4
Stk4	1.46	1.45	1.59	0.78	1.04
Stk40	NA	NA	1.49	0.79	0
Stmn1	0.36	0.35	2.98	2.4	1.33
Stmn2	0	1	1.41	0.07	1.75
Stmn3	0	2.47	2.28	2.09	3.23
Stmn4	0.85	2.07	0.7	2.96	2.14
Stom	NA	3.65	1	2.37	3.94
Stoml1	0.71	2.52	0.66	3.82	0
Stoml2	0	2.91	0	0.65	4.9
Stoml3	0	NA	1.29	1.98	NA
Ston1	NA	0.08	0.56	NA	0
Ston2	NA	NA	1.18	1.2	0
Stox1	NA	NA	0.44	2.91	1.85
Stox2	NA	NA	0.03	0.61	1.51
Stpg1	NA	NA	0.51	0.97	NA
Stpg2	NA	NA	NA	1.41	NA
Stra13	0.66	1.53	2.88	0	0
Stra6	0.23	2.03	0.93	0.22	0
Stra8	2.71	NA	NA	1.27	NA
Strada	NA	1.28	2.07	1.53	0
Stradb	NA	NA	0.21	0	0
Strap	0.19	1.4	3.4	0.27	0
Strbp	0	NA	0.67	0	0.85
Strc	0	NA	1.05	2.53	0
Strip1	NA	NA	0.81	0	NA
Strip2	NA	NA	0.83	0.8	NA

Strn	0.14	0.73	0.83	2.38	0
Strn3	1.48	0.47	0.77	0.36	1.12
Strn4	2.6	0.32	0.08	0.98	0
Sts	1.89	2.63	0.02	0.76	1.83
Stt3a	NA	1.88	2.96	0.11	0
Stt3b	NA	NA	1.63	0	1
Stub1	0.34	0	1.82	NA	0
Stx11	0	2.34	1.59	0.6	0
Stx12	0.17	0.57	0.64	0.97	0
Stx16	0.5	3.03	1.55	0	0.52
Stx17	0.26	0.87	0.36	1.03	0.79
Stx18	0.77	0.59	2.6	1.12	3.15
Stx19	NA	NA	1.64	2.94	NA
Stx1a	0	0	0	0.32	0
Stx1b	NA	NA	0.81	1.49	2.59
Stx1b2	0.85	NA	NA	NA	NA
Stx2	NA	0.46	7.83	0.67	0
Stx3	0	0.18	0.47	1.54	3.25
Stx5a	0	NA	NA	NA	NA
Stx6	1.28	0.59	1.56	NA	2.18
Stx7	0.35	0.1	1.05	0	1.59
Stx8	0.49	0	1.31	1.21	0
Stxbp1	0.06	1.7	0.52	0	0
Stxbp2	0	0.22	0.63	1.53	0
Stxbp3	1.09	NA	NA	NA	NA
Stxbp4	0.78	NA	0.38	0	0.77
Stxbp5	1.81	NA	1.39	0.56	1.65
Stxbp5l	NA	2.3	0.2	1.81	0
Stxbp6	3.57	0.82	0.35	1.61	3.51
Styk1	NA	1.14	2.85	0.18	1.94
Styx	NA	NA	1.69	0.71	5.01
Styxl1	NA	1.07	2.24	1.41	0
Sub1	NA	1.03	0.88	0.88	1.83
Sucla2	0	1.04	0.99	1.18	1.73
Suclg1	0	4.67	1.55	0.84	0.7
Suclg2	0	0	2.76	0.76	2.81
Sucnr1	0.9	NA	0.44	0.73	0
Suco	NA	0.95	0.87	0.66	NA
Suds3	NA	NA	1.59	1.62	0
Sufu	0	NA	0	2.05	0.51
Sugp1	NA	0	0.59	0.59	NA

Sugp2	NA	0	1.35	1.41	NA
Sugt1	1.54	NA	1.96	0.17	1.47
Suhw2	3.85	NA	NA	NA	NA
Suhw3	3.04	NA	NA	NA	NA
Suhw4	0	NA	NA	NA	NA
Sulf1	0.41	0.47	0.03	0.34	0
Sulf2	0.66	NA	1.73	1.83	0
Sult1a1	0	2.13	2.28	1.77	0
Sult1b1	1.7	2.25	3.23	0.16	0
Sult1c1	0.97	NA	NA	NA	NA
Sult1c2	2.97	0	0.95	3.52	3.27
Sult1e1	0	1.29	0	3.09	1.24
Sult2a1	2.56	NA	NA	NA	NA
Sult2b1	0.5	0	0.71	1.33	1.43
Sult4a1	2.07	0.01	0.22	0.54	2.78
Sult6b1	NA	NA	NA	0	NA
Sumf1	0.86	NA	1.78	0	0
Sumf2	0.08	NA	0.91	0.01	9.04
Sumo1	0.53	0	2.86	0.64	1.63
Sumo2	1.71	1.64	1.33	1.05	NA
Sumo3	0	0.53	0.94	0	0
Sun1	NA	0	1.28	0	NA
Sun2	NA	0	0.58	0	NA
Sun3	NA	NA	1.45	NA	NA
Sun5	NA	NA	1.82	1.66	NA
Sunc1	2.59	NA	NA	NA	NA
Suox	0.45	2.72	3.36	1.91	5.33
Supt16h	0.24	NA	NA	NA	0
Supt3h	0.83	NA	NA	NA	1.6
Supt5h	1.2	NA	NA	NA	3.96
Supt6h	0.6	NA	NA	NA	0
Supt7l	NA	0	0.99	1.81	0
Supv3l1	0.44	1.43	0.26	0.9	1.88
Surb7	2.13	NA	NA	NA	NA
Surf1	0.19	3.01	1.51	1.43	1.06
Surf2	0.07	1.22	0	0.54	1.44
Surf4	2.11	NA	1.48	0	3.76
Surf5	0.05	NA	NA	NA	NA
Surf6	0	NA	3.15	1.4	0
Susd1	NA	NA	10.53	4.21	3.35
Susd2	3.34	NA	1.09	0	0

Susd3	NA	NA	1.77	1.46	0.71
Susd4	NA	0	1.39	1.23	0.84
Susd5	NA	0.95	1.3	NA	1.86
Suv39h1	0	0	1.3	2.17	2.6
Suv39h2	1.84	0.47	0.43	NA	0.23
Suv420h1	NA	1.15	2.01	0	1.41
Suv420h2	0.1	NA	3.66	0.35	2.65
Suz12	NA	0.97	0.43	0.83	0
Sv2a	0.89	0	1.71	0	0.11
Sv2b	0	1.9	1.06	0.4	3.66
Sv2c	0.46	0	0.67	0	0
Svep1	NA	1.4	0	0	0
Svil	0	1.64	1.65	0	0
Svip	NA	NA	1.25	1.22	0
Svop	NA	NA	3.56	5.54	0
Svopl	NA	NA	3.69	0.98	NA
Swap70	0.56	0.12	0.71	0.72	0
Swi5	NA	NA	1.3	1.69	NA
Swsap1	NA	NA	0.75	1.38	NA
Swt1	NA	NA	1.27	0.52	NA
Syap1	0	NA	1.08	2.04	0
Sybl1	0	NA	NA	NA	NA
Sybu	NA	1.98	2.65	1.42	NA
Syce1	NA	NA	0	NA	2.21
Syce1l	NA	0.69	0.94	0.88	NA
Syce2	NA	NA	3.18	0.97	0
Syce3	NA	NA	0	NA	NA
Sycn	2.89	NA	0	4.03	2.2
Sycp1	0	0.97	4.69	0.34	1.95
Sycp2	NA	0.23	1.35	1.75	0
Sycp2l	NA	NA	NA	NA	1.28
Sycp3	2.43	NA	0	NA	0.82
Syde1	NA	1.29	1.15	2.97	6.2
Syde2	NA	NA	2.91	2.93	NA
Syf2	NA	4.45	0.95	1.42	0
Syk	0.57	0.87	1.97	2.63	NA
Sympk	1.65	0	1.09	1.54	0.52
Syn1	1.92	0.59	2.68	2.87	1.91
Syn2	0.11	0.77	0.34	0.98	1.11
Syn3	0.7	0.29	0	2.54	1.38
Sync	NA	0.84	0.93	0	1.41

Syncrip	0	1.36	0.49	0	1.98
Syndig1	NA	1.67	13.71	0.71	NA
Syndig1l	NA	NA	0	0.99	NA
Syne1	0.32	0.62	1.04	0	0.31
Syne2	NA	0	0.23	0	1.1
Syne3	NA	NA	0	1.42	NA
Syne4	NA	NA	2.63	0.62	NA
Syngr1	0.49	0.58	1.67	0.64	0.78
Syngr2	1.38	1.79	0.08	1.77	1.26
Syngr3	1.76	0	0.76	1.16	0.23
Syngr4	1.51	2.21	0.47	3.83	0.17
Synj1	0.6	1.34	1.29	0	0
Synj2	2.23	0.43	0.77	1.55	0.02
Synj2bp	0	0	0.92	0.97	1.12
Synm	NA	0.56	1.47	0	0.11
Synpo	0.82	2.38	1.17	0.57	0.97
Synpo2	0.19	NA	0.72	1.1	0
Synpo2l	0	0.28	0.03	2.9	0.93
Synpr	0.21	NA	1.1	0.4	1.33
Synrg	NA	0	1.84	0.22	0
Syp	2.25	0	1.59	1.33	1.86
Sypl2	NA	NA	0.57	1.3	1.81
Sys1	NA	NA	1.32	NA	0
Syt1	0.75	0.79	2.18	0	0.64
Syt10	0	NA	NA	1.49	NA
Syt11	0	1.86	1.42	0	0
Syt12	3.2	1.71	0.47	2.57	0
Syt13	1.77	2.53	0.21	2.5	1.46
Syt14	0.94	NA	NA	0.6	NA
Syt15	0.19	NA	NA	1.28	NA
Syt16	NA	NA	1.69	0	NA
Syt17	NA	1.08	0	2.23	1.3
Syt2	0.11	0.52	0.17	0.67	0
Syt3	3.9	NA	0.9	1.32	0
Syt4	0.73	NA	1.6	1.58	0.99
Syt5	0.92	1.27	1.44	2.11	0
Syt6	0.32	NA	0.27	0.85	0
Syt7	0.07	NA	0	0	0
Syt8	1.71	NA	1.97	3.3	5.76
Syt9	1.73	NA	0.9	0	4.56
Sytl1	16.24	NA	2.35	0.74	0

Sytl2	0.02	0	0.98	2.42	1.35
Sytl3	1.93	NA	0.87	0.62	0
Sytl4	0.03	NA	0.85	0	0
Sytl5	1.05	NA	NA	1.17	NA
Syvn1	0.54	NA	1.07	0	0
Szrd1	NA	0.65	0	3.49	NA
Szt2	NA	0	0.51	0.73	NA
T	2.14	3.47	1.94	0.54	1.08
Taar1	NA	NA	4.32	0	NA
Taar2	NA	NA	NA	0	NA
Taar3	NA	NA	NA	0	NA
Taar5	NA	NA	NA	1.45	NA
Taar6	NA	NA	NA	1.52	NA
Taar9	NA	NA	NA	1.21	NA
Tab1	NA	0.74	0.72	0	NA
Tab2	NA	0.46	3.2	1.33	NA
Tab3	NA	NA	2.58	0.41	NA
Tac1	0	1.6	1.32	3.32	0
Tac4	0.68	NA	4.43	1.47	NA
Tacc1	NA	0.22	2.05	0.43	0
Tacc2	0.3	0.87	3.75	1.94	0
Tacc3	0	0.78	2.68	0.18	2.81
Taco1	NA	NA	NA	0.71	NA
Tacr1	0.63	0	2.07	2.8	0
Tacr2	0.37	0.33	0	0.72	2.35
Tacr3	0	1.14	0.54	2.52	4.02
Tacstd1	1.11	NA	NA	NA	NA
Tacstd2	1.48	0.19	0.65	2.51	0
Tada1	NA	NA	0	0.28	NA
Tada2a	NA	1.31	1.13	0.86	NA
Tada2b	NA	NA	1.58	3.13	NA
Tada2l	0	NA	NA	NA	NA
Tada3	NA	0.5	0.97	0.15	NA
Tada3l	0	NA	NA	NA	NA
Taf1	NA	0.86	0.85	0.3	0.76
Taf10	0.95	0.57	4.02	0.11	0.56
Taf11	0	0.76	0.33	2.11	8.52
Taf12	0.38	0	2.36	0.1	1.84
Taf13	0.39	0.35	1.89	0.19	0
Taf15	0	0	0.71	0.11	1.98
Taf1a	0.54	0.61	2.64	0.21	1.65

Taf1b	18.04	1.45	0.76	0.49	2.7
Taf1c	0.87	0.02	0.26	NA	0
Taf1d	NA	0.69	1.63	NA	1.98
Taf2	4.36	0.25	1.49	0	3.08
Taf3	0.28	NA	2.22	1.4	0
Taf4b	0.98	1.54	1.63	0.62	0.17
Taf5	0	2.38	1.1	1.1	2.39
Taf5l	0	1.21	3.89	3.1	0
Taf6	0.27	0	0	2.98	0
Taf6l	0	0.51	2.99	0	0
Taf7	0.41	0	0.65	1.13	1.67
Taf7l	0	0.45	0.44	0.75	1.82
Taf8	NA	NA	1.42	5.02	1.91
Taf9	0	0.75	1.4	0.77	0
Taf9b	NA	0.65	1.36	1.96	0.8
Tagap	0.11	NA	NA	3.11	NA
Tagln	0.53	0.99	2.16	NA	1.65
Tagln2	0.72	0	0.24	3.07	0
Tagln3	2.05	0.69	0.29	0.68	0
Tal1	1.29	0.91	2.1	0.76	3.03
Tal2	1.62	NA	0.54	0	0
Taldo1	0.27	1.55	2.47	1.96	0.66
Tamm41	NA	NA	1.75	2.42	NA
Tanc1	NA	NA	1.55	1.77	0
Tanc2	NA	0.74	1.83	0	1.47
Tango2	NA	NA	3.66	0.49	NA
Tango6	NA	NA	0.28	2.41	NA
Tank	0.17	0.07	2	0	0
Taok1	NA	0.94	0.2	0.11	0.71
Taok2	NA	0.99	0.09	0	0.22
Taok3	NA	0.04	0.73	0	1.38
Tap1	0	1.59	0.51	0	0
Tap2	0	0.84	0.75	0	0.46
Tapbp	0.35	0.99	1.25	0.39	0
Tapbpl	NA	2.12	1.12	0.78	5.35
Tapt1	NA	1.54	2.01	0.26	1.24
Tarbp2	0	0.98	1.73	1.15	0.97
Tardbp	0.02	0	1.42	0.68	2.18
Tars	0.54	1.34	0.54	0.9	1.62
Tars2	NA	0.62	0.11	4.4	0.62
Tarsl2	NA	NA	0.33	0.65	0

Tas1r1	2.56	NA	0	2.9	NA
Tas1r2	0.22	NA	0.56	0.62	NA
Tas1r3	NA	NA	NA	0	NA
Tasp1	NA	2.35	0	1.27	2.46
Tat	2	1.58	1.79	0	1.56
Tatdn1	2	NA	1.83	NA	1.84
Tatdn2	NA	1.24	1.38	3.37	3.83
Tatdn3	NA	NA	0.31	NA	0
Tax1bp1	2	0.52	1.03	1.29	0.89
Tax1bp3	0	1.79	0.43	1.06	0
Taz	0.1	1.76	0.82	0.81	1.6
Tbata	NA	NA	1.88	0.31	NA
Tbc1d1	0.25	0.61	0.48	0.99	0.85
Tbc1d10a	NA	NA	1.59	1.54	1.49
Tbc1d10b	NA	1.65	0	2.16	1.48
Tbc1d10c	NA	NA	1.2	0.69	0.06
Tbc1d12	0.96	0.98	0.4	1.49	2.73
Tbc1d13	0.19	1.22	0.38	0	0
Tbc1d14	NA	NA	2.12	1.99	0
Tbc1d15	5.85	0	1.31	1.58	0
Tbc1d16	NA	1.8	1.7	0.31	1.09
Tbc1d17	0	0	2.16	0	2.25
Tbc1d19	NA	1.61	2.03	0	0
Tbc1d2	0.25	0.06	3.53	4.07	NA
Tbc1d20	NA	NA	0.88	0	0.22
Tbc1d21	NA	NA	0	0.94	2.47
Tbc1d22a	NA	0	1.88	0	0
Tbc1d22b	NA	0	0.83	8.79	NA
Tbc1d23	NA	NA	1.89	0.66	2.65
Tbc1d24	NA	NA	2.39	1.76	0
Tbc1d25	NA	NA	1.71	1.49	0.04
Tbc1d2b	NA	1.46	3.23	2.85	0
Tbc1d30	NA	1.59	0.11	0	4.09
Tbc1d4	0.59	0	2.63	2.22	3.5
Tbc1d5	1.32	0	0.49	0.38	0.73
Tbc1d7	0.51	NA	0	1.8	0.14
Tbc1d8	0	0	2.23	0.01	0.38
Tbc1d8b	NA	1.57	1.34	0	0
Tbc1d9	NA	2.34	0.75	0	0
Tbc1d9b	NA	1.63	0.51	0	1.44
Tbca	0.35	0	1.74	0	0

Tbcb	NA	0.01	2.28	NA	0
Tbcc	0.25	1.64	0.59	0.64	0
Tbccd1	NA	0	2.83	0	1.07
Tbcd	0.73	0.36	1.22	0	0.95
Tbce	0	2.64	1.28	0.69	3.44
Tbcel	NA	NA	0.78	0	0
Tbck	NA	NA	0	0.32	0
Tbk1	0	0.03	0.57	0.78	0
Tbkbp1	NA	0	0	0.42	0
Tbl1x	0	0.55	1.01	0.74	0.04
Tbl1xr1	NA	0.7	0.97	0.41	2.4
Tbl2	0.18	0.59	0.36	0.2	0.75
Tbl3	0.91	1.65	0.25	0	1.09
Tbn	1.63	NA	NA	NA	NA
Tbp	0.42	0.77	1.28	12.56	0
Tbpl1	1.35	1.52	1.94	NA	0
Tbpl2	NA	NA	NA	1.18	NA
Tbr1	NA	1.25	1.63	0	0
Tbrg1	0.34	NA	0.77	2.57	2.99
Tbrg4	0.54	0.97	0.18	1.82	NA
Tbx1	0.61	0.49	0.08	0.88	9.95
Tbx10	0.82	NA	NA	1.4	NA
Tbx15	0.84	NA	0.62	1.35	2.09
Tbx18	0.81	NA	1.02	0.78	2.66
Tbx19	0	3.02	0.99	0.6	4.28
Tbx2	0.05	0.82	1.15	0.33	0
Tbx20	NA	NA	1.03	1.22	NA
Tbx21	3.47	0.08	1.44	0.95	2.32
Tbx22	0.45	NA	1.69	2.86	0.06
Tbx3	0	0.94	0.59	1.99	1.19
Tbx4	1.21	0.74	0.19	0.63	0
Tbx5	0.05	0.68	0.19	2.42	0
Tbx6	0	1.79	0.49	0.84	11.6
Tbxa2r	0.39	1.28	2.8	1.25	2.18
Tbxas1	0.09	3.22	1.31	3.33	0
Tc2n	NA	NA	0.5	1.28	0
Tcaim	NA	NA	0.39	0.78	NA
Tcam1	NA	NA	NA	NA	0
Tcap	3.03	0.02	1.26	1.24	0
Tcea1	0.54	0.52	4.12	1.88	7
Tcea2	2.56	0	0.88	0	0.53

Tcea3	0.77	NA	2.37	2.28	0
Tceal1	0.46	0.66	1.91	1.26	0
Tceal3	NA	NA	0.94	0.41	NA
Tceal5	3.43	NA	NA	1.45	NA
Tceal6	NA	NA	NA	4.91	NA
Tceal7	0	NA	1.95	NA	2.21
Tceal8	0	NA	2.42	0	NA
Tceanc	NA	NA	0.97	1.79	NA
Tceanc2	NA	NA	0.61	1.21	NA
Tceb1	0	3.29	2.81	0.71	1.54
Tceb2	0	1.06	2.94	0	0.44
Tceb3	0.59	0.42	1.7	1.1	1.58
Tcerg1	0.41	1.65	0.21	3.05	0.99
Tcerg1l	0	NA	0	2.06	0.77
Tcf1	0	NA	NA	NA	NA
Tcf12	0.31	0.23	1.73	2.16	1.12
Tcf15	3.18	3.01	2.99	1.19	0
Tcf19	0.64	NA	2.44	1.01	1.29
Tcf2	0.49	NA	NA	NA	NA
Tcf20	1.18	0.23	0.92	1.05	0
Tcf21	0	1.19	0.91	0.82	0
Tcf23	2.38	NA	NA	0.83	NA
Tcf25	NA	0.05	0.48	0.12	0.77
Tcf3	2.04	1.97	1.98	1.35	0.91
Tcf4	0.25	0.74	0.75	0.75	0
Tcf7	0.9	0	0.47	0.28	2.66
Tcf7l1	NA	1.53	1.15	1.19	1.81
Tcf7l2	0.34	0	0	0.38	1.42
Tcfl5	1.52	2.76	0.14	NA	4.26
Tchh	NA	0.71	1.98	1.93	1.36
Tchhl1	NA	NA	NA	1.09	NA
Tchp	NA	NA	2.12	1.67	3.66
Tcirg1	0.75	0.47	1.83	0	3.29
Tcn2	1.17	0	2.06	0	0
Tcof1	0.8	0.89	1.03	1.83	0.09
Tcp1	0.1	2	0.88	0.55	1.87
Tcp10	0	NA	NA	NA	NA
Tcp11	0	1.79	2.23	0.1	2.92
Tcp11l1	NA	NA	NA	1.69	NA
Tcp11l2	NA	NA	0.62	0.19	NA
Tcta	0.14	1.58	1.32	1.78	0.05

Tcte1	NA	NA	2.46	NA	0
Tcte3	0.37	NA	NA	NA	NA
Tctex1d1	NA	NA	1.12	0.55	NA
Tctex1d2	NA	NA	2.28	0	2.84
Tctex1d4	NA	NA	NA	1.08	NA
Tctn1	NA	NA	NA	0.96	NA
Tctn2	NA	3.08	0	0.58	1.72
Tctn3	NA	0	1.8	1.1	0
Tdg	0.31	0.99	2.18	NA	NA
Tdgf1	1.23	NA	NA	1.22	NA
Tdh	0.73	NA	0.15	0.98	NA
Tdo2	1.89	1.79	0.25	0	2.86
Tdp1	NA	0	0.44	1.56	2.07
Tdp2	NA	3.16	0.62	0.74	NA
Tdrd1	0.99	0	1.5	0.61	0
Tdrd12	NA	1.77	2.82	1.93	1.8
Tdrd3	0.08	0	2.17	0.74	0
Tdrd5	NA	NA	0	4.08	4.58
Tdrd6	1.96	NA	NA	1.42	NA
Tdrd7	1.28	0	1.47	0	0
Tdrd9	0.18	NA	0.73	1.68	0
Tdrkh	0	0	2.71	0.02	1.27
Tead1	0.65	1.04	1.16	3.37	0
Tead2	0	NA	2.48	3.83	5.98
Tead3	0.99	0	1.42	0	0
Tead4	0.44	0.58	0.42	4.08	5.14
Tec	0.56	4.26	0.91	0.02	2.54
Tecpr1	NA	NA	3.63	0.76	1.52
Tecpr2	NA	0.89	1.27	3.47	8.51
Tecr	NA	0	0.79	2.31	3.64
Tecrl	NA	NA	0	0.4	0
Tecta	0.28	0.47	2.2	1.29	0
Tectb	0.88	NA	1.5	1.82	NA
Teddm1	NA	NA	NA	1.04	NA
Tef	0.46	0.97	1.52	1.69	0.06
Tefm	NA	1.47	2.19	0.78	NA
Tegt	0.54	NA	NA	NA	NA
Tek	2.41	0.62	0.74	0	1.08
Tekt1	1.56	NA	1.19	2.37	0
Tekt2	1.22	2.69	0	0.14	4.36
Tekt3	0.25	NA	1.14	0.44	0

Tekt4	NA	NA	NA	0.63	NA
Tekt5	NA	NA	1.26	0.51	NA
Telo2	NA	0	1.18	1.57	0
Ten1	NA	NA	1.68	1.61	NA
Tenc1	0.07	1.18	2.89	0.4	3.39
Tenm1	NA	0	0	0.66	NA
Tenm2	NA	NA	0.82	0.01	NA
Tenm3	NA	1.69	0.93	0	NA
Tenm4	NA	0.23	6.7	0	NA
Tenr	0.89	NA	NA	NA	NA
Tep1	1.22	1.38	2.29	0	0.11
Tepp	NA	NA	0.3	1.94	1.57
Terc	1.77	NA	NA	0.16	NA
Terf1	2.33	2.16	1.18	0.96	2.42
Terf2	0	0	0.59	3.01	0
Terf2ip	0	0.95	1.08	NA	0
Tert	1.41	1.27	0.65	2.81	1.73
Tes	1.2	1.01	0.76	0.27	0.97
Tesc	0	0	0.3	0.26	NA
Tesk1	1.07	0.2	2.19	2.03	0.05
Tesk2	0.99	0.15	2.99	1.31	0
Tespa1	NA	0	0.5	0.82	NA
Tessp2	2.19	NA	NA	NA	NA
Tet1	NA	NA	0	1.18	0
Tet2	NA	NA	1.42	1.25	0.53
Tet3	NA	0.4	2.05	1.13	0
Tex10	0.11	0	1.56	3.2	0.18
Tex101	1.77	NA	0	0.58	0
Tex11	1.86	2.08	0.61	0	6.15
Tex12	1.46	1.5	3.38	1.32	0.62
Tex13a	NA	0.62	0.29	0.72	3.52
Tex14	0	1.33	0.07	0.48	6.02
Tex15	2.82	1.24	2.6	0	0.02
Tex2	0	0.13	0.83	0.71	0.38
Tex22	NA	NA	1.29	NA	NA
Tex26	NA	NA	1.5	2.68	NA
Tex261	0.46	1.01	1.65	0.71	2.7
Tex264	0.55	0.7	2.11	1.04	2.51
Tex28	NA	NA	NA	5.68	NA
Tex29	NA	NA	0.56	2.08	NA
Tex30	NA	1.65	1.09	0	NA

Tex33	NA	NA	1.37	1.85	NA
Tex35	NA	NA	4.16	0	NA
Tex36	NA	NA	1.34	3.17	NA
Tex37	NA	NA	0	2.95	NA
Tex38	NA	NA	1.36	3.22	NA
Tex40	NA	1.06	0.08	NA	NA
Tex9	0	NA	1.35	0.82	0.05
Tfam	0	1.57	1.62	0.62	1.06
Tfap2a	NA	0.11	1.06	0.95	NA
Tfap2b	NA	0.9	0.67	0.93	NA
Tfap2c	NA	0.17	0.33	0	NA
Tfap2d	NA	NA	4.69	3.62	NA
Tfap2e	NA	NA	0.3	1.91	NA
Tfap4	NA	1.45	2.59	2.02	NA
Tfb1m	0.22	0	0.58	0.74	0
Tfb2m	0.41	0.12	0.31	0.67	0
Tfcp2	NA	0	1.26	0.81	NA
Tfcp2l1	NA	0.83	0.8	0.36	NA
Tfdp1	0.89	1.7	0.94	0.21	0
Tfdp2	NA	2.14	0.47	0	1.02
Tfe3	NA	1.37	1.24	1.63	NA
Tfeb	NA	2.63	0.76	0.87	NA
Tfec	NA	0.06	1.08	0	NA
Tff1	0.54	1.43	0.33	1.22	3.62
Tff2	4.37	2.02	0	0.69	1.77
Tff3	0	2.57	3	1.96	3.37
Tfg	0.4	0.88	1.41	0.89	3.14
Tfip11	1.46	0	1.51	1.48	0
Tfpi	0.25	0	1.13	0	1.49
Tfpi2	0	1.43	3.29	0.55	5.85
Tfpt	1.67	0.01	1.06	1.29	2.56
Tfr2	NA	1.23	0.63	1.81	NA
Tfrc	0.71	1.05	2.24	0	2.36
Tg	NA	2.12	0.65	0.15	0
Tgds	NA	1.31	0.91	1.19	0
Tgfa	0.31	1.38	0.11	1.79	1.91
Tgfb1	0.78	0.07	2.51	1.52	1.97
Tgfb1i1	2.62	2.58	2.23	2.12	1.81
Tgfb2	1.64	2.42	1.28	2.8	0.89
Tgfb3	1.66	1.08	2.15	0.53	0
Tgfbi	1.32	3.44	0.08	0.74	0

Tgfbr1	0.55	0.07	2.83	3.77	0
Tgfbr2	0	0.61	2.07	NA	0.55
Tgfbr3	0.88	0	0.04	1.73	0
Tgfbrap1	0.71	0	2.66	3.38	0.85
Tgif1	NA	1.3	2.05	0.32	2.59
Tgif2	0	1.41	0.85	1.59	0.45
Tgm1	0.55	1.55	0	0.65	0
Tgm2	0.04	2.19	0.04	4.08	0.14
Tgm3	0.41	0.1	1.18	1.67	0
Tgm4	0.6	0.56	2.09	0	NA
Tgm5	0	1.78	0.2	0.26	0
Tgm6	1.65	NA	0	1.54	2.38
Tgm7	NA	NA	NA	1.26	NA
Tgoln2	4.11	0.81	0.63	2.09	NA
Tgs1	NA	0.24	1.15	0	0.05
Th	0.25	1.89	1.64	1.92	5.95
Th1l	0.39	NA	NA	NA	2.76
Thada	0	0	0.63	1.41	2.17
Thap1	0.36	0.85	1.6	1.06	1.79
Thap11	0.11	0.61	1.51	1.25	1.6
Thap2	NA	NA	1.02	0.07	1.09
Thap3	NA	2.87	1.58	0.97	2.43
Thap4	NA	0.89	0.16	0.97	1.28
Thap6	0.06	NA	0.48	1.67	0
Thap7	0.1	0.13	0.14	2.46	0
Thbd	0.52	1.57	0.54	0.14	0
Thbs1	0.15	0.81	2.8	1.99	0
Thbs2	0.61	0.53	3.26	0	0
Thbs3	0	2.48	1.53	0.65	0
Thbs4	1.98	1.86	1.9	0.9	0
Theg	0.33	2.53	0.95	0.91	0
Them2	0.2	NA	NA	NA	NA
Them4	NA	NA	1.55	1.21	0
Them5	NA	NA	0.97	0.78	NA
Them6	NA	0	1.05	0.53	NA
Themis	NA	NA	1.55	1.84	NA
Themis2	NA	1.01	3.87	0.85	NA
Thg1l	NA	1.14	0.7	1.07	2.9
Thnsl1	0.47	0.18	1.98	0.81	1.58
Thnsl2	NA	0.45	0.01	1.11	2.06
Thoc1	1.4	1.64	0.2	0.5	0

Thoc2	NA	2.2	1.29	0	3.01
Thoc3	0.08	NA	NA	NA	NA
Thoc4	1.1	NA	NA	NA	0
Thoc5	NA	2.21	1.55	0.65	0.96
Thoc6	NA	1.84	0	0	0
Thoc7	NA	0	1.84	0.22	NA
Thop1	0.12	1.27	0.05	2.36	0
Thpo	1.36	0.83	1.46	1.15	6.8
Thra	0.96	0.76	3.09	NA	1.52
Thrap1	1.88	NA	NA	NA	NA
Thrap2	0.54	NA	NA	NA	NA
Thrap3	1.07	0.77	0.61	1.93	0.62
Thrap4	0.27	NA	NA	NA	NA
Thrap5	2.27	NA	NA	NA	NA
Thrap6	1.05	NA	NA	NA	NA
Thrb	2.31	0.89	1.68	0	0
Thrsp	0	NA	0	0.43	1.89
Thsd1	0.83	NA	NA	1.61	NA
Thsd4	NA	1	0	1.09	0
Thsd7a	NA	0.31	0.85	0.17	0
Thsd7b	NA	NA	0.03	2.64	1.04
Thtpa	0.03	1.44	0.01	1	1.07
Thumpd1	0.62	0.8	1.74	1.29	0
Thumpd2	0	0.18	0.27	1.12	1.27
Thumpd3	NA	NA	1.65	1.14	0
Thy1	0.41	1.13	1.27	2.66	1.96
Thyn1	NA	1.11	1.46	0	0
Tia1	0.86	1.08	0.86	0.79	0
Tiaf1	1.65	NA	NA	NA	NA
Tial1	0.62	0.89	0.73	2.61	0.84
Tiam1	0.96	1.05	0	1.33	0
Tiam2	1.81	0.2	1.33	NA	0.7
Ticam1	NA	1.36	2.12	1.06	5.22
Ticam2	0.69	NA	NA	NA	NA
Ticrr	NA	NA	1.19	1.49	NA
Tie1	3.02	1.57	0.45	1.41	2.94
Tifa	NA	NA	1.9	0.6	0
Tifab	NA	NA	0	0.74	7
Tigd2	0.1	NA	2.15	0.15	2.51
Tigd3	0	NA	4.57	4.49	3.12
Tigd4	0.08	NA	NA	3.61	NA

Tigd5	1.42	NA	1.14	1.54	0.82
Tigit	NA	NA	NA	1.44	NA
Timd4	0.36	NA	3.01	0	NA
Timeless	0	0.88	2.07	0	1.85
Timm10	0.13	0.19	1.31	2.33	2.89
Timm10b	NA	2.27	1.39	1.93	NA
Timm13	NA	1.62	1.34	NA	2.1
Timm17a	0.46	0.71	0.9	0.97	0
Timm17b	0	2.06	0.57	1.5	0
Timm21	NA	NA	2.63	0.4	NA
Timm22	0	1.56	0	0.6	0
Timm23	0	0.34	1.09	0.08	NA
Timm44	0	1.43	2.38	0.9	0
Timm50	3.56	0.57	0.57	0.85	0.85
Timm8a	0.18	NA	NA	NA	NA
Timm8b	1.88	0	0.13	NA	3.98
Timm9	3.51	0	0.16	NA	1.67
Timmdc1	NA	NA	0.32	1.21	NA
Timp1	0.81	0.53	0.56	0.23	0
Timp2	0.39	1.59	1.74	1.67	1.08
Timp3	0	0.66	0.89	1.5	1.07
Timp4	0.31	0.34	0.63	0.49	1.45
Tinag	0.14	NA	0.08	0	0
Tinagl1	NA	1.38	0	3.08	0.41
Tinf2	0.41	0	0.68	0.12	1.27
Tiparp	0.03	0.12	1.6	2.65	0
Tipin	0	1.65	2.67	0.99	0
Tiprl	NA	1.82	1.77	3.26	2.06
Tirap	0.74	NA	0.98	1.6	2.08
Titf1	1.17	NA	NA	NA	NA
Tjap1	NA	2.76	0.72	3.55	0
Tjp1	19.16	0.4	0.84	0.15	2
Tjp2	0.4	0.39	0.99	4.06	0.63
Tjp3	1	1.62	3.96	NA	0
Tk1	0.03	2.65	2.18	2.36	0
Tk2	0	1.95	1.35	0.12	0.19
Tkt	0	2.15	0	0.68	1.22
Tktl1	1.01	0	0.22	0	1.63
Tktl2	NA	NA	4.28	6.16	0
Tlcd1	NA	NA	1.33	0.51	0
Tlcd2	NA	NA	0.66	1.69	NA

Tldc1	NA	0	0.65	0	NA
Tldc2	NA	NA	NA	3.2	NA
Tle1	0.47	0.27	0.9	4.32	0
Tle2	0.49	3.6	1.53	0.41	0
Tle3	0.35	2.44	0.9	0	0
Tle4	1.21	0.84	1.18	1	0
Tle6	1.1	1.26	1.54	1.56	3.58
Tlk1	0.03	1.35	1.55	0.83	0
Tlk2	0.34	0.59	0.68	0.92	0
Tll1	1	0	0	0.54	1.74
Tll2	0.23	1.23	1.6	0	3.53
Tln1	0.95	1.51	3.27	0	1.68
Tln2	0.51	0.61	1.28	0	2.3
Tloc1	1.14	NA	NA	NA	NA
Tlr1	0.49	0.93	2.7	0.9	2.15
Tlr2	0.24	0.3	1.51	2.45	0
Tlr3	0.46	0.54	2.21	0.02	0.19
Tlr4	2.47	0.65	0.35	4.12	0
Tlr5	0	0	0	0.12	0
Tlr6	0	0.36	1.57	2.6	0.33
Tlr7	0.27	1.17	2.12	0	2.22
Tlr8	1.02	0	2.82	0.92	3.13
Tlr9	2.02	NA	1.98	1.56	1.66
Tlx1	1.13	1.89	0.38	0.25	0
Tlx2	0.65	1.53	2.13	0.98	0
Tlx3	0	1.97	0.83	1.33	0
Tm2d1	NA	0.55	1.16	3.06	1.01
Tm2d2	NA	NA	1.13	0.79	2.22
Tm2d3	NA	1.28	2.49	NA	0
Tm4sf1	0	0.48	0.7	1.91	2.29
Tm4sf11	0	NA	NA	NA	NA
Tm4sf19	NA	NA	NA	0.99	NA
Tm4sf20	NA	0	1.98	0.51	0
Tm4sf4	0	1.68	1.1	0.89	1.24
Tm4sf5	0	1.86	2.45	3.05	9.27
Tm6sf1	9.35	1.13	0.93	0.29	3.47
Tm6sf2	0.55	2.15	1.14	1.59	0
Tm7sf2	0	1.23	2.36	2.6	0.67
Tm7sf3	0	1.2	0	1.05	0
Tm7sf4	0.33	NA	NA	NA	0
Tm9sf1	0	1.39	1.01	1.92	0

Tm9sf2	0	0.01	2.32	1.33	1.45
Tm9sf3	NA	0.12	1.15	1.04	0
Tm9sf4	0.45	0.86	6.11	1.6	0
Tma16	NA	2.57	1.61	2.06	NA
Tma7	NA	1.15	2.1	0	NA
Tmbim1	NA	1.59	2.16	2.78	2.01
Tmbim4	NA	0.04	1.43	1.7	3.83
Tmbim6	NA	1.32	1.02	1.33	0.26
Tmc1	0	NA	0.03	1.45	NA
Tmc2	0	NA	3.32	0.11	NA
Tmc3	2.26	NA	NA	9.83	NA
Tmc4	0	NA	1.62	2.32	0.99
Tmc5	1.6	1.9	0.47	0	7.79
Tmc6	0.15	0	1.8	0.93	0
Tmc7	0	2.01	0.31	0	1.32
Tmc8	NA	NA	0	1.22	3.27
Tmcc1	NA	1.39	0.6	5.14	1.94
Tmcc2	NA	2.48	1.29	2.48	3.15
Tmcc3	NA	NA	2.31	2.17	0
Tmco1	NA	1.98	0	0	0
Tmco2	NA	NA	1.65	3.72	2.48
Tmco3	NA	0	0.29	1.03	0
Tmco4	NA	NA	0.15	5.86	0
Tmco5b	NA	NA	NA	NA	0
Tmco6	NA	0	0.74	0.45	1.84
Tmco7	NA	NA	NA	NA	6.59
Tmed1	NA	3.08	1.33	0.74	2.89
Tmed10	NA	1.73	1.35	0.04	3.25
Tmed2	NA	1.03	3.97	0.96	0
Tmed3	NA	0.98	0.76	0.94	6.02
Tmed4	NA	NA	0.43	0.06	0
Tmed5	NA	0.21	0.43	0.77	1.83
Tmed6	NA	NA	0	0.71	0
Tmed7	NA	0.99	1.98	NA	0
Tmed8	NA	NA	0	0.14	1.37
Tmed9	NA	1.5	0.51	1.78	0
Tmeff1	1.27	0	0.58	0.64	1.26
Tmeff2	0	NA	0.27	0	1.27
Tmem1	0.07	NA	NA	NA	NA
Tmem10	0.06	NA	NA	NA	NA
Tmem100	NA	1.05	0.8	0	2.52

Tmem101	NA	NA	3.01	3.88	0
Tmem102	NA	NA	2.31	0	0
Tmem104	NA	0.86	1.71	0.03	2.03
Tmem106a	NA	NA	0.8	0.01	3.98
Tmem106b	NA	1.63	0.8	4.64	0
Tmem106c	NA	1.5	0.65	0	0.61
Tmem107	NA	NA	0	0.25	0
Tmem108	NA	NA	0.01	1.21	5.32
Tmem109	NA	1.29	0.93	0.03	0
Tmem11	NA	0	5.31	0.67	0.36
Tmem110	NA	0	1.57	4.05	2.31
Tmem111	NA	NA	NA	NA	1.26
Tmem114	NA	NA	NA	NA	0
Tmem115	NA	1.83	1.85	2.49	0
Tmem116	NA	NA	1.02	3.56	0
Tmem117	NA	NA	4.38	0	0.67
Tmem119	NA	NA	2.42	0.86	1.36
Tmem120a	NA	NA	2.6	2.42	0
Tmem120b	NA	1.01	1.51	0	0.85
Tmem121	NA	1.41	2.78	0.6	0
Tmem123	NA	0.64	0.04	0.63	0
Tmem125	NA	NA	1.39	2.85	1.23
Tmem126a	NA	NA	3.94	0.41	0
Tmem126b	NA	4.53	1.01	0.44	0.72
Tmem127	NA	0.77	0	0	0
Tmem128	NA	NA	3.08	0.02	1.51
Tmem129	NA	NA	0.19	0.96	0
Tmem130	NA	NA	0.22	0	6.34
Tmem131	NA	3.15	2.28	0.13	0
Tmem132a	NA	2.26	0.58	0.34	3.9
Tmem132b	NA	NA	NA	1.53	NA
Tmem132c	NA	NA	0.03	0.22	0
Tmem132d	NA	NA	0	0.65	4.05
Tmem132e	NA	NA	1.1	0	2.15
Tmem134	NA	0	0.65	0.43	0.51
Tmem135	NA	0.5	1.01	0	4.24
Tmem136	NA	NA	1.21	0.52	1.33
Tmem138	NA	NA	0.96	2.95	0
Tmem139	NA	NA	2.29	1.19	0
Tmem140	NA	2.15	2.16	NA	0
Tmem141	NA	NA	2.08	1.43	0

Tmem143	NA	0.62	0.83	0.79	0
Tmem144	NA	1.26	0.98	1.93	0.68
Tmem145	NA	NA	NA	1.5	NA
Tmem146	NA	NA	NA	NA	0
Tmem147	NA	2.16	2.44	0.1	0
Tmem149	NA	NA	NA	NA	0.54
Tmem14a	0.84	0	1.94	1.44	2.74
Tmem14c	0.52	NA	1.57	0.22	0
Tmem150a	NA	NA	0.98	2.45	0.77
Tmem150b	NA	NA	1.57	NA	NA
Tmem150c	NA	NA	0.94	NA	NA
Tmem151a	NA	NA	3.09	1.01	2.18
Tmem151b	NA	0.16	0.43	2.12	0
Tmem154	NA	NA	1.98	1.01	0
Tmem158	NA	0.62	8.09	1.52	0
Tmem159	NA	0.89	2.31	0.72	0.89
Tmem160	NA	1.33	5.5	2.23	0
Tmem161a	NA	0	1.72	2.69	1.57
Tmem161b	NA	NA	1.38	0.67	3.27
Tmem163	NA	NA	4.85	1.52	NA
Tmem164	NA	3.33	1.52	2.8	2.38
Tmem165	NA	0	7.42	0.38	0
Tmem167b	NA	NA	2.58	1.46	1.59
Tmem168	NA	0.82	2.01	0.13	1.56
Tmem169	NA	NA	2.32	1.41	0.44
Tmem16b	0.04	NA	NA	NA	NA
Tmem16d	0.6	NA	NA	NA	NA
Tmem16f	0	NA	NA	NA	NA
Tmem17	1.2	NA	0.23	0.42	0
Tmem170b	NA	NA	2.76	NA	6.21
Tmem171	NA	NA	1.34	0.93	0.87
Tmem173	NA	NA	1.76	2.52	0
Tmem174	NA	NA	1.06	2.47	0
Tmem175	NA	NA	1.86	1.22	0
Tmem176a	NA	2.74	2.22	1.07	3.12
Tmem176b	NA	3.28	2.17	12.11	3.68
Tmem177	NA	0	0.81	0.96	1.55
Tmem178	NA	NA	NA	NA	0
Tmem179	NA	NA	1.87	0.17	0
Tmem179b	NA	NA	0.02	NA	14.81
Tmem18	0.42	NA	2.22	3.09	1.34

Tmem180	NA	0	1.49	3.16	1.93
Tmem182	NA	NA	0.86	1.96	0.23
Tmem183a	NA	2.38	0.8	NA	0
Tmem184a	NA	NA	2.86	1.34	8.51
Tmem184b	NA	3.73	2.87	0	0
Tmem184c	NA	1.1	2	0.54	0
Tmem185b	NA	0.17	2.89	1.09	18.46
Tmem186	NA	2.11	0.95	2.61	2.62
Tmem188	NA	NA	NA	NA	0.49
Tmem189	NA	NA	1.88	NA	NA
Tmem19	0	0.8	0.74	0.01	0
Tmem190	NA	NA	7.4	1.13	NA
Tmem192	NA	NA	0.74	1.73	1.87
Tmem194b	NA	NA	1.6	0.32	0.14
Tmem196	NA	NA	NA	1.95	NA
Tmem198	NA	NA	1.09	6.18	4.12
Tmem198b	NA	NA	1.02	1.36	NA
Tmem199	NA	3.04	1.9	0.89	0
Tmem2	0.24	0.5	0.98	3.11	0.31
Tmem20	0.5	NA	NA	NA	NA
Tmem200a	NA	NA	0.61	1.19	0
Tmem200c	NA	NA	0	NA	NA
Tmem201	NA	NA	0	1.36	1.05
Tmem202	NA	NA	NA	0	NA
Tmem203	NA	NA	1.88	0	0
Tmem204	NA	0	1.12	1.03	0.72
Tmem205	NA	NA	2.12	1.5	1.18
Tmem206	NA	1.51	0.5	0.95	0
Tmem207	NA	NA	0.45	0.99	2.25
Tmem208	NA	0	0.98	NA	1.57
Tmem209	NA	1.14	0.57	0.52	0
Tmem211	NA	NA	NA	0.08	NA
Tmem212	NA	0	2.52	1.13	1.24
Tmem213	NA	NA	1.06	NA	0
Tmem214	NA	0	1.16	0.09	1.27
Tmem215	NA	NA	NA	0.62	NA
Tmem216	NA	NA	1.06	NA	3.52
Tmem217	NA	NA	2.3	0.05	2.25
Tmem218	NA	NA	0.69	0.16	4.59
Tmem219	NA	NA	0.77	0.02	0
Tmem220	NA	NA	NA	0.84	NA

Tmem221	NA	NA	NA	0.32	NA
Tmem222	NA	1.83	0.12	0	NA
Tmem223	NA	0.15	1.17	1.52	0
Tmem225	NA	NA	3.67	3.96	1.9
Tmem229a	NA	NA	NA	0.63	NA
Tmem229b	NA	NA	0	0.15	NA
Tmem23	0.76	NA	NA	NA	NA
Tmem230	NA	1.36	3.24	4.77	NA
Tmem231	NA	0.07	0.22	0	NA
Tmem232	NA	NA	1.44	0	NA
Tmem234	NA	NA	3.61	1.04	NA
Tmem236	NA	NA	NA	2.26	NA
Tmem237	NA	NA	1.23	0.88	NA
Tmem238	NA	NA	0.84	NA	NA
Tmem239	NA	NA	2.08	1.88	NA
Tmem24	0.53	NA	NA	NA	NA
Tmem241	NA	NA	0.45	0	NA
Tmem242	NA	0.94	1.6	0.94	NA
Tmem243	NA	0	2.1	1.54	NA
Tmem245	NA	NA	1.41	1.28	NA
Tmem246	NA	2.51	1.01	2.86	NA
Tmem247	NA	NA	2.59	0.64	NA
Tmem248	NA	1.27	2.3	0.63	NA
Tmem25	3.73	NA	3.38	0.91	0.68
Tmem251	NA	NA	NA	1.07	NA
Tmem252	NA	NA	2.99	NA	NA
Tmem255a	NA	0.67	0	2.23	NA
Tmem255b	NA	NA	NA	2.92	NA
Tmem256	NA	NA	2.8	1.09	NA
Tmem258	NA	1.19	1.34	0	NA
Tmem259	NA	0.02	1.97	0.08	NA
Tmem26	0	NA	3.11	1.46	NA
Tmem260	NA	1.76	1.99	0.24	NA
Tmem27	0	NA	1.03	1.69	0.75
Tmem30a	NA	0.3	0.93	1.08	1.75
Tmem30b	2.28	0	0.66	0.81	3.03
Tmem32	0.31	NA	NA	NA	NA
Tmem33	NA	0.82	1.28	0	1.2
Tmem34	0.24	NA	NA	NA	NA
Tmem35	0.72	0.08	2.81	0.59	0
Tmem37	NA	NA	1.45	0.43	2.17

Tmem38a	NA	NA	0	NA	0
Tmem38b	NA	0.88	0.55	0.04	4.28
Tmem39a	NA	0.52	0.95	2.14	0.72
Tmem39b	NA	2.59	0	2.58	4.6
Tmem4	0.26	NA	NA	NA	NA
Tmem40	NA	1.43	1.07	0.83	0
Tmem41a	NA	NA	2.72	3.2	0
Tmem41b	NA	0.85	2.1	0.7	0.03
Tmem42	NA	NA	2.02	NA	0
Tmem43	NA	0.29	1.46	0.5	0
Tmem44	NA	NA	1.67	0.81	0
Tmem45a	NA	0	4.96	1.55	3.28
Tmem45b	NA	NA	0.52	0.9	6.29
Tmem47	NA	0	0.32	2.31	1.84
Tmem48	NA	NA	NA	NA	0
Tmem49	NA	NA	NA	NA	0
Tmem5	0.61	2.54	2.48	NA	2.14
Tmem50a	NA	1.44	2.08	1.27	0
Tmem50b	NA	0	2.97	2.48	0
Tmem51	NA	1.63	0.49	2.14	4.11
Tmem52	NA	NA	1.89	0.3	0
Tmem52b	NA	NA	2.05	0	NA
Tmem53	NA	3.76	1.26	0	2.94
Tmem54	NA	NA	0.88	0.57	1.57
Tmem55a	NA	NA	0.52	0	0
Tmem55b	NA	NA	1.26	0	0
Tmem56	NA	NA	0.11	0.99	0.83
Tmem57	NA	0.51	1.4	0.19	1.2
Tmem59	NA	0	3.25	4.14	1.17
Tmem59l	NA	1.71	2.69	1	2.15
Tmem60	NA	NA	1.44	0.57	0
Tmem61	NA	NA	0	NA	5.4
Tmem62	NA	4.23	2.21	1.86	1.28
Tmem63a	NA	1.02	1.49	1.02	1.65
Tmem63b	NA	NA	1.3	1.93	NA
Tmem63c	NA	NA	5.12	0	2.14
Tmem64	NA	NA	1.96	0	0.77
Tmem65	NA	NA	0.82	1.22	0.28
Tmem66	NA	0	0.6	2.43	2.91
Tmem67	NA	NA	0	1.19	0.8
Tmem68	NA	NA	1.51	0.39	0

Tmem69	NA	NA	1.62	0.54	0
Tmem70	NA	0	0.3	1.5	5.94
Tmem71	NA	NA	4.66	3.11	0.38
Tmem72	NA	NA	1.87	0	NA
Tmem74	NA	NA	0.36	0	NA
Tmem74b	NA	0.61	1.79	4.63	NA
Tmem79	NA	NA	0.74	1.36	0
Tmem8	0.75	NA	NA	NA	NA
Tmem80	NA	2.26	3.45	2.58	2.03
Tmem81	NA	NA	NA	0.16	NA
Tmem82	NA	NA	NA	0.38	NA
Tmem85	NA	NA	NA	NA	0
Tmem86a	NA	NA	1.4	NA	3.71
Tmem86b	NA	NA	1.32	1.63	1.06
Tmem87a	NA	0.08	1.35	0.59	0.68
Tmem87b	NA	NA	0.73	0	1.45
Tmem88	NA	NA	1.95	NA	0
Tmem89	NA	NA	NA	0.63	NA
Tmem8b	NA	0	9.13	1.53	0.6
Tmem8c	NA	NA	NA	0.38	NA
Tmem9	0	NA	1.46	2.57	7.64
Tmem90a	NA	NA	NA	NA	0
Tmem90b	NA	NA	NA	NA	1.66
Tmem91	NA	NA	2.34	1.81	0
Tmem92	NA	NA	NA	0	NA
Tmem93	NA	NA	NA	NA	0
Tmem95	NA	NA	NA	0	NA
Tmem97	NA	0	2.7	0.69	1.05
Tmem98	NA	NA	2.37	0	0
Tmem9b	NA	1.31	1.87	1	3.45
Tmepai	0	NA	NA	NA	NA
Tmf1	NA	0.67	1.06	0	0.38
Tmie	0.19	NA	0.74	0	NA
Tmigd1	NA	NA	NA	1.44	NA
Tmlhe	0.92	1.72	1.77	1.56	0.21
Tmod1	0.73	2.21	0.6	NA	1.24
Tmod2	0.38	1.36	0.59	3.07	0.83
Tmod3	0.2	1.14	0.64	1.93	0
Tmod4	0.89	NA	2.32	NA	1.89
Tmpit	0.21	NA	NA	NA	NA
Tmpo	0.59	0.6	1.37	0	0

Tmprss11a	NA	NA	NA	2.16	NA
Tmprss11d	NA	1.29	2.85	1.21	0
Tmprss11e	NA	1.61	0	1.84	1.05
Tmprss11f	NA	NA	NA	5.03	NA
Tmprss12	NA	NA	NA	0.15	NA
Tmprss13	NA	NA	0.12	3.07	3.38
Tmprss15	NA	1.32	1.78	0.5	NA
Tmprss2	2.4	0.59	0.13	1.07	1.48
Tmprss3	0	1.02	1.89	1.11	0.9
Tmprss4	0.61	0.83	0.83	1.26	0
Tmprss5	4.2	0	0.15	0.6	0
Tmprss6	0.68	0.15	0.09	1.73	2.22
Tmprss7	0.37	NA	NA	0.14	NA
Tmprss9	NA	NA	NA	1.25	NA
Tmsb10	1.06	1.67	0.06	0.25	0
Tmsb15a	NA	0	0.45	5.33	5.38
Tmsb4x	NA	NA	NA	0	NA
Tmtc1	NA	NA	0	2.27	1.02
Tmtc2	NA	NA	1.82	13.93	0
Tmtc3	NA	NA	1.38	0.59	0.41
Tmtc4	NA	NA	1.2	1.28	1.69
Tmub1	NA	NA	0.86	1.08	2.26
Tmub2	NA	0	5.37	0.13	0
Tmx1	NA	3.72	1.84	0.8	0
Tmx2	NA	0	0.72	0.59	0
Tmx3	NA	NA	2.14	0.91	0
Tmx4	NA	0.53	1.87	0	0
Tnc	0	1.2	1.37	0.12	0
Tnf	0	0.46	1.04	1.41	0
Tnfaip1	4.21	0.79	0.38	3.17	1.97
Tnfaip2	0	2.07	1.12	2.63	0
Tnfaip3	0.05	0.53	1.9	3.12	0
Tnfaip6	0	0	2.03	1.21	0
Tnfaip8	1.01	2.69	1.64	0	4.17
Tnfaip8l1	NA	NA	1.66	0.05	0
Tnfaip8l2	NA	NA	2.25	1.68	0
Tnfaip8l3	NA	NA	NA	0.06	NA
Tnfrsf10b	0	0.7	0.88	0.59	0
Tnfrsf11a	0	1.19	1.05	0.85	2.76
Tnfrsf11b	1.13	0.62	0.82	1.57	1.5
Tnfrsf12a	0.85	0.76	0.83	3.7	1.79

Tnfrsf13b	1.7	1.38	3.03	0.95	0.65
Tnfrsf13c	0.24	NA	2.21	0	NA
Tnfrsf14	1.61	1.26	3.53	1.02	1.23
Tnfrsf17	8.91	1.06	2.76	0.72	1.14
Tnfrsf18	1.59	NA	0.26	0.93	0
Tnfrsf19	2.95	NA	0.69	0	0
Tnfrsf19l	0.22	NA	NA	NA	NA
Tnfrsf1a	0.18	1.11	1.76	3.68	0
Tnfrsf1b	0.21	2.48	0.96	1.51	0.48
Tnfrsf21	1	2.26	3.05	1.14	2.52
Tnfrsf25	0.66	0	1.17	NA	0
Tnfrsf4	0	0.84	0.06	2.34	0
Tnfrsf8	0.42	2.34	1.32	0.97	0.4
Tnfrsf9	0.04	0.73	1.31	0.08	0
Tnfsf10	0.42	1.85	1.64	0	0.05
Tnfsf11	0.45	1.03	1.03	2.35	0.87
Tnfsf12	0	NA	NA	NA	NA
Tnfsf13	0	NA	NA	NA	NA
Tnfsf13b	0.67	NA	0.92	0	0
Tnfsf14	0	0.15	2.96	0.81	0
Tnfsf15	0.47	NA	NA	1.82	NA
Tnfsf18	0.42	NA	NA	2.59	NA
Tnfsf4	1.78	1.57	2.23	1.74	0.34
Tnfsf5ip1	2.25	NA	NA	NA	NA
Tnfsf8	2.25	0.01	0.53	2.06	0
Tnfsf9	0	1.46	0.83	0.86	7.61
Tnik	0	0	0.8	0	1.28
Tnip1	0	2.98	1.79	7.1	0
Tnip2	0	0.05	1.14	0.36	0
Tnip3	NA	NA	NA	1.04	NA
Tnk1	1.13	0	0	1.35	0.63
Tnk2	0	1.47	1.24	0.58	0
Tnks	0	0.04	0.53	0	0.92
Tnks1bp1	0	NA	1.84	2.23	0
Tnks2	NA	0.28	1.13	3.9	0.48
Tnmd	4.26	1.94	2.62	3.5	8.19
Tnn	2.22	0	0.95	1.64	6.67
Tnnc1	1.54	1.19	5.11	0	1.82
Tnnc2	0	0.64	0.24	0.68	2.69
Tnni1	1.03	2.44	4.01	0.95	5.14
Tnni2	0.7	0.78	2.08	1.43	3.24

Tnni3	NA	1.08	0	0.67	0
Tnni3k	0	1.47	0	NA	1.75
Tnnt1	3.15	2.44	1.51	0.27	1.33
Tnnt2	0.9	0	0.76	1.86	0
Tnnt3	0.9	0.74	1.44	0.67	0
Tnp1	0	0.78	0.75	1.89	0
Tnp2	1.99	0.95	1.07	1.35	0
Tnpo1	0	1.14	1.89	0	0
Tnpo2	1.92	0	0.89	2.61	0
Tnpo3	1.34	0.25	0.99	0	0
Tnr	0	1.4	0.98	0.1	2.49
Tnrc15	0	NA	NA	NA	NA
Tnrc18	NA	NA	1.45	0.85	0.79
Tnrc4	0.36	NA	NA	NA	NA
Tnrc6a	NA	NA	1.7	5.71	0
Tnrc6b	NA	0.04	1.08	3	1.14
Tnrc6c	NA	NA	1.77	1.45	0
Tnrc9	5.23	NA	NA	NA	NA
Tns1	NA	1.58	3.4	0	0
Tns3	NA	1.38	1.12	0	0
Tns4	NA	1.56	0.54	0.03	3.68
Tnxb	0.3	0.56	0.89	0.35	4.31
Tob1	0	0	0.63	0.13	2.62
Tob2	1.07	1.08	0.68	1.35	0
Toe1	NA	1.77	2.99	0.16	2.25
Tollip	3.53	0.41	0.66	0	0.03
Tom1	0.36	0	2.55	2.09	3.19
Tom1l1	NA	1.83	0.56	0	0
Tom1l2	0	0.94	1.2	0	0.11
Tomm20	3.4	2.13	1.11	0.64	0
Tomm20l	NA	NA	NA	0	NA
Tomm22	0.34	1.1	0	0.24	0
Tomm34	0.51	0	0.02	0.4	0
Tomm40	0	2.24	1.61	2.04	0.12
Tomm40l	NA	NA	0.51	0.97	1.51
Tomm5	NA	NA	1.54	2.64	0
Tomm6	NA	NA	NA	0.59	NA
Tomm7	0.69	NA	NA	NA	NA
Tomm70a	0	1.36	1.36	4.45	0
Tonsl	NA	3.5	3.03	3.39	NA
Top1	0.41	0	1.01	0.3	0

Top1mt	0	NA	0.52	2.58	0
Top2a	0.52	0.38	0.87	0.36	0
Top2b	0.68	0.02	0.86	0.41	0
Top3a	0.95	0.39	1.53	0.33	NA
Top3b	0	0.45	1.16	0	NA
Topaz1	NA	NA	NA	2.37	NA
Topbp1	0	0.47	1.96	0.73	4
Topors	0.31	0.61	1.74	3.41	0
Tor1a	NA	0.02	1.91	0.33	3.95
Tor1aip1	NA	0.6	1.19	1.7	0
Tor1aip2	NA	NA	0.53	0.02	0.4
Tor1b	1.25	0.54	0.68	0.08	2.58
Tor2a	0	NA	0.71	4.05	0
Tor3a	0.8	2.7	1.8	1.56	2.78
Tor4a	NA	2.89	1.48	0.95	NA
Tox	1.34	0	0.79	0.64	2.61
Tox2	NA	NA	3.18	1.54	0
Tox3	NA	1.48	3.14	0.23	1.55
Tox4	NA	0.69	1.5	1.18	0
Tp53i11	0.88	NA	NA	NA	NA
Tpbg	0.77	0.97	0.42	1.76	1.22
Tpcn1	0.32	0	1.1	1.39	0
Tpcn2	0.83	NA	0.63	0	0.19
Tpd52	1.26	2.46	1.83	0.83	2.52
Tpd52l1	0.66	0	0	0.08	0
Tpd52l2	0.03	1.02	0.31	0.88	0.68
Tpgs1	NA	NA	1.2	0.18	NA
Tpgs2	NA	0.47	0.2	0	NA
Tph1	1.84	1.03	0.55	0	0
Tph2	0.94	NA	3.07	0.23	NA
Tpi1	0.69	1.87	0.33	1.7	0
Tpk1	0.04	2.22	2.17	2.54	5.38
Tpm1	0.26	1.48	0.54	0.61	0.86
Tpm2	0	0.32	0.57	1.29	0.52
Tpm3	0	1.43	0.34	0.56	0.6
Tpm4	0.08	1.73	2.3	0.75	0
Tpmt	0.02	0	1.04	0.6	0.4
Tpo	0.97	1.43	2.05	1.07	0
Tpp1	NA	0.72	3.5	5.77	0.12
Tpp2	0	0	1.53	0.86	3.75
Tppp	NA	4.94	0.42	2.32	3.05

Tppp2	NA	NA	NA	0.7	NA
Tppp3	NA	1.16	0	1.82	2.73
Tpr	1.81	0.56	1.37	0	1.1
Tpra1	NA	3.64	1.44	1.46	4.19
Tprkb	NA	0.52	2.02	0.97	2.15
Tprn	NA	NA	2.82	1.59	NA
Tpsab1	NA	NA	NA	NA	6.64
Tpsb2	NA	0.94	0.69	0.76	0
Tpsg1	5.36	2.99	0.18	NA	7.02
Tpst1	0.41	0.04	1.78	1.26	0
Tpst2	0	0	0.42	2.84	0
Tpt1	0.04	0.7	2.62	0	0
Tpte	0	1.48	1.51	1.22	4.22
Tpx2	0.27	0.38	1.32	0.29	0
Tra2a	NA	0.66	0.61	0.04	0.36
Tra2b	NA	0.82	0.67	0.07	1.01
Trabd	NA	1.87	0.57	0.76	2.3
Trabd2b	NA	NA	0.98	0.94	NA
Tradd	0.61	0	0.93	1.32	0.19
Traf1	0.15	2.55	1.19	1.68	2.48
Traf2	3.47	2.67	0.21	0.91	0
Traf3	0.84	1.22	0.1	2.49	0
Traf3ip1	0.22	3.67	0.92	1.15	0.85
Traf3ip2	NA	0.03	2.85	0	1.48
Traf3ip3	NA	0	2.75	0	2.39
Traf4	1.46	0	1.54	1.49	0.2
Traf5	0.62	0	0.42	0	0
Traf6	0	0.26	0.89	1.31	0.06
Traf7	3.39	NA	2.42	1.23	1.64
Trafd1	NA	0.89	0.83	0.81	0
Traip	3.95	0.06	2.07	0	0.82
Trak1	NA	0.76	0.73	1.21	0.03
Trak2	NA	0.98	0.43	4.5	4.05
Tram1	0.05	1.09	2.06	1.55	0
Tram1l1	0.06	NA	1.15	3.06	0
Tram2	0	0	0.97	1.19	0
Trank1	NA	0	2.44	6.25	NA
Trap1	0.41	1.72	1.22	0	0.35
Trappc1	NA	NA	2.28	5.23	1.17
Trappc10	NA	0	2.18	0	3.43
Trappc11	NA	0	1.84	0	NA

Trappc12	NA	0	0.86	0.23	NA
Trappc13	NA	0.67	1.05	0.6	NA
Trappc2	0.4	0	2.25	0.48	3.35
Trappc2l	NA	0.58	2.15	0.42	0.92
Trappc3	0.66	0	1.2	1.28	2.96
Trappc3l	NA	NA	NA	8.25	NA
Trappc4	0.81	1.29	0.51	0.28	2.5
Trappc5	NA	NA	0.54	0.78	1.8
Trappc6a	NA	0	1.3	0.92	0
Trappc6b	0.44	NA	2.29	1.11	1.44
Trappc8	NA	0.53	0.59	5.76	NA
Trappc9	NA	0.14	1.38	0	0
Trat1	NA	0.21	3.3	1.04	0
Trdmt1	NA	0.2	0.91	0.39	2.91
Trdn	0.54	1.1	0.48	0.8	0
Treh	0.98	1.95	0.27	1.35	5.57
Trem1	0.72	4.21	0.09	1.64	0
Trem2	0.64	0.6	0.32	1.34	2.19
Treml1	1.33	NA	1.49	0.43	NA
Treml2	NA	0	1.07	1.11	0
Treml4	0.16	NA	3.54	2.05	NA
Trerf1	0.14	NA	NA	0.92	NA
Trex1	0.41	NA	NA	NA	0.19
Trex2	3.84	1.47	2.25	0.89	0
Trfp	0.85	NA	NA	NA	NA
Trh	0	1.74	2.45	2.92	0
Trhde	1.3	0.97	1.67	NA	2.15
Trhr	1.84	1.13	0.57	1.22	0
Triad3	0.39	NA	NA	NA	NA
Triap1	NA	1.95	0.57	2.13	0
Trib1	0.22	0.14	0	1.5	0
Trib2	2.23	0	0	0	1.87
Trib3	1.5	1.18	2.24	0	0.66
Tril	NA	0.82	0.53	1.04	NA
Trim10	0.48	2.47	0.85	0	4.52
Trim11	0.84	NA	1.59	1.41	0
Trim13	2.13	2.4	2.5	NA	4.22
Trim14	0.82	0.62	1.55	2.19	0
Trim15	0.21	2.03	2.71	3.89	0
Trim16	0.99	0.86	2.89	0.82	1.58
Trim17	0.59	1.49	0.91	NA	6.35

Trim2	0.74	0.05	1.4	1.52	1.08
Trim21	0	0	0	0	0
Trim23	0.07	1.15	0.99	0.89	1.01
Trim24	1.08	0.35	1.02	0	2.22
Trim25	0.84	0	1.43	0	0
Trim26	0.64	0.1	1.08	0	0.4
Trim27	0.34	1.4	0.75	0.71	1.02
Trim28	0.35	0	1.04	3.14	0
Trim29	0.06	1	1.38	0.68	15.12
Trim3	1.33	1.01	2.73	2.73	6.49
Trim31	0.06	1.59	1.4	1.65	3.7
Trim32	0	0	1.56	1.43	0
Trim33	0.71	0.97	0	0	1.41
Trim34	0.57	NA	NA	NA	NA
Trim35	0	NA	1.51	4.69	3.72
Trim36	0.36	2.1	0.1	0	2.29
Trim37	0	1.18	1.98	0.87	1.49
Trim38	NA	NA	NA	4.96	NA
Trim39	0.36	NA	2.41	NA	0.89
Trim40	NA	NA	2.24	0.57	NA
Trim41	0.33	NA	1.31	0.44	0
Trim42	2.79	NA	0.9	0.8	NA
Trim43b	NA	NA	NA	0.44	NA
Trim44	0.05	0	2.95	0.93	2.53
Trim45	0.38	1.13	0.9	1.5	0
Trim46	0	1.2	1.48	1.84	8.97
Trim47	0.02	NA	2.82	1.09	0.89
Trim5	NA	1.1	2.39	3	1.25
Trim50	1.99	NA	NA	0.45	NA
Trim52	NA	NA	NA	0.36	NA
Trim54	NA	NA	1.05	1.35	5.27
Trim55	NA	NA	NA	0.99	NA
Trim56	0.35	NA	2.11	0.51	NA
Trim59	1.91	NA	1.97	0	0
Trim6	0.44	NA	0.34	NA	0.97
Trim61	NA	NA	0.74	2.24	2.75
Trim62	NA	0	1.47	0.29	1.2
Trim63	NA	NA	0.55	0.99	2.72
Trim65	NA	NA	2.02	0	1.27
Trim66	NA	1.64	0.42	1.27	0
Trim67	NA	NA	1.25	1.1	1.67

Trim68	NA	0	0.12	0.25	2.84
Trim69	NA	NA	0.96	0	0
Trim7	1.43	NA	1.1	2.77	0.7
Trim71	NA	NA	NA	2.05	NA
Trim72	NA	NA	1.47	0.17	NA
Trim8	0.11	0	0.95	1.67	0
Trim9	0.66	1.38	1.3	1.62	0.71
Triml1	NA	NA	2.27	1.23	NA
Triml2	NA	NA	NA	1.72	NA
Trio	6.23	0.42	0.71	0	0.11
Triobp	0.79	2.73	1.02	0	0
Trip10	0	1.51	1.57	2.46	3.28
Trip11	NA	0	0.26	1.29	0.49
Trip12	0.15	0.63	1.52	0.83	0
Trip13	0	0.68	1.93	0.77	0.09
Trip4	0.69	1.96	1.67	0.1	2.37
Trip6	6.89	0	1.38	2.32	6.79
Triqk	NA	NA	0	NA	NA
Trit1	5.16	0	1.15	0.77	0
Trmt1	0	0.33	0.24	1.79	1.02
Trmt10a	NA	NA	1.05	0.74	NA
Trmt10b	NA	NA	0.71	1.37	NA
Trmt10c	NA	NA	1.82	0.79	NA
Trmt11	NA	0	1.56	0.91	2
Trmt112	NA	0.1	1.82	0	0
Trmt12	NA	1.59	1.74	0.02	4.12
Trmt13	NA	0.71	0.9	0	NA
Trmt1l	NA	1.29	0.75	0.77	NA
Trmt2a	NA	0.07	0.02	0.28	0
Trmt2b	NA	0	1.15	0	1.31
Trmt44	NA	0.65	1.55	1.44	NA
Trmt5	NA	0	0.47	0.23	3.64
Trmt6	NA	NA	0.93	1.12	0
Trmt61a	NA	1.36	1.08	3.19	0
Trmt61b	NA	1.98	2.32	0.47	3.59
Trmu	NA	1.13	1.29	2.38	2.19
Trnau1ap	NA	0	2.07	0.32	0.85
Trnp1	NA	NA	0.01	0.54	1.36
Trnt1	0.99	NA	3.25	0.42	1.42
Tro	0.41	1.16	2.4	0	3.83
Troap	1.55	1.7	2.53	0	0

Trove2	NA	1.5	0.93	0.79	0
Trpa1	NA	0.01	0.46	1.52	5.35
Trpc1	2.24	1.21	1.22	0.62	0.8
Trpc2	NA	1.35	1.14	1.11	0
Trpc3	3.58	0	1.04	0.06	1.54
Trpc4	0.49	1.02	1.93	0.09	1.03
Trpc4ap	0.14	1.31	1.57	0	1.45
Trpc5	3.18	2.05	0.87	2.46	2
Trpc6	0.27	1.38	2	0.46	0
Trpc7	1.64	1.94	0	2.76	0
Trpm1	0.12	0.5	0.78	0.34	2.3
Trpm2	3	0	1.13	1.39	0
Trpm3	0.52	0.22	0.22	0.42	0
Trpm4	0.85	2.25	1.32	0.12	1.18
Trpm5	0.3	NA	1.3	0	1.97
Trpm6	9.69	0.52	0.77	4.11	0
Trpm7	1.32	NA	1.99	0.35	0.38
Trpm8	3.39	0.3	0.61	1.99	0
Trps1	1	0.13	0.03	1.86	0.58
Trpt1	NA	NA	2.8	1.21	2.05
Trpv1	NA	2.46	0	NA	2.53
Trpv2	0.27	1.59	3.01	1.34	0
Trpv3	2.29	NA	NA	2.63	NA
Trpv4	0.65	2.46	2.13	0.77	5.43
Trpv5	0.1	NA	NA	0	NA
Trpv6	2.62	0.26	1.42	0.75	0
Trrap	NA	0	0.82	0	1.75
Trub1	NA	NA	0.86	1.09	0.54
Trub2	NA	NA	0.94	NA	0
Tsacc	NA	NA	3.76	3.26	NA
Tsc1	0.43	0	1.89	0	2.45
Tsc2	0	1.06	1.93	0.89	2.96
Tsc22d1	NA	0.51	0.27	1.75	0.56
Tsc22d2	NA	0.76	0.83	3.04	1.75
Tsc22d3	NA	0	0.94	1.75	3.32
Tsc22d4	NA	1.42	2.91	1.78	4.44
Tscot	1.04	NA	NA	NA	NA
Tsen15	NA	NA	1.25	0.15	0
Tsen2	NA	0.9	0	1.99	0.29
Tsen34	NA	0.08	1.01	0.73	0
Tsen54	NA	NA	1.56	1.15	0

Tsfm	NA	0.82	1.28	1.16	1.15
Tsg101	1.26	3.86	2	1.6	0
Tsga10	0.6	0.88	1.76	1.34	0
Tsga10ip	0.43	NA	NA	NA	NA
Tsga13	0.52	NA	2.58	0.79	NA
Tsga14	0.37	NA	NA	NA	1.28
Tsga2	0.71	NA	NA	NA	NA
Tshb	6.66	2.09	1.43	0.98	0
Tshr	0	0.59	0.83	0	1.5
Tshz1	NA	NA	0.44	1.97	0
Tshz2	NA	1.1	0.76	1.48	0.65
Tshz3	NA	NA	0.26	0.23	2.84
Tsix	NA	NA	3.29	NA	3.12
Tsks	NA	1	0.4	0.99	0
Tsku	NA	1.09	1.77	1.5	0
Tslp	1.1	NA	3.3	0.43	1.27
Tsn	0.62	0.48	0.19	0.46	0
Tsnax	0.21	0.79	0.75	NA	0
Tsnaxip1	0.82	2.07	0	1.92	0
Tsp50	3.46	NA	NA	NA	NA
Tspan1	NA	1.35	0.11	0.34	0
Tspan10	NA	NA	1.95	1.58	4.37
Tspan11	NA	NA	0.34	0	0.53
Tspan12	NA	2.14	2.55	1.44	1.25
Tspan13	NA	1.66	0.79	1.86	0
Tspan14	NA	1.52	2.18	0	1.31
Tspan15	NA	1.71	1.97	1.68	3.5
Tspan17	NA	NA	2.76	1.66	1.59
Tspan18	NA	NA	0.39	1.82	0.53
Tspan2	NA	2.38	0.05	1.22	1.46
Tspan3	NA	2.14	2.88	0.27	0
Tspan31	NA	0.66	1.71	0.72	0
Tspan32	NA	0.04	3.14	2.33	2.29
Tspan33	NA	NA	1.8	1.69	0.02
Tspan4	NA	0	2.21	2.27	0
Tspan5	NA	1.73	0.69	0.44	2.66
Tspan6	NA	0.16	0.58	0.17	0.57
Tspan7	NA	0.58	0.15	1.21	2.13
Tspan8	NA	1.36	1.16	1.15	1.33
Tspan9	NA	1.86	1.44	6.7	0.52
Tspear	NA	NA	NA	0.73	NA

Tspo	NA	0	0.46	0.92	0
Tspo2	NA	1.42	1.17	0.91	NA
Tspyl1	0.04	0	0.45	0.47	0
Tspyl2	NA	1.39	1.96	0.1	0
Tspyl3	NA	NA	NA	NA	1.09
Tspyl4	NA	0.08	1.89	0.94	0
Tspyl5	4.44	6.15	1.31	1.42	0
Tsr1	NA	1.44	0.43	NA	0.77
Tsr2	NA	2.43	0.77	1.39	1.61
Tsr3	NA	0	1.86	0.54	NA
Tsrc1	0.21	NA	NA	NA	NA
Tssc1	0	1.33	0.2	0.33	0.19
Tssc4	0	3.02	4.54	0.63	0
Tssk2	NA	0.81	2.19	0.43	0
Tssk3	NA	NA	0.74	0.42	1.57
Tssk4	NA	NA	0.02	1.1	1.2
Tssk6	NA	NA	1.02	0.59	0
Tst	1.05	0.52	1.16	0.77	2.02
Tsta3	0.08	1.9	0.71	0.97	1.4
Tstd1	NA	NA	0	NA	1.78
Tstd2	NA	2.64	0.95	0.92	0
Ttbk1	0	NA	0	0.89	1.63
Ttbk2	NA	1.69	0.23	0.17	0.13
Ttc1	0.69	1.22	0.66	0.48	4.59
Ttc12	0	1.82	1.31	0	2.2
Ttc13	0.48	1.4	0.91	0.99	2.18
Ttc14	0.12	NA	0.62	0.31	1.06
Ttc15	0	NA	NA	NA	1.93
Ttc16	0.24	NA	0.64	0.07	0
Ttc17	1.01	1.94	1.08	0	0
Ttc18	0.26	1.14	0.7	0.03	2.2
Ttc19	0.37	0.67	1.12	0.52	3.9
Ttc21a	NA	NA	0.76	0	0.18
Ttc21b	NA	0.32	3.55	1.06	1.84
Ttc22	NA	0.28	0.71	2.54	0
Ttc23	NA	0	1.15	0.59	0
Ttc23l	NA	NA	2.34	1.31	0
Ttc24	NA	NA	NA	0.82	NA
Ttc25	NA	NA	3	0.23	7.42
Ttc26	NA	1.22	2.28	0.63	5.1
Ttc27	NA	0	1.27	0.74	0.93

Ttc28	NA	0	0.73	0.98	0.25
Ttc29	NA	NA	2.55	0.92	0
Ttc3	0.19	0	1.1	2.05	0
Ttc30b	NA	NA	0.99	2.47	2.46
Ttc32	NA	NA	0.2	0	3.02
Ttc33	NA	2.08	1.39	1.97	1.56
Ttc35	NA	NA	NA	NA	0
Ttc36	NA	NA	1.41	1.58	1.64
Ttc37	NA	1.55	1.84	0.82	0
Ttc38	NA	2.39	3.26	0.45	0
Ttc39a	NA	1.62	2.05	2.37	0
Ttc39b	NA	NA	11.17	2.02	3.17
Ttc39c	NA	NA	2	2.31	2.34
Ttc4	0	0	1.22	0.13	0
Ttc5	0	NA	1.28	0.2	5.58
Ttc6	NA	NA	1.85	NA	NA
Ttc7b	1.16	NA	1.89	1.99	2.09
Ttc8	0	NA	1.68	0.13	0
Ttc9	NA	1.15	1.31	0.86	0
Ttc9b	NA	NA	1.36	2.2	2.78
Ttc9c	NA	NA	0.72	1.76	0.07
Ttf1	0	0	0.52	3.28	2.95
Ttf2	0.72	0.14	0.73	3.42	0
Tti1	NA	0	1.93	1.09	NA
Tti2	NA	0.85	0.9	NA	NA
Ttk	0	1.2	1.68	0.31	0
Ttl	0.81	NA	0.9	0.89	0
Ttll1	0.53	0	2.09	1.63	0.77
Ttll10	NA	NA	0	1.07	0
Ttll11	NA	NA	0.21	0.57	0.47
Ttll12	NA	0.06	1.24	3.9	1.39
Ttll13	NA	NA	0.56	2.56	NA
Ttll3	NA	1.79	1.37	NA	0.08
Ttll4	0	0.95	0	2.12	0
Ttll5	NA	0.63	1.1	0.64	0.15
Ttll6	NA	NA	1.05	1.15	1.84
Ttll7	NA	1.16	0.35	0.49	1.11
Ttll9	NA	NA	0.52	2.94	2.85
Ttn	0.32	1.27	0.24	0	1.22
Ttpa	2.13	2.14	3.02	1.44	3.36
Ttpal	NA	0.09	1.16	0	0.85

Ttr	0	0.6	0.9	0.49	10.62
Ttrap	0	NA	NA	NA	NA
Ttyh1	0.79	0.61	1.05	0	0
Ttyh2	0.85	NA	0	1.08	0
Ttyh3	0.8	NA	2.46	1.54	0
Tub	0.48	1.58	0.47	NA	2.49
Tuba1a	NA	1.92	1.19	0.3	0.09
Tuba1b	NA	1.7	0.64	0.47	0
Tuba4a	NA	1.27	1.74	0.84	0.05
Tuba8	0.61	3.09	2.88	2.93	0
Tubal3	NA	NA	NA	0.93	NA
Tubb1	NA	0	0.91	0.24	0
Tubb2a	NA	0.01	1.22	0.63	0.76
Tubb2b	NA	1.2	1.36	0	0.71
Tubb2c	NA	NA	NA	NA	0.49
Tubb3	0.34	1.09	0	2.34	0.87
Tubb4	0.39	NA	NA	NA	3
Tubb4a	NA	1.69	0.7	0.53	NA
Tubb4b	NA	0.18	1.29	0	NA
Tubb6	NA	1.06	0.29	0.66	0.82
Tubd1	0.67	2.6	0.75	0	0
Tube1	0	NA	0.25	NA	1.28
Tubg1	0.09	0.85	0.89	1.37	1.68
Tubg2	0	0	0.67	0.56	NA
Tubgcp2	0.71	0.67	2.22	0.72	3.11
Tubgcp3	1.41	0	2.7	0	4.44
Tubgcp4	NA	0	1.81	0.18	0
Tubgcp5	0	0	0.12	0.88	0.29
Tubgcp6	0.58	NA	1.57	1.09	1.93
Tufm	0.26	0.03	0.7	3.81	0.09
Tuft1	0.22	0.21	1.24	0.39	2.32
Tug1	NA	1.01	1.08	1.41	1.87
Tulp1	0.5	2.41	1.26	0.64	3.85
Tulp2	2.28	1.59	0.94	1.18	0
Tulp3	0.62	0	0.1	0.41	1.15
Tulp4	0	1.18	1.31	1.24	0
Tusc1	0.19	NA	1.02	1.48	0.46
Tusc2	1.86	2.72	0.87	NA	0
Tusc3	0.07	1.95	2.63	0	4.54
Tusc4	0	NA	NA	NA	NA
Tusc5	NA	NA	0.6	1.13	0

Tut1	NA	1	2.33	1.44	0
Tvp23a	NA	NA	2.21	2.07	NA
Tvp23b	NA	1.37	2.49	NA	NA
Twf1	NA	2.04	1.38	0.64	0
Twf2	NA	0.32	2.53	8.18	0
Twist1	2.09	0.92	1.22	0.9	1.03
Twist2	0.87	NA	1.99	3.17	4.34
Twistnb	0.34	1.16	2.04	3.03	2.15
Twsg1	0.7	0.88	0.63	0.06	0
Txk	1.22	0	1.73	0	4.46
Txlna	NA	1.06	1.36	0	0
Txlnb	NA	NA	0.75	1.08	1.04
Txlng	NA	0.13	0.75	1.34	NA
Txn2	0.15	1.67	0.46	2.48	0
Txndc1	0.62	NA	NA	NA	NA
Txndc11	NA	NA	0.16	0.85	2.65
Txndc12	NA	NA	1.89	0	0
Txndc15	NA	0.87	1.59	0.64	0
Txndc16	NA	NA	2.01	0	0
Txndc17	NA	NA	2.13	0.73	0
Txndc2	0	NA	0	2.55	0.68
Txndc3	0	NA	NA	NA	4.14
Txndc4	0.97	NA	NA	NA	NA
Txndc5	0.44	0	0.56	NA	0
Txndc8	0.98	NA	3.59	6.92	NA
Txndc9	0.36	0.23	1.7	0	1.83
Txnip	0.98	0	2.56	0.54	1.13
Txnl1	0	0.24	1.03	0.79	0
Txnl2	0.49	NA	NA	NA	NA
Txnl4a	NA	0.49	0.56	0.76	2.59
Txnl4b	0.77	0.08	2.49	0.45	11.9
Txnl5	1.57	NA	NA	NA	NA
Txnl6	0.74	NA	NA	NA	NA
Txnrd1	3.74	1.33	0.56	0.49	3.8
Txnrd2	0	1.61	1.17	0.61	0.93
Txnrd3	0	0.89	0.72	NA	1.31
Tyk2	0.83	0.67	1.92	0.68	0.59
Tyms	0.2	0.6	0.46	NA	0.69
Tyr	2.84	0.95	0	0	0
Tyro3	0.91	1.32	2.7	2.02	NA
Tyrobp	1.34	0.29	0.68	NA	0

Tyrp1	0.55	0.38	0.42	0.05	2.32
Tysnd1	0	NA	0.33	1.33	0
Tyw1	NA	0	2.63	1.14	0
Tyw3	NA	NA	0.37	0.18	0
Tyw5	NA	NA	2.2	0	NA
U2af1	0	1.66	1.81	0.54	0.22
U2af1l4	NA	NA	1.66	2.07	NA
U2af2	0.09	1.62	0.19	0.85	1.42
U2surp	NA	1.11	1.09	1.23	NA
Uaca	1.08	NA	0.19	2.3	0
Uap1	0.86	1.44	2.86	1.42	NA
Uap1l1	0.02	0.53	1.34	0.71	0
Uba1	NA	0.32	0.54	0	0.05
Uba2	NA	2.03	1.02	0.7	0
Uba3	NA	0.3	2.46	1.01	1.28
Uba5	NA	1.64	2.33	0.57	2.84
Uba52	0.13	0	1.14	0	0
Uba6	NA	0.79	1.08	0.49	0.99
Uba7	NA	0	1.84	0.6	1.8
Ubac1	NA	2.07	0.59	0.8	0
Ubac2	NA	NA	0.33	0.93	2.72
Ubadc1	0	NA	NA	NA	NA
Ubald1	NA	NA	1.49	0	NA
Ubald2	NA	NA	3.11	NA	NA
Ubap1	0.1	1.25	0.79	0.87	0
Ubap2	0.12	0	3.02	1.69	0
Ubap2l	0.17	1.14	2.42	1.46	0.91
Ubash3a	0.7	0	0.93	0.76	0
Ubash3b	NA	NA	2.67	2.19	1.25
Ubb	1.33	NA	NA	NA	NA
Ubc	NA	0.61	1.71	1.84	2.11
Ubd	1.12	0.9	4.67	1.46	NA
Ube1dc1	0.83	NA	NA	NA	NA
Ube1l	0	NA	NA	NA	NA
Ube2a	3.24	0	3.26	1.41	0
Ube2b	0.27	1.56	0.59	1.21	1.28
Ube2c	0.3	2.27	7.57	8.59	1.39
Ube2cbp	NA	NA	NA	NA	1.58
Ube2d1	0	2.35	0	0	0
Ube2d2	0.32	NA	NA	NA	0.65
Ube2d3	0.63	1.75	1.67	1.03	0

Ube2e1	0.07	1.52	2.52	NA	2.69
Ube2e2	0.23	NA	0.21	0.91	2.98
Ube2e3	0.63	0	2.45	NA	0
Ube2f	NA	NA	2.07	0.8	0.05
Ube2g1	0.79	0.42	2.35	0	3.33
Ube2g2	0.09	2.12	0.32	1.04	0
Ube2h	0	2.88	1.42	0	0
Ube2i	0	0	0.93	0.01	0.66
Ube2j1	0.01	0.83	0.76	0.81	0
Ube2j2	0.5	NA	2.44	2.07	1.59
Ube2k	NA	0	1.1	0.4	0
Ube2l3	3.02	0.83	0.89	0	0
Ube2l6	0.22	1.54	2.26	0	1.66
Ube2m	0	1.09	1.06	1.1	0
Ube2n	0	1.09	0.26	0.86	0
Ube2o	NA	0.44	1.94	1.44	1.89
Ube2q1	NA	0	0.55	0.98	1.68
Ube2q2	NA	NA	3.13	1.89	0
Ube2ql1	NA	NA	0.73	NA	NA
Ube2r2	2.97	NA	1.11	0.84	0
Ube2s	NA	1.57	2.78	2.48	2.43
Ube2t	NA	NA	2.11	3.02	2.34
Ube2u	NA	NA	3.64	0	0
Ube2v1	0.06	NA	NA	1.3	NA
Ube2v2	0	0.98	1.33	0.65	0
Ube2w	NA	0.79	0.85	0.12	2.79
Ube2z	NA	0.93	1.44	0	0.8
Ube3a	0.4	0.1	1.19	0.11	0
Ube3b	0	1.27	1.81	0.98	0
Ube3c	1.13	0.29	0.19	2.4	1.09
Ube4a	0	0.69	0.81	0.3	1.43
Ube4b	0	0	1.3	0	0.54
Ubfd1	NA	1.75	1.2	NA	0.62
Ubiad1	NA	0	0	1.44	0
Ubl3	0.34	1.03	2.52	2.37	0
Ubl4a	NA	NA	NA	0.28	NA
Ubl4b	NA	NA	0.85	2.24	NA
Ubl5	0.57	1.59	1.07	0	0
Ubl7	NA	NA	0.5	1.69	0
Ublcp1	NA	NA	2.4	0.48	3.39
Ubn1	0	0	1.37	0	0.88

Ubn2	NA	NA	0.96	1.46	0.51
Ubox5	NA	0.71	2.02	NA	1.28
Ubp1	0	0	2.92	1.73	2.25
Ubqln1	0.1	NA	1.66	1.11	2.39
Ubqln2	0.27	1.12	0.86	0.32	0.92
Ubqln3	NA	1.61	0	2.23	5.06
Ubqln4	NA	2.46	1.04	1.97	0.57
Ubqlnl	NA	NA	0.85	2.39	0.79
Ubr1	0.83	NA	0.79	0	0
Ubr2	NA	1.26	0.8	0	1.11
Ubr3	NA	NA	1.08	0	0
Ubr4	NA	2.37	0.79	NA	0
Ubr5	NA	0.21	1.42	0	3.12
Ubr7	NA	0	1.73	1.15	2.63
Ubtd1	0	0	1.96	0.43	0
Ubtd2	NA	NA	1.86	0.75	0
Ubtf	0.77	0	0.17	4.6	0
Ubtfl1	NA	NA	NA	0.85	NA
Ubxd1	0.92	NA	NA	NA	NA
Ubxd2	2.69	NA	NA	NA	NA
Ubxd3	0.93	NA	NA	NA	NA
Ubxd4	0.88	NA	NA	NA	NA
Ubxn1	NA	1.17	4.48	0.1	0
Ubxn10	NA	NA	0.59	2.05	2.21
Ubxn11	NA	NA	0	0.08	2.81
Ubxn2a	NA	NA	2.57	0.95	1.47
Ubxn2b	NA	1.69	0.69	0.81	2.67
Ubxn4	NA	0	2.23	0.55	0.36
Ubxn6	NA	0.75	0.96	0.88	0.84
Ubxn7	NA	0.71	1.07	0	0
Ubxn8	NA	1.98	2.26	1.06	1.87
Uchl1	0.41	2.12	1.28	0.32	0
Uchl3	0	NA	NA	0	NA
Uchl5	0.46	1.09	0.33	0.64	1.78
Uck1	0.33	NA	2.72	0.39	0
Uck2	NA	1.54	1.1	0.94	0
Uckl1	0	0	1.96	1.77	0.72
Ucma	NA	NA	1.91	0.56	0.64
Ucn	1.53	1.42	4.73	NA	0
Ucn2	0	NA	0.34	0.36	2.03
Ucn3	0.09	NA	2.98	1.9	NA

Ucp1	1.38	0.79	0.67	1.26	1.54
Ucp2	0	0	0.84	5.96	0
Ucp3	0.95	0.82	0.62	0.26	2.73
Uevld	NA	1.06	2.57	1.2	1.64
Ufc1	NA	1.83	2.25	1.9	0
Ufd1l	0	0	3.66	0.88	2.16
Ufl1	NA	1.35	1.22	0.2	NA
Ufm1	NA	1.44	1.66	0.58	0
Ufsp1	NA	NA	0.09	0.4	0.9
Ufsp2	NA	2.41	1.79	1.22	0
Ugcg	0.58	2.1	2.04	0.05	0
Ugcgl1	0.61	NA	NA	NA	NA
Ugdh	1.05	2.19	2.09	0.56	1.45
Uggt1	NA	0.9	0.31	0	0
Uggt2	NA	1.2	0.43	0	0.62
Ugp2	0.83	0	1.75	0.09	0
Ugt1a1	2.82	1.36	1.87	NA	NA
Ugt1a10	3.73	NA	NA	NA	NA
Ugt1a5	2.31	NA	NA	NA	NA
Ugt1a6	0	NA	NA	NA	NA
Ugt1a9	0.33	NA	NA	NA	NA
Ugt2a1	0.85	NA	NA	NA	NA
Ugt2a2	NA	3.07	0.66	0.84	NA
Ugt2a3	NA	2.67	0.48	1.37	1.35
Ugt3a1	NA	NA	0.38	0	2.69
Ugt3a2	NA	NA	NA	0.89	NA
Ugt8	2.1	NA	NA	NA	NA
Uhmk1	NA	NA	0.52	0.02	5.57
Uhrf1	0.03	NA	2.08	0	0.4
Uhrf1bp1	NA	NA	0.75	1.39	0.49
Uhrf1bp1l	NA	0.05	0.66	NA	0.23
Uhrf2	0.76	NA	1.14	0.6	0
Uimc1	NA	3.73	1	0.55	0.73
Ulbp1	NA	0.12	2.21	NA	0
Ulk1	0.15	0.78	1.51	0	0.13
Ulk2	0.91	0.16	3.08	4.09	1.13
Ulk3	NA	NA	2.45	0	0
Ulk4	NA	1.07	1.62	0.27	0.3
Umod	2.41	0.67	0.29	1.47	1.14
Umodl1	NA	NA	1.13	2.9	NA
Umps	0.52	0.65	0.38	0.66	1.3

Unc119	1.06	1.89	1.7	1.05	2.03
Unc119b	NA	0	0.49	0	0.56
Unc13a	0	0	0.34	0	0
Unc13b	0.36	0.6	1.83	1.07	2.31
Unc13c	0	NA	0	0.38	NA
Unc13d	0.08	NA	1.75	3.34	0
Unc45a	NA	0.6	1.44	0	0.3
Unc45b	NA	NA	1.97	0	2.27
Unc50	0	0.14	0.8	1.22	2.72
Unc5a	0.75	NA	0.57	0	3.91
Unc5b	0.27	0.18	1.93	0	1.27
Unc5c	0.54	2.47	0.27	0.28	1.27
Unc5cl	1.96	NA	0.42	0.93	0.41
Unc5d	1.71	NA	1.92	0	1.99
Unc79	NA	NA	1.01	0.6	NA
Unc80	NA	NA	1.26	1.13	2.48
Unc84a	0.03	NA	NA	NA	NA
Unc93a	2.13	NA	NA	NA	NA
Unc93b1	NA	2.25	2.2	0.46	0
Uncx	NA	NA	NA	1.36	NA
Uncx4.1	0.59	NA	NA	NA	NA
Ung	0.18	2.29	1.47	5.46	1.98
Unk	NA	NA	1.38	0	1
Unkl	NA	3.05	2.35	1.45	0
Upb1	0	3.59	2.57	1.02	2.1
Upf1	NA	1.97	0.96	0	0.65
Upf2	0.03	0.68	1.13	0.94	1.26
Upf3a	0	0.92	2.04	0.96	1.83
Upf3b	0	0.55	0.9	0.41	0
Upk1a	1.5	3.24	1.49	0.44	4.69
Upk1b	0.41	0	1.98	4.93	1.13
Upk2	4.23	1.63	0.66	1.54	0
Upk3a	0.53	2.43	2.05	3.33	1.82
Upk3b	1.28	1.15	1.14	1.07	0.57
Upk3bl	NA	NA	NA	1.31	NA
Upp1	0.78	0	1.79	7.35	0
Upp2	0.33	NA	0	1.98	1.13
Uprt	NA	NA	NA	0.38	NA
Uqcc	NA	0	0	0.46	0.75
Uqcr	0.19	NA	NA	NA	NA
Uqcr10	NA	1.16	0.89	0	NA

Uqcr11	NA	1.41	2.05	2.15	NA
Uqcrb	0.22	0	0.81	0.56	0
Uqcrc1	0	1.59	1.08	6.49	0
Uqcrc2	0	1.45	0.04	NA	0
Uqcrfs1	0	0.19	3.51	0.58	0
Uqcrh	0.76	1.37	3.37	0.3	0
Uqcrq	NA	0	0.6	0.69	1.66
Urb1	NA	0.05	0.36	NA	0
Urb2	NA	2.39	0	1.65	1.19
Urgcp	NA	0.63	0.81	3.99	NA
Uri1	NA	0	1.56	0.85	NA
Urm1	NA	0.9	0.87	1.69	0
Uroc1	NA	NA	0	0.76	NA
Urod	0	1.84	0.97	0.74	0
Uros	0	0.04	0	1.21	0
Usb1	NA	0	1.14	0.24	NA
Use1	NA	0.47	1.55	0.43	2.61
Usf1	0.31	NA	1.57	1.63	0.93
Usf2	0.55	0.53	1.63	0.82	0.87
Ush1c	0.58	0.31	0.09	0.21	0
Ush1g	1.38	NA	NA	2.88	NA
Ush2a	0.72	2.63	0	2.17	1.5
Ush3a	0.91	NA	NA	NA	NA
Ushbp1	0	NA	0.38	3.08	0
Usmg5	1.89	NA	1.88	0	0
Uso1	NA	0.92	2.62	0.31	0
Usp1	0.25	1.4	1.27	0.66	2.18
Usp10	2.47	1.16	0.77	0.66	1.01
Usp11	0.28	0	0.61	0	5.84
Usp12	0.32	1.64	1.1	0.79	0
Usp13	0.23	0	0.77	0.14	0.73
Usp14	0.1	0	3.11	NA	0
Usp15	0.74	2.08	0	0.46	2.23
Usp16	2.34	0.79	2.12	0.65	1.73
Usp18	0.18	2.25	5.57	0.38	NA
Usp19	0	0	1.46	0	0.87
Usp2	0.76	0.36	2.29	1.06	0
Usp20	0.72	0	0.23	0.02	1.95
Usp21	0.12	0	1.56	0.59	14.97
Usp22	0.14	0.96	2.14	0	0
Usp24	0.2	0	1.63	3.61	0

Usp25	0.18	1.49	1.46	1.06	0.59
Usp26	4.58	NA	0.97	1.46	4.12
Usp27x	2.22	1.74	1.23	2.81	0.68
Usp28	1.46	NA	5.55	0	0
Usp29	0.52	1.38	0.64	NA	0
Usp3	0	2.03	1.03	0	2.65
Usp30	NA	NA	0.8	0	1.98
Usp31	1.46	NA	0.75	1.57	0
Usp32	NA	1.77	0.92	1.08	0
Usp33	0	1.45	0.44	1.58	0
Usp34	1.84	0.05	2.04	NA	0.81
Usp35	NA	NA	NA	2.05	NA
Usp36	0.3	0	0.24	2.18	0
Usp37	0.03	NA	1.65	1.08	1.74
Usp38	0.08	NA	1.02	0.63	0.38
Usp39	0.18	1.12	2.63	1.36	0
Usp4	1.5	0	2	0	1.11
Usp40	0.43	NA	0.06	0.72	0
Usp42	NA	NA	1.17	0.25	0.52
Usp43	NA	NA	0.38	0	2.88
Usp44	NA	NA	NA	0.86	NA
Usp45	NA	NA	0.23	0	0
Usp46	NA	0.74	0.72	1.52	0.91
Usp47	0.09	0.67	1.15	0.44	2.37
Usp48	0	0.09	0.74	0.44	0.8
Usp49	0.1	1.47	0.98	0.11	0
Usp5	0	1.92	0	4.43	0.03
Usp50	NA	NA	NA	0	NA
Usp51	NA	NA	NA	2.49	NA
Usp52	0	NA	NA	NA	NA
Usp53	1.52	1.28	1.13	0.44	0.11
Usp54	2.29	NA	0.99	1.87	0.42
Usp6nl	0.65	0.59	1.3	0.2	2.01
Usp7	0.85	0	0.29	0	1.13
Usp8	0.3	0.88	0.9	1.38	1.03
Usp9x	0.77	1	1.79	0	0.26
Usp9y	0.69	3.13	0.94	0.9	0
Uspl1	NA	0	0.56	0	3.16
Ust	0.44	0	2.28	0.11	0.69
Utf1	2.13	0.88	0.69	0.9	0
Utp11l	NA	1.39	2.02	0.41	0

Utp14a	0.02	1.45	0.63	0.81	1.71
Utp15	NA	NA	1.06	2.09	1.87
Utp18	NA	0.78	0	1.99	0.85
Utp20	NA	1	0.28	4.66	0.04
Utp23	NA	NA	0.95	2.95	2.74
Utp3	NA	1.17	0.47	2.54	0
Utp6	NA	1.28	0.46	0.64	2.09
Utrn	1.16	0	3.47	0	1.44
Uts2	0.34	1.62	0	1.94	4.57
Uts2b	NA	NA	NA	0.56	NA
Uts2r	1.44	NA	1.26	1.38	NA
Utx	0.2	NA	NA	NA	NA
Uty	0.51	1.08	0.12	0.6	0.88
Uvrag	NA	0	1.58	0.88	2.2
Uvssa	NA	NA	0.93	0	NA
Uxs1	2.55	1.38	1.24	0.67	0
Uxt	1.54	3.02	2.48	1.28	4.23
Vac14	NA	0.7	1.96	2.26	0
Vamp1	0.58	0.51	0.59	NA	0
Vamp2	0.46	0	1.37	0	0.9
Vamp3	0	0	0.95	0.84	0
Vamp4	0	0.64	3.91	0.56	2.56
Vamp5	1.56	0	0	0.18	1.22
Vamp7	NA	3.66	1.97	1.32	0
Vamp8	0.55	0.62	0.26	0	0
Vangl1	1.14	1.12	1.39	1.01	1.46
Vangl2	NA	NA	0.76	1.08	2.31
Vapa	1.26	0	1.64	0.36	0.66
Vapb	1.7	1.36	0.82	0.74	0
Vars	NA	0.03	0.51	1.4	1.19
Vars2	0.78	NA	0.69	1.15	0
Vash1	NA	0.26	0.71	2.19	1.75
Vash2	NA	1.26	1.54	0.11	1.02
Vasn	NA	NA	2	0	1.61
Vasp	1.01	1.67	0.84	2.22	0
Vat1	0.57	1.1	1.88	0.28	1.57
Vat1l	NA	NA	0.51	1.09	0.38
Vav1	0.59	0	2.26	2.19	0
Vav2	1	1.33	1.05	7.23	0
Vav3	0	0	1.53	3.17	0
Vax1	4.02	NA	NA	1.02	NA

Vax2	0.98	0.12	0.11	1.44	0
Vbp1	0.4	2.3	2.61	2.49	0
Vcam1	2.32	0.32	0.91	2.38	0
Vcan	NA	0.39	0.41	0	0.83
Vcl	0.33	0	0.31	0	0
Vcp	0	1.03	1.46	0	2.01
Vcpip1	NA	0	0.37	0.85	0
Vdac1	0.71	0.65	0.34	0.18	0
Vdac2	0.84	2.42	5.44	0.93	0
Vdac3	0	0.74	1.01	0.94	0
Vdp	0.72	NA	NA	NA	NA
Vdr	0.35	0.85	0.45	0	0
Vegfa	0.32	0.84	1.58	2.09	0.92
Vegfb	0.42	0	2.69	0.48	0
Vegfc	0.2	1.07	0	0.17	1.57
Veph1	NA	NA	1.18	0.24	12.98
Vezf1	0.26	0	1.05	0.08	1.96
Vezt	NA	1.16	0.99	0.03	1.4
Vgf	NA	2.28	0.47	1.9	0
Vgll1	1.45	0	2.32	1.52	0
Vgll2	0	NA	3.46	0.33	0.78
Vgll3	NA	1.05	0.58	1	1.23
Vgll4	0.29	0	1.48	0	0
Vhl	NA	1.28	1.62	0.14	3.88
Vil1	1.27	1.54	1.97	2.93	0
Vil2	2.53	NA	NA	NA	NA
Vill	NA	1.59	0	2.75	0
Vim	3	1.64	1.64	1.02	5.39
Vimp	NA	NA	1.85	1.79	NA
Vip	7.45	2.35	0.69	0.72	1.33
Vipas39	NA	0.2	0.68	0.69	NA
Vipr1	0	0	1.55	0.41	2.31
Vipr2	4.75	0.11	0	0.12	0
Vit	0.88	NA	0.22	1	0
Vkorc1	0.31	3.2	1.27	0.8	0
Vkorc1l1	0.1	NA	1.11	0.46	0
Vldlr	0.77	0.41	1.32	0	0.04
Vma21	NA	NA	0.74	0.09	NA
Vmac	NA	NA	NA	1.13	NA
Vmo1	NA	NA	NA	1.79	NA
Vmp1	NA	NA	1.02	NA	NA

Vnn1	1.15	1.64	1.93	4.59	1.95
Vnn3	0.11	0	2.58	10.37	0
Vopp1	NA	0.95	2.21	7.02	2.85
Vprbp	NA	0	0.9	NA	0
Vpreb1	0.32	0.8	1.09	1.18	1.86
Vpreb3	2.29	0	1.56	1.91	0.91
Vps11	4.12	0	0.99	0.56	2.24
Vps13a	NA	0.17	0	0	0
Vps13b	NA	0.86	1.3	2.18	0.05
Vps13c	1.94	0.92	0.55	0	1.6
Vps13d	5.32	0	0.57	1.14	1.12
Vps16	0	2.12	0.01	0.53	0.76
Vps18	0.51	NA	2.17	3.2	0.08
Vps24	0.48	NA	NA	NA	NA
Vps25	NA	NA	0.8	0.76	2.27
Vps26a	NA	1.1	1.36	0.95	2.34
Vps26b	NA	NA	0.46	0	0
Vps28	0.98	2.66	2.67	1.68	5.4
Vps29	0.18	NA	0.68	0	2.18
Vps33a	2.08	0.29	1	0	0
Vps33b	0	2.37	2.33	0	2.09
Vps35	0.2	0.36	0.98	0.45	0
Vps36	NA	NA	0.66	1.44	0
Vps37a	NA	NA	2.21	NA	0
Vps37b	NA	1.22	2.13	2.15	0.82
Vps37c	NA	2.55	1.44	0.83	1.93
Vps37d	NA	NA	1.16	1.27	10.07
Vps39	0	0.63	2.52	0.33	0.58
Vps41	0	1.21	0.45	0	0
Vps45	0.12	1.63	1.15	0.96	0
Vps4a	0.61	2.24	1.53	1.08	0.03
Vps4b	0.98	1.23	0.78	0.52	1.01
Vps51	NA	0	0.7	5.94	NA
Vps52	0.17	0	0.07	0.94	NA
Vps53	NA	0.93	1.44	0	1.65
Vps54	0.33	0.36	0.84	1.53	2.4
Vps72	NA	1.26	0.5	0.73	1.67
Vps8	NA	1.47	2.34	0.3	0
Vps9d1	NA	0.57	0.62	1.69	NA
Vrk1	0.01	0.66	2	1.07	2.96
Vrk2	1.14	1.19	1.51	0.9	3.11

Vrk3	NA	0.5	2.91	0.82	0.5
Vrtn	NA	0	2.17	1.45	NA
Vsig1	NA	NA	3.18	1.25	2.6
Vsig10	NA	0.89	0.52	0.07	NA
Vsig10l	NA	NA	0.79	0.75	NA
Vsig2	NA	NA	0.73	1.77	1.37
Vsig4	2.09	4.21	4.24	1.85	NA
Vsig8	NA	NA	NA	1.32	NA
Vsnl1	0.41	0.27	0.11	0.87	1.37
Vstm2a	NA	NA	0	0.22	4.05
Vstm2b	NA	NA	NA	0.65	NA
Vstm2l	NA	NA	0	1.21	2.79
Vstm4	NA	0.19	0	0.98	NA
Vsx1	0	0.13	0.33	0	1.97
Vsx2	NA	NA	NA	1.36	NA
Vta1	NA	NA	1.15	1.32	2.66
Vtcn1	NA	3.28	2.3	2.73	2.75
Vti1a	0.64	NA	0.22	0.22	1.08
Vti1b	0.02	2.07	3.35	0.79	0
Vtn	0	2.83	1.58	0.71	3.7
Vwa1	NA	0.8	0.1	1.39	3.14
Vwa2	NA	NA	NA	1.37	NA
Vwa3a	NA	NA	1	1.56	0
Vwa3b	NA	NA	0.86	0.51	NA
Vwa5a	NA	0	1.66	3.25	1.05
Vwa5b1	NA	NA	NA	1.23	NA
Vwa5b2	NA	NA	0.6	0.07	3.4
Vwa7	NA	0.47	0.02	NA	NA
Vwa8	NA	0	2.11	0	NA
Vwa9	NA	0.63	1.4	0	NA
Vwc2	NA	NA	NA	3.75	NA
Vwc2l	NA	NA	NA	2.02	NA
Vwce	NA	NA	2.12	0.27	0
Vwde	NA	NA	NA	1.51	NA
Vwf	0.9	0.02	0.89	0.61	0
Wac	0.34	0.18	1.11	0.33	0
Wapal	NA	0.85	1.09	0	0.54
Wars	0.29	0.63	1.49	0.52	0.51
Wars2	NA	0.34	0.25	0.11	0
Was	0.06	0.95	2.34	0.2	0.96
Wasf1	0.51	2.62	0.14	4.68	0.65

Wasf2	0.53	0.47	1.46	0.75	0.55
Wasf3	1.81	1.35	0.35	1.2	2.57
Wasl	0	0.51	1.72	0.66	0
Wbp1	NA	NA	NA	0	NA
Wbp11	1.41	0	0.78	0.02	0
Wbp1l	NA	0	1.53	0.58	NA
Wbp2	0	1.04	5.15	0	1.84
Wbp2nl	NA	NA	1.26	3.13	NA
Wbp4	0.97	0.13	1.14	0.35	0.26
Wbp5	0.01	0	1.6	0	1.42
Wbscr16	0.38	2.73	0.91	0.67	0
Wbscr17	0	NA	NA	0.54	NA
Wbscr18	0	NA	NA	NA	NA
Wbscr22	0.32	3	1.49	1.23	1.48
Wbscr27	1.57	NA	0.95	4.21	NA
Wbscr28	NA	NA	0.69	1.28	3.09
Wdfy1	0.17	NA	1.38	0.8	1.07
Wdfy2	0	NA	1.76	0	0
Wdfy3	0.44	0	1.16	1.26	0.02
Wdfy4	NA	NA	0.54	0.71	1.12
Wdhd1	0.37	1.14	1.46	0.05	0.09
Wdpcp	NA	NA	1.56	1.88	NA
Wdr1	0.22	0.62	0.4	2.82	0
Wdr11	NA	0	2.28	0.01	NA
Wdr12	0	0.9	0.36	1.4	1.61
Wdr13	0	3.44	2.58	0.21	0.62
Wdr16	NA	NA	0.39	1.9	0
Wdr17	0.14	NA	0	3.01	NA
Wdr18	0	1.29	0.87	1.69	2.47
Wdr19	0.46	2.33	0.92	0	0
Wdr20	0	NA	NA	NA	NA
Wdr22	0	NA	NA	NA	NA
Wdr23	0	NA	NA	NA	NA
Wdr24	NA	NA	2.25	2.97	2.99
Wdr25	NA	0.14	4.22	0.5	1.95
Wdr26	0	0.78	1.05	2.34	0.08
Wdr27	NA	NA	2.5	2.99	0
Wdr3	0	2.56	2.49	1.09	0
Wdr31	2.03	NA	0.38	0.23	0
Wdr33	NA	0.01	0.05	0.09	1.12
Wdr34	0	NA	1.5	0.06	0

Wdr35	1.93	NA	0.8	0	1.21
Wdr36	0.03	NA	0.49	0.61	4.09
Wdr37	0.84	0.95	1.4	0.39	1.35
Wdr38	NA	NA	0.23	1.62	2.29
Wdr4	0	1.26	0.41	2.54	0.95
Wdr40a	0.78	NA	NA	NA	NA
Wdr40b	1.07	NA	NA	NA	NA
Wdr41	0	0.14	1.55	1.57	0.12
Wdr42a	0	NA	NA	NA	NA
Wdr43	NA	1.33	0.04	1.23	2.27
Wdr44	0.25	1.18	0.66	0.77	0
Wdr45	NA	0.87	0.84	1.51	0.88
Wdr45b	NA	2.32	1.54	0.96	NA
Wdr45l	NA	NA	NA	NA	2.88
Wdr46	NA	0.93	1.22	1.48	0
Wdr47	NA	0.74	1.9	0.49	0.08
Wdr48	NA	0.48	0.97	NA	0.01
Wdr49	NA	NA	NA	0	NA
Wdr5	0.34	NA	0.1	2.64	2.42
Wdr52	NA	NA	NA	1	NA
Wdr53	NA	NA	1.59	0.58	0
Wdr54	NA	NA	1.23	0	3.48
Wdr55	NA	1.09	0.5	NA	0.76
Wdr59	NA	0	2.05	0	5.43
Wdr5b	0.6	0.82	1.67	1.63	0
Wdr6	NA	0	3.15	2.47	0
Wdr60	NA	3.25	0.01	0	2.38
Wdr61	NA	0.63	0.76	1.28	0
Wdr62	NA	1.21	2.04	0	0
Wdr63	NA	NA	0.42	1.39	0.32
Wdr64	NA	NA	1.96	0	NA
Wdr65	NA	NA	1.57	0.38	NA
Wdr66	NA	NA	0.59	1.92	0.55
Wdr67	NA	0	1.27	0.93	0
Wdr69	NA	NA	NA	NA	0
Wdr7	0	0.55	1.2	1.82	2.86
Wdr70	NA	0.45	0	0.43	2.2
Wdr72	NA	NA	NA	2	NA
Wdr73	NA	0	2.05	0.69	0
Wdr74	NA	2.03	1.33	1.19	0
Wdr75	NA	NA	1.41	0.47	0

Wdr76	NA	2.39	0.22	0.93	0
Wdr77	NA	2	1.66	1.09	2.08
Wdr78	NA	0.91	0.42	0.08	2.71
Wdr8	0.23	NA	NA	NA	0
Wdr81	NA	NA	1.98	0.5	0
Wdr82	NA	0	3.29	0.87	2.37
Wdr83	NA	NA	0	2.56	NA
Wdr85	NA	NA	0	NA	0
Wdr86	NA	NA	1.46	0.93	4.47
Wdr88	NA	NA	NA	3.49	NA
Wdr89	NA	NA	0.64	0.48	0
Wdr90	NA	NA	2.71	0.7	3.11
Wdr91	NA	0	0	0	0
Wdr92	NA	NA	0.38	0.64	0.68
Wdr93	NA	NA	NA	0	NA
Wdr96	NA	NA	NA	0.49	NA
Wdsub1	NA	NA	3.19	1.06	0
Wdtc1	0.26	0.64	0.78	0	0
Wdyhv1	NA	2.46	0.92	0.78	0
Wee1	0	0	0.26	0	0
Wfdc1	2.14	1.47	0.86	1.4	0
Wfdc11	NA	NA	NA	1.01	NA
Wfdc12	0.38	NA	0.59	0	NA
Wfdc2	NA	1.09	1.24	1.34	2.72
Wfdc3	NA	NA	0	1.08	0
Wfdc5	1.57	NA	1.86	5.14	3.43
Wfdc8	NA	NA	NA	4.96	NA
Wfdc9	NA	NA	NA	8.09	NA
Wfikkn1	0.46	NA	2.36	0.53	NA
Wfikkn2	0	NA	0.87	0.61	0.03
Wfs1	1.09	1.94	1.4	0	2.29
Whamm	NA	NA	0	1.67	2.1
Whsc1	1.26	0.48	1.61	0	0
Whsc1l1	0.52	1.18	0.23	1.38	2.11
Whsc2	0	NA	NA	NA	6.17
Wibg	NA	NA	3.57	3.56	0
Wif1	3.15	0	2.03	1.63	1.59
Wipf1	NA	0	0.9	2.05	0
Wipf2	NA	1.29	0.21	1.64	0.25
Wipf3	NA	NA	1.29	1.16	0
Wipi1	NA	1.82	1.12	0	0.5

Wipi2	NA	0.16	0.28	1.18	0
Wisp1	0.57	1.16	2.3	1.12	4.26
Wisp2	0.01	2.19	0.06	1.52	2.27
Wisp3	NA	0.15	0.67	2.07	0
Wiz	NA	0.38	0.09	0.8	0.5
Wls	NA	1.64	2.43	2.51	NA
Wnk1	NA	0.54	0.43	1.45	1.36
Wnk2	NA	NA	0.69	1.34	0
Wnk3	NA	NA	NA	0	NA
Wnk4	NA	NA	3.45	0.91	0
Wnt1	3.23	1.49	0	0.32	4.23
Wnt10a	0.74	NA	0.87	3.18	0.98
Wnt10b	1.88	0.56	3.15	0.94	0
Wnt11	1.17	2.47	1.62	1.39	0
Wnt16	0.04	0.2	0.7	3.78	0.39
Wnt2	1.14	2.11	0.24	1.41	3.58
Wnt2b	0.18	1.32	0.49	2.69	4.86
Wnt3	0.78	1.65	1.06	1.1	2.44
Wnt3a	1.45	NA	NA	3.04	NA
Wnt4	0.48	0.93	1.83	0	0
Wnt5a	0.43	0	1.17	0	0
Wnt5b	0.78	1.72	0.56	2.8	1.63
Wnt6	1.44	0	0	0.93	0
Wnt7a	3.71	0.44	1.65	1.2	0.95
Wnt7b	2.66	NA	1.27	1.51	3.38
Wnt8a	0	NA	0.68	0	2.97
Wnt8b	1.82	1.01	0.47	0.87	1.6
Wnt9a	0.33	NA	1.35	0.89	1.43
Wnt9b	0.02	NA	0.06	2.02	NA
Wrap53	NA	0	0.9	1.52	0.75
Wrap73	NA	1.47	0.69	0.74	NA
Wrb	0	1.22	0.4	1.08	0
Wrn	0.07	0.02	0.31	0.77	2.28
Wrnip1	0.08	1.22	1.26	0.57	0.58
Wsb1	0.27	0	0.36	0.84	2.9
Wsb2	1.78	0.05	1.71	0.79	0.5
Wscd1	NA	1.68	0.7	0.12	1.64
Wscd2	NA	0.66	1.29	0.31	0
Wt1	0.9	0.68	1.24	1.19	2.49
Wtap	0	0.74	1.82	0.24	0
Wtip	1.62	NA	2.68	1.85	1.84

Wwc1	NA	1.03	1.37	0	1.57
Wwc2	NA	1.84	1.78	2.38	1.02
Wwox	0.38	0	1.04	0.21	0
Wwp1	0	0	2.91	0.51	0
Wwp2	NA	0	1.19	2.51	0.66
Wwtr1	NA	0.51	0.61	1.35	1.31
Xab1	0.16	NA	NA	NA	NA
Xab2	0.63	0	1.5	0	1.18
Xaf1	NA	0.41	0.52	0	0
Xbp1	1	3.17	2.45	0.39	0
Xcl1	0	2.19	1.76	0.79	3.88
Xcr1	3.96	1.28	1.35	0.23	0
Xdh	0.5	3.27	1.47	0.55	0
Xiap	NA	0.67	0.79	0.02	0
Xirp1	NA	NA	0	2.02	0
Xirp2	NA	NA	NA	2.39	NA
Xist	0.15	0.2	0.42	NA	1.87
Xk	NA	2.14	1.1	1.69	0.87
Xkr4	NA	NA	0.77	0.05	0
Xkr5	NA	NA	NA	0.96	NA
Xkr6	NA	NA	1.98	0.92	0.59
Xkr7	NA	NA	NA	1.38	NA
Xkr8	NA	1.29	1.54	0.67	7.79
Xkr9	NA	NA	NA	0.86	NA
Xkrx	NA	NA	6.27	0	2.22
Xlkd1	2.07	NA	NA	NA	NA
Xpa	0	0.37	0.93	NA	3.81
Xpc	0	0	2.76	0	0
Xpnpep1	0.74	0.77	0.52	0.02	0.54
Xpnpep2	0	1.63	1.51	0.44	0
Xpnpep3	NA	2.26	0.38	1.12	1.66
Xpo1	0	0.98	0.72	0.5	0
Xpo4	1.7	0.81	0	10.87	1.41
Xpo5	1.18	NA	0.05	3.78	0
Xpo6	1.07	0	2.1	1.76	0
Xpo7	1.07	0.08	0.43	0.39	0
Xpot	NA	1.45	2.04	2.09	2.78
Xpr1	0.43	NA	1.54	0.87	0
Xrcc1	0.63	0	2.09	0.12	1.63
Xrcc2	0.75	1.36	1.89	0.69	0
Xrcc3	0	0.9	0.73	1.6	0.03

Xrcc4	0	0.01	1.97	1.48	0
Xrcc5	21.66	2.52	0.46	0.41	1.43
Xrcc6	NA	0	1.47	1.3	0
Xrcc6bp1	NA	NA	1.2	0.82	7.8
Xrn1	0	NA	0.9	1.03	1.67
Xrn2	0.45	NA	0.92	0.59	3.73
Xxylt1	NA	NA	0.05	2.43	NA
Xylb	0	0.53	4.99	1.79	2.71
Xylt1	4.06	NA	NA	1.97	NA
Xylt2	1.06	0	2.26	0.49	10.32
Yae1d1	NA	NA	1.06	1.03	NA
Yaf2	0.61	2.32	2.83	1.07	1.55
Yap1	0	1.57	0.72	1.38	1.27
Yars	0.79	3.15	0.9	0.47	2.18
Yars2	NA	1.56	3.76	1.19	0
Ybey	NA	NA	2.36	2.03	NA
Ybx1	NA	0	0.34	0	2.6
Ybx2	2.28	1.82	1	0	NA
Ybx3	NA	0.58	0.71	0	NA
Ydjc	NA	NA	3.02	NA	3.31
Yeats2	0.68	0	0.38	0	3.54
Yeats4	0.29	1.26	0.88	0.86	0
Yes1	NA	0.88	0.69	1.91	0
Yif1a	NA	2.22	2.67	1.77	0
Yif1b	NA	0.18	0.01	0.39	1.02
Yipf1	NA	0	1.42	1.95	2.22
Yipf2	NA	0.93	4.04	1.57	3.24
Yipf3	NA	0.73	1.68	4.48	2.79
Yipf4	NA	1.14	1.74	0	1.99
Yipf5	NA	1.25	2.21	1.95	0.44
Yipf6	NA	0.89	1.4	0	0
Yipf7	NA	NA	NA	0.11	NA
Yjefn3	NA	NA	2.01	0.66	2.19
Ykt6	NA	0	1.24	2.04	0
Ylpm1	NA	0	1.01	1.22	1.77
Yme1l1	0.17	0.77	1.31	1.39	0
Yod1	NA	1.85	0.84	0.49	7.14
Ypel1	1.9	0	0.46	0.91	0
Ypel2	NA	NA	1.57	0	5.9
Ypel3	0.66	NA	2.06	0.65	0.46
Ypel4	NA	NA	2.64	0.07	0

Ypel5	0.08	2.56	1.53	0	0
Yrdc	NA	1.55	2.99	2.44	3.82
Ysk4	1.48	NA	NA	NA	NA
Ythdc1	NA	1.72	0.96	0.52	0.53
Ythdc2	0	0.74	0.64	1.13	0
Ythdf1	1.37	2.03	2.18	0.83	1.74
Ythdf2	0	1.78	1.62	0	2.54
Ythdf3	0	0	1.99	0.47	2.85
Ywhab	0	1.73	1	0.12	0
Ywhae	0.1	0.92	1.89	1.1	0.21
Ywhag	0.48	NA	1.67	1.22	0
Ywhah	0	0.49	1.03	0.51	0.61
Ywhaq	0	1.91	2.22	NA	0
Ywhaz	1.11	0	4.86	1.28	1.26
Yy1	0	0	0.56	1.35	2.14
Yy2	NA	0.47	0.19	3.6	NA
Zadh1	1.29	NA	NA	NA	NA
Zadh2	0	NA	2.53	1.08	0
Zan	2.81	NA	0	1.33	3.14
Zap70	0.99	0	0.4	1.21	3
Zar1	7.06	NA	1.44	0	NA
Zar1l	NA	NA	NA	0.29	NA
Zbbx	NA	0.39	2.85	3.57	3.92
Zbed3	0	NA	2.39	3.93	0.24
Zbed4	0.25	0.04	1.87	NA	0.25
Zbed6	NA	NA	0.11	NA	NA
Zbp1	0	0.6	0.97	0.86	0.03
Zbtb1	0	1.16	1.4	0	0.06
Zbtb10	0.39	1.43	0.93	3.14	1.29
Zbtb11	1.77	1.12	1.18	0.47	1.62
Zbtb12	NA	NA	1.77	1.78	5.19
Zbtb14	NA	0.05	0.8	0.39	NA
Zbtb16	2.98	0.96	1.13	1.38	0.64
Zbtb17	1.12	1.32	1.06	2.94	1.17
Zbtb18	NA	0.01	4.4	0	NA
Zbtb2	NA	NA	1.93	0.71	1.99
Zbtb20	NA	0.44	1.44	0.94	0.82
Zbtb21	NA	NA	4.9	1.66	NA
Zbtb22	NA	0.62	0.74	1.15	0
Zbtb24	0	0.03	1.04	0.87	2.69
Zbtb25	NA	0	0.83	0	0

Zbtb26	NA	NA	1.66	1.75	0
Zbtb3	0	0.27	0.28	0	3.67
Zbtb32	NA	1.97	0	0.53	0
Zbtb33	1.39	0.78	0.46	1.48	0.83
Zbtb34	NA	NA	1.38	1.75	2.28
Zbtb37	0	NA	0.46	0.68	2.43
Zbtb38	NA	0.11	0.61	0	8.47
Zbtb39	NA	0	2.54	1.97	3.58
Zbtb4	1.39	NA	0.55	1.24	0
Zbtb40	NA	0	0.38	0	NA
Zbtb41	NA	NA	1.91	1.51	0
Zbtb42	NA	NA	1.06	0.73	0
Zbtb43	NA	1.27	0.38	0	0
Zbtb44	NA	1.77	0.68	0.95	0
Zbtb45	NA	NA	3.42	2.05	0.41
Zbtb46	NA	NA	0.04	2.53	0
Zbtb48	NA	2.83	3.65	4.92	2.65
Zbtb49	NA	NA	0.64	0.54	NA
Zbtb5	0.07	0	0.26	0.14	18.94
Zbtb6	NA	2.85	1.69	0.05	0
Zbtb7a	NA	0.67	1.27	1.82	1.15
Zbtb7b	NA	0.39	0	1.47	0
Zbtb7c	NA	0.37	0.76	1.55	0
Zbtb8	0.95	NA	NA	NA	NA
Zbtb8a	NA	NA	1.65	NA	2.82
Zbtb8b	NA	NA	4.02	0	NA
Zbtb8os	NA	NA	1.67	1.73	1.28
Zbtb9	NA	NA	1.93	0.9	1.3
Zc2hc1a	NA	0.92	0.28	1.04	NA
Zc2hc1b	NA	NA	2.22	0	NA
Zc2hc1c	NA	1.48	1.37	1.32	NA
Zc3h10	NA	NA	1.2	0	1.49
Zc3h11a	NA	0.85	1.5	0.85	0
Zc3h12a	NA	0.08	0.38	2.6	0
Zc3h12b	NA	NA	NA	0.05	NA
Zc3h12c	NA	NA	1.05	1.2	2.26
Zc3h12d	NA	NA	2.35	0	NA
Zc3h13	NA	0	0.79	0.05	0
Zc3h14	NA	0	3.76	0	4.28
Zc3h15	NA	1.03	1.7	0.78	0
Zc3h18	NA	NA	3.18	0.79	0

Zc3h3	NA	2.86	3.18	0.86	2.72
Zc3h4	NA	0	1.36	1.41	0
Zc3h6	NA	NA	1.27	1.07	1.92
Zc3h7a	NA	0	1.79	1.1	2.9
Zc3h7b	NA	0.37	1	1.21	0
Zc3h8	NA	NA	1.53	0.65	4.49
Zc3hav1	0	1.07	1.79	0	0
Zc3hav1l	NA	NA	0.94	0.34	3.69
Zc3hc1	NA	NA	0.97	2.01	3.63
Zc4h2	NA	0	4.02	0.68	0
Zcchc10	NA	0	1.07	0.05	3.44
Zcchc11	0.91	0.58	1.19	0.74	1.65
Zcchc12	6.61	NA	2.59	1.78	0
Zcchc13	NA	NA	1.21	2.79	NA
Zcchc14	0	0	0.23	1.06	0.75
Zcchc16	NA	NA	NA	0.76	NA
Zcchc17	NA	NA	1.58	0.56	0
Zcchc18	NA	NA	0.14	0.53	0.32
Zcchc2	2.81	1.54	5.16	0	0
Zcchc24	NA	0.17	0.86	0.59	2.25
Zcchc3	0.48	NA	2.11	0	0
Zcchc4	0.34	0.02	1.63	1.15	2.5
Zcchc5	0	NA	0.39	2.41	NA
Zcchc6	0.67	0.03	0.59	0.74	0.18
Zcchc7	0	NA	0.19	0	1.64
Zcchc8	1.61	0	1.37	0.95	0
Zcchc9	0.1	NA	2.42	0.8	0
Zcrb1	NA	NA	0.41	0	2.13
Zcwpw1	NA	NA	NA	1.17	NA
Zcwpw2	NA	1.57	3.77	4.19	0.88
Zdbf2	NA	NA	2.41	0.48	2.88
Zdhhc1	0.13	NA	0.41	5.82	0
Zdhhc11	0.61	0.02	1.16	0.49	0.85
Zdhhc12	0.39	NA	1.63	0.1	0.22
Zdhhc13	0.7	0	0.68	0.69	0.55
Zdhhc14	0	1.49	2.43	0.15	2.25
Zdhhc15	2.85	NA	0	0.67	NA
Zdhhc16	0.4	NA	0.97	0	3
Zdhhc17	NA	1.14	1.44	0.26	0.57
Zdhhc18	NA	1.43	0.1	1.46	0
Zdhhc19	0.97	NA	1.93	0.08	0.95

Zdhhc2	0	NA	1.99	0.77	0.73
Zdhhc20	NA	NA	1.08	0.85	0
Zdhhc21	0.28	NA	1.06	0.36	0.76
Zdhhc22	NA	NA	2.61	2.12	2.48
Zdhhc23	NA	NA	1.21	3.94	2.07
Zdhhc24	NA	0.94	0.15	0.93	0.58
Zdhhc3	0.44	0.02	0.66	0.24	0
Zdhhc4	1.31	0.38	0.48	0.57	0
Zdhhc5	1.69	NA	0.99	3.76	0.3
Zdhhc6	0.12	0.54	0.32	0.3	0.08
Zdhhc7	0.23	0.95	0.79	0.69	0
Zdhhc8	0.8	NA	2.05	0.79	0.66
Zdhhc9	1.25	NA	1.42	1.6	0.25
Zeb1	NA	0.65	0.7	0.34	1.09
Zeb2	NA	0.89	1.93	1.17	0.1
Zer1	NA	0.48	3.27	0.64	0
Zfand1	NA	0.14	1.42	0.57	4.59
Zfand2a	NA	NA	2.62	0.91	0
Zfand2b	NA	NA	2.97	1.25	4.16
Zfand3	NA	0	1.04	0.06	0
Zfand4	NA	NA	1.64	0	NA
Zfand5	NA	0.6	2.26	0	1.45
Zfand6	NA	1	1.41	1.61	0.92
Zfat	NA	NA	0	0.31	0
Zfc3h1	NA	4.14	1.37	2.65	0
Zfhx3	NA	2.01	0.51	1.42	0.94
Zfhx4	NA	1.32	1.39	3.72	0.85
Zfp1	0.67	NA	2.82	1.16	1.06
Zfp106	0	NA	NA	NA	0.9
Zfp112	NA	2.15	0.78	3.2	0.19
Zfp14	NA	NA	1.25	1.64	1.63
Zfp161	0.15	NA	NA	NA	0
Zfp2	0.15	1.48	0	2.73	1.19
Zfp28	0	NA	1.09	0.23	0.57
Zfp3	NA	NA	1.24	0	3.09
Zfp30	2.72	1.71	1.96	0	0
Zfp36	NA	1.94	1.51	1.19	0
Zfp36l1	0.07	0.67	0.8	0	0
Zfp36l2	NA	0	1.3	1.48	2.39
Zfp37	0.51	1.01	1.23	0.64	0.92
Zfp41	0.93	NA	1.87	1.74	0.37

Zfp42	0	NA	3.4	1.62	0.16
Zfp57	4.13	NA	0	1.07	0
Zfp62	NA	NA	0.93	0	1.42
Zfp64	0.89	0.02	0.02	NA	0.08
Zfp69	NA	NA	0.6	0.91	NA
Zfp82	NA	NA	1.72	1.3	1.24
Zfp90	0	NA	2.46	0.45	0
Zfp91	1.42	NA	0.72	NA	0
Zfp92	NA	NA	NA	1.11	NA
Zfpl1	0.92	1.49	1.77	6.49	1.18
Zfpm1	0.34	NA	0.71	0.83	NA
Zfpm2	0.98	1.22	0.96	2.43	0
Zfr	1.74	0.9	0.66	0.97	0.49
Zfr2	NA	1.25	1.6	0.69	1.21
Zfx	0.95	0.43	0.98	0.56	0
Zfyve1	0	NA	1.33	0.05	0.69
Zfyve16	0	0.01	1.52	1.4	0
Zfyve19	0	NA	1.14	0.91	2.91
Zfyve20	0	NA	1.94	0.88	0
Zfyve21	0	0.31	1.49	0.83	0
Zfyve26	0	0.98	0.21	1.58	1.75
Zfyve27	4.23	NA	1.7	0.43	0
Zfyve28	1.3	NA	1.24	0.43	0
Zfyve9	0.94	0.95	0	3.01	5.18
Zg16	NA	0.65	1.45	0	1.74
Zglp1	NA	NA	1.35	NA	NA
Zgpat	NA	0	0.65	1.05	NA
Zhx1	0.18	NA	1.96	NA	2.41
Zhx2	0	1.93	1.59	0	0
Zhx3	0.06	0	0.8	0.15	0.88
Zic1	2.4	0.23	0.9	0.33	1.49
Zic2	1.11	NA	1.63	0.94	1.45
Zic3	0.11	1.3	1.19	0	2.01
Zic4	1.4	0.99	2.46	0.05	1.79
Zic5	5.25	NA	1.26	0.98	NA
Zik1	0.9	NA	3.34	0.74	0.2
Zim2	1.72	2.58	0.18	NA	0
Zim3	0.23	NA	0	0.02	NA
Zkscan1	NA	0.97	1.14	0	0.03
Zkscan2	NA	NA	NA	0	NA
Zkscan3	NA	2.06	0.78	0.8	0.18

Zkscan4	NA	0.41	1.48	0	5.7
Zkscan5	NA	1	0.53	0	1.06
Zkscan7	NA	NA	NA	0.05	NA
Zkscan8	NA	1.32	0.82	1.28	NA
Zmat1	NA	NA	0.68	0.19	1.45
Zmat2	0	NA	1.58	1.03	1.99
Zmat3	NA	0	1.99	0.83	2.16
Zmat4	NA	0.89	0	1.5	3.18
Zmat5	NA	2.78	3.11	NA	1.09
Zmiz1	NA	0	0.99	1.22	0.5
Zmiz2	NA	0	2.04	1.16	2
Zmpste24	0	0.03	1.12	1.05	2.09
Zmym1	0.39	3.6	3.21	0.75	0
Zmym2	NA	1.56	1.49	0	0
Zmym3	NA	0	0.28	2.9	0
Zmym4	NA	0	1.88	0.62	1.84
Zmym5	NA	2.25	2.36	2.16	0.78
Zmynd10	2.14	3.48	0.86	5.07	0
Zmynd11	2.19	0	0.59	1.03	4.67
Zmynd12	NA	NA	0.63	0.07	0.99
Zmynd15	NA	NA	1.39	1.83	0
Zmynd17	1.43	NA	NA	NA	5.35
Zmynd19	1.58	NA	1.18	1.06	0
Zmynd8	NA	0.29	1.15	0	0
Znf142	0.29	NA	NA	NA	NA
Znf24	2.04	NA	NA	NA	NA
Znf512b	NA	0	0.13	2.45	0
Znfx1	NA	NA	0.5	0	0
Znhit1	1.16	0	0	1.31	2.39
Znhit2	0.38	1.2	1.46	1.25	1.78
Znhit3	NA	0	2.64	1.81	0
Znhit6	NA	0	1.68	1.32	0
Znrd1	0.29	NA	1.11	2.87	0
Znrf1	0.92	NA	1.76	1.17	0.44
Znrf2	NA	NA	2.56	1.72	0
Znrf3	NA	NA	1.54	2.74	NA
Znrf4	1.41	2.92	0	2.79	0
Zp1	0.74	NA	0.24	1.85	5.22
Zp2	3.44	1.79	3.61	1.33	4.32
Zp3	0	NA	NA	1.37	NA
Zpbp	0.11	0.82	0.13	0.91	2.21

Zpbp2	NA	NA	0.64	2.66	0
Zpld1	NA	NA	NA	1.83	NA
Zranb1	0	NA	2.22	0	0.94
Zranb2	NA	NA	2.46	0.91	0
Zranb3	2.34	NA	0.75	0.61	0.51
Zrsr1	NA	3.31	3.88	0	11.54
Zrsr2	NA	1	0.93	0.97	0.89
Zscan10	NA	NA	0	1.29	NA
Zscan12	NA	0.09	0.96	1.84	0.63
Zscan18	NA	0	3.17	0	0
Zscan2	NA	4.8	1.95	0	3
Zscan20	NA	NA	2.8	1.88	NA
Zscan21	NA	NA	0.95	0.18	1.49
Zscan22	NA	NA	1.07	5.62	2.68
Zscan25	NA	NA	1.73	1.19	NA
Zscan26	NA	0	2.17	0.38	NA
Zscan29	NA	NA	1.22	0.74	1.63
Zscan30	NA	NA	NA	3.24	NA
Zscan5b	NA	NA	NA	1.65	NA
Zswim1	0.4	2.2	0.18	7.64	5.16
Zswim2	0.97	NA	1.29	0.84	NA
Zswim3	0.89	NA	0.14	0.06	1.92
Zswim4	0.93	NA	2.28	1.98	0
Zswim5	1	NA	0	0.92	2.29
Zswim6	0	NA	1.46	3.1	2.04
Zswim7	NA	NA	1.53	NA	0
Zswim8	NA	1.03	1.7	0.71	NA
Zufsp	NA	NA	2.51	0.58	2.04
Zw10	3.89	0	1.41	0.1	1.25
Zwilch	NA	0.79	2.84	NA	0
Zwint	0.35	1.38	0.72	0.45	0.04
Zxda	0.42	NA	1.92	0.79	NA
Zxdb	NA	0	1.4	NA	0.82
Zxdc	NA	0.6	0.59	0.86	0
Zyg11a	NA	NA	0	2.26	0
Zyg11b	NA	NA	2.14	0	0
Zyx	0.33	0.94	0.44	1.47	0
Zzef1	0.7	0.73	1.93	0	0.49
Zzz3	0	0.8	1.56	0.69	0
