# Supplementary material for: Evidence-Based Translation for the Genomic Responses of Murine Models for the Study of Human Immunity
Source: PLoS One. 2015 Feb 13;10(2):e0118017. doi: 10.1371/journal.pone.0118017 (PMC4332676; doi:10.1371/journal.pone.0118017)
Supplement: S2 Table — (PDF) [file pone.0118017.s004.pdf]

Table S2. Genes benefited by EBT.

Symbol	Name
AP2M1	Adaptor-Related Protein Complex 2, Mu 1 Subunit
CXCL10	chemokine (C-X-C motif) ligand 10
IFITM2	Interferon Induced Transmembrane Protein 2
IFITM3	Interferon Induced Transmembrane Protein 3
IL1RN	Interleukin 1 Receptor Antagonist
LGALS38BP	Lectin, Galactoside-Binding, Soluble, 3 Binding Protein
LPIN1	Lipin 1
PSMD5	proteasome (prosome, macropain) 26S subunit, non-ATPase, 5
