# Supplementary material for: Evidence-Based Translation for the Genomic Responses of Murine Models for the Study of Human Immunity
Source: PLoS One. 2015 Feb 13;10(2):e0118017. doi: 10.1371/journal.pone.0118017 (PMC4332676; doi:10.1371/journal.pone.0118017)
Supplement: S3 Table — (PDF) [file pone.0118017.s005.pdf]

Table S3. Top 5 biological functions enriched in the benefited genes by EBT.

P-values for enrichment were calculated by Fisher's exact tests.

Category	Gene Set	P-Value
Canonical Pathways	Immune System	$6.15x10^{-28}$
	Cytokine Signaling in Immune System	$2.19x10^{-21}$
	Interferon Signaling	$6.98x10^{-15}$
	Metabolism of RNA	$1.36x10^{-13}$
	Metabolism of mRNA	$9.45x10^{-12}$
Gene Ontology Biological Processes	Bipolymer Metabolic Processes	$3.27x10^{-19}$
	Protein Metabolic Process	$5.29x10^{-15}$
	Cellular Macromolecule Metabolic Process	$4.07x10^{-14}$
	Nucleobase/side/tide and Nucleic Acid Metabolic Processes	$1.95x10^{-16}$
	Cellular Macromolecule Metabolic Process	$2.16x10^{-13}$
