# Supplementary material for: Evidence-Based Translation for the Genomic Responses of Murine Models for the Study of Human Immunity
Source: PLoS One. 2015 Feb 13;10(2):e0118017. doi: 10.1371/journal.pone.0118017 (PMC4332676; doi:10.1371/journal.pone.0118017)
Supplement: S4 Table — (PDF) [file pone.0118017.s006.pdf]

Table S4. Factors contributing to EBT.

The improvement of agreement percentages of the overall and specific responses were linearly modeled with rank correlation coefficients of genomic responses between mouse and human experiments as well as between training and test experiments. P-values were calculated against the null effects of the factors.

	Factor (rank correlation of genomic responses between …)	P-value
Overall response	Mouse and human experiments in the training set	0.004
	Mouse and human experiments in the test set	0.210
	Mouse experiments in the training and test sets	7.19×10^{-14}
	Human experiments in the training and test sets	0.002
Specific response	Mouse and human experiments in the training set	0.019
	Mouse and human experiments in the test set	6.64×10^{-4}
	Mouse experiments in the training and test sets	0.711
	Human experiments in the training and test sets	0.035
